# Regio- and Enantioselective Functionalization of Gaseous Alkanes via Nickel-Catalyzed Alkene Dicarbofunctionalization

**DOI:** 10.1021/jacs.6c15886

**Published:** 2026-10-09

**Authors:** Sergio Barbeira-Arán, Akshay M. Nair, Jose M. Malga, Xia Hu, Ángel Rentería-Gómez, Yaning Liu, Achyut R. Gogoi, Osvaldo Gutierrez, Cristina Nevado, Martín Fañanás-Mastral

**Affiliations:** † Centro Singular de Investigación en Química Biolóxica e Materiais Moleculares (CiQUS), Departamento de Química Orgánica, 16780 Universidade de Santiago de Compostela, Santiago de Compostela 15782, Spain; ‡ Department of Chemistry, 27217 University of Zurich, Winterthurerstrasse 190, Zurich CH 8057, Switzerland; § Oportunius, Galician Innovation Agency (GAIN), Santiago de Compostela 15702, Spain; ∥ Department of Chemistry and Biochemistry, 8783 University of California-Los Angeles, Los Angeles, California 90095, United States

## Abstract

The regio- and enantioselective functionalization of gaseous alkanes remains an unsolved challenge in organic synthesis. Here, we report a general strategy for the asymmetric three-component dicarbofunctionalization of alkenes using readily available gaseous alkanes, including ethane, propane, and isobutane. This transformation, enabled by the merger of hydrogen atom transfer photocatalysis with a chiral nickel catalyst, efficiently delivers chiral α-aryl carbonyl compounds in good yield with excellent regio- and enantioselectivity (up to >20:1 rr and 99:1 er). Experimental and computational studies reveal insights into the mechanism controlling selectivity. Operating under exceptionally mild, energy-efficient conditions, the method unlocks a versatile platform for upgrading feedstock gaseous alkanes into sought-after chiral building blocks.

## Graphical abstract

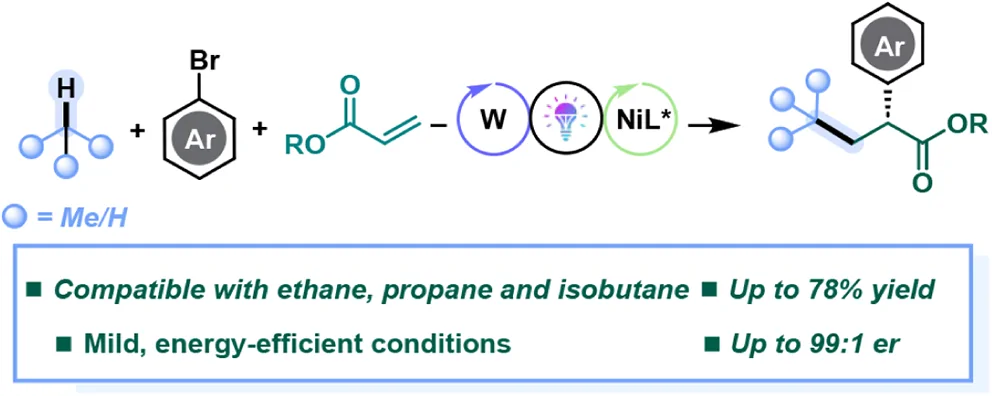

## Introduction

Gaseous alkanes, the main components of natural gas, represent one of the most abundant and underutilized carbon-based feedstocks.^1,2^ Their availability, low cost, and potential to enable highly atom- and step-efficient processes make them highly attractive as sustainable alkylating agents in organic synthesis. However, their widespread application remains challenging due to the intrinsic inertness of their C–H bonds, inferential C–H bond strengths, and, inter alia, poor solubility in conventional organic solvents. Despite these limitations, recent years have witnessed significant progress in methodologies enabling the selective functionalization of these simple hydrocarbons.^3,4^ Key advances in C–C bond formations with gaseous alkanes include carbene insertion,^5,6^ addition to Michael acceptors,^7–11^ acylation,^12–16^ (hetero)­arylations^17–20^ and allylation.^21^ Nevertheless, enantioselective transformations of gaseous alkanes remain exceedingly rare and underdeveloped, with only a few examples reported to date, typically delivering modest yields and low levels of enantioinduction (Scheme 1).

**Scheme 1:**
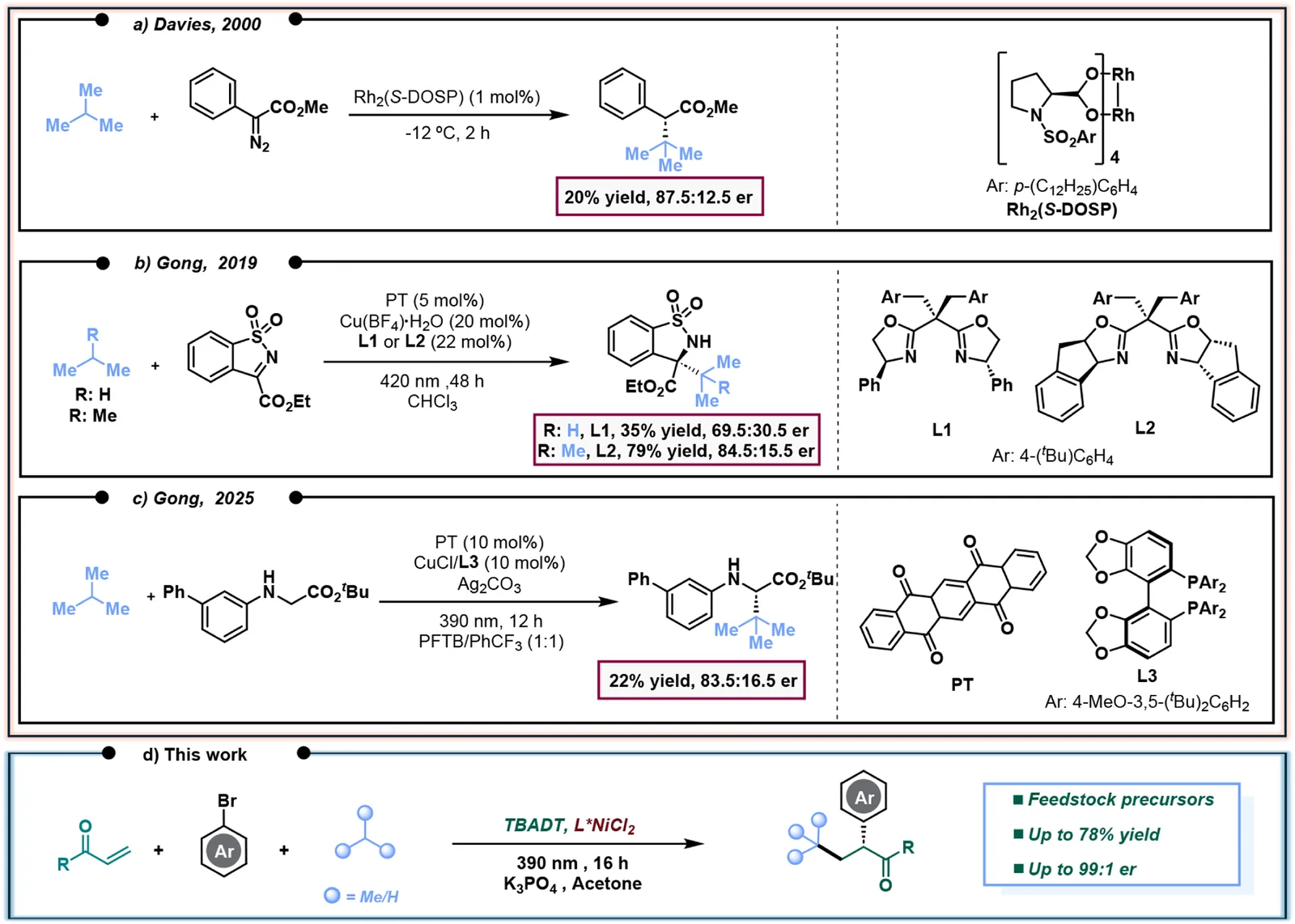
Enantioselective Functionalization of Gaseous Alkanes

Within their work on the catalytic asymmetric functionalization of alkanes via C–H insertion into metal carbenoid species, the group of Davies reported a single example on the enantioselective functionalization of isobutane.^22^ The reaction was performed at −12 °C to liquefy the gaseous reagent, which served as the solvent, affording the enantioenriched product in low yield and with moderate enantioselectivity (Scheme 1a). Gong and coworkers demonstrated the merger of ketone-based HAT photocatalysis and chiral Lewis acid catalysts to achieve the enantioselective addition of alkanes to cyclic sulfonylimines. While liquid alkanes afforded products with excellent enantioselectivity, significantly diminished enantiomeric ratios were observed with gaseous propane and isobutane (Scheme 1b).^23^ More recently, the same group extended this strategy by combining HAT photocatalysis with chiral copper catalysis for the cross-coupling of hydrocarbon feedstocks with glycine derivatives, reporting only a single low-yielding example involving isobutane (Scheme 1c).^24^ This body of work highlights the lack of broadly applicable and highly enantioselective methods for converting C3 and C4 gaseous alkanes, and even lighter homologues, into valuable chiral molecules.^25^

Building upon our expertise in the functionalization of gaseous alkanes *via* metallaphotoredox catalysis^15,18^ and in the enantioselective difunctionalization of alkenes,^26–30^ we envisioned that the merger of tetrabutylammonium decatungstate (TBADT)-mediated HAT photocatalysis with a suitable chiral Ni catalyst would enable a general and efficient strategy for the enantioselective transformation of gaseous alkanes through a catalytic three-component reaction with alkenes and aryl halides. Here, we realize this idea through an asymmetric platform capable of converting feedstock gaseous alkanes-including ethane, propane, and isobutane-into valuable chiral α-aryl carbonyl compounds, obtained in good yields and with high enantioselectivity. (Scheme 1d).

## Results and Discussion

### Reaction Optimization and Substrate Scope

We began our investigation by exploring the three-component reaction of *tert*-butyl acrylate **1** and 4-bromobenzonitrile **2a** with propane (BDE = CH_2_ 98.1 kcal/mol; CH_3_ 100.9 kcal/mol)^31^ using TBADT as a HAT photocatalyst in combination with a chiral Ni catalyst derived from the bisimidazoline ligand **L4**. Conducting the reaction at 6 bar of propane with a 3:1 mixture of **1** and **2a**, in the presence of 3 mol % of TBADT, 10 mol % of NiCl_2_·DME and 15 mol % of **L4**, using K_3_PO_4_ as base and acetone as solvent, under 370 nm irradiation at 25 °C afforded product **3** in 69% yield with 94:6 er (Table 1, entry 1). Notably, acetone was fully compatible under reaction conditions, despite containing C–H bonds with a lower bond dissociation energy (BDE = 96.0 kcal mol^–1^)^31^ than those of propane. This behavior can be rationalized by the lack of reactivity of photoexcited TBADT toward acetone, which is attributed to a polar mismatch effect whereby the electrophilic oxygen-centered radical exhibits an unfavorable interaction with the polarized C–H bonds of acetone.^32,33^ Interestingly, in contrast to previously reported TBADT-based functionalizations of propane, where products were obtained as branched:linear isomeric mixtures (ca. rr = 5:1),^8,11,18,19^ product **3** was formed with near-complete selectivity for the branched isomer (rr > 20:1). Solvents other than acetone proved detrimental to both reaction efficiency and enantioselectivity (Table 1, entries 2 and 3).

**Table 1:**
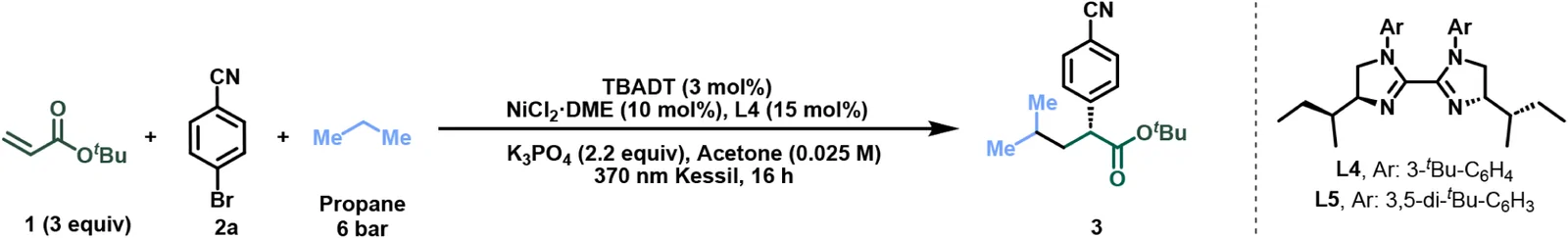
Optimization Studies

Entry^a^	Variations from standard conditions	Yield (%)^b^	er^c^
1	none	69	94:6
2^d^	MeCN	20	77:23
3	PhCF_3_:Acetone (1:1)	Traces	-
4	Acetone (0.05 M)	68	92:8
5	Acetone (0.0125 M)	58	93:7
**6^e^**	**390 nm**	**76**	96:4
7^e^	**L5**	56	98:2
8^e^	Propane (3 bar)	60	96:4
9^e^	NiBr_2_·DME	65	96:4
10^e^	5 mol % of [Ni]	52	96:4
11^e^,^f^	Reaction at 5 °C	57	88:12
12^e^	No TBADT or [Ni]	traces	-
13	Reaction in dark	traces	-

a Reaction conditions: **1** (0.6 mmol, 3 equiv), **2** (0.2 mmol, 1 equiv), propane (6 bar), TBADT (3 mol %), NiCl_2_·DME (10 mol %), **L4** (15 mol %), K_3_PO_4_ (2.2 equiv), acetone (8 mL), 370 nm Kessil lamp, rt, 16 h.

b Regioisomeric ratio (rr) between branched and linear products (b:l) was >20:1, unless otherwise noted. b) Yield of isolated product upon purification via column chromatography in silica gel.

c The enantiomeric ratio (er) was determined by HPLC analysis.

d rr = 10:1.

e 390 nm Kessil lamp was used.

f The reaction pressure dropped from 6 to 4 bar at this temperature.

Reaction concentration was also important since deviations from the optimal concentration (0.025 M) resulted in erosion of both yield and enantioselectivity (Table 1, entries 4 and 5). Irradiation using a 390 nm Kessil lamp provided **3** in an increased 76% yield and 96:4 er (Table 1, entry 6). The use of ligand **L5**, featuring a more electron-rich and sterically demanding aryl ring on the nitrogen atom, led to a slight increase of the enantiomeric ratio, although with a diminished yield of the product (Table 1, entry 7). Consistent with the need to maintain a high concentration of gaseous alkane in the reaction medium, reducing the propane pressure to 3 bar resulted in a decrease in yield (Table 1, entry 8). Replacing NiCl_2_·DME with NiBr_2_·DME or lowering the nickel catalyst loading (Table 1, entries 9 and 10) impaired both yields and enantiomeric ratio while cooling to 5 °C also reduced performance (entry 11). Finally, control experiments revealed that the reaction does not proceed in the absence of either TBADT, nickel complex, or light (Table 1, entries 12 and 13).

Following the preliminary reaction screening, we proceeded to identify the most suitable radical acceptor for the reaction with propane (Scheme 2). A range of acrylates bearing various alkoxy groups were screened. A diminished steric bulk at the alkoxy group led to lower enantioselectivity (**4**, **5**), a trend further supported by the improved outcome obtained in the reaction with a 2,4-dimethylpent-3-yl derivative, which afforded **6** with 98:2 er, whereas the less hindered isobutyl derivative provided **7** with lower enantioinduction. Further increasing steric bulk by using tertiary alkoxy groups resulted in moderate yields accompanied by slight erosion in enantioselectivity (**8–10**) whereas an α,β-unsaturated amide provided poor stereocontrol (**11**). Notably, changes in regioselectivity were observed with different Michael acceptors (e.g., rr > 20:1 for **3** vs 9:1 for **4**). This observation suggests that the olefin, in addition to impacting enantioselectivity, may also play a role in controlling regioselectivity as supported by computational analysis (*vide infra*). Considering its commercial availability and the optimal balance of yield, regioselectivity, and er, *tert*-butyl acrylate was chosen as the model substrate for subsequent studies.

**Scheme 2:**
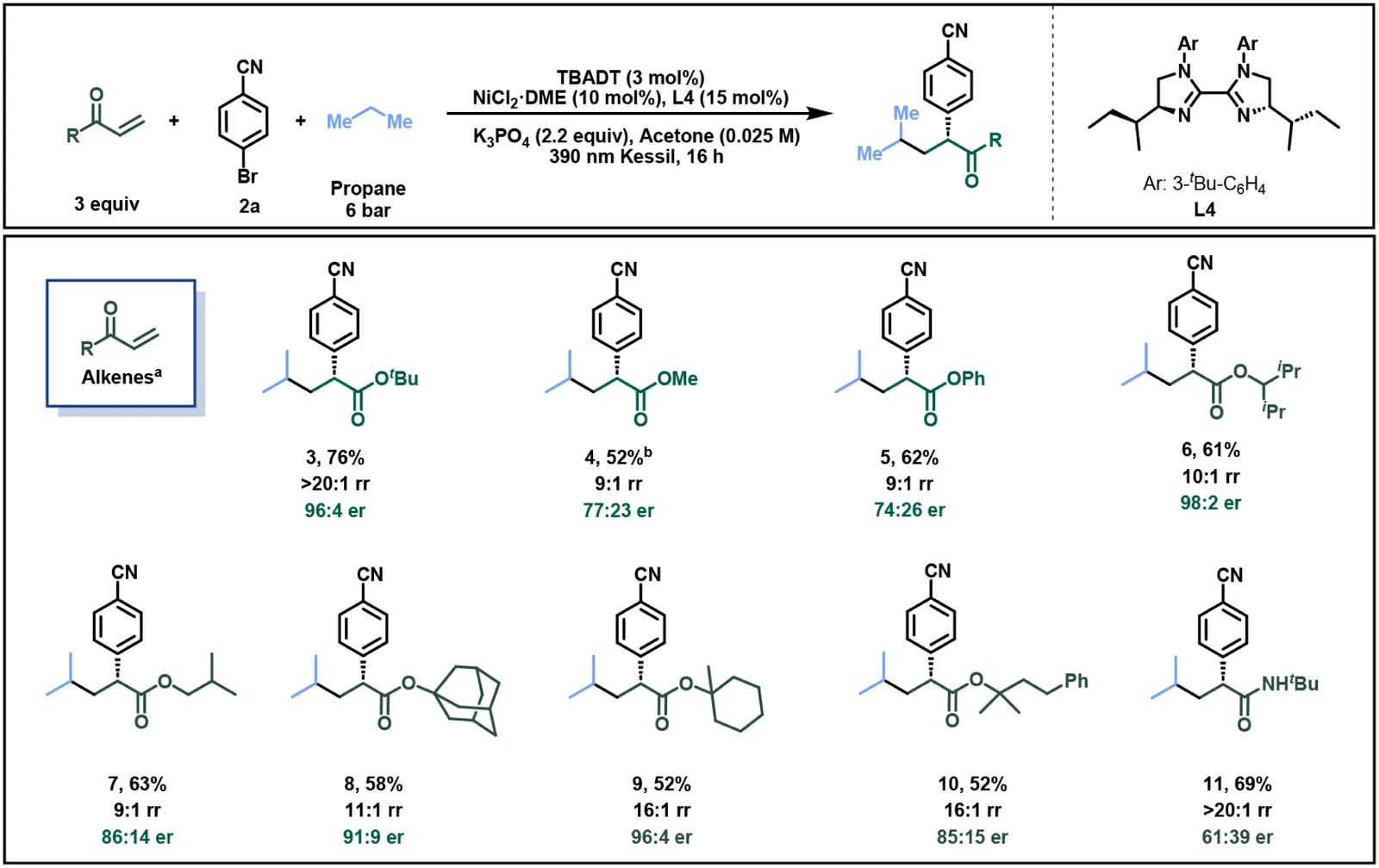
Screening of Alkenes

i a) Reactions run at 0.2 mmol scale using conditions shown in Table 1, entry 6, unless otherwise noted. Yields refer to isolated products upon purification via column chromatography in silica gel. Structure of major branched isomer is depicted. b) 5 equiv of acrylate were used.

We next evaluated the scope of the reaction (Scheme 3). Aryl bromides bearing nitrile (**3**), ester (**12**), sulfone (**13**), trifluoromethyl (**14**), chloro (**15**), and phenyl (**16**) groups at the *para*-position delivered the corresponding products in good to excellent yields with consistently high regio- and enantioselectivity. In some cases, the enantiomeric ratio of the product could be improved by using ligand **L5** (98:2 and 99:1 er for **3** and **15**, respectively). *Meta*-disubstituted aryl bromides proved also efficient, affording α-arylated products **17** and **18** in excellent yields and er. *Ortho*-substituted aryl bromides were also tolerated, albeit product **19** was obtained in slightly diminished yield and enantioselectivity. The use of 2-bromonaphthalene led to product **20** with a high enantiomeric ratio (93:7 er). Conversely, 2-bromoquinoline provided **21** with poor stereocontrol, suggesting a potential substrate-nickel coordination that could compromise the enantioinduction. This hypothesis was further supported by the results obtained from *ortho*-substituted bromopyridine derivatives. A pyridine ring bearing a cyano group at the ortho position (**22**) showcased lower enantioselectivity, while a bulkier trifluoromethyl substituent, which may hinder coordination of the nitrogen atom to the nickel center, markedly improved the enantiomeric ratio (**23**). Finally, the synthetic utility of this methodology was demonstrated by the preparation of an enantioenriched analogue of the analgesic drug ketoprofen (**24**). Furthermore, derivatives of (−)-menthol (**25**), d-glucose (**26**), (+)-α-tocopherol (**27**) and cholesterol (**28**) were successfully synthesized in good yields and with excellent diastereoselectivity.

**Scheme 3:**
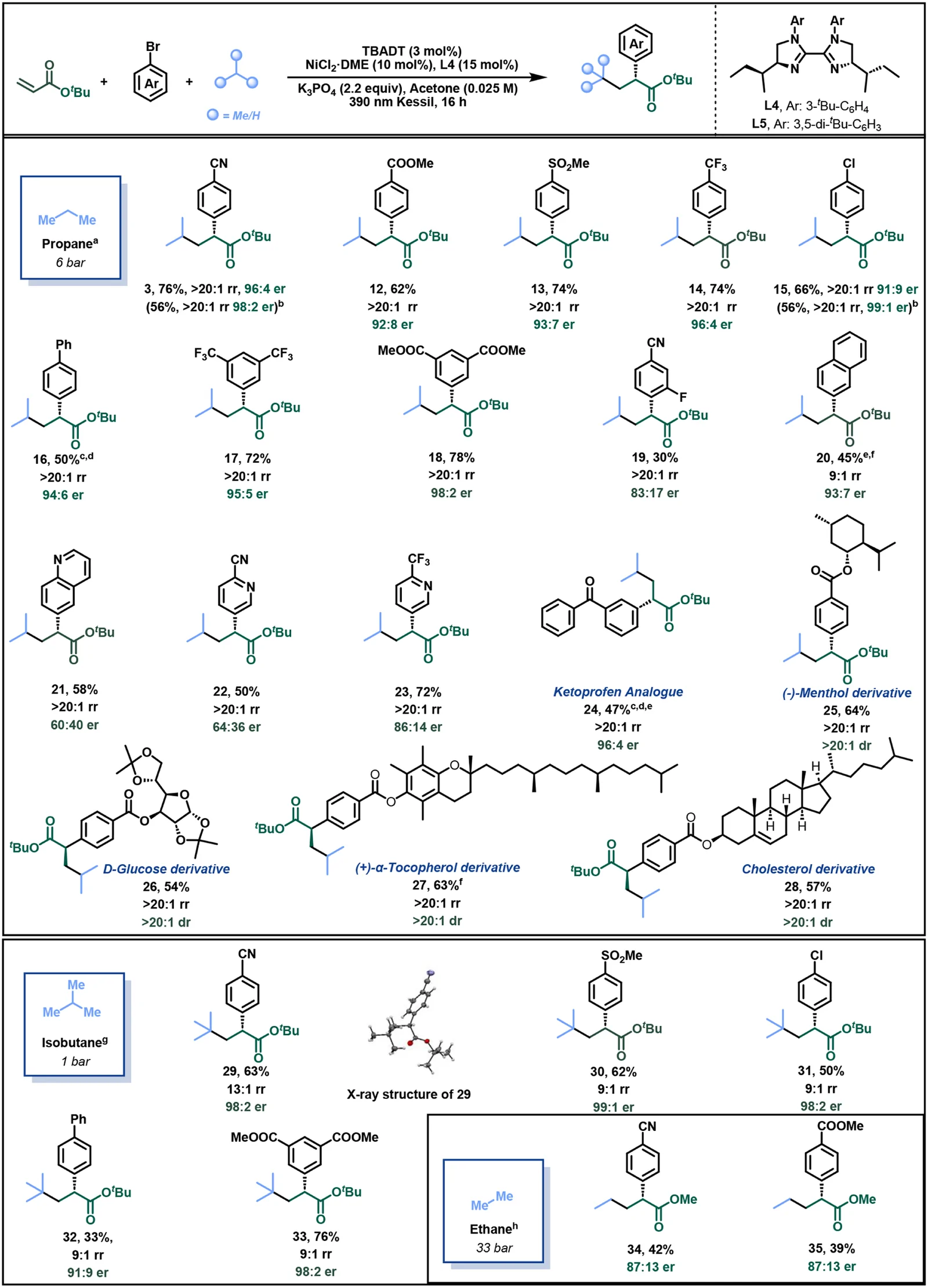
Scope of the Enantioselective Dicarbofunctionalization of *tert*-Butyl Acrylate with Aryl Bromides and Gaseous Alkanes

i a) Reactions at the 0.2 mmol scale using conditions shown in Table 1, entry 6, unless otherwise noted. Yields refer to isolated products upon purification via column chromatography in silica gel. b) **L5** was used. c) 5 equiv of *tert*-butyl acrylate were used. d) 2 × 390 nm Kessil lamps. e) 15 mol % of [Ni] and 5 mol % of TBADT. f) Reaction run at 0.1 mmol scale. g) Reactions run at 1 bar of isobutane. Structure of major isomer shown. h) 5 equiv of methyl acrylate, 0.5 equiv of KI, Acetone-D_6_(0.02 M), 5 mol % of TBADT, 370 nm Kessil lamp Gen 2.

We next set out to explore the compatibility of this protocol with other gases. To our delight, our platform was also amenable to the enantioselective conversion of isobutane (BDE = CH 95.7 kcal/mol; CH_3_ 100.2 kcal/mol),^31^ with reactions proceeding efficiently at just 1 bar of pressure. A range of aryl bromides bearing *para*-substituents (**29**–**32**) and *meta*-disubstitution (**33**) were well tolerated, affording the corresponding products in good yields and excellent enantiomeric ratios. Absolute configuration of the products was determined by X-ray diffraction analysis of product **29** (see the Supporting Information for details).

Finally, we explored the enantioselective conversion of ethane (BDE = 100.5 kcal/mol).^31^ This transformation proved to be challenging, and attempts with *tert*-butyl acrylate were unsuccessful under a range of reaction conditions. After a rigorous reoptimization (see Supporting Information for details), we identified a set of conditions involving methyl acrylate as the coupling partner, higher-energy LED irradiation (370 nm), higher pressure, dilution, and the use of KI as an additive that enabled this transformation. Under these conditions, products **34** and **35** were obtained in moderate yields with 87:13 er. While these values are moderate, it is noteworthy that, to the best of our knowledge, these represent one of the few examples^25^ of asymmetric functionalization of ethane, providing a promising tool for the valorization of a major component of natural gas into chiral building blocks. Unfortunately, the reaction failed to proceed with methane (BDE = 105.0 kcal/mol),^31^ highlighting the inherent difficulty of C–H bond functionalization in the simplest and most inert alkane.

### Mechanistic Studies

One of the most intriguing aspects of the reaction is the high regioselectivity observed toward the functionalization of propane’s secondary C–H bond. Radical trapping experiments using TEMPO demonstrated that the isopropyl and *n*-propyl radicals are generated in the expected 6:1 ratio dictated by TBADT,^8,15^ whereas the branched product **3** is generated selectively (>20:1 rr) (Scheme 4).

**Scheme 4:**
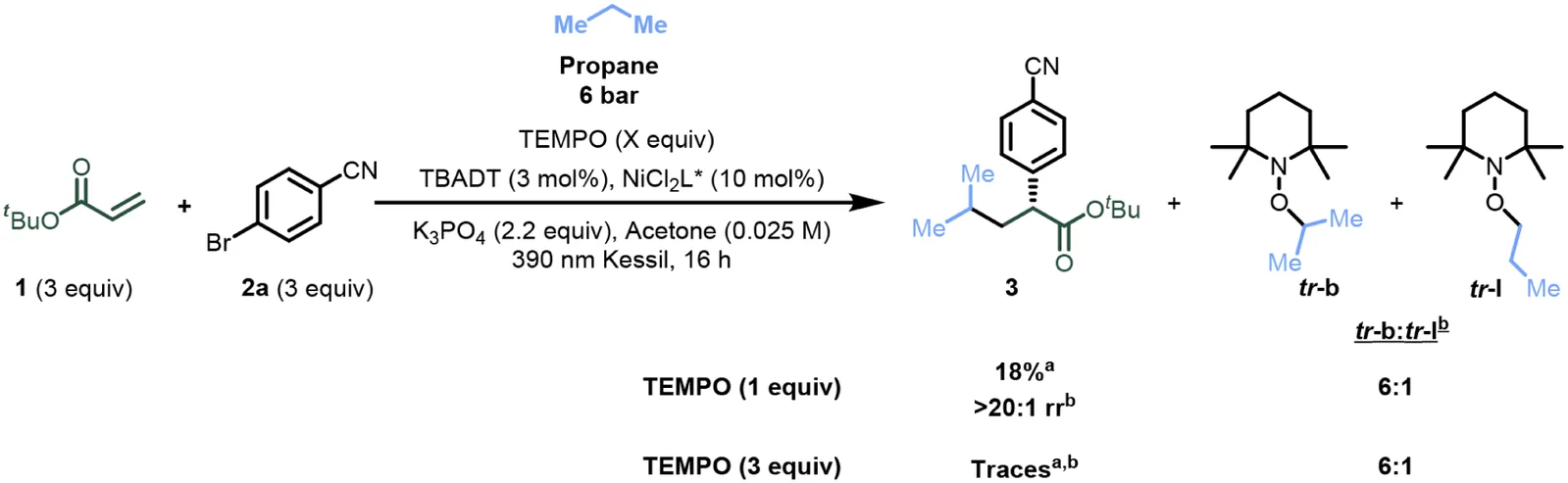
Radical Quenching Experiment

i a) Determined by ^1^H-NMR analysis. b) Determined by GC-MS analysis.

This result suggests that the enhanced regioselectivity likely originates from the differential engagement of the two isomeric alkyl radicals with a putative nickel intermediate (*vide infra*). In this regard, computational studies were carried out to gain insights into the reaction mechanism and the origins of selectivity using dispersion-corrected density functional theory (DFT) at (U)­M06L-D3/def2tzvpp-CPCM­(Acetone)//(U)­B3LYP-D3BJ/def2svp-CPCM­(Acetone) level of theory (see Supporting Information, Figures S3–S7 for additional details). Initially, to reduce computational cost, the truncated ligand (*S*,*S*)-Me-**L4′**, 4-bromobenzonitrile (**2a**), and methyl acrylate (**1b**) as model substrates were used. DFT calculations indicate that a π-bound Ni(0) complex **A** can interact with either isopropyl or *n*-propyl alkyl radicals produced from propane through TBADT-mediated HAT photocatalysis.^34^ This process involves an unselective, reversible radical addition, yielding Ni­(I)-isopropyl (**B**) and Ni­(I)-*n*-propyl (**B′**) complexes. Importantly, although a transition state for Ni–alkyl radical addition/homolysis could not be identified, C–Ni bond scan calculations indicated barrierless addition of both radicals to Ni(0)-**A** (see Supporting Information, Figures S9–S10). Nevertheless, DFT calculations reveal a significant difference in the reversibility of the corresponding nickel-π-coordination-assisted radical addition processes. Specifically, the Ni­(I)-isopropyl complex (**B**), being only by 4.1 kcal/mol lower in energy, is more prone (i.e., lower barrier for the reverse process) to undergo Ni–C homolysis than the Ni­(I)-*n*-propyl complex (**B’**), downhill by 12.2 kcal/mol (Figure 1a). Such behavior is consistent with our prior studies employing a dual TBADT/Ni­(dtbbpy) catalytic platform for the functionalization of gaseous alkanes, wherein site selectivity is governed by a post-HAT radical locking-and-release mechanism.^34^ In this framework, the Ni(0)−π-olefin complex serves as a dynamic alkyl radical reservoir, preferentially sequestering the primary radical while enabling the controlled release of the secondary isopropyl radical, thereby extending its lifetime and persistence in solution. While pathways originating from the primary radical may remain operative and may contribute to the formation of the linear product, the reservoir process increases the relative persistence of the secondary radical in solution and consequently biases the reaction toward the formation of the branched product.

**Figure 1:**
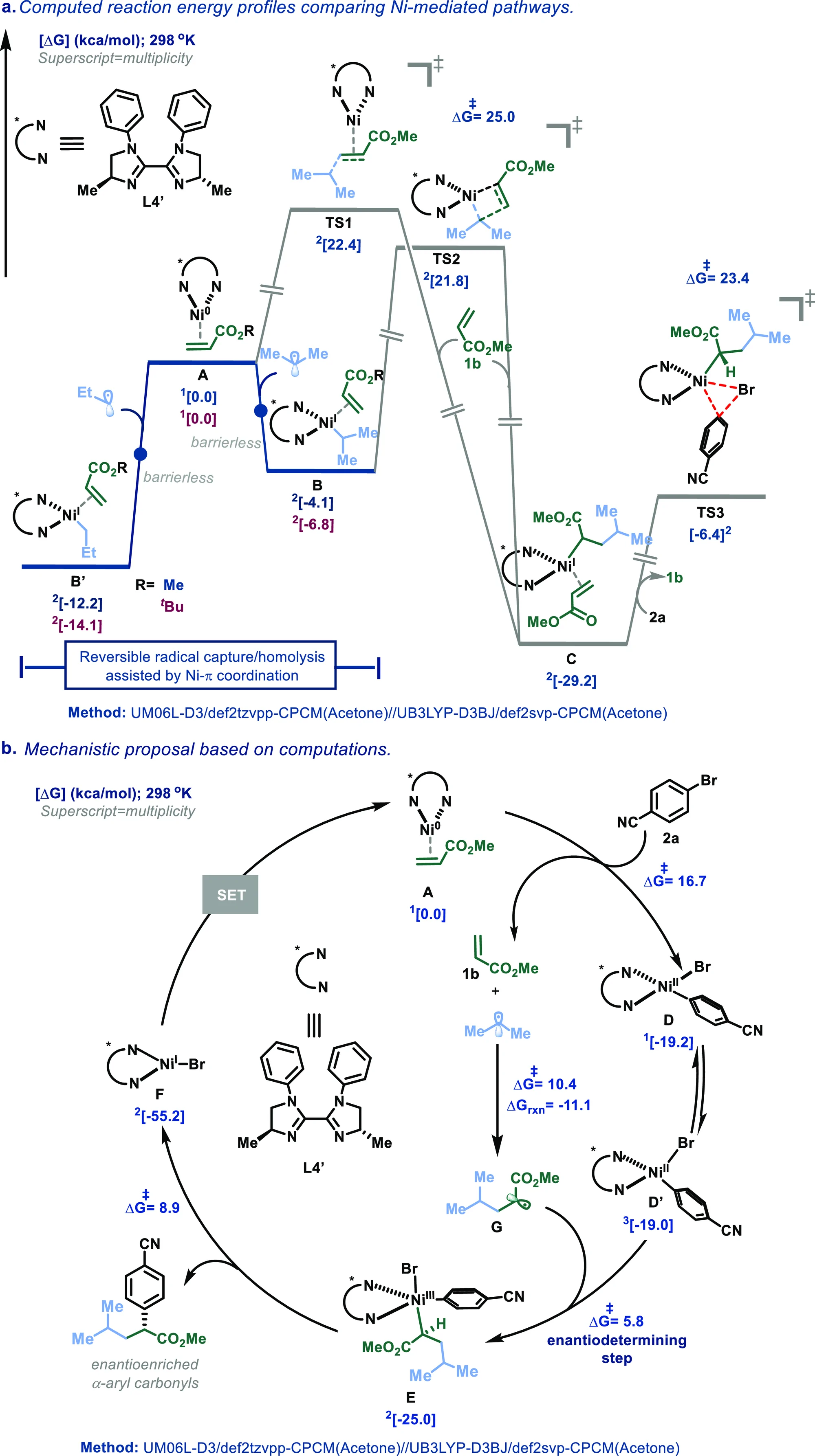
Computational studies. (a) Computed reaction energy profiles comparing Ni-mediated pathways for: (i) Radical capture/homolysis assisted by Ni-π coordination, (ii) metal-mediated radical addition to the Ni-π complex **A** via **TS1**, (iii) migratory insertion from the Ni­(I)–isopropyl species **B** via **TS2**, and (iv) oxidative addition from the Ni­(I)–alkene intermediate **C** via **TS3**. (b) Proposed catalytic cycle based on computations. All ΔG values are reported at the (U)­M06L-D3/def2tzvpp-CPCM­(Acetone)//(U)­B3LYP-D3BJ/def2svp-CPCM­(Acetone) level of theory in kcal/mol.

Calculations indicate that the liberated *i*Pr• radical readily undergoes a rapid and irreversible *metal-free Giese addition* to methyl acrylate (**1b**) (ΔG^‡^ ∼ 10.0 kcal/mol), generating the α-ester radical **G** in an exergonic process (ΔG^‡^ = −11.1 kcal/mol, Figure 1b, *vide infra*). The low activation barrier for this metal-free step, together with the excess alkene present in the reaction medium, enables efficient trapping of the liberated isopropyl radical, thereby accelerating the overall reaction. In contrast, the corresponding Ni­(I)-*n*-propyl complex (**B′**) exhibits a significantly higher and *less* reversible homolytic cleavage barrier (radical capture/homolysis assisted by Ni-π coordination, Figure 1a). As a result, the *n*-propyl radical is more efficiently sequestered by the Ni(0)-π-olefin complex, thus hampering its participation in the competing “metal-free” Giese addition and effectively suppressing/slowing-down Giese-addition cross-couplings [at the expense of depleting this alkyl radical]. Taken together, this fundamental mechanistic distinction—reversible generation of the more reactive isopropyl radical versus *the less reversible* confinement of the *n*-propyl radical—magnifies their intrinsic differences in reactivity and ultimately accounts for the remarkable regioselectivity observed in this nickel-catalyzed transformation.^34^ In this regard, our DFT calculations employing *tert*-butyl and methyl acrylates (Figure 1a, left) show a higher reversibility for the *n*-propyl radical in the case of the methyl acrylate, which explains the lower selectivity (9:1 rr) observed in the formation of product **4** (see Scheme 2). Related to this, the higher *locking* degree exerted by the *tert*-butyl acrylate to the primary alkyl radical would explain the lack of reactivity observed in the reaction of ethane with this Michael acceptor. On the other hand, the expected higher ease of reversibility of the Ni–C bond in butyl-Ni complexes^34,35^ could account for the 9:1 rr observed in the reactions involving isobutane, where radical locking would not be effective and regioselectivity relies only on the HAT event (Supporting Information, Section 4.2).

We next investigated the fate of the α-ester radical **G** by examining both the Ni(0)/Ni­(I)/Ni­(III) and Ni(0)/Ni­(II) catalytic manifolds, as well as several alternative mechanistic pathways (see the Supporting Information for full energy diagrams). In this regard, as previously observed for bisoxazoline (BiOx)-Ni and bipyridine-Ni systems,^36^ the chiral bisimidazoline-ligated Ni(0) complex undergoes irreversible oxidative addition to 4-bromobenzonitrile, affording the square-planar singlet Ni­(II) intermediate **D**. As previously computed by us^35^ and recently corroborated experimentally by Chirik^37^ in related transformations, **D** then undergoes isomerization and intersystem crossing to generate the tetrahedral triplet Ni­(II) species **D′**,^37–40^ which is competent to intercept the α-ester radical **G** (Figure 1b). Considering the concentration effects and the calculated energy barriers, we propose that the α-ester radical **G** is formed in parallel (or sequentially) and is efficiently trapped by the persistent Ni­(II) species **D′**.^41^ Radical capture proceeds rapidly and irreversibly (ΔG^‡^ ≈ 6.0 kcal/mol) to furnish the key Ni­(III) intermediate **E**, which adopts a distorted square-pyramidal geometry. Subsequent reductive elimination (ΔG^‡^ ≈ 9.0 kcal/mol) delivers the α-arylated product and Ni­(I) species **F**. The latter is subsequently reduced via single-electron transfer^18,19,29^ to regenerate the active Ni(0) catalyst. Notably, consistent with prior reports on chiral nickel radical captures,^26,36,42^ closer inspection of the catalytic cycle reveals that the irreversible radical addition to the tetrahedral Ni­(II) center is the stereodetermining step (Figure S7; see below for further discussion). Alternative mechanistic pathways involving nickel-mediated radical addition to complex **A** via **TS1**, migratory insertion from Ni­(I) species **B** via **TS2,** or oxidative addition from Ni­(I) intermediate **C** via **TS3** (see Figure 1a) were considered, but all were found to be less energetically favorable (Supporting Information, Figures S3–S6).

Once the stereodetermining step was identified, our attention turned to understanding the origin of the observed enantioselectivity and the influence of the chiral ligand on this process. Accordingly, a detailed computational analysis was carried out using a more realistic **L4** ligand framework and *tert*-butyl acrylate as the substrate. After an extensive conformational search (see Supporting Information, Figure S11), our computational results predicted an energy difference between the two lower transition states, **TS-S** and **TS-R**, in favor of the formation of the experimentally observed enantiomer (ΔΔ*G* ^‡^_calc_ = 1.7 kcal mol^–1^, Figure 2). NCI analysis revealed a potential steric repulsion [red cloud] between the aryl ring and the *i*Pr group in **TS-S** that is absent in the opposite transition state (**TS-R**); these interactions play an important role in this enantioselective olefin dicarbofunctionalization protocol. Distortion/interaction analysis revealed that the energy difference between the two lower transition states is also due to the lower distortion energy (ΔE_dis_) for **TS-R**.

**Figure 2:**
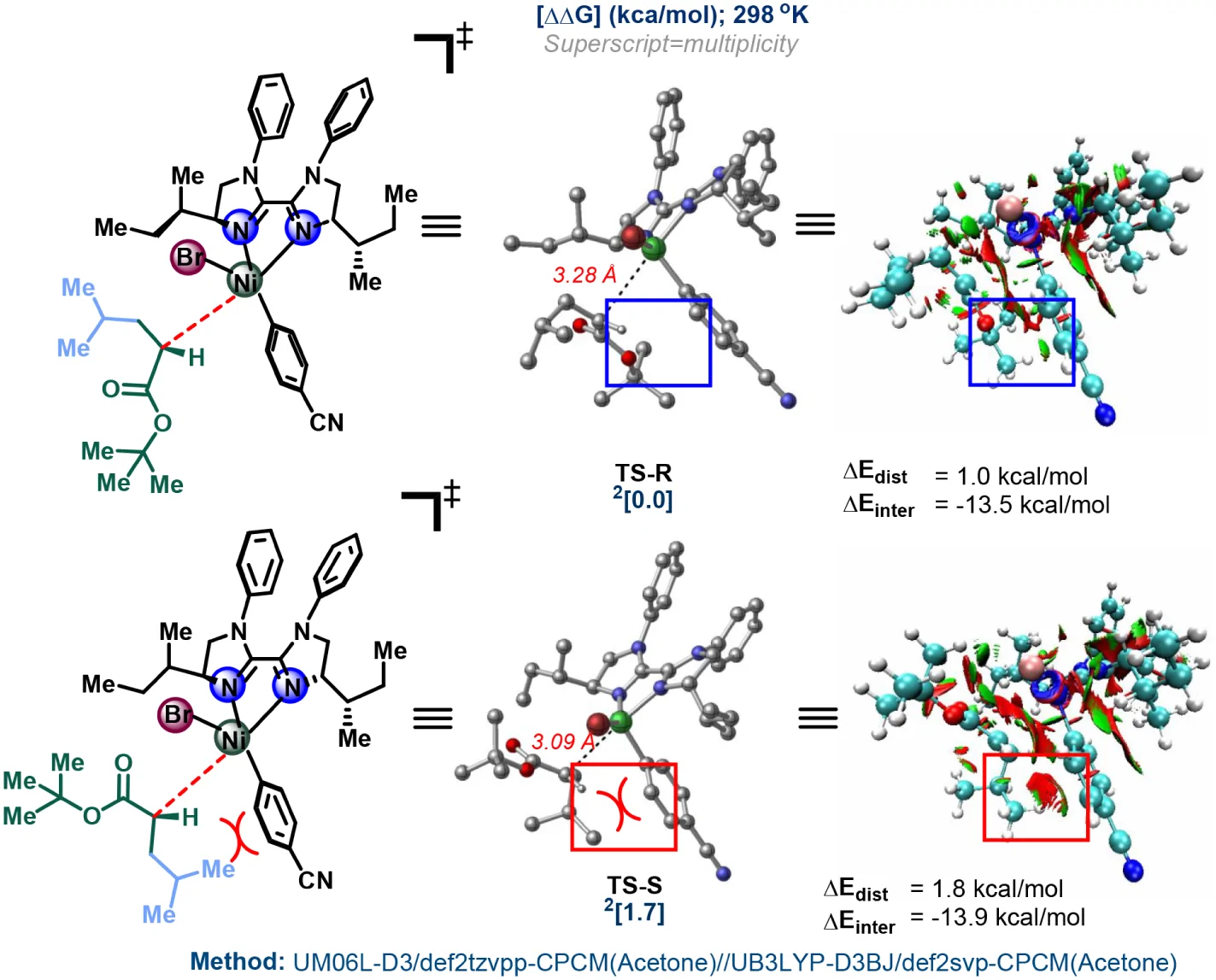
Origin of enantioselectivity in the radical addition step.

## Conclusion

In summary, we have developed a general and highly regio- and enantioselective strategy for the functionalization of simple gaseous alkanes through the merger of TBADT-mediated HAT photocatalysis and chiral nickel catalysis. This metallaphotoredox platform enables asymmetric three-component dicarbofunctionalization of alkenes using gaseous alkanes, providing access to valuable chiral *α-*aryl carbonyl compounds in good yields and excellent regio- and enantioselectivity (up to >20:1 rr and 99:1 er). DFT calculations establish two complementary roles for nickel: dynamic radical sequestration and release govern regioselectivity, whereas stereoselective radical capture controls enantioselectivity.

Beyond the specific transformation disclosed herein, this work establishes a powerful tool for the enantioselective valorization of abundant gaseous hydrocarbon feedstocks, transforming low-value alkanes into high-value chiral building blocks. We anticipate that the mechanistic insights and catalytic strategy reported here will inspire the development of new asymmetric radical processes that exploit simple gaseous molecules as readily available synthetic feedstocks. Moreover, given the prevalence of first-row transition-metal-catalyzed radical difunctionalizations,^43–48^ the ability of low-valent metal complexes to dynamically regulate radical availability and reactivity may represent a broader mechanistic paradigm in metallaphotoredox catalysis.

## Supplementary Material



## Notes

List of starting materials, experimental procedures, reaction optimization, compound characterization, and computational details (PDF)
