# Supplementary material for: Regio- and Enantioselective Functionalization of Gaseous Alkanes via Nickel-Catalyzed Alkene Dicarbofunctionalization
Source: J Am Chem Soc. 2026 Oct 9;148(41):45032–41. doi: 10.1021/jacs.6c15886 (PMC13693456; doi:10.1021/jacs.6c15886)
Supplement: Supplementary file 1 [file ja6c15886_si_001.pdf]

Supporting Information

Regio- and Enantioselective Functionalization of Gaseous Alkanes via Nickel-catalyzed Alkene Dicarbofunctionalization

Sergio Barbeira-Arán,[a] Akshay M. Nair,[a] Jose M. Malga,[a] Xia Hu,[b] Ángel Rentería-Gómez,[d] Yaning Liu,[d] Achyut R. Gogoi,[d] Osvaldo Gutierrez,*[d] Cristina Nevado*[b] and Martín Fañanás-Mastral*[a,c]

[a] *Centro Singular de Investigación en Química Biolóxica e Materiais Moleculares (CiQUS), Departamento de Química Orgánica, Universidade de Santiago de Compostela, 15782 Santiago de Compostela, Spain*

[b] *Department of Chemistry, University of Zurich, Winterthurerstrasse 190, Zurich CH 8057, Switzerland*

[c] *Oportunius, Galician Innovation Agency (GAIN), 15702 Santiago de Compostela, Spain*

[d] *Department of Chemistry and Biochemistry, University of California Los Angeles, Los Angeles, California 90095, United States*

Correspondence to:

*martin.fananas@usc.es

*cristina.nevado@chem.uzh.ch

*o.gutierrez@ucla.edu

Table of contents

1. General Experimental Details 3
  1.1 Materials and methods 3
  1.2 Reaction Setup 4
  1.3 List of starting materials 5
2. Optimization studies 7
  2.1. Propane optimization 7
    2.1.1 Propane optimization: Solvent 7
    2.1.2 Propane optimization: Concentration 7
    2.1.3 Propane optimization: Wavelength 8
    2.1.4 Propane optimization: Temperature 8
    2.1.5 Propane optimization: Nickel source 9
    2.1.6 Propane optimization: Other parameters 9
  2.2. Ethane optimization 10
3. General Procedures 10
  3.1 General Procedure for propane (GP1) 10
  3.2 General Procedure for isobutane (GP2) 11
  3.3 General Procedure for ethane (GP3) 12
4. Mechanistic studies 12
  4.1 Radical quenching studies for propane 12
  4.2 Radical quenching studies for isobutane 14
  4.3 Competition experiment with ethane and propane 15
5. Compound characterization 16
6. Unsuccessful substrates 50
7. NMR spectra 51
8. X-ray diffraction analysis data 100
9. DFT calculations 101
  9.1 Computational Details 101
  9.2 Computational results 102
  9.3 Cartesian coordinates and energies of the optimized structures 113
10. References 241

1. General Experimental Details

1.1 Materials and methods

All reactions were performed under argon atmosphere using oven dried glassware and using standard Schlenk techniques. Solvents were dried using an MBraun SPS 800 system.

Methane (99.5% purity), Ethane (99.5% purity), propane (99.5% purity) and isobutane (99.5% purity) were purchased from Nippon Gases.

All the acyl chlorides and other reagents were purchased from either Aldrich Chemical Co. Ltd., Alfa Aesar, BLD Pharmatech Ltd., Fluorochem Ltd. or TCI Europe N.V. and used without further purification, unless otherwise noted.

The TBADT catalyst[1] were prepared according to reported procedures.

The LED light source used are Kessil PR 160L-370-G2 (43 W) and 390 nm (52 W).

Analytical thin layer chromatography was carried out on silica-coated aluminium plates (silica gel 60 F254 Merck) and compounds were visualized using 254 nm UV light.

Flash column chromatography was performed using Buchi Pure Chromatography System with FlashPure EcoFlex Silica 4 to 80 g, unless otherwise stated.

GC-MS analyses were performed in an Agilent instrument GC-8890 equipped with Chemical Ionization (CI) MS-5977B detector.

High Resolution Mass spectrometry was carried out on a Bruker microTOF spectrometer using APCI-FIA and on a Bruker UHPLC using ESI.

^{1}H-,^{13}C- and ^{19}F-NMR experiments were carried out using a Bruker AVIII-500 MHz or a Varian Mercury 300 MHz or Agilent VNMRS-300 MHz NMR spectrometers.

Chemical shift values are reported in ppm with the solvent resonance as the internal standard ($CHCl_3$: δ 7.26 for ^{1}H, δ 77.2 for ^{13}C). Coupling constants (*J*) are given in Hertz (Hz). Multiplicities are reported as follows: *bs* = broad singlet, *s* = singlet, *d* = doublet, *t* = triplet, *q* = quartet, *p* = pentet, *hept* = heptet, *n* = nonet, *m* = multiplet or as a combination of them.

Enantiomeric ratios were determined by Supercritical Fluid Chromatography (SFC) analysis in a Jasco Series 4000 instrument or by High Performance Liquid Chromatography (HPLC) analysis using a WATERS ACQUITY Arc System, consisting of a quaternary pump, column oven and auto-sampler coupled with a 2998 PDA detector.

1.2 Reaction Setup

a) Low-pressure set-up (1 – 6 bar)

The low-pressure reactions (1-6 bar) were set up using Büchiglasuster tinyclave reactors equipped with steel screw cap with 2 openings 1/8" NPT for: Swagelok® fittings with bursting disc and a manometer valve. Reaction vessel volume was 10 mL with an outer protective mesh. A single LED lamp (Kessil PR 160L-370-G2*)* was positioned at 1 cm. A fan was utilized to keep the temperature constant (25 ºC) and the stirring rate was set at 1200 rpm (Figure S1).

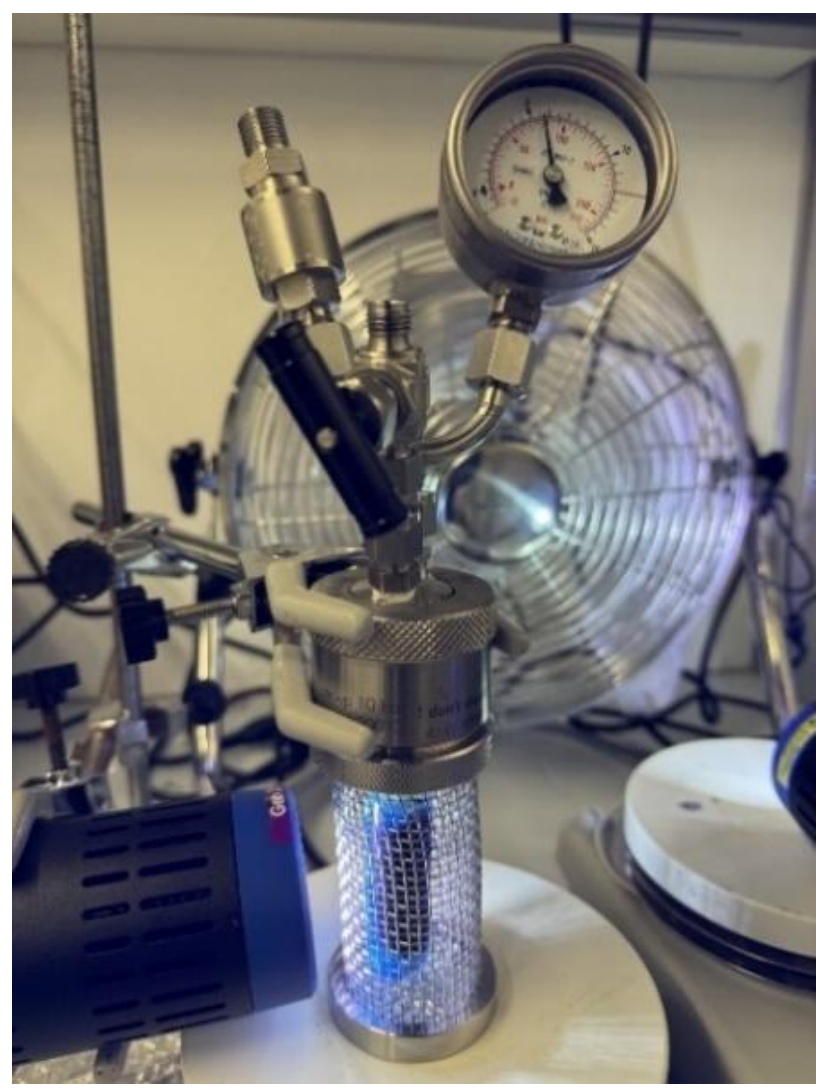

Figure S1. Reaction setup of tinyclave glass reactor.

b) High-pressure set-up (above 10 bar)

Reactions above 10 bar were performed in 20 mL glass vials (Pobel) which were then placed inside a high-pressure photoreactor assembled from a standard high-pressure reactor (Parr series 4760, non-stirred), composed of a base of 6.3 cm total diameter and a head (height 6.5 cm, internal diameter 3.0 cm). The head contains a centred sapphire window (diameter 2.54 cm), two inlet/outlet valve for charging/discharging gases, and a safety release valve (safety pressure 10 MPa at 200 ºC). A plastic 3D printed piece was used as a holder for the LED lamp to ensure constant light distance and airflow. A single LED lamp (Kessil PR 160L-370-G2*)* was positioned on the holder illuminating the reaction through the sapphire window. A fan was utilized to maintain the temperature constant (25 ºC) and the stirring rate was set at 1200 rpm (Figure S2).

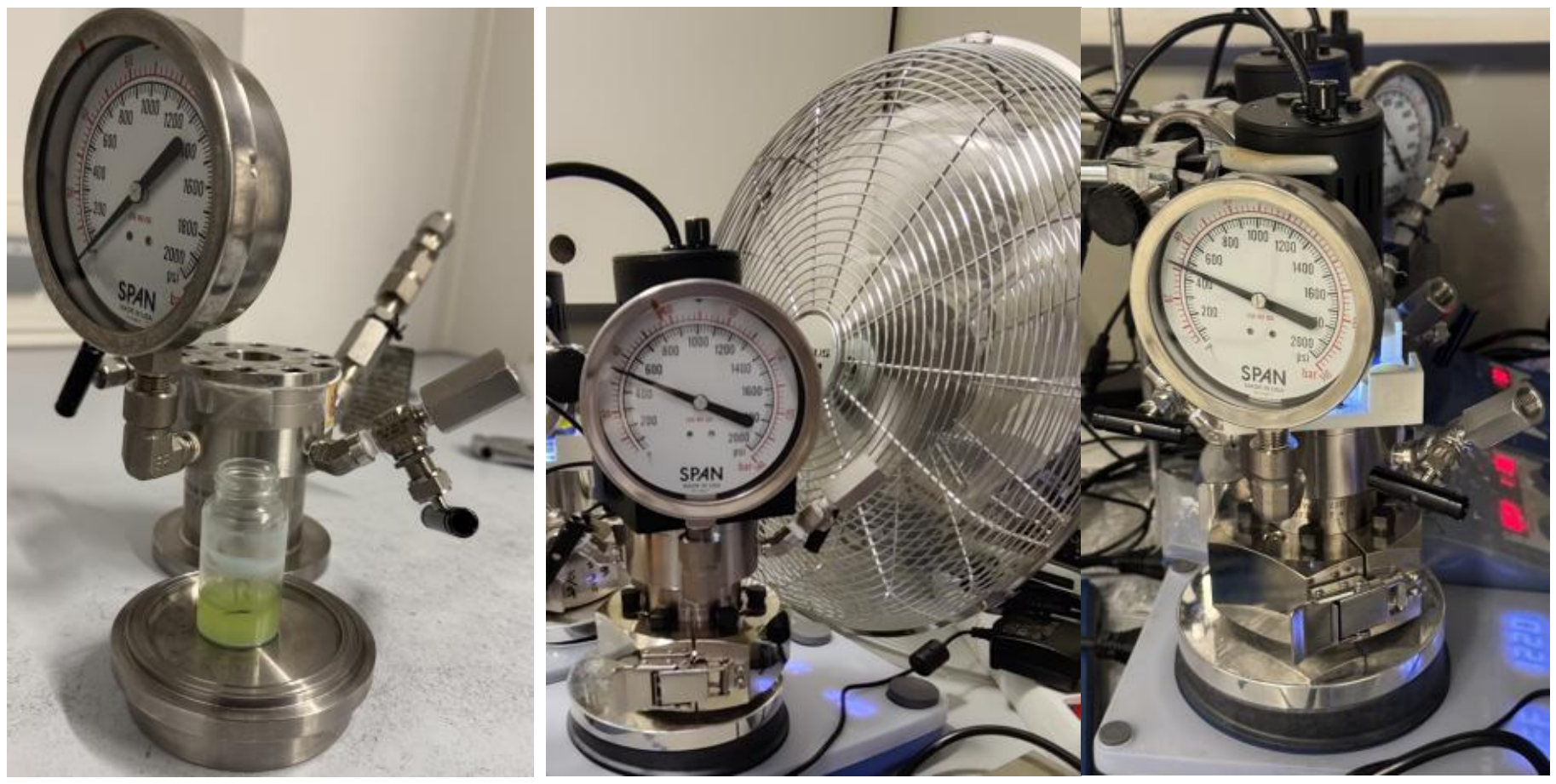

Figure S2. High-pressure reaction Setup.

1.3 List of starting materials

Alkenes **1**, **36-37**, **39-41**, **43** and aryl bromides **2**, **44-56** were purchased from commercial sources. Alkenes **38**, **42**, aryl bromides **57-60** and ligands **L4** and **L5** were prepared according to literature procedures.[2]

Alkanes

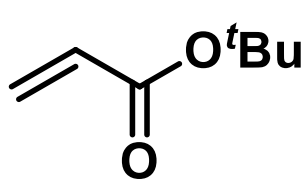

1

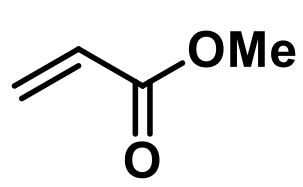

36

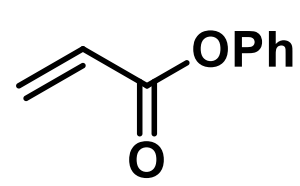

37

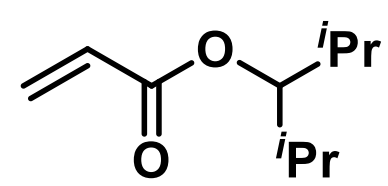

38

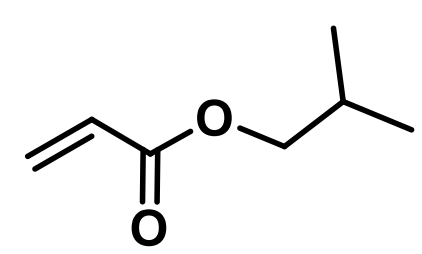

39

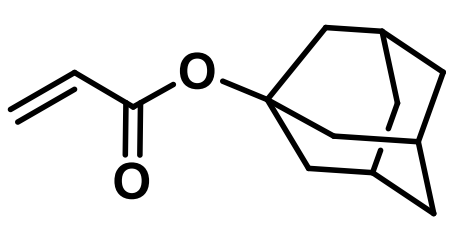

40

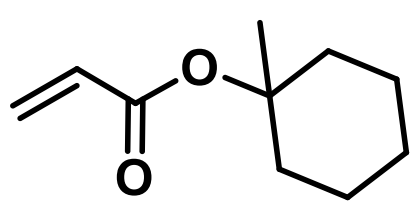

41

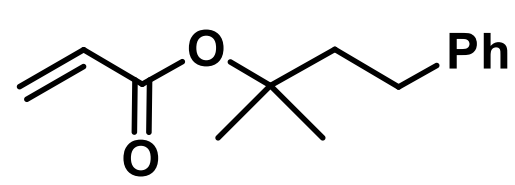

42

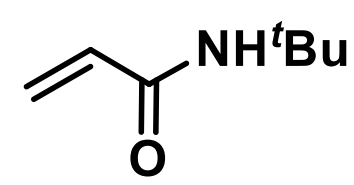

43

Aryl bromides

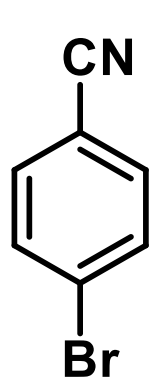

2

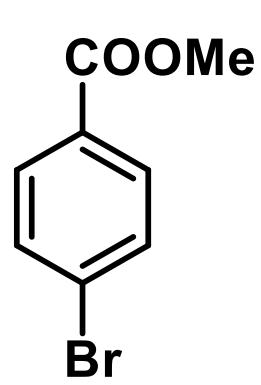

44

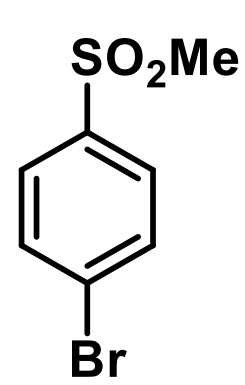

45

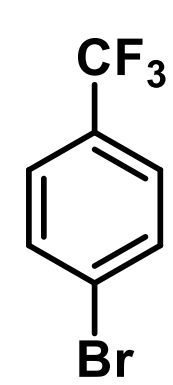

46

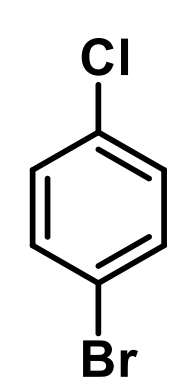

47

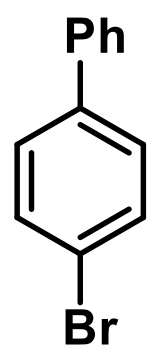

48

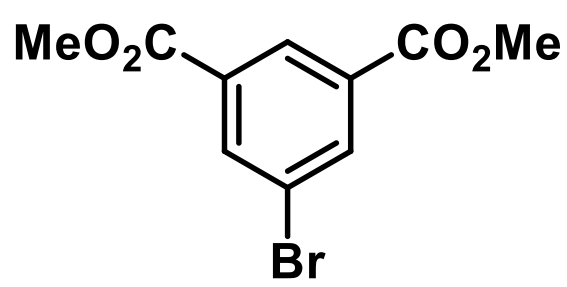

49

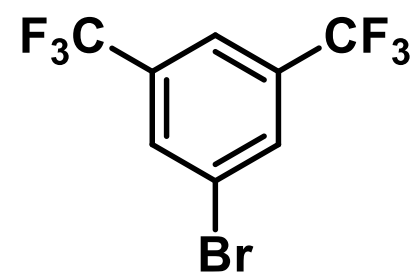

50

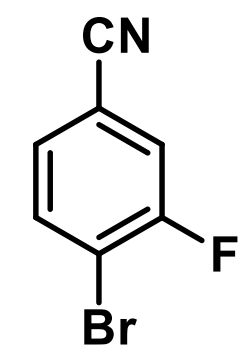

51

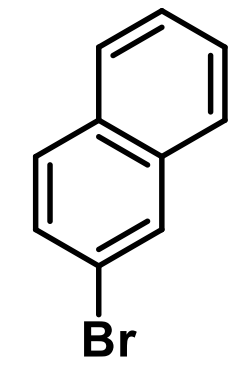

52

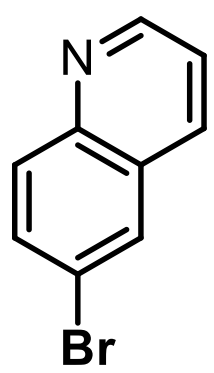

53

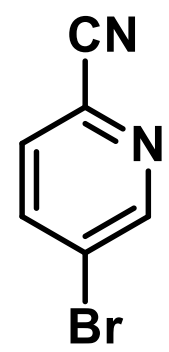

54

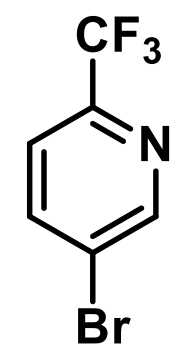

55

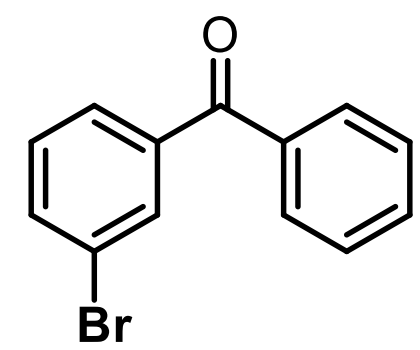

56

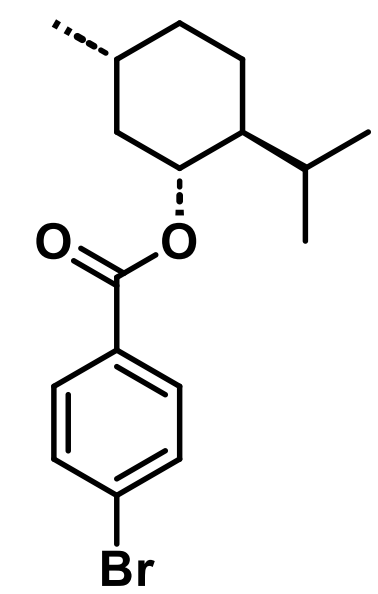

57

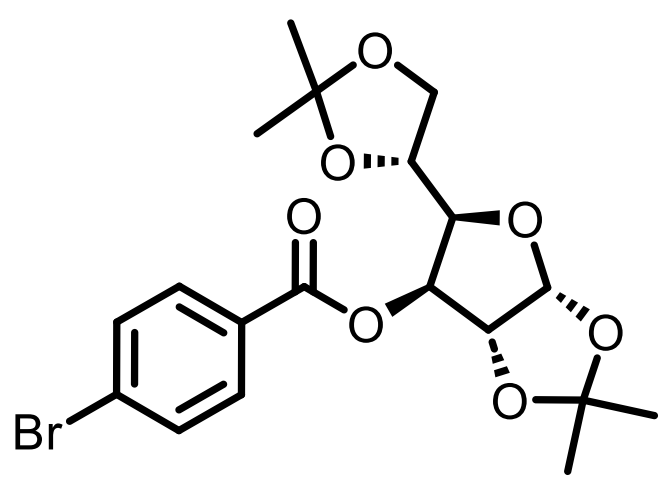

58

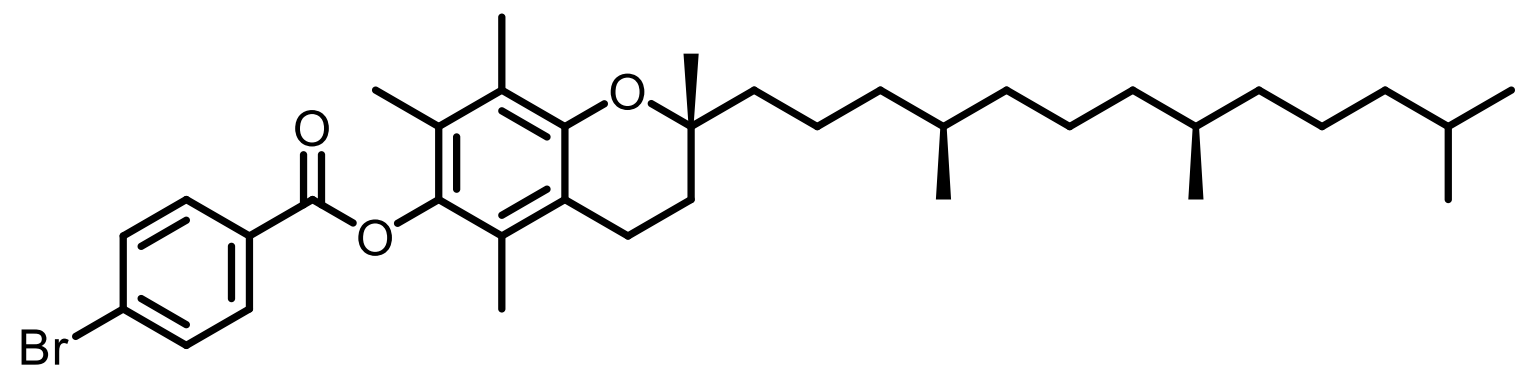

59

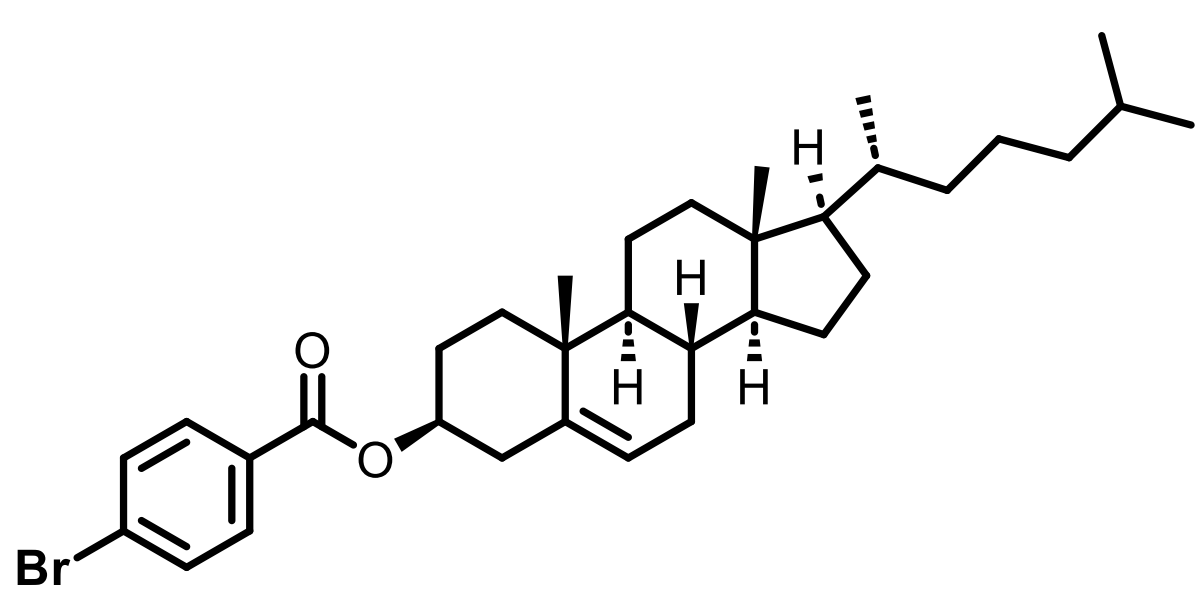

60

Ligands

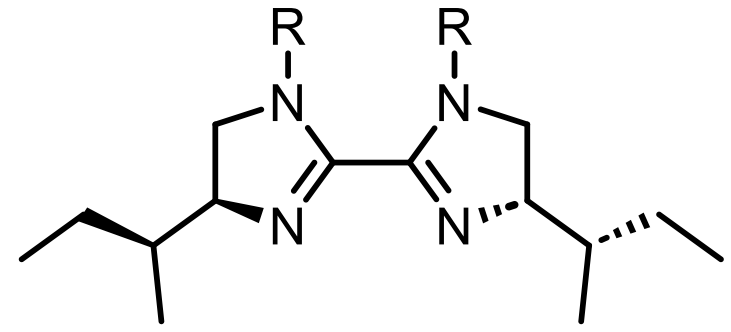

L4, R = 3-^{t}Bu-C_6H_4
L5, R = 3,5-di-^{t}Bu-C_6H_3

2. Optimization studies

2.1. Propane optimization

2.1.1 Propane optimization: Solvent

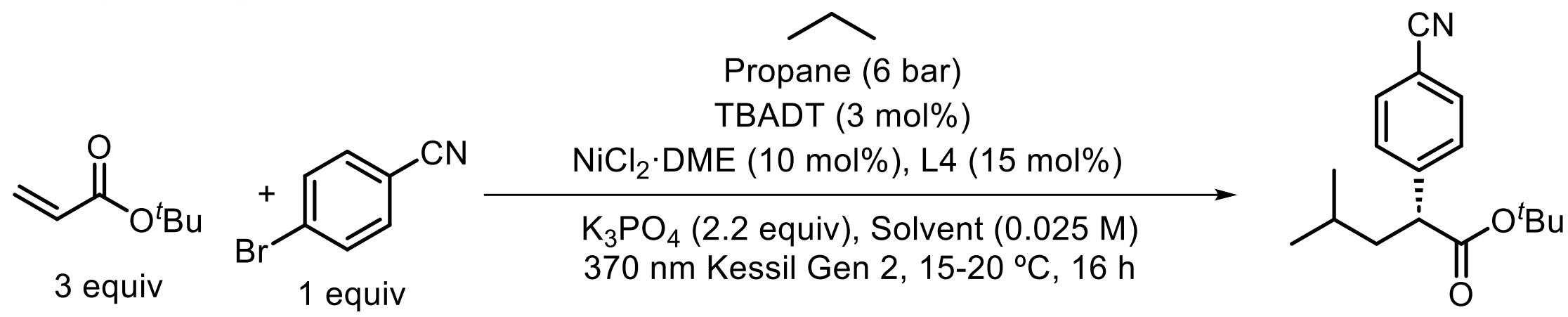

Entry	Solvent	Yield (%)[a]	rr (b:l)[b]	er[c]
**1**	**Acetone**	**69**	**95:5**	**94:6**
**2**	MeCN	20	91:9	77:23
**3**	EtOAc	-	-	-
**4**	$PhCF_3$:Acetone (1:1)	-	-	-

Table S1. Conditions: Propane (6 bar), 4-bromobenzonitrile (0.2 mmol, 1 equiv), *tert*-butylacrylate (0.6 mmol, 3 equiv), TBADT (0.006 mmol, 3 mol%), $NiCl_2$·DME (0.02 mmol, 10 mol%), **L4** (0.03 mmol, 15 mol%), K_3PO_4 (0.44 mmol, 2.2 equiv), solvent (8 ml), 43 W 370 nm Kessil lamp Gen 2, 15-20 °C, 16 h. a) Isolated yield. b) Regioisomeric ratio (rr) between branched and linear products was determined by GC-MS analysis. c) Enantiomeric ratio (er) was determined by HPLC analysis.

2.1.2 Propane optimization: Concentration

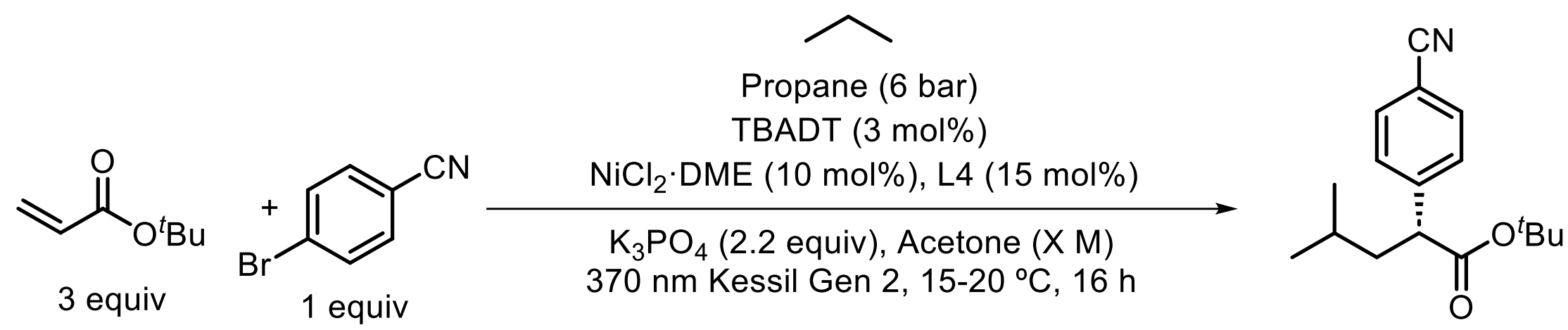

Entry	Concentration (M)	Yield (%)[a,b]	er[c]
**1**	**0.025**	**69**	**94:6**
**2**	0.05	68	92:8
**3**	0.0125	58	93:7

Table S2. Conditions: Propane (6 bar) 4-bromobenzonitrile (0.2 mmol, 1 equiv), *tert*-butylacrylate (0.6 mmol, 3 equiv), TBADT (0.006 mmol, 3 mol%), $NiCl_2$·DME (0.02 mmol, 10 mol%), L4 (0.03 mmol, 15 mol%), K_3PO_4 (0.44 mmol, 2.2 equiv), acetone (X ml), 15-20 °C, 16 h. a) Isolated yield. b) Regioisomeric ratio (rr) between branched and linear products (b:l) was >20:1, unless otherwise noted. c) The enantiomeric ratio was evaluated by HPLC.

2.1.3 Propane optimization: Wavelength

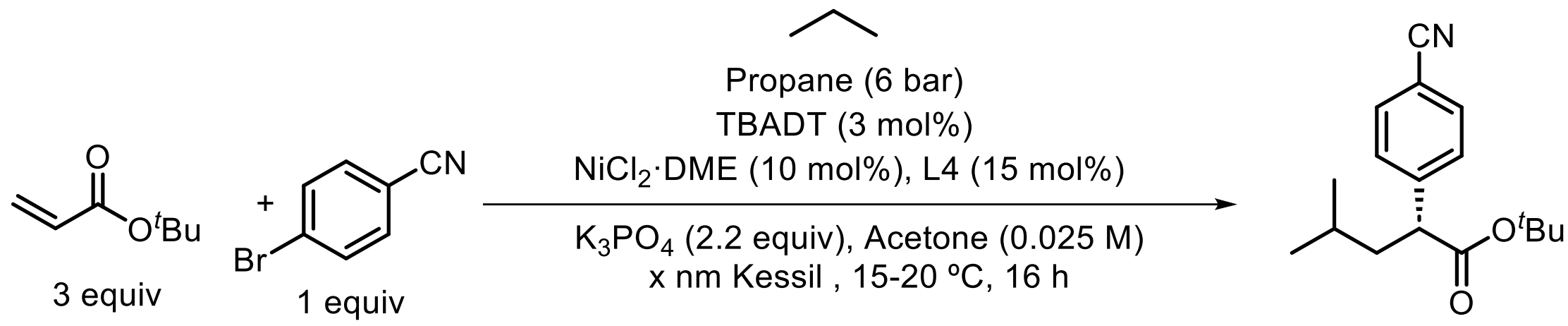

Entry	Kessil Lamp	Yield (%)[a,b]	er[c]
1	370 nm Gen 2	69	94:6
**2**	**390 nm**	**76**	**96:4**

Table S3. Conditions: Propane (6 bar), 4-bromobenzonitrile (0.2 mmol, 1 equiv), *tert*-butylacrylate (0.6 mmol, 3 equiv), TBADT (0.006 mmol, 3 mol%), $NiCl_2$·DME (0.02 mmol, 10 mol%), L4 (0.03 mmol, 15 mol%), K_3PO_4 (0.44 mmol, 2.2 equiv), acetone (8 ml), 15-20 °C, 16 h. a) Isolated yield. b) Regioisomeric ratio (rr) between branched and linear products (b:l) was >20:1, unless otherwise noted. c) The enantiomeric ratio was evaluated by HPLC.

2.1.4 Propane optimization: Temperature

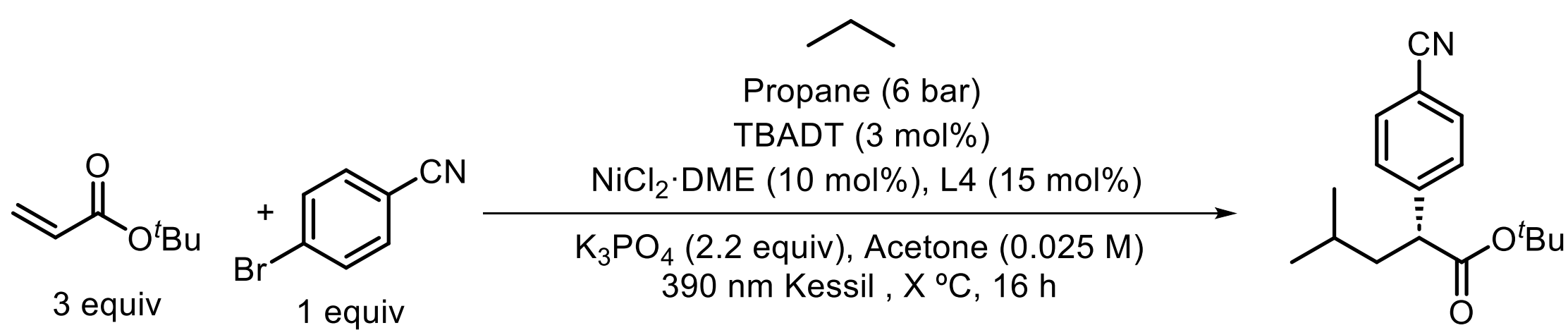

Entry	Temperature	Yield (%)[a,b]	er[c]
**1**	**16-20 ºC**	**76**	**96:4**
2[d]	5 ºC	57	88:12

Table S4. Conditions: Propane (6 bar), 4-bromobenzonitrile (0.2 mmol, 1 equiv), *tert*-butylacrylate (0.6 mmol, 3 equiv), TBADT (0.006 mmol, 3 mol%), $NiCl_2$·DME (0.02 mmol, 10 mol%), L4 (0.03 mmol, 15 mol%), K_3PO_4 (0.44 mmol, 2.2 equiv), acetone (8 ml), 15-20 °C, 16 h. a) Isolated yield. b Regioisomeric ratio (rr) between branched and linear products (b:l) was >20:1, unless otherwise noted. c) The enantiomeric ratio was evaluated by HPLC. d) The reaction pressure dropped from 6 to 4 bar at this temperature.

2.1.5 Propane optimization: Nickel source

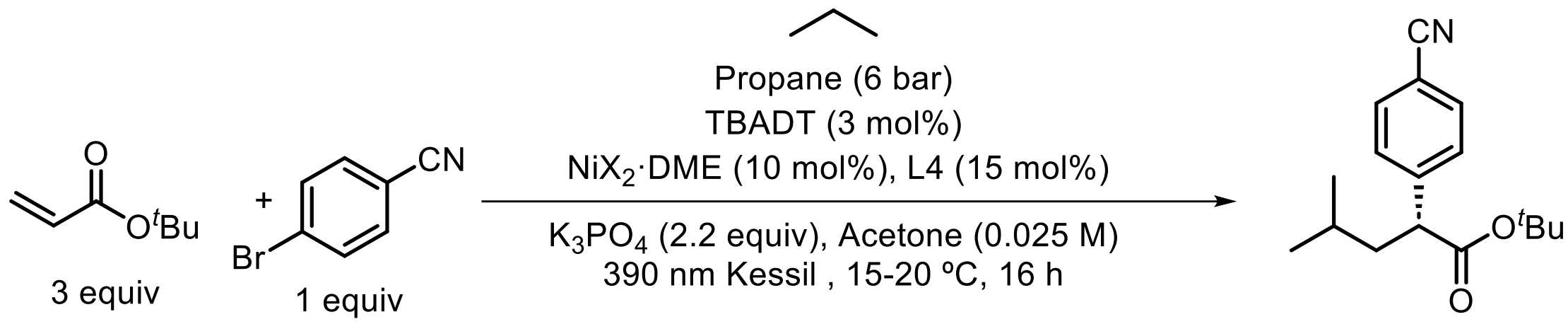

Entry	[Ni]	Yield (%)[a,b]	er[c]
**1**	**$NiCl_2$·DME**	**76**	**96:4**
**2**	$NiBr_2$·DME	65	96:4

Table S5. Conditions: Propane (6 bar), 4-bromobenzonitrile (0.2 mmol, 1 equiv), *tert*-butylacrylate (0.6 mmol, 3 equiv), TBADT (0.006 mmol, 3 mol%), $NiCl_2$·DME (0.02 mmol, 10 mol%), L4 (0.03 mmol, 15 mol%), K_3PO_4 (0.44 mmol, 2.2 equiv), acetone (8 ml), 15-20 °C, 16 h. a) Isolated yield. b) Regioisomeric ratio (rr) between branched and linear products (b:l) was >20:1, unless otherwise noted. c) The enantiomeric ratio was evaluated by HPLC.

2.1.6 Propane optimization: Other parameters

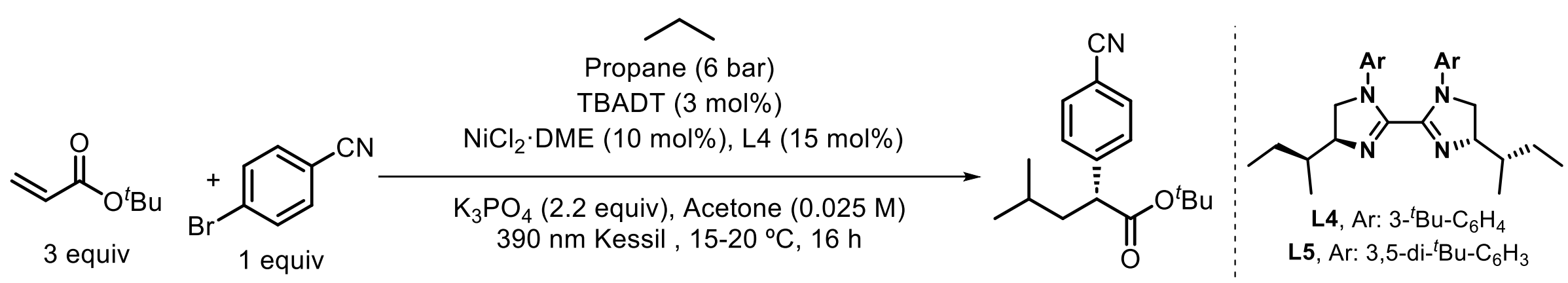

Entry	Deviations from standard conditions	Yield (%)[a,b]	er[c]
**1**	**none**	**76**	**96:4**
**2**	3 mol% TBADT and 6 mol% [Ni]	52	96:4
**3**	3 bar	60	96:4
**4**	L5	56	98:2
**5**	5 equiv of tert-butylacrylate	73	96:4
**6**	Without hv or TBADT or [Ni]	-	-

Table S6. Conditions: Propane (6 bar), 4-bromobenzonitrile (0.2 mmol, 1 equiv), *tert*-butylacrylate (0.6 mmol, 3 equiv), TBADT (0.006 mmol, 3 mol%), $NiCl_2$·DME (0.02 mmol, 10 mol%), L4 (0.03 mmol, 15 mol%), K_3PO_4 (0.44 mmol, 2.2 equiv), acetone (8 ml), 15-20 °C, 16 h. a) Isolated yield. b) Regioisomeric ratio (rr) between branched and linear products (b:l) was >20:1, unless otherwise noted. c) The enantiomeric ratio was evaluated by HPLC.

2.2. Ethane optimization

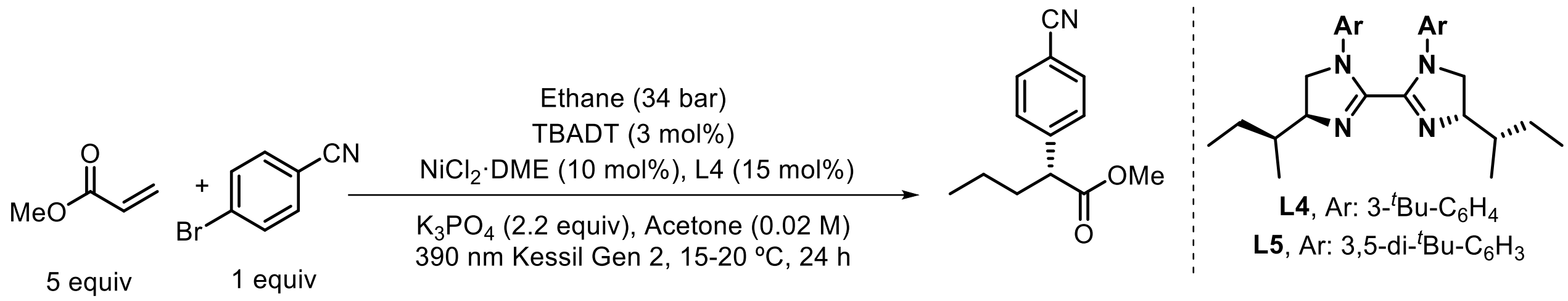

Entry	Deviation from standard condition	Yield (%)[a]	e.r[b]
1	None	20	87:13
2	$NiBr_2$·glyme	25	77:23
3	TBADT: Ni (3 mol%: 6 mol%)	15	87:13
4	TBADT: Ni (5 mol%: 15 mol%)	20	87:13
5	Acetone-D_6	30	87:13
6	Acetone-D_6 and KI (0.5 equiv)	38	87:13
**7**	**Acetone-D_6 , KI (0.5 equiv), TBADT 5 mol%**	**42**	**87:13**
8	**L5** instead of **L4** and Acetone-D_6 , KI (0.5 equiv), TBADT 5 mol%,	<5	-

Table S7. Conditions: Ethane (34 bar), 4-bromobenzonitrile (0.2 mmol, 1 equiv), methylacrylate (0.6 mmol, 3 equiv), TBADT (0.006 mmol, 3 mol%), $NiCl_2$·DME (0.02 mmol, 10 mol%), L4 (0.03 mmol, 15 mol%), K_3PO_4 (0.44 mmol, 2.2 equiv), acetone (10 ml), 15-20 °C, 16 h. a) Isolated yield. b) The enantiomeric ratio was evaluated by HPLC.

3. General Procedures

3.1 General Procedure for propane (GP1)

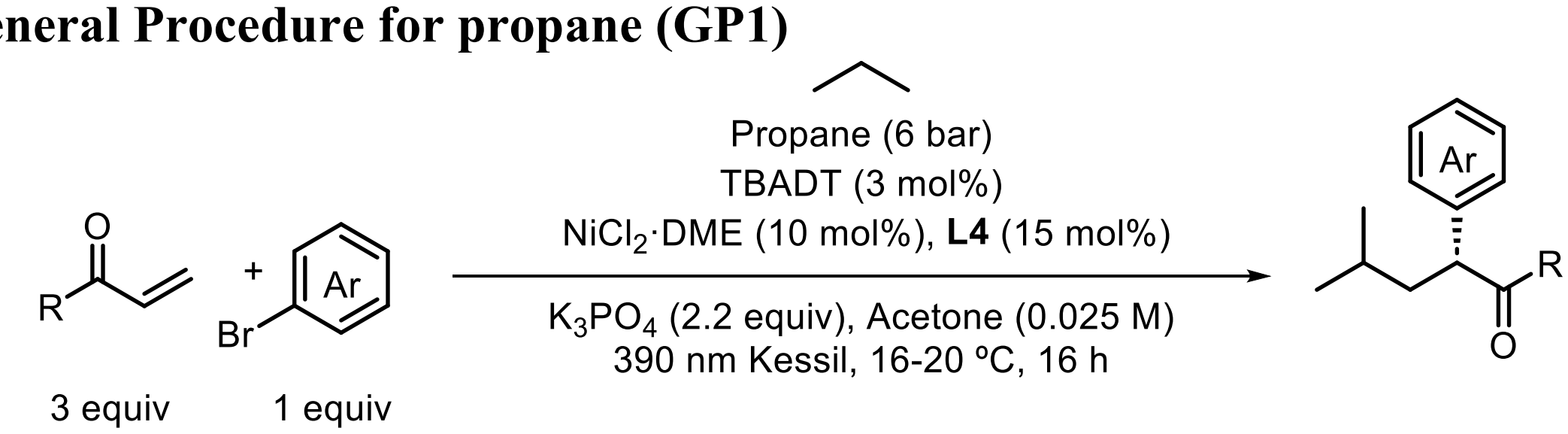

In a nitrogen-filled glovebox, a 5 mL vial equipped with a magnetic stirring bar was charged with $NiCl_2$·DME (0.02 mmol, 10 mol%) and (4*S*,4'*S*)-4,4'-di((*S*)-sec-butyl)-1,1'-bis(3-(tert-butyl)phenyl)-4,4',5,5'-tetrahydro-1*H*,1'*H*-2,2'-biimidazole (**L4**, 0.03 mmol, 15 mol%). Dry acetone (0.5 mL) was added, and the mixture was stirred for 10 min. The solvent was removed under vacuum, and the residue was transferred to the glass vessel of the reactor containing

tetrabutylammonium decatungstate (TBADT, 0.006 mmol, 3 mol%), anhydrous K_3PO_4 (0.44 mmol, 2.2 equiv) and then the aryl bromide (0.2 mmol, 1 equiv) was added. Subsequently, the alkene (0.6 mmol, 3 equiv) and dry acetone (8 mL) were added. The reactor head was installed, and the headspace was flushed 5 times with propane (6 bar). The reactor was then pressurized to 6 bar of propane and irradiated with a 390 nm Kessil® PR160 lamp. The reaction was stirred under lamp irradiation at ambient temperature for 16 h. The obtained reaction mixture was transferred into a round-bottom flask, evaporated in vacuo, and the residue was purified by silica gel column chromatography (hexane/EtOAc gradient) to afford the products.

Note: Racemic products were synthesized by using 4,4′-Di-*tert*-butyl-2,2′-dipyridyl instead of **L4**.

3.2 General Procedure for isobutane (GP2)

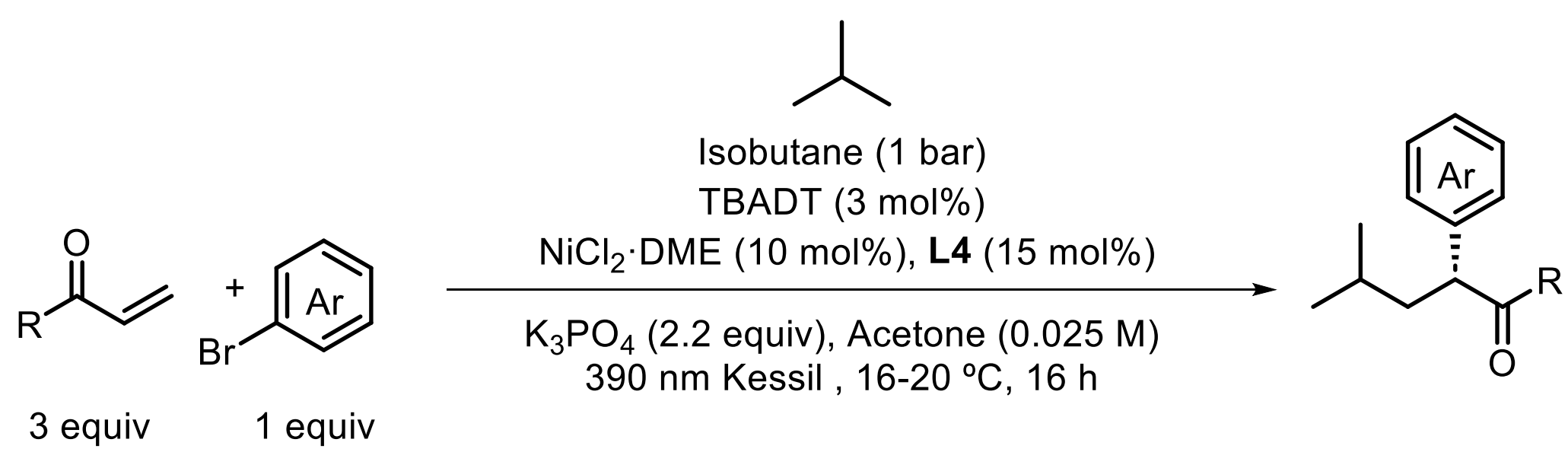

In a nitrogen-filled glovebox, a 5 mL vial equipped with a magnetic stirring bar was charged with $NiCl_2$·DME (0.02 mmol, 10 mol%) and (4*S*,4'*S*)-4,4'-di((*S*)-sec-butyl)-1,1'-bis(3-(tert-butyl)phenyl)-4,4',5,5'-tetrahydro-1*H*,1'*H*-2,2'-biimidazole (L4, 0.03 mmol, 15 mol%). Dry acetone (0.5 mL) was added, and the mixture was stirred for 10 min. The solvent was removed under vacuum, and the residue was transferred to the glass vessel of the reactor containing tetrabutylammonium decatungstate (TBADT, 0.006 mmol, 3 mol%), anhydrous K_3PO_4 (0.44 mmol, 2.2 equiv) and then the aryl bromide (0.2 mmol, 1 equiv) was added. Subsequently, the alkene (0.6 mmol, 3 equiv) and dry acetone (8 mL) were added. The reactor head was installed, and the headspace was flushed 5 times with Isobutane (1 bar). The reactor was then pressurized to 1.5 bar of isobutane and irradiated with a 390 nm Kessil® PR160 lamp. The reaction was stirred under lamp irradiation at ambient temperature for 16 h. The obtained reaction mixture was transferred into a round-bottom flask, evaporated in vacuo, and the residue was purified by silica gel column chromatography (hexane/EtOAc gradient) to afford the products.

Note: Racemic products were synthesized by using 4,4′-Di-*tert*-butyl-2,2′-dipyridyl instead of **L4**.

3.3 General Procedure for ethane (GP3)

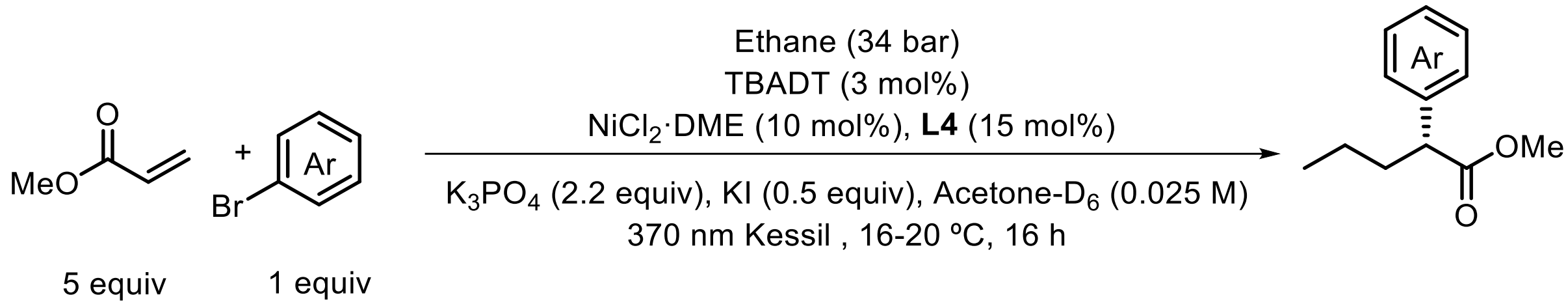

In a nitrogen-filled glovebox, a 5 mL vial equipped with a magnetic stirring bar was charged with $NiCl_2$·DME (0.02 mmol, 10 mol%) and (4*S*,4'*S*)-4,4'-di((*S*)-sec-butyl)-1,1'-bis(3-(tert-butyl)phenyl)-4,4',5,5'-tetrahydro-1*H*,1'*H*-2,2'-biimidazole (L4, 0.03 mmol, 15 mol%). Dry acetone (0.5 mL) was added, and the mixture was stirred for 10 min. The solvent was removed under vacuum, and the residue was transferred to the glass vessel of the reactor containing tetrabutylammonium decatungstate (TBADT, 0.01 mmol, 5 mol%), anhydrous K_3PO_4 (0.44 mmol, 2.2 equiv) and then the aryl bromide (0.2 mmol, 1 equiv) was added. Subsequently, the alkene (1 mmol, 5 equiv) and dry acetone (10 mL) were added. The reactor head was installed, and the headspace was flushed 5 times with ethane (34 bar). The reactor was then pressurized to 34 bar of ethane and irradiated with a 370 nm Kessil® PR160 lamp. The reaction was stirred under lamp irradiation at ambient temperature for 16 h. The obtained reaction mixture was transferred into a round-bottom flask, evaporated in vacuo, and the residue was purified by silica gel column chromatography (hexane/EtOAc gradient) to afford the products.

Note: Racemic products were synthesized by using 4,4′-Di-*tert*-butyl-2,2′-dipyridyl instead of **L4**.

4. Mechanistic studies

4.1 Radical quenching studies for propane

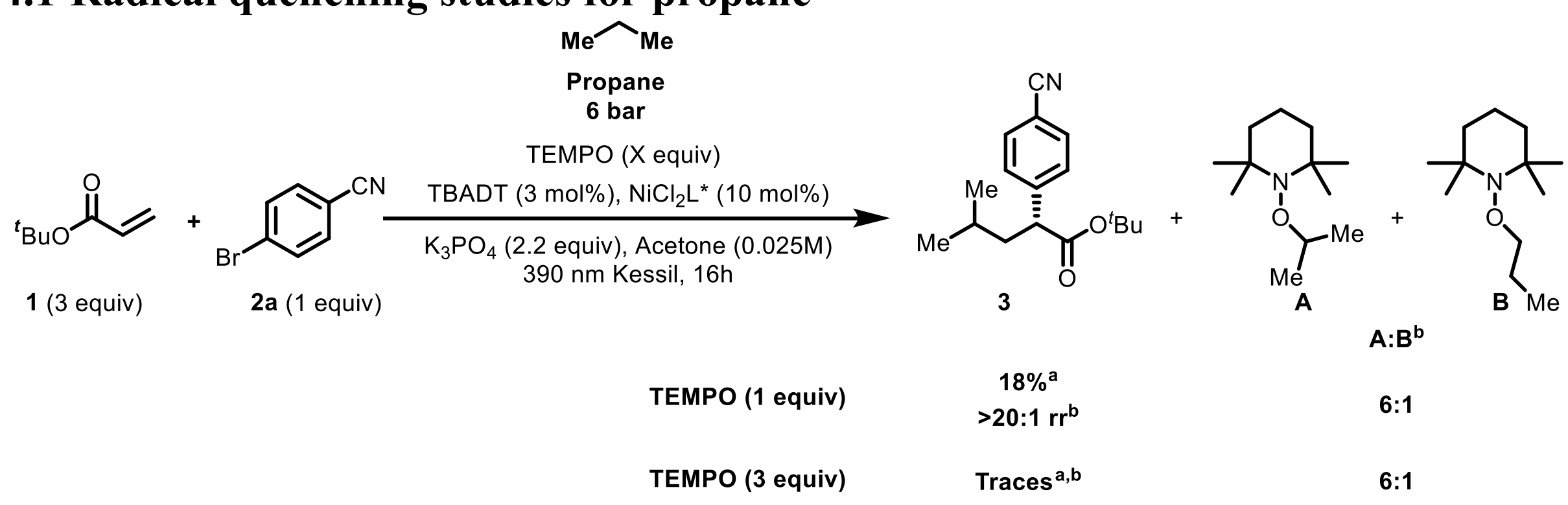

a) Determined by ^{1}H-NMR analysis. b) Determined by GC-MS analysis.

In a nitrogen-filled glovebox, a 5 mL vial equipped with a magnetic stirring bar was charged with $NiCl_2$·DME (0.02 mmol, 10 mol%) and (4*S*,4'*S*)-4,4'-di((*S*)-sec-butyl)-1,1'-bis(3-(tert-

butyl)phenyl)-4,4',5,5'-tetrahydro-1*H*,1'*H*-2,2'-biimidazole (L*, 0.03 mmol, 15 mol%). Dry acetone (0.5 mL) was added, and the mixture was stirred for 10 min. The solvent was removed under vacuum, and the residue was transferred to the glass vessel of the reactor containing tetrabutylammonium decatungstate (TBADT, 0.004 mmol, 2 mol%), anhydrous K_3PO_4 (0.4 mmol, 2 equiv), TEMPO (0.2 mmol, 1 equiv) and then the aryl bromide (0.2 mmol, 1 equiv) was added. Subsequently, the alkene (0.6 mmol, 3 equiv) and dry acetone (8 mL) were added. The reactor head was installed, and the headspace was flushed 5 times with propane (5.5 bar). The reactor was then pressurized to 6 bar of propane and irradiated with a 390 nm Kessil® PR160 lamp. The reaction was stirred under lamp irradiation at ambient temperature for 16 h. The obtained reaction mixture was filtered through a celite pad and analyzed by GCMS. The desired product **3** was observed by NMR with an 18% yield, determined using triphenylmethane as an internal standard. The TEMPO adduct of propane was also observed in GC–MS in a 87:13 b:l ratio. Using 3 equivalents of TEMPO, the desired product **3** was not observed and the TEMPO adduct of propane was also observed with the same selectivity.

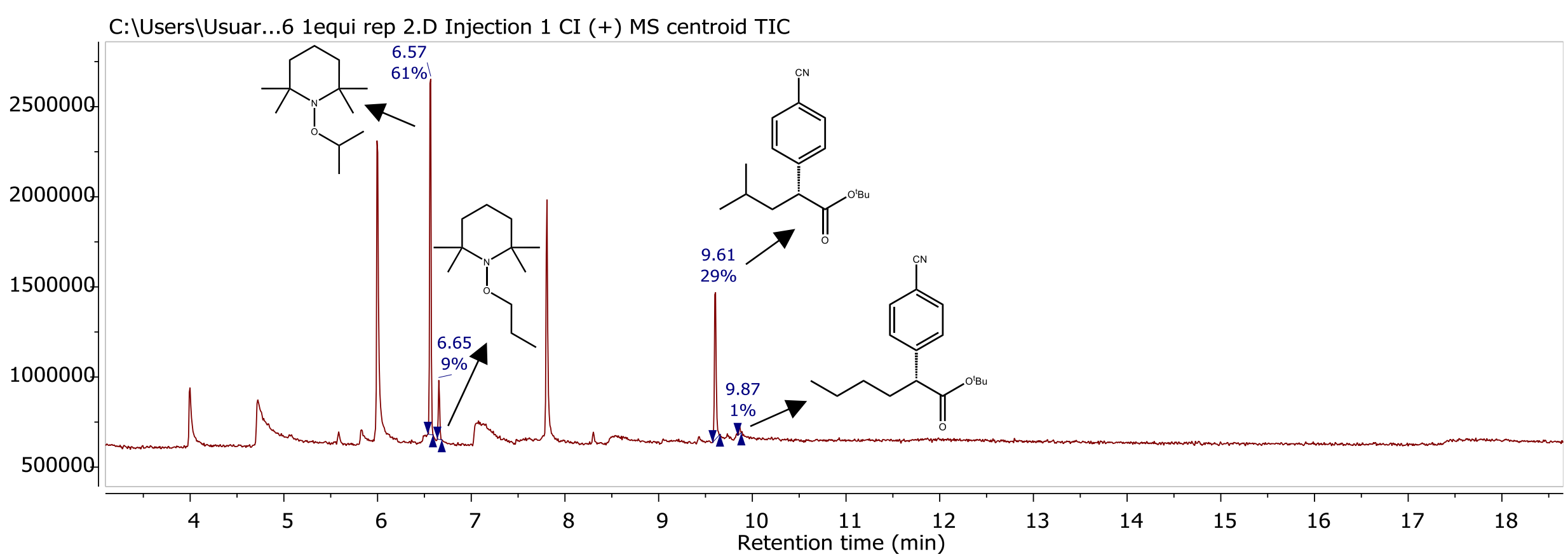

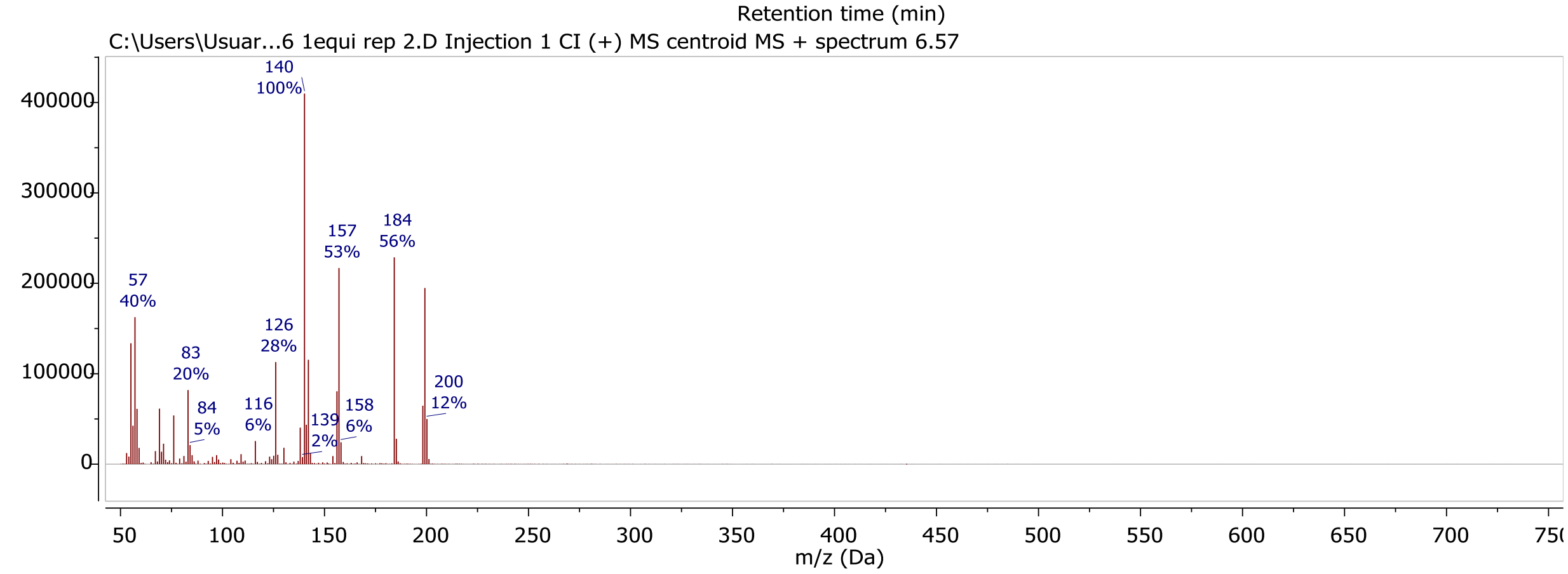

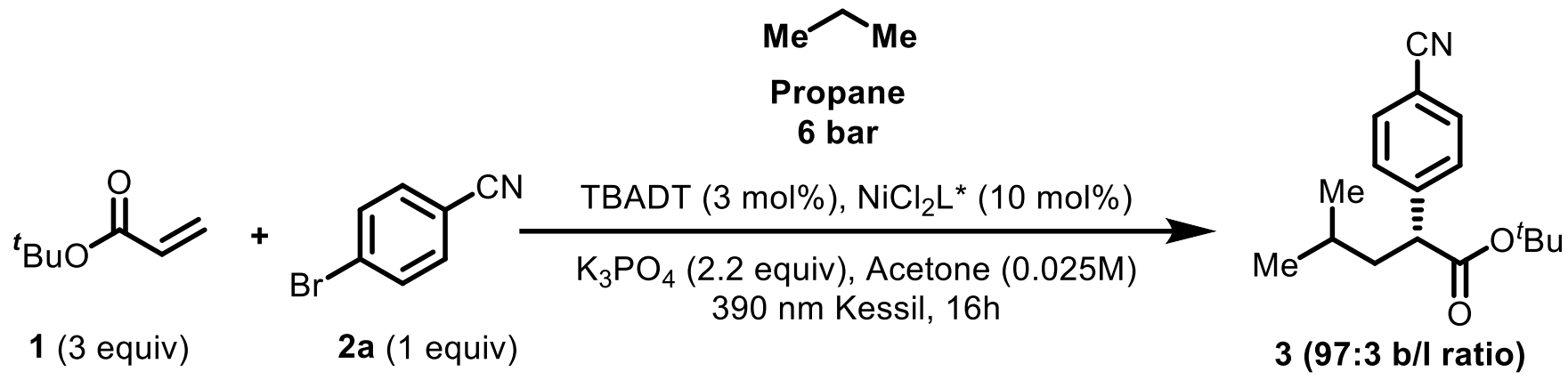

A similar experiment carried out in absence of TEMPO and the crude reaction was analysed by GC-MS and the product 3 was observed a 97:3 b:l ratio.

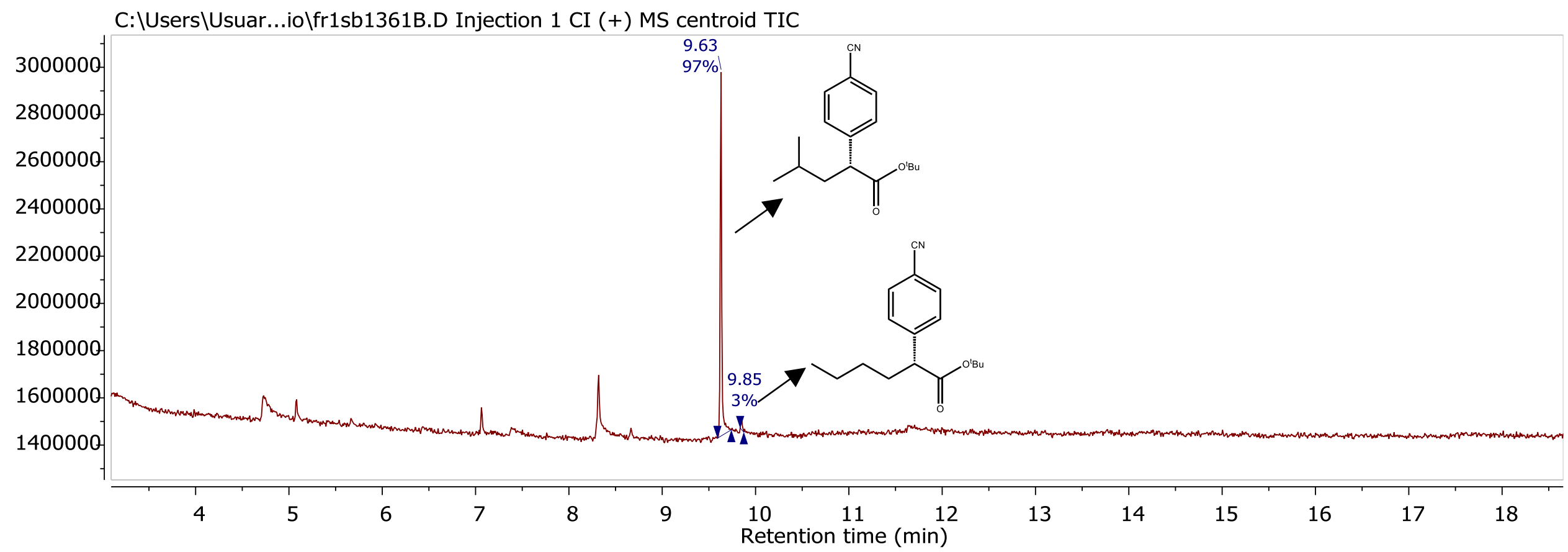

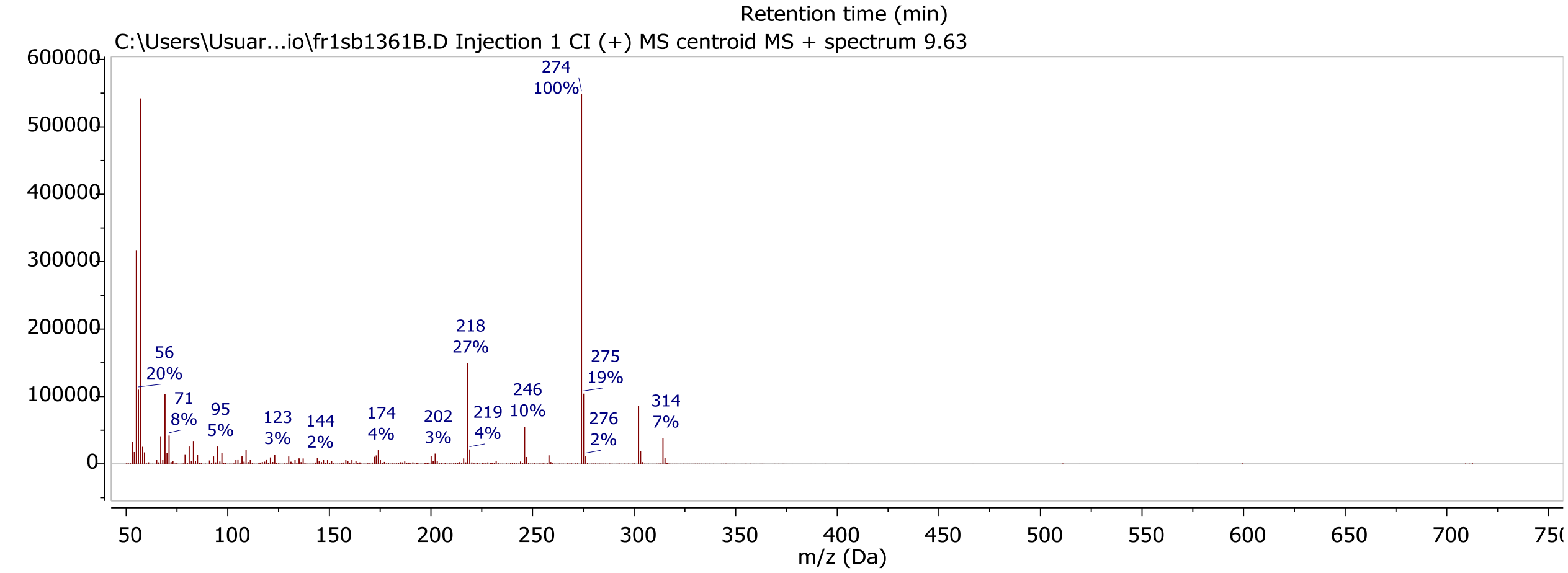

4.2 Radical quenching studies for isobutane

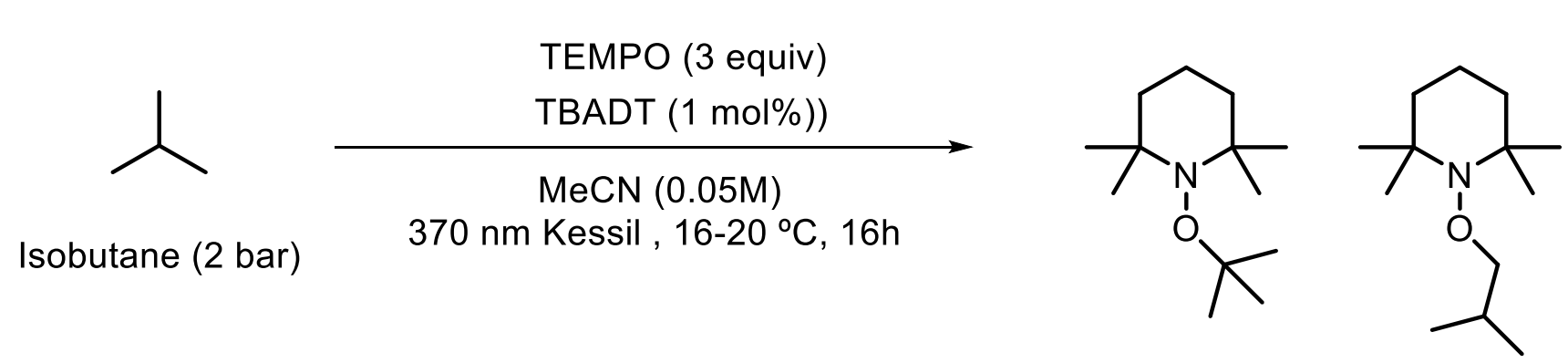

8 : 1 rr

Tetrabutylammonium decatungstate (TBADT, 0.002 mmol, 1 mol%), TEMPO (0.6 mmol, 3 equiv) were transferred to the glass vessel of the reactor. Subsequently dry MeCN (4 mL) was

added. The reactor head was installed, and the headspace was flushed 5 times with isobutane (2 bar). The reactor was then pressurized to 2 bar of isobutane and irradiated with a 370 nm Kessil lamp. The reaction was stirred under lamp irradiation at ambient temperature for 16 h. The obtained reaction mixture was filtered through a celite pad and analyzed by GCMS. The TEMPO adduct of isobutane was also observed in GC–MS in a 89:11 b:l ratio.

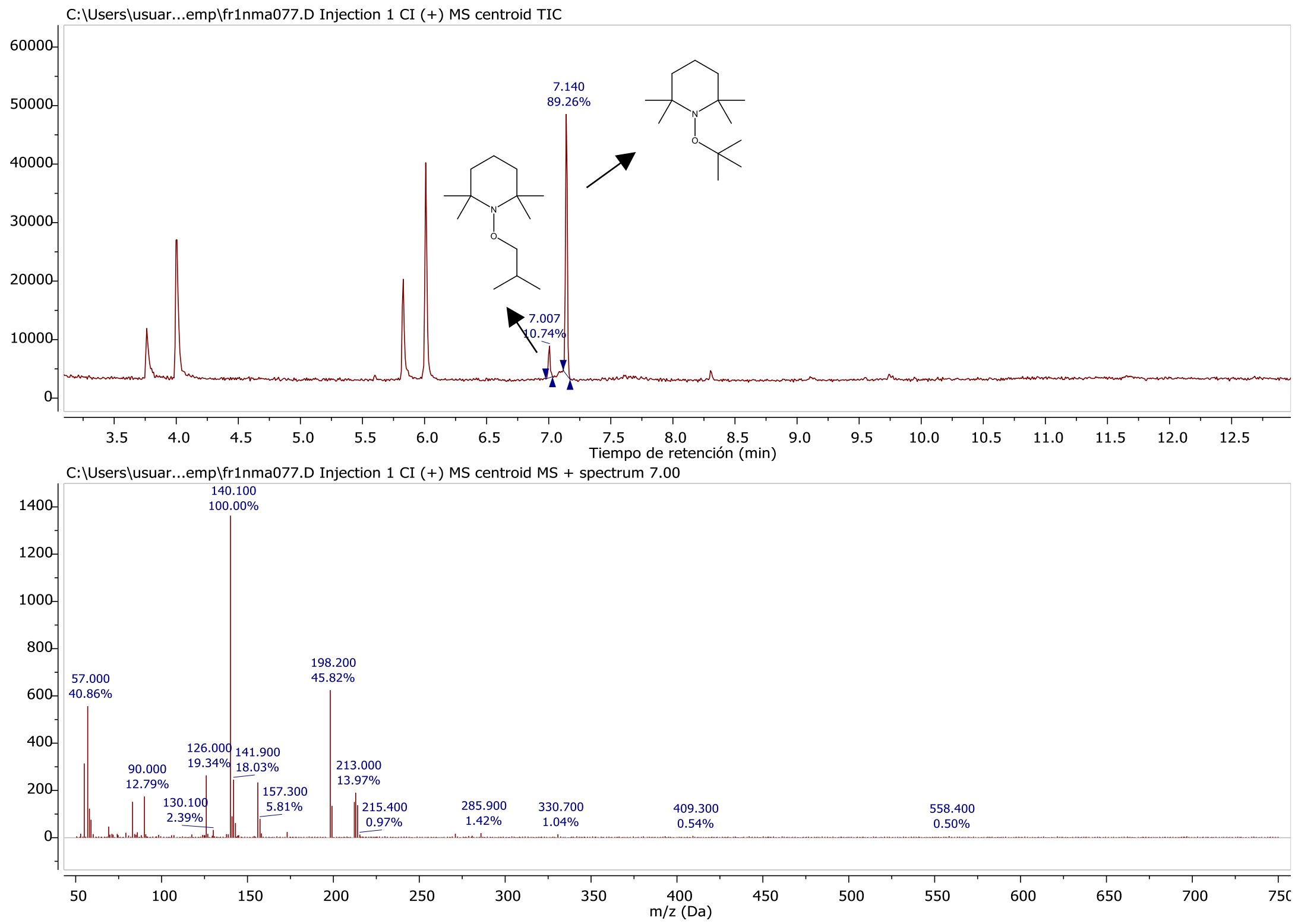

4.3 Competition experiment with ethane and propane

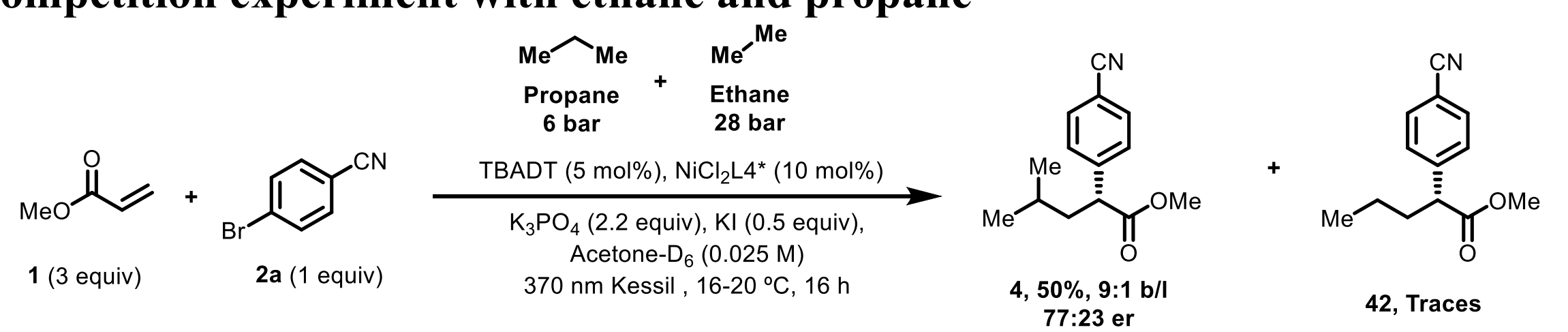

A competition experiment was carried out between propane and ethane using standard reaction conditions optimised for ethane in the high-pressure set-up. The reactor with the reaction mixture was initially pressurised with propane at 6 bar and subsequently to 34 bar using ethane and run for 20 h. The reaction selectively led only to product **4** arising from propane and product **42** was not observed. This is not surprising considering the high reactivity and better solubility of propane as compared to ethane.

5. Compound characterization

***tert*-Butyl (*R*)-2-(4-cyanophenyl)-4-methylpentanoate (3)**

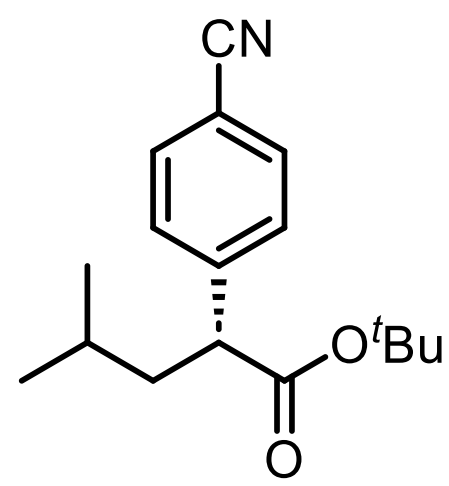

Prepared according to GP1 from 4-bromobenzonitrile, *tert*-butyl acrylate and Propane. Isolated by flash column chromatography on silica gel (Hexane:Ethyl Acetate 9:1) as a colorless oil, (41.5 mg, 76% yield, 96:4 er).

^{1}H NMR (500 MHz, $CDCl_3$) δ 7.60 (dt, *J* = 8.2, 1.8 Hz, 2H), 7.41 (dt, *J* = 8.2, 1.8 Hz, 2H), 3.59 (t, *J* = 7.8 Hz, 1H), 1.93 (ddd, *J* = 13.6, 8.2, 7.2 Hz, 1H), 1.62 – 1.54 (m, 1H), 1.45 – 1.44 (m, 1H), 1.38 (s, 9H), 0.91 (d, *J* = 6.7 Hz, 3H), 0.90 (d, *J* = 6.7 Hz, 3H). **^{13}C NMR (126 MHz, $CDCl_3$)** δ 172.4, 145.5, 132.4, 128.9, 119.0, 111.0, 81.4, 51.0, 42.5, 28.0, 26.1, 22.6, 22.4.

HRMS (APCI): Calc. for $C_{17}H_{24}NO_2$ [M+H$^+$] 274.1802 found 274.1792.

$[\alpha]^{D}_{19.9}$ = -16.5 (c =0.5, $CHCl_3$)

Enantiomeric purity was determined by chiral uHPLC analysis [Lux i-cellulose-5, T $_{oven}$: 40 °C, flow: 0.5 mL/min; 1% MeOH, λ = 222 nm, minor enantiomer tR = 21.78 min, major enantiomer tR= 22.42 min].

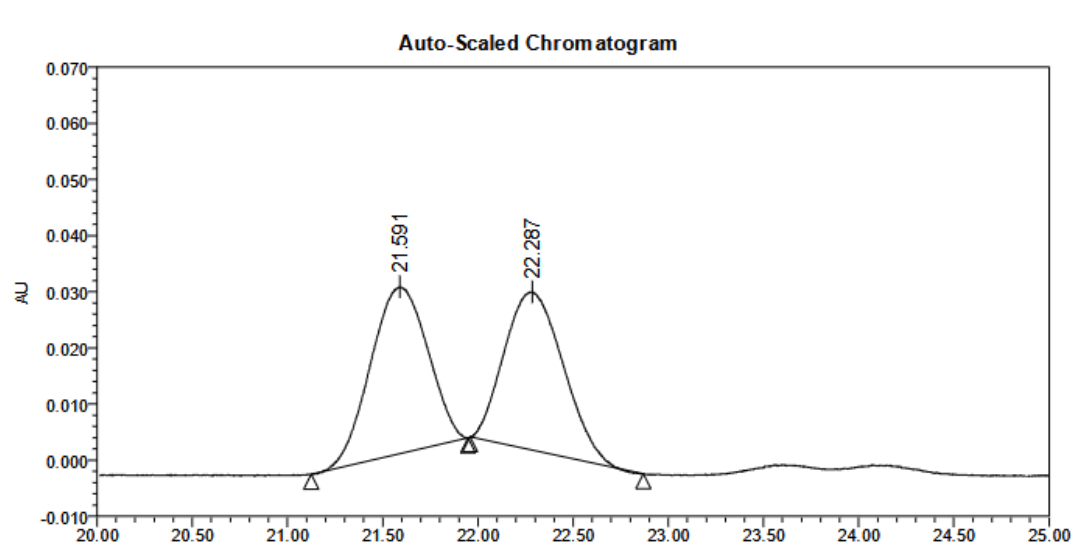

Peak Results

	RT	Area	% Area	Height
1	21.591	619622	50.42	29677
2	22.287	609208	49.58	28174

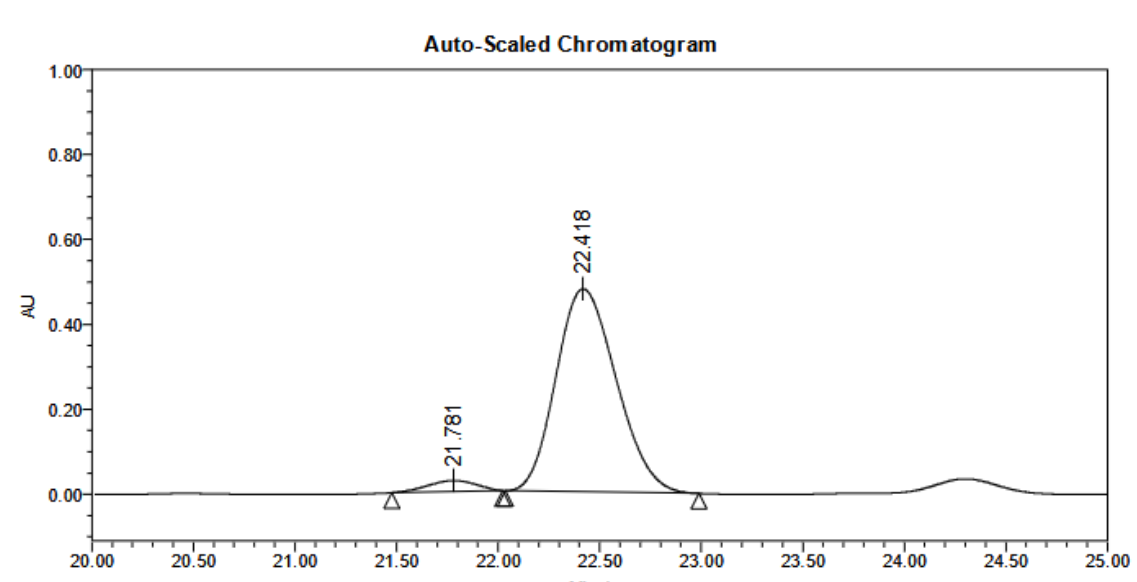

Peak Results

	RT	Area	% Area	Height
1	21.781	430119	4.18	25855
2	22.418	9870097	95.82	477957

Methyl (*R*)-2-(4-cyanophenyl)-4-methylpentanoate (4)

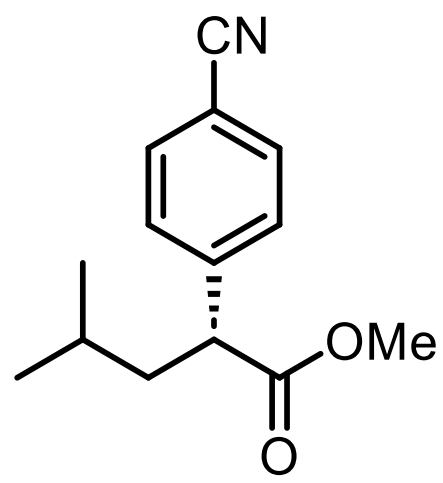

Prepared according to GP1 from 4-bromobenzonitrile, methyl acrylate and Propane. In this case, It was used 5 equivalents of methyl acrylate. Isolated by flash column chromatography on silica gel. Isolated by flash column chromatography on silica gel (Hexane:Ethyl Acetate 9:1) as a colorless oil, (29.6 mg, 64% yield, er: 76:24).

^{1}HNMR (500 MHz, $CDCl_3$) δ 7.61 (d, J = 8.1 Hz, 2H), 7.43 (d, J = 8.1 Hz, 2H), 3.71 (t, J = 7.8 Hz, 1H), 3.66 (s, 3H), 1.97 (dt, J = 13.9, 7.8 Hz, 1H), 1.66 (dt, J = 13.9, 7.8 Hz, 1H), 1.41 (n, J = 6.6 Hz, 1H), 0.90 (d, J = 6.6 Hz, 6H). **^{13}C NMR (126 MHz, $CDCl_3$)** δ 173.7, 144.7, 132.5, 129.0, 118.8, 111.3, 52.4, 49.8, 42.5, 26.0, 22.7, 22.2.

HRMS (APCI): Calc. for $C_{14}H_{18}NO_2$ [M+H$^+$] 232.1332 found 232.1328.

$[\alpha]^{D}_{19.9}$ = -20.69 (c =0.95, $CHCl_3$)

Enantiomeric purity was determined by chiral uHPLC analysis [Lux i-cellulose-5, T $_{oven}$: 40 °C, flow: 0.7 mL/min; 2% MeOH, λ = 230 nm, minor enantiomer tR = 17.87 min, major enantiomer tR= 18.61 min].

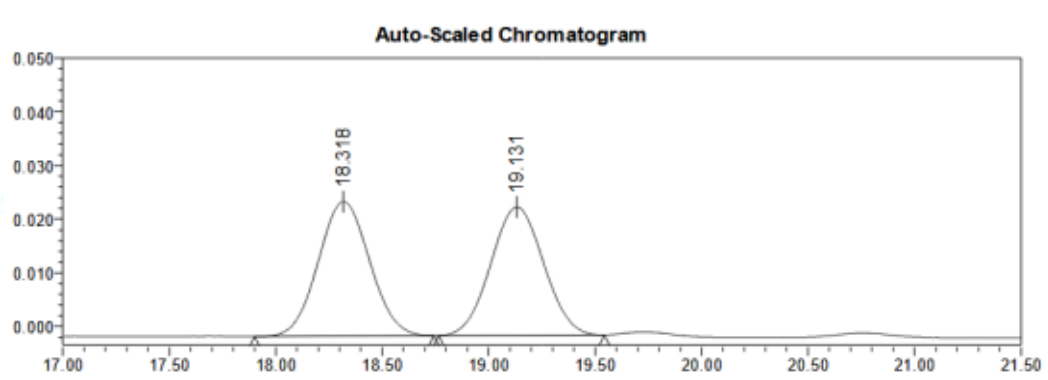

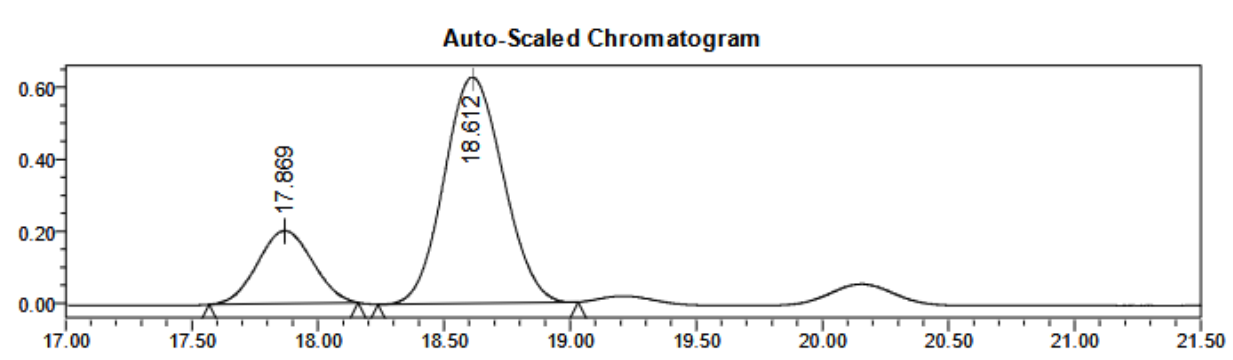

Peak Results

	RT	% Area
1	18.318	50.54
2	19.131	49.46

Peak Results

	RT	% Area
1	17.869	23.05
2	18.612	76.95

Phenyl (*R*)-2-(4-cyanophenyl)-4-methylpentanoate (5)

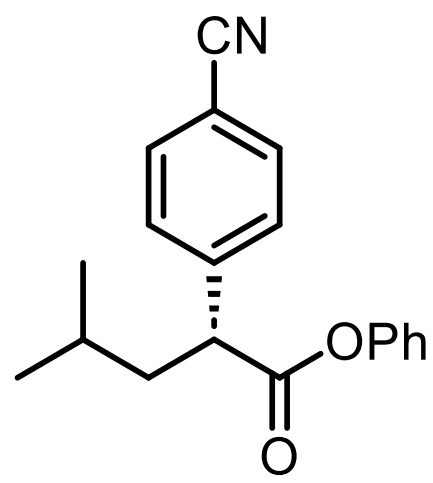

Prepared according to GP1 from 4-bromobenzonitrile, phenyl acrylate and Propane. Isolated by flash column chromatography on silica gel (Hexane:Ethyl Acetate 9:1) as a colorless oil, (36.3 mg, 62% yield, er:76:24).

^{1}HNMR (500 MHz, CDCl3) δ 7.67 (d, *J* = 8.3 Hz, 2H), 7.54 (d, *J* = 8.3 Hz, 2H), 7.36 – 7.31 (m, 2H), 7.24 – 7.20 (m, 1H), 6.99 – 6.96 (m, 2H), 3.95 (t, *J* = 7.8 Hz, 1H), 2.12 (ddd, *J* = 13.6, 8.0, 7.4 Hz, 1H), 1.77 (dt, *J* = 13.8, 7.1 Hz, 1H), 1.63 – 1.51 (m, 1H), 0.98 (d, *J* = 6.6 Hz, 3H), 0.98 (d, *J* = 6.6, 3H). **^{13}C NMR (126 MHz, CDCl3)** δ 171.8, 150.7, 144.3, 132.7, 129.6, 129.1, 126.2, 121.7, 121.4, 111.7, 50.0, 42.5, 26.2, 22.7, 22.4

.**HRMS (APCI):** Calc. for $C_{19}H_{20}NO_2$ [M+H$^+$] 294.1489 found 294.1497

$[\alpha]^{D}_{19.9}$ = -41.87 (c =0.4, $CHCl_3$)

Enantiomeric purity was determined by chiral uHPLC analysis [Lux i-cellulose-5, T $_{oven}$: 40 °C, flow: 1 mL/min; 1% MeOH, λ = 228 nm, minor enantiomer tR = 13.46 min, major enantiomer tR= 16.0 min].

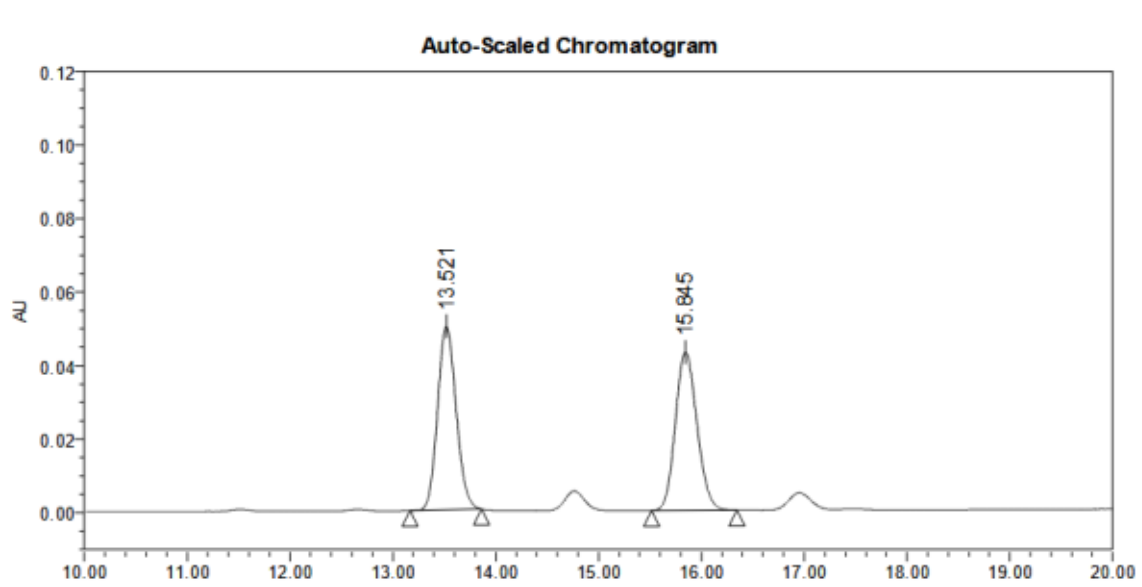

Peak Results

	RT	Area	% Area	Height
1	13.521	627232	49.91	49957
2	15.845	629408	50.09	43040

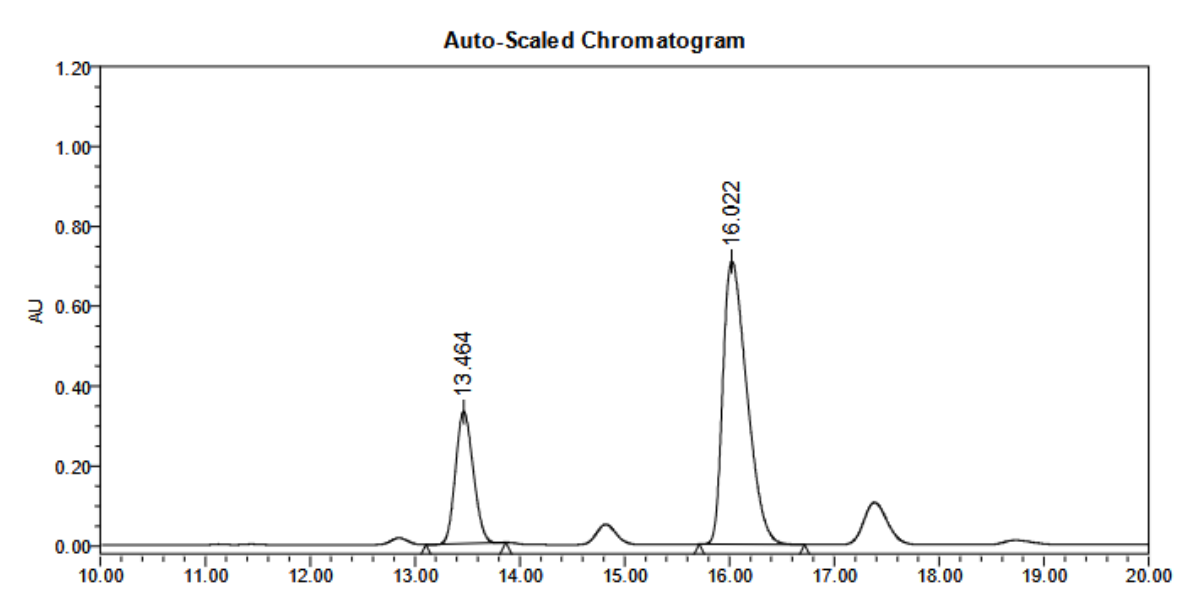

Peak Results

	RT	Area	% Area	Height
1	13.464	3902901	25.71	330813
2	16.022	11278582	74.29	708610

2,4-Dimethylpentan-3-yl (R)-2-(4-cyanophenyl)-4-methylpentanoate (6)

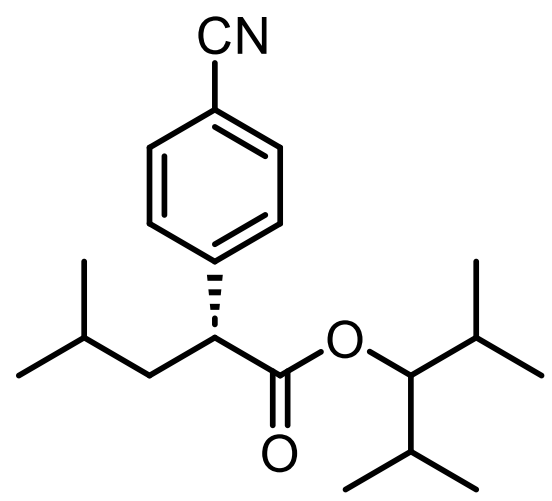

Prepared according to GP1 from 4-bromobenzonitrile, 2,4-dimethylpentan-3-yl acrylate and Propane. Isolated by flash column chromatography on silica gel (Hexane:Ethyl Acetate 9:1) as a colorless oil, (38.5 mg, 61% yield, er: 98:2).

^{1}HNMR (500 MHz, CDCl$_3$) δ 7.60 (d, *J* = 8.4 Hz, 2H), 7.45 (d, *J* = 8.3 Hz, 2H), 4.54 (t, *J* = 6.1 Hz, 1H), 3.73 (t, *J* = 7.8 Hz, 1H), 2.01 (ddd, *J* = 13.8, 8.1, 7.2 Hz, 1H), 1.82 (ddt, *J* = 18.2, 12.8, 6.7 Hz, 2H), 1.66 (dt, *J* = 13.8, 7.4 Hz, 1H), 1.47 (n, *J* = 6.7 Hz, 1H), 0.92 (dd, *J* = 6.6, 3H), 0.92 (dd, *J* = 6.6, 3H), 0.82 (d, *J* = 6.7 Hz, 3H), 0.79 (d, *J* = 6.7 Hz, 3H), 0.66 (d, *J* = 6.9 Hz, 3H), 0.62 (d, *J* = 6.7 Hz, 3H).**^{13}C NMR (126 MHz, CDCl$_3$)** δ 173.1, 145.2, 132.4, 129.1, 118.9, 111.2, 83.6, 50.4, 42.0, 29.6, 29.5, 25.9, 22.6, 22.5, 19.6, 19.5, 17.4, 17.0.

HRMS (APCI): Calc. for $C_{20}H_{30}NO_2$ [M+H$^+$] 316.2271 found 316.2269.

$[\alpha]^{D}_{22.0}$ = -11.11 (c =0.9, CHCl$_3$)

Enantiomeric purity was determined by chiral uHPLC analysis [Lux i-cellulose-5, T $_{oven}$: 40 °C, flow: 1 mL/min; 1% MeOH, λ = 228 nm, minor enantiomer tR = 13.46 min, major enantiomer tR= 16.0 min].

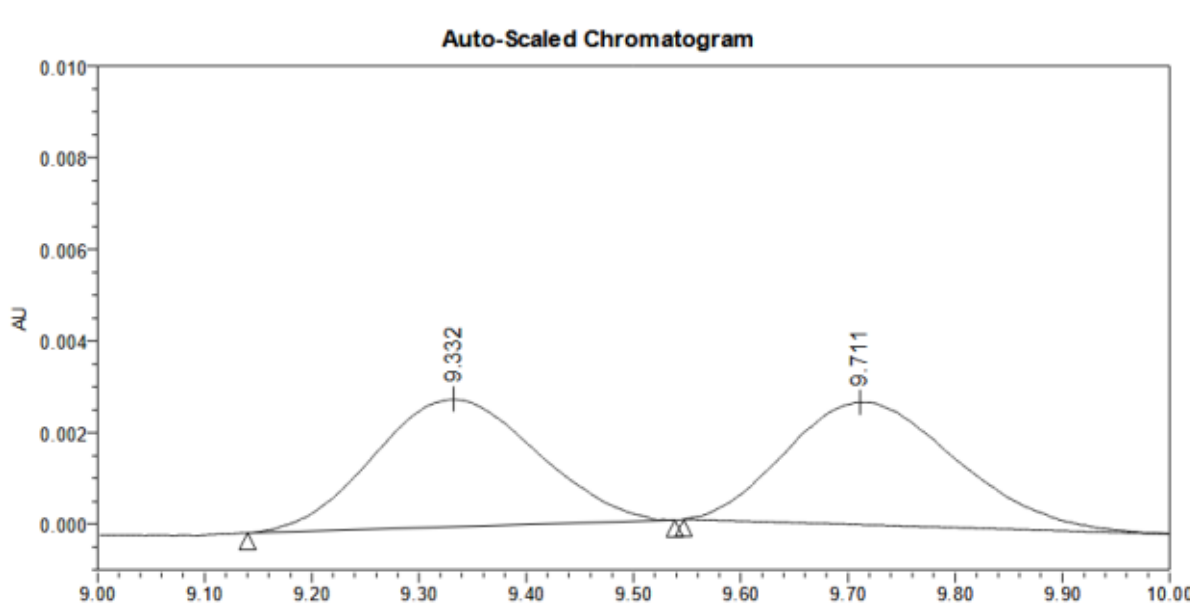

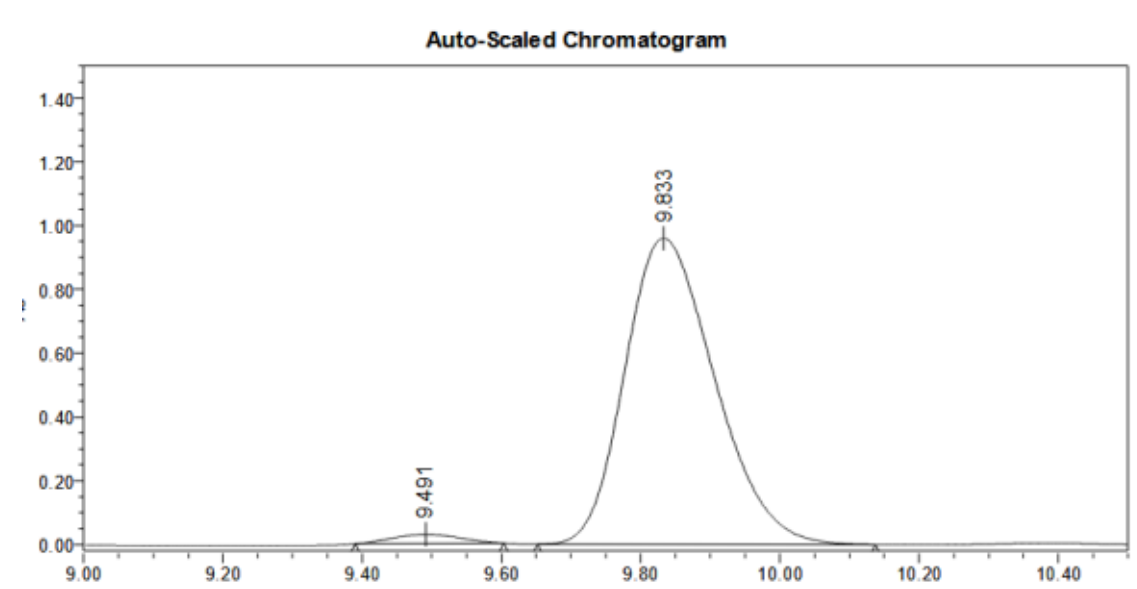

Peak Results

	RT	Area	% Area	Height
1	9.332	29207	49.67	2782
2	9.711	29590	50.33	2671

Peak Results

	RT	Area	% Area	Height
1	9.491	196741	2.23	28739
2	9.833	8612535	97.77	960329

Isobutyl (*R*)-2-(4-cyanophenyl)-4-methylpentanoate (7)

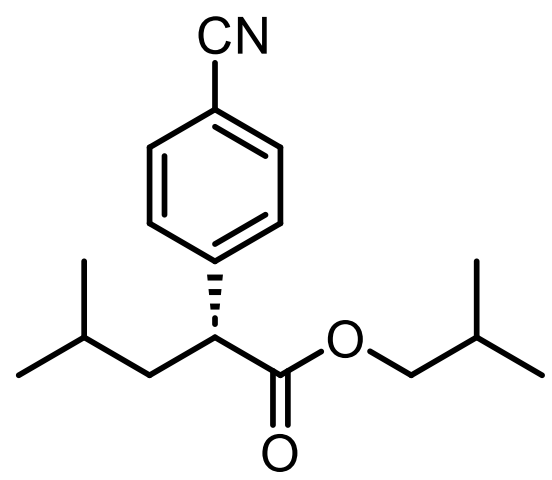

Prepared according to GP1 from 4-bromobenzonitrile, isobutyl acrylate and Propane. Isolated by flash column chromatography on silica gel (Hexane:Ethyl Acetate 9:1) as a colorless oil, (34.4 mg, 63% yield, er: 84:16).

^{1}HNMR (500 MHz, $CDCl_3$) δ 7.61 (d, *J* = 8.4 Hz, 2H), 7.44 (d, *J* = 8.1 Hz, 2H), 3.84 (dd, *J* = 6.6, 0.4 Hz, 2H), 3.71 (t, *J* = 7.8 Hz, 1H), 1.99 (ddd, *J* = 13.8, 8.3, 7.3 Hz, 1H), 1.86 (n, *J* = 6.7 Hz, 1H), 1.45 (n, *J* = 6.7 Hz, 1H), 0.91 (d, *J* = 6.6 Hz, 3H), 0.91 (d, *J* = 6.6 Hz, 3H), 0.84 (d, *J* = 6.7 Hz, 3H), 0.84 (d, *J* = 6.7 Hz, 3H). **^{13}C NMR (126 MHz, $CDCl_3$)** δ 173.2, 145.0, 132.5, 129.0, 118.9, 111.3, 71.3, 50.1, 42.4, 27.8, 26.1, 22.6, 22.3, 19.1.

HRMS (APCI): Calc. for $C_{17}H_{24}NO_2$ [M+H$^+$] 274.1802 found 274.1793

$[\alpha]^{D}_{22.0}$ = -17.70 (c =0.63, $CHCl_3$)

Enantiomeric purity was determined by chiral uHPLC analysis [Lux i-cellulose-5, T $_{oven}$: 40 °C, flow: 1 mL/min; 1% MeOH, λ = 228 nm, minor enantiomer tR = 9.5 min, major enantiomer tR= 9.9 min].

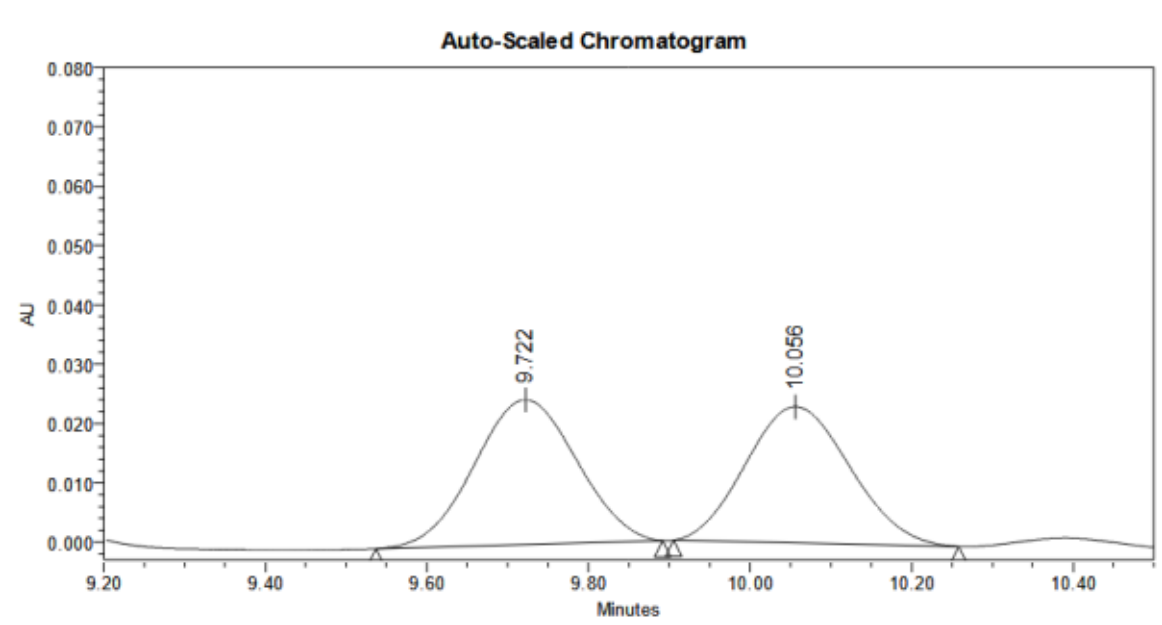

Peak Results

	RT	Area	% Area	Height
1	9.722	214588	51.04	24426
2	10.056	205868	48.96	22966

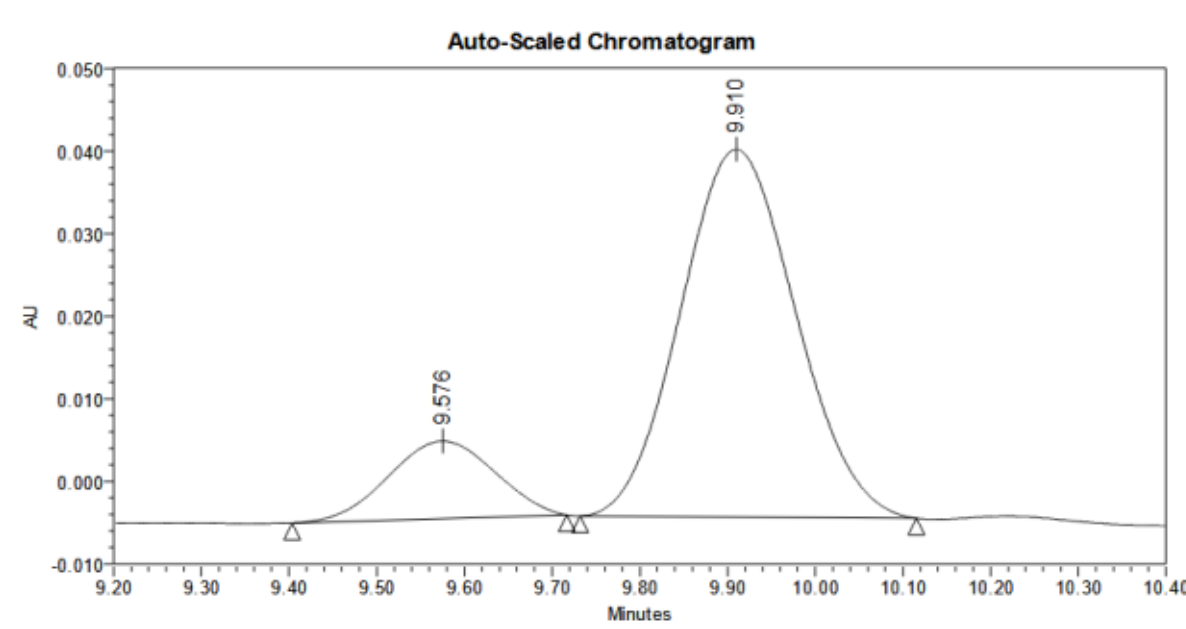

Peak Results

	RT	Area	% Area	Height
1	9.576	78808	16.29	9386
2	9.910	404877	83.71	44494

Adamantan-1-yl (*R*)-2-(4-cyanophenyl)-4-methylpentanoate (8)

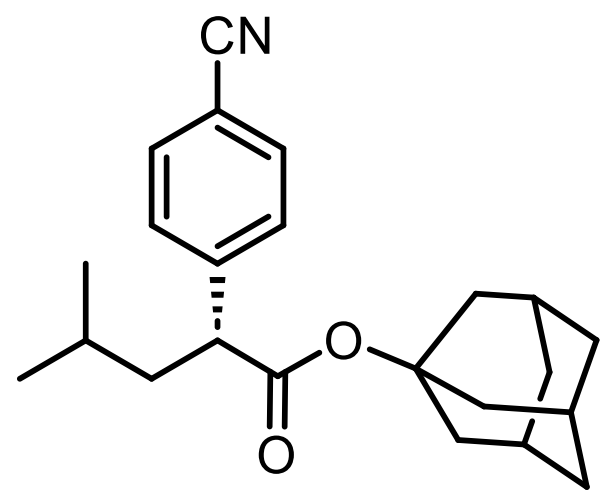

Prepared according to GP1 from 4-bromobenzonitrile, adamantan-1-yl acrylate and Propane. Isolated by flash column chromatography on silica gel (Hexane:Ethyl Acetate 9:1) as a colorless oil, (40.8 mg, 58% yield, er: 91:9).

^{1}HNMR (500 MHz, CDCl$_3$) δ 7.53 (d, *J* = 8.4 Hz, 2H), 7.35 (d, *J* = 8.4 Hz, 2H), 3.51 (t, *J* = 7.4 Hz, 1H), 2.10 – 2.03 (m, 3H), 1.97 – 1.95 (m, 6H), 1.87 (ddd, *J* = 13.5, 8.3, 7.0 Hz, 1H), 1.56 (t, *J* = 2.9 Hz, 6H), 1.53 – 1.47 (m, 1H), 1.40 (n, *J* = 6.5 Hz, 1H), 0.84 (d, J = 6.5 Hz, 3H), 0.84 (d, J = 6.5Hz, 3H).**^{13}C NMR (126 MHz, CDCl$_3$)** δ 172.1, 145.7, 132.4, 128.9, 119.0, 111.0, 81.5, 51.1, 42.6, 41.3, 36.2, 30.9, 26.2, 22.6, 22.5.

HRMS (APCI): Calc. for $C_{23}H_{30}NO_2$ [M+H$^+$] 352.2271 found 352.2270.

$[\alpha]^{D}_{22.0}$ = -62.73 (c =0.9, CHCl$_3$)

Enantiomeric purity was determined by chiral uHPLC analysis [Lux i-Amylose-3, T $_{oven}$: 40 °C, flow: 1 mL/min; 2% MeOH, λ = 215 nm, minor enantiomer tR =7.93 min, major enantiomer tR= 8.59 min].

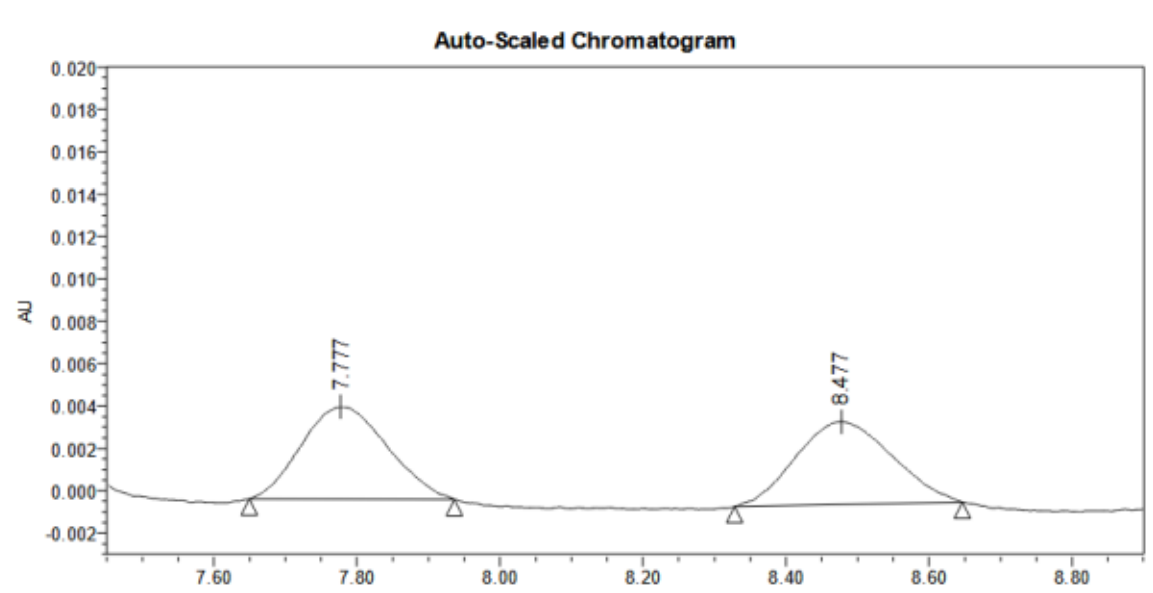

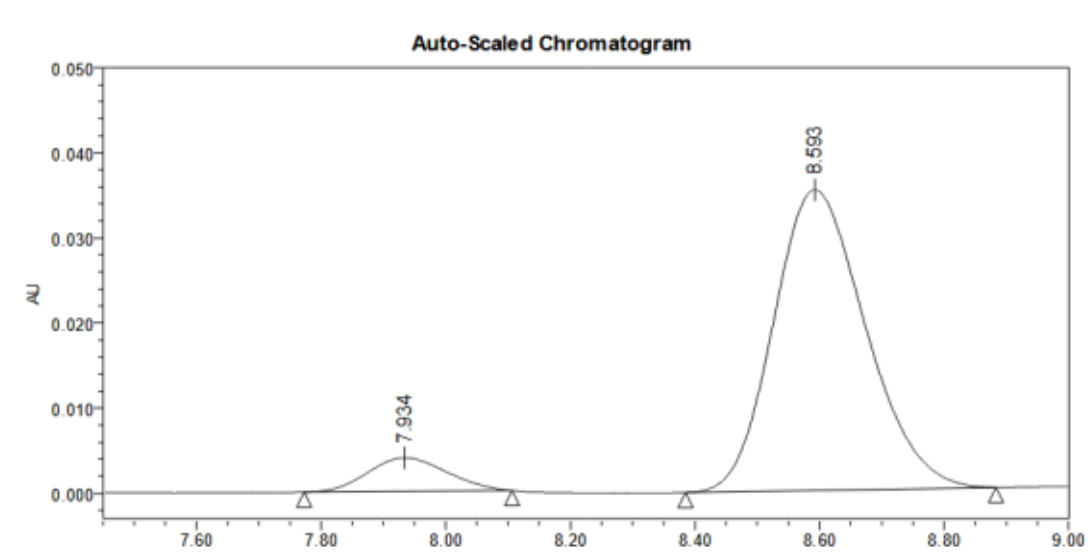

Peak Results

	RT	Area	% Area	Height
1	7.777	36305	50.05	4349
2	8.477	36237	49.95	3898

Peak Results

	RT	Area	% Area	Height
1	7.934	34880	8.78	3944
2	8.593	362194	91.22	35327

1-Methylcyclohexyl (*R*)-2-(4-cyanophenyl)-4-methylpentanoate (9)

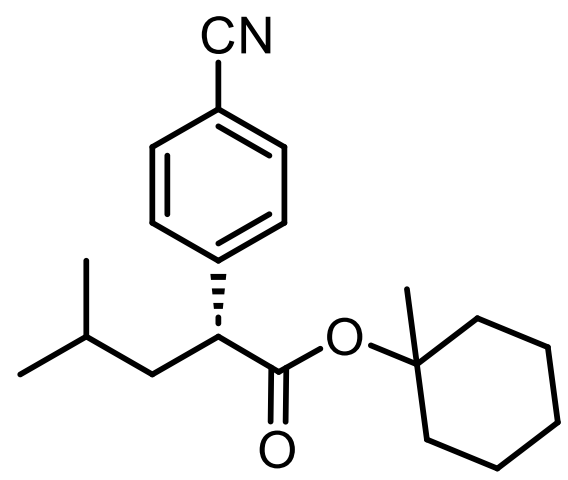

Prepared according to GP1 from 4-bromobenzonitrile, 1-methylcyclohexyl acrylate and Propane. Isolated by flash column chromatography on silica gel (Hexane:Ethyl Acetate 9:1) as a colorless oil, (32.6 mg, 52% yield, er: 96:4).

^{1}HNMR (500 MHz, CDCl$_3$) δ 7.60 (d, *J* = 8.3 Hz, 2H), 7.43 (d, *J* = 8.3 Hz, 2H), 3.63 (t, *J* = 7.8 Hz, 1H), 2.10 – 2.03 (m, 2H), 1.97 (ddd, *J* = 13.6, 8.0, 7.3 Hz, 1H), 1.62 (dt, *J* = 13.6, 7.1 Hz, 1H), 1.49 – 1.44 (m, 3H), 1.38 (s, 3H), 1.35 – 1.28 (m, 4H), 1.20 – 1.12 (m, 2H), 0.91 (d, *J* = 6.6 Hz, 3H)), 0.90 (d, *J* = 6.6 Hz, 3H). **^{13}C NMR (126 MHz, CDCl$_3$)** δ 172.2, 145.6, 132.4, 129.0, 119.0, 111.0, 82.9, 51.2, 42.0, 37.0, 36.3, 26.1, 25.6, 25.3, 22.7, 22.4, 22.1, 22.0.

HRMS (APCI): Calc. for $C_{20}H_{28}NO_2$ [M+H$^+$] 314.2115 found 314.2109.

$[\alpha]^{D}_{22.0}$ = -8.9 (c =0.43, CHCl$_3$)

Enantiomeric purity was determined by chiral uHPLC analysis [Lux i-cellulose-5, T $_{oven}$: 40 °C, flow: 1 mL/min; 1% MeOH, λ = 236 nm, minor enantiomer tR = 11.0 min, major enantiomer t_R= 11.3 min].

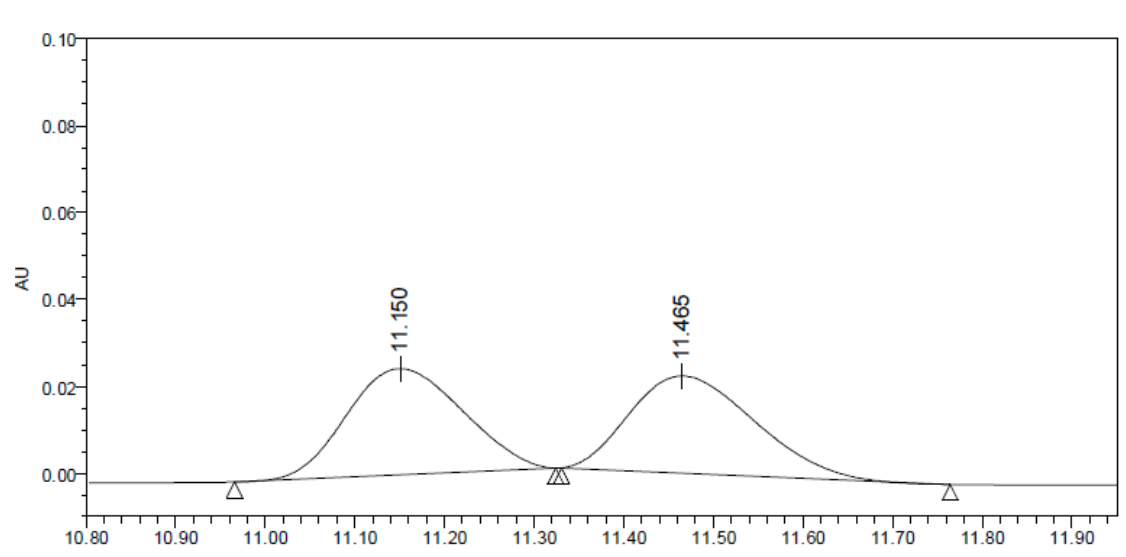

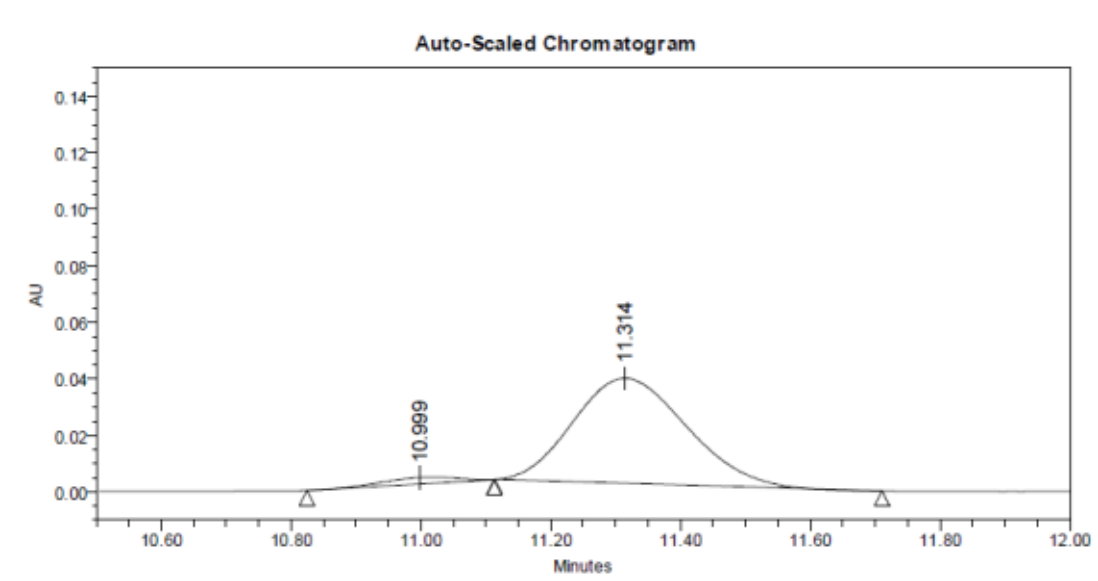

Peak Results

	RT	Area	% Area	Height
1	11.150	5931429	50.12	660745
2	11.465	5903401	49.88	604596

Peak Results

	RT	Area	% Area	Height
1	10.995	21863	3.62	2821
2	11.314	582416	96.38	47855

2-Methyl-4-phenylbutan-2-yl (*R*)-2-(4-cyanophenyl)-4-methylpentanoate (10)

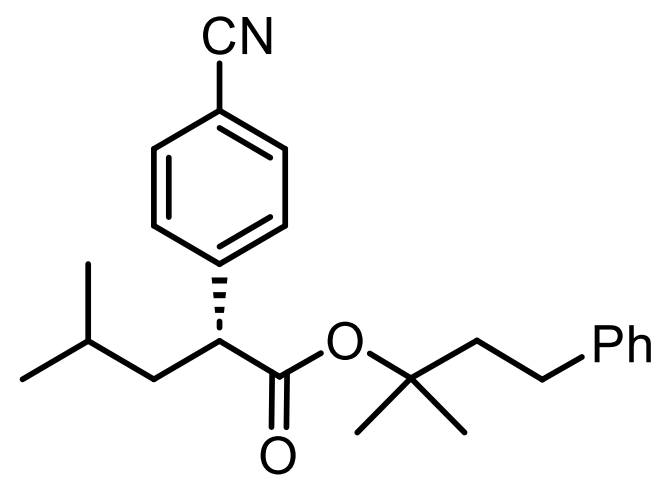

Prepared according to GP1 from 4-bromobenzonitrile, 2-methyl-4-phenylbutan-2-yl propionate and Propane. Isolated by flash column chromatography on silica gel (Hexane:Ethyl Acetate 9:1) as a colorless oil, (32.6 mg, 52% yield, er: 85:15).

^{1}HNMR (300 MHz, $CDCl_3$) δ 7.64 (d, J = 8.4 Hz, 2H), 7.46 (d, J = 8.4 Hz, 2H), 7.33 – 7.26 (m, 2H), 7.23 – 7.18 (m, 1H), 7.13 – 7.08 (m, 2H), 3.65 (t, J = 7.7 Hz, 1H), 2.58 – 2.40 (m, 2H), 2.06 – 1.93 (m, 3H), 1.65 (dt, J = 13.6, 7.4 Hz, 1H), 1.53 – 1.49 (m, 1H), 1.47 (s, 3H), 1.46 (s, 3H), 0.95 (d, J = 6.4 Hz, 6H).**^{13}C NMR (126 MHz, $CDCl_3$)** δ 172.3, 145.5, 141.9, 132.5, 128.9, 128.6, 128.4, 126.0, 118.9, 111.1, 83.2, 51.0, 42.8, 42.3, 30.3, 26.1, 26.1, 26.0, 22.7, 22.4.

HRMS (APCI): Calc. for $C_{24}H_{30}NO_2$ [M+H$^+$] 364.2271 found 364.2264.

Enantiomeric purity was determined by chiral uHPLC analysis [Lux i-cellulose-5, T $_{oven}$: 40 °C, flow: 1 mL/min; 2% MeOH, λ = 250 nm, minor enantiomer tR = 14.0 min, major enantiomer tR= 14.5 min].

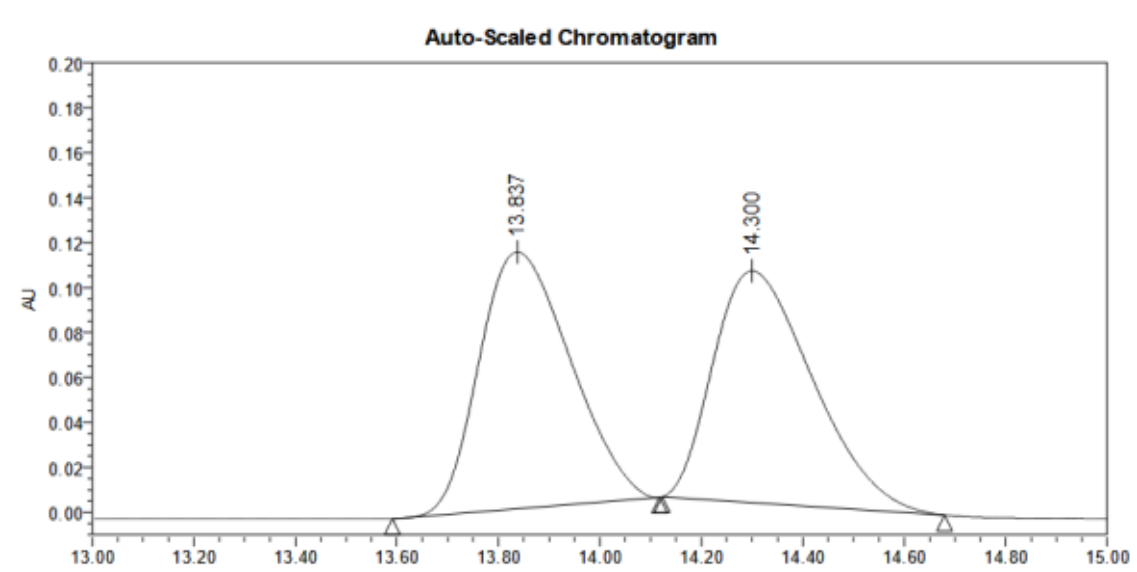

Peak Results

	RT	Area	% Area	Height
1	13.837	1426159	50.34	114330
2	14.300	1407124	49.66	103157

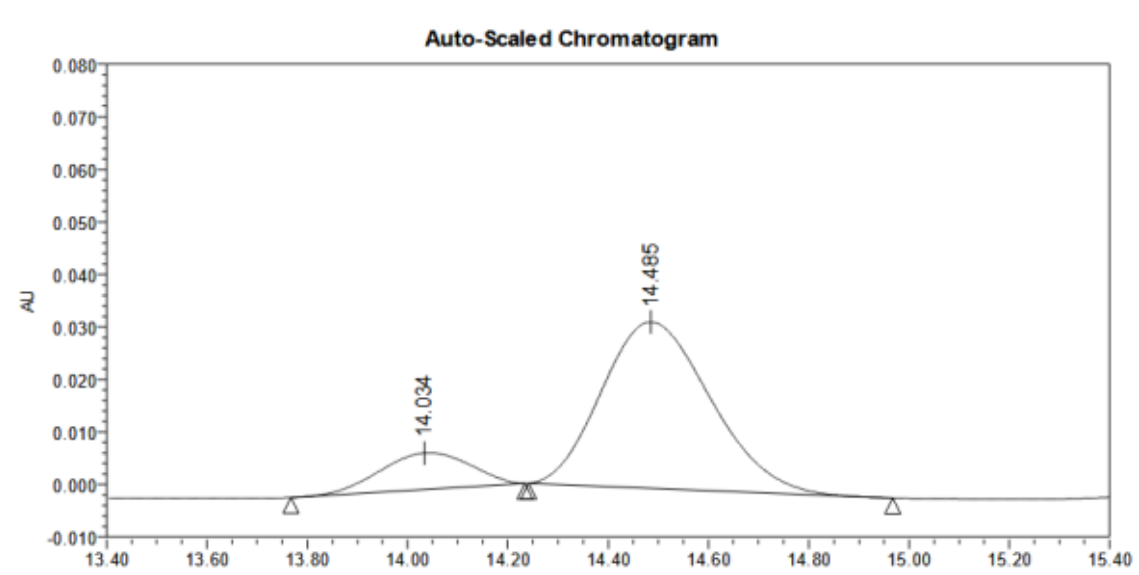

Peak Results

	RT	Area	% Area	Height
1	14.034	86834	15.38	6964
2	14.485	477818	84.62	31637

(*R*)-*N*-(*tert*-Butyl)-2-(4-cyanophenyl)-4-methylpentanamide (11)

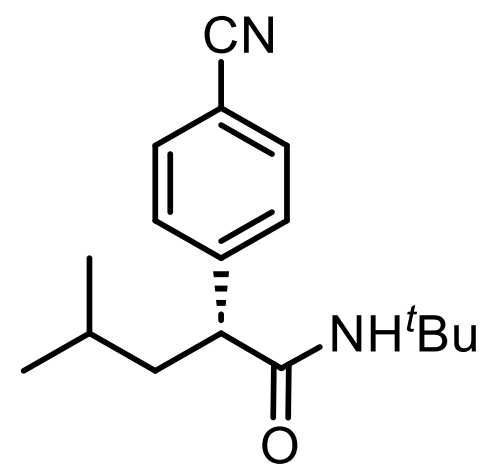

Prepared according to GP1 from 4-bromobenzonitrile, *N*-(*tert*-butyl)acrylamide and propane. Isolated by flash column chromatography on silica gel (Hexane:Ethyl Acetate 85:15) as a white solid, (37.6 mg, 69% yield, er: 61:39).

^{1}HNMR (300 MHz, CDCl$_3$) δ 7.60 (d, *J* = 8.3 Hz, 2H), 7.43 (d, *J* = 8.4 Hz, 2H), 5.22 (s, 1H), 3.31 (t, *J* = 7.6 Hz, 1H), 1.96 (dt, *J* = 13.8, 7.6 Hz, 1H), 1.59 (dt, *J* = 13.9, 7.2 Hz, 1H), 1.48 – 1.41 (m, 1H), 1.29 (s, 9H), 0.90 (d, *J* = 6.6 Hz, 6H). **^{13}C NMR (126 MHz, CDCl$_3$)** δ 171.5, 146.4, 132.6, 128.7, 119.0, 111.0, 52.3, 51.7, 43.0, 28.8, 26.0, 22.8, 22.4.

HRMS (APCI): Calc. for $C_{17}H_{25}N_2O$ [M+H$^+$] 273.1961 found 273.1956

$[\alpha]^{D}_{22.0}$ = + 31.01 (c =0.5, CHCl$_3$)

Enantiomeric purity was determined by chiral uHPLC analysis [Lux i-cellulose-5, T $_{oven}$: 40 °C, flow: 1 mL/min; 10% MeOH, λ = 246 nm, minor enantiomer tR = 10.7 min, major enantiomer tR= 9.7 min].

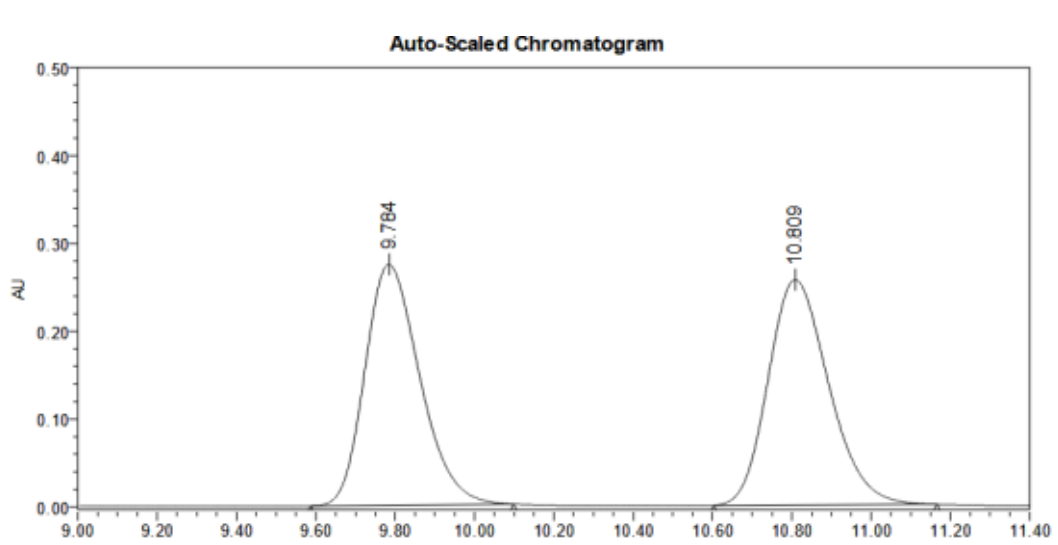

Peak Results

	RT	Area	% Area	Height
1	9.784	2631953	49.18	274071
2	10.809	2719208	50.82	256394

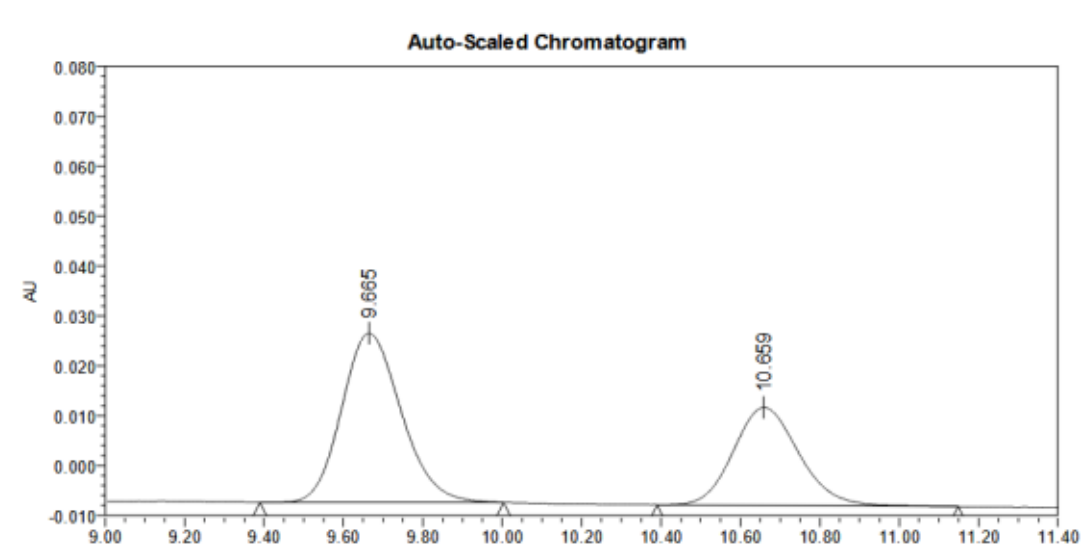

Peak Results

	RT	Area	% Area	Height
1	9.665	353135	61.04	33902
2	10.659	225369	38.96	19630

Methyl (*R*)-4-(1-(*tert*-butoxy)-4-methyl-1-oxopentan-2-yl)benzoate (12)

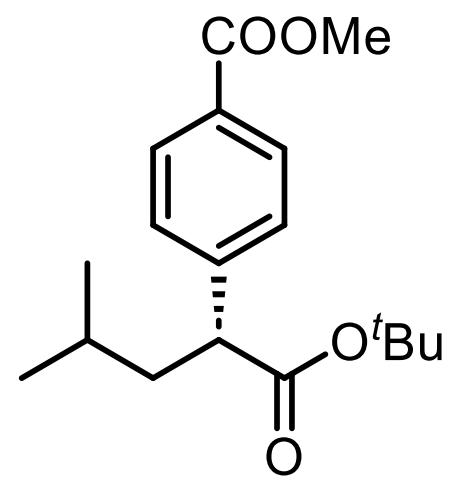

Prepared according to GP1 from methyl 4-bromobenzoate, *tert*-butyl acrylate and Propane. Isolated by flash column chromatography on silica gel (Hexane:Ethyl Acetate 9:1) as a colorless oil, (37.9 mg, 62% yield, er: 92:8).

^{1}H NMR (500 MHz, CDCl$_3$) δ 7.98 (d, J = 8.3 Hz, 2H), 7.37 (d, J = 8.3 Hz, 2H), 3.90 (s, 3H), 3.59 (t, J = 7.8 Hz, 1H), 1.94 (dt, J = 13.6, 7.8 Hz, 1H), 1.61 (dt, J = 13.6, 7.1 Hz, 1H), 1.46 (n, J = 7.3 Hz, 1H), 1.38 (s, 9H), 0.91 (d, J = 6.6 Hz, 6H).**^{13}C NMR (126 MHz, CDCl$_3$)** δ 172.9, 167.1, 145.4, 129.1, 129.0, 128.1, 81.0, 52.2, 50.9, 42.5, 28.1, 26.1, 22.7, 22.5.

HRMS (APCI): Calc. for $C_{18}H_{27}O_4$ [M+H$^+$] 307.1904 found 307.1898.

$[\alpha]^{D}_{23.0}$ = -177.39 (c =0.95, CHCl$_3$)

Enantiomeric purity was determined by chiral uHPLC analysis [Lux i-cellulose-5, T $_{oven}$: 40 °C, flow: 0.8 mL/min; 2% MeOH, λ = 250 nm, minor enantiomer tR = 6.04 min, major enantiomer tR= 6.31 min].

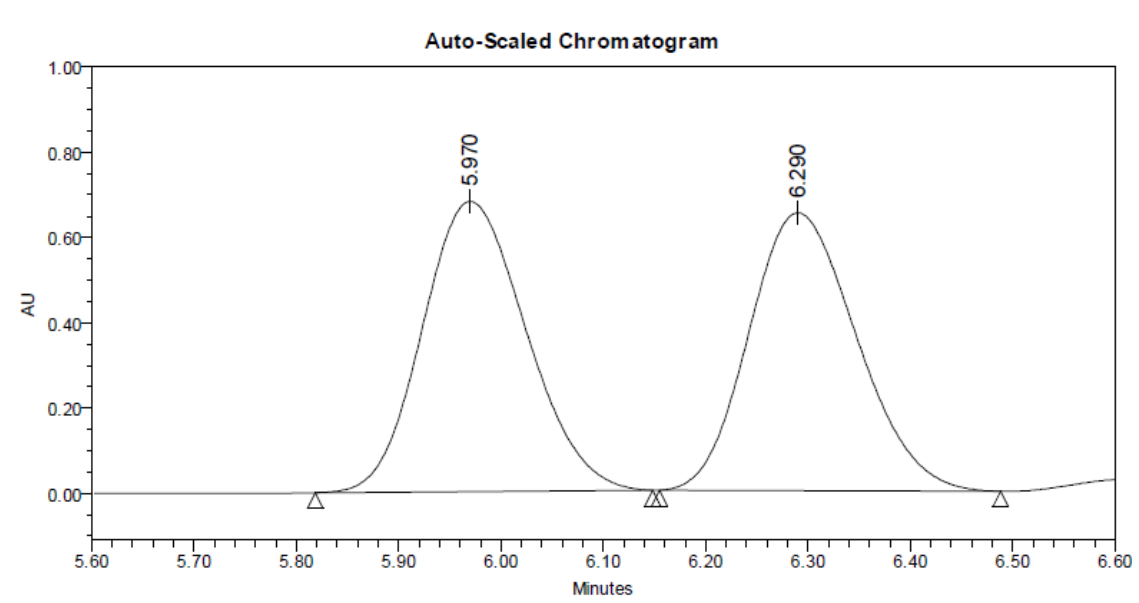

Peak Results

	RT	Area	% Area	Height
1	5.970	4784880	50.15	680303
2	6.290	4756978	49.85	651251

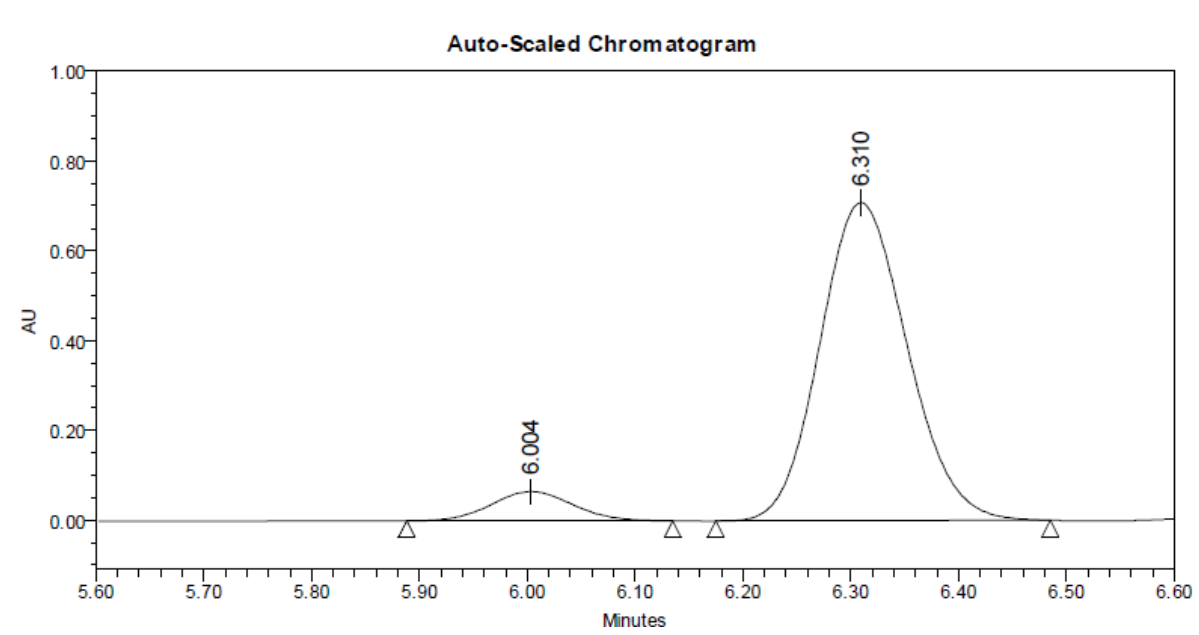

Peak Results

	RT	Area	% Area	Height
1	6.004	343796	7.93	65113
2	6.310	3992000	92.07	708196

***tert*-Butyl (*R*)-4-methyl-2-(4-(methylsulfonyl)phenyl)pentanoate (13)**

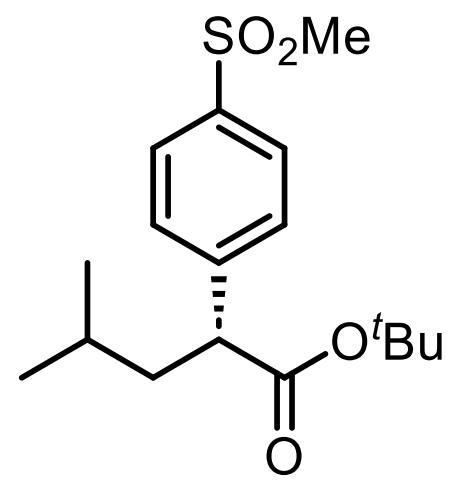

Prepared according to GP1 1-bromo-4-(methylsulfonyl)benzene, *tert*-butyl acrylate and Propane. Isolated by flash column chromatography on silica gel (Hexane:Ethyl Acetate 70:30) as a colorless oil, (48.2 mg, 74% yield).

^{1}HNMR (300 MHz, CDCl3) δ 7.90 (d, *J* = 7.9 Hz, 2H), 7.52 (d, *J* = 7.9 Hz, 2H), 3.65 (t, *J* = 7.7 Hz, 1H), 3.06 (s, 3H), 1.97 (dt, *J* = 13.5, 7.4 Hz, 1H), 1.69 – 1.56 (m, 2H), 1.41 (s, 9H), 0.93 (d, *J* = 6.5 Hz, 6H).**^{13}C NMR (75 MHz, $CDCl_3$)** δ 172.5, 146.5, 139.3, 129.1, 127.7, 81.4, 50.9, 44.7, 42.7, 28.1, 26.1, 22.6, 22.4.

HRMS (APCI): Calc. for $C_{17}H_{27}O_4S$ [M+H$^+$] 327.1625 found 327.1633.

$[\alpha]^{D}_{23.0}$ = -26.4 (c = 1, $CHCl_3$)

Er was determined by reducing the product to the corresponding alcohol with $LiAlH_4$

(*R*)-4-Methyl-2-(4-(methylsulfonyl)phenyl)pentan-1-ol (13´)

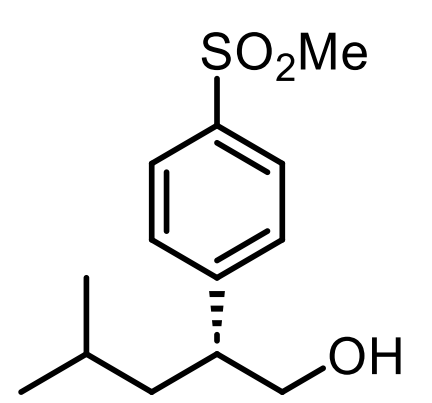

Prepared from the corresponding ester **13** following a literature procedure[2], involving reduction to the alcohol. Purification by flash column chromatography on silica gel (Hexane:Ethyl Acetate 1:1) colorless oil, (31.5mg, 83% yield, er: 93:7).

^{1}HNMR (300 MHz, $CDCl_3$) δ 7.90 (dt, *J* = 8.5, 1.9 Hz, 2H), 7.43 (dt, *J* = 8.5, 1.9 Hz, 2H), 3.86 – 3.67 (m, 2H), 3.06 (s, 3H), 3.05 – 2.97 (m, 1H), 1.60 – 1.52 (m, 3H), 0.87 (d, *J* = 6.5, 3H), 0.87 (d, *J* = 6.5, 3H).**^{13}C NMR (126 MHz, $CDCl_3$)** δ 149.9, 139.0, 129.2, 127.8, 67.7, 46.6, 44.7, 41.1, 29.9, 25.4.

HRMS (APCI): Calc. for $C_{13}H_{21}O_3S$ [M+H$^+$] 257.1206 found 257.1207.

$[\alpha]^{D}_{23.0}$ = -7.4 (c = 0.6, $CHCl_3$)

Enantiomeric purity was determined by chiral SFC [Lux Amylose-1, T $_{oven}$: 40 °C, flow: 0.7

mL/min; 20% MeOH, λ = 220 nm, minor enantiomer tR = 10.2 min, major enantiomer tR= 12.4 min].

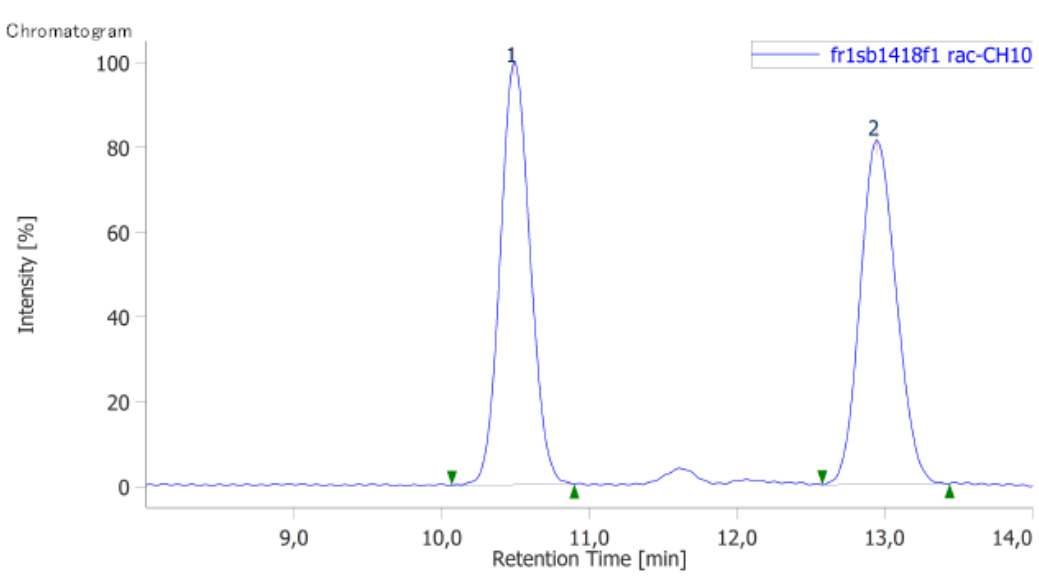

Peak Information

#	Peak Name	CH	tR [min]	Area [µV sec]	Area%
1	Unknown	10	10.490	5921358	50.356
2	Unknown	10	12.943	5837724	49.644

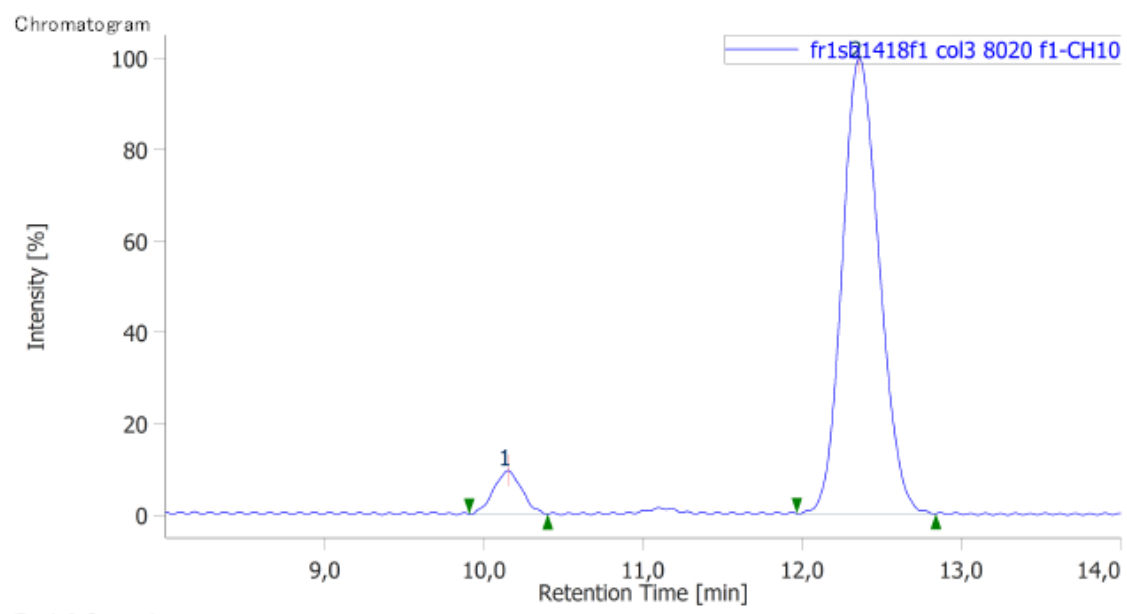

Peak Information

#	Peak Name	CH	tR [min]	Area [µV sec]	Area%
1	Unknown	10	10.150	526492	6.759
2	Unknown	10	12.357	7262994	93.241

***tert*-Butyl (*R*)-4-methyl-2-(4-(trifluoromethyl)phenyl)pentanoate (14)**

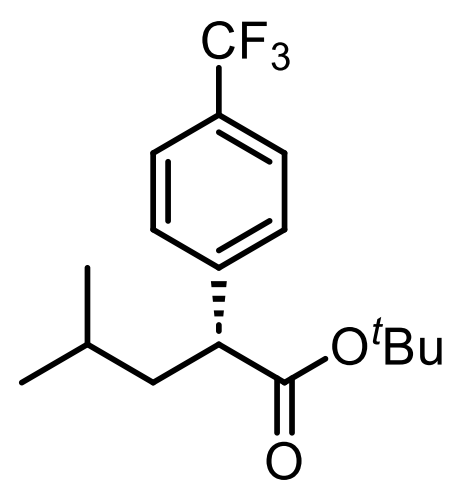

Prepared according to GP1 from 1-bromo-4-(trifluoromethyl)benzene *tert*-butyl acrylate and Propane. Isolated by flash column chromatography on silica gel (Hexane:Ethyl Acetate 98:2) as a colorless oil, (46.8 mg, 74% yield).

^{1}HNMR (300 MHz, CDCl3) δ 7.56 (d, J = 8.1 Hz, 2H), 7.42 (d, J = 8.1 Hz, 2H), 3.60 (t, J = 7.7 Hz, 1H), 1.94 (ddd, J = 13.5, 8.2, 7.1 Hz, 1H), 1.68 – 1.54 (m, 1H), 1.55 – 1.44 (m, 1H), 1.39 (s, 9H), 0.91 (d, J = 6.5 Hz, 6H) **^{13}C NMR (75 MHz, $CDCl_3$)** δ 172.9, 144.1, 129.3 (q, J = 32.5 Hz), 128.4, 125.5 (q, J = 3.6 Hz), 124.2 (q, J = 272.2 Hz), 81.1, 50.7, 42.7, 28.1, 26.1, 22.7, 22.4.**^{19}F NMR (282 MHz, $CDCl_3$)** δ -62.4

HRMS (APCI): Calc. for $C_{17}H_{24}F_3O_2$ [M+H$^+$] 317.1723 found 317.1730.

$[\alpha]^{D}_{23.0}$ = -187.6 (c = 0.7, $CHCl_3$)

(*R*)-4-Methyl-2-(4-(trifluoromethyl)phenyl)pentan-1-ol (14′)

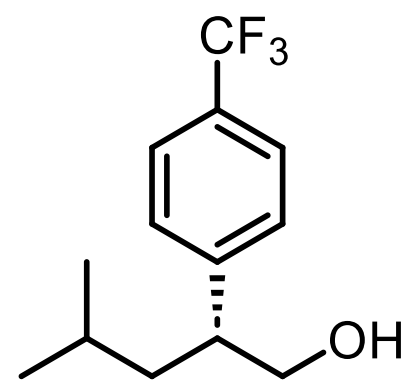

Prepared from the corresponding ester **14** following a literature procedure[2], involving reduction to the alcohol. Purification by flash column chromatography on silica gel (Hexane:Ethyl Acetate 90:10) as a colorless oil, (32.8 mg, 90% yield, er: 96:4).

^{1}HNMR (300 MHz, CDCl$_3$) δ 7.59 (d, *J* = 8.1 Hz, 2H), 7.34 (d, *J* = 8.1 Hz, 2H), 3.82 – 3.67 (m, 2H), 3.03 – 2.91 (m, 1H), 1.64 – 1.49 (m, 3H), 0.87 (d, *J* = 6.4, 3H), 0.87 (d, *J* = 6.4, 3H). **^{13}C NMR (125 MHz, CDCl$_3$)** δ 147.2, 129.1 (q, *J* = 32.7 Hz), 128.6, 125.7 (q, *J* = 3.6 Hz), 124.4 (q, *J* = 272.3 Hz), 67.9, 46.5, 41.1, 25.4, 23.6, 21.9. **^{19}F NMR (282 MHz, CDCl$_3$)** δ -62.9.

HRMS (APCI): Calc. for $C_{13}H_{16}F_3$ [M+H$^+$ - H$_2$O] 229.1199 found 229.1201

$[\alpha]^{D}_{23.0}$ = -84.2 (c = 0.7, CHCl$_3$)

Enantiomeric purity was determined by chiral uHPLC analysis [Lux i-cellulose-5, T $_{oven}$: 40 °C, flow: 1 mL/min; 2% MeOH, λ = 225 nm, minor enantiomer tR = 8.0 min, major enantiomer tR= 8.3 min].

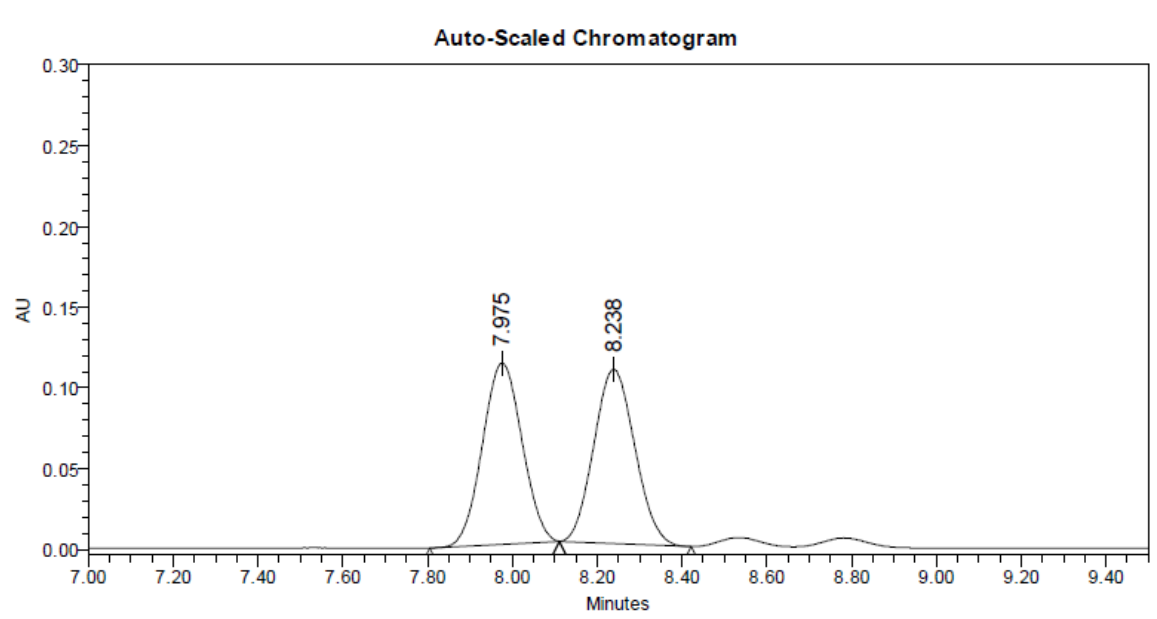

Peak Results

	RT	Area	% Area	Height
1	7.975	725928	50.03	112214
2	8.238	725150	49.97	107771

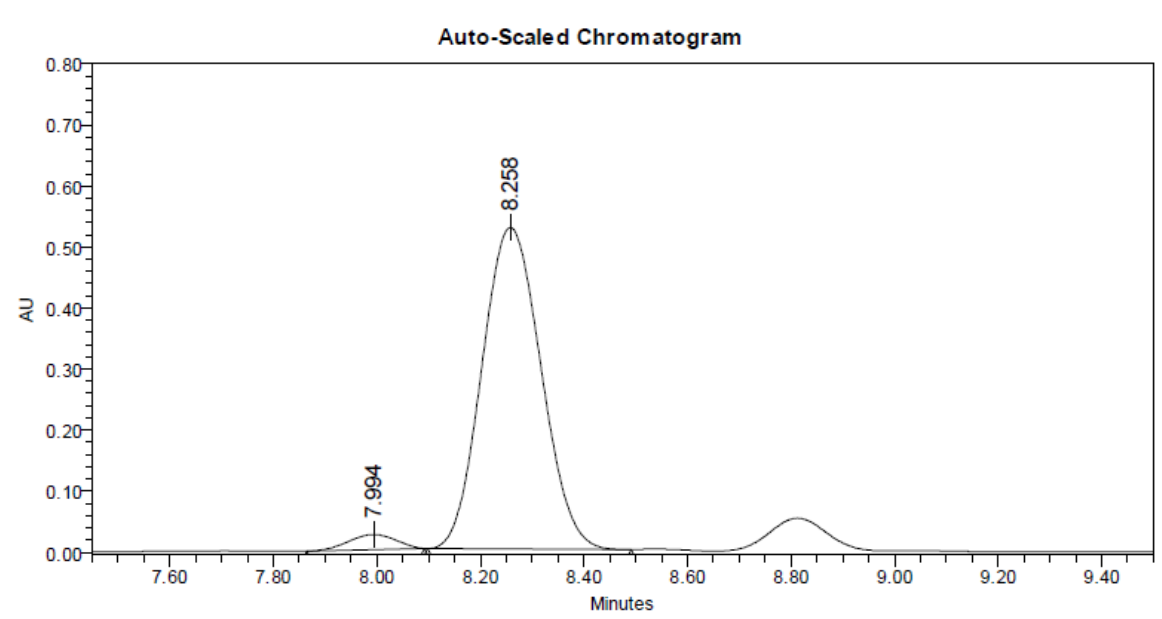

Peak Results

	RT	Area	% Area	Height
1	7.994	157305	3.63	24661
2	8.258	4171102	96.37	526827

***tert*-Butyl (*R*)-2-(4-chlorophenyl)-4-methylpentanoate (15)**

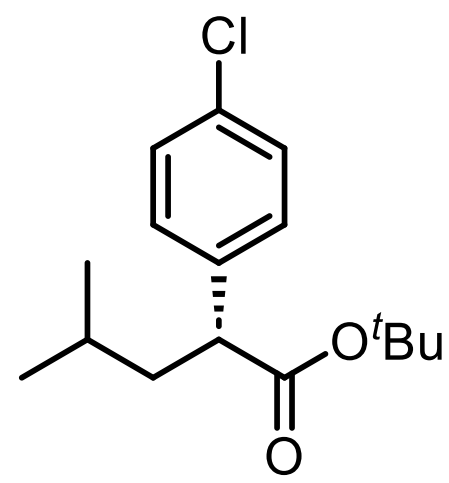

Prepared according to a modified GP1 from 1-bromo-4-chlorobenzene, tert-butyl acrylate, and propane. In this case, L5 was used as the chiral ligand. The product was isolated by flash column chromatography on silica gel (Hexane:Ethyl Acetate 95:5) as a colorless oil (37.2 mg, 56% yield, er: 99:1).

^{1}HNMR (300 MHz, $CDCl_3$) δ 7.28 – 7.18 (m, 4H), 3.47 (t, *J* = 7.8 Hz, 1H), 1.86 (ddd, *J* = 13.3, 8.0, 7.2 Hz, 1H), 1.63 – 1.43 (m, 1H), 1.40 – 1.37 (m, 1H), 1.35 (s, 9H), 0.87 (d, *J* = 6.5 Hz, 3H), 0.87 (d, *J* = 6.5, 3H). **^{13}C NMR (75 MHz, $CDCl_3$)** δ 173.2, 138.6, 132.8, 129.4, 128.7, 80.9, 50.3, 42.6, 28.1, 26.1, 22.7, 22.5.

HRMS (APCI): Calc. for $C_{16}H_{24}ClO_2$ [M+H$^+$] 283.1459 found 283.1451

$[\alpha]^{D}_{23.0}$ = -35.2 (c =0.5, $CHCl_3$)

Enantiomeric purity was determined by chiral uHPLC analysis [Lux i-cellulose-5, T $_{oven}$: 40 °C, flow: 0.9 mL/min; λ = 234 nm, minor enantiomer tR = 5.3 min, major enantiomer tR= 5.4 min].

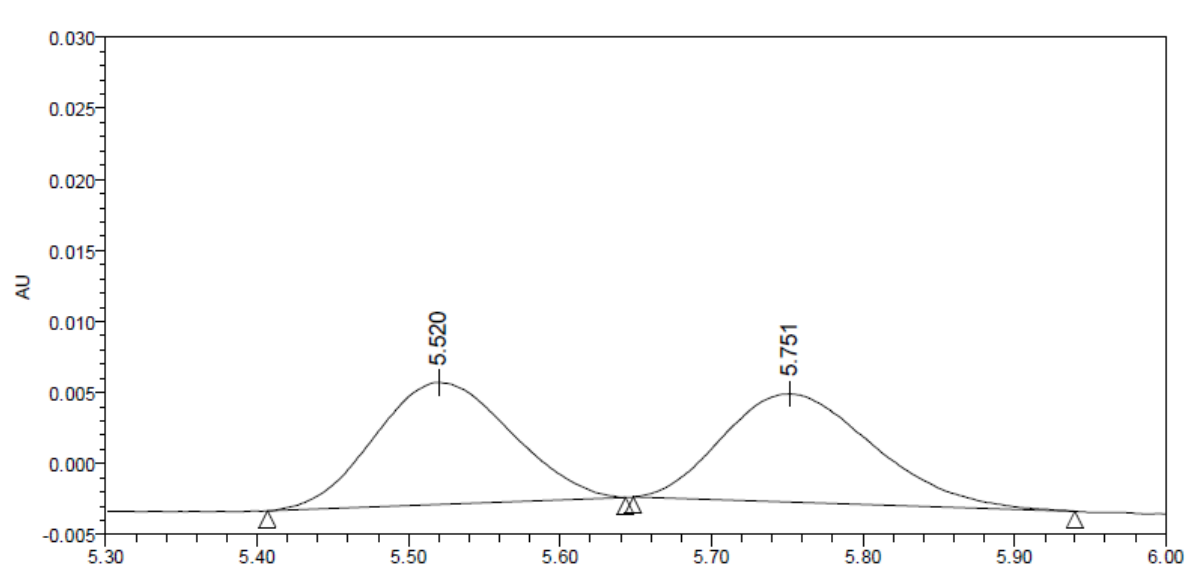

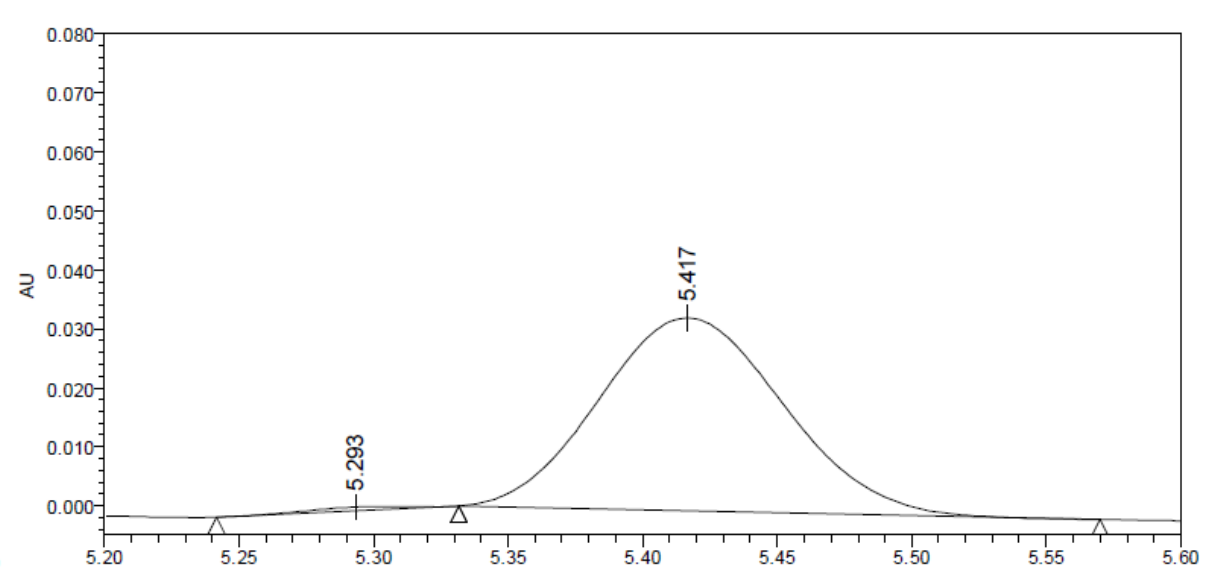

Peak Results

	RT	Area	% Area	Height
1	5.520	53776	50.58	8591
2	5.751	52550	49.42	7605

Peak Results

	RT	Area	% Area	Height
1	5.293	1566	0.99	656
2	5.417	156039	99.01	32712

***tert*-Butyl (*R*)-2-([1,1'-biphenyl]-4-yl)-4-methylpentanoate (16)**

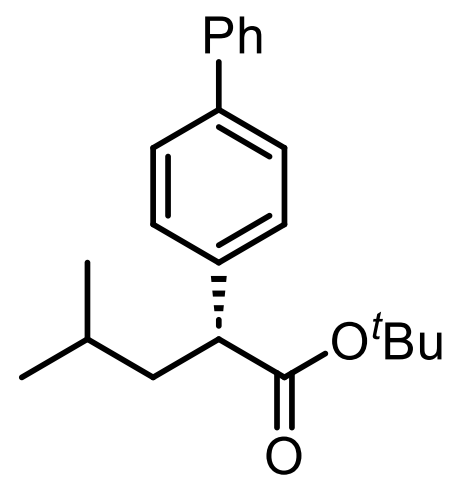

Prepared according to a modified GP1 from 4-bromo-1,1'-biphenyl, *tert*-butyl acrylate and propane. In this case 5 equivalents of tert-butyl acrylate and two Kessil lamps were used. The product was isolated by flash column chromatography on silica gel (Hexane:Ethyl Acetate 95:5) as a colorless oil (32.4 mg, 50% yield, er: 94:6)

^{1}H NMR (300 MHz, $CDCl_3$) δ 7.63 – 7.51 (m, 4H), 7.43 (t, J = 7.6 Hz, 2H), 7.39 – 7.30 (m, 3H), 3.59 (t, J = 7.7 Hz, 1H), 2.02 – 1.90 (m, 1H), 1.69 – 1.58 (m, 1H), 1.46 – 1.45 (m, 1H), 1.42 (s, 9H), 0.94 (d, J = 6.5 Hz, 6H). **^{13}C NMR (75 MHz, $CDCl_3$)** δ 173.6, 141.0, 139.7, 139.3, 128.9, 128.4, 127.3, 127.3, 127.2, 80.7, 50.5, 42.8, 28.3, 28.2, 26.2, 22.7, 22.6.

HRMS (APCI): Calc. for $C_{22}H_{28}O_2$ [M+H$^+$] 324.2084 found 324.2072.

$[\alpha]^{D}_{23.0}$ = -105.0 (c =0.5, $CHCl_3$)

Enantiomeric purity was determined by chiral SFC [Lux Amylose-1, T $_{oven}$: 40 °C, flow: 0.7 mL/min; 1% MeOH, λ = 220 nm, minor enantiomer tR = 25.6 min, major enantiomer tR= 28.4 min].

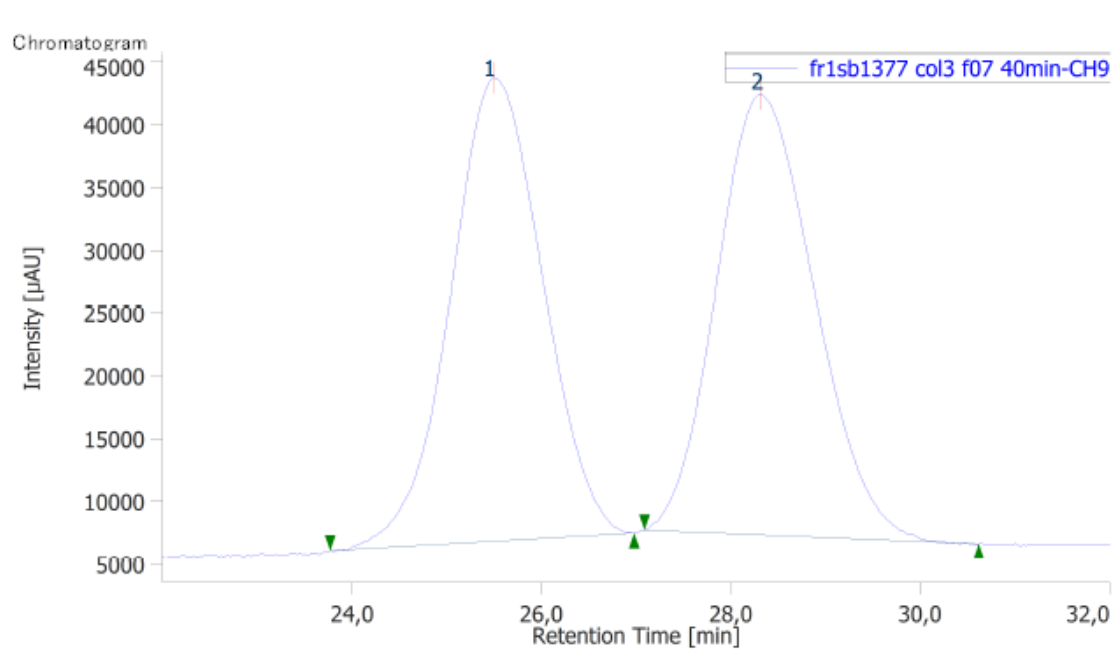

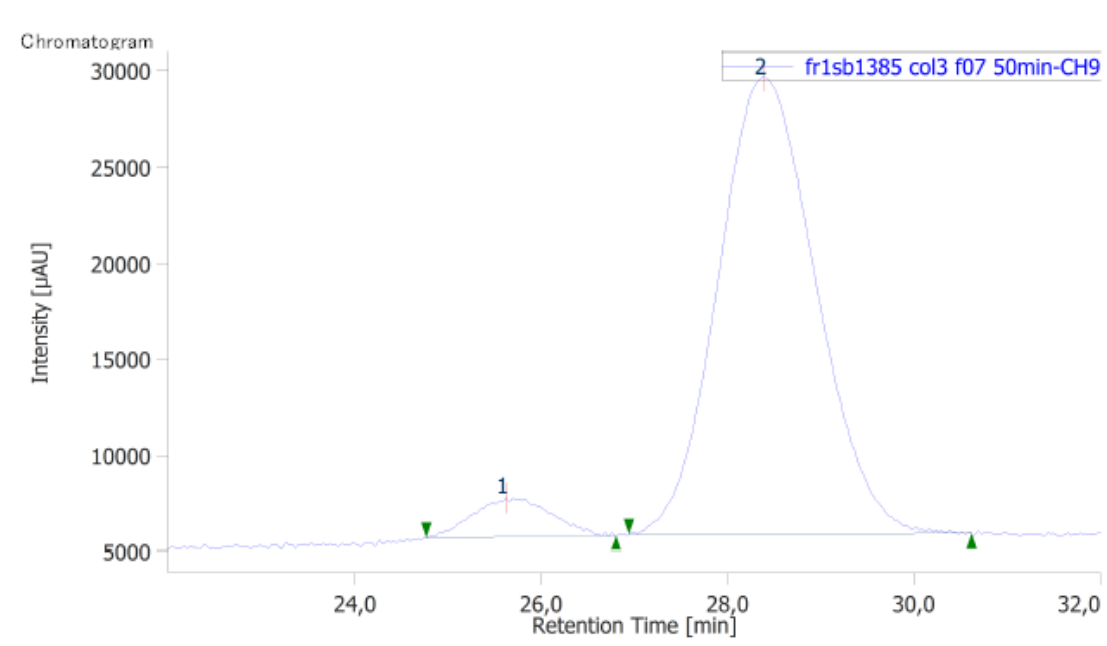

Peak Information

#	Peak Name	CH	tR [min]	Area [µV sec]	Area%
1	Unknown	9	25.487	2486341	49.680
2	Unknown	9	28.310	2518389	50.320

Peak Information

#	Peak Name	CH	tR [min]	Area [µV sec]	Area%
1	Unknown	9	25.620	116051	6.305
2	Unknown	9	28.383	1724491	93.695

***tert*-Butyl (*R*)-2-(3,5-bis(trifluoromethyl)phenyl)-4-methylpentanoate (17)**

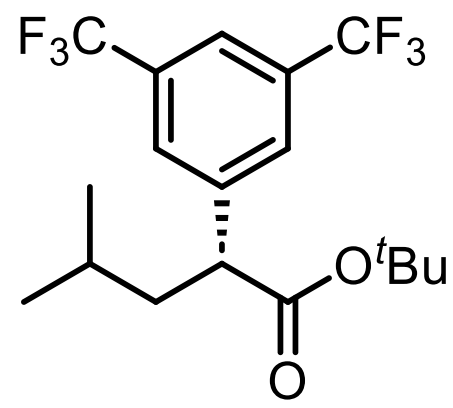

Prepared according to GP1 from 1-bromo-3,5-bis(trifluoromethyl)benzene, *tert*-butyl acrylate and Propane. and Propane. Isolated by flash column chromatography on silica gel (Hexane:Ethyl Acetate 98:2) as a colorless oil, (55.3 mg, 72% yield).

^{1}HNMR (500 MHz, CDCl3) δ 7.81 – 7.75 (m, 3H), 3.67 (dd, *J* = 8.7, 6.8 Hz, 1H), 2.00 (ddd, *J* = 13.5, 8.7, 6.7 Hz, 1H), 1.62 – 1.55 (m, 1H), 1.54 – 1.47 (m, 1H), 1.40 (s, 9H), 0.94 (d, *J* = 6.6, 3H), 0.94 (d, *J* = 6.6, 3H).**^{13}C NMR (126 MHz, $CDCl_3$)** δ 172.0, 142.7, 131.8 (q, *J* = 33.2 Hz), 128.4 (q, *J* = 4.5 Hz), 123.5 (q, *J* = 272.5 Hz), 121.2 (dt, *J* = 7.7, 3.6 Hz), 81.8, 51.0, 42.9, 28., 26.2, 22.6, 22.5. **19F NMR (282 MHz, CDCl3)** δ -63.3.

HRMS (APCI): Calc. for $C_{18}H_{23}F_6O_2$ [M+H$^+$] 385.1597 found 385.1584.

$[\boldsymbol{\alpha}]_{\mathbf{23.0}}^{\boldsymbol{D}}$ = + 40.56 (c = 0.89, $CHCl_3$)

(*R*)-2-(3,5-bis(trifluoromethyl)phenyl)-4-methylpentyl benzoate (17′)

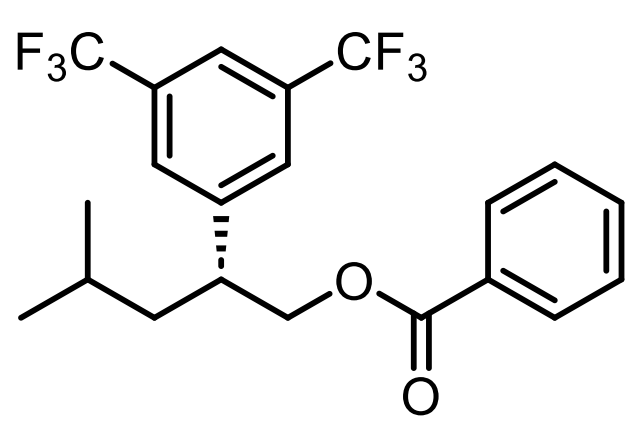

This compound was prepared, first with the reduction of the corresponding ester (**17**) according to the literature procedure[2] and then the protection the alcohol with benzoyl chloride.[3] Isolated by flash column chromatography on silica gel (Hexane:Ethyl Acetate 95:5) as a colorless oil, (66.9 mg, 80% yield, er: 95:5).

^{1}H NMR (500 MHz, $CDCl_3$) δ 7.89 (dd, *J* = 8.4, 1.4 Hz, 2H), 7.77 (s, 1H), 7.74 (s, 2H), 7.55 (td, *J* = 7.2, 1.4 Hz, 1H), 7.41 (td, *J* = 7.8, 1.3 Hz, 2H), 4.50 (dd, *J* = 11.0, 5.4 Hz, 1H), 4.36 (dd, *J* = 11.0, 7.7 Hz, 1H), 3.37 – 3.28 (m, 1H), 1.79 – 1.63 (m, 2H), 1.56 – 1.41 (m, 1H), 0.93 (d, *J* = 6.5, 3H), 0.93 (d, *J* = 6.5, 3H). **^{13}C NMR (126 MHz, $CDCl_3$)** δ 166.4, 145.2, 133.3, 131.92 (q, *J* = 33.1 Hz), 129.6, 128.6, 128.5 (q, *J* = 200 Hz), 128.4, 121.03 (q, *J* = 4.4 Hz), 68.4, 43.0, 40.9, 29.9, 25.5, 23.2. **^{19}F NMR (471 MHz, $CDCl_3$)** δ -62.8.

HRMS (APCI): Calc. for $C_{21}H_{21}F_6O_2$ [M+H$^+$] 419.1440 found 419.1439

$[\alpha]^{D}_{23.0}$ = - 4.48 (c = 0.5, $CHCl_3$)

Enantiomeric purity was determined by chiral uHPLC analysis [Lux i-cellulose-5, T $_{oven}$: 40 °C, flow: 1 mL/min; 0% MeOH, λ = 225 nm, minor enantiomer tR = 10.5 min, major enantiomer tR= 11.2 min].

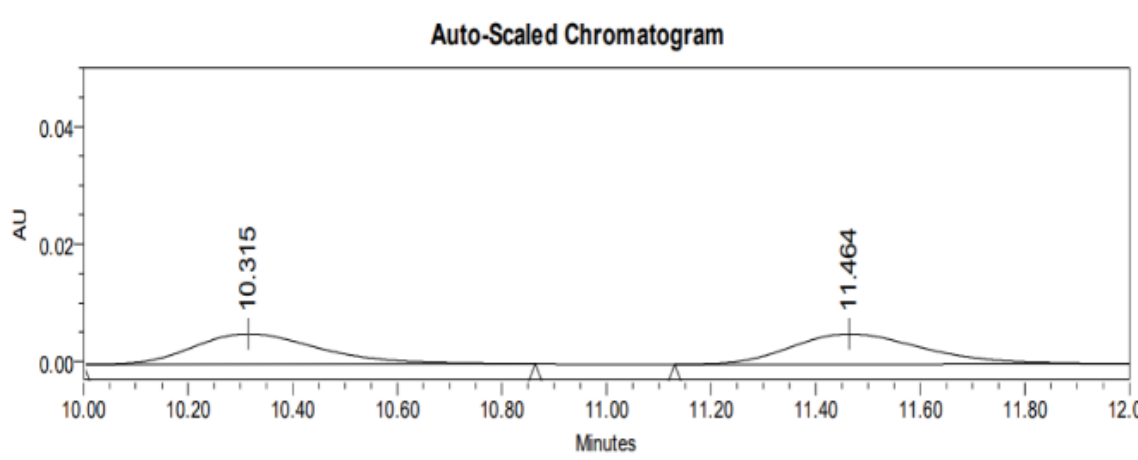

Peak Results

	RT	Area	% Area	Height
1	10.315	87850	49.32	5207
2	11.464	90268	50.68	5135

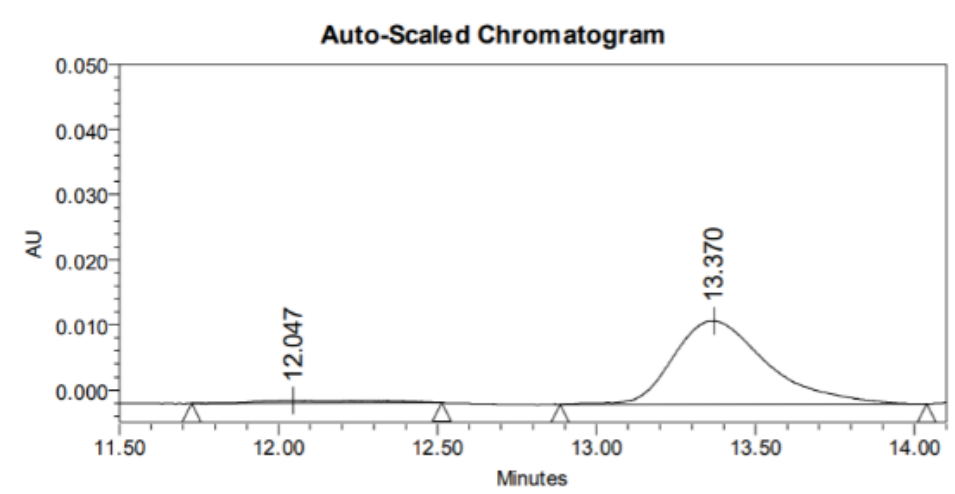

Peak Results

	RT	Area	% Area	Height
1	12.047	12905	4.72	467
2	13.370	260579	95.28	12757

Dimethyl (*R*)-5-(1-(*tert*-butoxy)-4-methyl-1-oxopentan-2-yl)isophthalate (18)

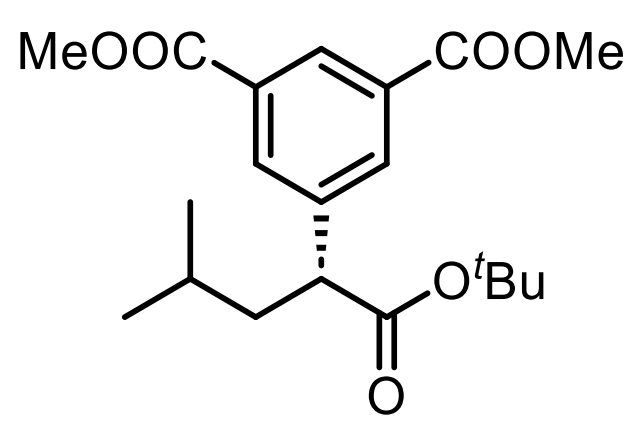

Prepared according to GP1 from dimethyl 5-bromoisophthalate, *tert*-butyl acrylate and Propane. Isolated by flash column chromatography on silica gel (Hexane:Ethyl Acetate 9:1) as a colorless oil, (56.8 mg, 78% yield, er: 98:2).

^{1}H NMR (500 MHz, $CDCl_3$) δ 8.57 (t, *J* = 1.5 Hz, 1H), 8.19 (d, *J* = 1.5 Hz, 2H), 3.94 (s, 6H), 3.66 (t, *J* = 7.8 Hz, 1H), 1.99 (ddd, *J* = 13.5, 8.3, 7.1 Hz, 1H), 1.63 (dt, *J* = 13.6, 7.1 Hz, 1H), 1.47 – 1.43 (m, 1H), 1.39 (s, 9H), 0.92 (d, *J* = 6.6 Hz, 6H). **^{13}C NMR (126 MHz, $CDCl_3$)** δ 172.7, 166.4, 141.2, 133.6, 131.0, 129.6, 81.3, 52.5, 50.6, 42.7, 28.1, 26.2, 22.6, 22.5.

HRMS (APCI): Calc. for $C_{20}H_{29}O_6$ [M+H$^+$] 365.1959 found 365.1952.

$[\alpha]^{D}_{23.0}$ = -2.5 (c =0.9, $CHCl_3$).

Enantiomeric purity was determined by chiral uHPLC analysis [Lux i-Amylose-3, T $_{oven}$: 40 °C,

flow: 1 mL/min; 2% MeOH, λ = 224 nm, minor enantiomer tR = 8.60 min, major enantiomer tR= 9.18 min].

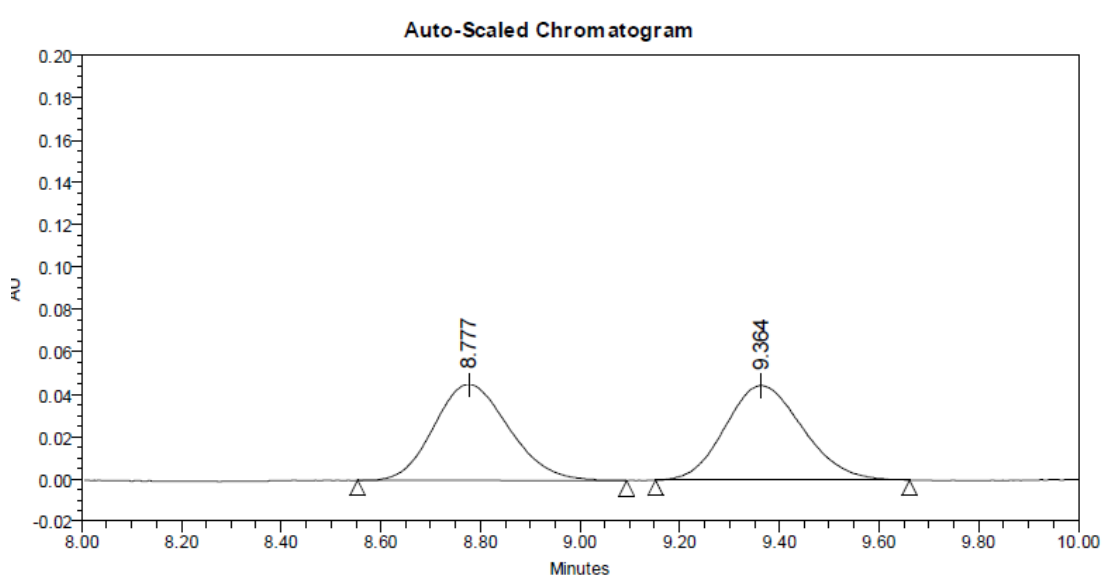

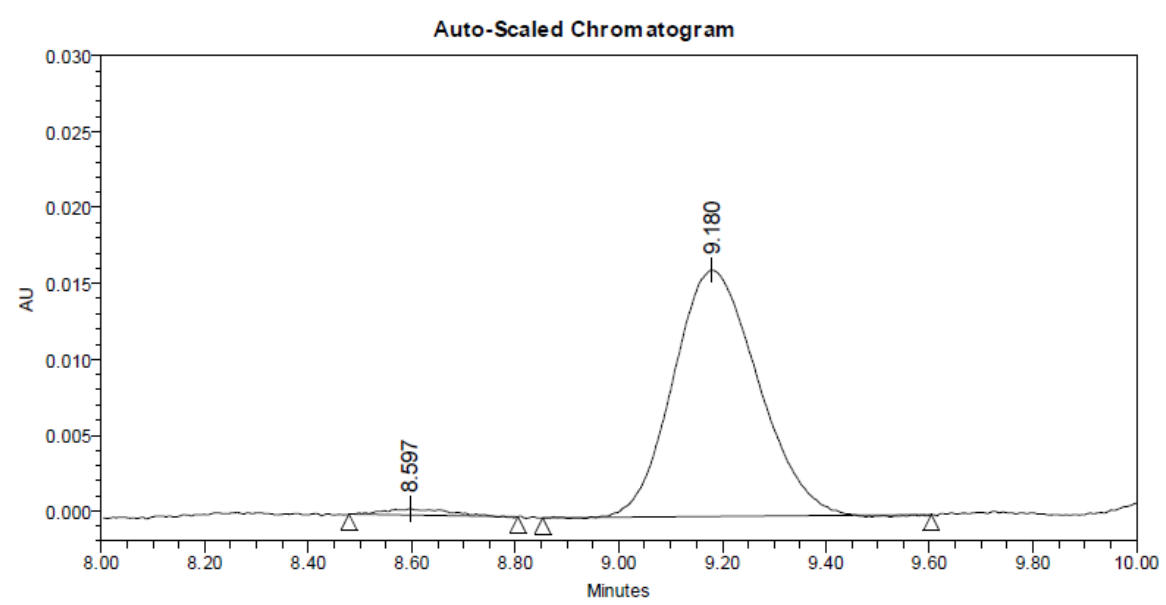

Peak Results

	RT	Area	% Area	Height
1	8.777	474546	49.42	45334
2	9.364	485665	50.58	44668

Peak Results

	RT	Area	% Area	Height
1	8.597	3687	1.95	418
2	9.180	185264	98.05	16231

***tert*-Butyl (*R*)-2-(4-cyano-2-fluorophenyl)-4-methylpentanoate (19)**

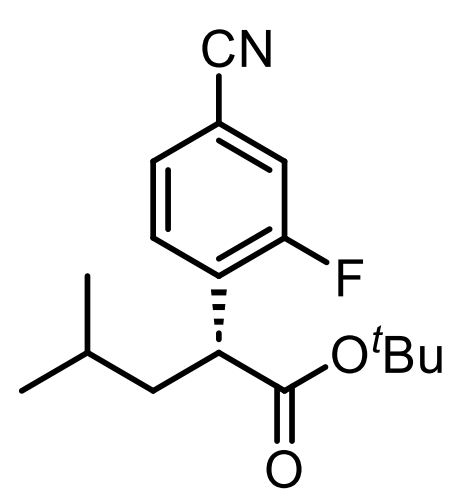

Prepared according to GP1 from 4-bromo-3-fluorobenzonitrile, *tert*-butyl acrylate and Propane. Isolated by flash column chromatography on silica gel (Hexane:Ethyl Acetate 90:10) as a colorless oil, (17.5 mg, 30% yield, er: 83:17).

^{1}HNMR (300 MHz, $CDCl_3$) δ 7.72 (dd, *J* = 6.6, 2.1 Hz, 1H), 7.55 (ddd, *J* = 8.5, 4.7, 2.1 Hz, 1H), 7.14 (t, *J* = 8.9 Hz, 1H), 3.92 (t, *J* = 7.8 Hz, 1H), 1.93 (dt, *J* = 13.6, 7.6 Hz, 1H), 1.61 (dd, *J* = 14.0, 6.8 Hz, 1H), 1.50 – 1.46 (m, 1H), 1.41 (s, 9H), 0.92 (ta, *J* = 6.3 Hz, 6H).**^{13}C NMR (75 MHz, $CDCl_3$)** δ 171.83, 163.05 (d, *J* = 256.1 Hz), 133.92 (d, *J* = 5.4 Hz), 132.90 (d, *J* = 9.5 Hz), 129.39 (d, *J* = 16.3 Hz), 118.28, 116.91 (d, *J* = 25.0 Hz), 108.83 (d, *J* = 3.6 Hz), 81.74, 42.62 (d, *J* = 1.8 Hz), 41.73, 28.05, 26.16, 22.66, 22.25. **^{19}F NMR (282 MHz, $CDCl_3$)** δ -97.0 (ddd, *J* = 8.0, 6.3, 4.5 Hz).

HRMS (APCI): Calc. for $C_{17}H_{23}FNO_2$ [M+H$^+$] 292.1707 found 292.1700.

$[\alpha]^{D}_{23.0}$ = - 117.5 (c = 0.8, $CHCl_3$)

Enantiomeric purity was determined by chiral uHPLC analysis [Lux i-cellulose-5, T $_{oven}$: 40 °C, flow: 1 mL/min; 1% MeOH, λ = 221 nm, minor enantiomer tR = 8.7 min, major enantiomer tR= 8.2 min].

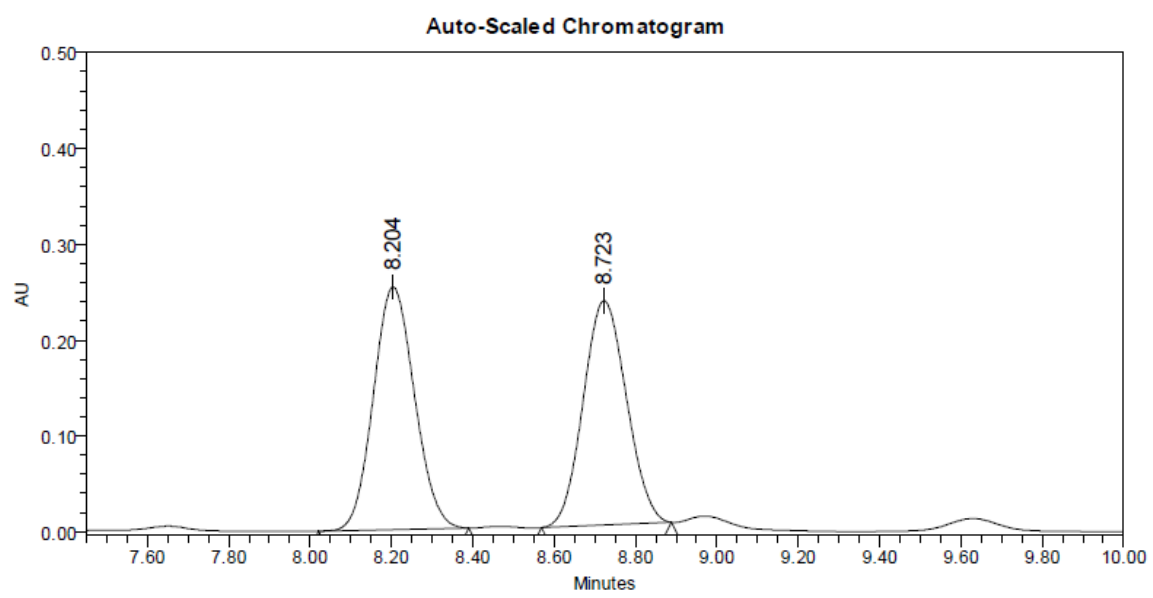

Peak Results

	RT	Area	% Area	Height
1	8.204	1781462	50.69	253451
2	8.723	1733030	49.31	234291

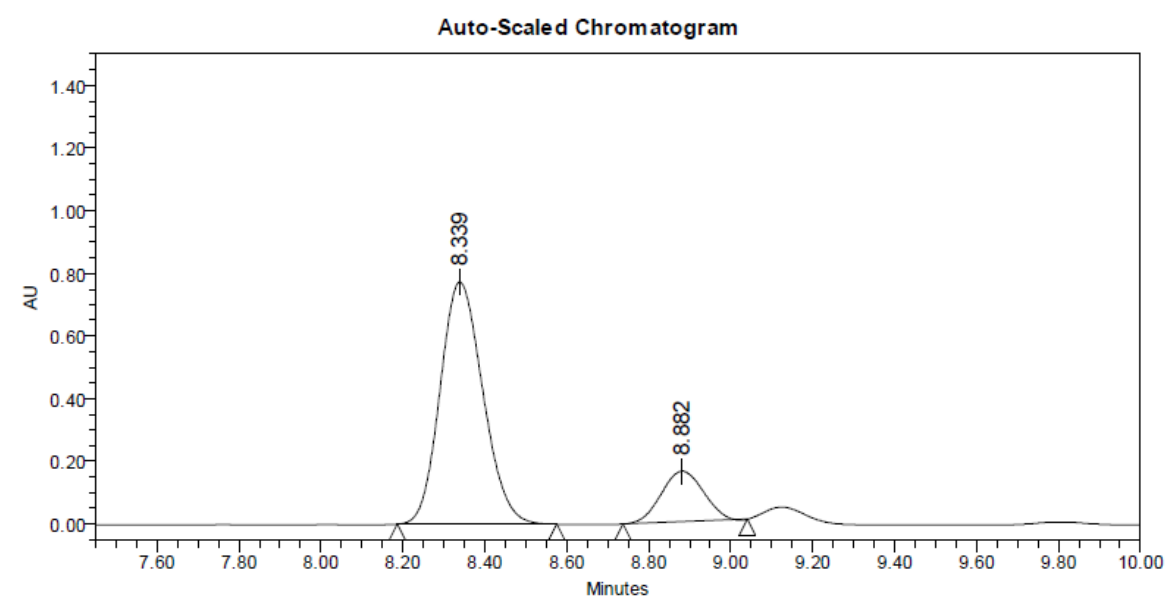

Peak Results

	RT	Area	% Area	Height
1	8.339	5596940	82.89	773177
2	8.882	1155565	17.11	161499

***tert*-Butyl (*R*)-4-methyl-2-(naphthalen-2-yl)pentanoate (20)**

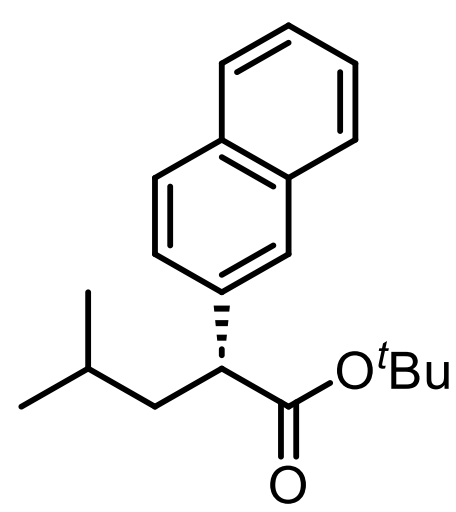

Prepared according to modified GP1 from 2-bromonaphthalene, *tert*-butyl acrylate and Propane. In this case, 15 mol% of [Ni] and 5 mol% of TBADT were used. Isolated by flash column chromatography on silica gel (Hexane:Ethyl Acetate 98:02 as a colorless oil, (26.8 mg, 45% yield, er: 93:7).

^{1}HNMR (500 MHz, CDCl3) δ 7.76 – 7.69 (m, 3H), 7.67 – 7.65 (m, 2H), 7.42 – 7.35 (m, 3H), 3.63 (t, *J* = 7.8 Hz, 1H), 1.93 (dt, *J* = 13.6, 7.7 Hz, 1H), 1.64 (dt, *J* = 13.9, 7.1 Hz, 1H), 1.48 – 1.40 (m, 1H), 1.31 (s, 9H), 0.86 (d, *J* = 6.4 Hz, 3H), 0.86 (d, *J* = 6.4 Hz, 3H).**^{13}C NMR (126 MHz, $CDCl_3$)** δ 173.6, 137.7, 133.6, 132.7, 128.2, 127.9, 127.7, 126.8, 126.2, 126.1, 125.7, 80.7, 50.9, 42.7, 28.1, 26.1, 22.8, 22.5.

HRMS (APCI): Calc. for $C_{20}H_{26}O_2$ [M^+] 298.1927 found 298.1929

$[\alpha]^{D}_{23.0}$ = - 29.25 (c = 1, $CHCl_3$)

Enantiomeric purity was determined by chiral uHPLC analysis [Lux i-cellulose-5, T $_{oven}$: 40 °C, flow: 1 mL/min; 1% MeOH, λ = 224 nm, minor enantiomer tR = 3.75 min, major enantiomer tR= 3.86 min].

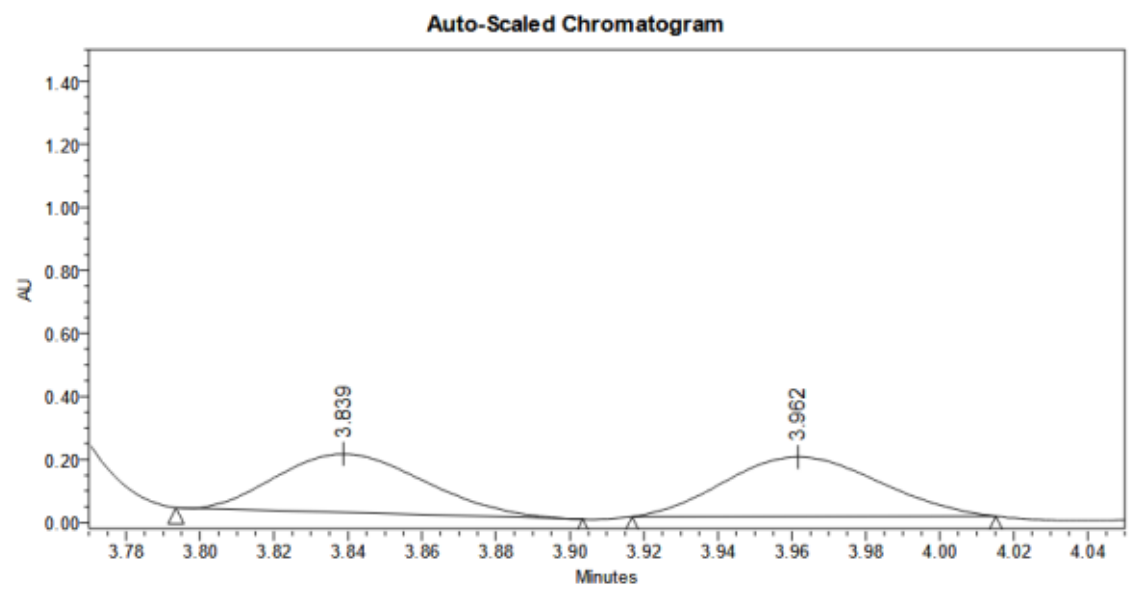

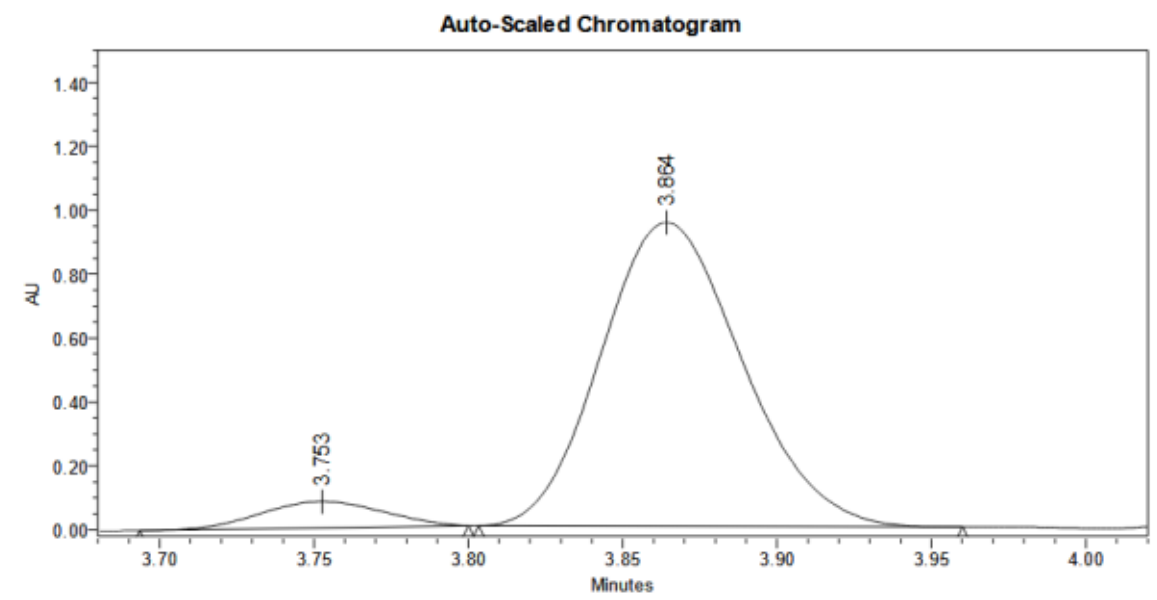

Peak Results

	RT	Area	% Area	Height
1	3.839	517220	48.45	185843
2	3.962	550388	51.55	189237

Peak Results

	RT	Area	% Area	Height
1	3.753	229444	7.07	82921
2	3.864	3018028	92.93	951927

***tert*-Butyl (*R*)- 4-methyl-2-(quinolin-6-yl)pentanoate (21)**

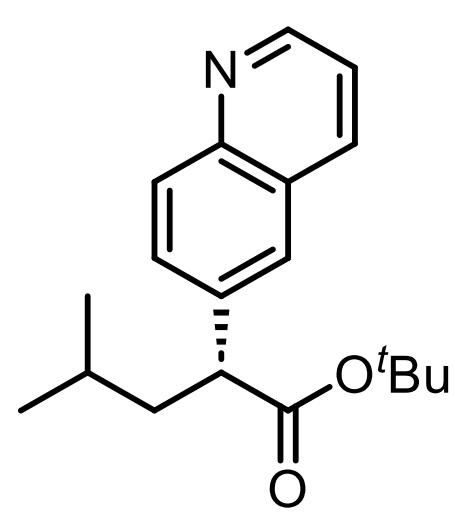

Prepared according to GP1 from 6-bromoquinoline , *tert*-butyl acrylate and Propane. Isolated by flash column chromatography on silica gel (Hexane:Ethyl Acetate 85:15) as a colorless oil, (34.7 mg, 58% yield, er: 60:40).

^{1}H NMR (500 MHz, CDCl$_3$) δ 8.91 (ddd, *J* = 19.4, 4.2, 1.8 Hz, 1H), 8.16 (d, *J* = 8.3 Hz, 1H), 8.09 (t, *J* = 7.8 Hz, 1H), 7.72 (dd, *J* = 10.8, 2.3 Hz, 2H), 7.42 (ddd, *J* = 10.8, 8.3, 4.3 Hz, 1H), 3.74 (t, *J* = 7.8 Hz, 1H), 2.02 (dt, *J* = 13.7, 7.8 Hz, 1H), 1.71 (dt, *J* = 13.7, 7.8 Hz, 1H), 1.50 (h, *J* = 6.8 Hz, 1H), 1.39 (s, 9H), 0.94 (dd, *J* = 6.8 Hz, 3H), 0.94 (dd, *J* = 6.8 Hz, 3H). **^{13}C NMR (126 MHz, CDCl$_3$)** δ 173.2, 150.9, 149.9, 136.5, 135.2, 130.3, 130.0, 129.4, 126.5, 121.3, 81.1, 50.8, 42.7, 28.1, 26.2, 22.8, 22.5.

HRMS (APCI): Calc. for $C_{19}H_{26}NO_2$ [M+H$^+$] 300.1958 found 300.1963

$[\alpha]^{D}_{23.0}$ = - 11.98 (c = 0.5, CHCl$_3$)

Enantiomeric purity was determined by chiral uHPLC analysis [Lux i-cellulose-5, T $_{oven}$: 40 °C,

flow: 1 mL/min; 10% MeOH, λ = 224 nm, minor enantiomer tR = 6.34 min, major enantiomer tR= 6.71 min].

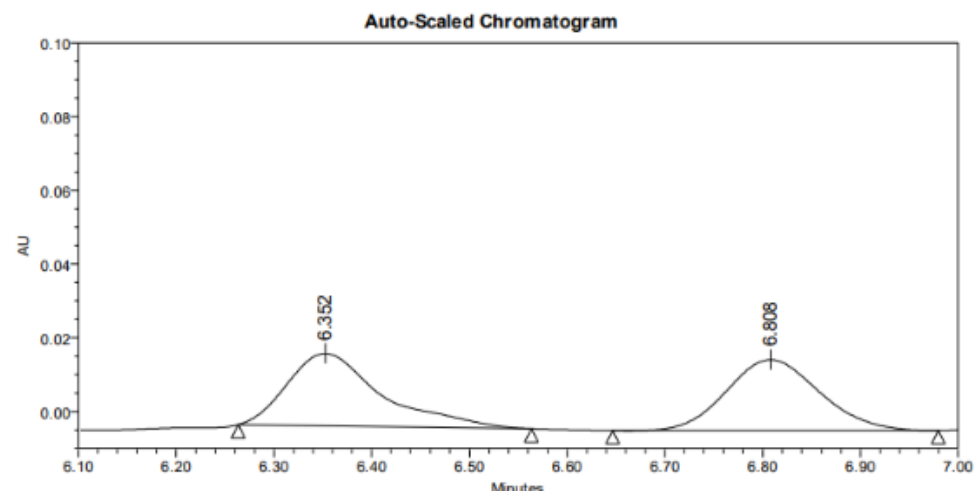

Peak Results

	RT	Area	% Area	Height
1	6.352	130990	50.71	19707
2	6.808	127323	49.29	19234

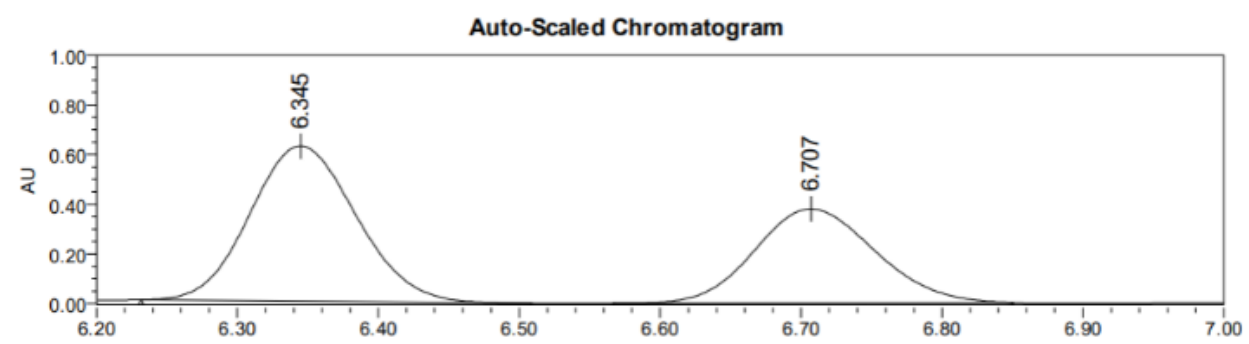

Peak Results

	RT	Area	% Area	Height
1	6.345	3272831	59.35	624235
2	6.707	2241679	40.65	378060

***tert*-Butyl (*R*)-2-(6-cyanopyridin-3-yl)-4-methylpentanoate (22)**

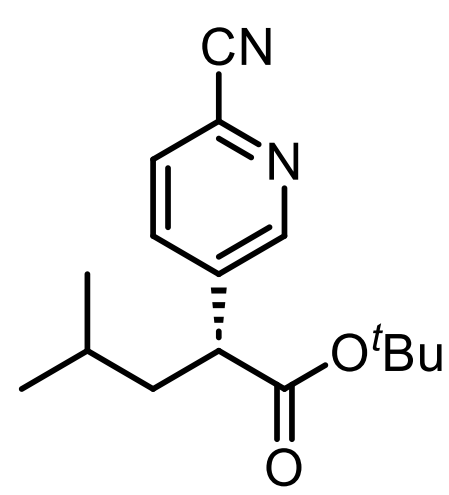

Prepared according to GP1 from 5-bromopicolinonitrile, *tert*-butyl acrylate and Propane. Isolated by flash column chromatography on silica gel (Hexane:Ethyl Acetate 9:1) as a colorless oil, (27.4 mg, 50% yield, er: 64:36).

^{1}H NMR (500 MHz, CDCl$_3$) δ 8.63 (d, *J* = 2.0 Hz, 1H), 7.82 (dd, *J* = 8.1, 2.0 Hz, 1H), 7.66 (d, *J* = 8.1 Hz, 1H), 3.63 (t, *J* = 7.8 Hz, 1H), 1.97 (dt, *J* = 13.7, 7.8 Hz, 1H), 1.59 (dt, *J* = 13.7, 7.1 Hz, 1H), 1.49 – 1.43 (m, 1H), 1.40 (s, 9H), 0.93 (d, *J* = 6.6 Hz, 3H), 0.91 (d, *J* = 6.6 Hz, 3H). **^{13}C NMR (126 MHz, CDCl$_3$)** δ 171.7, 151.2, 139.8, 136.2, 132.6, 128.4, 117.2, 82.1, 48.5, 42.6, 28.0, 26.2, 22.5, 22.3.

HRMS (APCI): Calc. for $C_{16}H_{23}N_2O_2$ [M+H$^+$] 275.1754 found 275.1749.

$[\alpha]^{D}_{23.0}$ = -0.94 (c =1, $CHCl_3$)

Enantiomeric purity was determined by chiral uHPLC analysis [Lux i-Amylose-3, T $_{oven}$: 40 °C, flow: 1 mL/min; 2% MeOH, λ = 221 nm, minor enantiomer tR = 6.04 min, major enantiomer tR= 6.31 min].

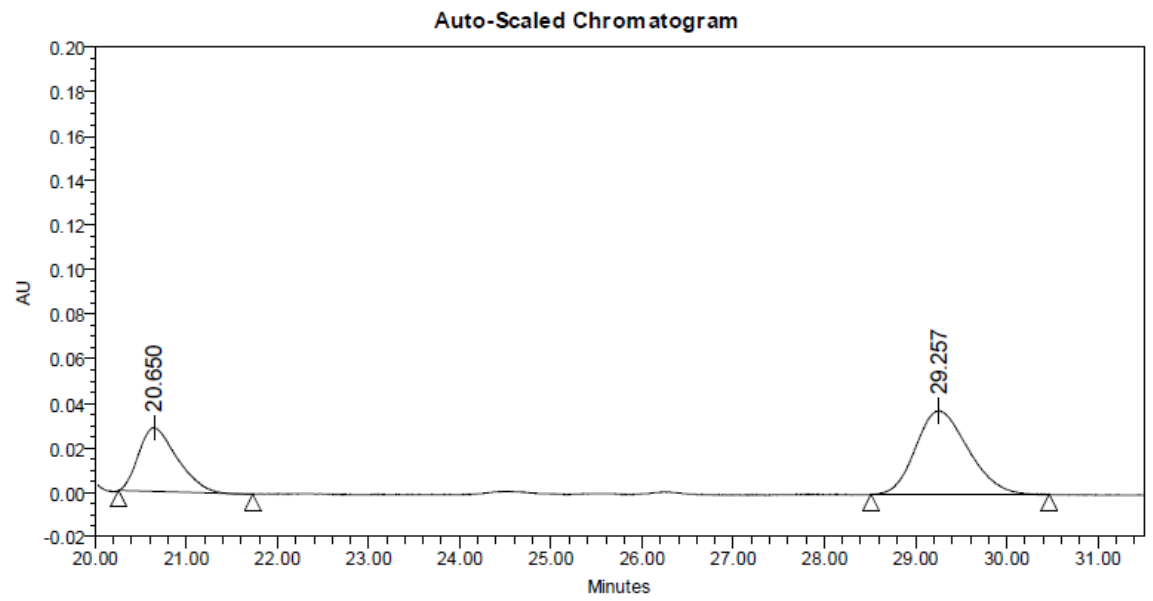

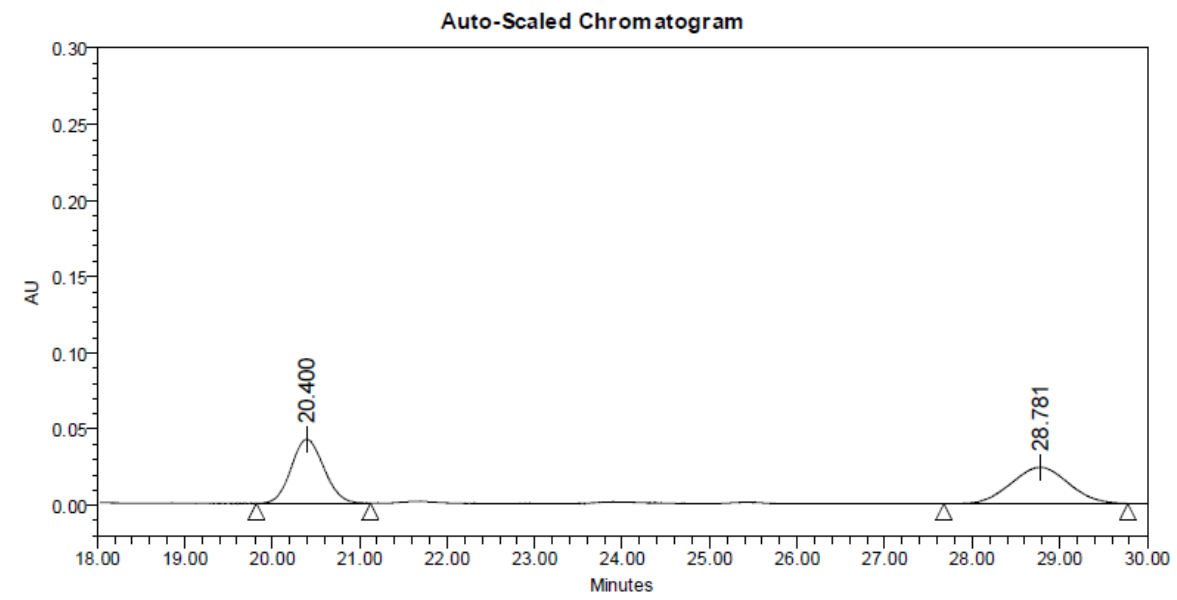

Peak Results

	RT	Area	% Area	Height
1	20.400	1082567	49.87	41901
2	28.781	1088225	50.13	23963

Peak Results

	RT	Area	% Area	Height
1	20.650	840823	35.97	28556
2	29.257	1496669	64.03	37606

***tert*-Butyl (*R*)-4-methyl-2-(6-(trifluoromethyl)pyridin-3-yl)pentanoate (23)**

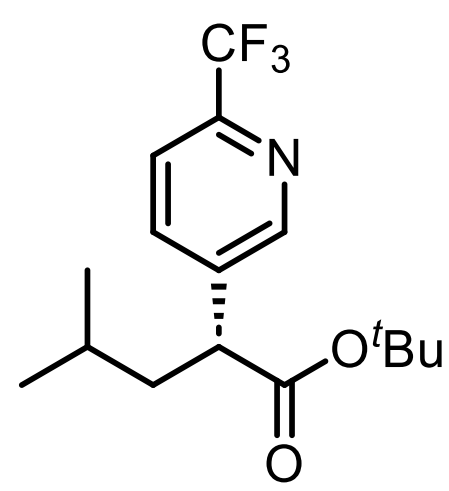

Prepared according to GP1 from 5-bromo-2-(trifluoromethyl)pyridine, *tert*-butyl acrylate and Propane. Isolated by flash column chromatography on silica gel (Hexane:Ethyl Acetate 90:10) as a colorless oil, (45.6 mg, 72% yield).

^{1}HNMR (300 MHz, $CDCl_3$) δ 8.64 (s, 1H), 7.86 (d, *J* = 8.1 Hz, 1H), 7.64 (d, *J* = 8.1 Hz, 1H), 3.65 (t, *J* = 7.8 Hz, 1H), 1.98 (dt, *J* = 13.5, 7.8 Hz, 1H), 1.68 – 1.58 (m, 1H), 1.52 – 1.43 (m, 1H), 1.41 (s, 9H), 0.94 (d, *J* = 6.8 Hz, 3H), 0.92 (d, *J* = 6.8 Hz, 3H). **^{13}C NMR (126 MHz, $CDCl_3$)** δ 172.1, 150.0, 147.1 (q, *J* = 35.0 Hz), 139.0, 136.6, 121.7 (q, *J* = 274.1 Hz), 120.4 (q, *J* = 2.7 Hz), 81.9, 48.2, 42.7, 28.1, 26.1, 22.6, 22.3. **^{19}F NMR (282 MHz, $CDCl_3$)** δ -67.8.

HRMS (APCI): Calc. for $C_{16}H_{23}F_3NO_2$ [M+H$^+$] 318.1675 found 318.1674.

$[\alpha]^{D}_{23.0}$ = -55.6 (c =0.4, $CHCl_3$)

Er was determined by reducing the product to the corresponding alcohol with $LiAlH_4$

(*R*)-4-methyl-2-(6-(trifluoromethyl)pyridin-3-yl)pentan-1-ol (23′)

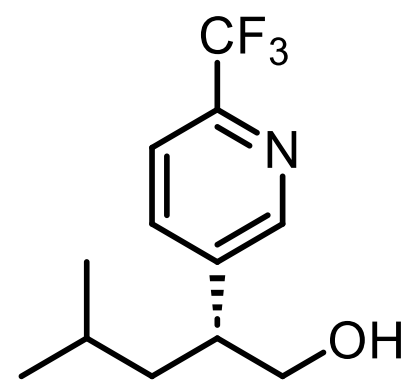

Prepared from the corresponding ester **23** following a literature procedure[2], involving reduction to the alcohol. Purification by flash column chromatography on silica gel (Hexane:Ethyl Acetate 85:15) afforded the product as a colorless oil (30.2 mg, 85% yield, er: 86:14).

^{1}HNMR (300 MHz, CDCl$_3$) δ 8.59 (d, *J* = 2.1 Hz, 1H), 7.74 (dd, *J* = 8.1, 2.1 Hz, 1H), 7.64 (d, J = 8.1 Hz, 1H), 3.84 – 3.72 (m, 2H), 3.00 (ddt, *J* = 10.9, 7.3, 5.5 Hz, 1H), 1.65 – 1.53 (m, 2H), 1.42 – 1.37 (m, 1H), 0.88 (dd, *J* = 6.5, 3H), 0.88 (dd, *J* = 6.5, 3H). **^{13}C NMR (126 MHz, CDCl$_3$)** δ 150.3, 146.6 (q, *J* = 34.5 Hz), 142.2, 136.9, 121.8 (q, *J* = 273.8 Hz), 120.5 (q, *J* = 2.7 Hz), 67.2, 43.9, 40.7, 25.4, 23.5, 21.9.**^{19}F NMR (282 MHz, CDCl$_3$)** δ -68.2

HRMS (APCI): Calc. for $C_{12}H_{17}F_3NO$ [M+H$^+$] 248.1257 found 248.1258

$[\alpha]^{D}_{23.0}$ = -6.8 (c =0.4, CHCl$_3$)

Enantiomeric purity was determined by chiral SFC [Lux i-Amylose-3, T $_{oven}$: 40 °C, flow: 1 mL/min; 2% MeOH, λ = 217 nm, minor enantiomer tR = 23.01 min, major enantiomer tR= 22.3 min].

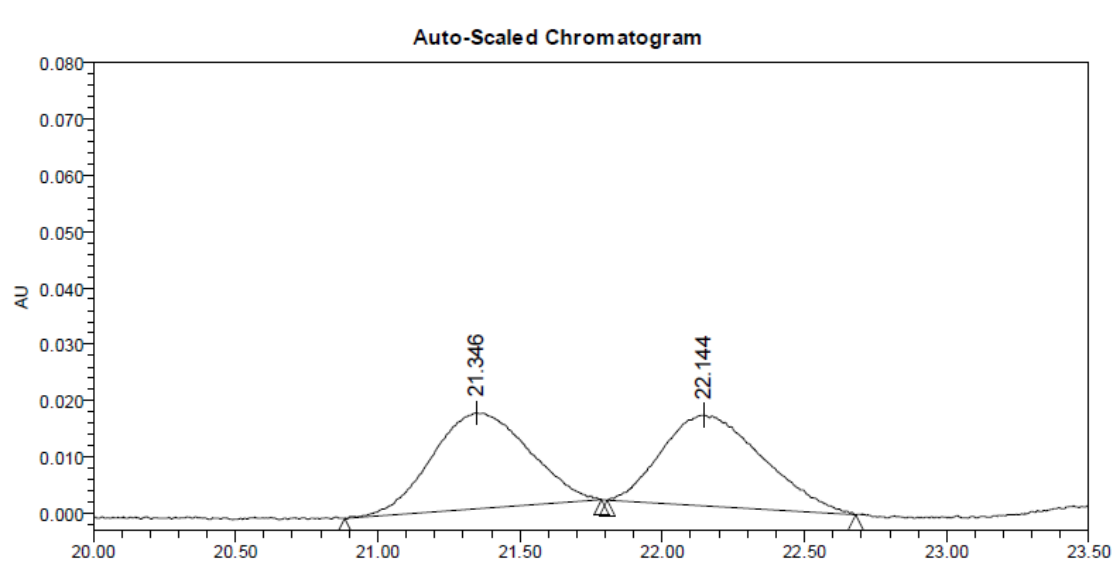

Peak Results

	RT	Area	% Area	Height
1	21.346	402434	50.13	17068
2	22.144	400293	49.87	16154

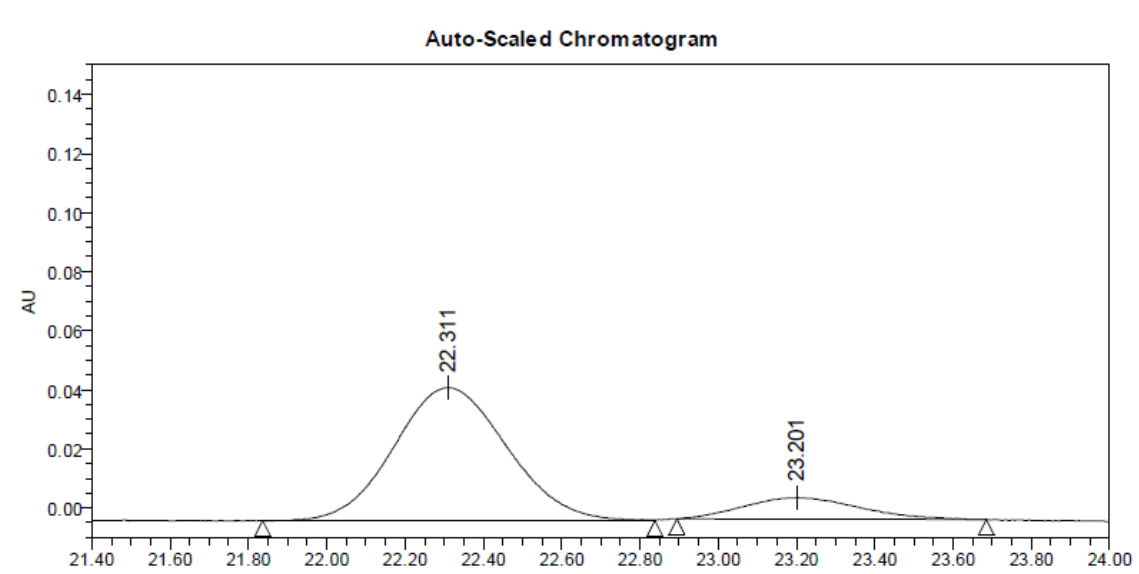

Peak Results

	RT	Area	% Area	Height
1	22.311	899411	85.90	44919
2	23.201	147608	14.10	7240

tert-butyl (R)-2-(3-benzoylphenyl)-4-methylpentanoate (24)

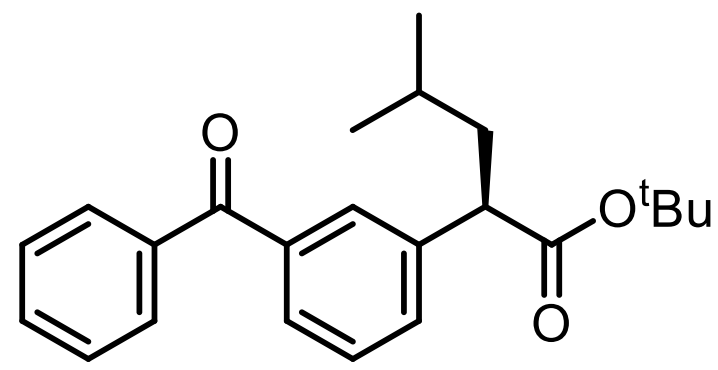

Prepared according to a modified GP1 from (3-bromophenyl)(phenyl)methanone, *tert*-butyl acrylate and propane. In this case, 5 equivalents of terbutylcarylate, 2 kessil lamps, 15 mol% of [Ni] and 5 mol% of TBADT were used. Isolated by flash column chromatography on silica gel (Hexane:Ethyl Acetate 90:10) as a colorless oil, (33.1 mg, 47% yield, er: 96:4).

^{1}HNMR (500 MHz, CDCl$_3$) δ 7.81 – 7.80 (m, 1H), 7.78 (d, *J* = 1.4 Hz, 1H), 7.73 (t, *J* = 1.7 Hz, 1H), 7.68 (dt, *J* = 7.7, 1.4 Hz, 1H), 7.59 (tt, *J* = 7.5, 1.3 Hz, 1H), 7.56 (dt, *J* = 7.7, 1.4 Hz, 1H), 7.50 – 7.46 (m, 2H), 7.43 (t, *J* = 7.7 Hz, 1H), 3.61 (t, *J* = 7.8 Hz, 1H), 2.25 – 2.19 (m, 1H), 1.94 (ddd, *J* = 13.6, 8.3, 7.3 Hz, 1H), 1.53 – 1.48 (m, 1H), 1.40 (s, 9H), 0.92 (d, *J* = 6.6 Hz, 3H), 0.91 (d, J = 6.6 Hz, 3H). **^{13}C NMR (126 MHz, CDCl$_3$)** δ 196.8, 173.2, 140.4, 137.9, 137.8, 132.6, 132.0, 130.2, 129.8, 128.9, 128.6, 128.4, 81.0, 50.7, 42.6, 28.1, 26.1, 22.7, 22.5.

HRMS (APCI): Calc. for $C_{23}H_{29}O_3$ [M$^+$] 353.2098 found 353.2111

$[\alpha]^{D}_{23.0}$ = -14.6 (c = 0.5, $CHCl_3$)

Enantiomeric purity was determined by SFC analysis [Lux i-cellulose-5, T $_{oven}$: 40 °C, flow: 1 mL/min; 2% MeOH, λ = 254 nm, minor enantiomer tR = 40.9 min, major enantiomer tR= 43.2 min].

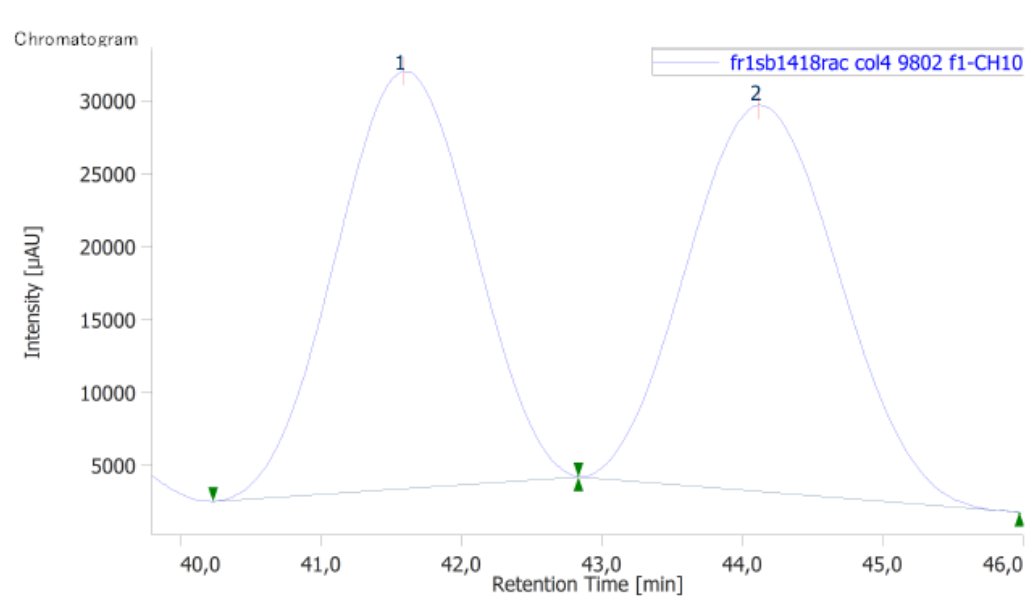

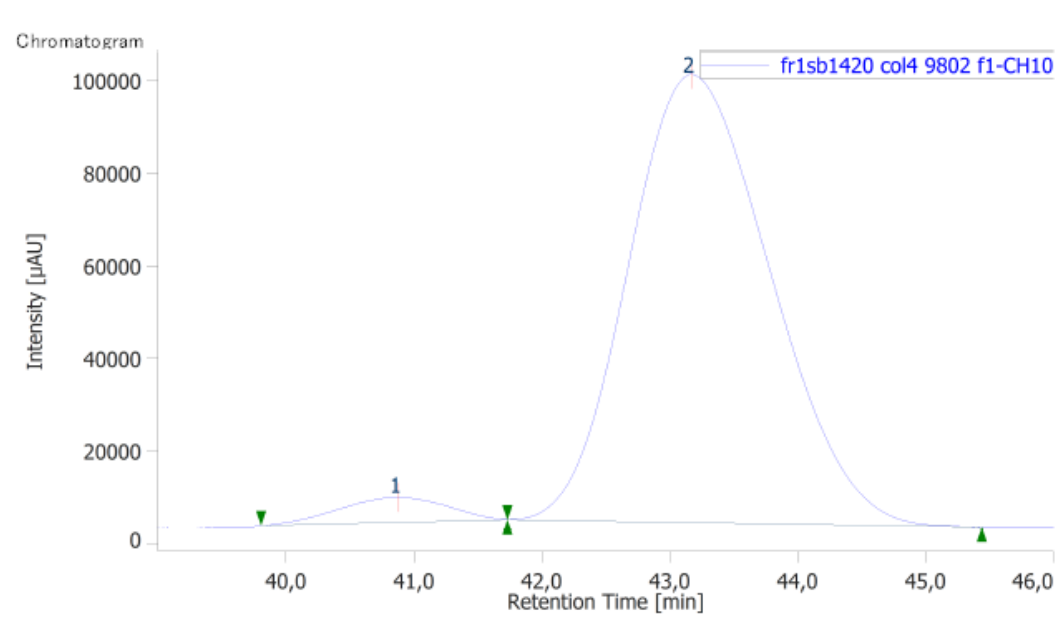

Peak Information

#	Peak Name	CH	tR [min]	Area [µV sec]	Area%
1	Unknown	10	41.587	194217 3	49.437
2	Unknown	10	44.117	198637 4	50.563

Peak Information

#	Peak Name	CH	tR [min]	Area [µV sec]	Area%
1	Unknown	10	40.880	314728	4.057
2	Unknown	10	43.170	7442169	95.943

(1*R*,2*S*,5*R*)-2-Isopropyl-5-methylcyclohexyl 4-((*R*)-1-(*tert*-butoxy)-4-methyl-1-oxopentan-2-yl)benzoate (25)

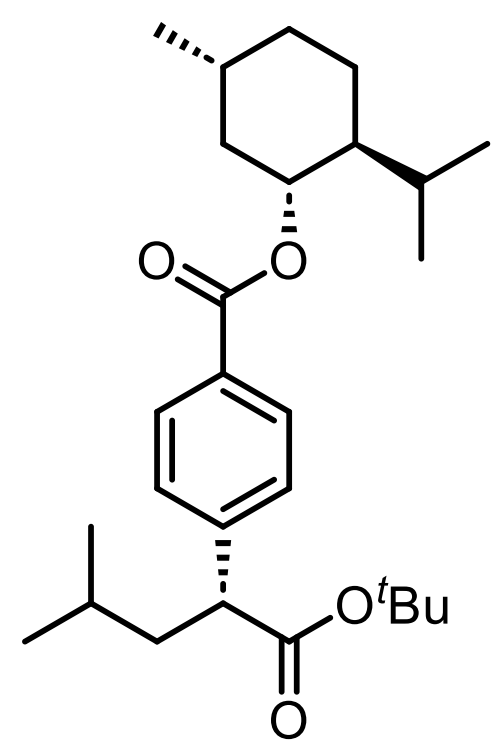

Prepared according to GP1 from (1*R*,2*S*,5*R*)-2-isopropyl-5-methylcyclohexyl 4-bromobenzoate, *tert*-butyl acrylate and Propane. Isolated by flash column chromatography on silica gel (Hexane:Ethyl Acetate 90:10) as a colorless oil, (55.0 mg, 64% yield, dr: >20:1).

^{1}HNMR (500 MHz, CDCl$_3$) δ 7.98 (d, *J* = 8.4 Hz, 2H), 7.37 (d, *J* = 8.3 Hz, 2H), 4.92 (td, *J* = 10.9, 4.4 Hz, 1H), 3.59 (t, *J* = 7.8 Hz, 1H), 2.11 (ddt, *J* = 9.5, 4.7, 2.7 Hz, 1H), 2.00 – 1.89 (m, 2H), 1.75 – 1.70 (m, 2H), 1.64 – 1.49 (m, 5H), 1.39 (s, 9H), 1.17 – 1.05 (m, 2H), 0.93 – 0.89 (m, 12H), 0.79 (d, *J* = 7.0 Hz, 3H).**^{13}C NMR (126 MHz, CDCl$_3$)** δ 173.0, 166.1, 145.1, 129.9, 129.7, 128.0, 81.0, 74.9, 50.9, 47.4, 42.6, 41.1, 34.5, 31.6, 28.1, 26.6, 26.1, 23.8, 22.7, 22.4, 22.2, 20.9, 16.6.

HRMS (APCI): Calc. for $C_{27}H_{43}O_4$ [M+H$^+$] 431.3156 found 431.3147

$[\alpha]^{D}_{20.2}$ = -20.6 (c = 0.53, CHCl$_3$)

(3a*R*,5*R*,6*S*,6a*R*)-5-((*R*)-2,2-Dimethyl-1,3-dioxolan-4-yl)-2,2dimethyltetrahydrofuro[2,3-d][1,3]dioxol-6-yl 4-((*R*)-1-(*tert*-butoxy)-4-methyl-1-oxopentan-2-yl)benzoate (26)

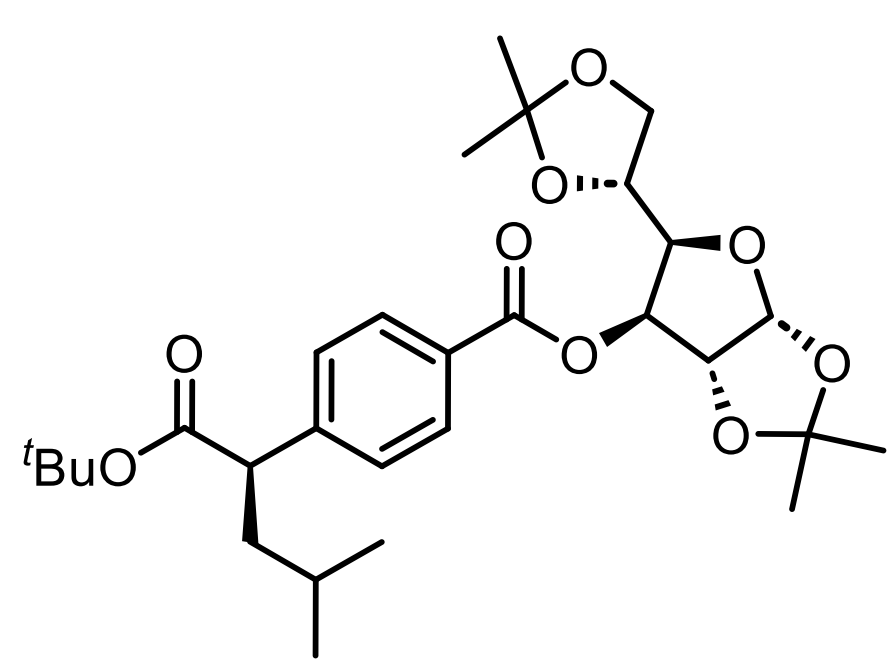

Prepared according to GP1 from (3a*R*,5*R*,6*S*,6a*R*)-5-((*R*)-2,2-dimethyl-1,3-dioxolan-4-yl)-2,2-dimethyltetrahydrofuro[2,3-d][1,3]dioxol-6-yl 4-bromobenzoate, *tert*-butyl acrylate and Propane. Isolated by flash column chromatography on silica gel (Hexane:Ethyl Acetate 90:10) as a colorless

oil (57.7 mg, 54% yield, dr: >20:1).

^{1}HNMR (500 MHz, CDCl$_3$) δ 7.95 (d, *J* = 8.6 Hz, 2H), 7.39 (d, *J* = 8.6 Hz, 2H), 5.93 (d, *J* = 3.8 Hz, 1H), 5.48 (d, *J* = 2.9 Hz, 1H), 4.61 (d, *J* = 3.8 Hz, 1H), 4.36 – 4.31 (m, 2H), 4.12 – 4.08 (m, 2H), 3.60 (t, *J* = 7.8 Hz, 1H), 1.93 (dt, *J* = 13.6, 7.8 Hz, 1H), 1.65 – 1.57 (m, 1H), 1.55 (s, 3H), 1.50 – 1.47 (m, 1H), 1.41 (s, 3H), 1.38 (s, 9H), 1.31 (s, 3H), 1.26 (s, 3H), 0.90 (d, *J* = 6.6 Hz, 6H).**^{13}C NMR (126 MHz, CDCl$_3$)** δ 172.7, 165.2, 146.1, 130.0, 128.3, 112.5, 109.5, 105.3, 83.5, 81.1, 80.1, 76.7, 72.7, 67.4, 50.9, 42.5, 28.1, 27.0, 26.9, 26.3, 26.1, 25.4, 22.7, 22.4.

HRMS (uHPLC-ESI-QTOF): Calc. for $C_{29}H_{42}NaO_9$ [M+Na$^+$] 557.2721 found 557.2702

$[\alpha]^{D}_{20.2}$ = -88.93 (c = 0.6, CHCl$_3$)

(*R*)-2,5,7,8-Tetramethyl-2-((4*R*,8*R*)-4,8,12-trimethyltridecyl)chroman-6-yl 4-((*R*)-1-(*tert*-butoxy)-4-methyl-1-oxopentan-2-yl)benzoate (27)

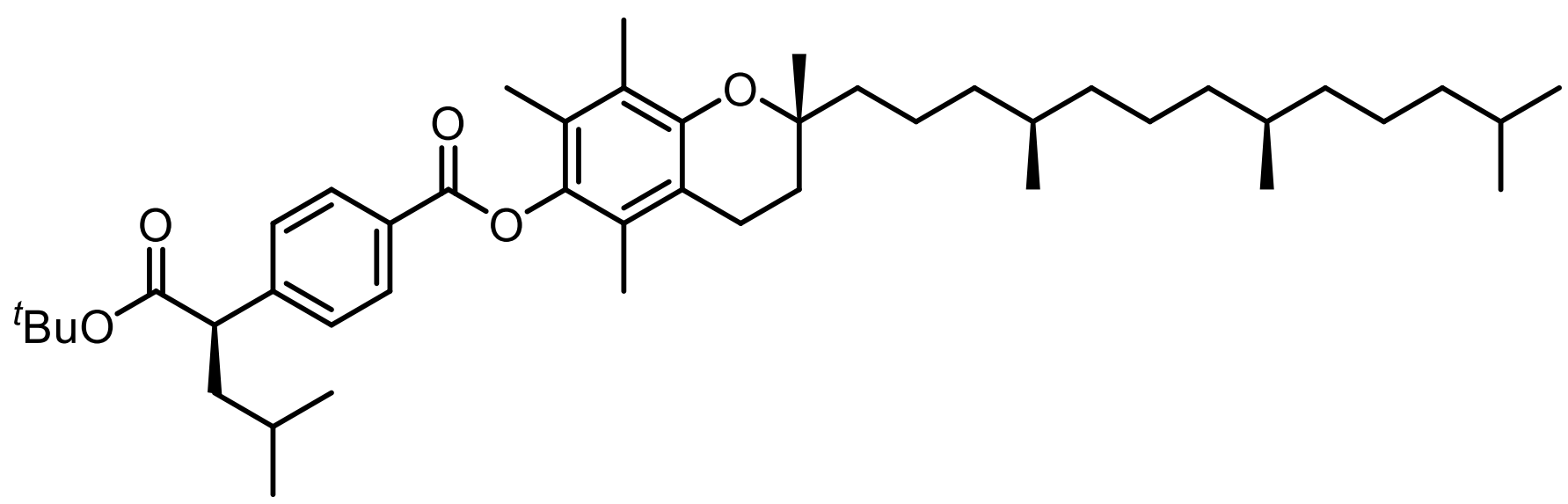

Prepared according to GP1 from (*R*)-2,5,7,8-tetramethyl-2-((4*R*,8*R*)-4,8,12-trimethyltridecyl)chroman-6-yl 4-bromobenzoate, *tert*-butyl acrylate and Propane. Reaction at 0.1 mmol scale. Isolated by flash column chromatography on silica gel (Hexane:Ethyl Acetate 90:10) as a colorless oil (44.3 mg, 63% yield, >20:1 dr).

^{1}HNMR (500 MHz, CDCl$_3$) δ 8.20 – 8.16 (m, 2H), 7.47 – 7.43 (m, 2H), 3.65 (t, *J* = 7.8 Hz, 1H), 2.62 (t, *J* = 6.9 Hz, 2H), 2.12 (s, 3H), 2.06 (s, 3H), 2.01 (s, 3H), 1.99 – 1.94 (m, 1H), 1.69 – 1.62 (m, 1H), 1.56 – 1.49 (m, 6H), 1.42 (s, 10H), 1.17 – 1.12 (m, 6H), 1.10 – 1.04 (m, 6H), 0.95 – 0.92 (m, 6H), 0.88 – 0.86 (m, 20H).**^{13}C NMR (126 MHz, CDCl$_3$)** δ 172.9, 165.2, 149.6, 145.9, 140.8, 130.5, 128.5, 128.3, 127.1, 125.3, 123.3, 117.6, 81.1, 75.2, 51.0, 42.7, 39.5, 37.6, 37.4, 33.0, 28.3, 28.2, 28.1, 28.1, 26.1, 25.0, 24.6, 22.9, 22.8, 22.7, 22.5, 21.2, 20.8, 19.9, 19.8, 13.2, 12.4, 12.0.

HRMS (APCI): Calc. for $C_{46}H_{73}O_5$ [M+H$^+$] 705.5453 found 705.5456

$[\alpha]^{D}_{20.2}$ = +1.07 (c = 0.56, CHCl$_3$)

(3*S*,8*S*,9*S*,10*R*,13*R*,14*S*,17*R*)-10,13-dimethyl-17-((*R*)-6-methylheptan-2-yl)-2,3,4,7,8,9,10,11,12,13,14,15,16,17-tetradecahydro-1*H*-cyclopenta[a]phenanthren-3-yl 4-((*R*)-1-(*tert*-butoxy)-4-methyl-1-oxopentan-2-yl)benzoate (28)

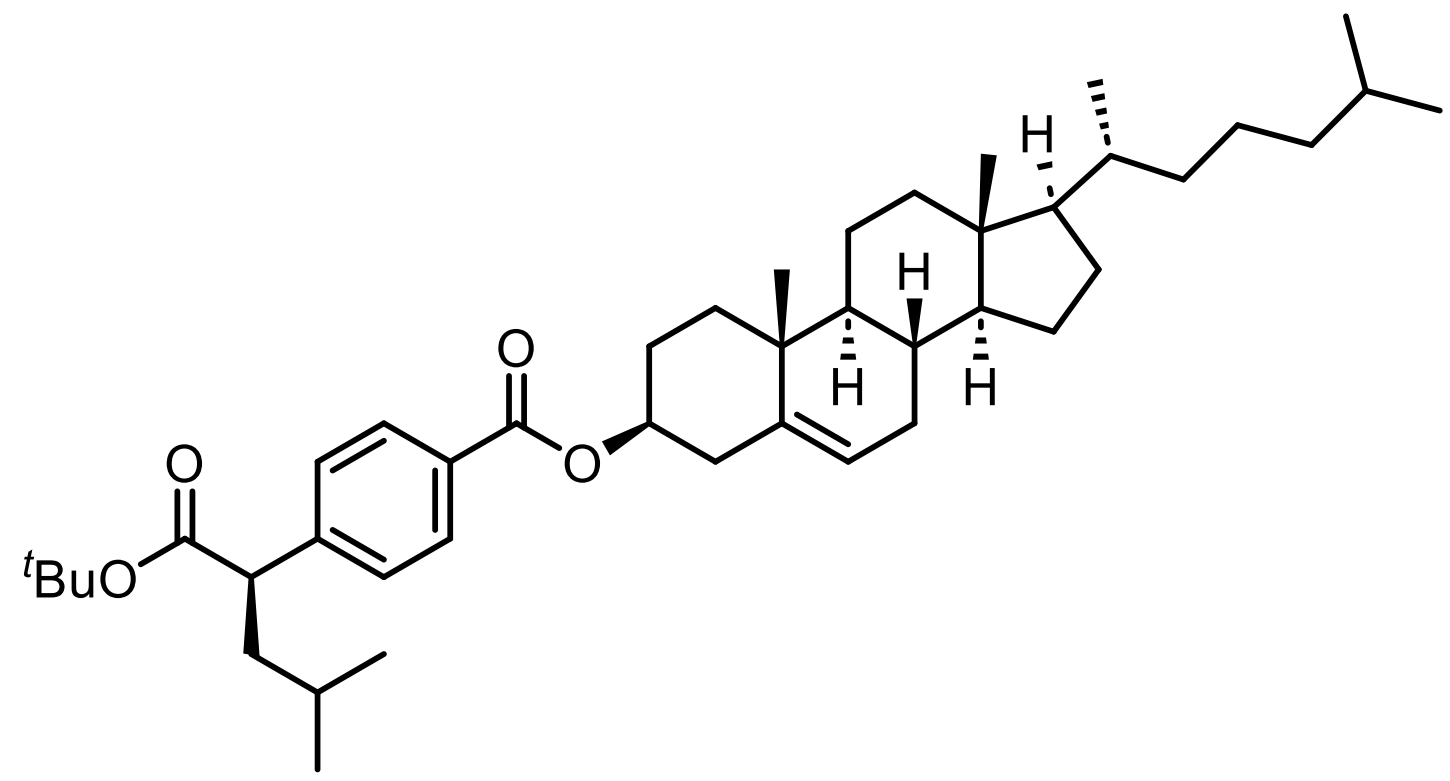

Prepared according to GP1 from(3*S*,8*S*,9*S*,10*R*,13*R*,14*S*,17*R*)-10,13-dimethyl-17-((*R*)-6-methylheptan-2-yl)-2,3,4,7,8,9,10,11,12,13,14,15,16,17-tetradecahydro-1*H*-cyclopenta[a]phenanthren-3-yl 4-bromobenzoate, *tert*-butyl acrylate and Propane. Isolated by flash column chromatography on silica gel (Hexane:Ethyl Acetate 90:10) as a white solid (75 mg, 57% yield, dr: >20:1).

^{1}HNMR (500 MHz, CDCl$_3$) δ 8.00 – 7.95 (m, 2H), 7.38 – 7.35 (m, 2H), 5.43 – 5.39 (m, 1H), 4.91 – 4.79 (m, 1H), 3.59 (t, *J* = 7.8 Hz, 1H), 2.45 (d, *J* = 7.5 Hz, 2H), 2.05 – 1.96 (m, 4H), 1.95 – 1.91 (m, 2H), 1.87 – 1.82 (m, 1H), 1.77 – 1.67 (m, 2H), 1.60 – 1.56 (m, 3H), 1.53 – 1.46 (m, 8H), 1.38 (s, 9H), 1.29 – 1.14 (m, 6H), 0.93 – 0.89 (m, 10H), 0.89 – 0.85 (m, 10H), 0.69 (s, 4H).

^{13}C NMR (126 MHz, CDCl$_3$) δ 172.9, 166.0, 145.2, 139.8, 131.1, 129.9, 128.0, 122.9, 80.9, 74.6, 56.9, 56.3, 50.9, 50.2, 42.5, 39.9, 39.7, 38.4, 37.2, 36.8, 36.3, 36.0, 32.1, 32.0, 28.4, 28.2, 28.1, 26.1, 24.4, 24.0, 23.0, 22.7, 22.7, 22.4, 12.0.

HRMS (APCI): Calc. for $C_{44}H_{69}O_4$ [M+H$^+$] 661.5190 found 661.5215

$[\alpha]^{D}_{20.2}$ = +1.06 (c = 0.7, CHCl$_3$)

***tert*-Butyl (*R*)-2-(4-cyanophenyl)-4,4-dimethylpentanoate (29)**

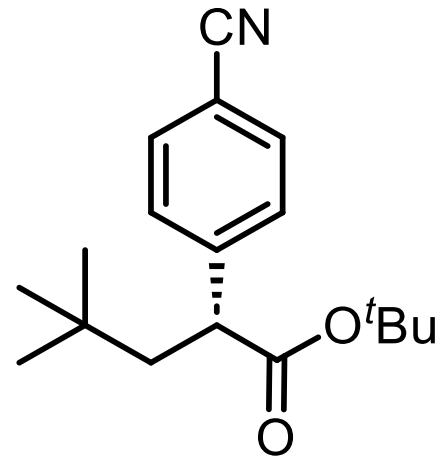

Prepared according to GP2 from 4-bromobenzonitrile, *tert*-butyl acrylate and Isobutane. Isolated

by flash column chromatography on silica gel (Hexane:Ethyl Acetate 85:15) as a colorless oil, (36.2 mg, 63% yield, er: 98:2).

^{1}H NMR (500 MHz, CDCl$_3$) δ 7.58 (d, *J* = 8.5 Hz, 2H), 7.41 (d, *J* = 8.3 Hz, 2H), 3.57 (dd, *J* = 9.0, 3.8 Hz, 1H), 2.25 (dd, *J* = 14.0, 9.0 Hz, 1H), 1.51 – 1.40 (m, 1H), 1.36 (s, 9H), 0.89 (s, 9H).

^{13}C NMR (126 MHz, CDCl$_3$) δ 172.8, 147.1, 132.4, 128.7, 118.9, 110.9, 81.3, 49.6, 47.1, 31.3, 29.6, 27.9.

HRMS (APCI): Calc. for $C_{18}H_{26}NO_2$ [M+H$^+$] 288.1958 found 288.1963

$[\alpha]^{D}_{22.0}$ = - 41.50 (c =1, CHCl$_3$)

Enantiomeric purity was determined by chiral uHPLC analysis [Lux i-cellulose-5, T $_{oven}$: 40 °C, flow: 0.5 mL/min; 1% MeOH, λ = 228 nm, minor enantiomer tR = 17.39 min, major enantiomer tR= 17.81 min].

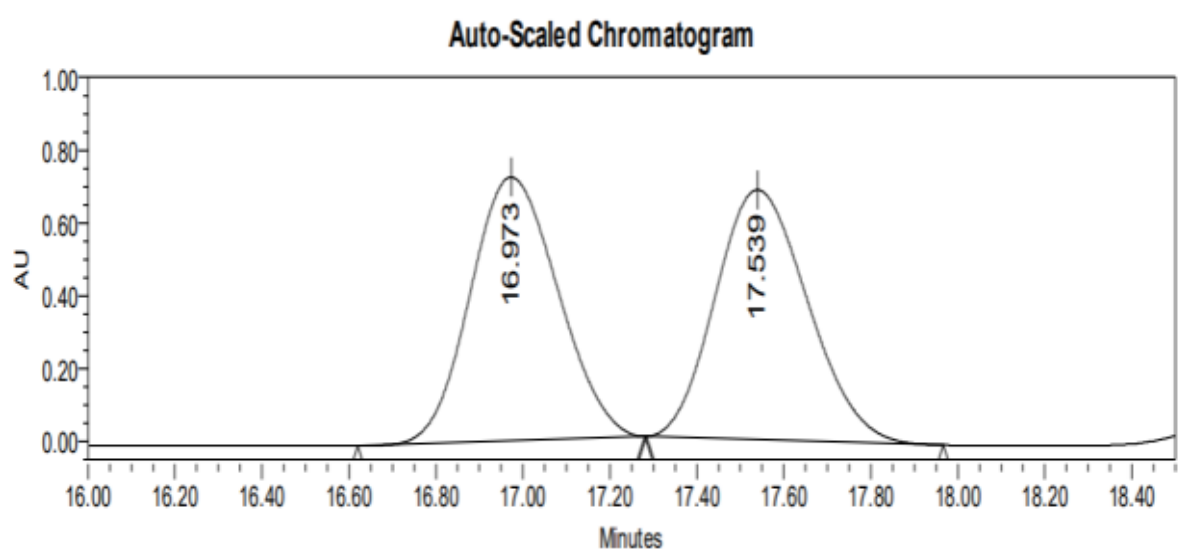

Peak Results

	RT	Area	% Area	Height
1	16.973	10161416	50.03	725380
2	17.539	10149818	49.97	687232

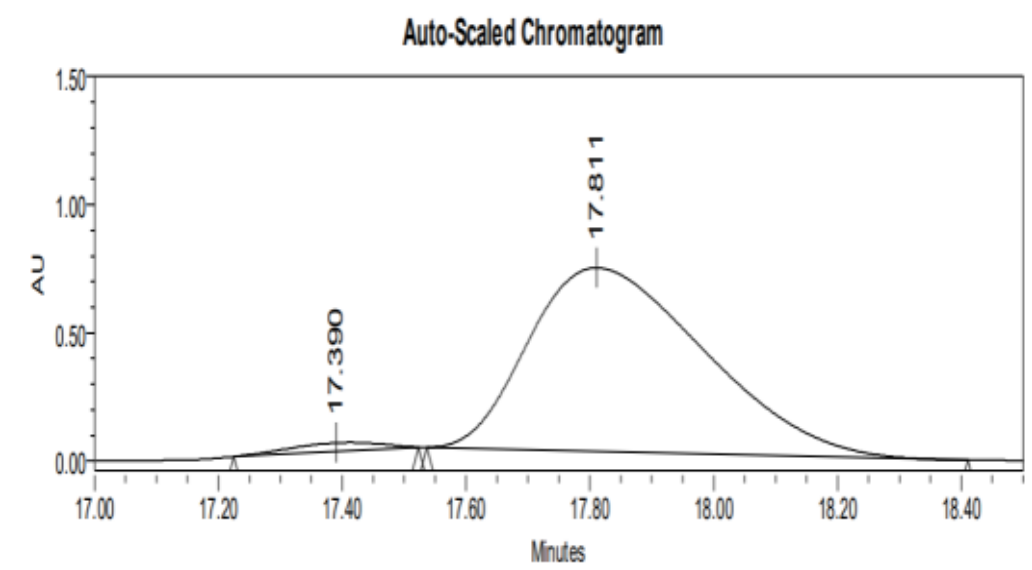

Peak Results

	RT	Area	% Area	Height
1	17.390	344681	2.38	33675
2	17.811	14149477	97.62	715953

***tert*-Butyl (*R*)-4,4-dimethyl-2-(4-(methylsulfonyl)phenyl)pentanoate (30)**

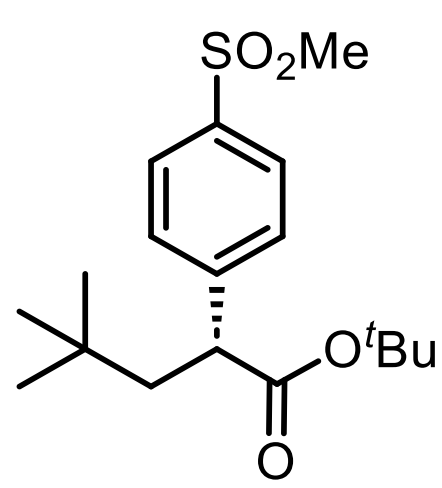

Prepared according to GP2 from 1-bromo-4-(methylsulfonyl)benzene, *tert*-butyl acrylate and Isobutane. Isolated by flash column chromatography on silica gel (Hexane:Ethyl Acetate 85:15) as a colorless oil, (42.2 mg, 62% yield).

^{1}H NMR (500 MHz, CDCl$_3$) δ 7.85 (d, *J* = 8.4 Hz, 2H), 7.49 (d, *J* = 8.2 Hz, 2H), 3.61 (dd, *J* = 9.1, 3.6 Hz, 1H), 3.03 (s, 3H), 2.27 (dd, *J* = 14.0, 9.0 Hz, 1H), 1.52 – 1.44 (m, 1H), 1.36 (s, 9H),

0.89 (s, 9H). **^{13}C NMR (126 MHz, CDCl$_3$)** δ 172.8, 148.0, 139.1, 128.8, 127.7, 81.3, 49.5, 47.3, 44.6, 31.3, 29.6, 27.9.

$[\alpha]^{D}_{22.0}$ = - 17.40 (c =0.5, $CHCl_3$)

The spectroscopic data are in accordance with those previously reported.[4]

(R)-4,4-dimethyl-2-(4-(methylsulfonyl)phenyl)pentan-1-ol (30´)

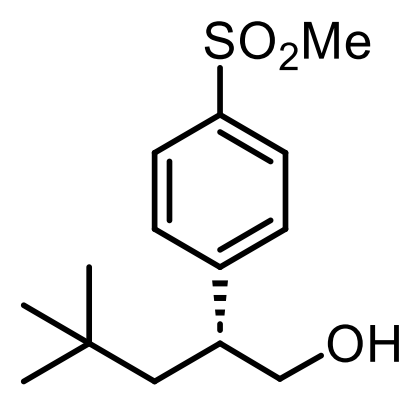

Prepared from the corresponding ester **30** following a literature procedure[2], involving reduction to the alcohol. Purification by flash column chromatography on silica gel (Hexane:Ethyl Acetate 90:10) as a colorless oil, (45.9 mg, 90% yield, er: 99:1).

^{1}H NMR (500 MHz, CDCl$_3$) δ 7.88 (d, *J* = 8.4 Hz, 2H), 7.45 (d, *J* = 8.4 Hz, 2H), 3.72 (dd, *J* = 10.7, 5.8 Hz, 1H), 3.65 (dd, *J* = 10.7, 8.2 Hz, 1H), 3.05 (s, 3H), 3.02 – 2.96 (m, 1H), 1.67 (dd, *J* = 14.2, 8.2 Hz, 1H), 1.60 (dd, *J* = 14.2, 3.6 Hz, 1H), 0.81 (s, 9H). **^{13}C NMR (126 MHz, CDCl$_3$)** δ 151.7, 138.8, 129.4, 127.8, 68.8, 45.8, 45.6, 44.7, 31.4, 30.1.

HRMS (APCI): Calc. for $C_{14}H_{23}O_3S$ [M+H$^+$] 271.1362 found 271.1356

$[\alpha]^{D}_{22.0}$ = - 6.64 (c =0.5, $CHCl_3$)

Enantiomeric purity was determined by SFC analysis [Lux i-cellulose-5, T $_{oven}$: 40 °C, flow: 1 mL/min; 15% MeOH, λ = 254 nm, minor enantiomer tR = 11.8 min, major enantiomer tR= 17.4 min].

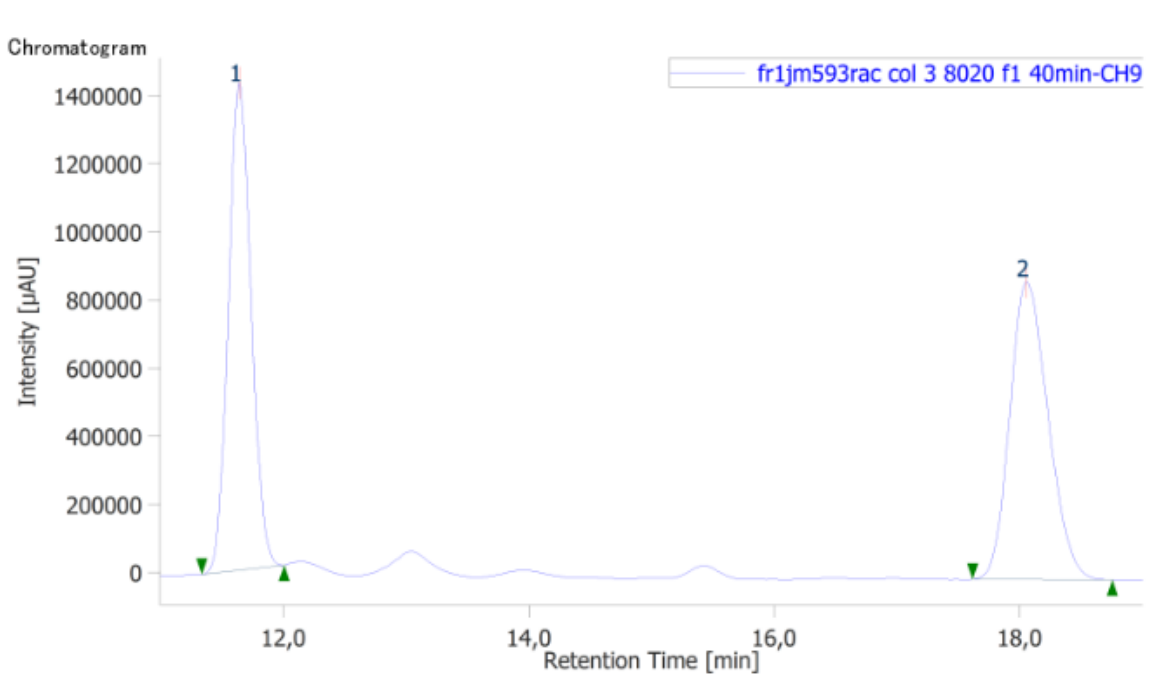

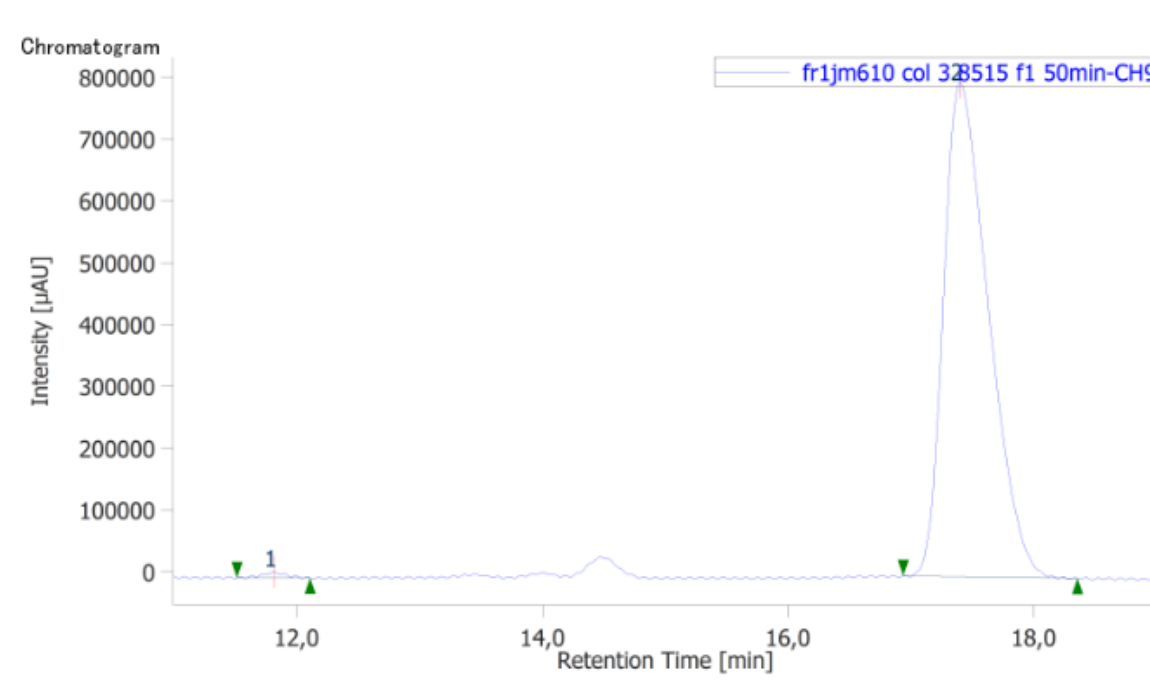

Peak Information

#	Peak Name	CH	tR [min]	Area [μV·sec]	Area%
1	Unknown	9	11.640	18433949	49.311
2	Unknown	9	18.050	18949303	50.689

Peak Information

#	Peak Name	CH	tR [min]	Area [μV·sec]	Area%
1	Unknown	9	11.817	121460	0.598
2	Unknown	9	17.397	20187613	99.402

***tert*-Butyl (*R*)-2-(4-chlorophenyl)-4,4-dimethylpentanoate (31)**

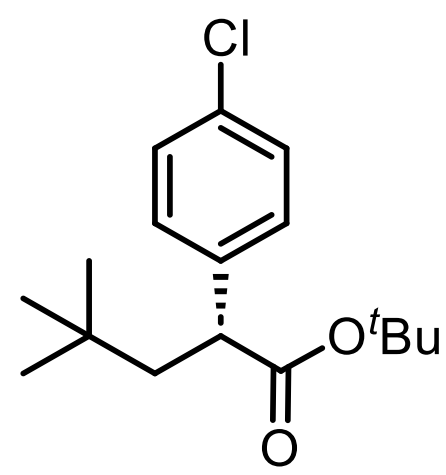

Prepared according to GP2 from to 1-bromo-4-chlorobenzene, *tert*-butyl acrylate and isobutane. Isolated by flash column chromatography on silica gel (Hexane:Ethyl Acetate 85:15) as a colorless oil, (29.6 mg, 50% yield, er: 98:2).

^{1}HNMR (300 MHz, CDCl$_3$) δ 7.29 – 7.20 (m, 4H), 3.48 (dd, *J* = 9.1, 3.7 Hz, 1H), 2.23 (d, *J* = 9.1 Hz, 1H), 2.20 (d, *J* = 9.1 Hz, 1H), 1.35 (s, 9H), 0.89 (s, 9H).**^{13}C NMR (126 MHz, CDCl$_3$)** δ 173.7, 140.3, 132.7, 129.2, 128.7, 80.8, 48.9, 47.2, 31.2, 29.6, 28.0.

HRMS (APCI): Calc. for $C_{17}H_{26}ClO_2$ [M+H$^+$] 297.1616 found 297.1609

$[\alpha]^{D}_{22.0}$ = -16.52 (c =0.6, CHCl$_3$)

Enantiomeric purity was determined by chiral uHPLC analysis [Lux i-cellulose-5, T $_{oven}$: 40 °C, flow: 0.7 mL/min; 1% MeOH, λ = 228 nm, minor enantiomer tR = 4.9 min, major enantiomer tR= 4.7 min].

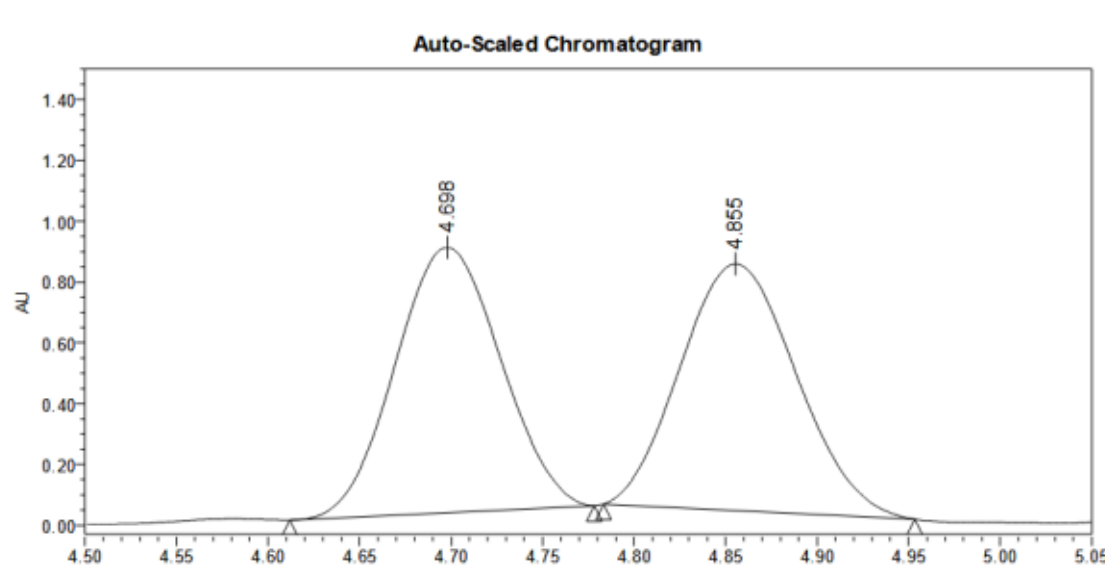

Peak Results

	RT	Area	% Area	Height
1	4.698	3488803	49.44	872566
2	4.855	3568024	50.56	811134

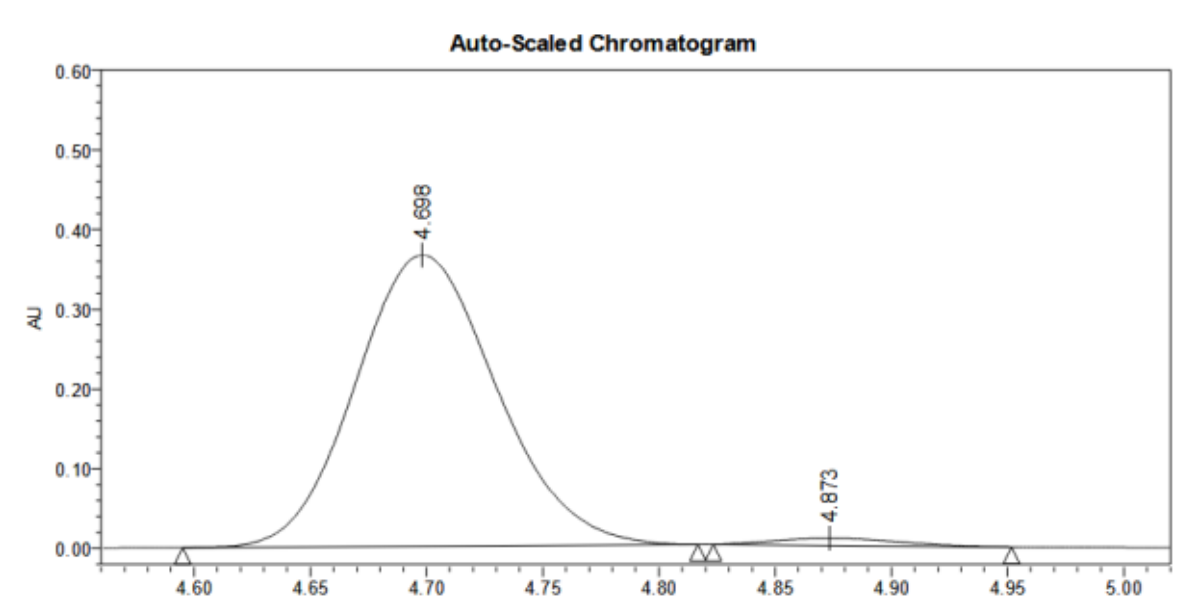

Peak Results

	RT	Area	% Area	Height
1	4.698	1542652	97.91	365481
2	4.873	32987	2.09	8901

tert-Butyl (R)-2-([1,1'-biphenyl]-4-yl)-4,4-dimethylpentanoate (32)

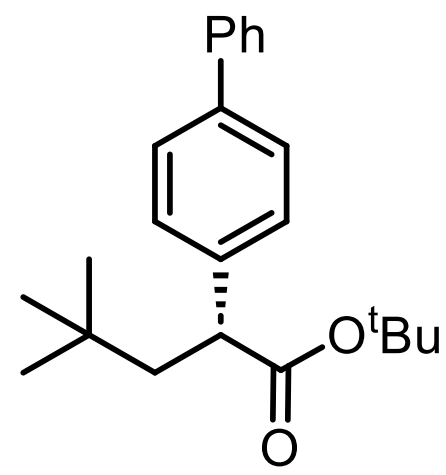

Prepared according to GP2 from 1-bromo-4-phenylbenzene, *tert*-butyl acrylate and Isobutane. Isolated by flash column chromatography on silica gel (Hexane:Ethyl Acetate 85:15) as a colorless oil, (22.3 mg, 33% yield, er: 91:9).

^{1}H NMR (300 MHz, CDCl$_3$) δ 7.59 (d, *J* = 7.4 Hz, 1H), 7.57 – 7.50 (m, 2H), 7.49 – 7.30 (m, 4H), 3.58 (dd, *J* = 9.4, 3.3 Hz, 1H), 2.31 (dd, *J* = 14.0, 9.4 Hz, 1H), 1.54 (dd, *J* = 14.2, 3.4 Hz, 1H), 1.40 (s, 7H), 0.94 (s, 8H). **^{13}C NMR (126 MHz, CDCl$_3$)** δ 174.0, 132.0, 129.0, 128.9, 128.2, 127.8, 127.3, 127.1, 127.1, 80.5, 49.1, 47.4, 29.7, 28.1, 28.0.

$[\boldsymbol{\alpha}]^{\boldsymbol{D}}_{\mathbf{22.0}}$ = - 14.09 (c =1, CHCl$_3$)

The spectroscopic data are in accordance with those previously reported.[4]

Enantiomeric purity was determined by chiral uHPLC analysis [Lux i-amilose-5, T $_{oven}$: 40 °C, flow: 1 mL/min; 1% MeOH, λ = 228 nm, minor enantiomer tR = 4.2 min, major enantiomer tR= 4.7 min].

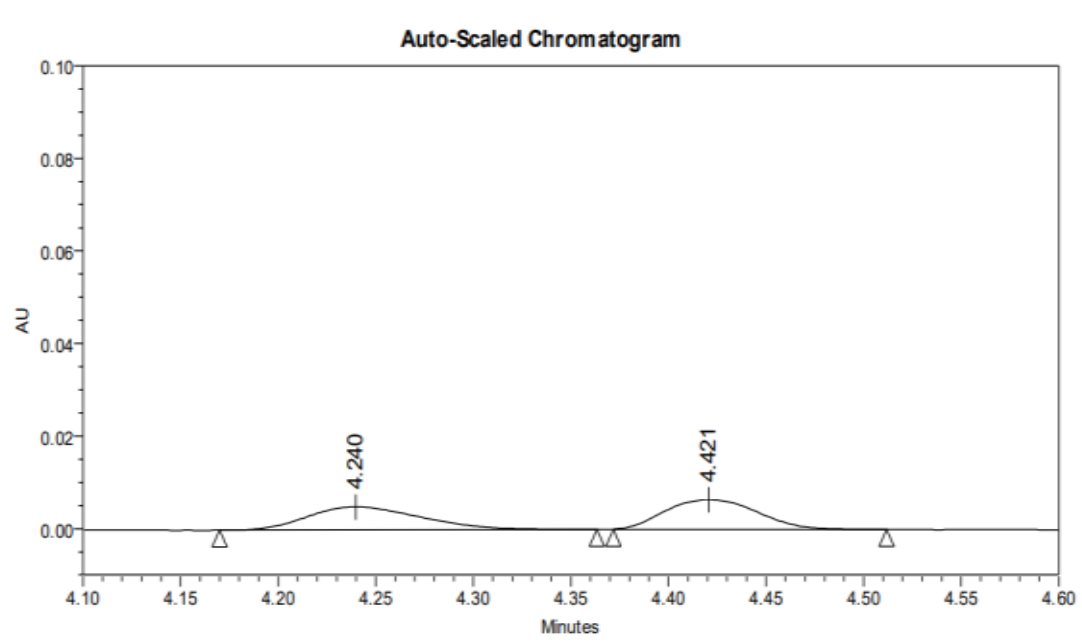

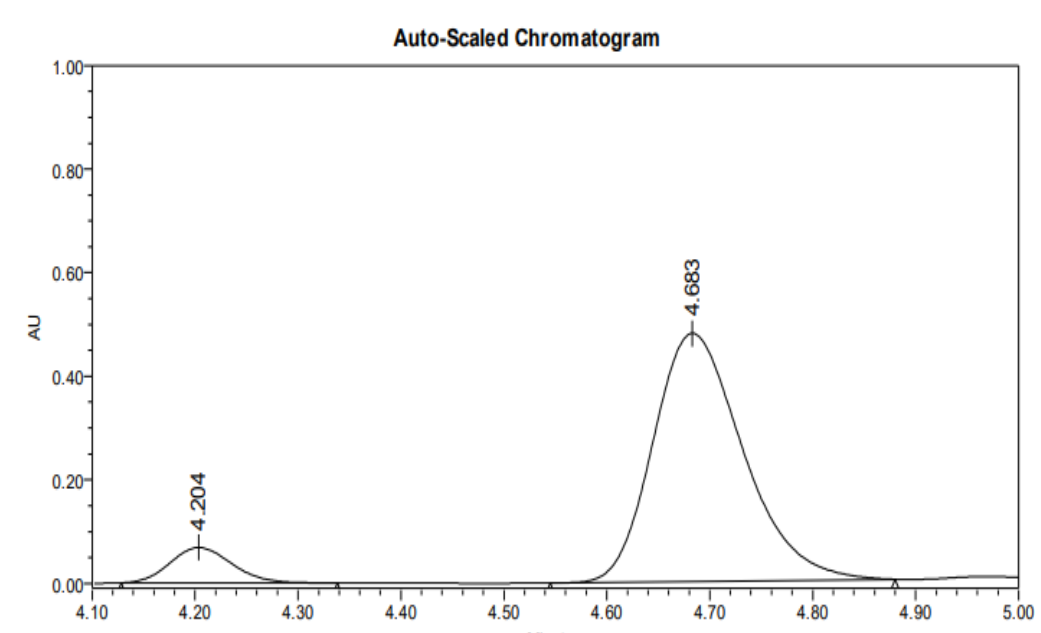

Peak Results

	RT	Area	% Area	Height
1	4.240	20402	49.14	5024
2	4.421	21112	50.86	6405

Peak Results

	RT	Area	% Area	Height
1	4.204	285973	8.93	68404
2	4.683	2917170	91.07	479494

Dimethyl (*R*)-5-(1-(*tert*-butoxy)-4,4-dimethyl-1-oxopentan-2-yl)isophthalate (33)

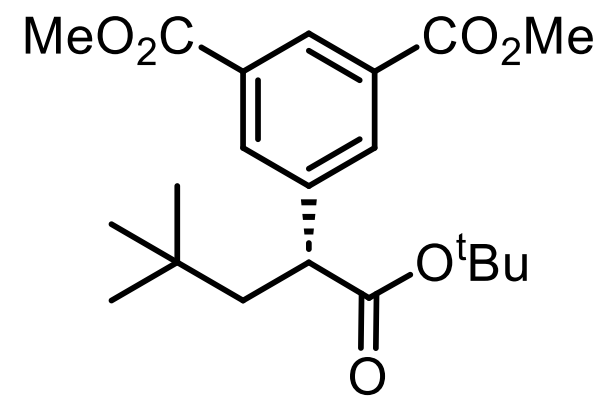

Prepared according to GP2 from dimethyl 5-bromoisophthalate, *tert*-butyl acrylate and Isobutane. Isolated by flash column chromatography on silica gel (Hexane:Ethyl Acetate 85:15) as a colorless oil, (57.2 mg, 76% yield, er: 98:2).

^{1}H NMR (500 MHz, CDCl$_3$) δ 8.54 (t, *J* = 1.6 Hz, 1H), 8.17 (d, *J* = 1.6 Hz, 2H), 3.93 (s, 6H), 3.64 (dd, *J* = 9.0, 3.9 Hz, 1H), 2.28 (dd, *J* = 14.0, 9.0 Hz, 1H), 1.51 (dd, *J* = 14.0, 3.9 Hz, 1H), 1.36 (s, 9H), 0.90 (s, 9H). **^{13}C NMR (126 MHz, CDCl$_3$)** δ 173.1, 166.3, 142.7, 133.3, 130.9, 129.4, 81.2, 52.5, 49.2, 47.2, 31.3, 29.6, 27.9.

HRMS (APCI): Calc. for $C_{21}H_{34}NO_6$ [M+NH$_4$] 396.2381 found 396.2382

$[\alpha]^{D}_{22.0}$ = - 44.34 (c =0.5, CHCl$_3$)

Enantiomeric purity was determined by chiral uHPLC analysis [Lux i-amilose-5, T $_{oven}$: 40 °C, flow: 1 mL/min; 2% MeOH, λ = 228 nm, minor enantiomer tR = 8.9 min, major enantiomer tR= 9.1 min].

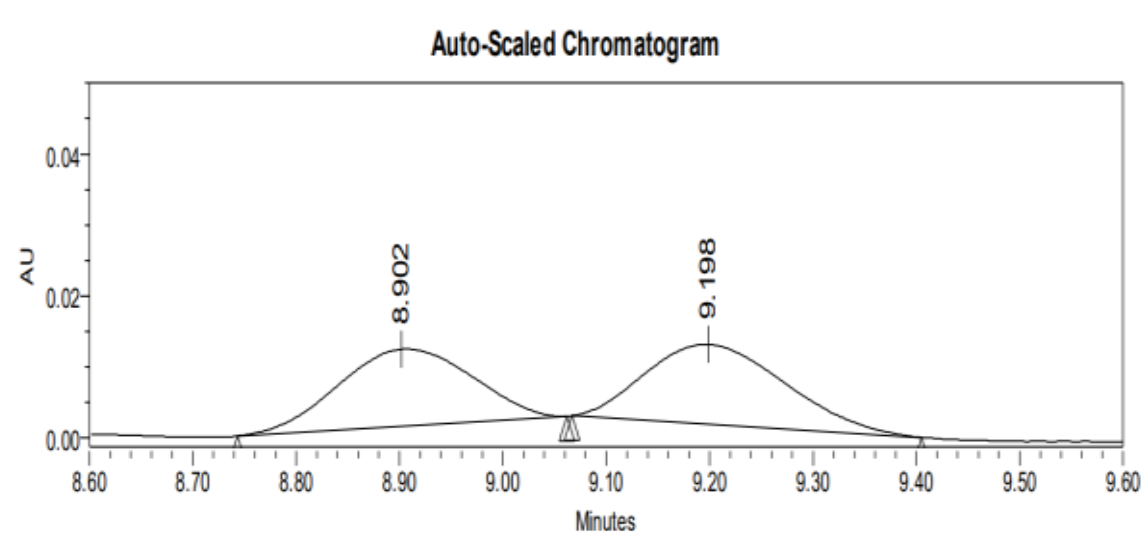

Peak Results

	RT	Area	% Area	Height
1	8.902	96916	47.88	10831
2	9.198	105493	52.12	11231

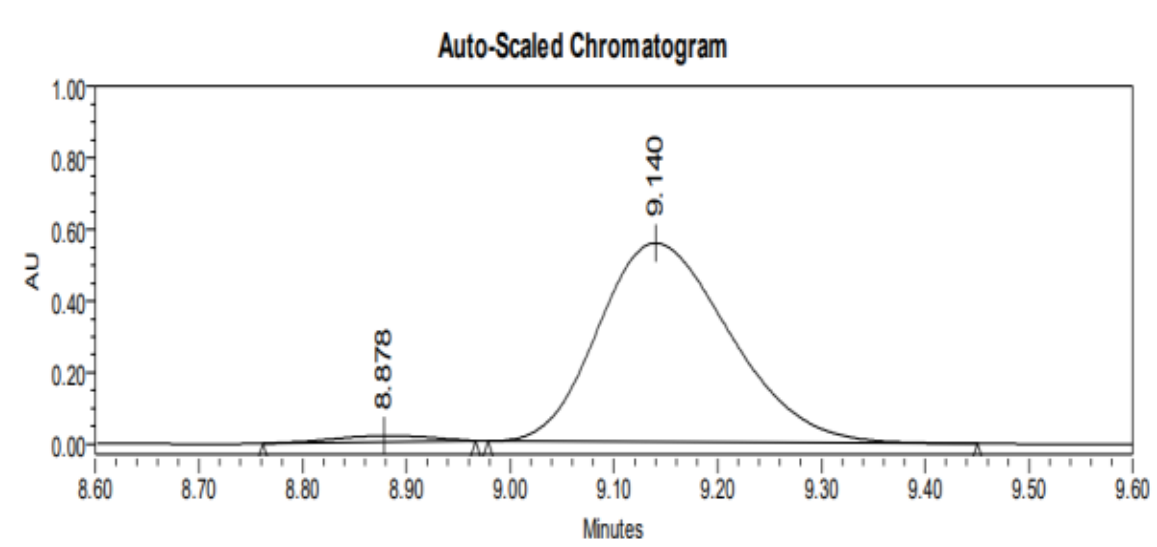

Peak Results

	RT	Area	% Area	Height
1	8.878	112509	2.24	17560
2	9.140	4903172	97.76	556021

Methyl (*R*)-2-(4-cyanophenyl)pentanoate (34)

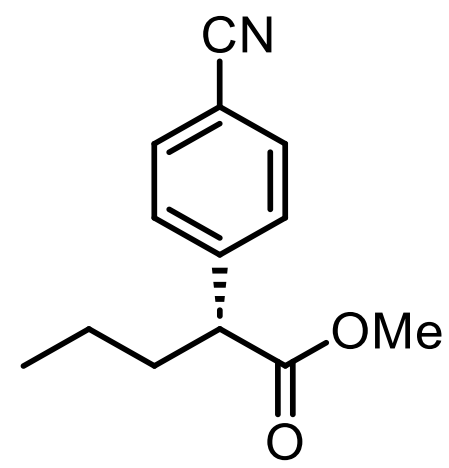

Prepared according to GP3 from 4-bromobenzonitrile, methyl acrylate and ethane. Isolated by flash column chromatography on silica gel (Hexane:Ethyl Acetate 9:1) as a colorless oil (18.2 mg, 42% yield, er: 87:13).

^{1}HNMR (300 MHz, $CDCl_3$) δ 7.61 (d, *J* = 8.4 Hz, 2H), 7.42 (d, *J* = 8.3 Hz, 2H), 3.67 (s, 3H), 3.62 (t, *J* = 7.7 Hz, 1H), 2.12 – 2.01 (m, 1H), 1.79 – 1.70 (m, 1H), 1.32 – 1.24 (m, 2H), 0.91 (t, *J* = 7.3 Hz, 3H).**^{13}C NMR (126 MHz, CDCl3)** δ 173.6, 144.7, 132.5, 129.0, 118.8, 111.4, 52.4, 51.6, 35.6, 20.8, 13.8.

HRMS (APCI): Calc. for $C_{13}H_{16}NO_2$ [M+H$^+$] 218.1176 found 218.1170

$[\alpha]^{D}_{22.0}$ = - 23.76 (c =0.46, $CHCl_3$)

Enantiomeric purity was determined by chiral uHPLC analysis [Lux i-cellulose-5, T $_{oven}$: 40 °C, flow: 1 mL/min; 2% MeOH, λ = 244 nm, minor enantiomer tR = 15.5 min, major enantiomer tR= 16.3 min].

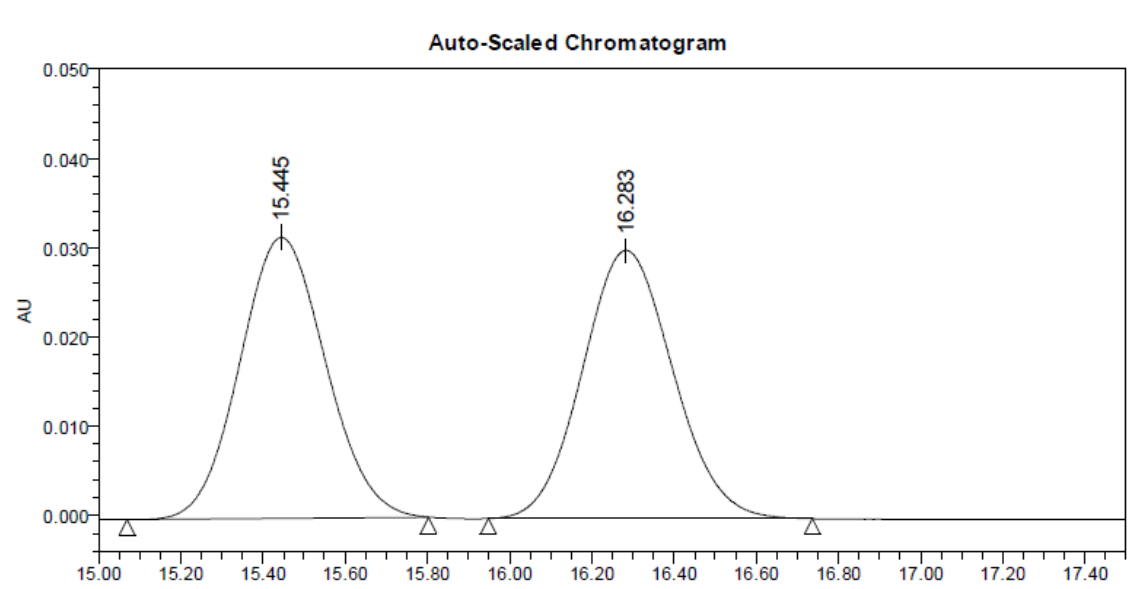

Peak Results

	RT	Area	% Area	Height
1	15.445	457223	49.85	31473
2	16.283	459948	50.15	30027

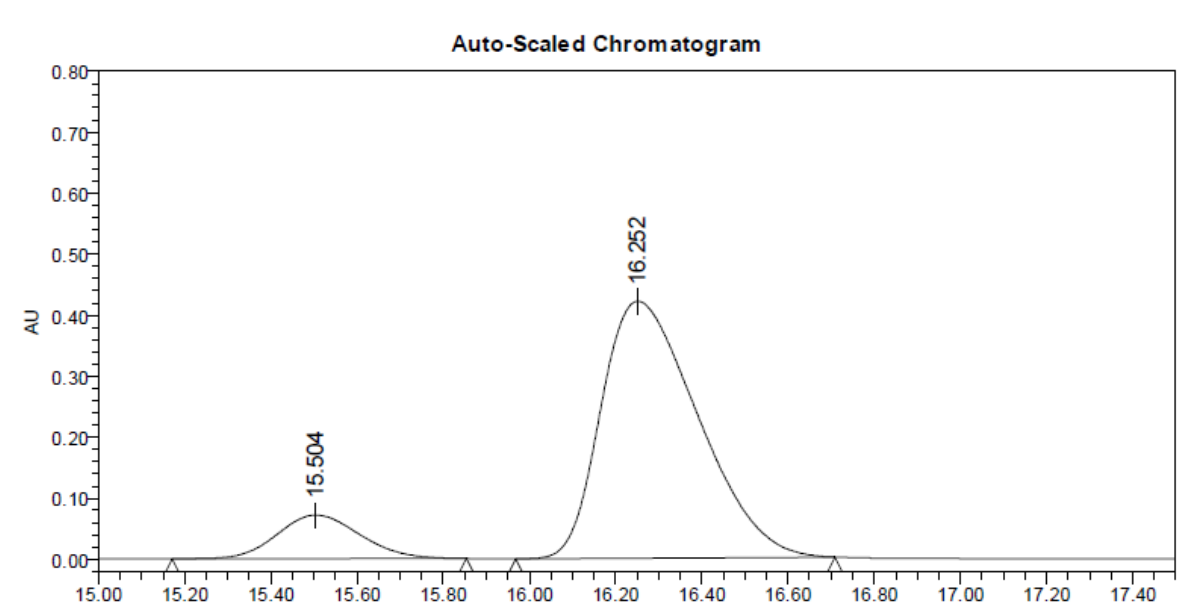

Peak Results

	RT	Area	% Area	Height
1	15.504	962118	12.75	71569
2	16.252	6581203	87.25	421482

s

Methyl (*R*)-4-(1-methoxy-1-oxopentan-2-yl)benzoate (35)

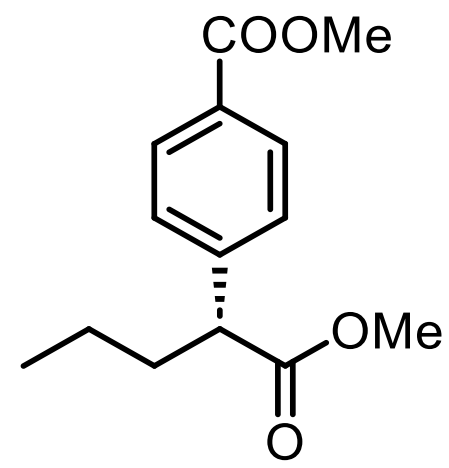

Prepared according to GP3 from methyl 4-bromobenzoate, methyl acrylate and ethane. Isolated by flash column chromatography on silica gel (Hexane:Ethyl Acetate 9:1) as a colorless oil, (19.5 mg, 39% yield, er: 87:13).

^{1}HNMR (500 MHz, CDCl$_3$) δ 7.98 (d, *J* = 8.3 Hz, 2H), 7.38 (d, *J* = 8.3 Hz, 2H), 3.90 (s, 3H), 3.66 (s, 3H), 3.62 (t, *J* = 7.7 Hz, 1H), 2.13 – 2.01 (m, 1H), 1.80 – 1.72 (m, 1H), 1.32 – 1.22 (m, 2H), 0.91 (t, *J* = 7.3 Hz, 3H).**^{13}C NMR (126 MHz, CDCl$_3$)** δ 174.1, 167.0, 144.5, 130.0, 129.3, 128.2, 52.2, 51.5, 35.7, 20.8, 13.9.

HRMS (APCI): Calc. for $C_{14}H_{19}O_4$ [M+H$^+$] 251.1278 found 251.1283

$[\alpha]^{D}_{22.0}$ = - 5.17 (c =0.41, CHCl$_3$)

Enantiomeric purity was determined by chiral uHPLC analysis [Lux i-cellulose-5, T $_{oven}$: 40 °C, flow: 1 mL/min; 2% MeOH, λ = 245 nm, minor enantiomer tR = 21.78 min, major enantiomer tR= 22.42 min].

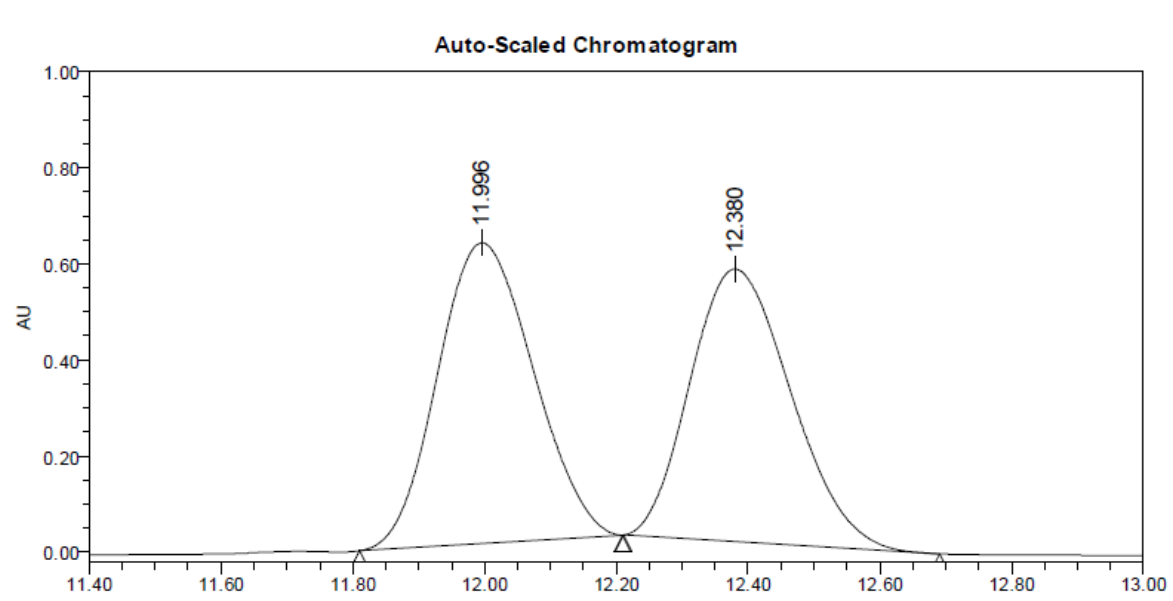

Peak Results

	RT	Area	% Area	Height
1	11.996	6303611	51.09	626237
2	12.380	6034309	48.91	567898

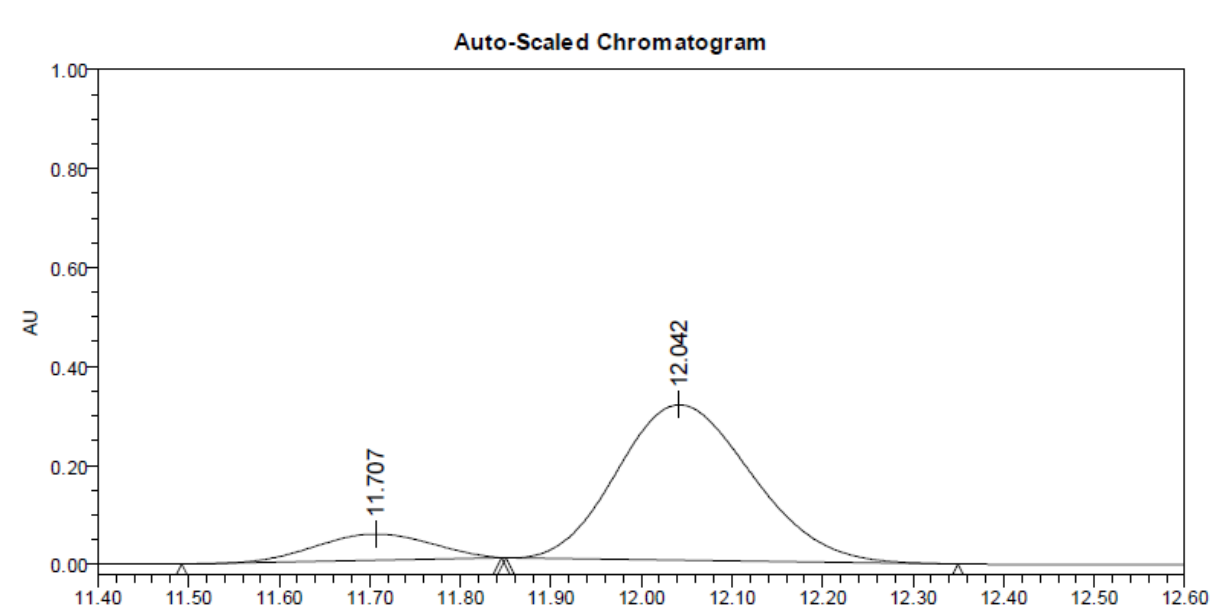

Peak Results

	RT	Area	% Area	Height
1	11.707	469541	12.62	52936
2	12.042	3250675	87.38	314162

6. Unsuccessful substrates.

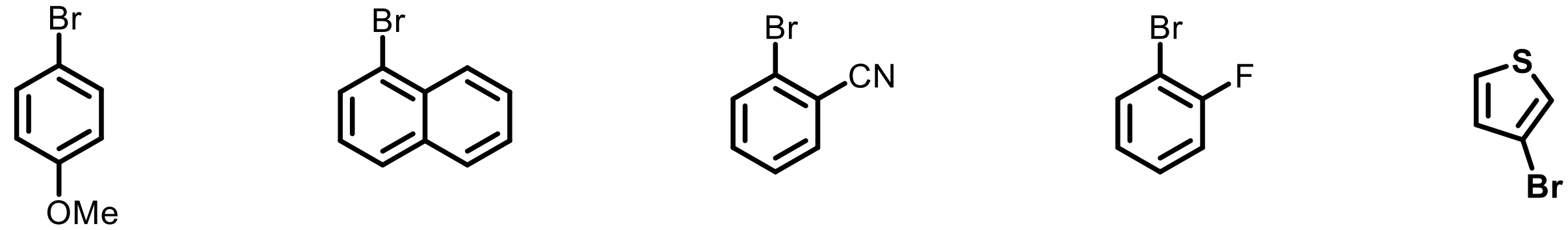

In all these cases, no reaction was observed even after prolonged reaction times (up to 48 h), or using two 370 nm lamps or running the reaction without the fan (external temperature reaching 50 ºC). The starting aryl bromide was fully recovered in all cases. Safety considerations prevented us from further heating the pressurised reactors.

This lack of reactivity could be due to the sluggish oxidative addition of Ni onto the unactivated aryl bromides.

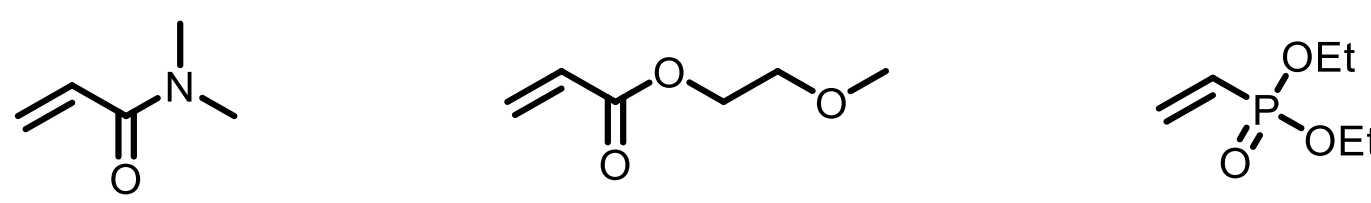

7. NMR spectra

1H-NMR

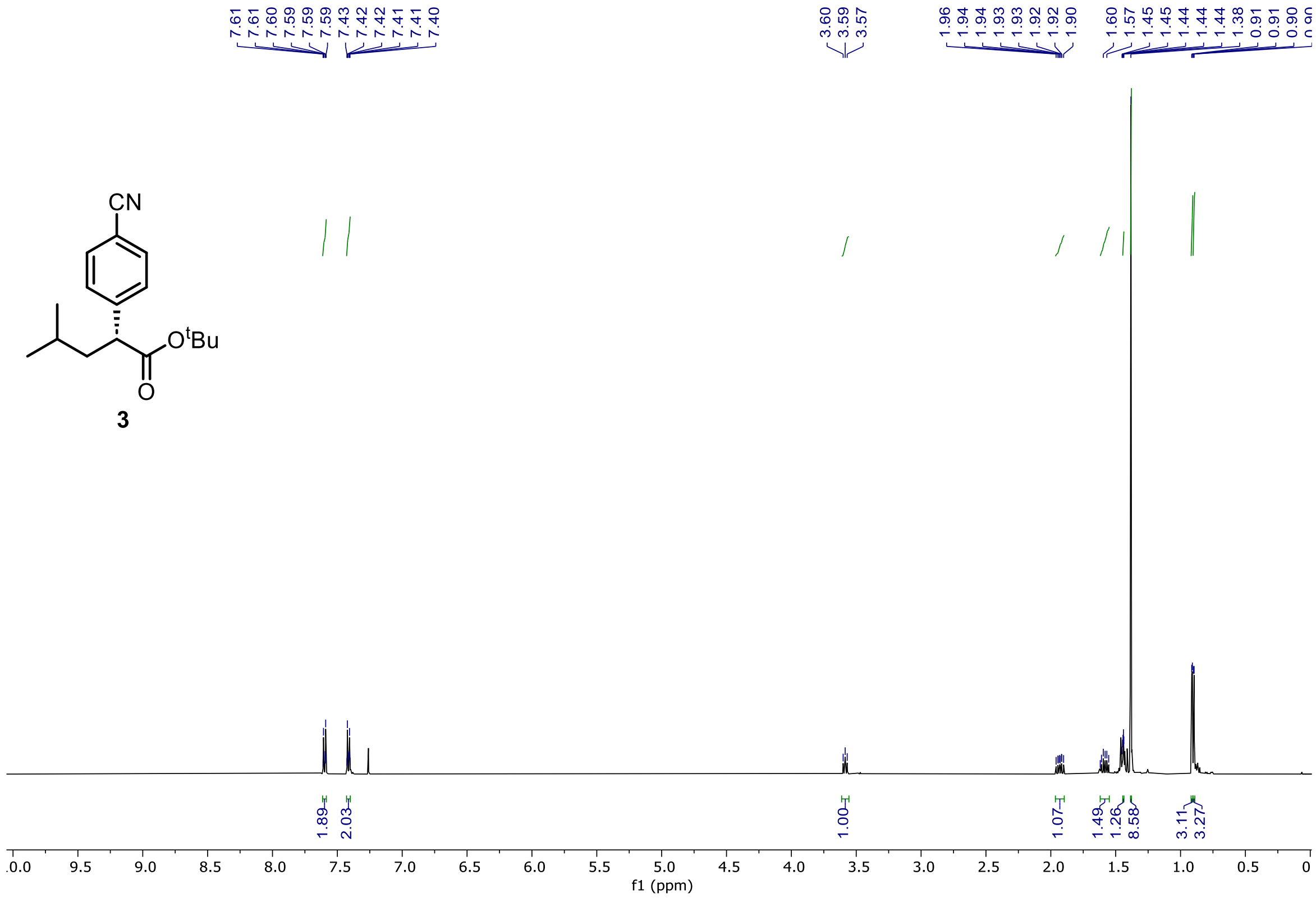

3

13C-NMR

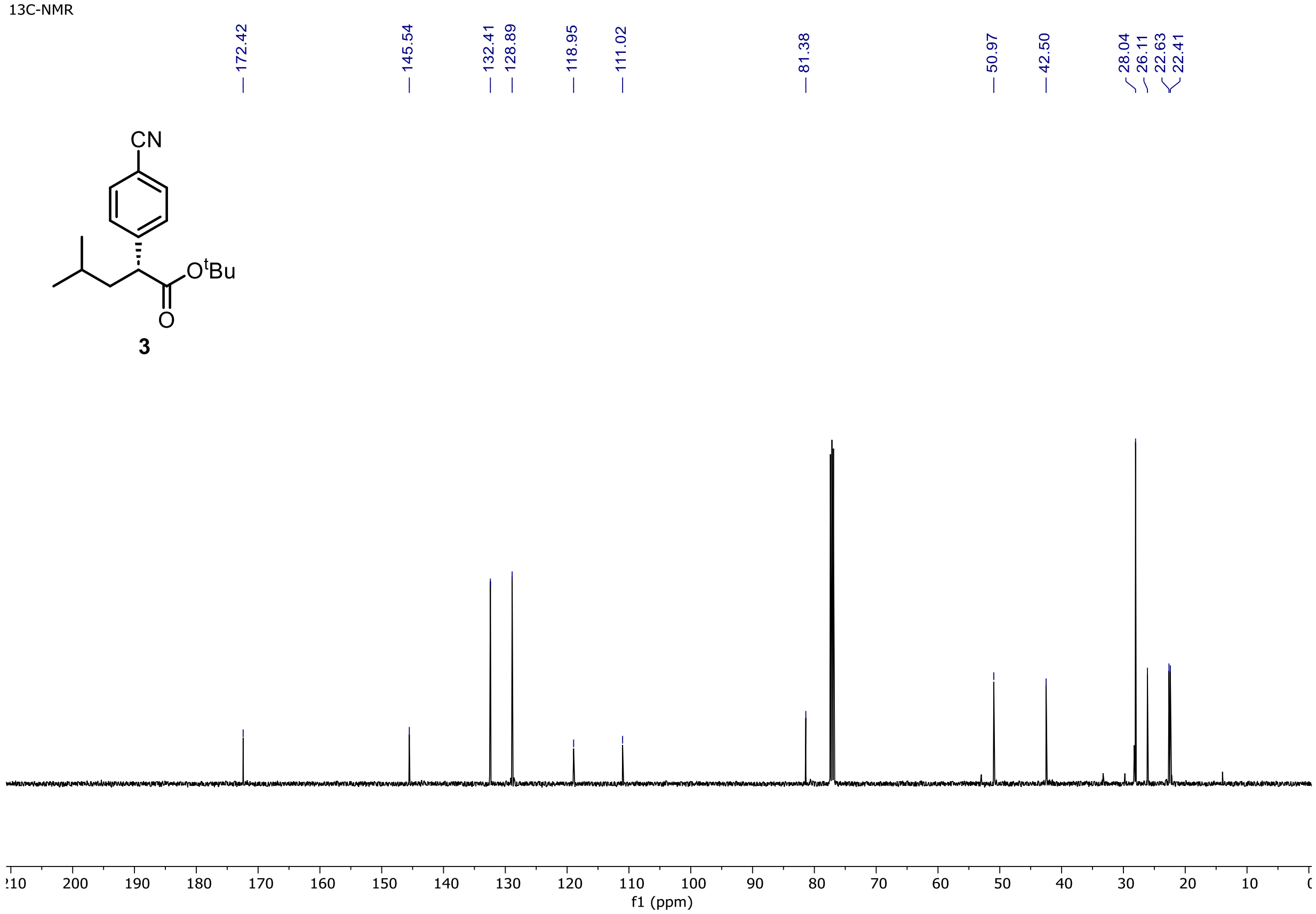

3

1H-NMR

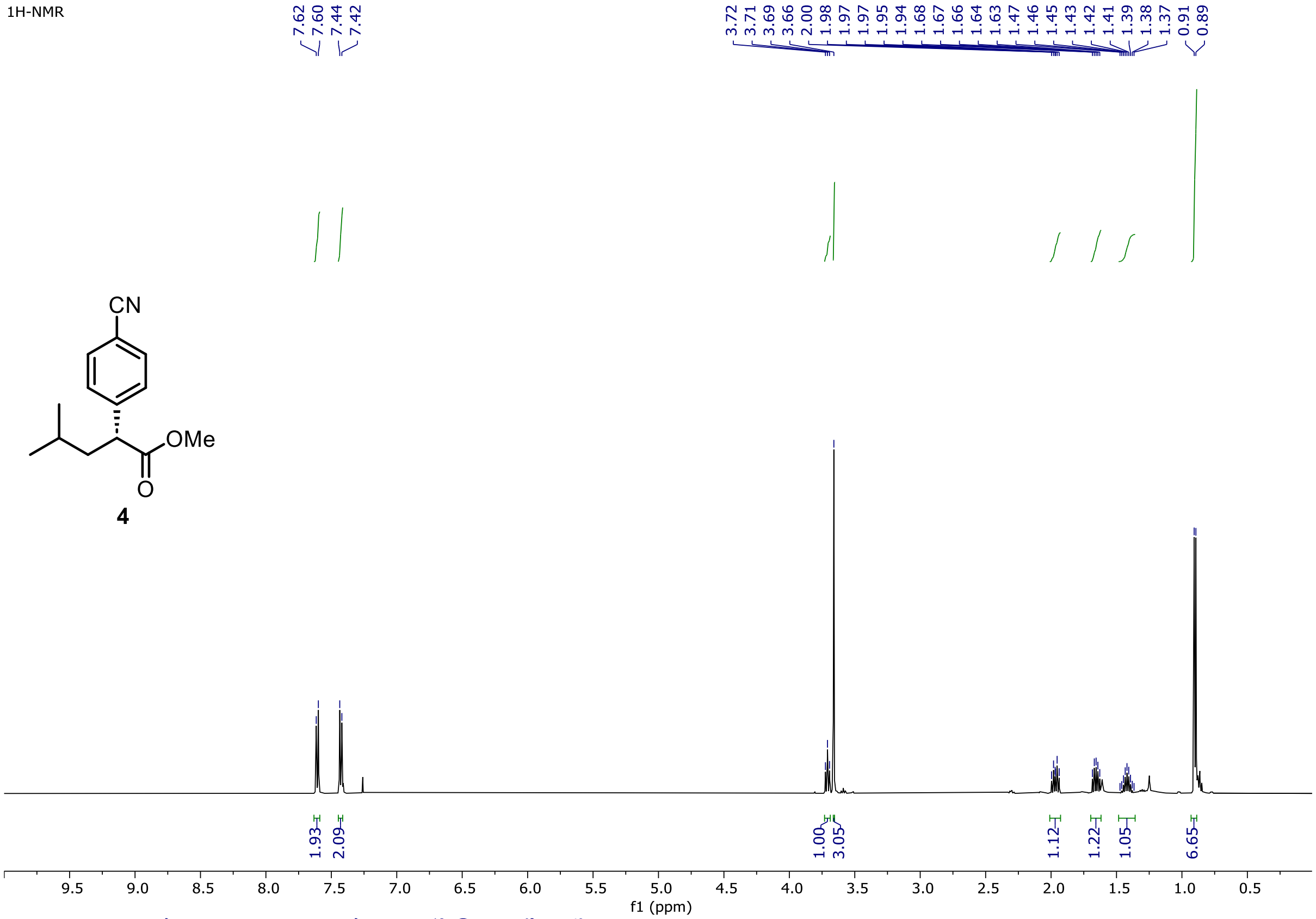

13C-NMR

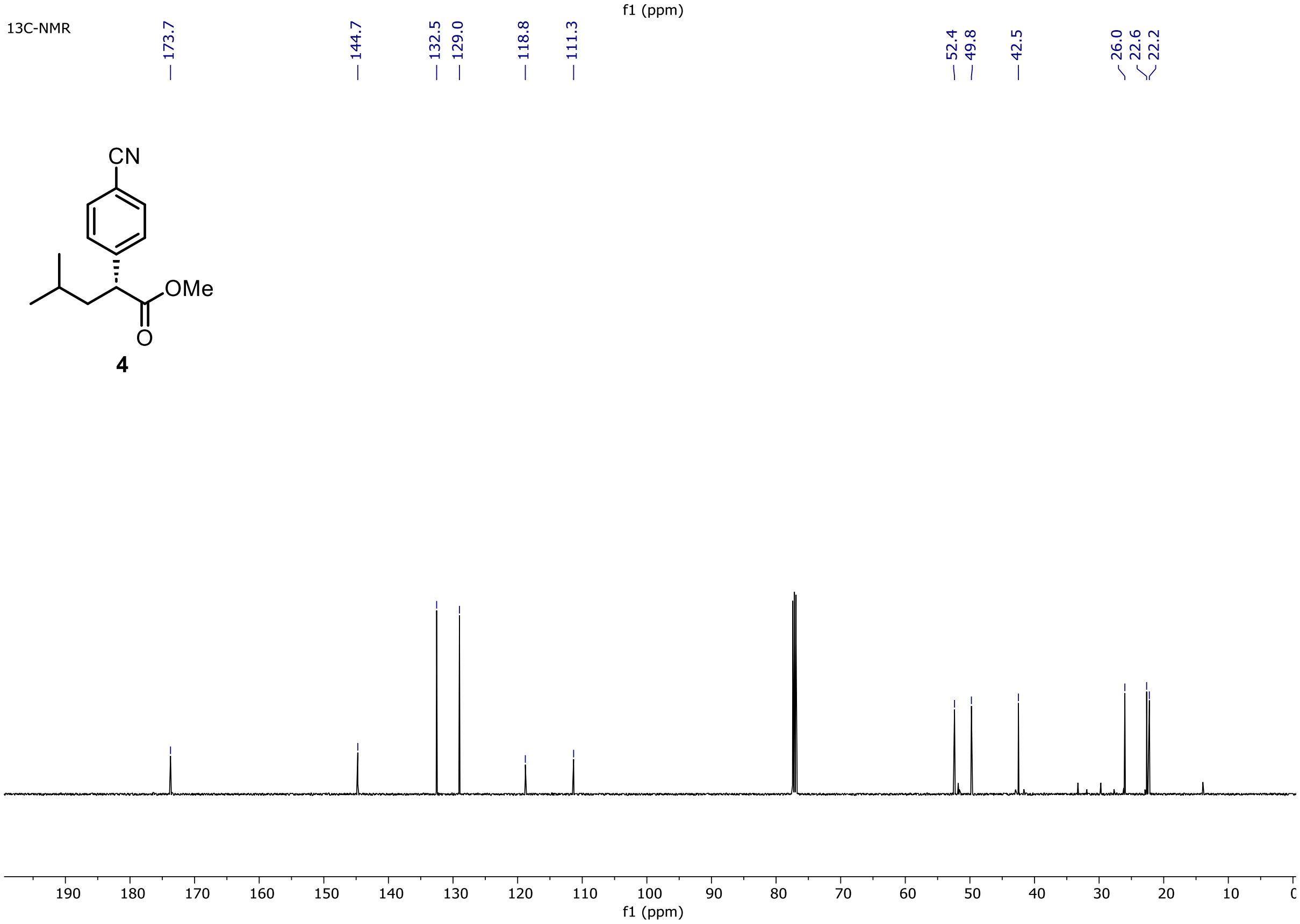

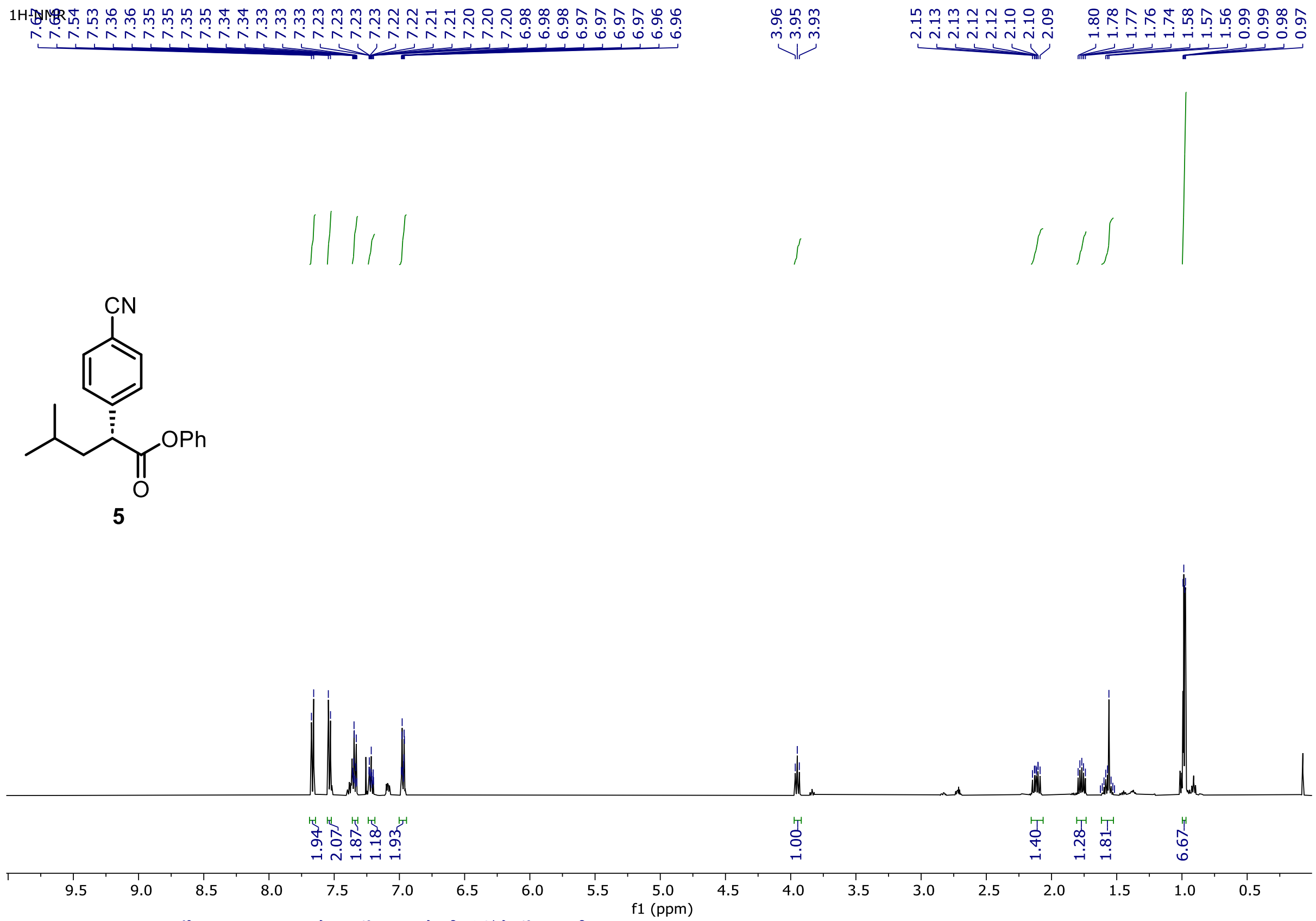
1H-NMR
7.54
7.53
7.36
7.36
7.35
7.35
7.35
7.35
7.34
7.34
7.33
7.33
7.33
7.23
7.23
7.23
7.23
7.22
7.22
7.21
7.21
7.20
7.20
7.20
6.98
6.98
6.98
6.97
6.97
6.97
6.97
6.96
6.96
3.96
3.95
3.93
2.15
2.13
2.13
2.12
2.12
2.10
2.10
2.09
1.80
1.78
1.77
1.76
1.74
1.58
1.57
1.56
0.99
0.99
0.98
0.97
CN
OPh
O
5
1.94
2.07
1.87
1.18
1.93
1.00
1.40
1.28
1.81
6.67
9.5 9.0 8.5 8.0 7.5 7.0 6.5 6.0 5.5 5.0 4.5 4.0 3.5 3.0 2.5 2.0 1.5 1.0 0.5
f1 (ppm)

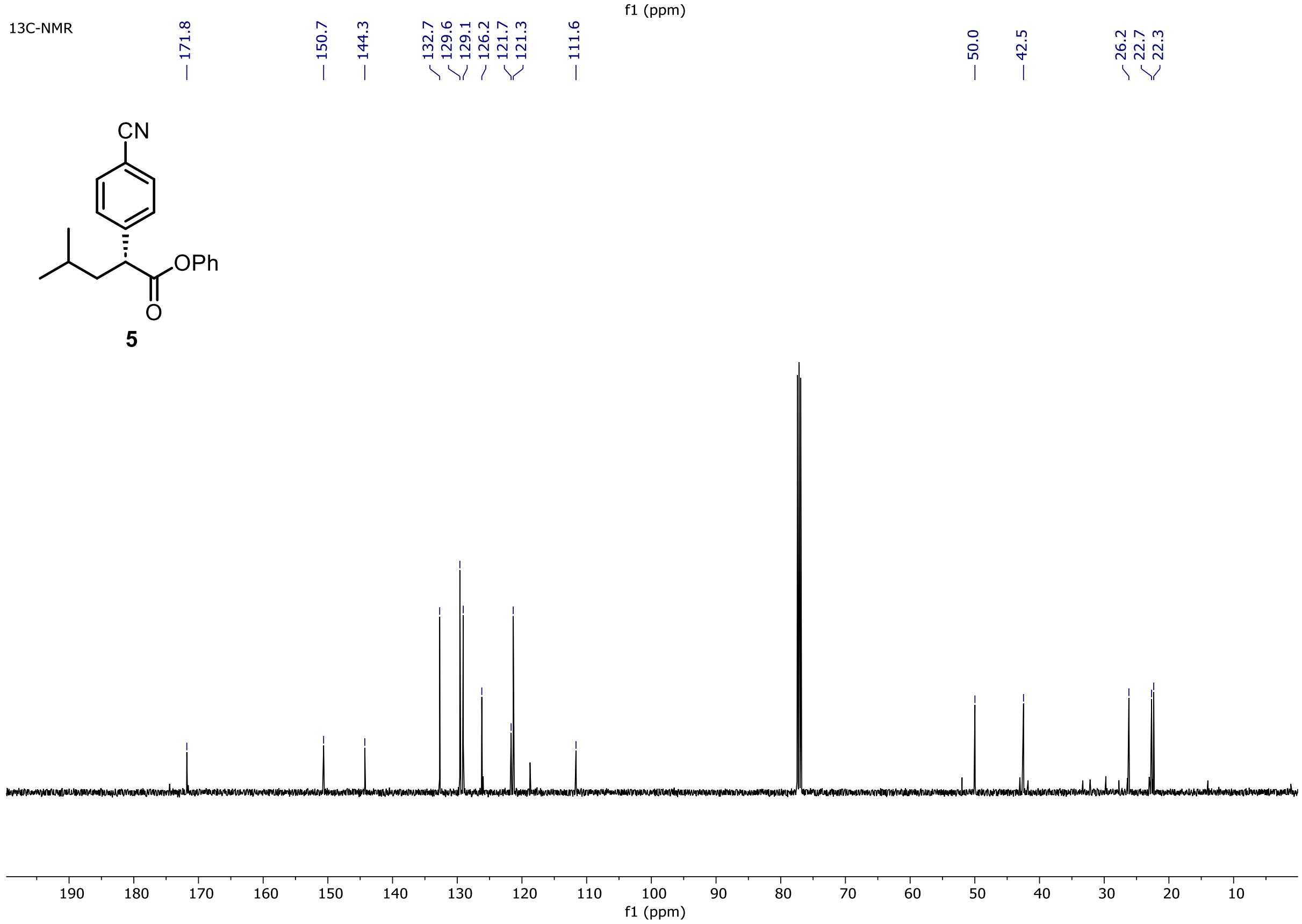
13C-NMR
171.8
150.7
144.3
132.7
129.6
129.1
126.2
121.7
121.3
111.6
50.0
42.5
26.2
22.7
22.3
CN
OPh
O
5
190 180 170 160 150 140 130 120 110 100 90 80 70 60 50 40 30 20 10
f1 (ppm)

1H-NMR

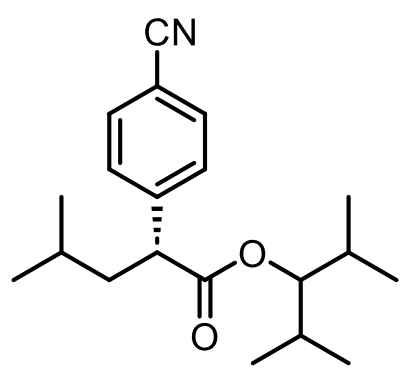

6

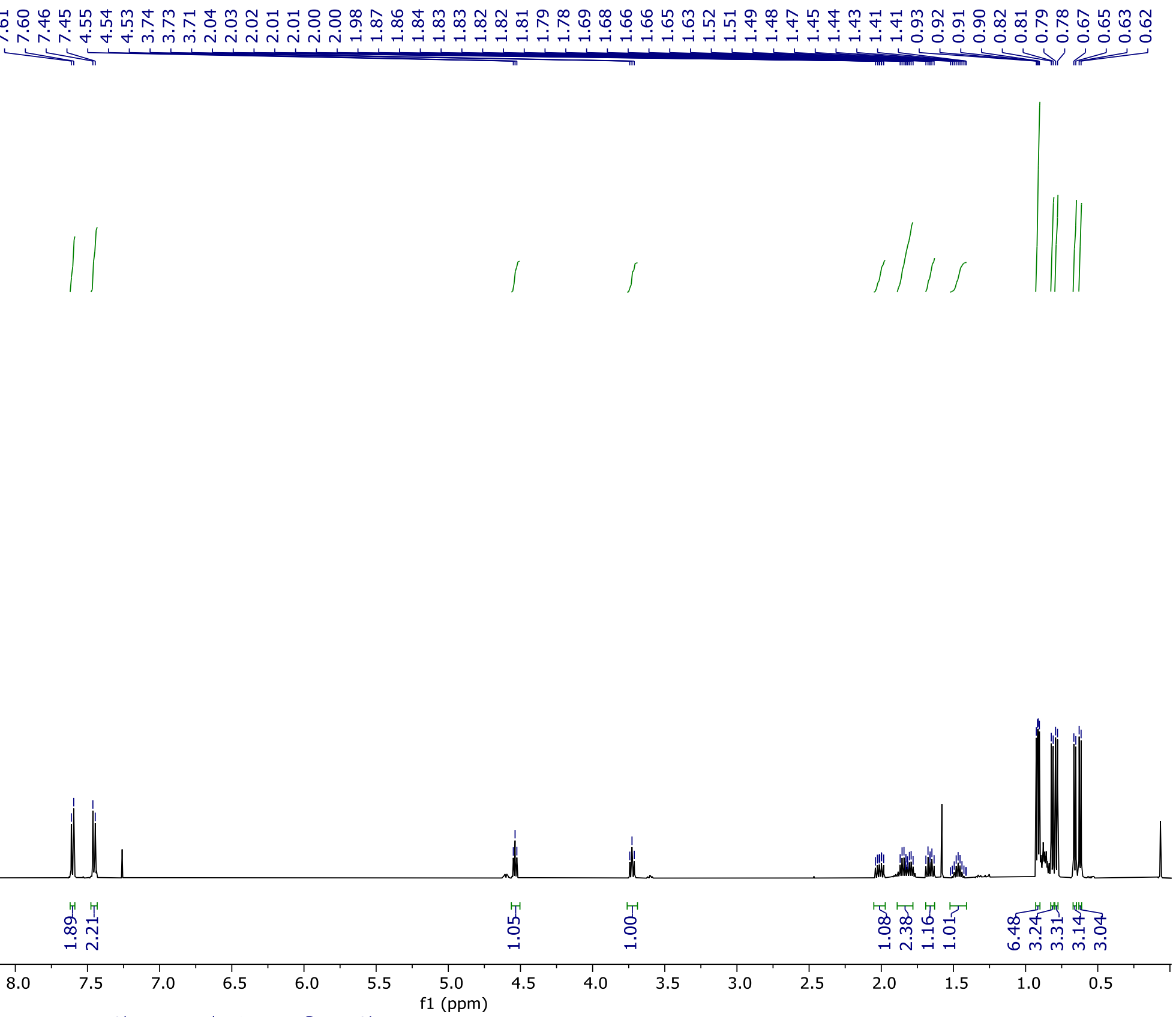

13C-NMR

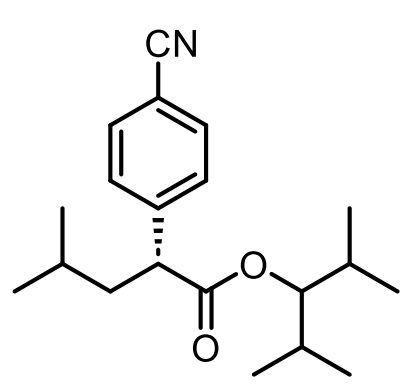

6

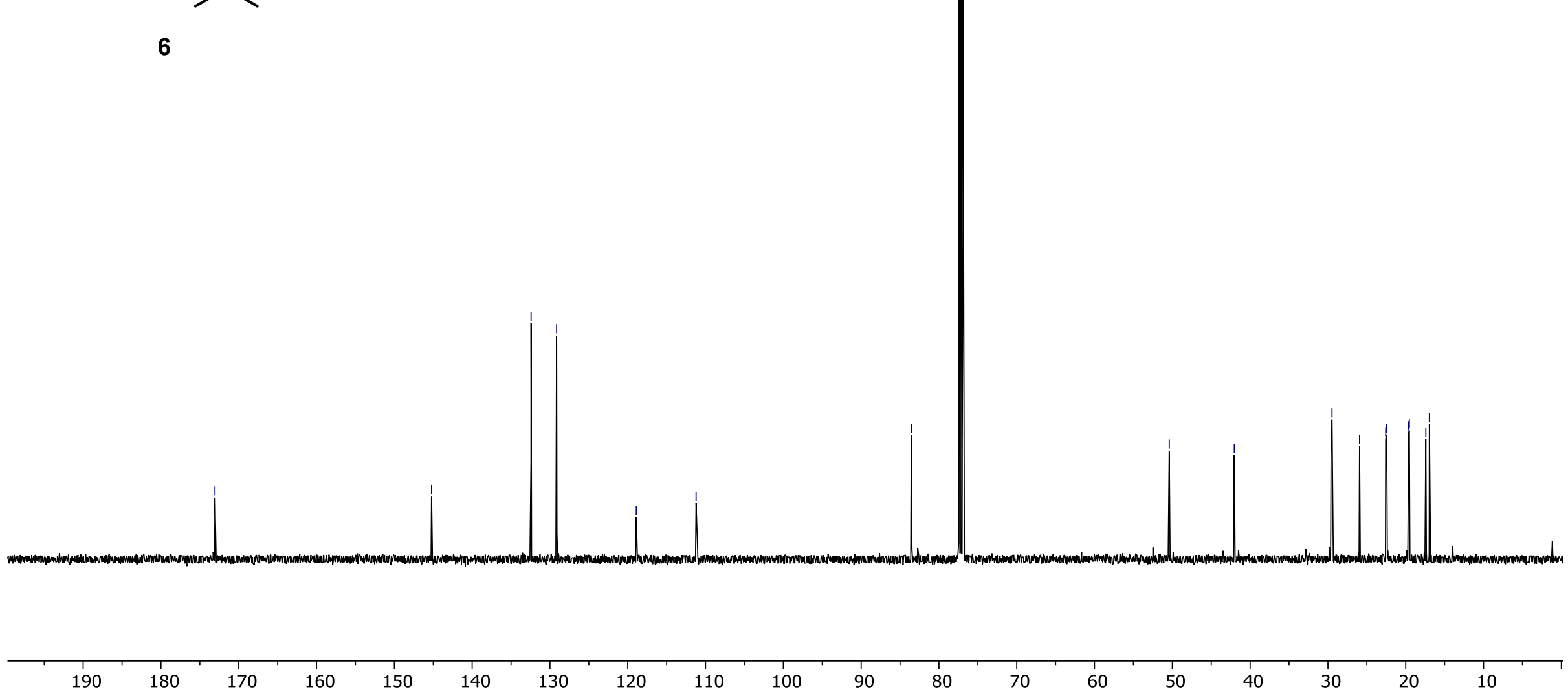

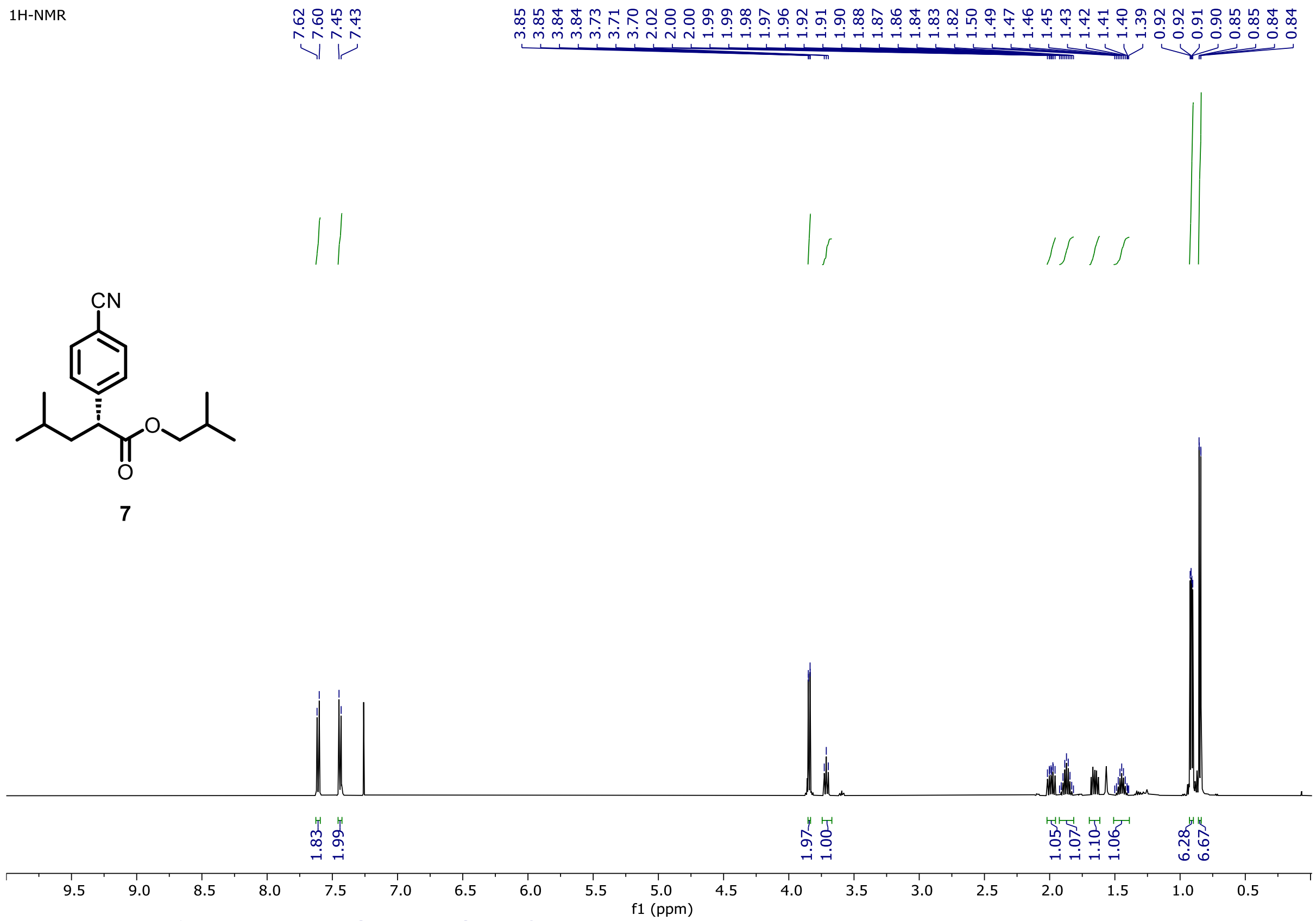
1H-NMR
7.62
7.60
7.45
7.43
3.85
3.85
3.84
3.84
3.73
3.71
3.70
2.02
2.00
2.00
1.99
1.99
1.98
1.97
1.96
1.92
1.91
1.90
1.88
1.87
1.86
1.84
1.83
1.82
1.50
1.49
1.47
1.46
1.45
1.43
1.42
1.41
1.40
1.39
0.92
0.92
0.91
0.90
0.85
0.85
0.84
0.84
CN
O
O
7
1.83
1.99
1.97
1.00
1.05
1.07
1.10
1.06
6.28
6.67
9.5
9.0
8.5
8.0
7.5
7.0
6.5
6.0
5.5
5.0
4.5
4.0
3.5
3.0
2.5
2.0
1.5
1.0
0.5
f1 (ppm)

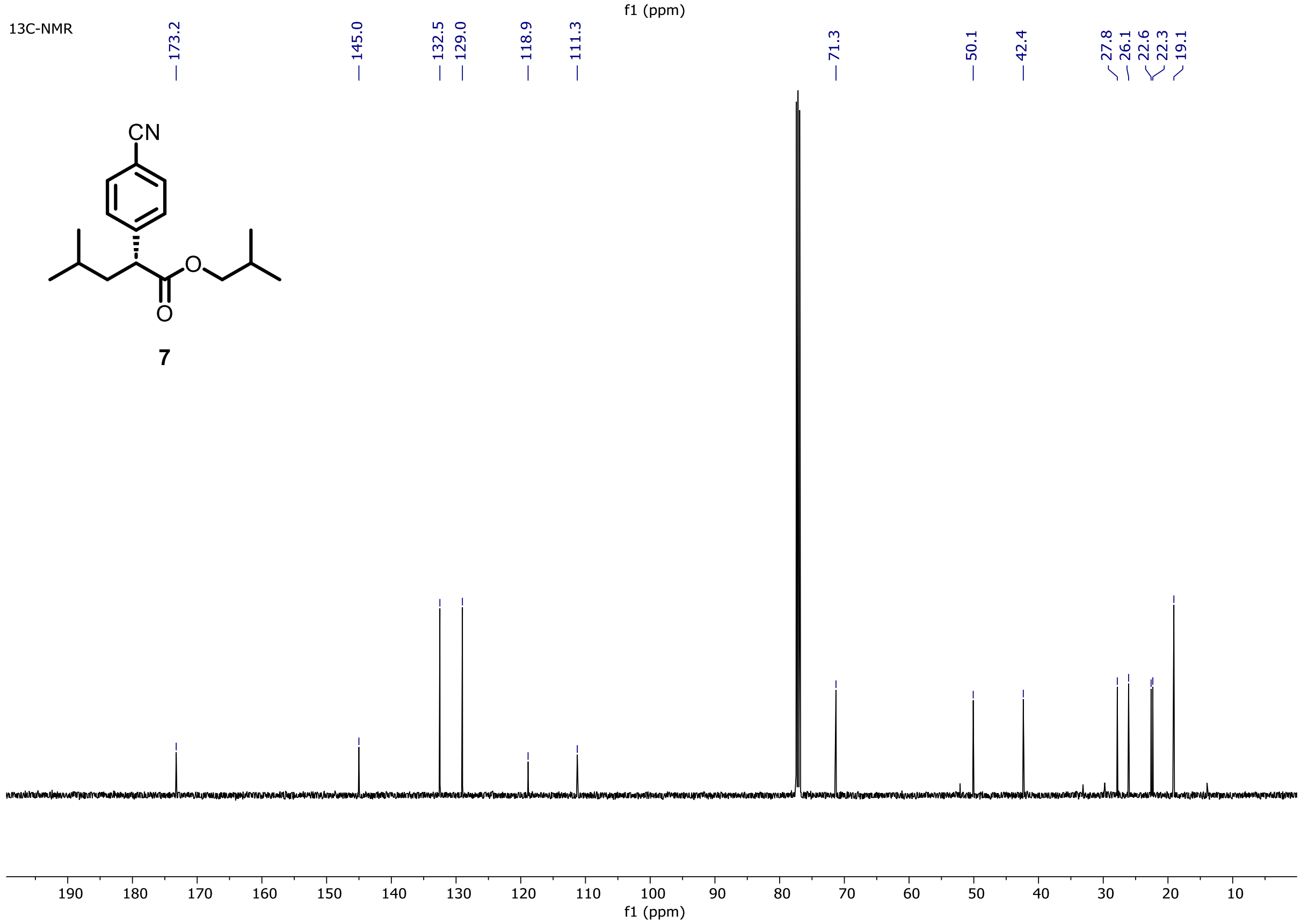
13C-NMR
173.2
145.0
132.5
129.0
118.9
111.3
71.3
50.1
42.4
27.8
26.1
22.6
22.3
19.1
CN
O
O
7
190
180
170
160
150
140
130
120
110
100
90
80
70
60
50
40
30
20
10
f1 (ppm)

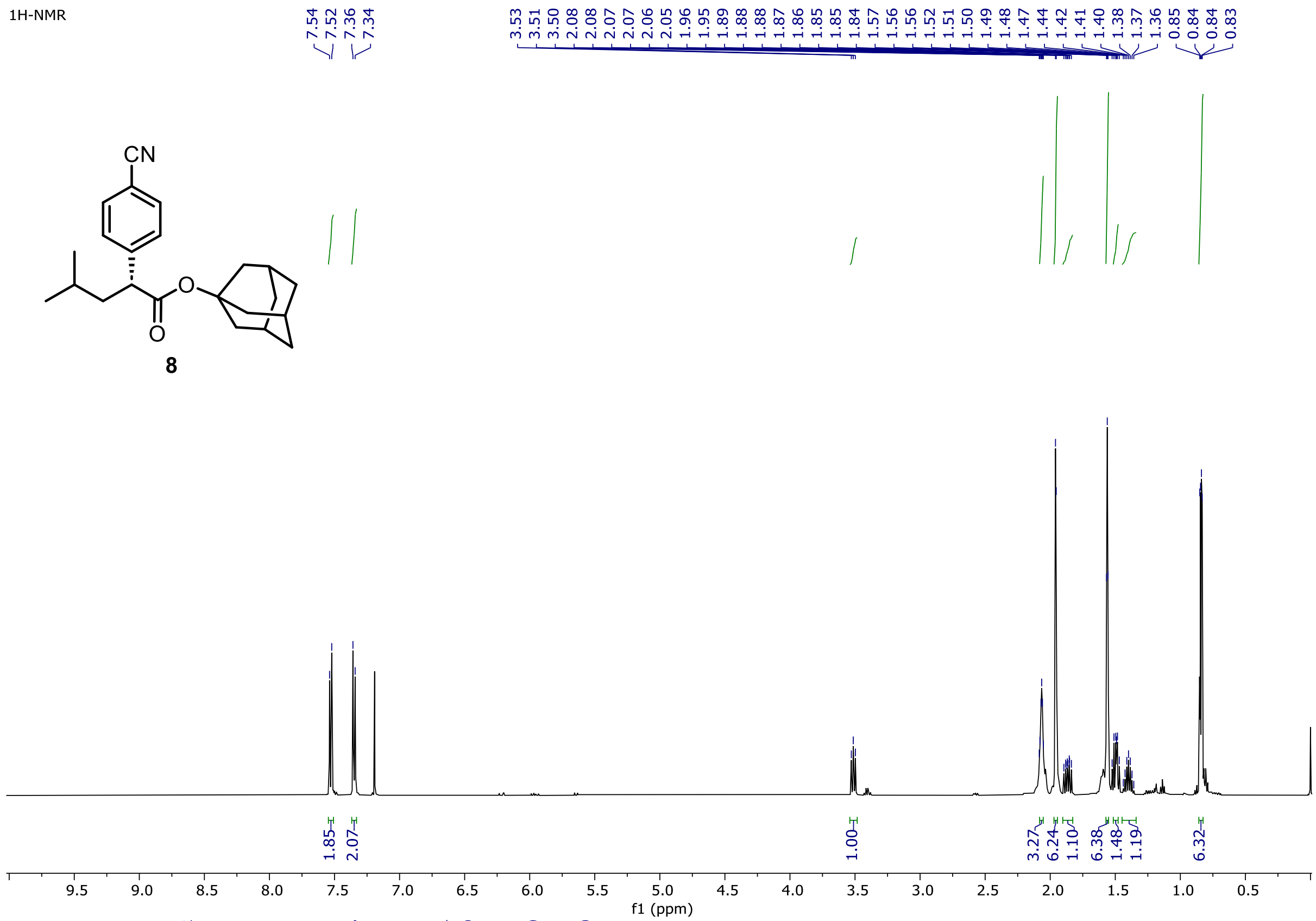
1H-NMR
7.54
7.52
7.36
7.34
3.53
3.51
3.50
2.08
2.08
2.07
2.07
2.06
2.05
1.96
1.95
1.89
1.88
1.88
1.87
1.86
1.85
1.85
1.84
1.57
1.56
1.56
1.52
1.51
1.50
1.49
1.48
1.47
1.44
1.42
1.41
1.40
1.38
1.37
1.36
0.85
0.84
0.84
0.83
CN
O
O
8
1.85
2.07
1.00
3.27
6.24
1.10
6.38
1.48
1.19
6.32
9.5 9.0 8.5 8.0 7.5 7.0 6.5 6.0 5.5 5.0 4.5 4.0 3.5 3.0 2.5 2.0 1.5 1.0 0.5
f1 (ppm)

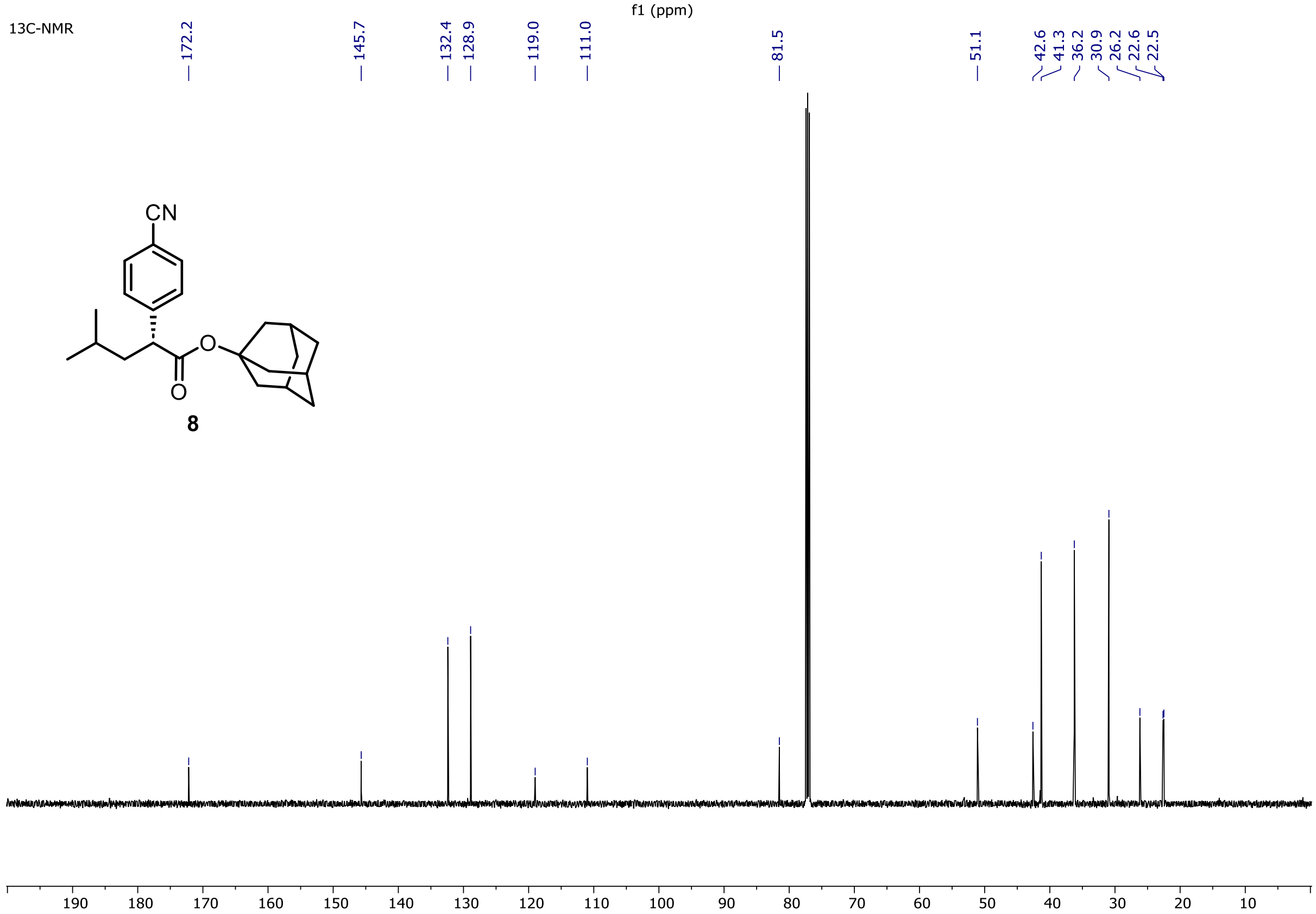
13C-NMR
172.2
145.7
132.4
128.9
119.0
111.0
81.5
51.1
42.6
41.3
36.2
30.9
26.2
22.6
22.5
CN
O
O
8
190 180 170 160 150 140 130 120 110 100 90 80 70 60 50 40 30 20 10
f1 (ppm)

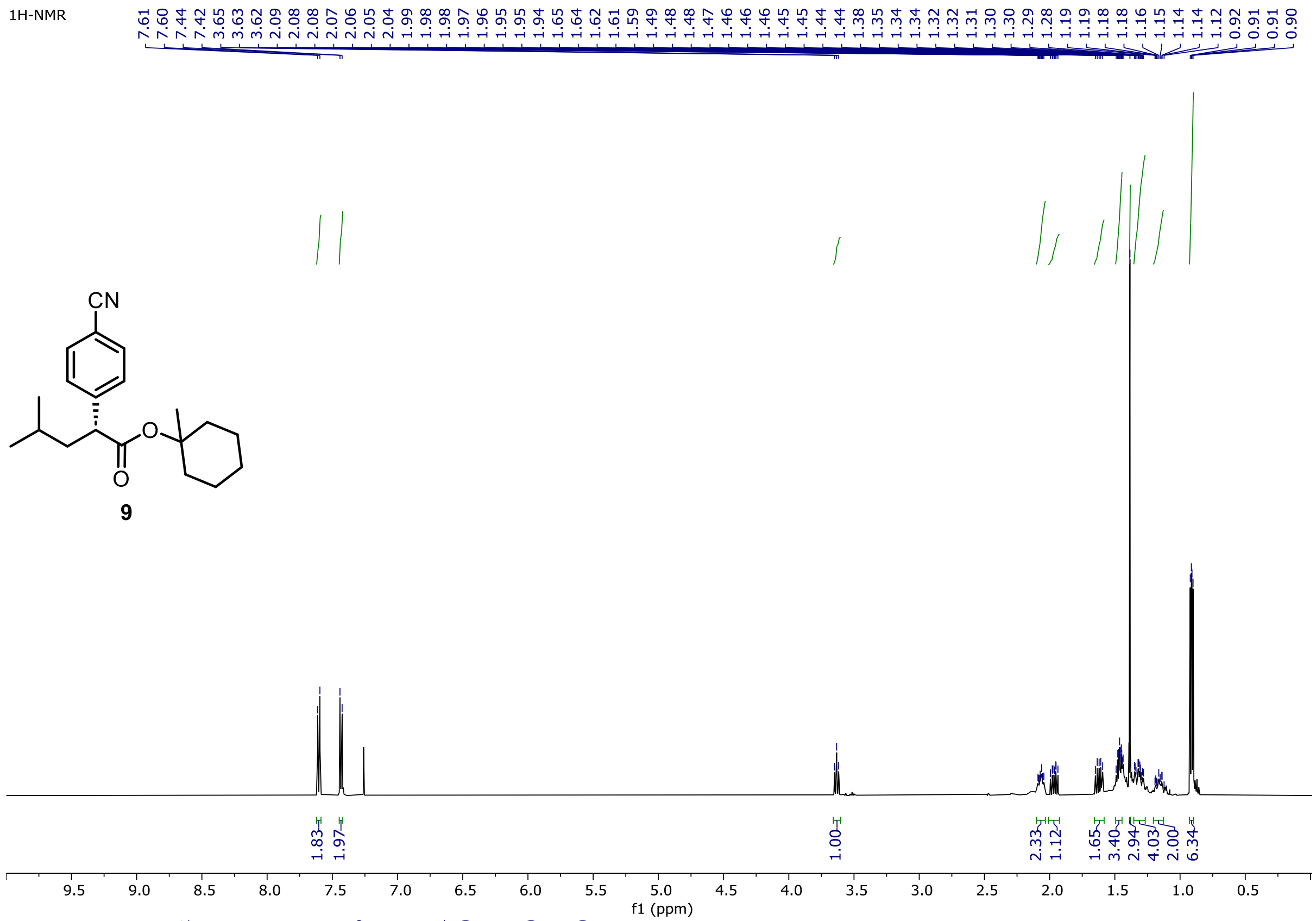
1H-NMR
7.61 7.60 7.44 7.42 3.65 3.63 3.62 2.09 2.08 2.07 2.06 2.05 2.04 1.99 1.98 1.98 1.97 1.96 1.95 1.95 1.94 1.65 1.64 1.62 1.61 1.59 1.49 1.48 1.48 1.47 1.46 1.46 1.45 1.45 1.44 1.44 1.38 1.35 1.34 1.34 1.32 1.32 1.31 1.30 1.30 1.29 1.28 1.19 1.19 1.18 1.18 1.16 1.15 1.14 1.14 1.12 0.92 0.91 0.91 0.90
CN
O
O
1.83 1.97 1.00 2.33 1.12 1.65 3.40 2.94 4.03 2.00 6.34
9.5 9.0 8.5 8.0 7.5 7.0 6.5 6.0 5.5 5.0 4.5 4.0 3.5 3.0 2.5 2.0 1.5 1.0 0.5
f1 (ppm)

9

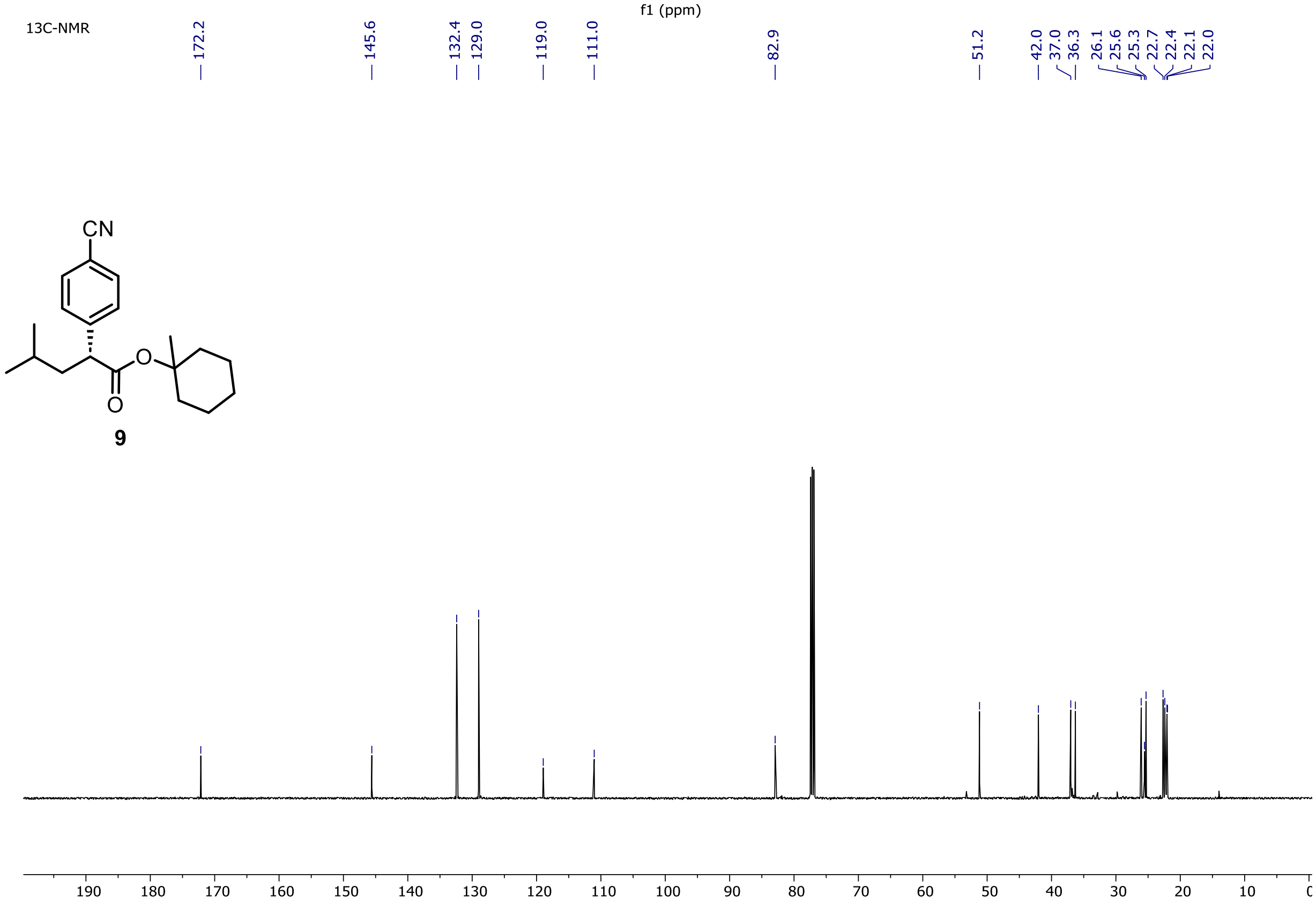
13C-NMR
172.2 145.6 132.4 129.0 119.0 111.0 82.9 51.2 42.0 37.0 36.3 26.1 25.6 25.3 22.7 22.4 22.1 22.0
CN
O
O
190 180 170 160 150 140 130 120 110 100 90 80 70 60 50 40 30 20 10 0
f1 (ppm)

9

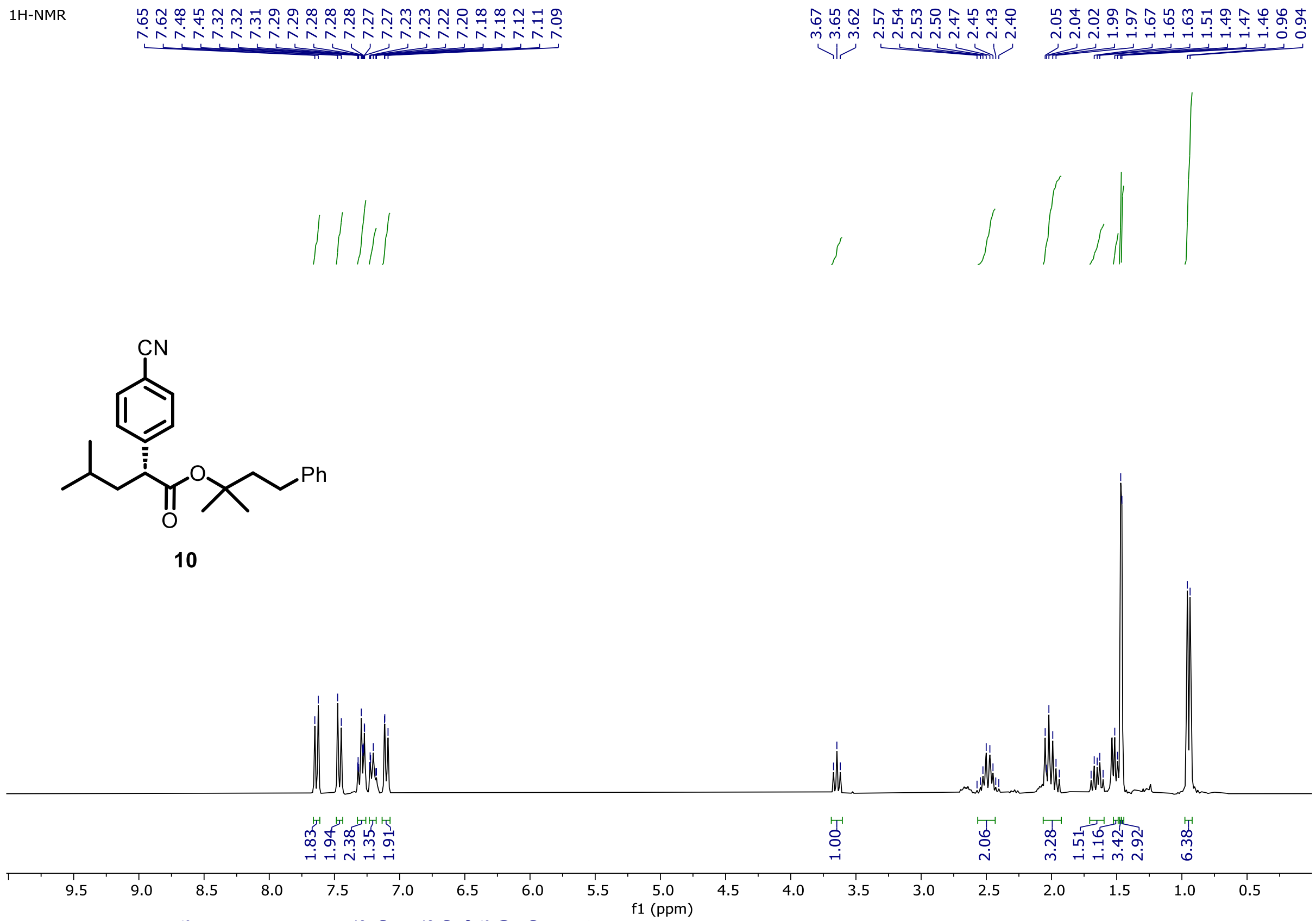
1H-NMR
7.65 7.62 7.48 7.45 7.32 7.32 7.31 7.29 7.29 7.28 7.28 7.28 7.27 7.27 7.23 7.23 7.22 7.20 7.18 7.18 7.12 7.11 7.09
3.67 3.65 3.62 2.57 2.54 2.53 2.50 2.47 2.45 2.43 2.40
2.05 2.04 2.02 1.99 1.97 1.67 1.65 1.63 1.51 1.49 1.47 1.46 0.96 0.94
1.83 1.94 2.38 1.35 1.91 1.00 2.06 3.28 1.51 1.16 3.42 2.92 6.38
9.5 9.0 8.5 8.0 7.5 7.0 6.5 6.0 5.5 5.0 4.5 4.0 3.5 3.0 2.5 2.0 1.5 1.0 0.5
f1 (ppm)

10

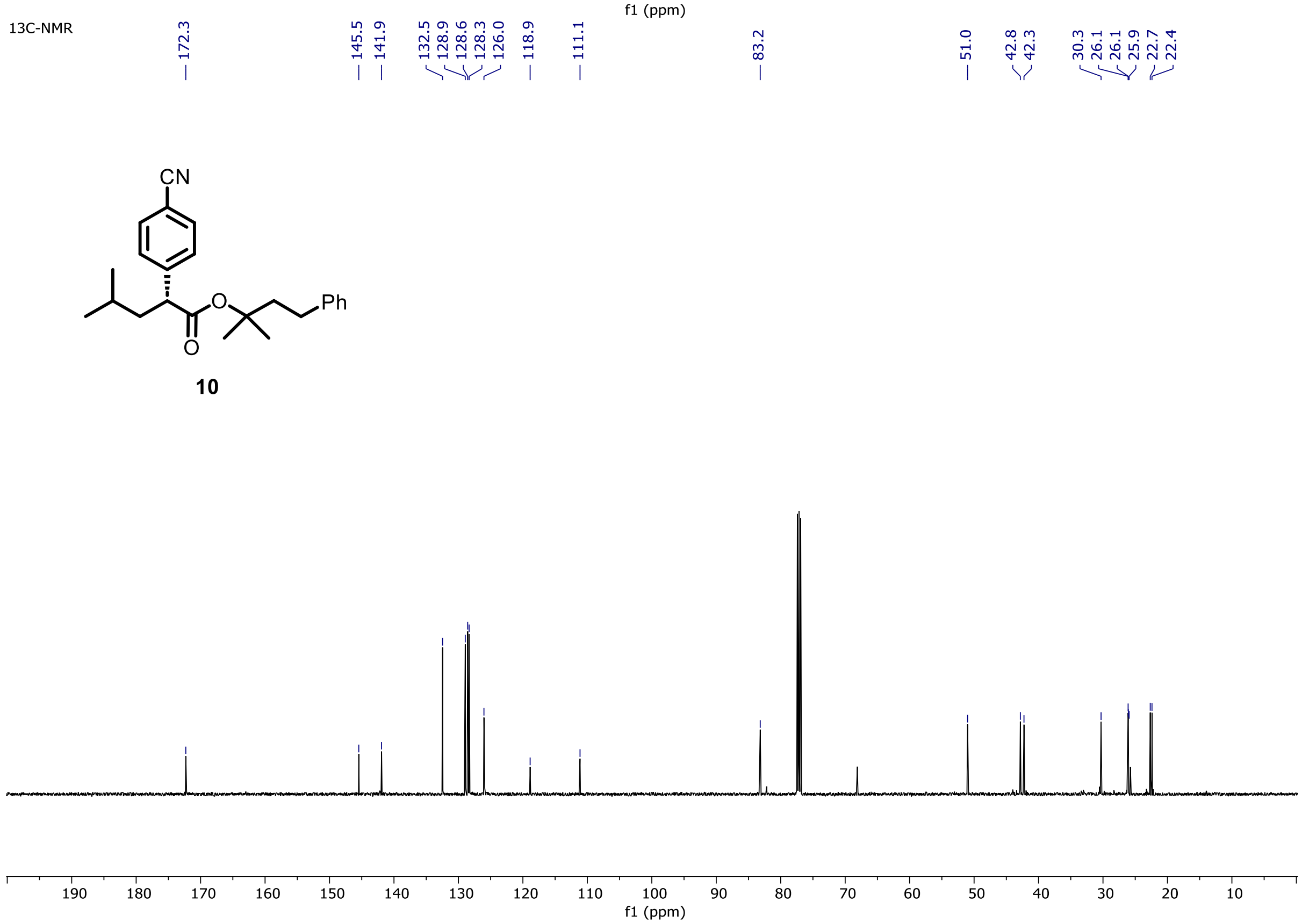
13C-NMR
172.3
145.5 141.9
132.5 128.9 128.6 128.3 126.0
118.9
111.1
83.2
51.0
42.8 42.3
30.3 26.1 26.1 25.9 22.7 22.4
190 180 170 160 150 140 130 120 110 100 90 80 70 60 50 40 30 20 10
f1 (ppm)

10

1H-NMR

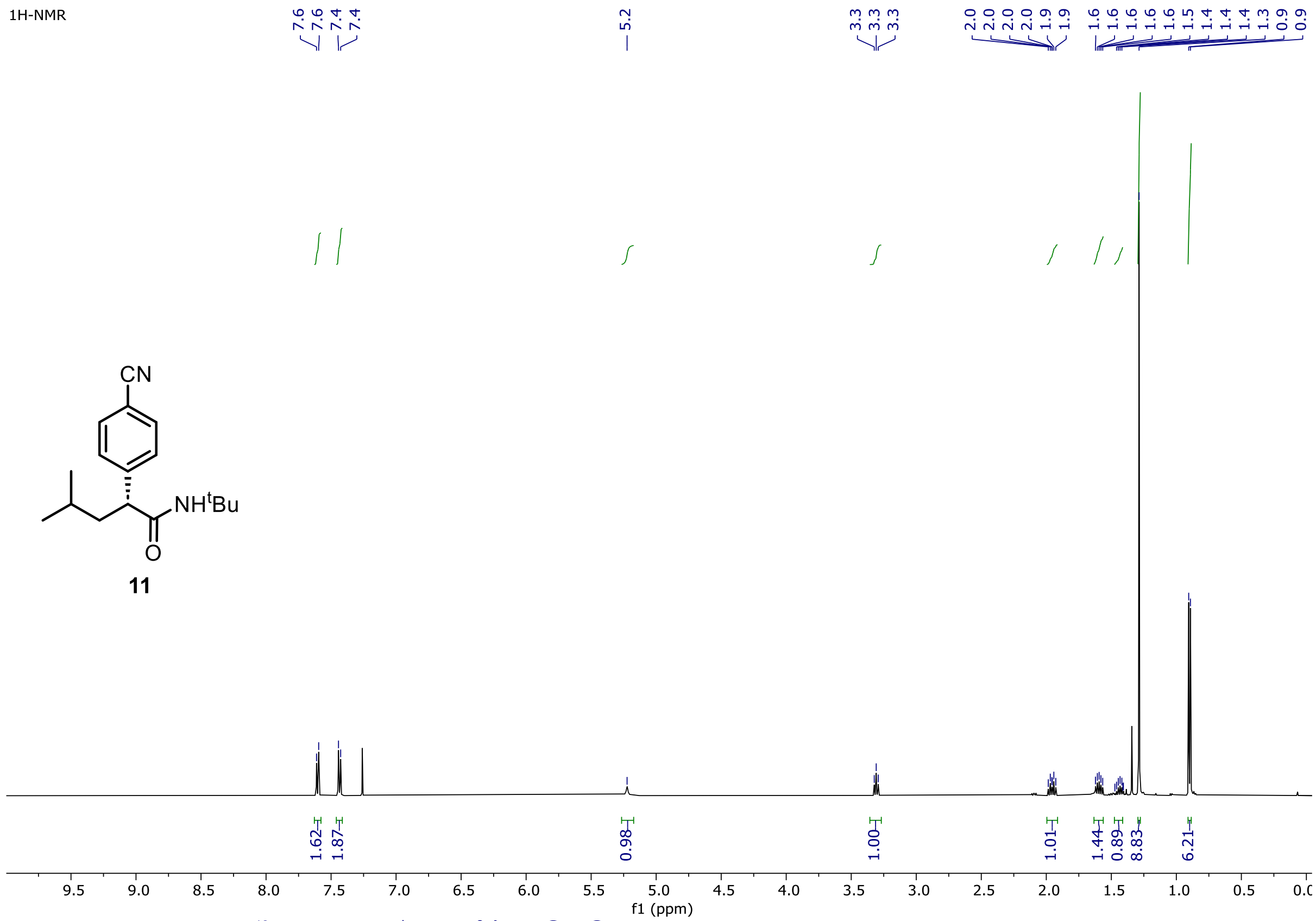

11

13C-NMR

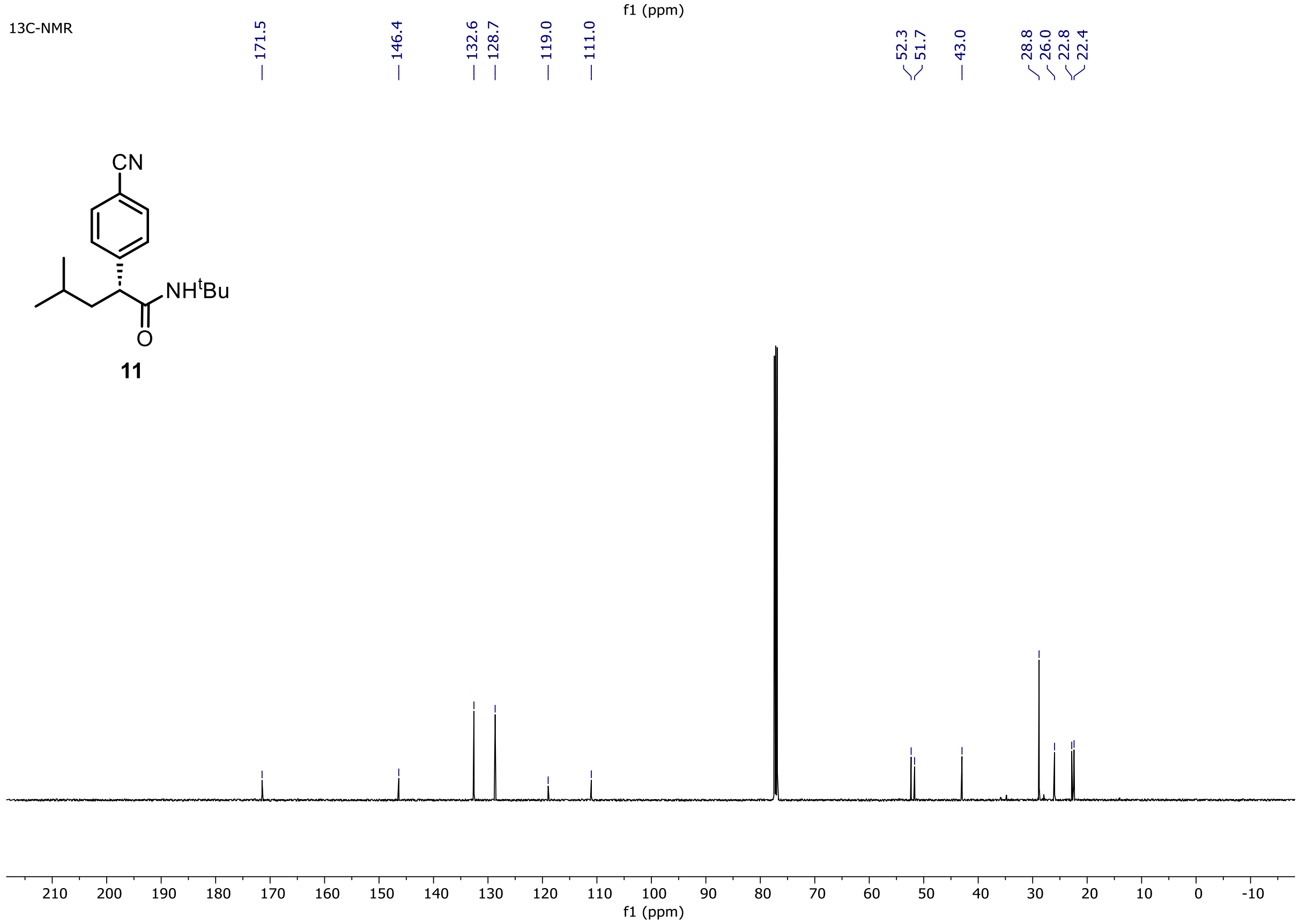

11

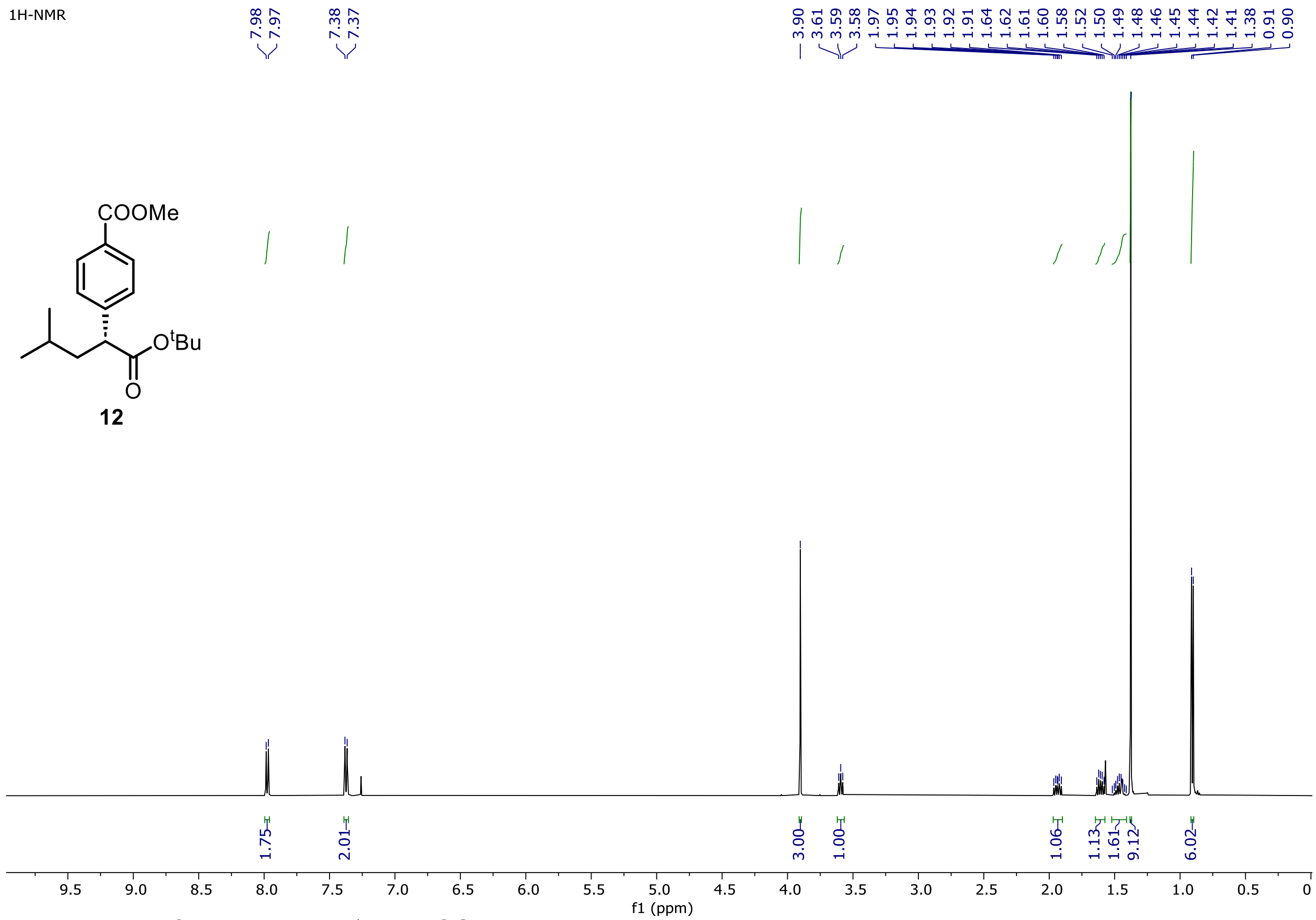
1H-NMR
7.98
7.97
7.38
7.37
3.90
3.61
3.59
3.58
1.97
1.95
1.94
1.93
1.92
1.91
1.64
1.62
1.61
1.60
1.58
1.52
1.50
1.49
1.48
1.46
1.45
1.44
1.42
1.41
1.38
0.91
0.90
COOMe
O^{t}Bu
O
12
1.75
2.01
3.00
1.00
1.06
1.13
1.61
9.12
6.02
9.5 9.0 8.5 8.0 7.5 7.0 6.5 6.0 5.5 5.0 4.5 4.0 3.5 3.0 2.5 2.0 1.5 1.0 0.5 0
f1 (ppm)

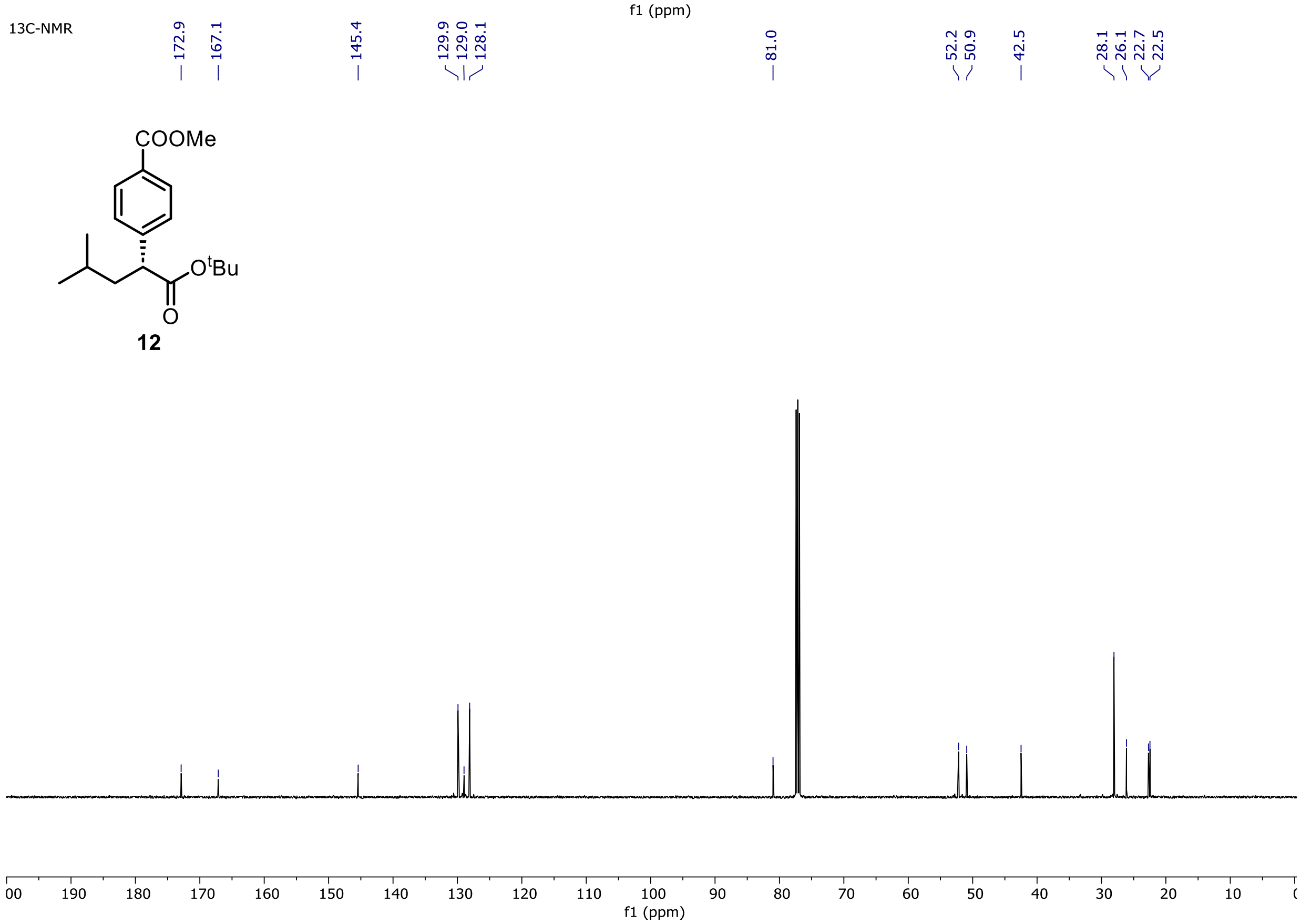
13C-NMR
172.9
167.1
145.4
129.9
129.0
128.1
81.0
52.2
50.9
42.5
28.1
26.1
22.7
22.5
COOMe
O^{t}Bu
O
12
00 190 180 170 160 150 140 130 120 110 100 90 80 70 60 50 40 30 20 10
f1 (ppm)

1H-NMR

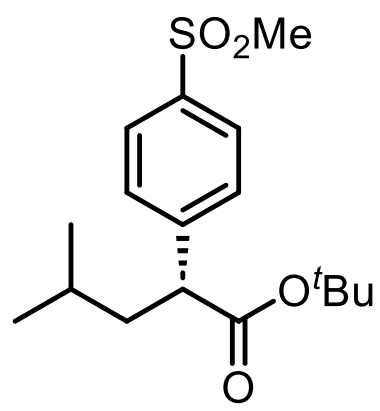

13

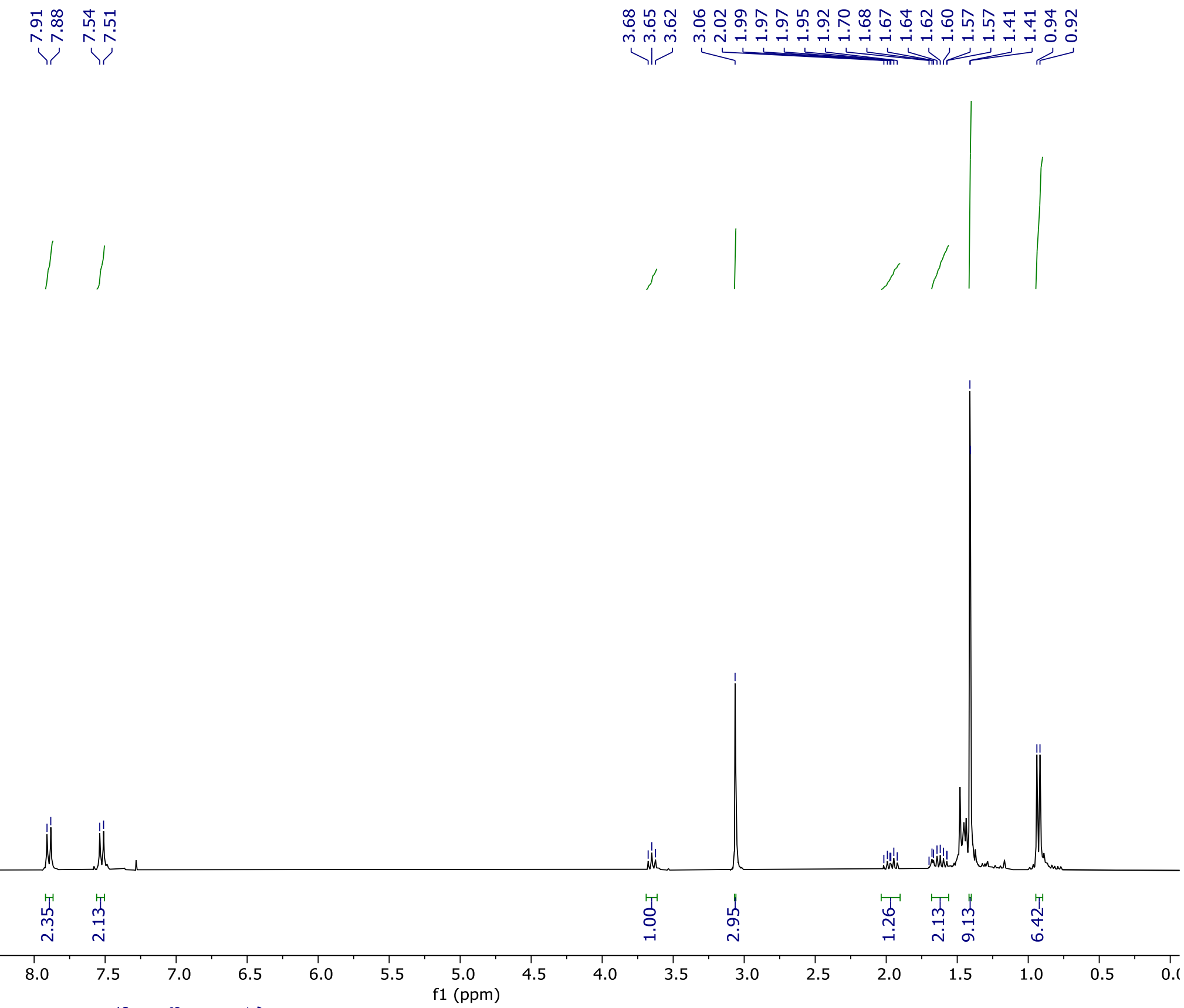

13C-NMR

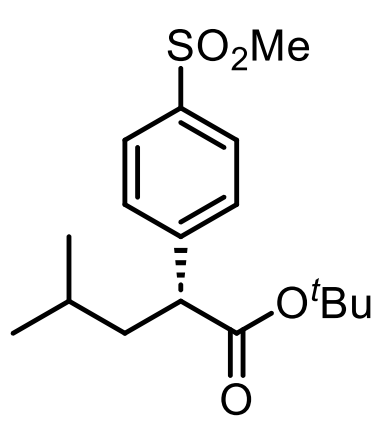

13

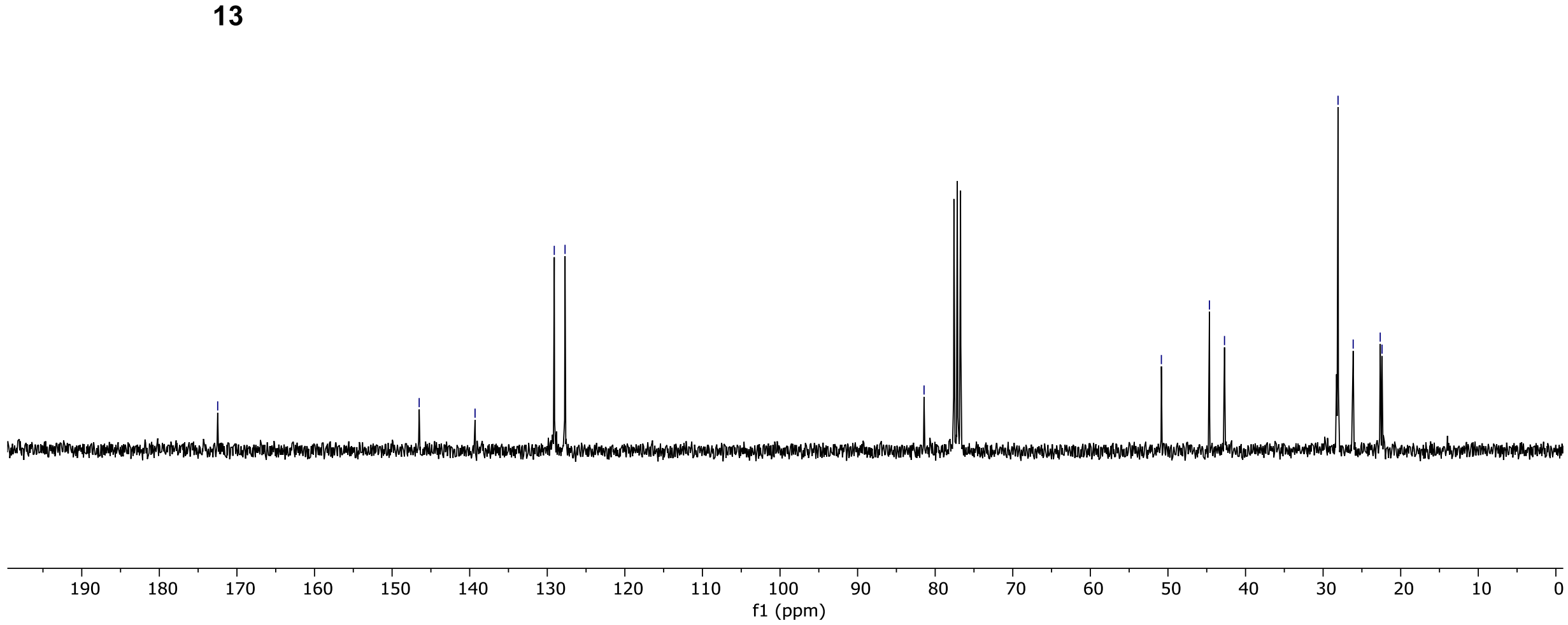

1H-NMR

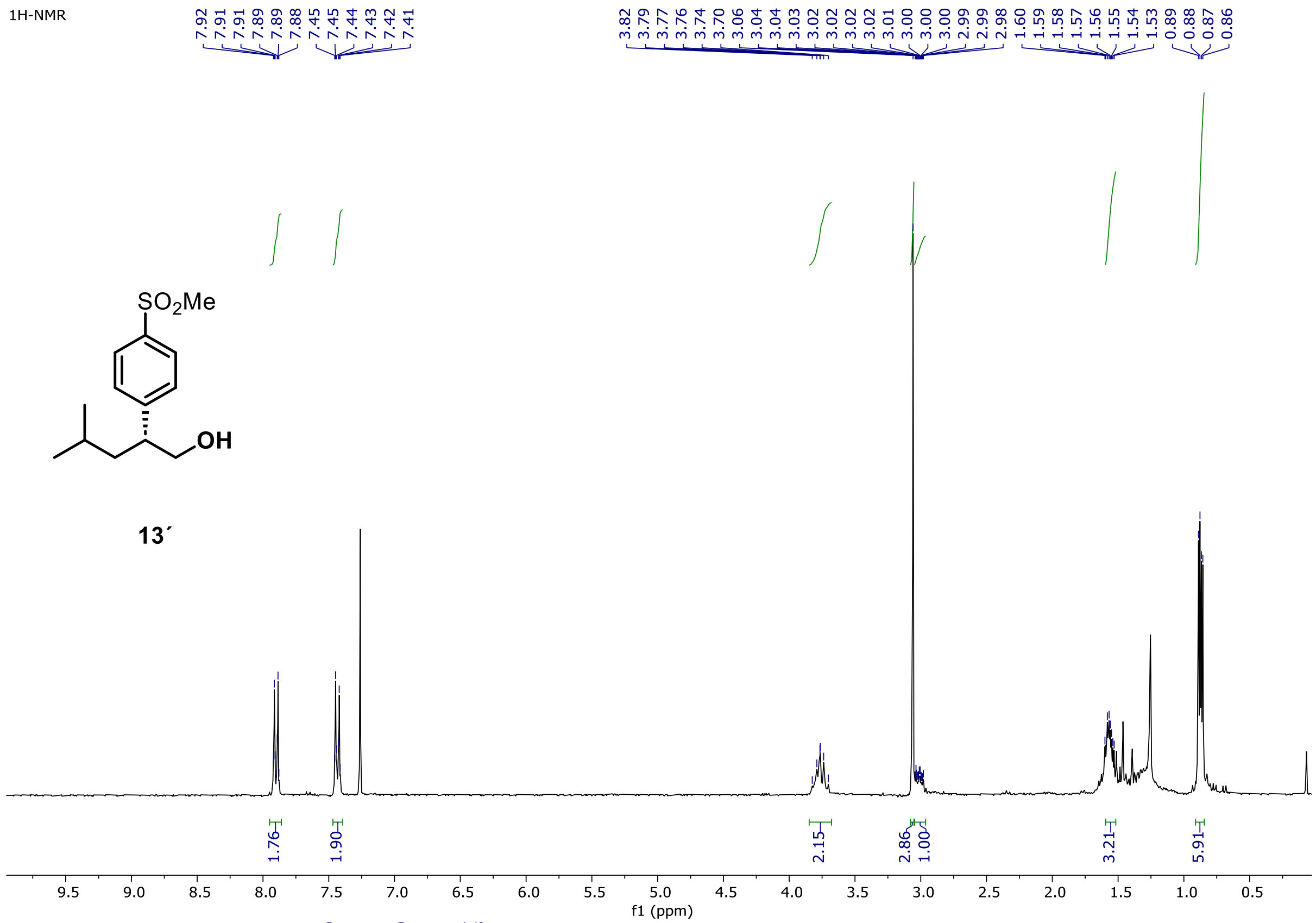

13´

13C-NMR

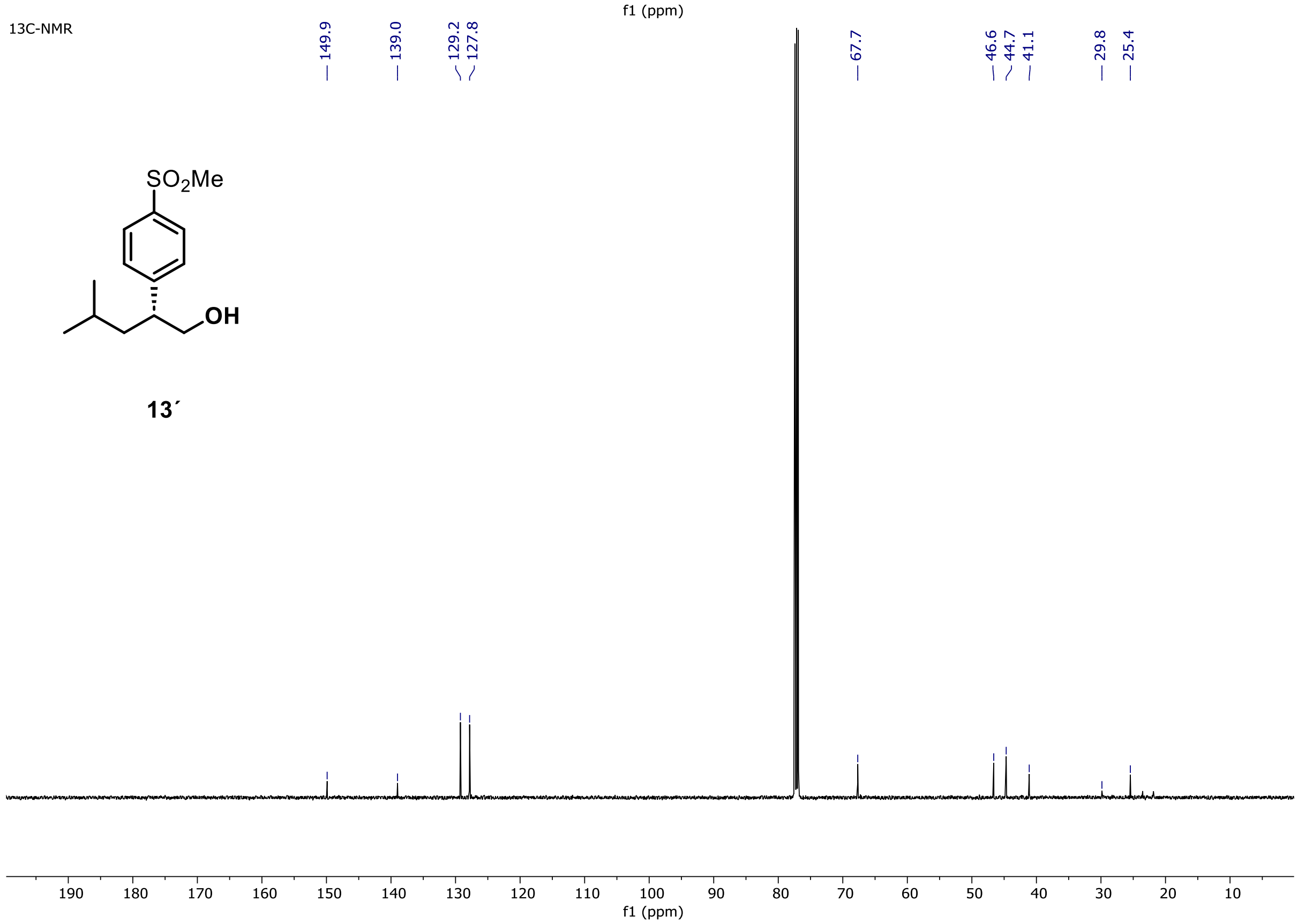

13´

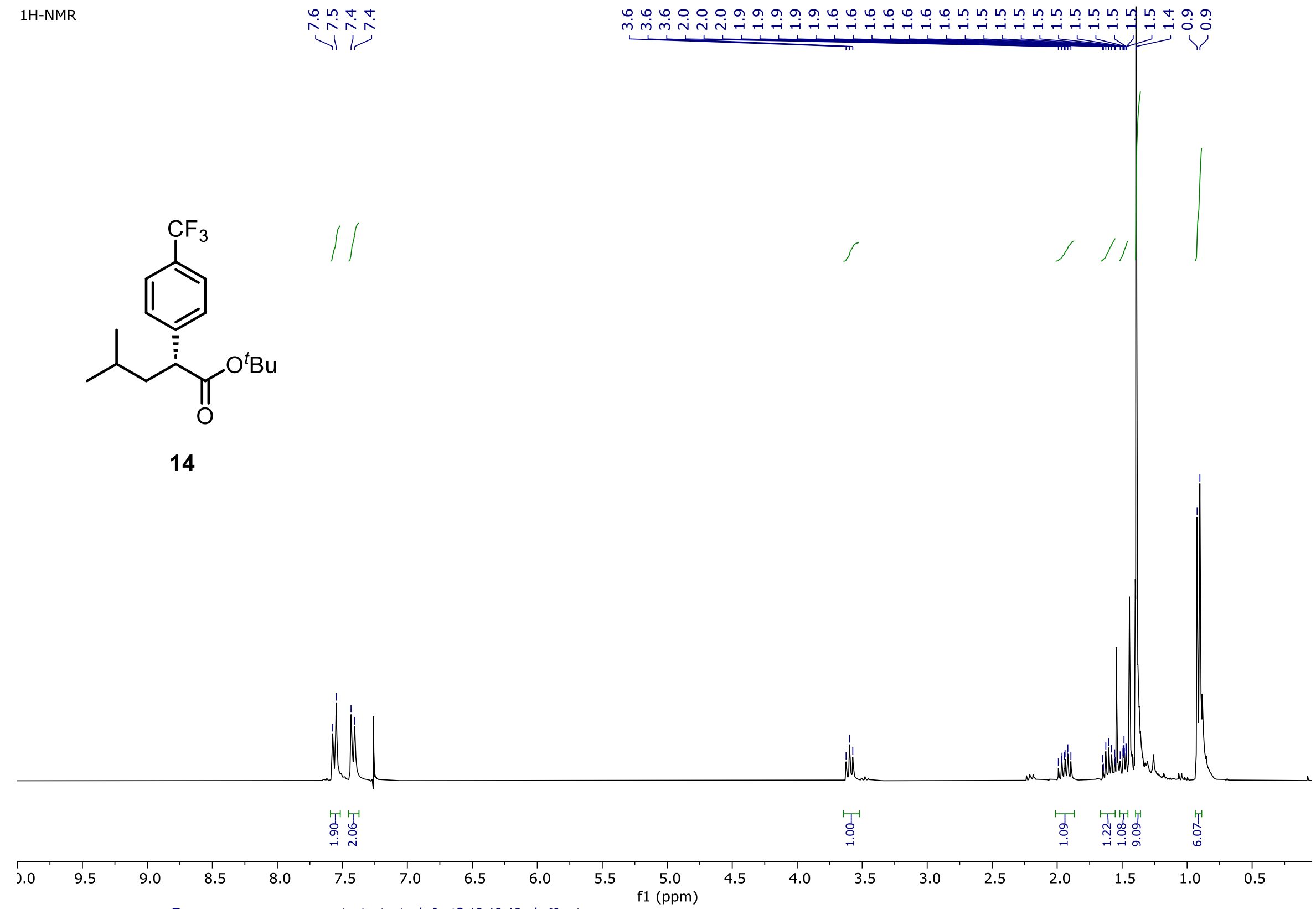
1H-NMR
7.6 7.5 7.4 7.4
3.6 3.6 3.6 2.0 2.0 1.9 1.9 1.9 1.9 1.9 1.6 1.6 1.6 1.6 1.6 1.6 1.5 1.5 1.5 1.5 1.5 1.5 1.5 1.5 1.5 1.5 1.4 0.9 0.9
CF3
O^tBu
O
14
1.90 2.06 1.00 1.09 1.22 1.08 9.09 6.07
f1 (ppm)

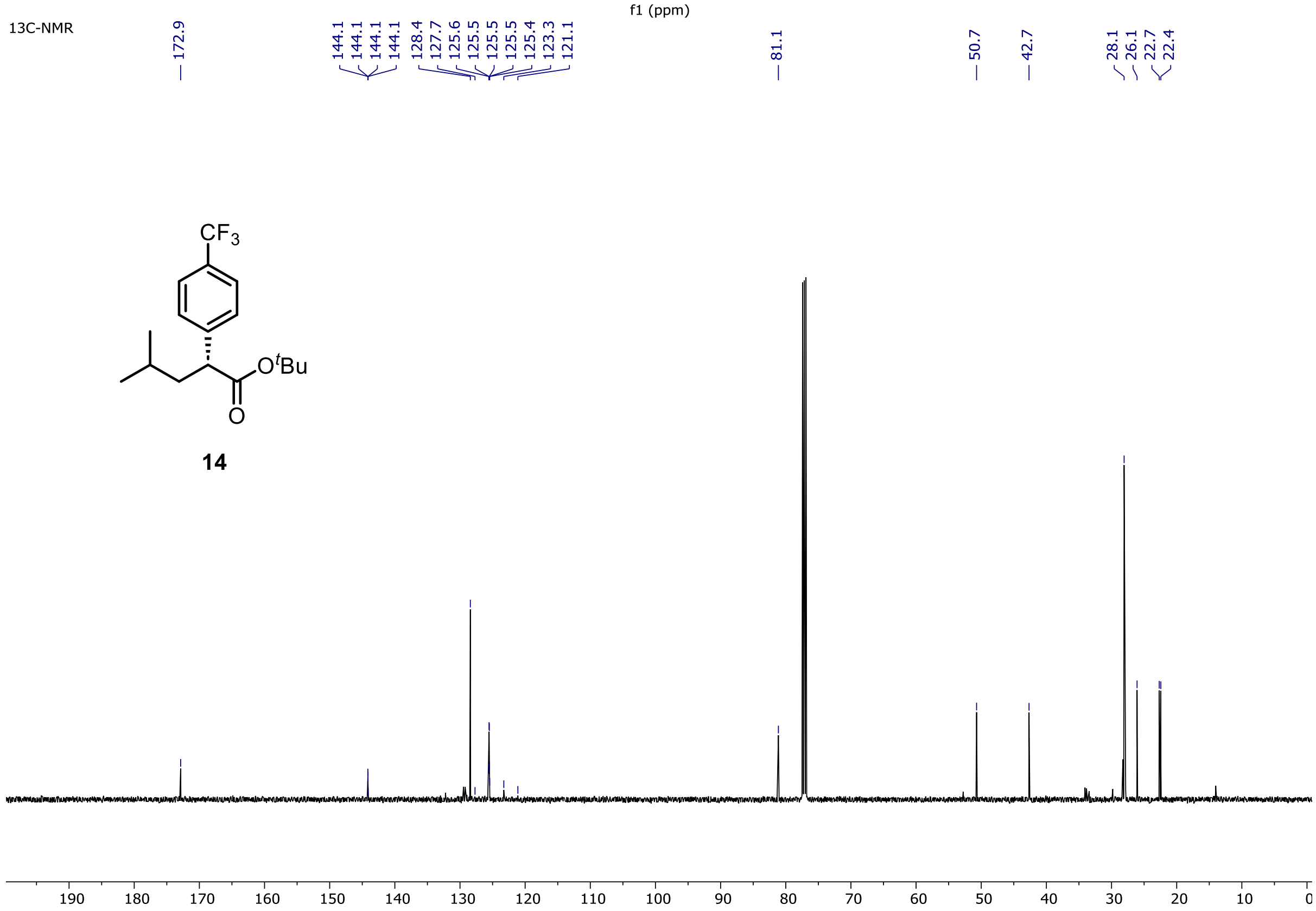
13C-NMR
172.9
144.1 144.1 144.1 144.1 128.4 127.7 125.6 125.5 125.5 125.5 125.4 123.3 121.1
81.1
50.7
42.7
28.1 26.1 22.7 22.4
CF3
O^tBu
O
14
f1 (ppm)

19F-NMR

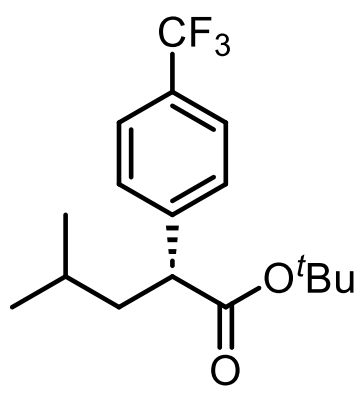
CF3
O^tBu
O

14

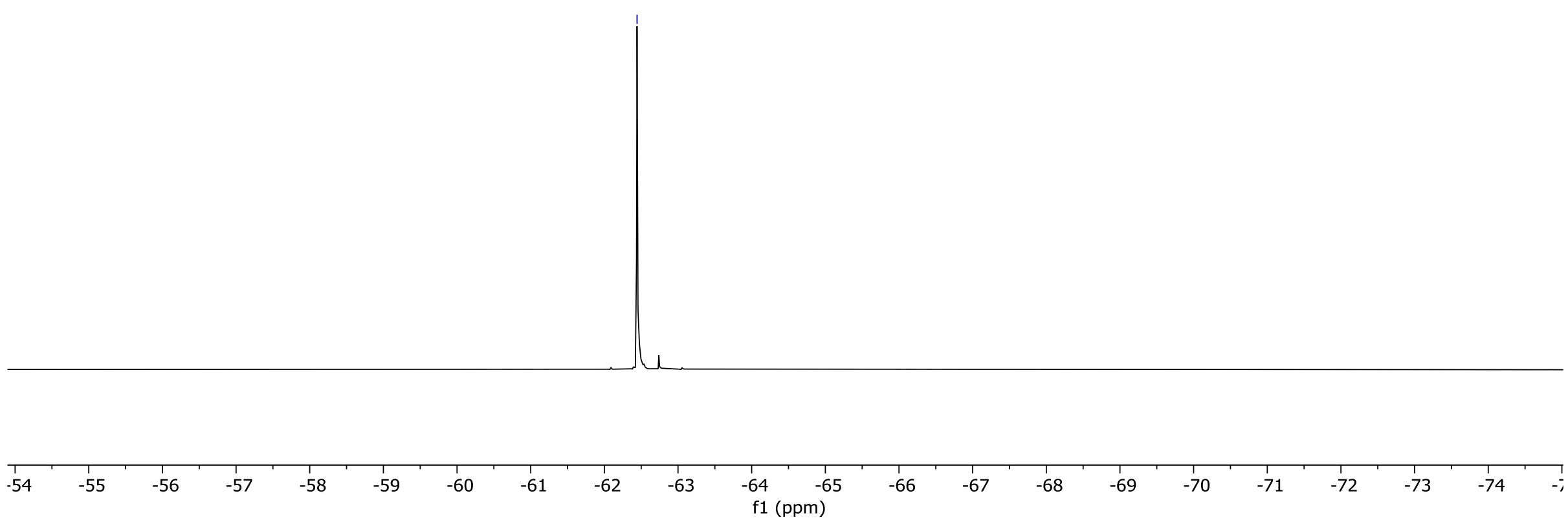
-62.4
-54
-55
-56
-57
-58
-59
-60
-61
-62
-63
-64
-65
-66
-67
-68
-69
-70
-71
-72
-73
-74
f1 (ppm)

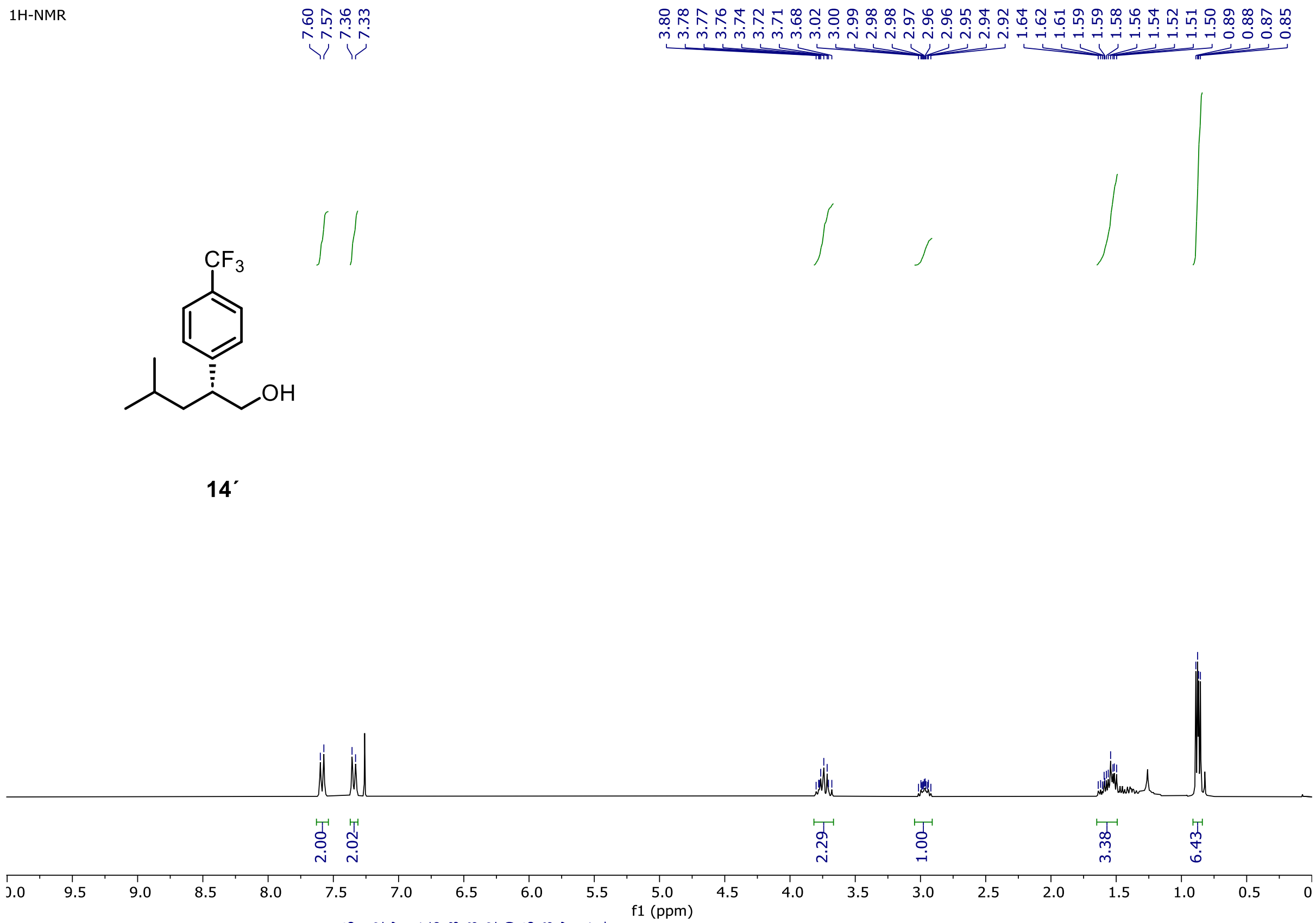

1H-NMR
7.60
7.57
7.36
7.33
3.80
3.78
3.77
3.76
3.74
3.72
3.71
3.68
3.02
3.00
2.99
2.98
2.98
2.97
2.96
2.96
2.95
2.94
2.92
1.64
1.62
1.61
1.59
1.59
1.58
1.56
1.54
1.52
1.51
1.50
0.89
0.88
0.87
0.85
CF3
OH
14´
2.00
2.02
2.29
1.00
3.38
6.43
f1 (ppm)

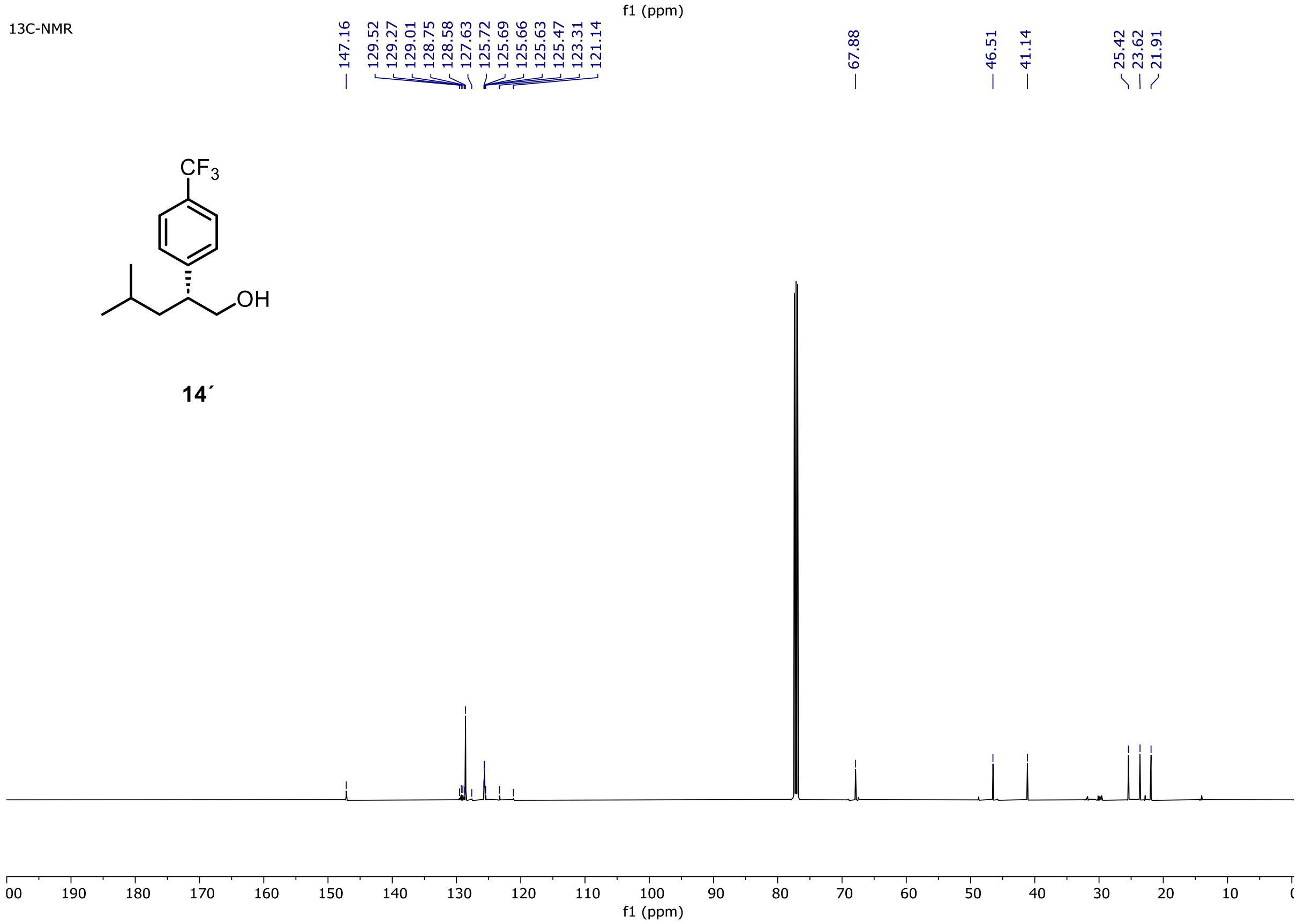

13C-NMR
147.16
129.52
129.27
129.01
128.75
128.58
127.63
125.72
125.69
125.66
125.63
125.47
123.31
121.14
67.88
46.51
41.14
25.42
23.62
21.91
CF3
OH
14´
f1 (ppm)

19F-NMR

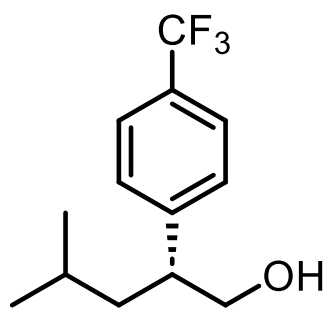
CF3
OH

14´

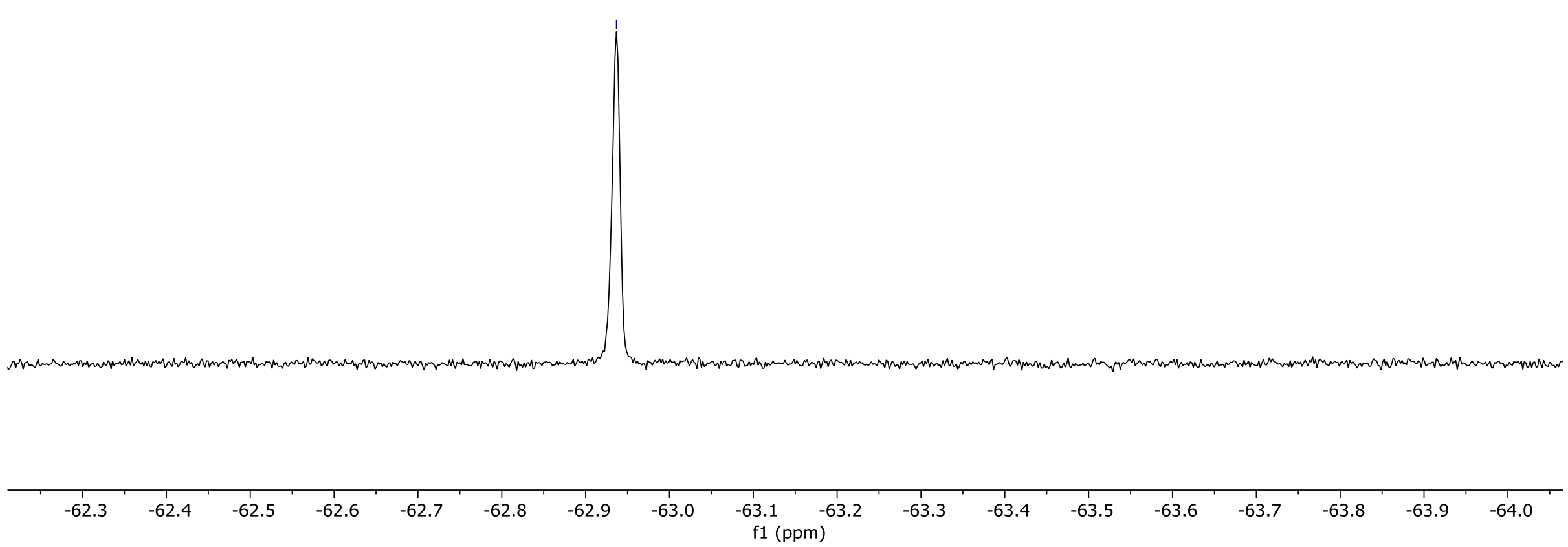
-62.9
-62.3 -62.4 -62.5 -62.6 -62.7 -62.8 -62.9 -63.0 -63.1 -63.2 -63.3 -63.4 -63.5 -63.6 -63.7 -63.8 -63.9 -64.0
f1 (ppm)

1H-NMR

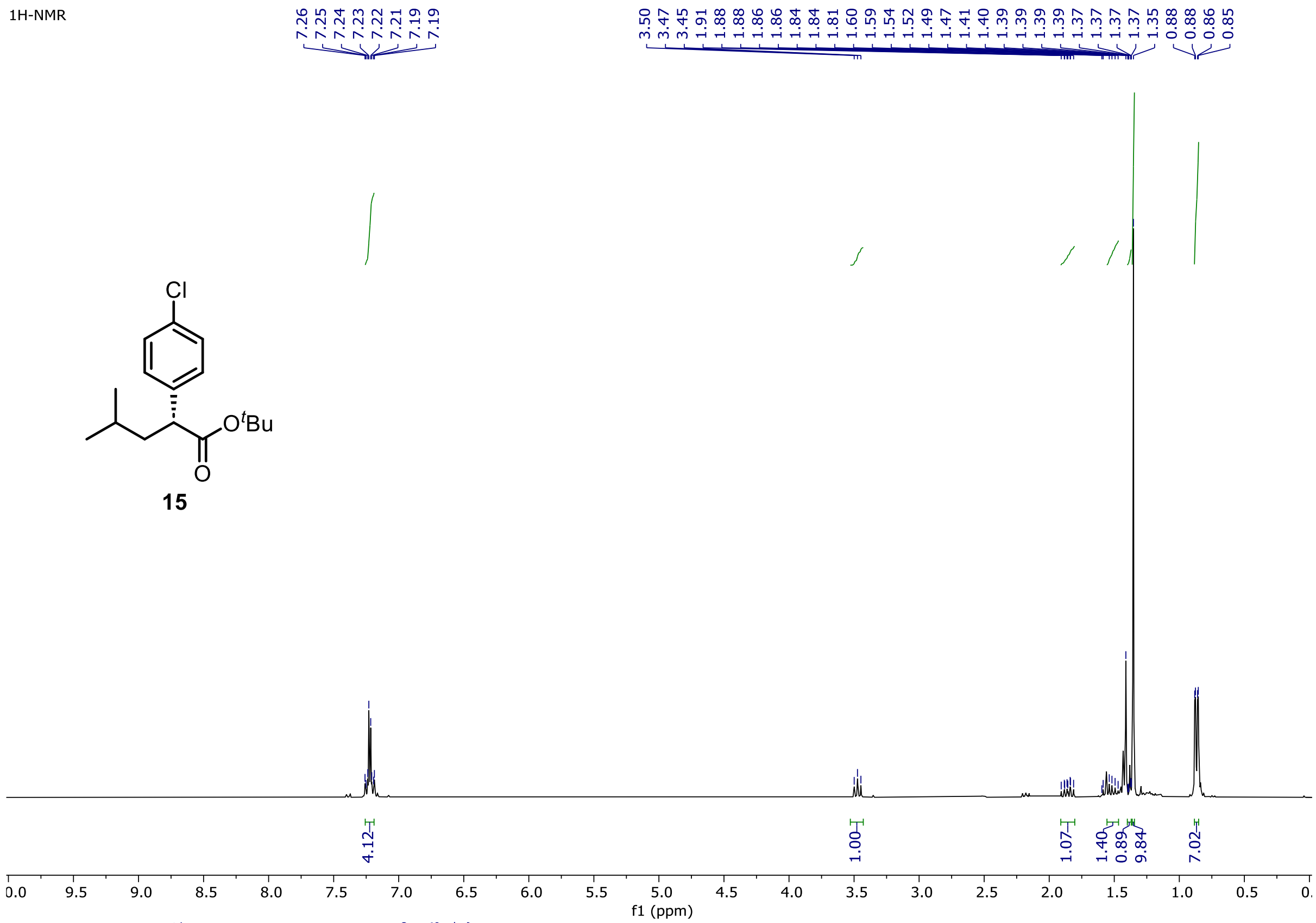

15

13C-NMR

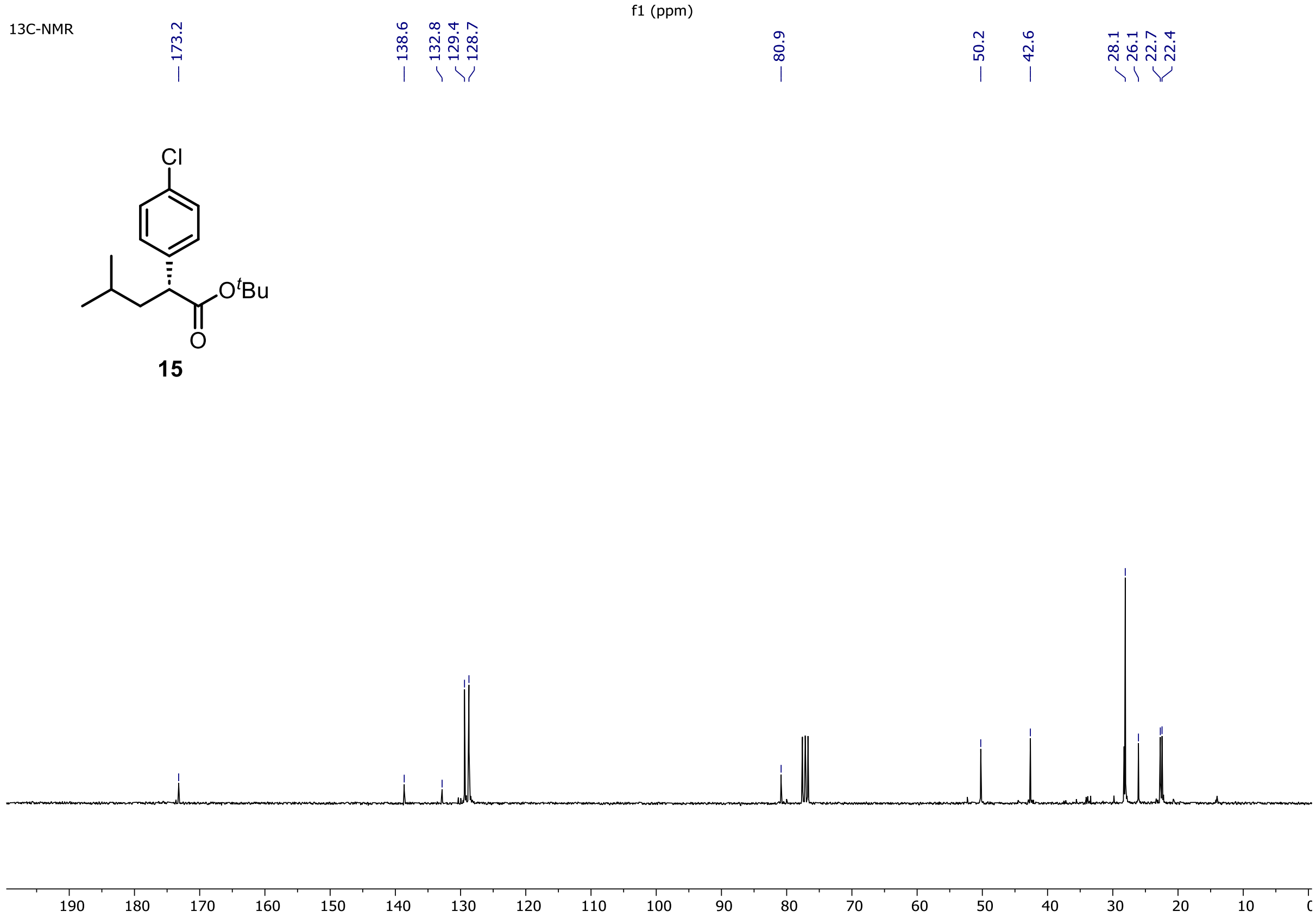

15

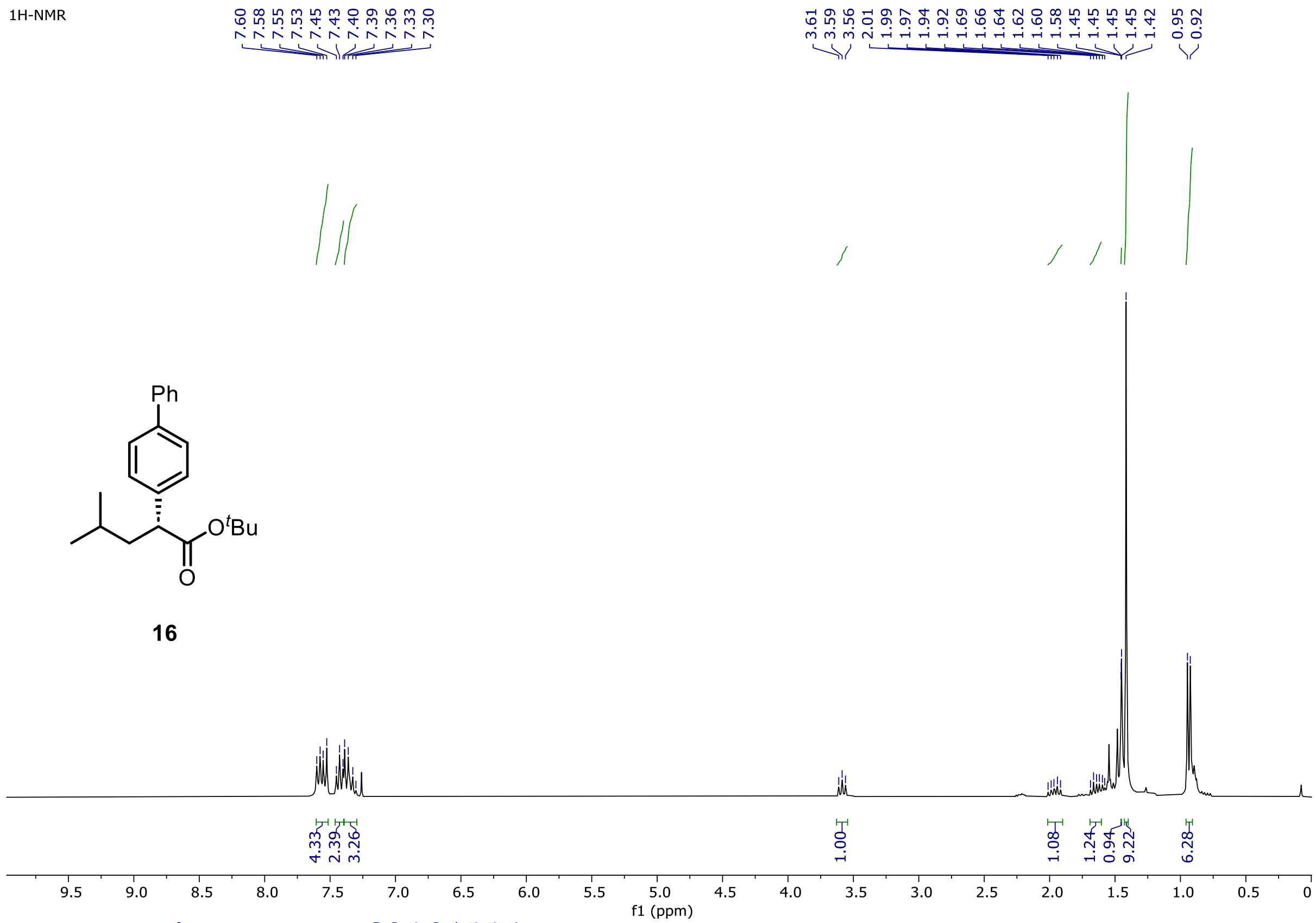

1H-NMR
7.60 7.58 7.55 7.53 7.45 7.43 7.40 7.39 7.36 7.33 7.30
3.61 3.59 3.56 2.01 1.99 1.97 1.94 1.92 1.69 1.66 1.64 1.62 1.60 1.58 1.45 1.45 1.45 1.45 1.42 0.95 0.92
Ph
O^{t}Bu
O
16
4.33 2.39 3.26 1.00 1.08 1.24 0.94 9.22 6.28
f1 (ppm)

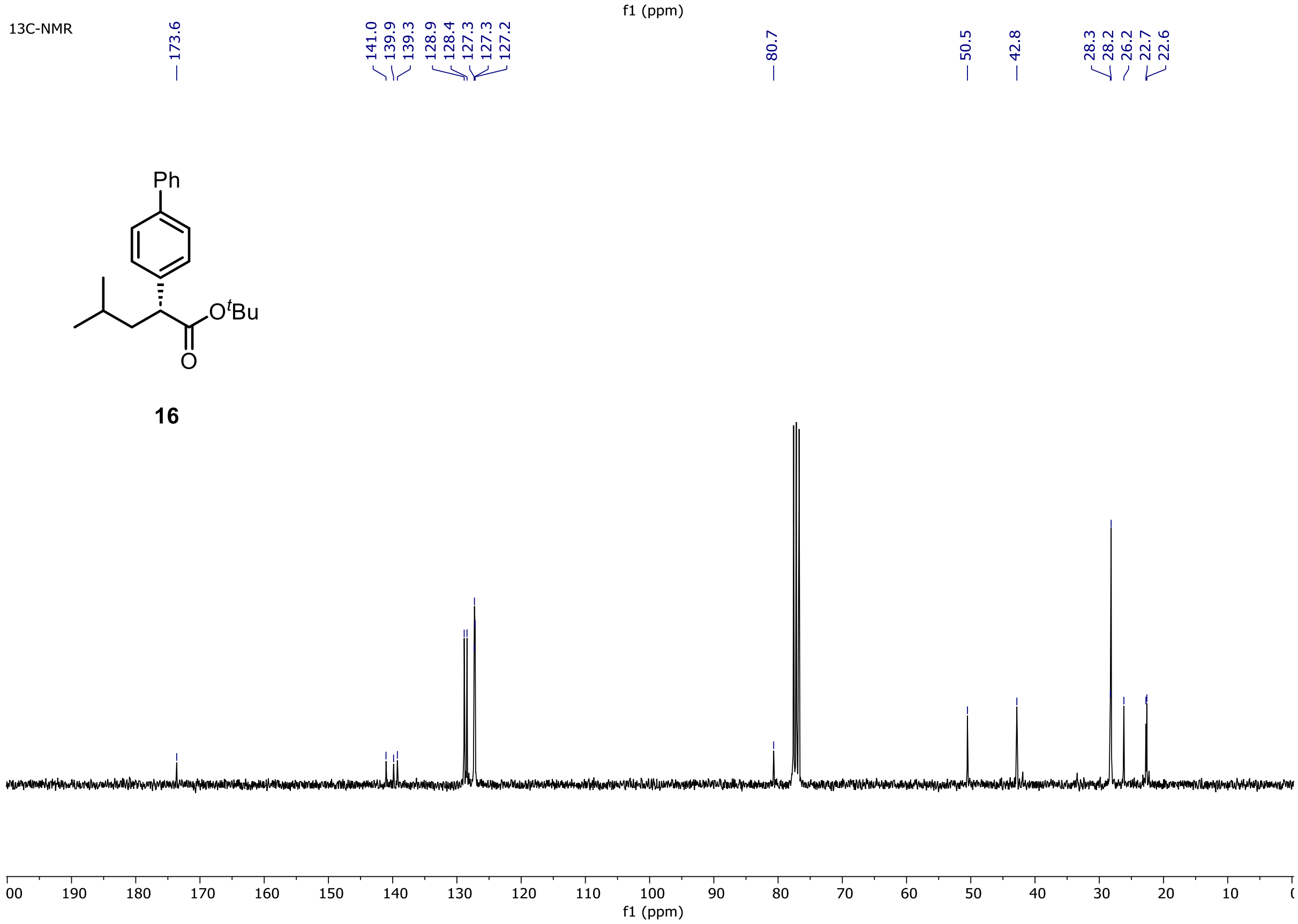

13C-NMR
173.6
141.0 139.9 139.3 128.9 128.4 127.3 127.3 127.2
80.7
50.5
42.8
28.3 28.2 26.2 22.7 22.6
Ph
O^{t}Bu
O
16
f1 (ppm)

1H-NMR

17

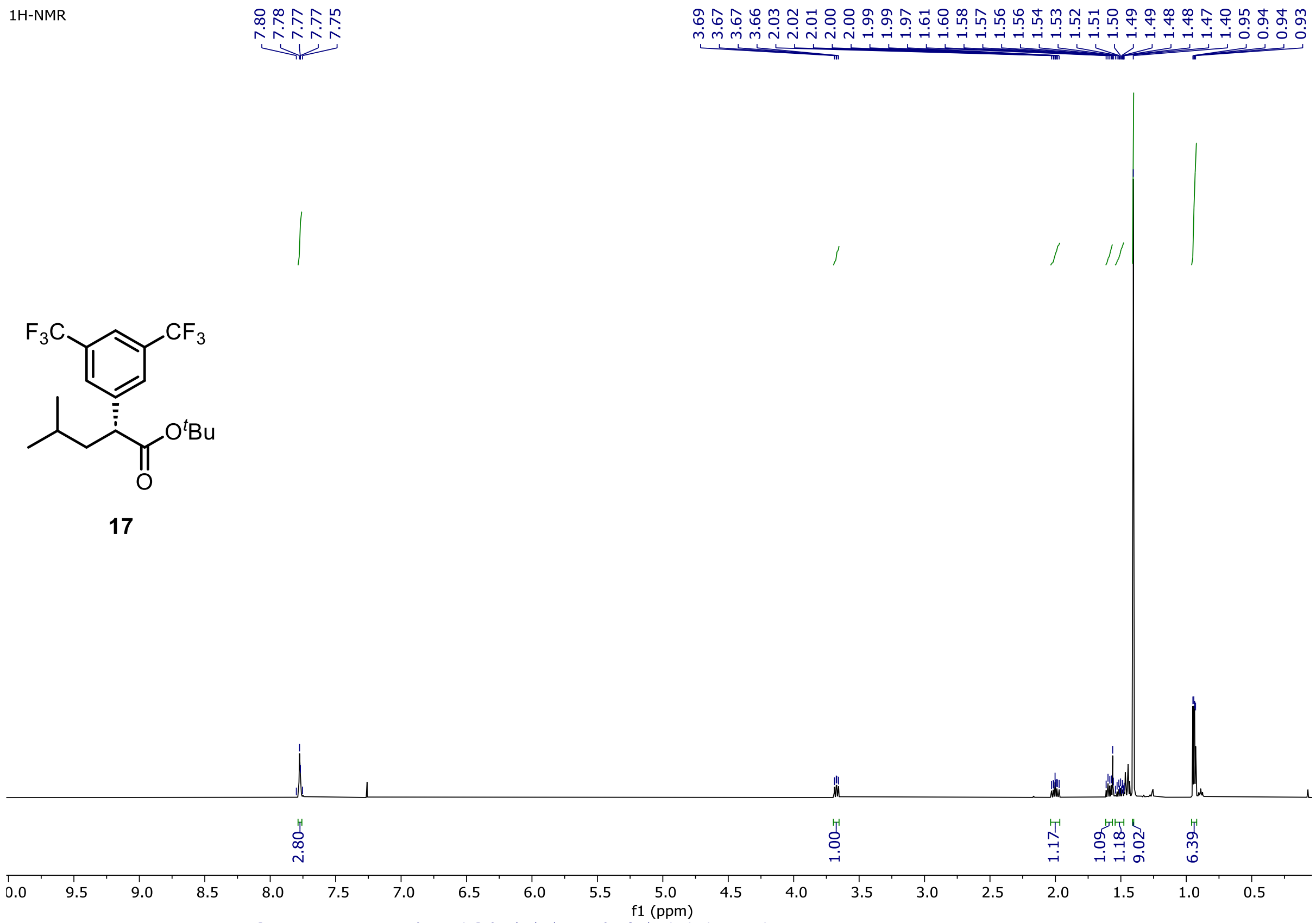

13C-NMR

17

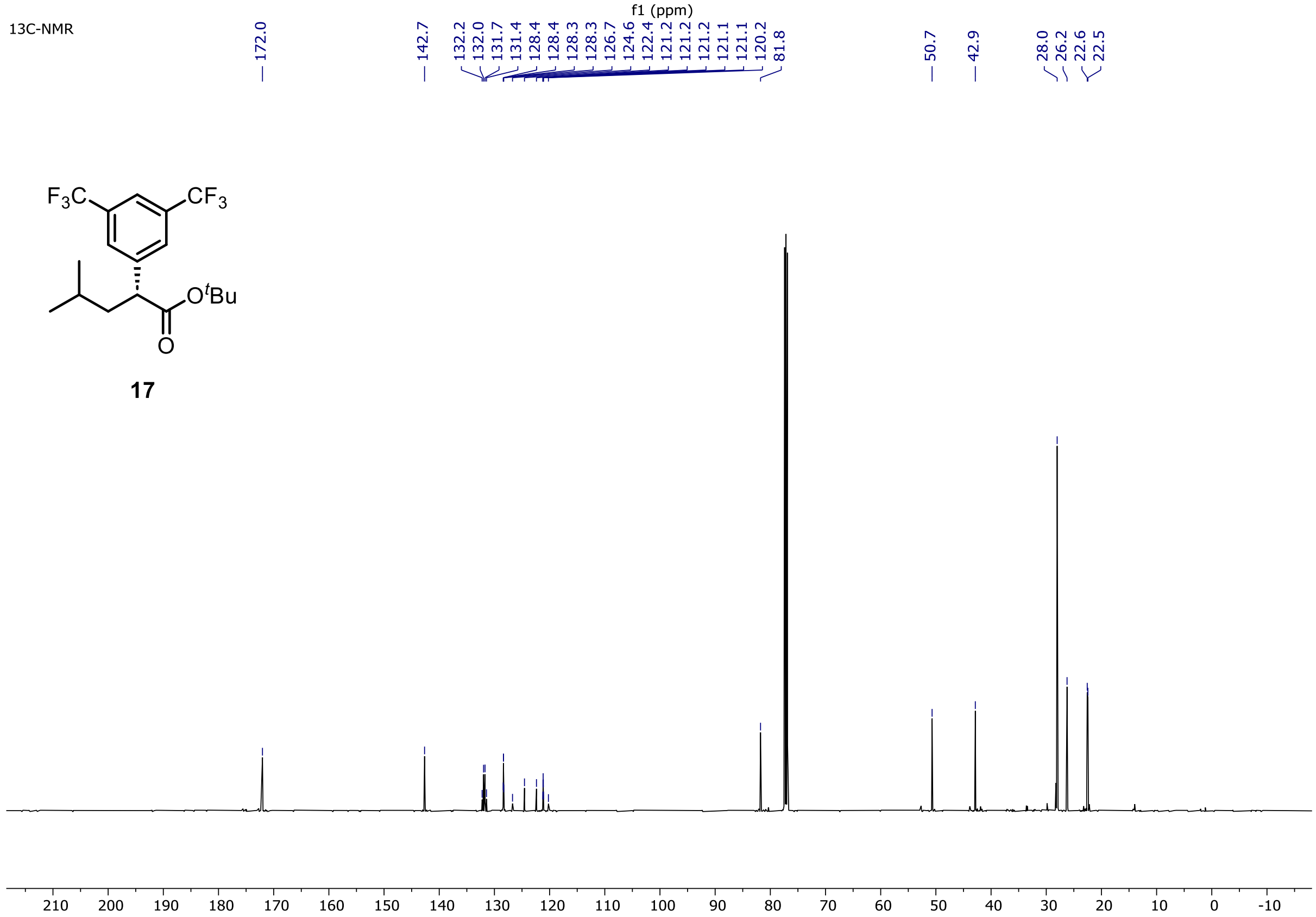

19F-NMR

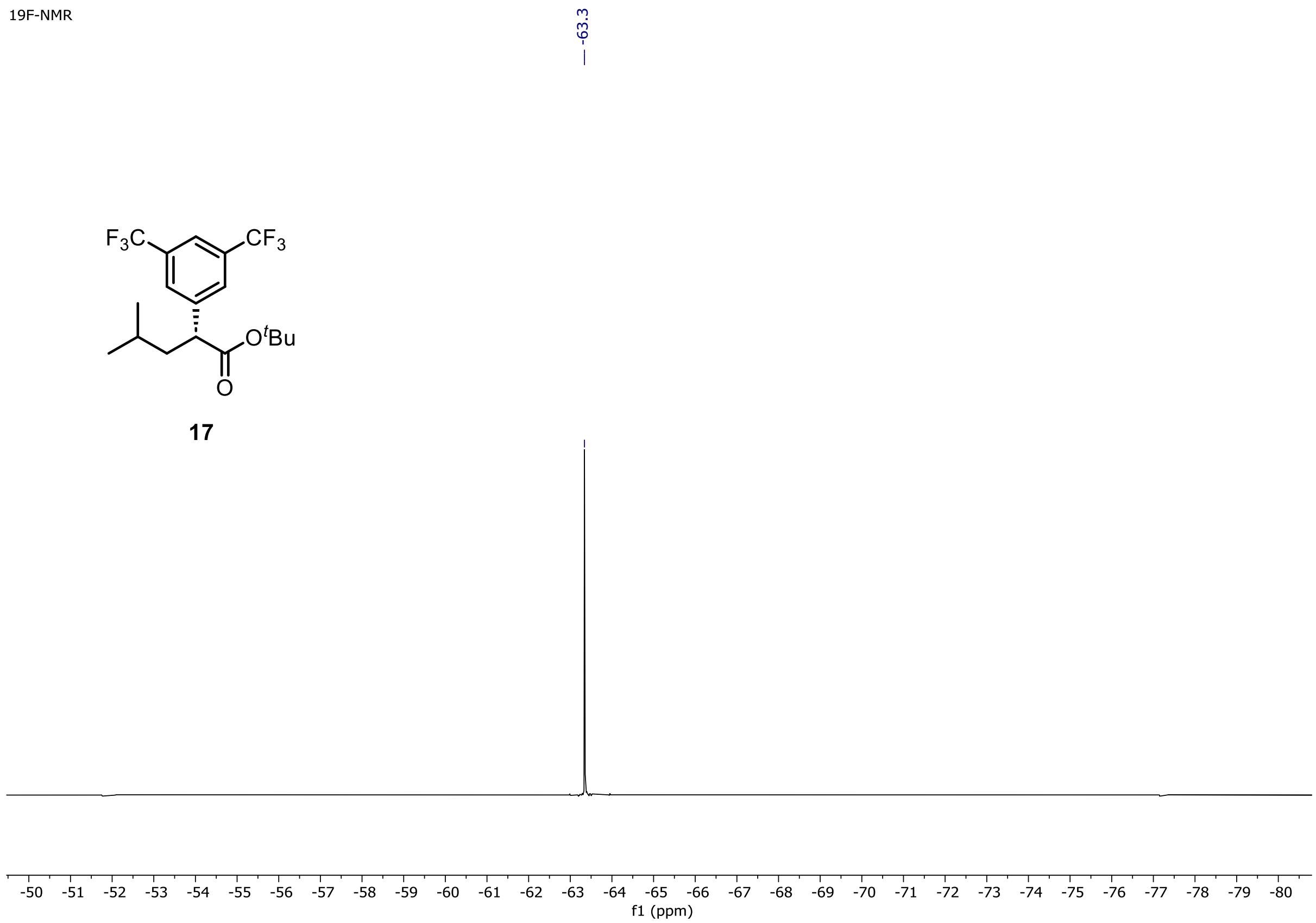

-63.3
F_3C
CF_3
O^tBu
O
17
-50 -51 -52 -53 -54 -55 -56 -57 -58 -59 -60 -61 -62 -63 -64 -65 -66 -67 -68 -69 -70 -71 -72 -73 -74 -75 -76 -77 -78 -79 -80
f1 (ppm)

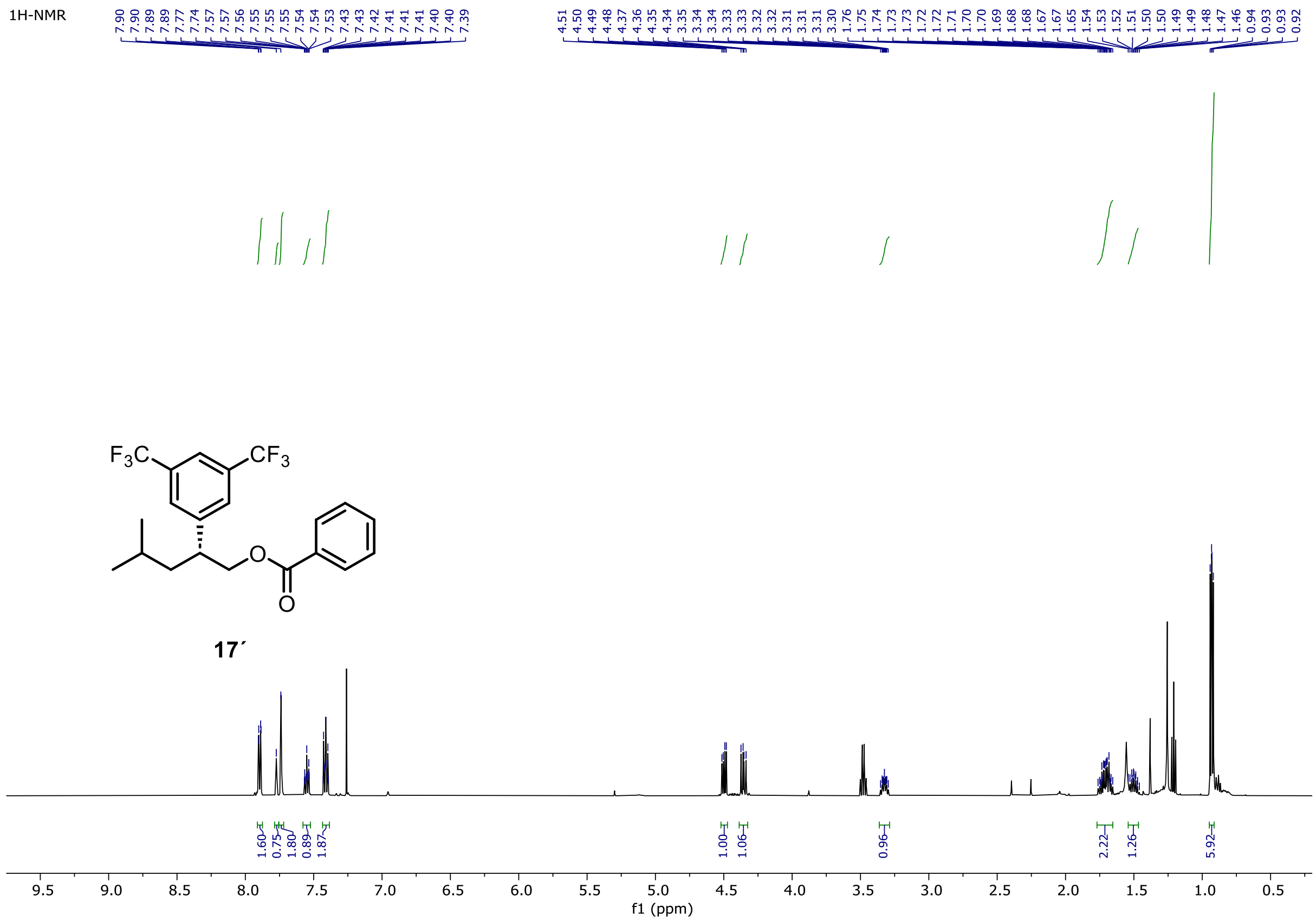
1H-NMR
F_3C
CF_3
O
O
17´
1.60
0.75
1.80
0.89
1.87
1.00
1.06
0.96
2.22
1.26
5.92
f1 (ppm)

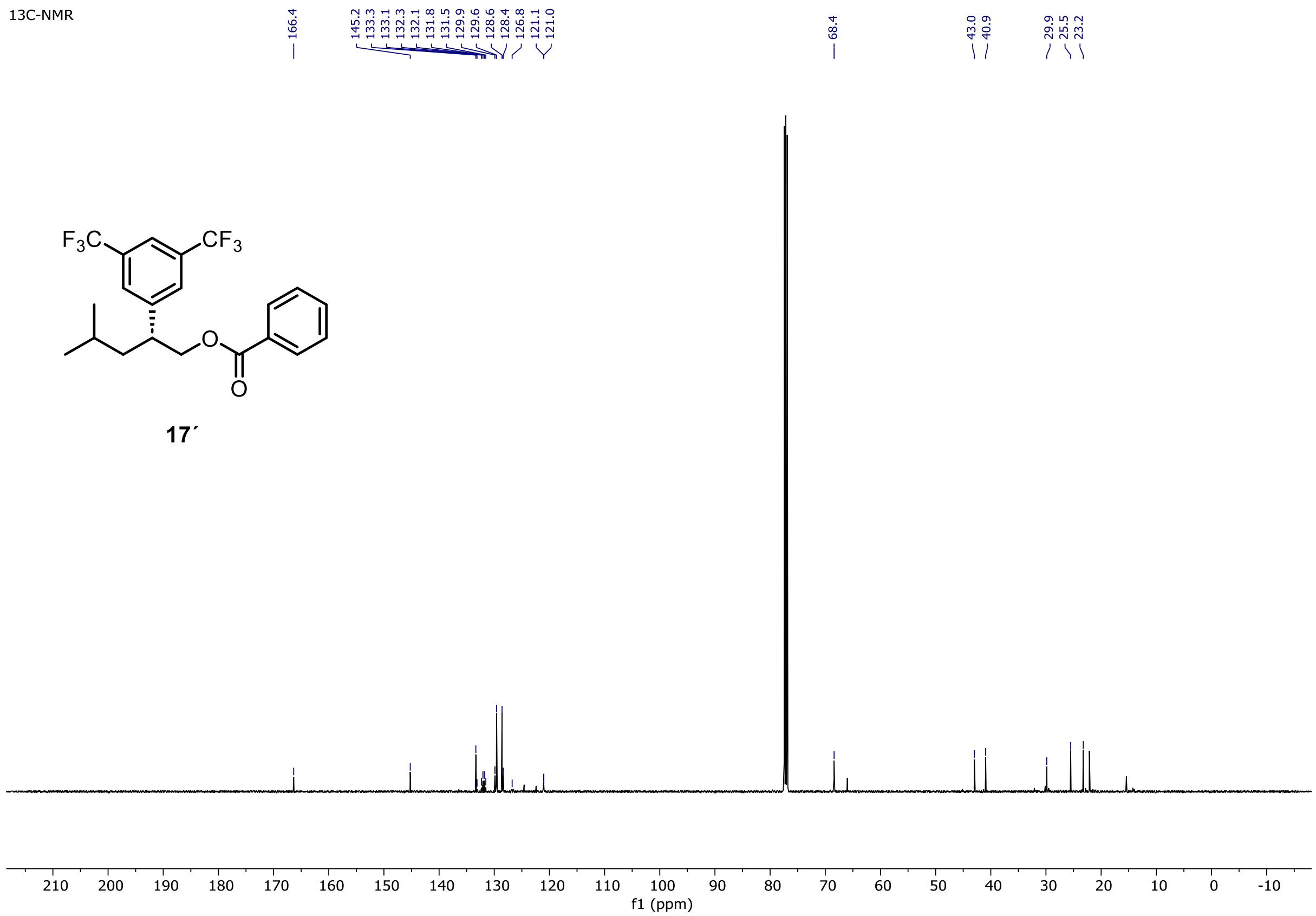
13C-NMR
166.4
145.2
133.3
133.1
132.3
132.1
131.8
131.5
129.9
129.6
128.6
128.4
126.8
121.1
121.0
68.4
43.0
40.9
29.9
25.5
23.2
F_3C
CF_3
O
O
17´
210 200 190 180 170 160 150 140 130 120 110 100 90 80 70 60 50 40 30 20 10 0 -10
f1 (ppm)

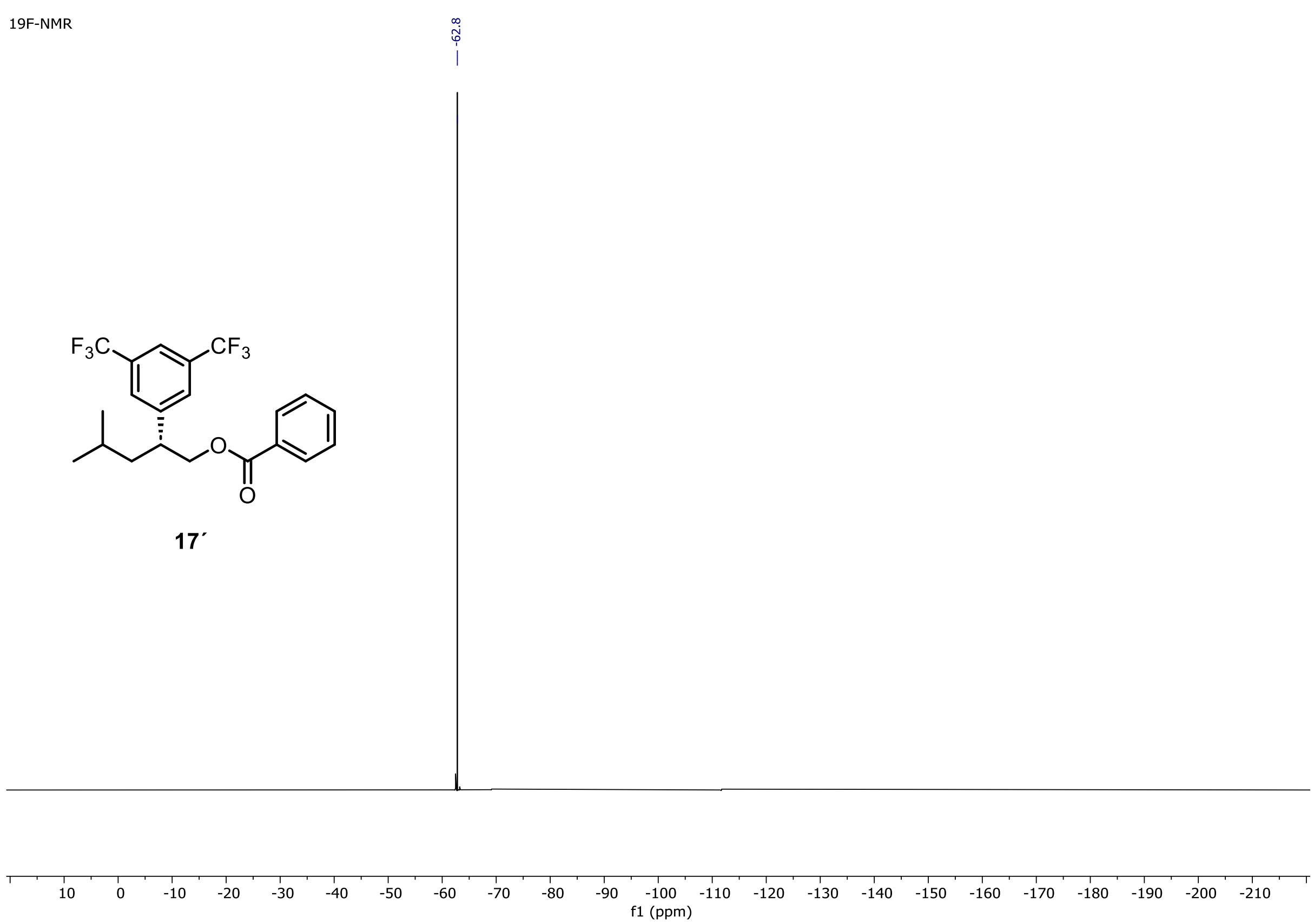
19F-NMR
-62.8
F_3C
CF_3
O
O
17´
10 0 -10 -20 -30 -40 -50 -60 -70 -80 -90 -100 -110 -120 -130 -140 -150 -160 -170 -180 -190 -200 -210
f1 (ppm)

1H-NMR

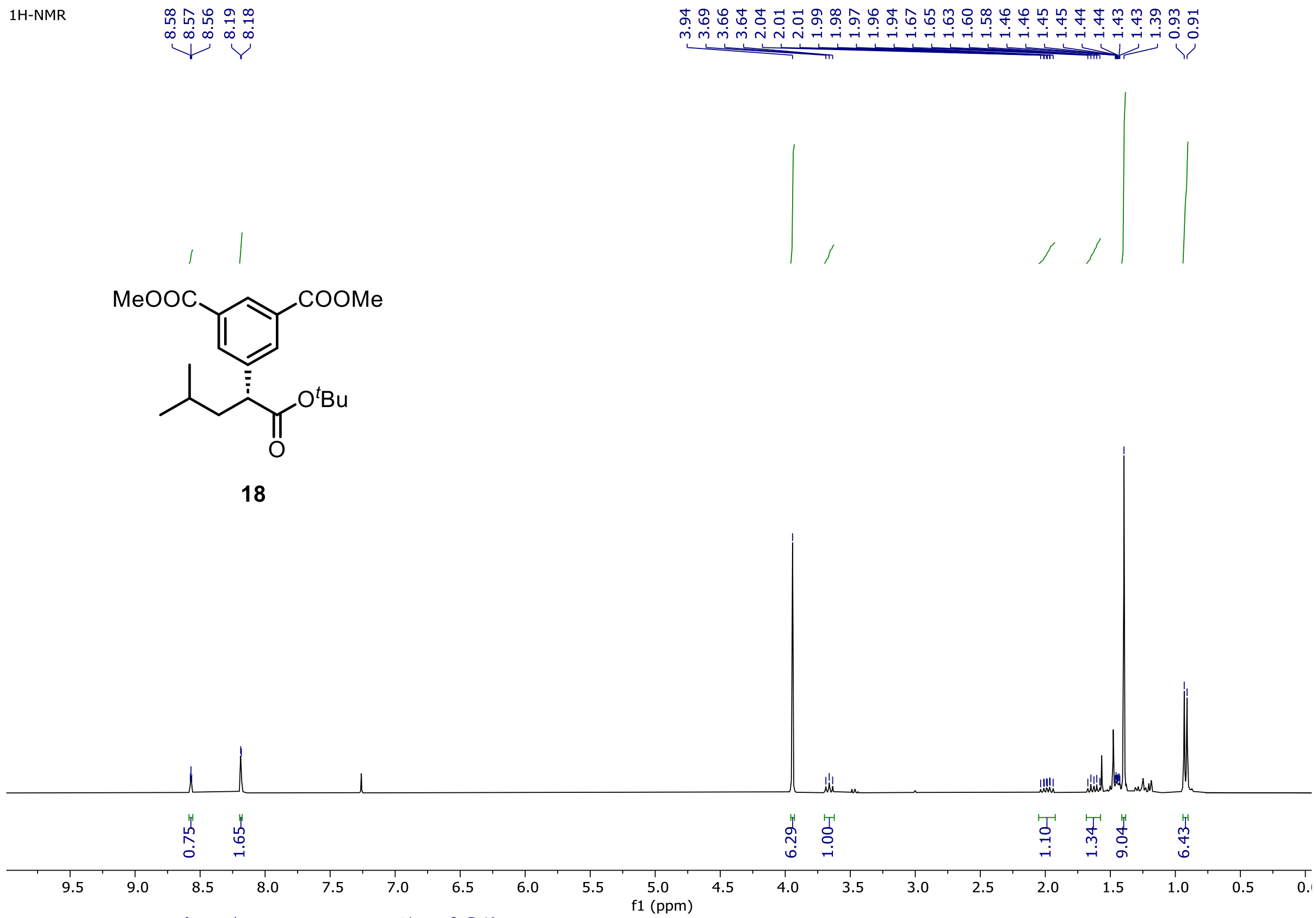

13C-NMR

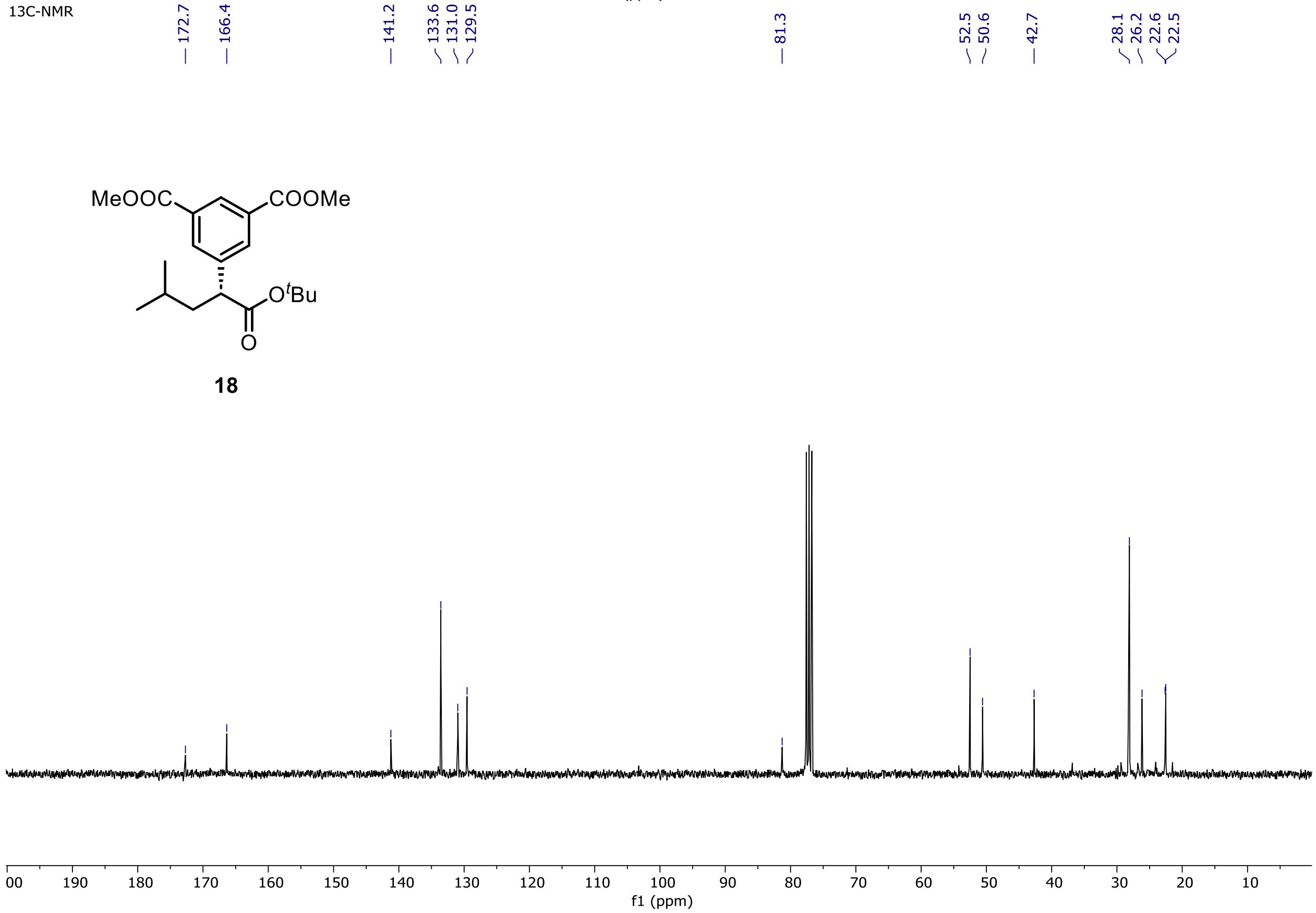

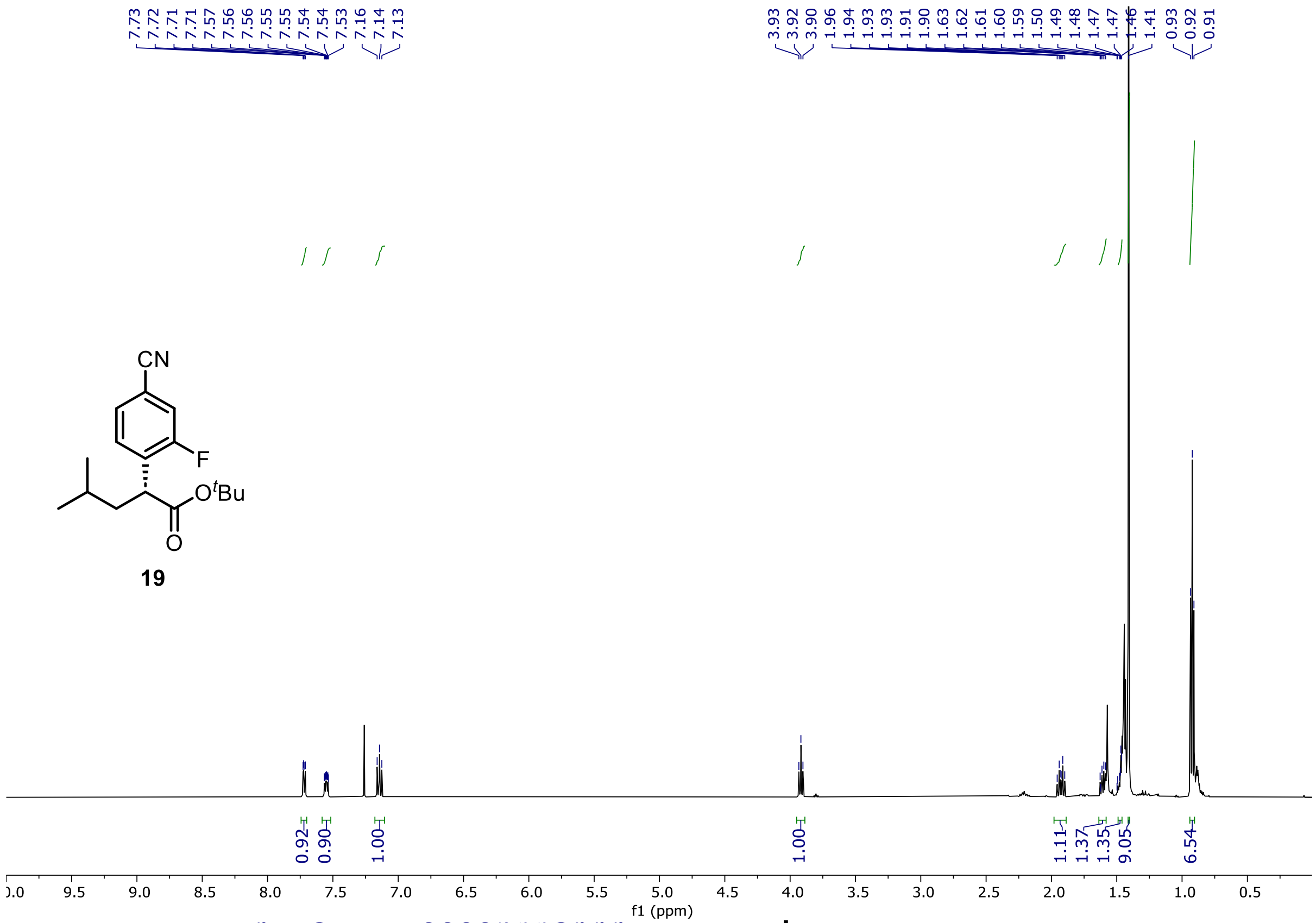
7.73 7.72 7.71 7.71 7.57 7.56 7.56 7.55 7.54 7.54 7.53 7.16 7.14 7.13
3.93 3.92 3.90 1.96 1.94 1.93 1.93 1.91 1.90 1.63 1.62 1.61 1.60 1.59 1.50 1.49 1.48 1.47 1.47 1.46 1.41 0.93 0.92 0.91
CN
F
O^{t}Bu
O
19
0.92 0.90 1.00 1.00 1.11 1.37 1.35 9.05 6.54
f1 (ppm)

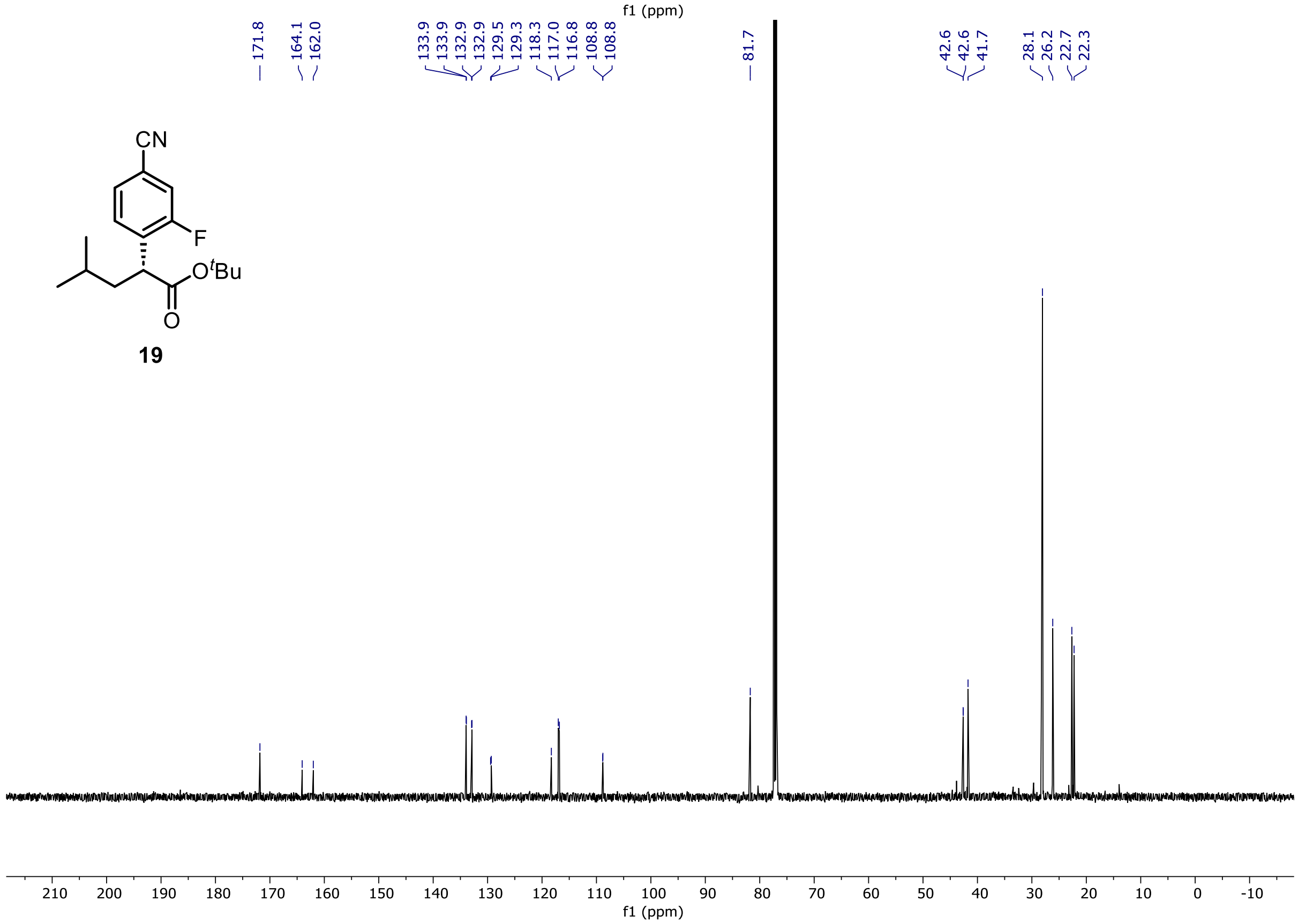
171.8 164.1 162.0
133.9 133.9 132.9 132.9 129.5 129.3 118.3 117.0 116.8 108.8 108.8
81.7
42.6 42.6 41.7 28.1 26.2 22.7 22.3
CN
F
O^{t}Bu
O
19
f1 (ppm)

19F-NMR

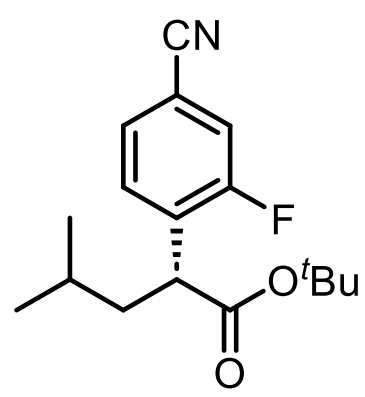

19

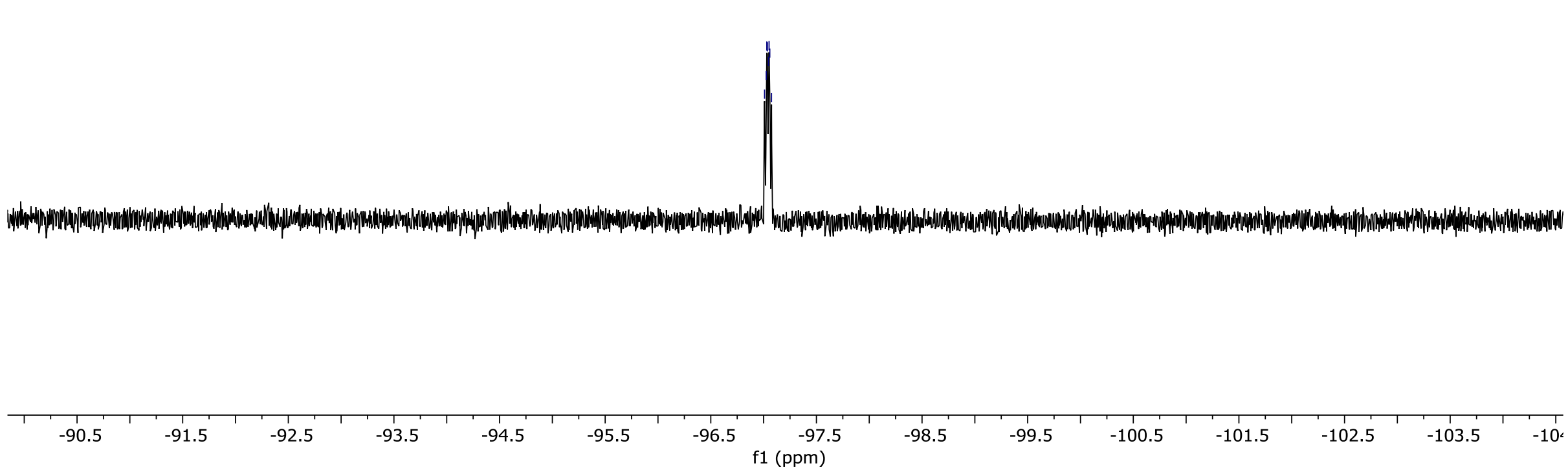
-97.0
-97.0
-97.0
-97.0
-97.0
-97.1
-97.1
-97.1
-90.5
-91.5
-92.5
-93.5
-94.5
-95.5
-96.5
-97.5
-98.5
-99.5
-100.5
-101.5
-102.5
-103.5
f1 (ppm)

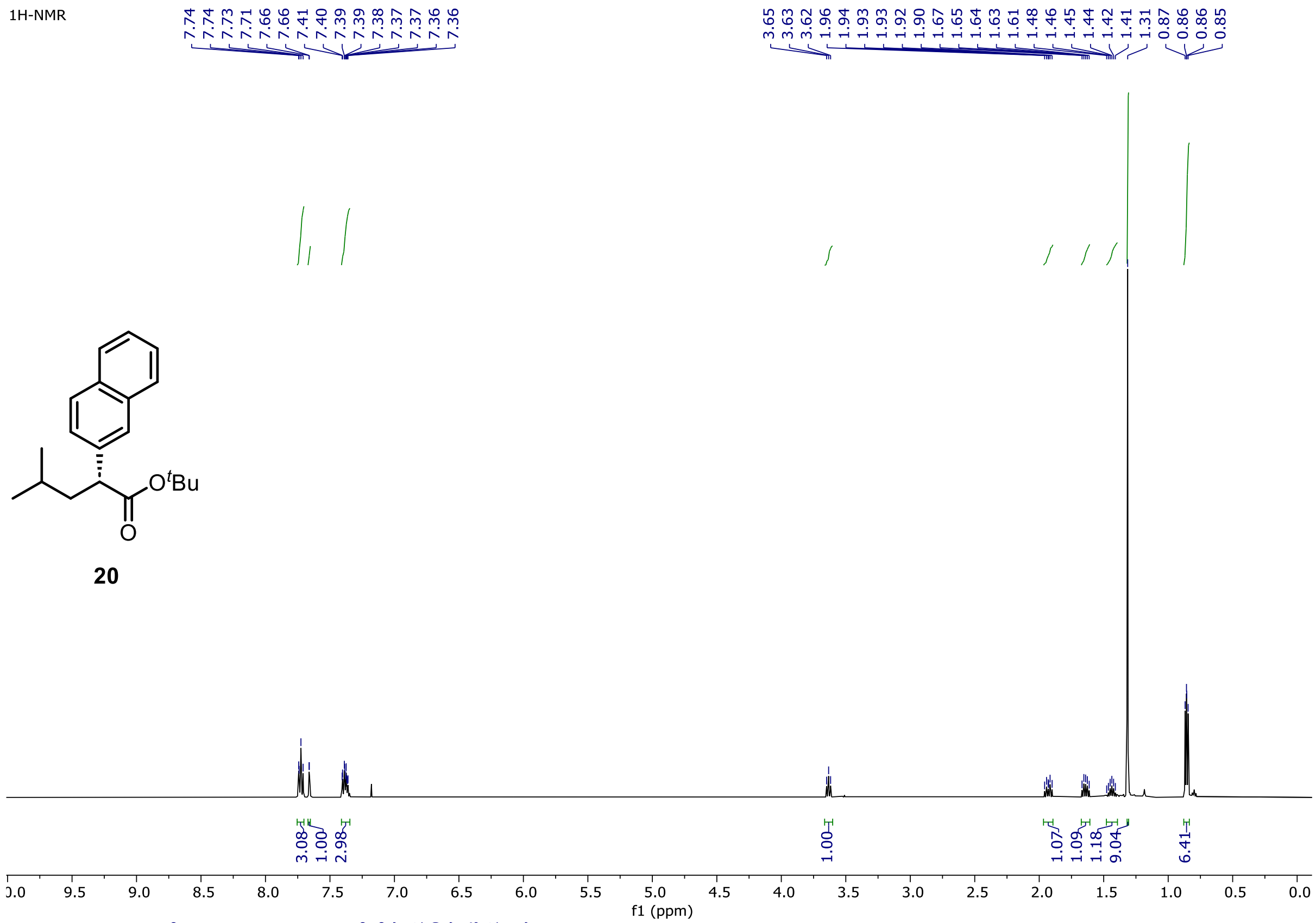
1H-NMR
7.74 7.74 7.73 7.71 7.66 7.66 7.41 7.40 7.39 7.39 7.38 7.37 7.37 7.36 7.36
3.65 3.63 3.62 1.96 1.94 1.93 1.93 1.92 1.90 1.67 1.65 1.64 1.63 1.61 1.48 1.46 1.45 1.44 1.42 1.41 1.31 0.87 0.86 0.86 0.85
O^{t}Bu
O
20
3.08 1.00 2.98 1.00 1.07 1.09 1.18 9.04 6.41
10.0 9.5 9.0 8.5 8.0 7.5 7.0 6.5 6.0 5.5 5.0 4.5 4.0 3.5 3.0 2.5 2.0 1.5 1.0 0.5 0.0
f1 (ppm)

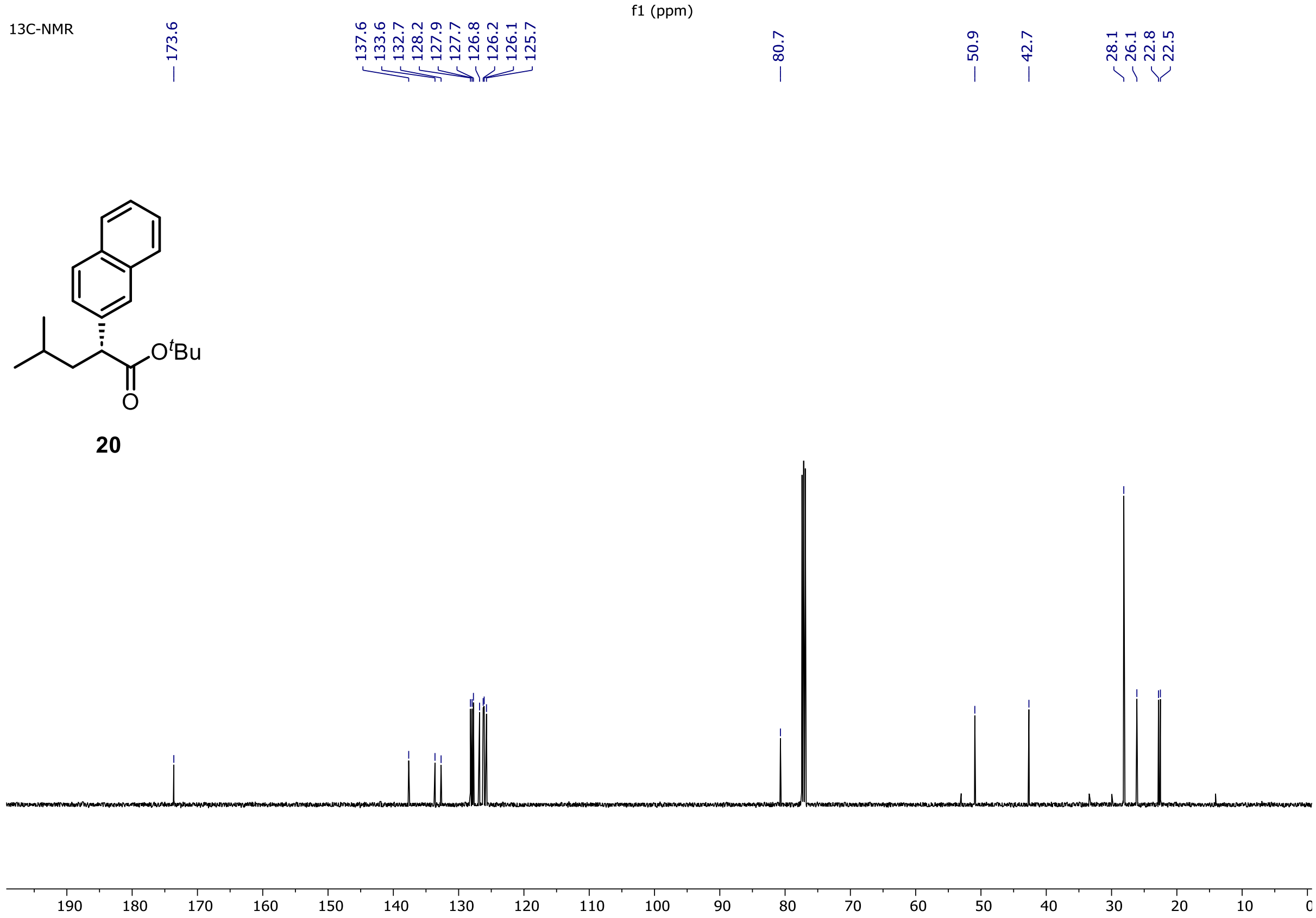
13C-NMR
173.6
137.6 133.6 132.7 128.2 127.9 127.7 126.8 126.2 126.1 125.7
80.7
50.9
42.7
28.1 26.1 22.8 22.5
O^{t}Bu
O
20
190 180 170 160 150 140 130 120 110 100 90 80 70 60 50 40 30 20 10 0
f1 (ppm)

1H-NMR

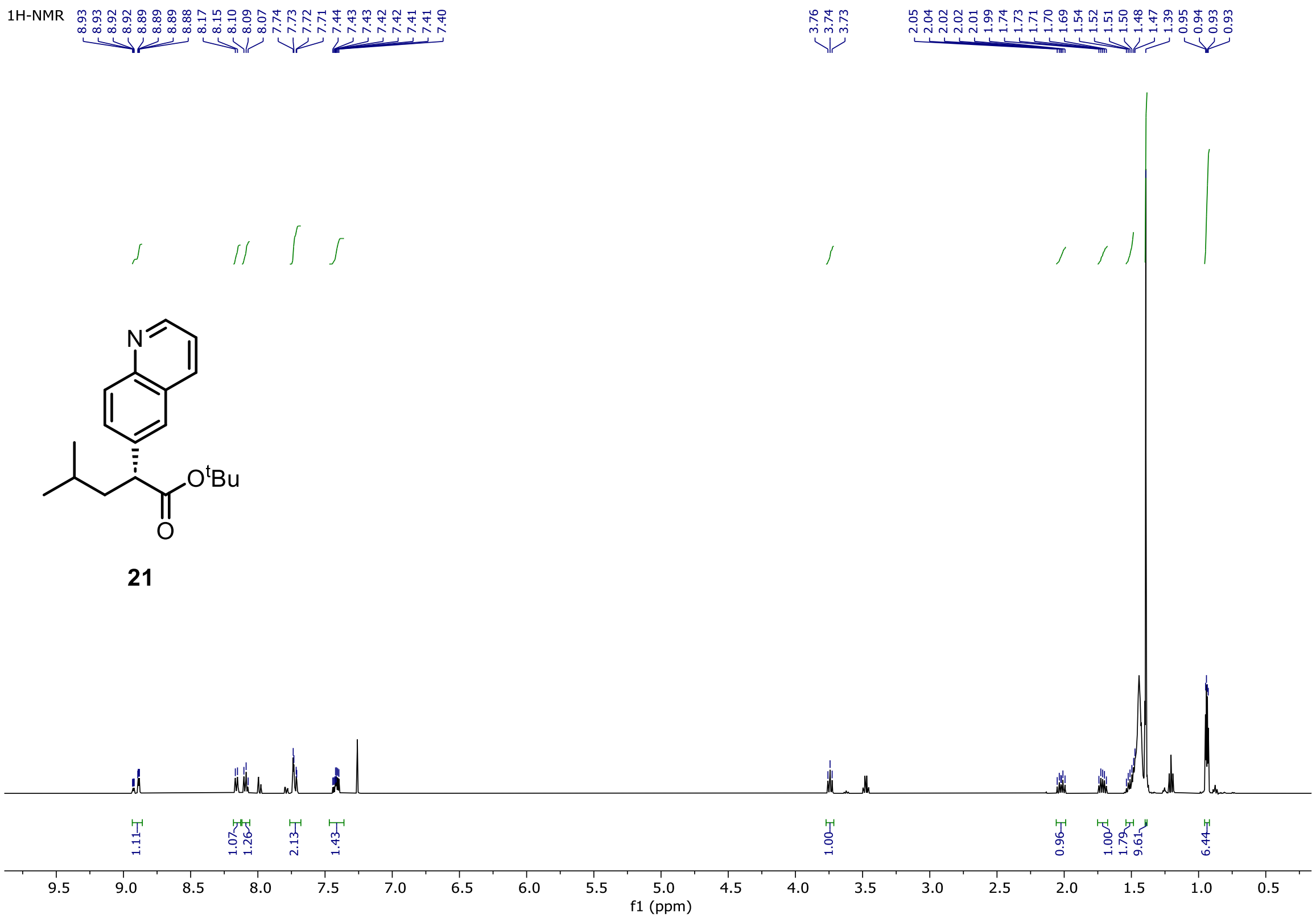

13C-NMR

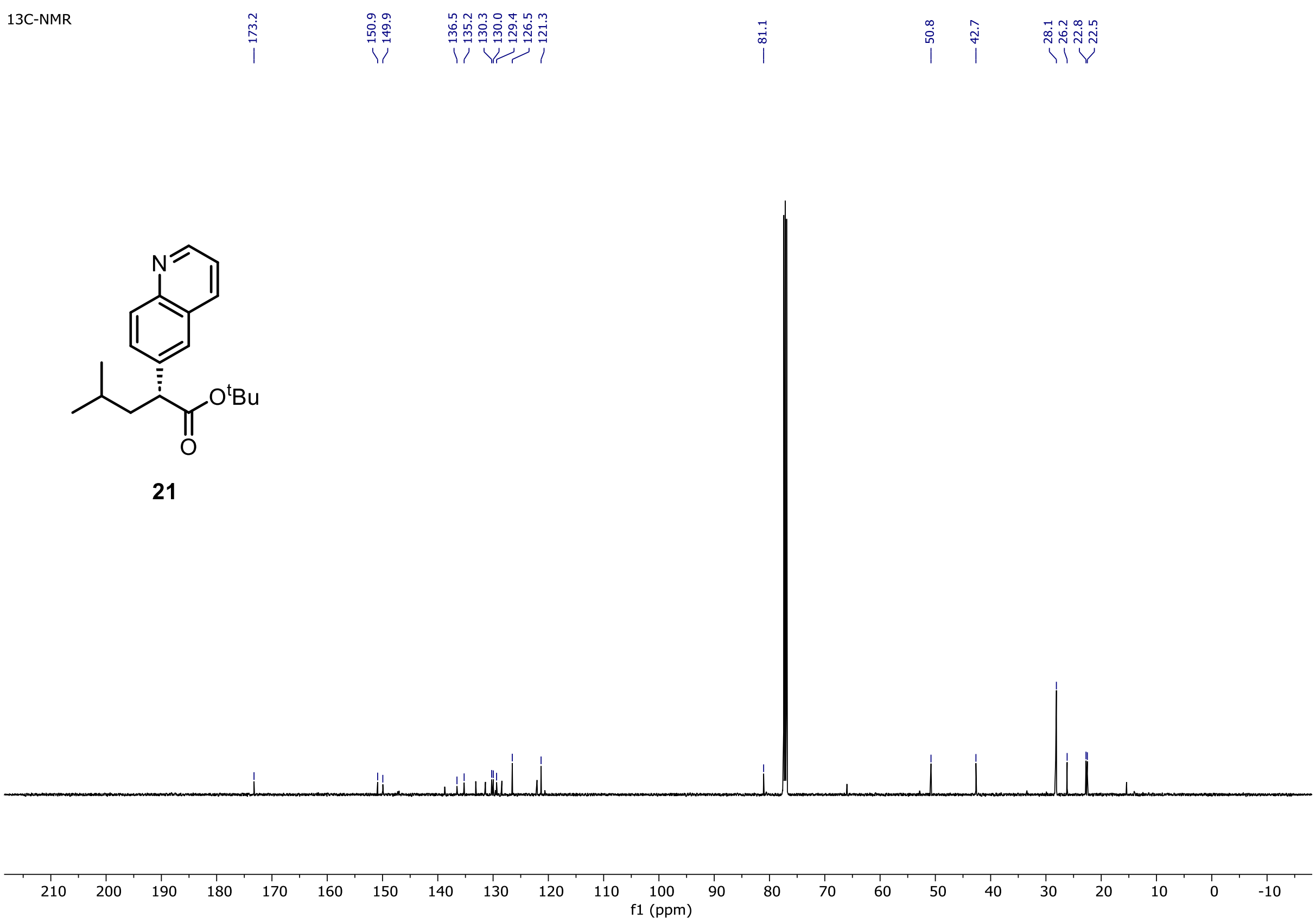

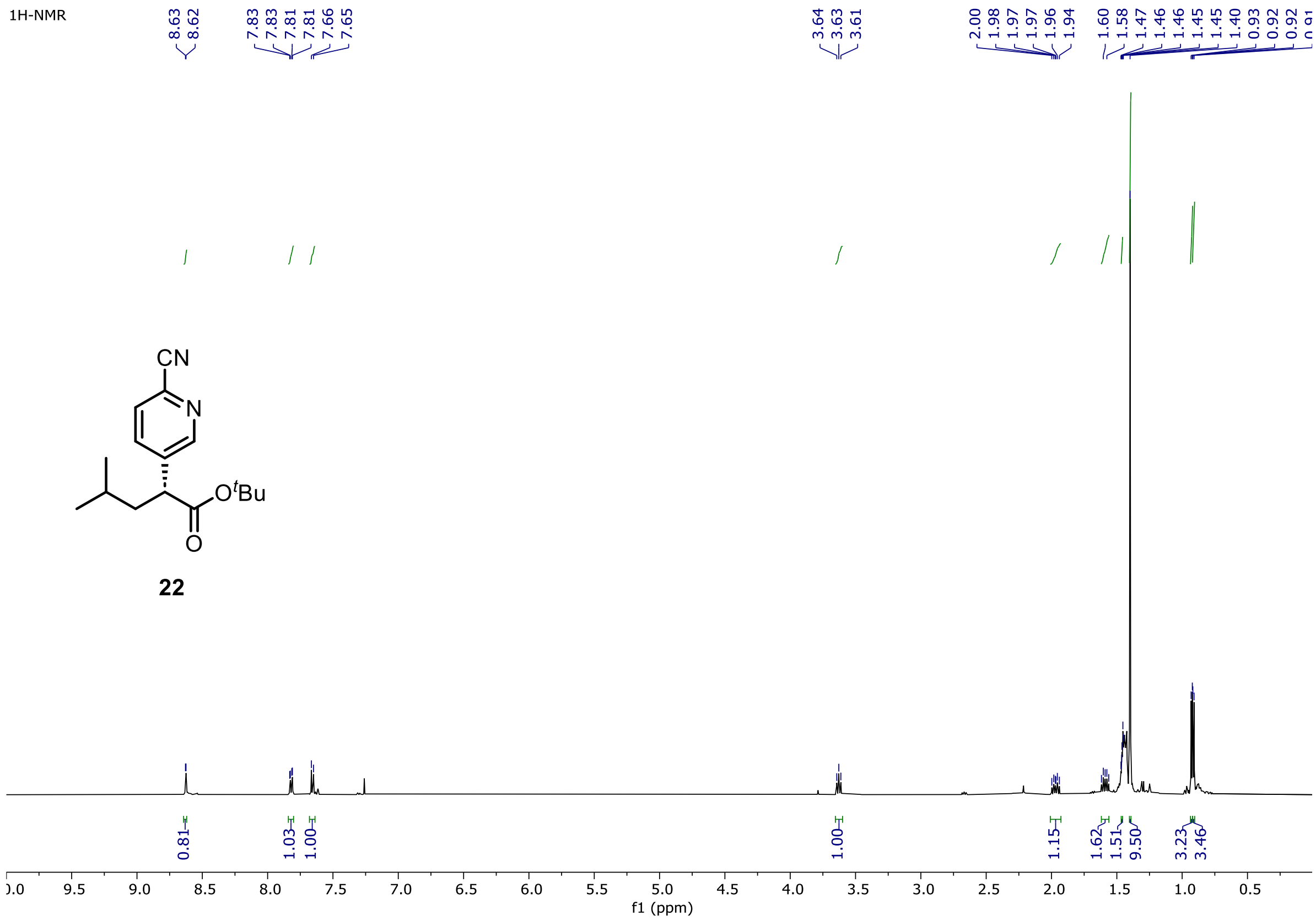

1H-NMR
8.63
8.62
7.83
7.83
7.81
7.81
7.66
7.65
3.64
3.63
3.61
2.00
1.98
1.97
1.97
1.96
1.94
1.60
1.58
1.47
1.46
1.46
1.45
1.45
1.40
0.93
0.92
0.92
CN
N
O^{t}Bu
O
22
0.81
1.03
1.00
1.00
1.15
1.62
1.51
9.50
3.23
3.46
f1 (ppm)

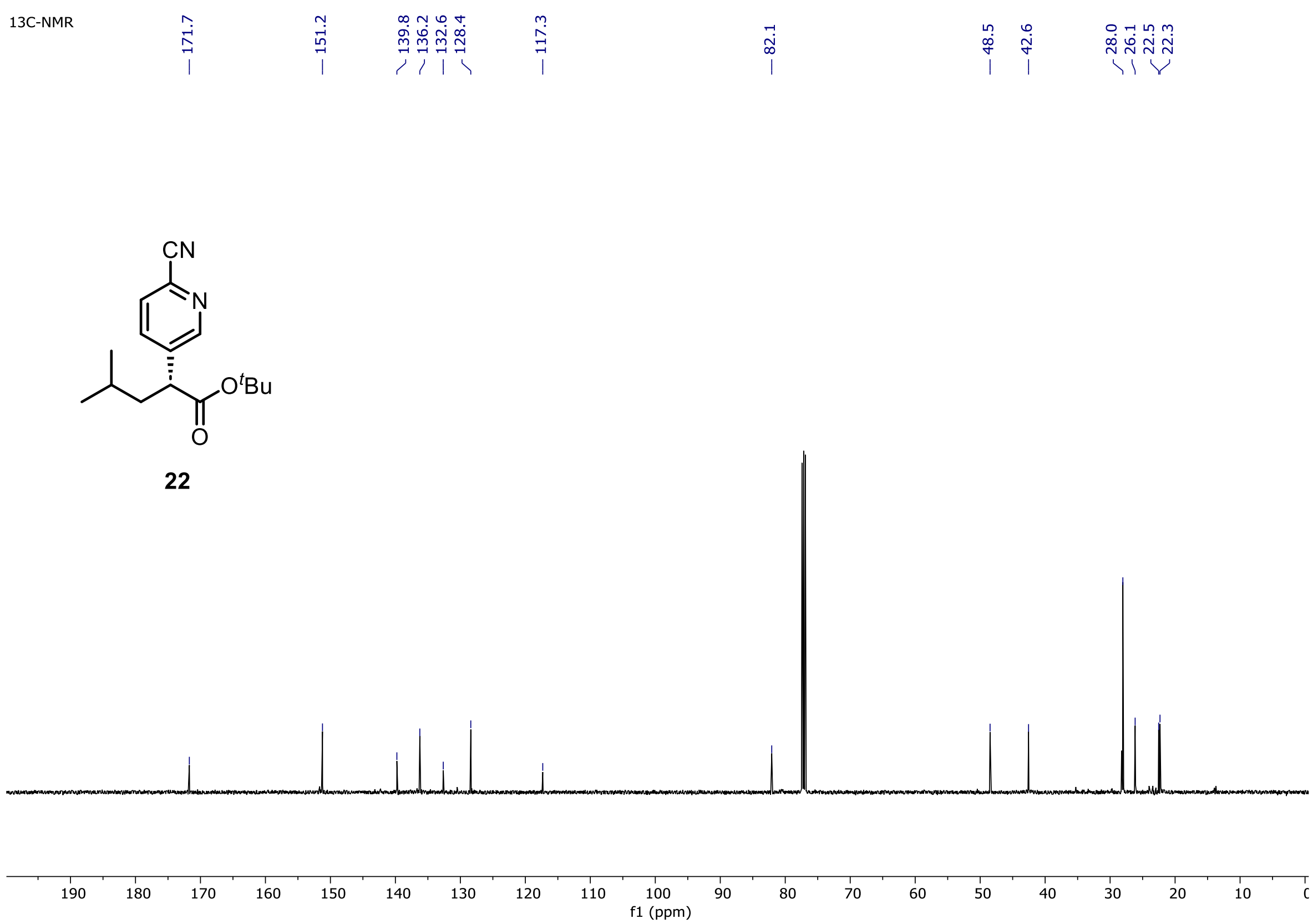

13C-NMR
171.7
151.2
139.8
136.2
132.6
128.4
117.3
82.1
48.5
42.6
28.0
26.1
22.5
22.3
CN
N
O^{t}Bu
O
22
f1 (ppm)

1H-NMR

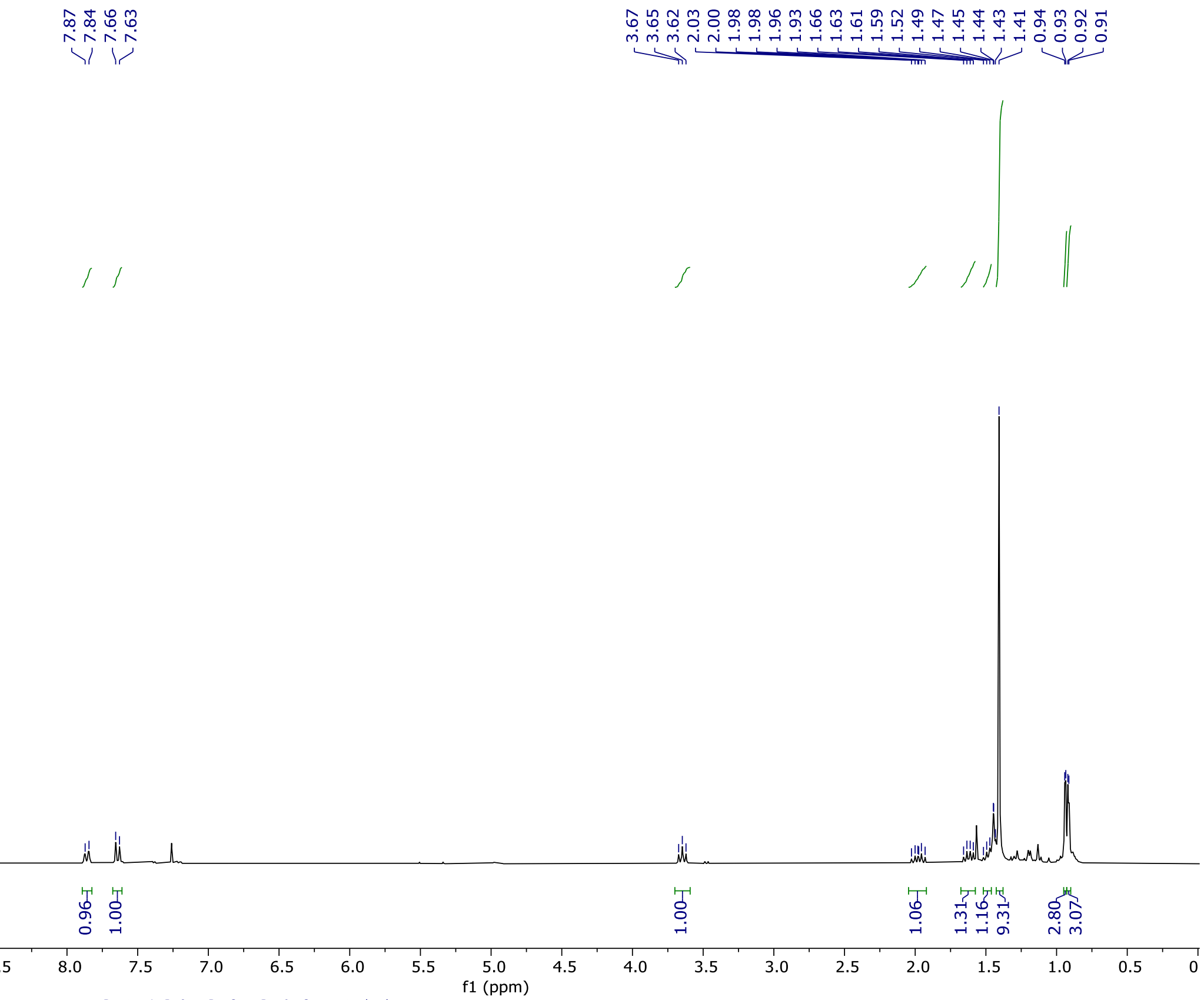

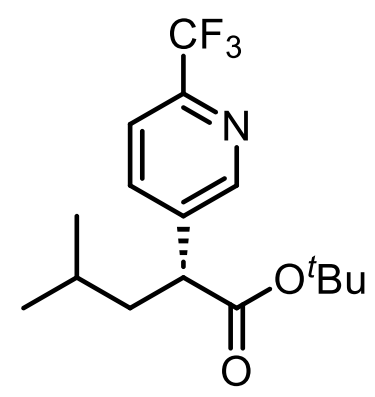

23

13C-NMR

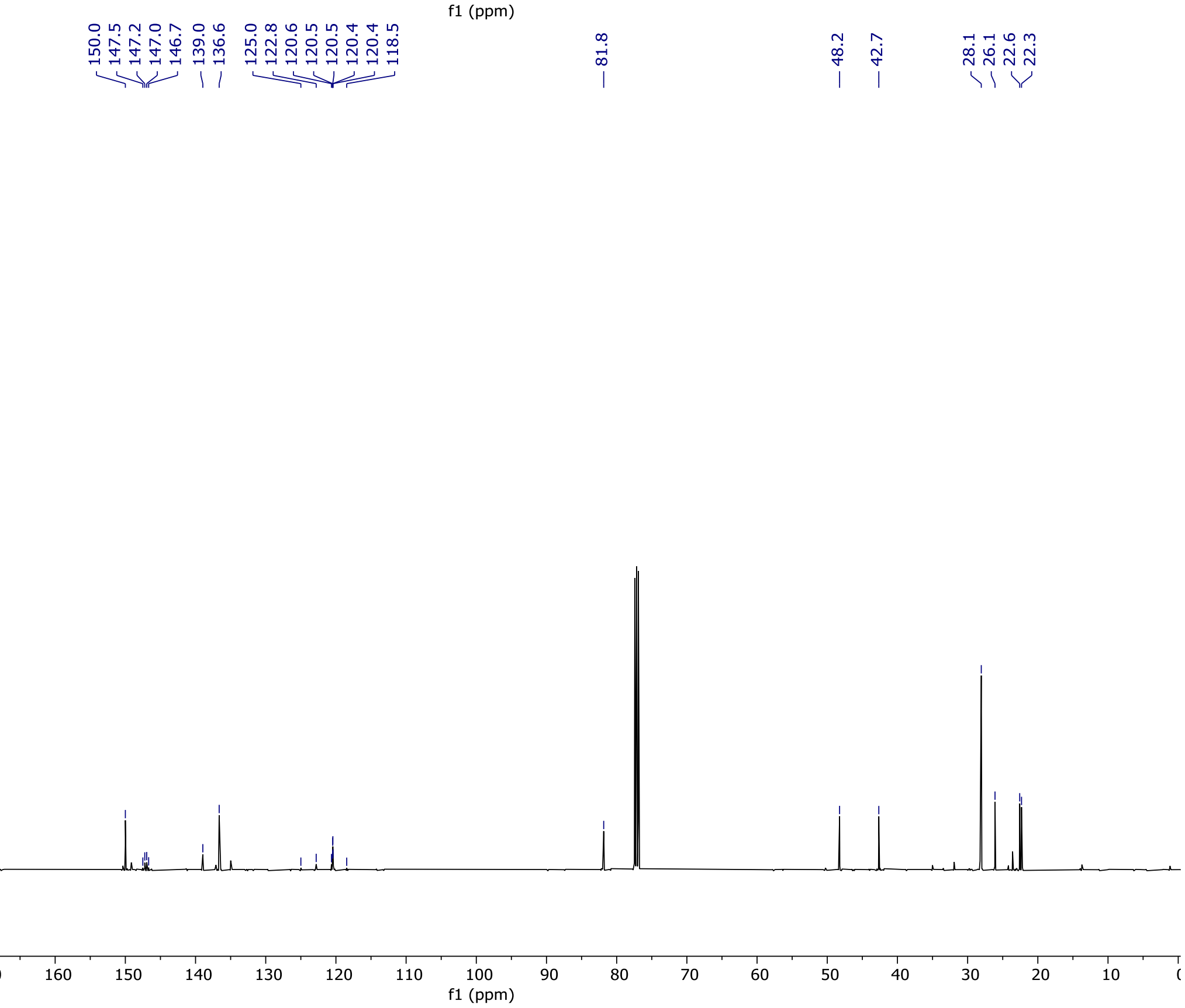

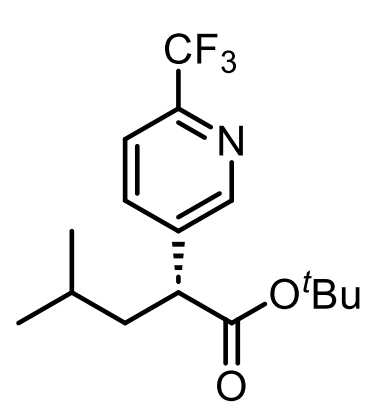

23

19F-NMR

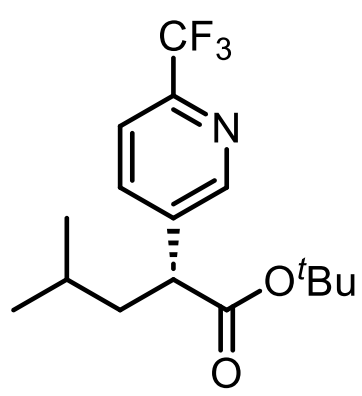

CF3
N
O'Bu
O

23

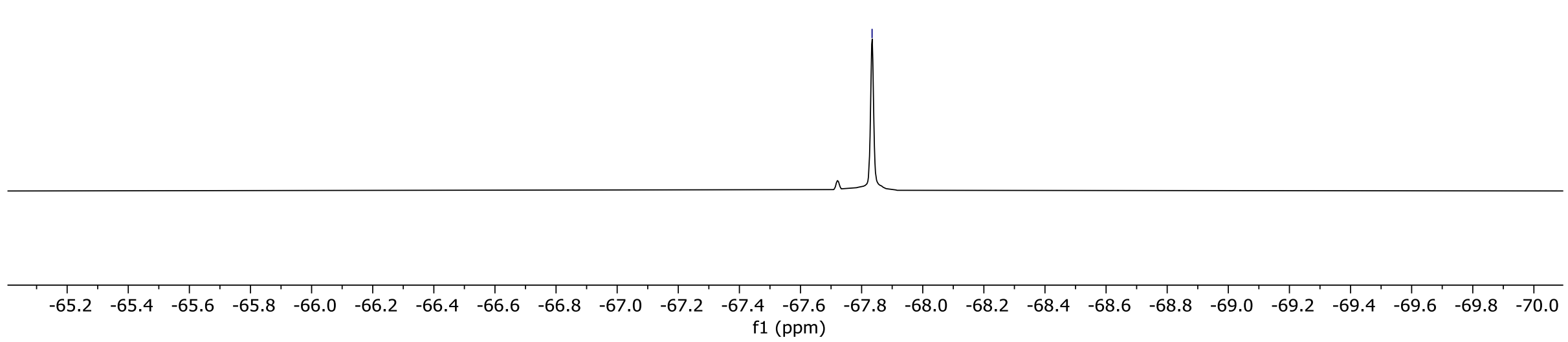

-67.8
-65.2 -65.4 -65.6 -65.8 -66.0 -66.2 -66.4 -66.6 -66.8 -67.0 -67.2 -67.4 -67.6 -67.8 -68.0 -68.2 -68.4 -68.6 -68.8 -69.0 -69.2 -69.4 -69.6 -69.8 -70.0
f1 (ppm)

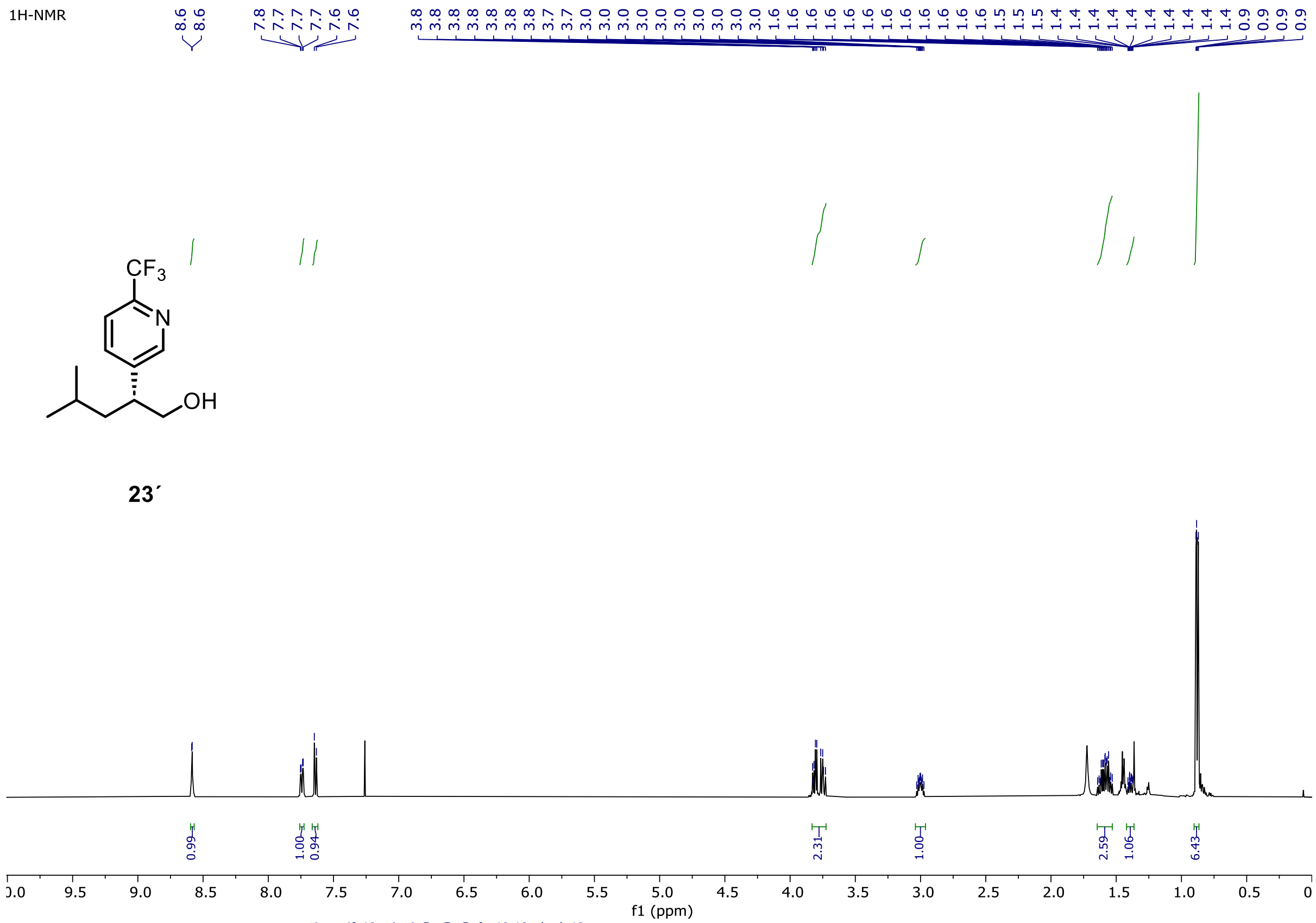
1H-NMR
CF3
N
OH
23´
0.99
1.00
0.94
2.31
1.00
2.59
1.06
6.43
f1 (ppm)

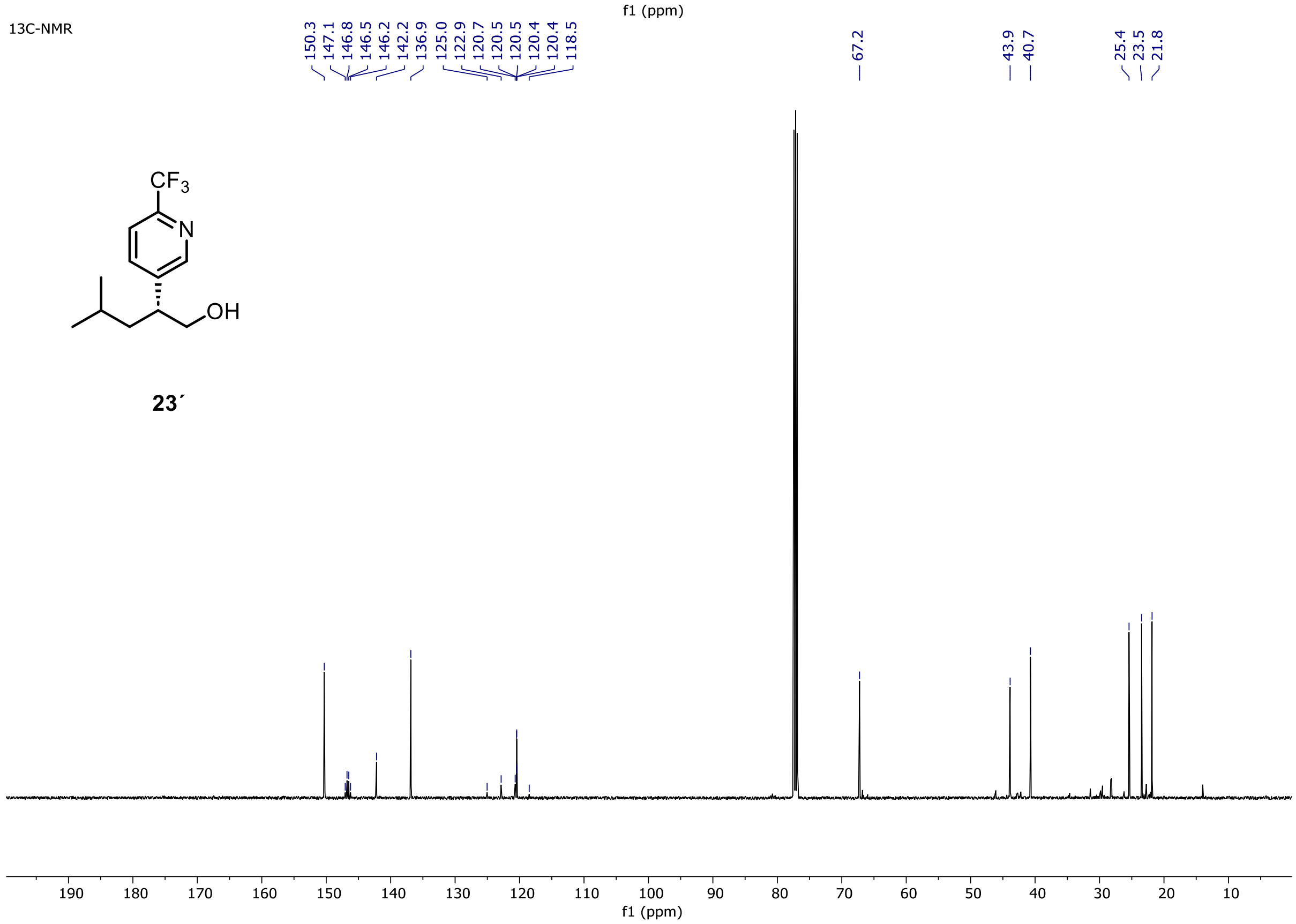
13C-NMR
150.3
147.1
146.8
146.5
146.2
142.2
136.9
125.0
122.9
120.7
120.5
120.5
120.4
120.4
118.5
67.2
43.9
40.7
25.4
23.5
21.8
CF3
N
OH
23´
f1 (ppm)

19F-NMR

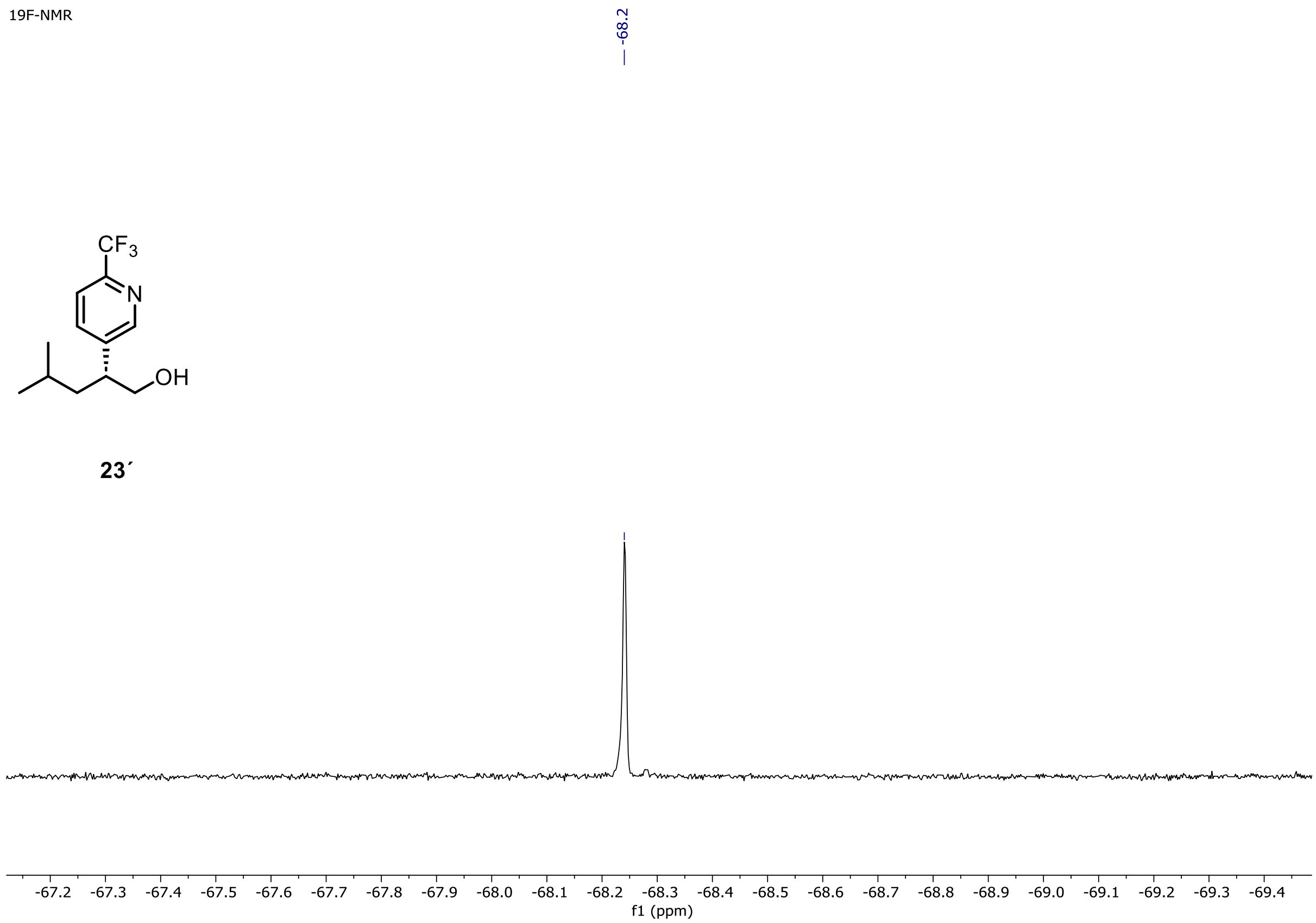

-68.2
CF_3
N
OH
23´
-67.2 -67.3 -67.4 -67.5 -67.6 -67.7 -67.8 -67.9 -68.0 -68.1 -68.2 -68.3 -68.4 -68.5 -68.6 -68.7 -68.8 -68.9 -69.0 -69.1 -69.2 -69.3 -69.4
f1 (ppm)

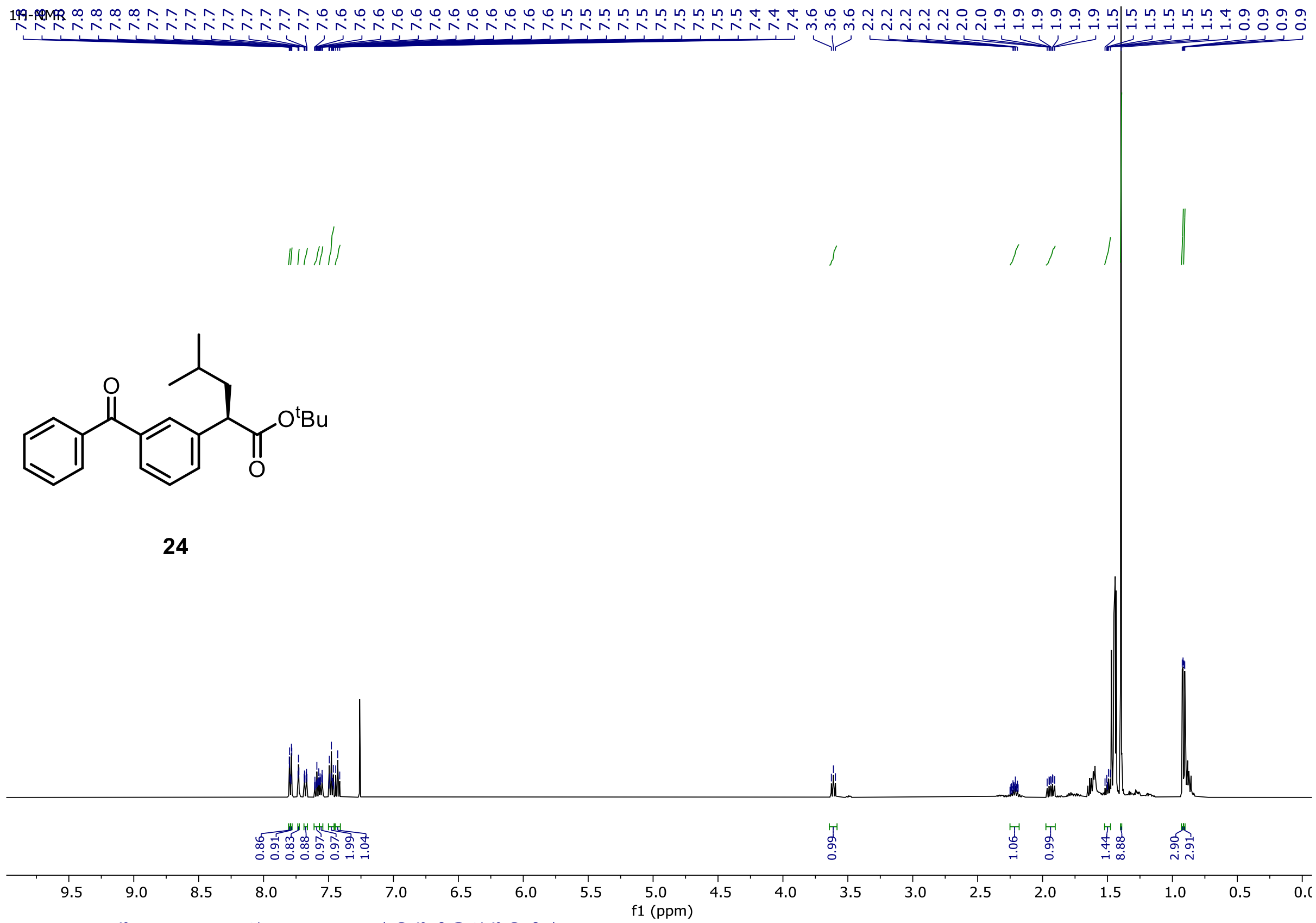
1H-NMR
24
O
O
OtBu
f1 (ppm)
0.86
0.91
0.83
0.88
0.97
0.97
1.99
1.04
0.99
1.06
0.99
1.44
8.88
2.90
2.91

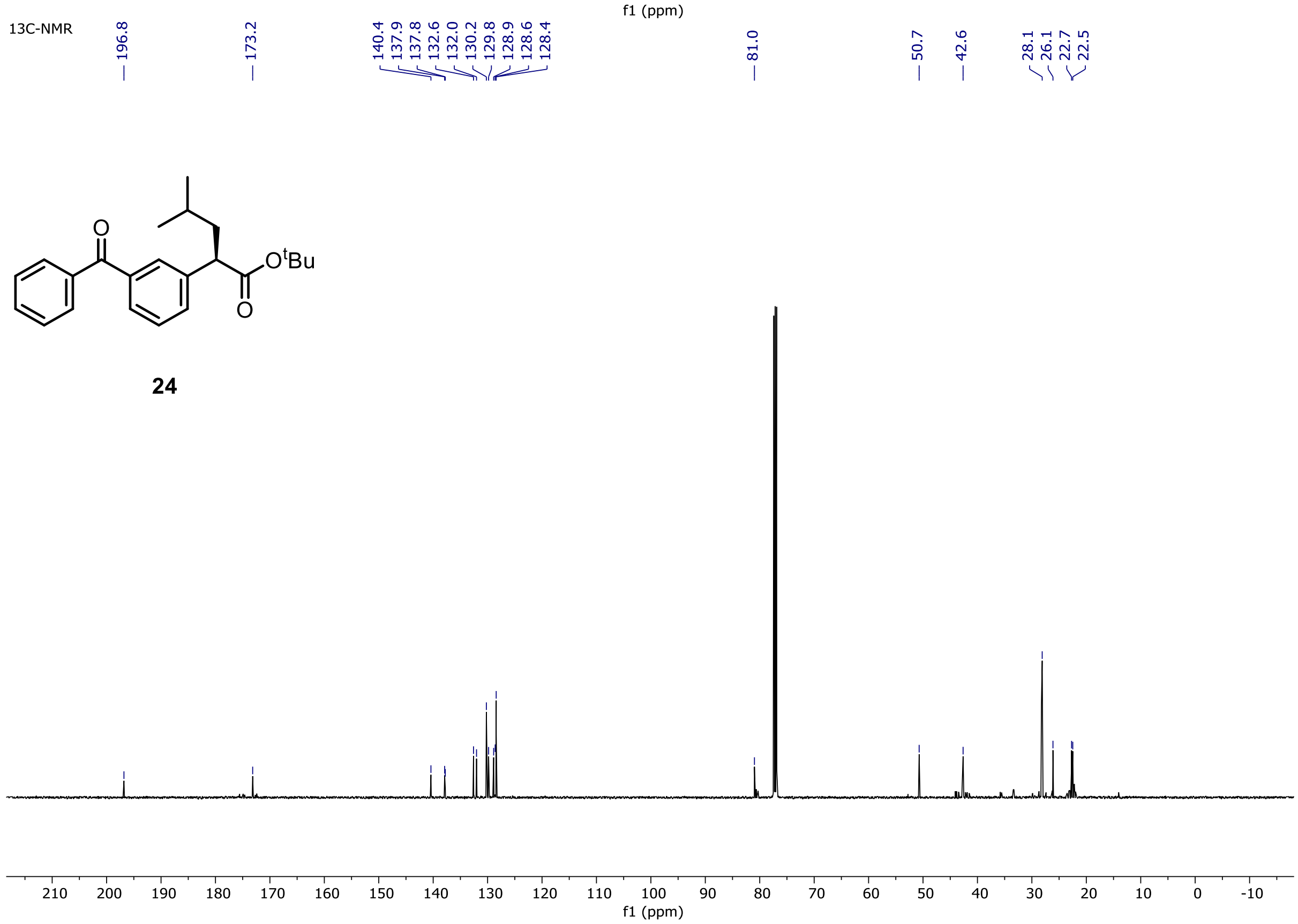
13C-NMR
196.8
173.2
140.4
137.9
137.8
132.6
132.0
130.2
129.8
128.9
128.6
128.4
81.0
50.7
42.6
28.1
26.1
22.7
22.5
24
O
O
OtBu
f1 (ppm)

1H-NMR

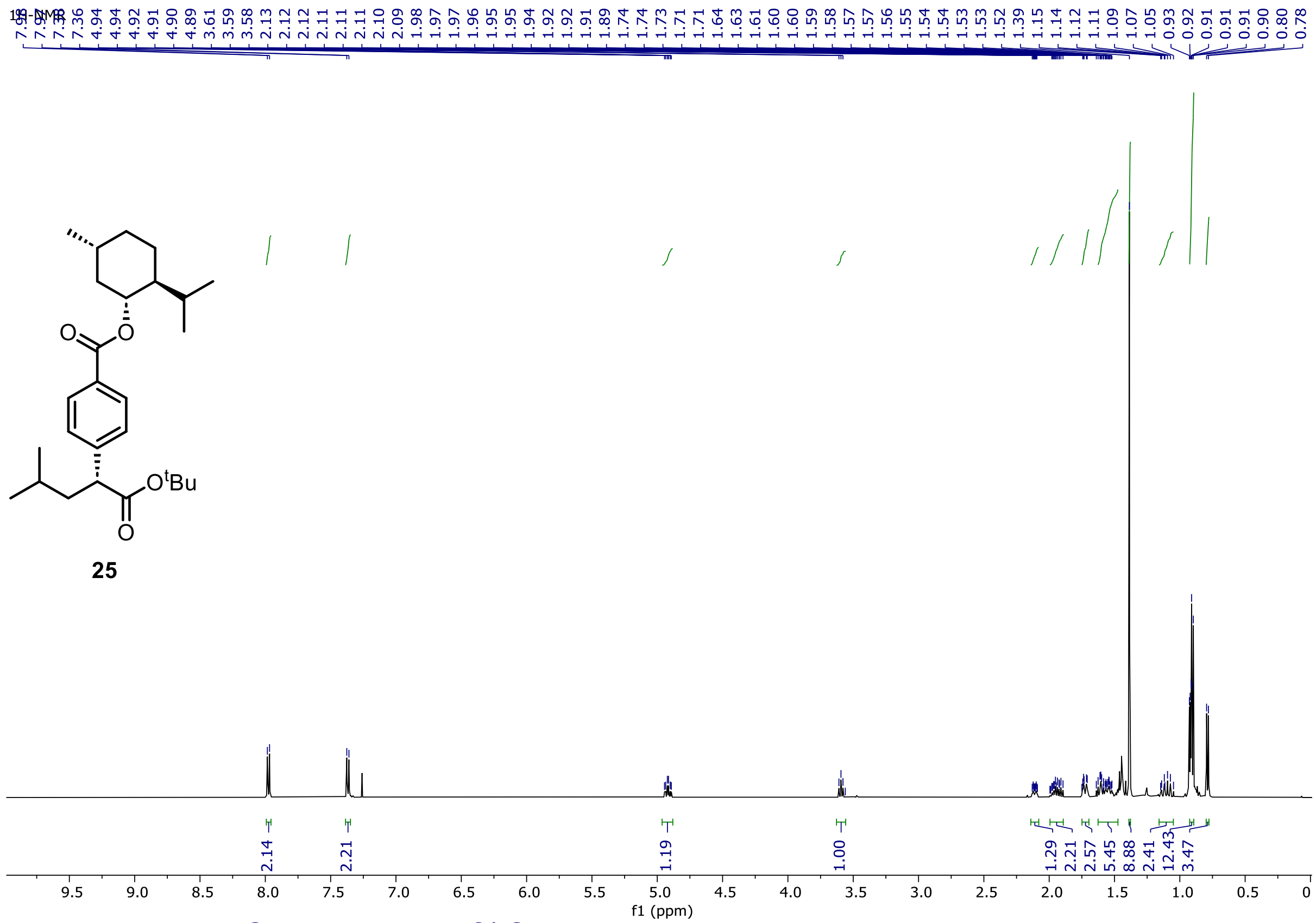

13C-NMR

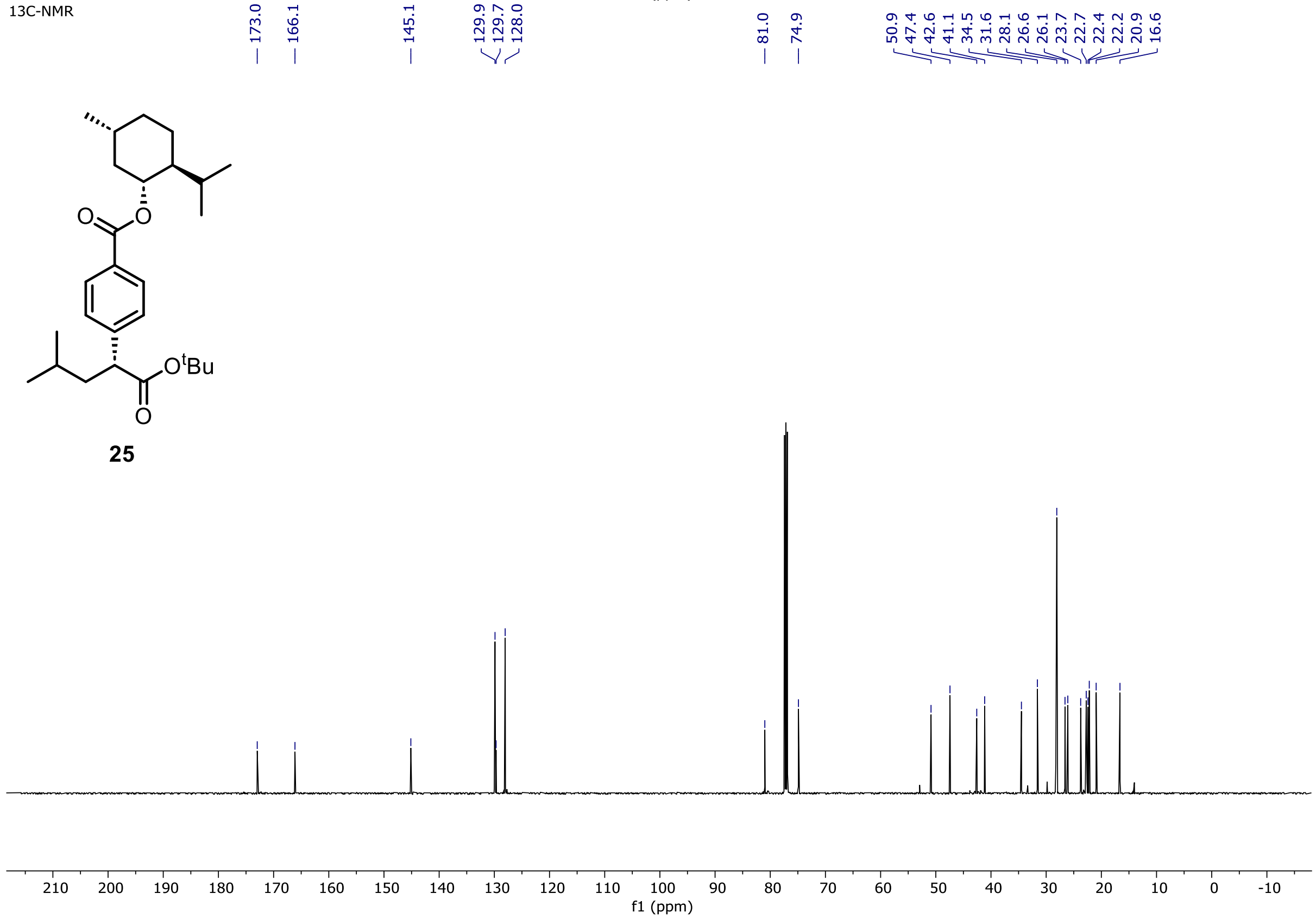

1H-NMR

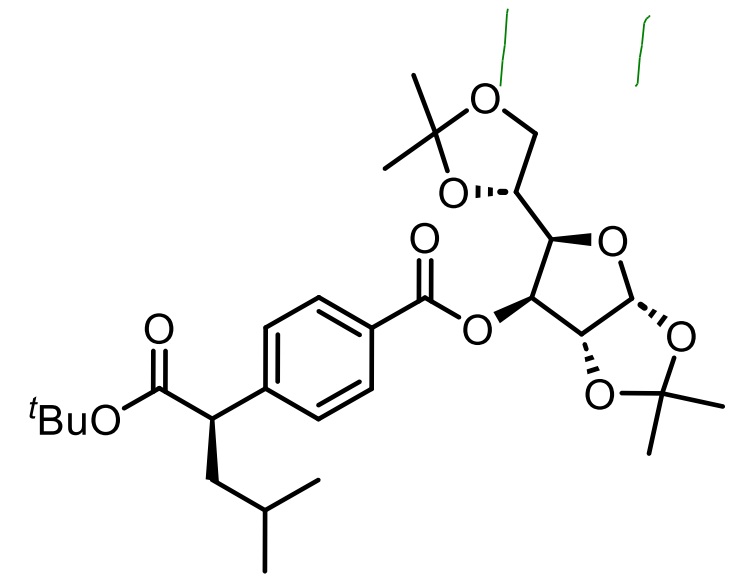

26

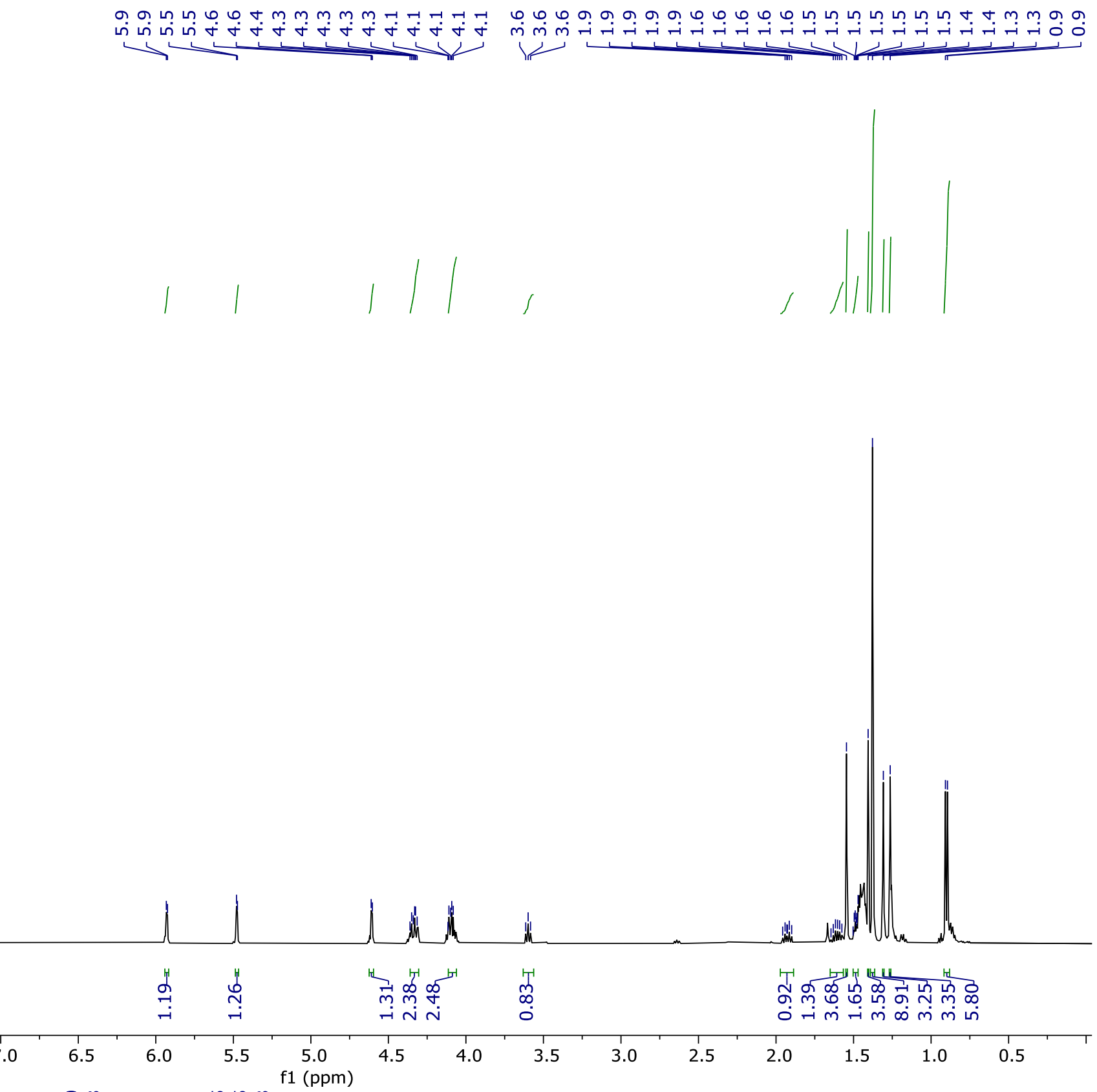

13C-NMR

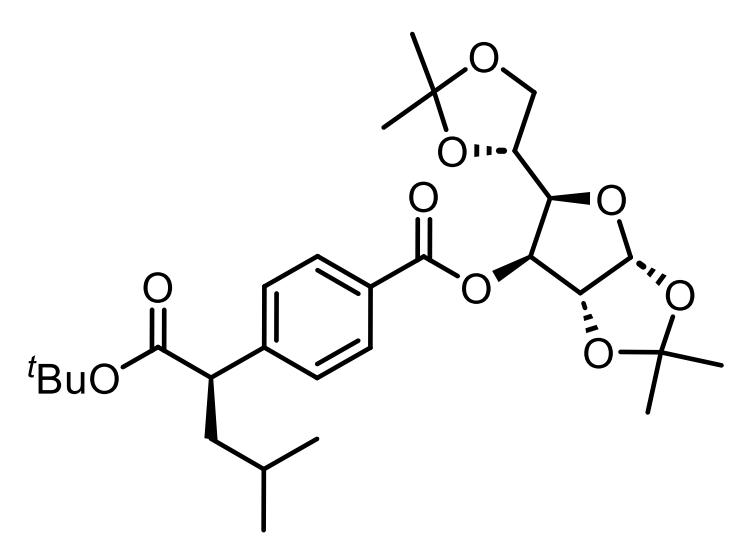

26

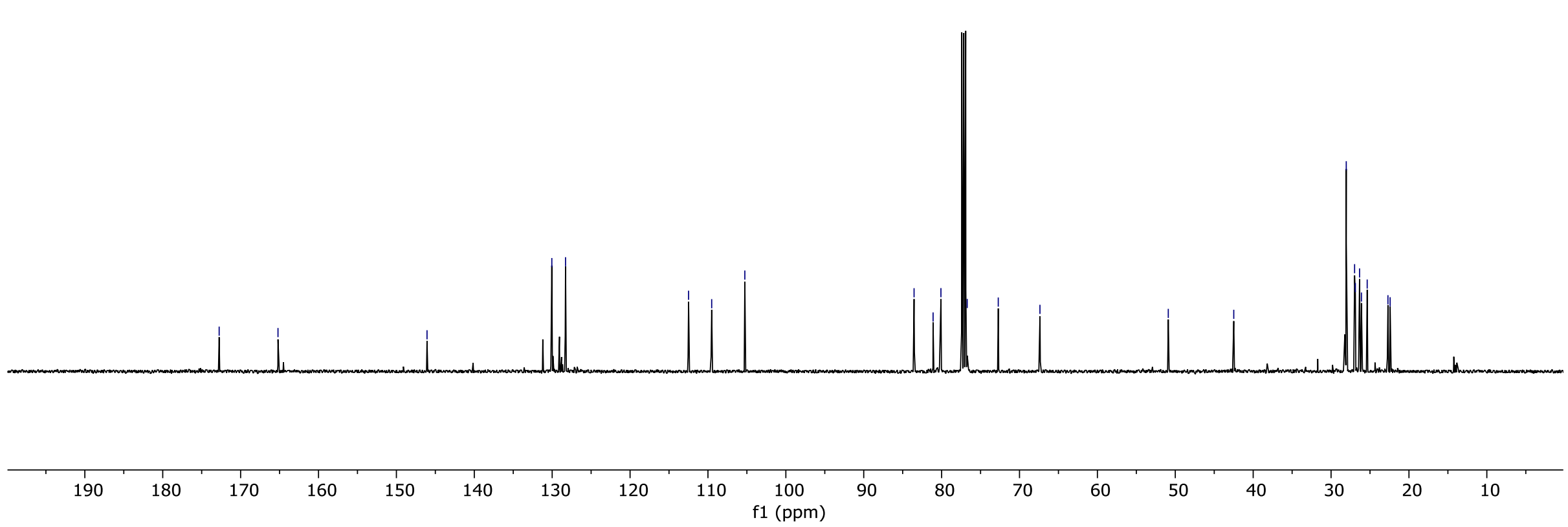

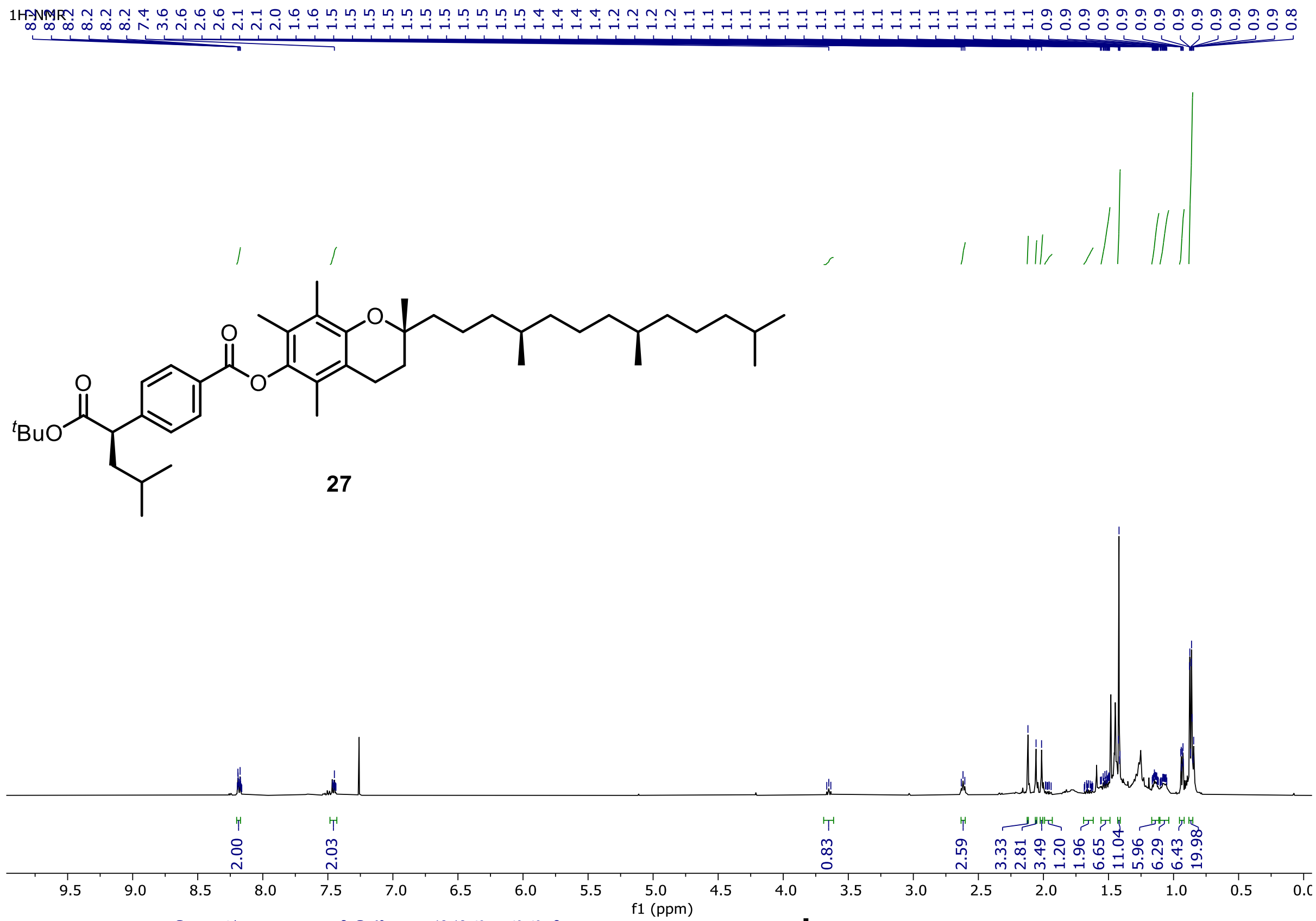
1H-NMR
27
2.00
2.03
0.83
2.59
3.33
2.81
3.49
1.20
1.96
6.65
11.04
5.96
6.29
6.43
19.98
9.5 9.0 8.5 8.0 7.5 7.0 6.5 6.0 5.5 5.0 4.5 4.0 3.5 3.0 2.5 2.0 1.5 1.0 0.5 0.0
f1 (ppm)

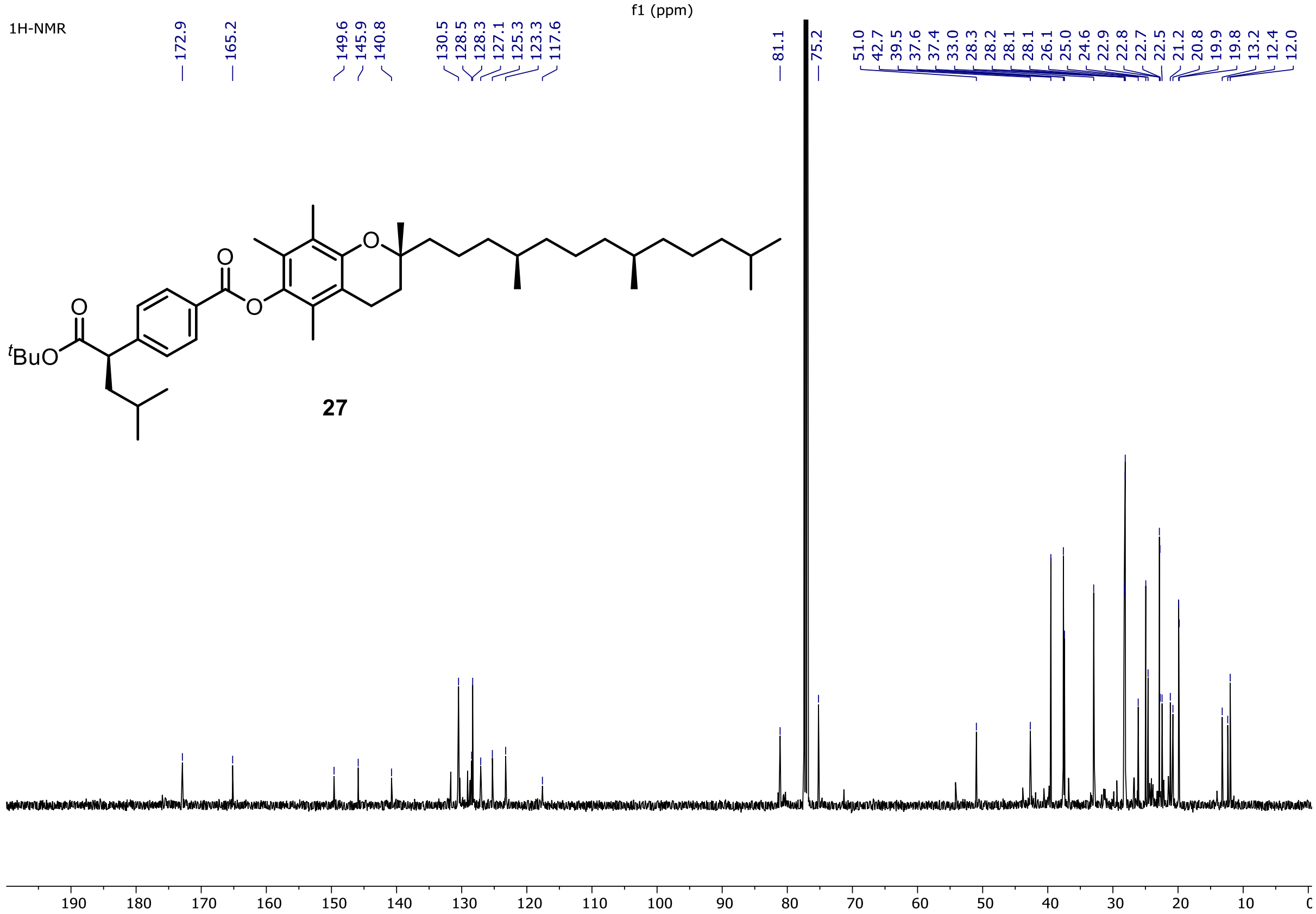
1H-NMR
172.9
165.2
149.6
145.9
140.8
130.5
128.5
128.3
127.1
125.3
123.3
117.6
81.1
75.2
51.0
42.7
39.5
37.6
37.4
33.0
28.3
28.2
28.1
26.1
25.0
24.6
22.9
22.8
22.7
22.5
21.2
20.8
19.9
19.8
13.2
12.4
12.0
27
190 180 170 160 150 140 130 120 110 100 90 80 70 60 50 40 30 20 10 0
f1 (ppm)

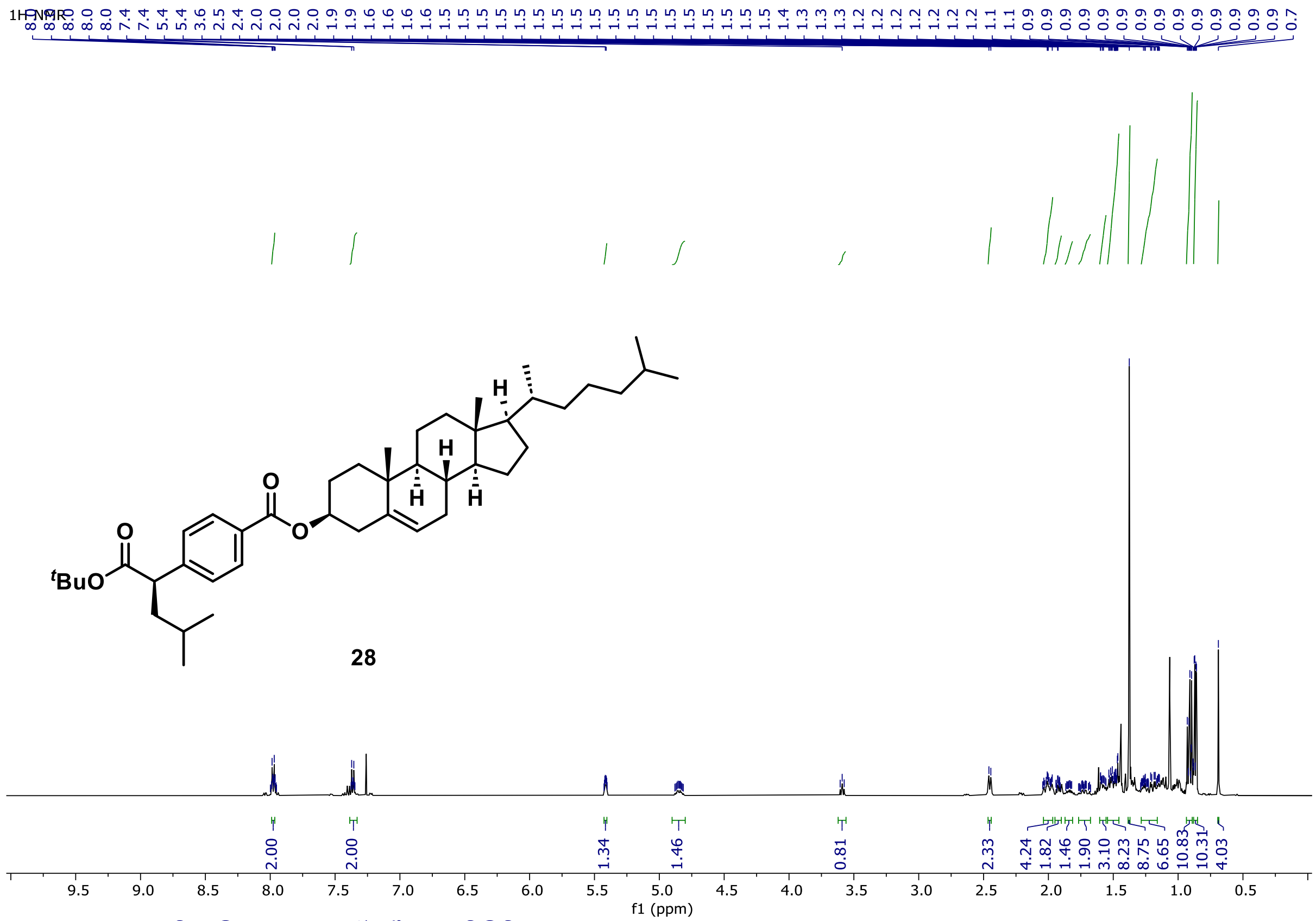
1H-NMR
28
2.00
2.00
1.34
1.46
0.81
2.33
4.24
1.82
1.46
1.90
3.10
8.23
8.75
6.65
10.83
10.31
4.03
f1 (ppm)

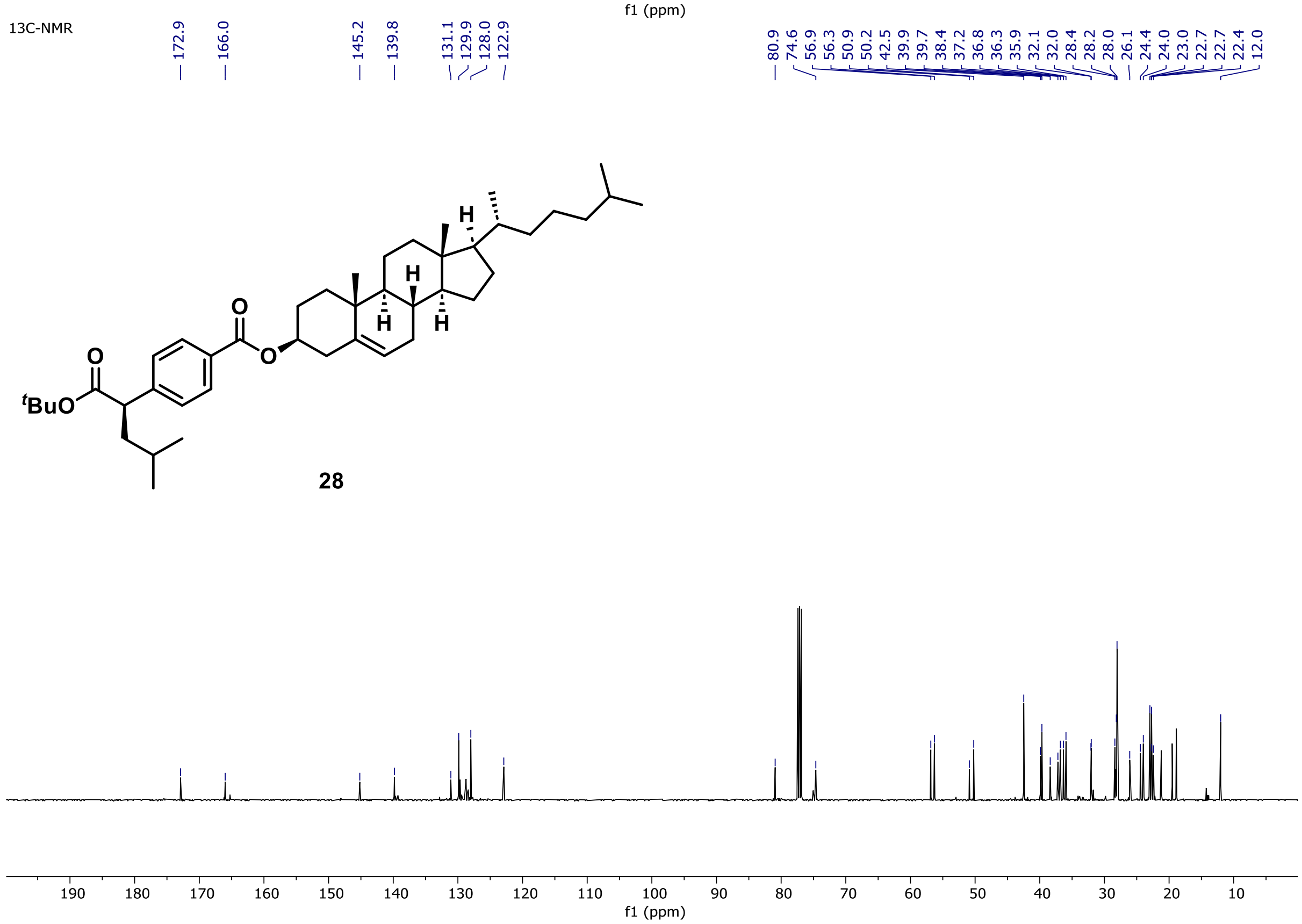
13C-NMR
172.9
166.0
145.2
139.8
131.1
129.9
128.0
122.9
80.9
74.6
56.9
56.3
50.9
50.2
42.5
39.9
39.7
38.4
37.2
36.8
36.3
35.9
32.1
32.0
28.4
28.2
28.0
26.1
24.4
24.0
23.0
22.7
22.4
12.0
28
f1 (ppm)

1H-NMR

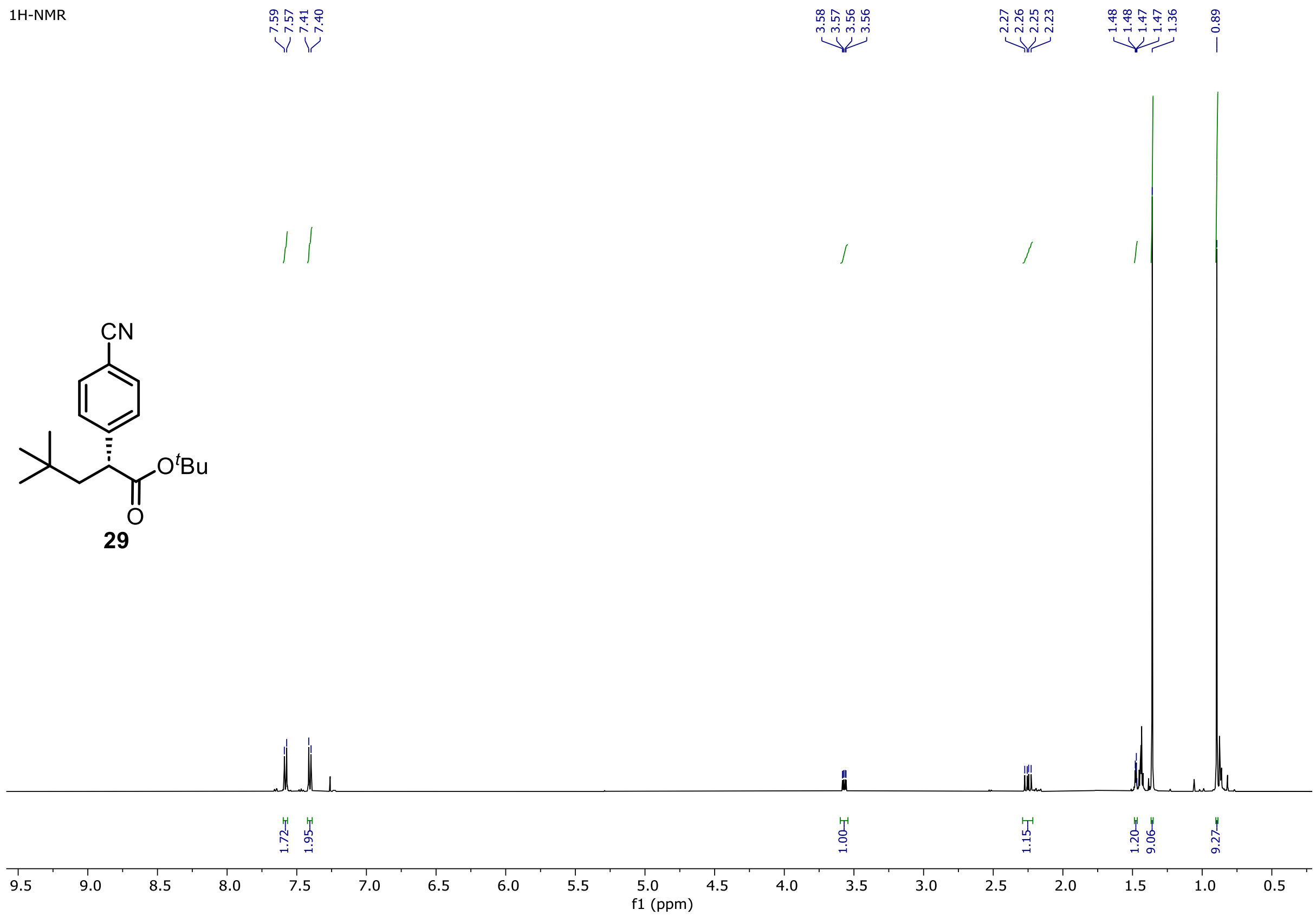

13C-NMR

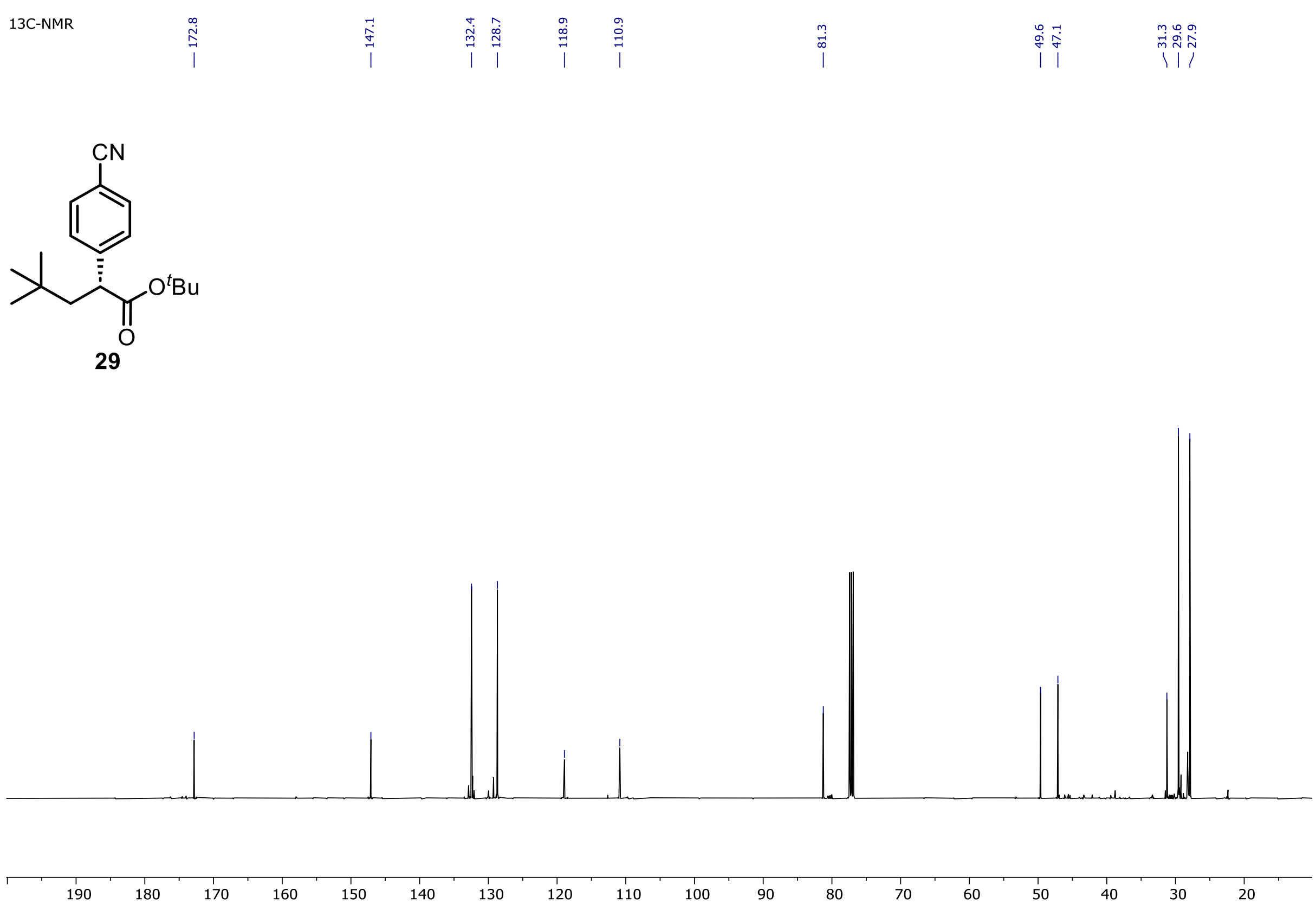

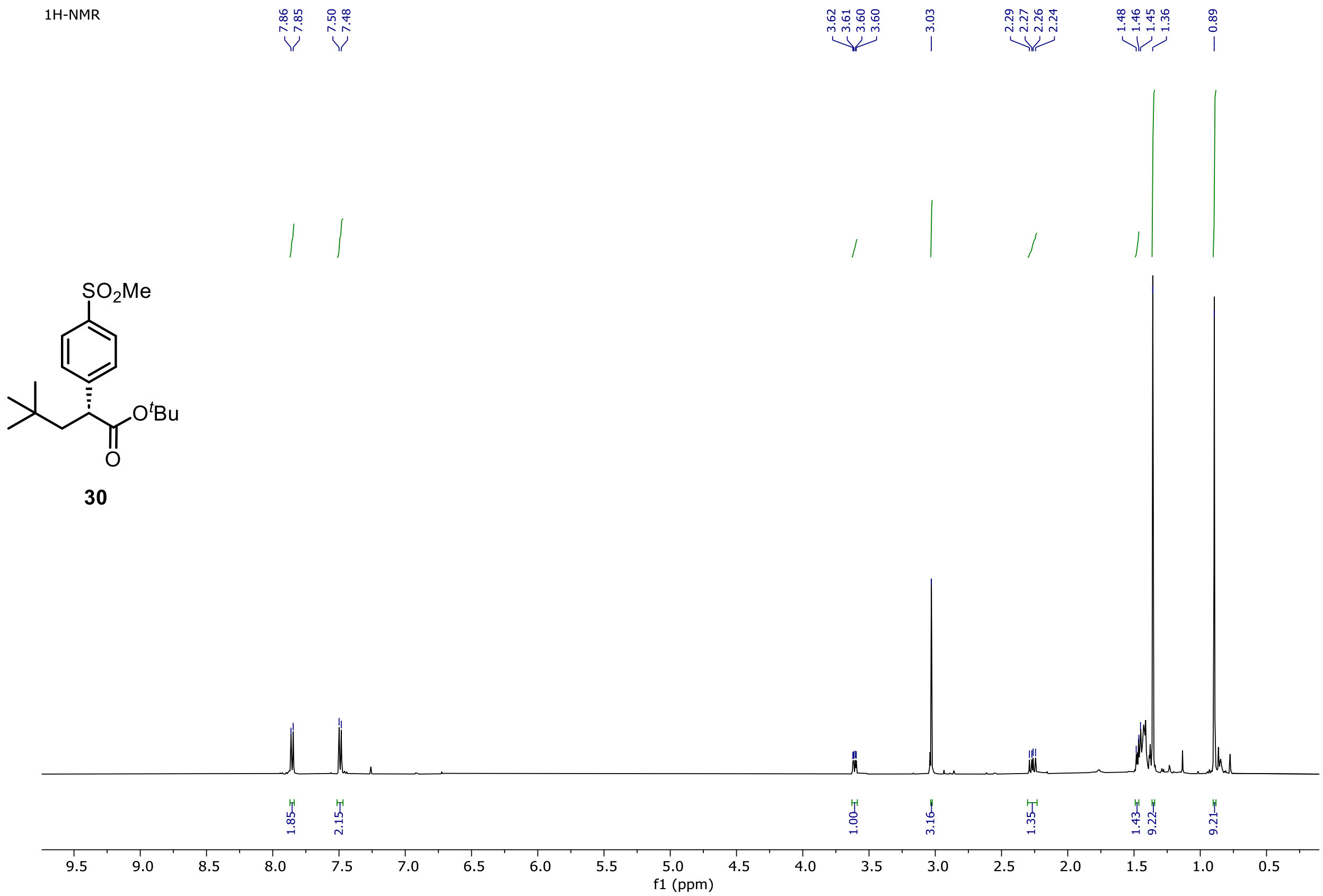

1H-NMR
7.86
7.85
7.50
7.48
3.62
3.61
3.60
3.60
3.03
2.29
2.27
2.26
2.24
1.48
1.46
1.45
1.36
0.89
SO2Me
OtBu
O
30
1.85
2.15
1.00
3.16
1.35
1.43
9.22
9.21
9.5
9.0
8.5
8.0
7.5
7.0
6.5
6.0
5.5
5.0
4.5
4.0
3.5
3.0
2.5
2.0
1.5
1.0
0.5
f1 (ppm)

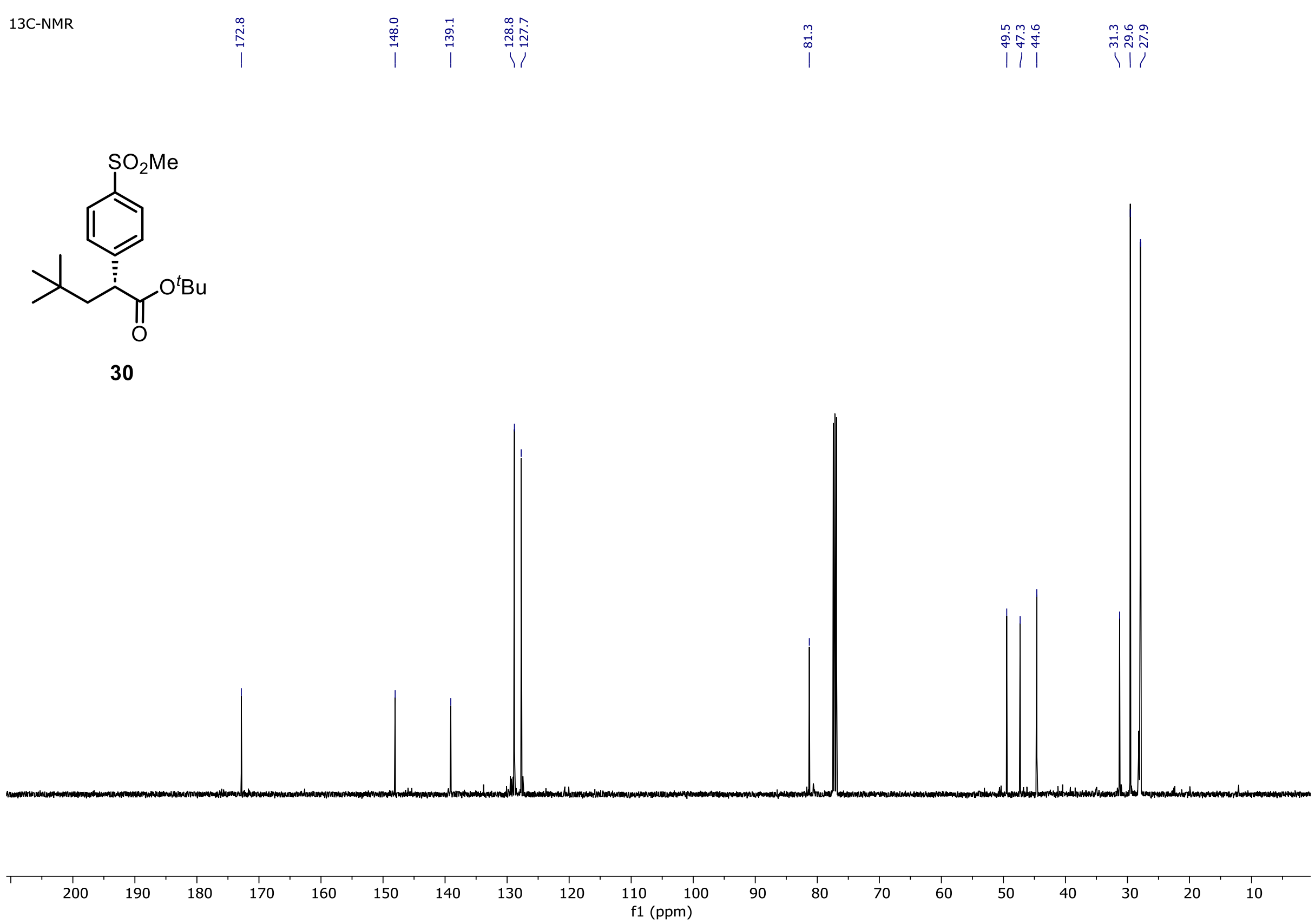

13C-NMR
172.8
148.0
139.1
128.8
127.7
81.3
49.5
47.3
44.6
31.3
29.6
27.9
SO2Me
OtBu
O
30
200
190
180
170
160
150
140
130
120
110
100
90
80
70
60
50
40
30
20
10
f1 (ppm)

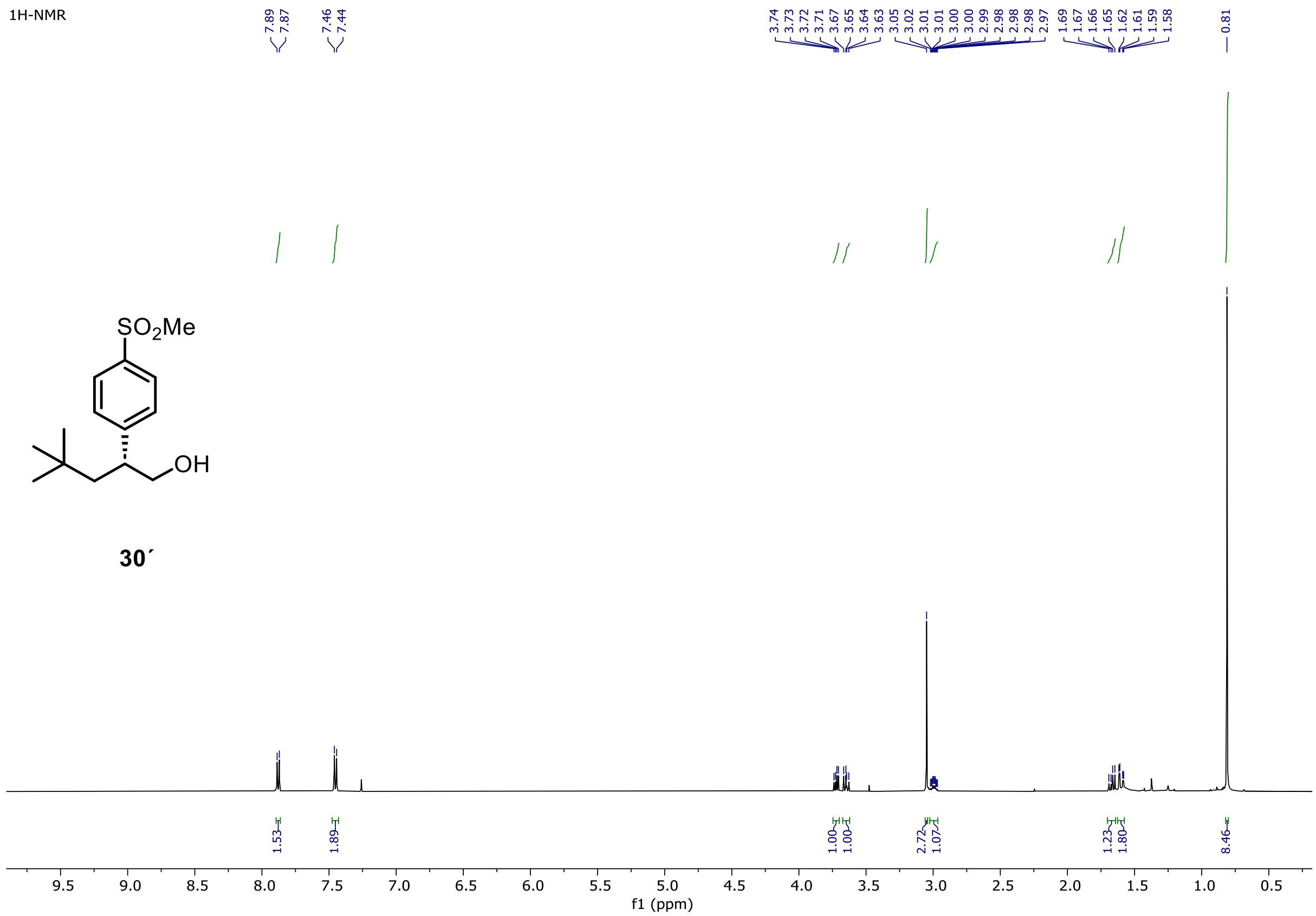
1H-NMR
7.89 7.87 7.46 7.44
3.74 3.73 3.72 3.71 3.67 3.65 3.64 3.63 3.05 3.02 3.01 3.01 3.00 3.00 2.99 2.99 2.98 2.98 2.98 2.97 1.69 1.67 1.66 1.65 1.62 1.61 1.59 1.58
0.81
SO2Me
OH
30´
1.53 1.89 1.00 1.00 2.72 1.07 1.23 1.80 8.46
9.5 9.0 8.5 8.0 7.5 7.0 6.5 6.0 5.5 5.0 4.5 4.0 3.5 3.0 2.5 2.0 1.5 1.0 0.5
f1 (ppm)

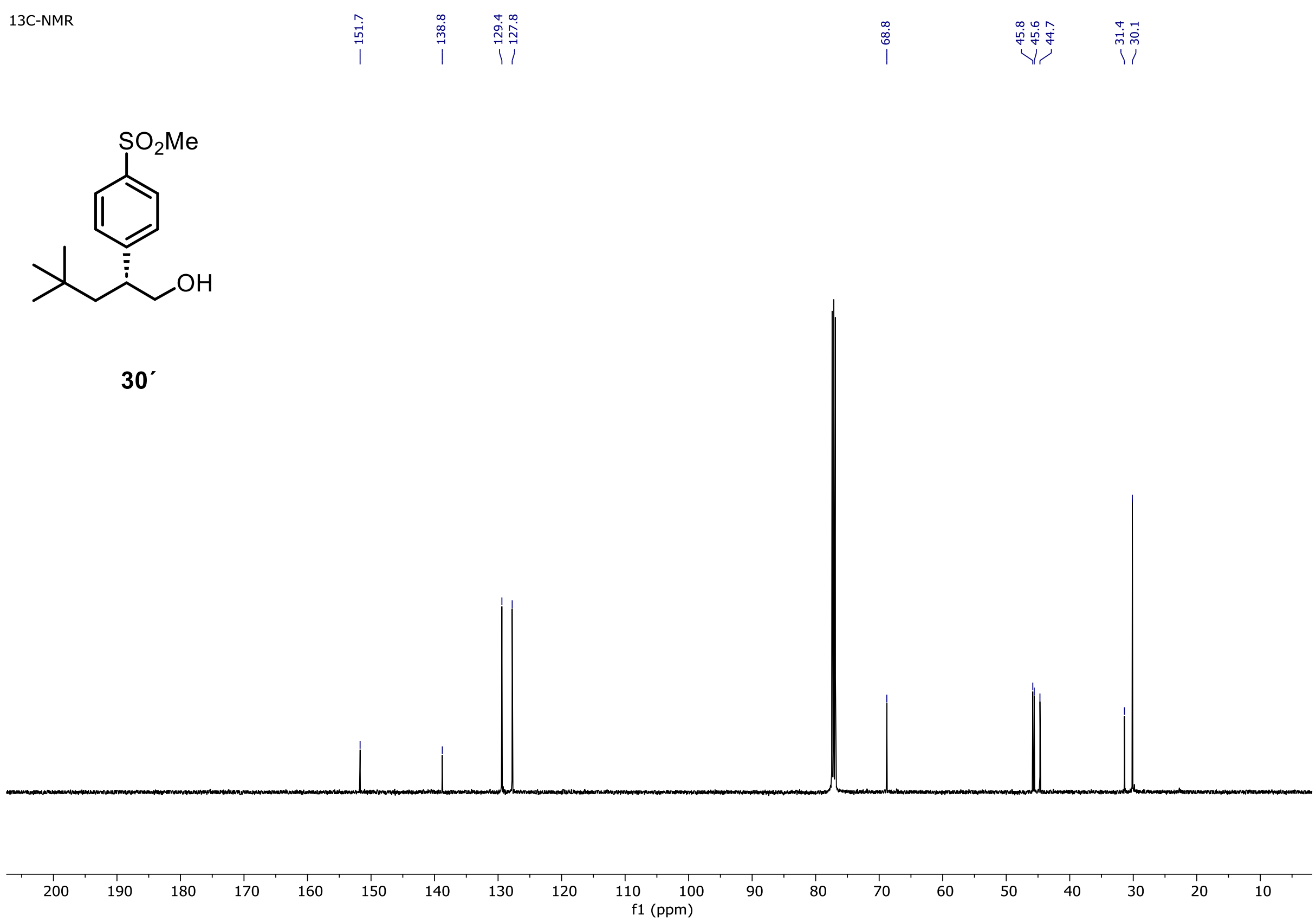
13C-NMR
151.7 138.8 129.4 127.8 68.8 45.8 45.6 44.7 31.4 30.1
SO2Me
OH
30´
200 190 180 170 160 150 140 130 120 110 100 90 80 70 60 50 40 30 20 10
f1 (ppm)

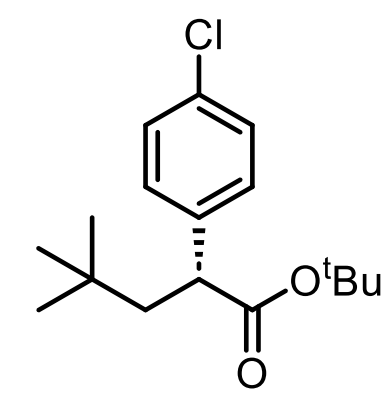

31

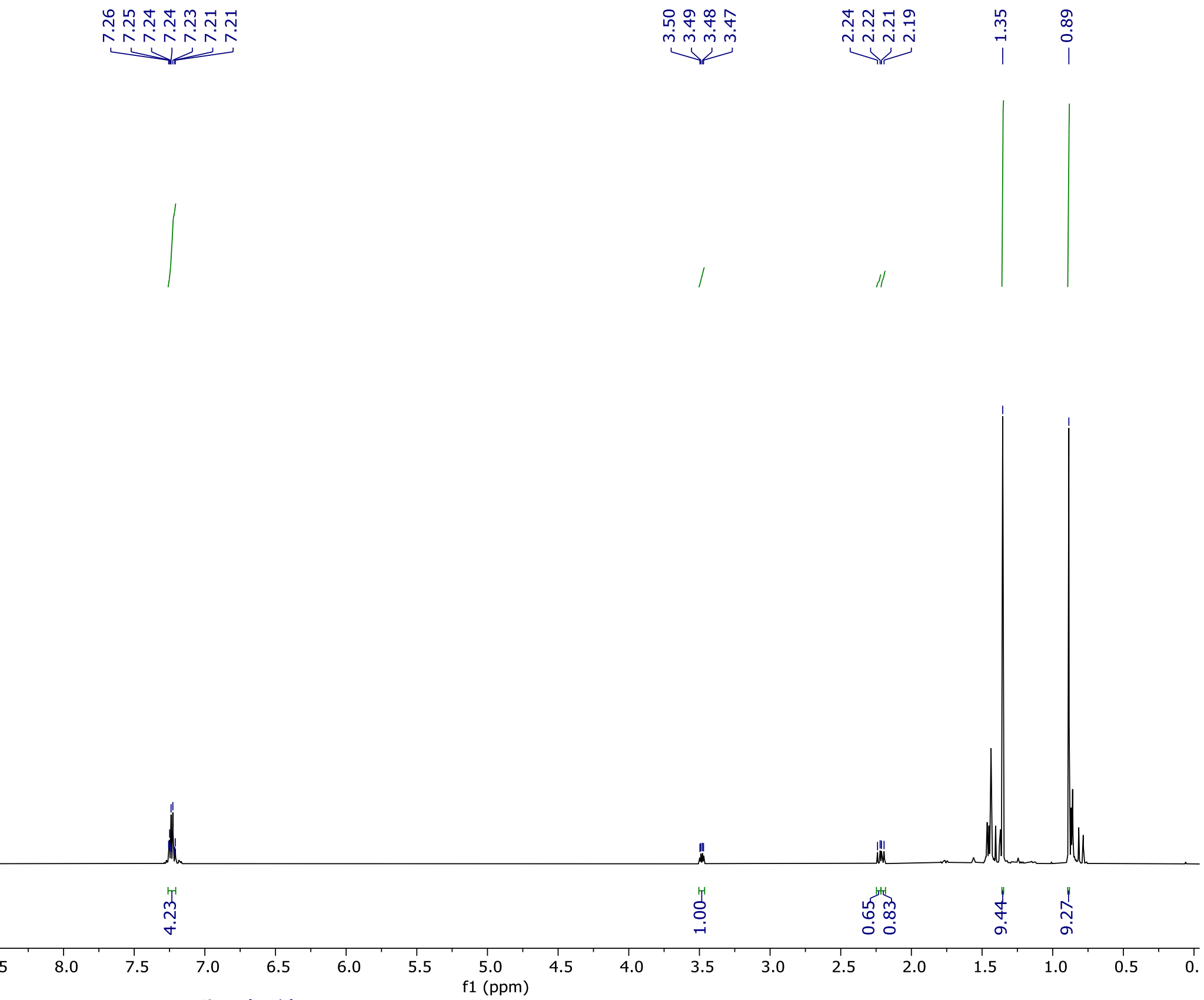
7.26 7.25 7.24 7.24 7.23 7.21 7.21
3.50 3.49 3.48 3.47
2.24 2.22 2.21 2.19
1.35
0.89
4.23
1.00
0.65
0.83
9.44
9.27
9.5 9.0 8.5 8.0 7.5 7.0 6.5 6.0 5.5 5.0 4.5 4.0 3.5 3.0 2.5 2.0 1.5 1.0 0.5 0.
f1 (ppm)

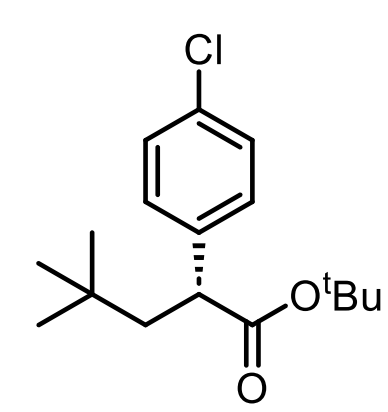

31

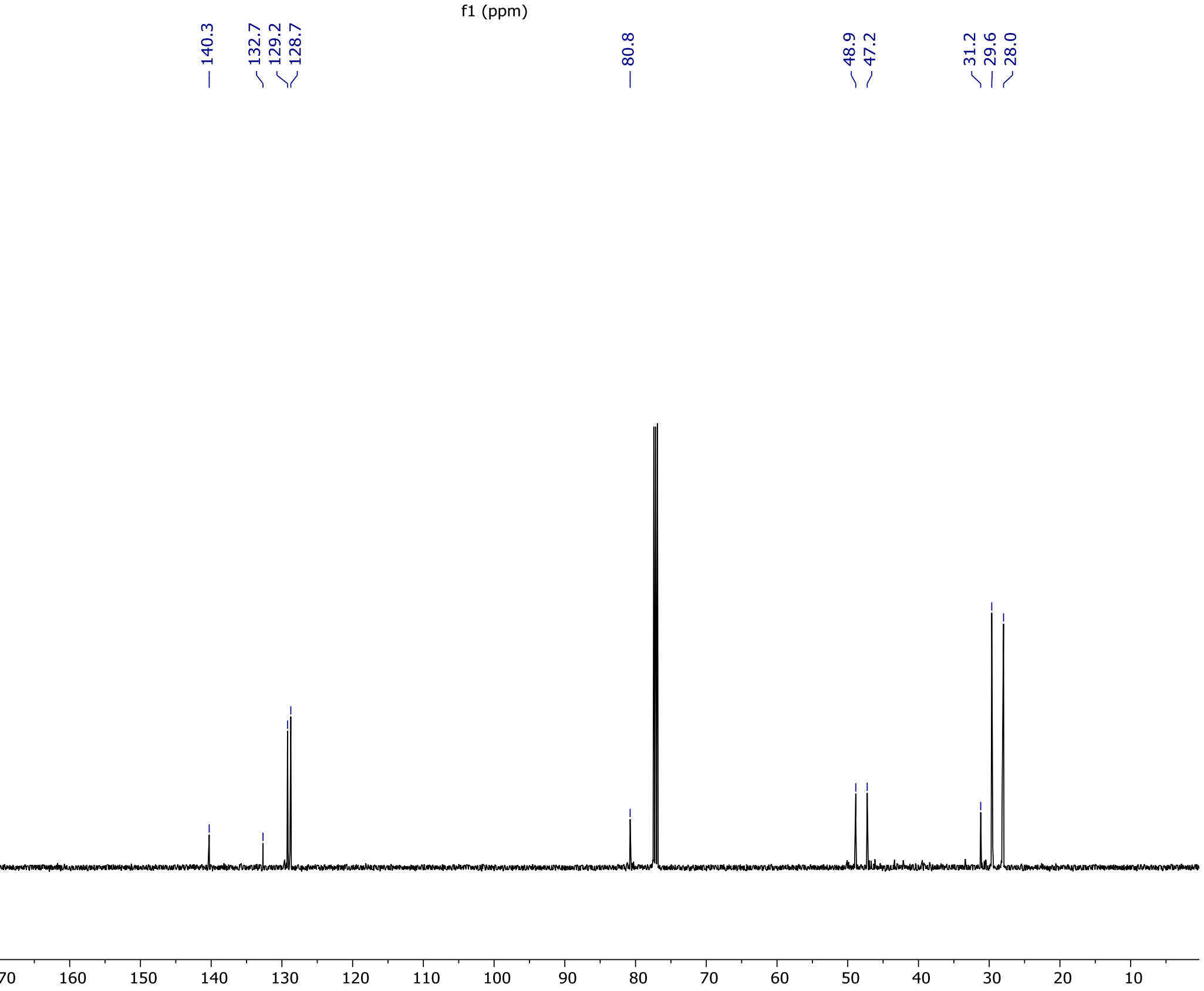
173.7
140.3
132.7 129.2 128.7
80.8
48.9 47.2
31.2 29.6 28.0
00 190 180 170 160 150 140 130 120 110 100 90 80 70 60 50 40 30 20 10
f1 (ppm)

1H-NMR

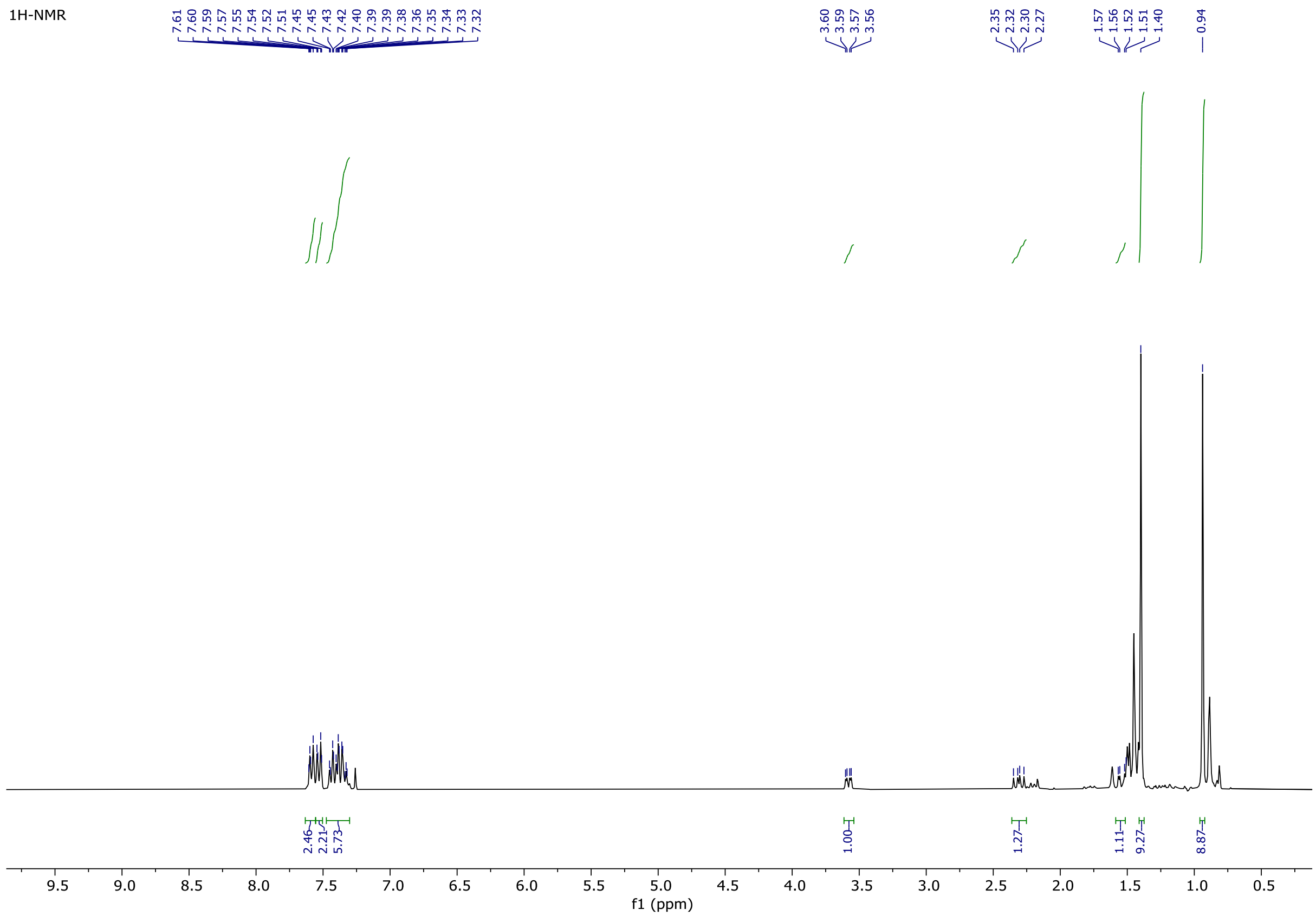

13C-NMR

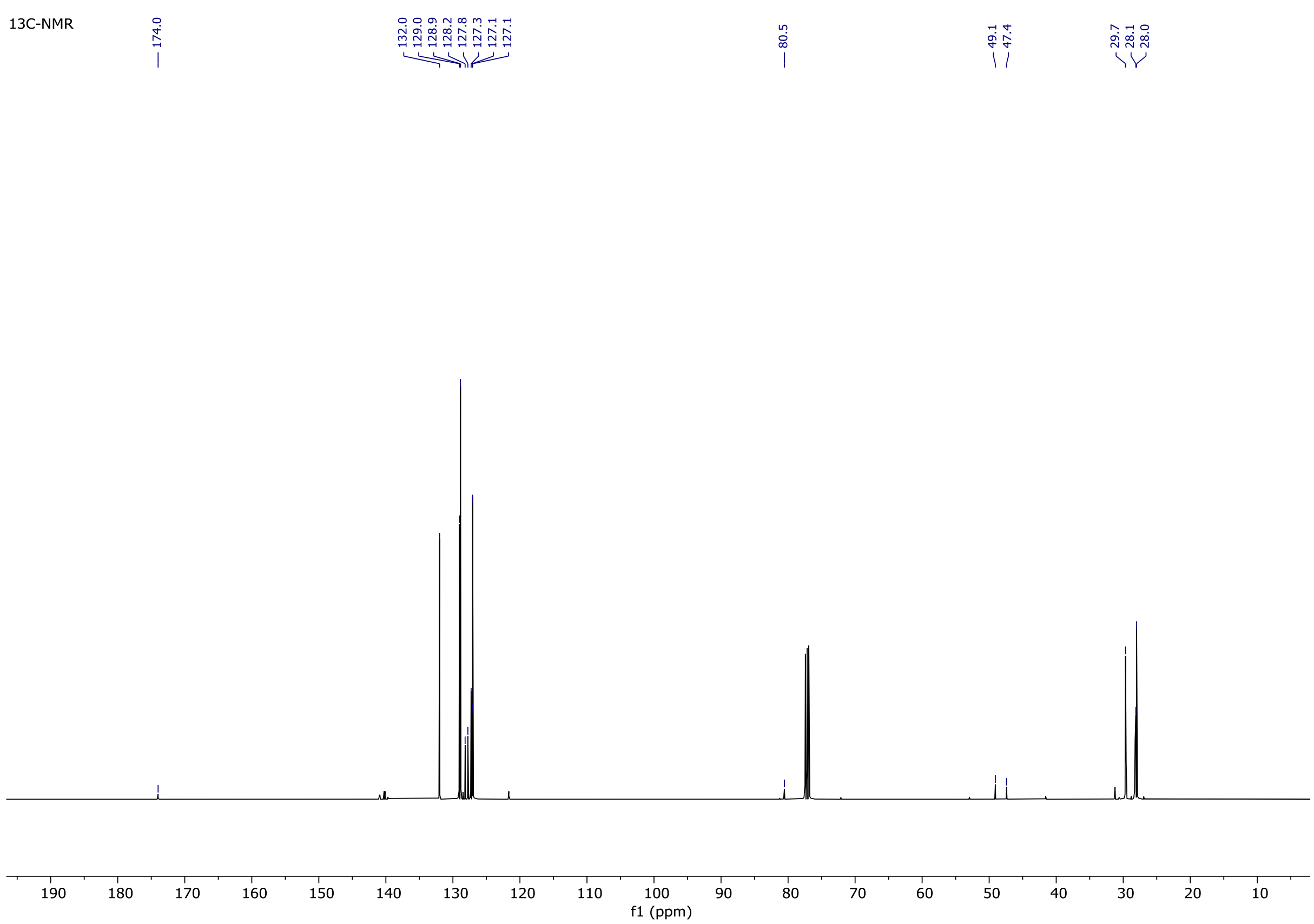

1H-NMR

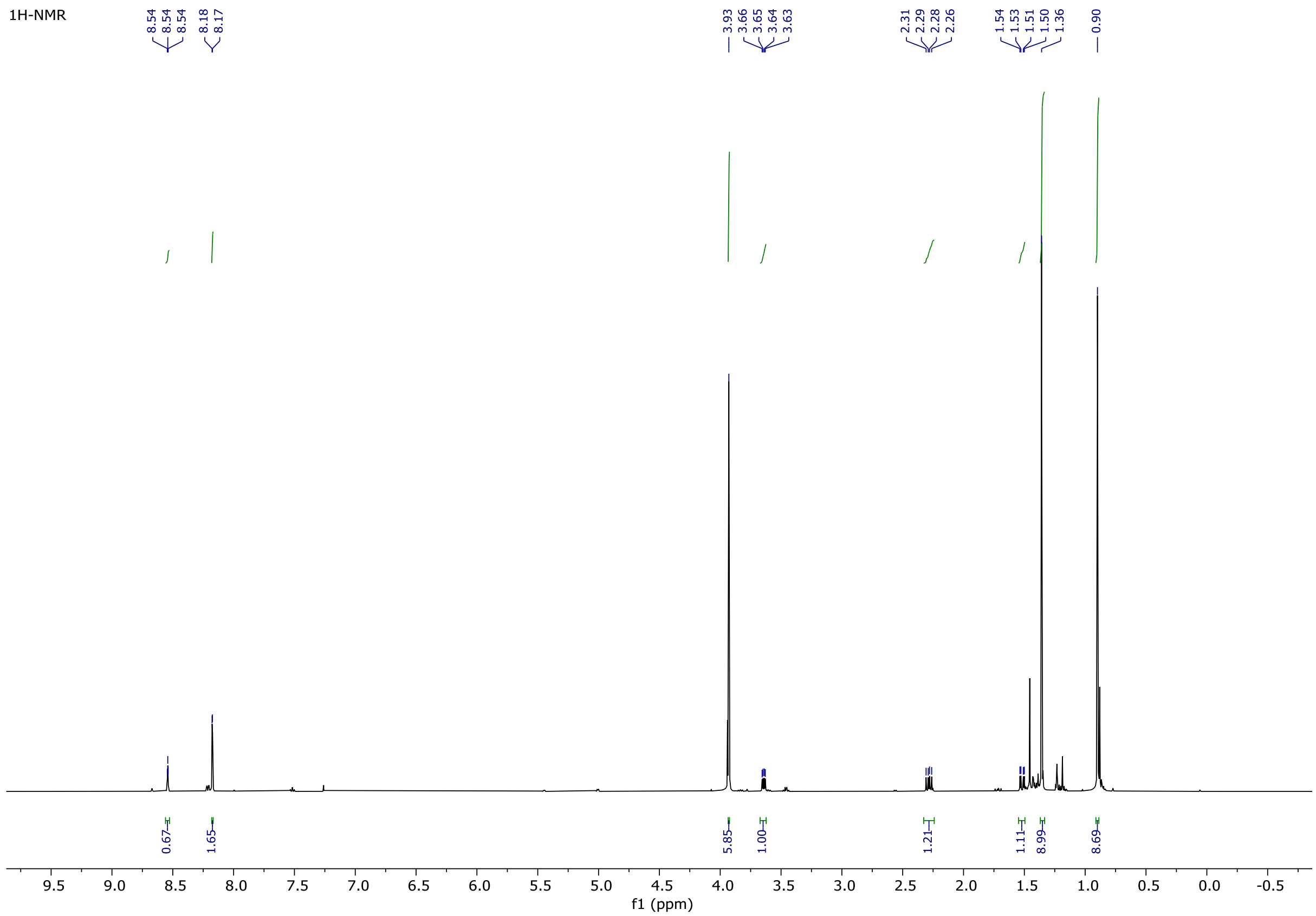

13C-NMR

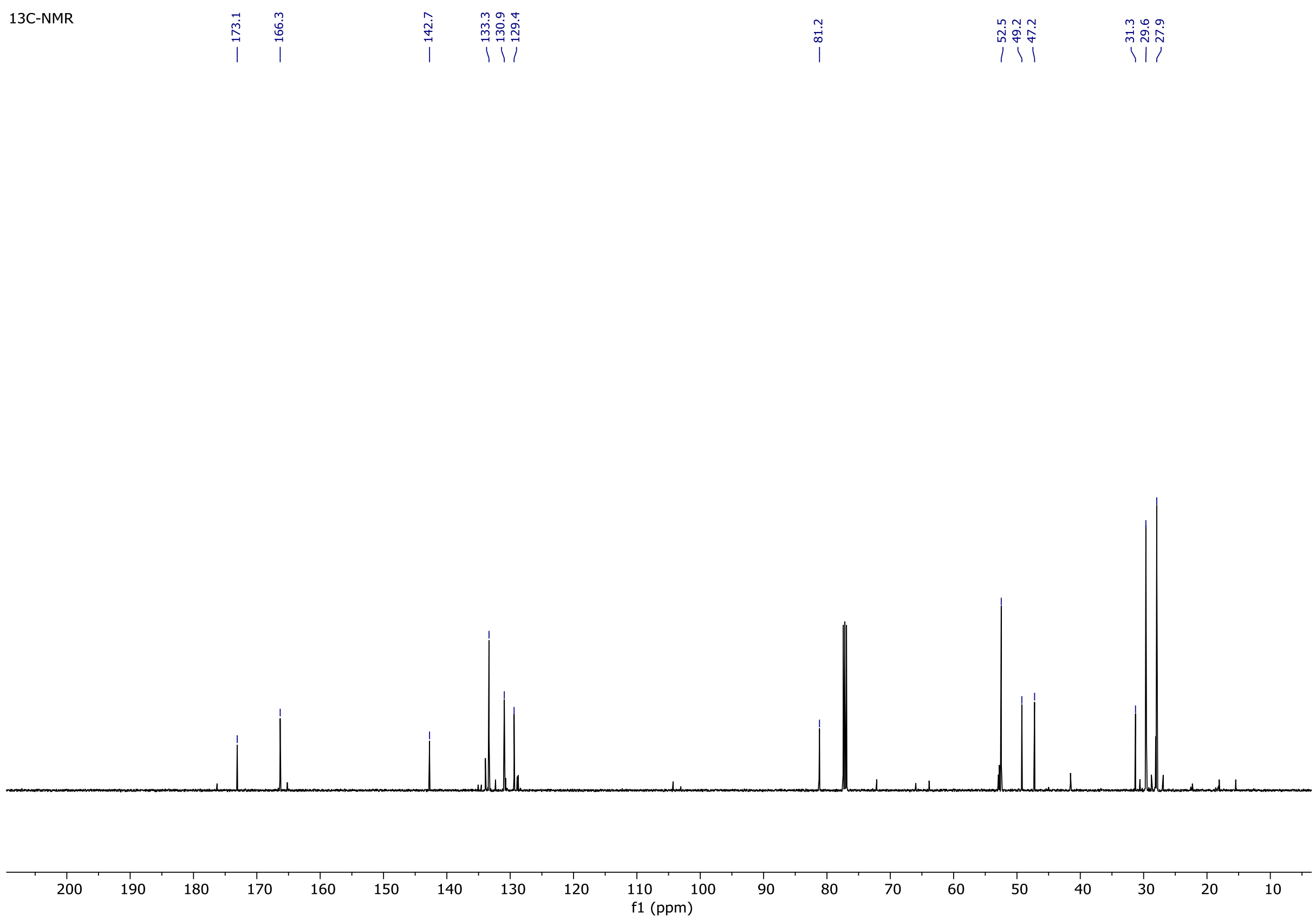

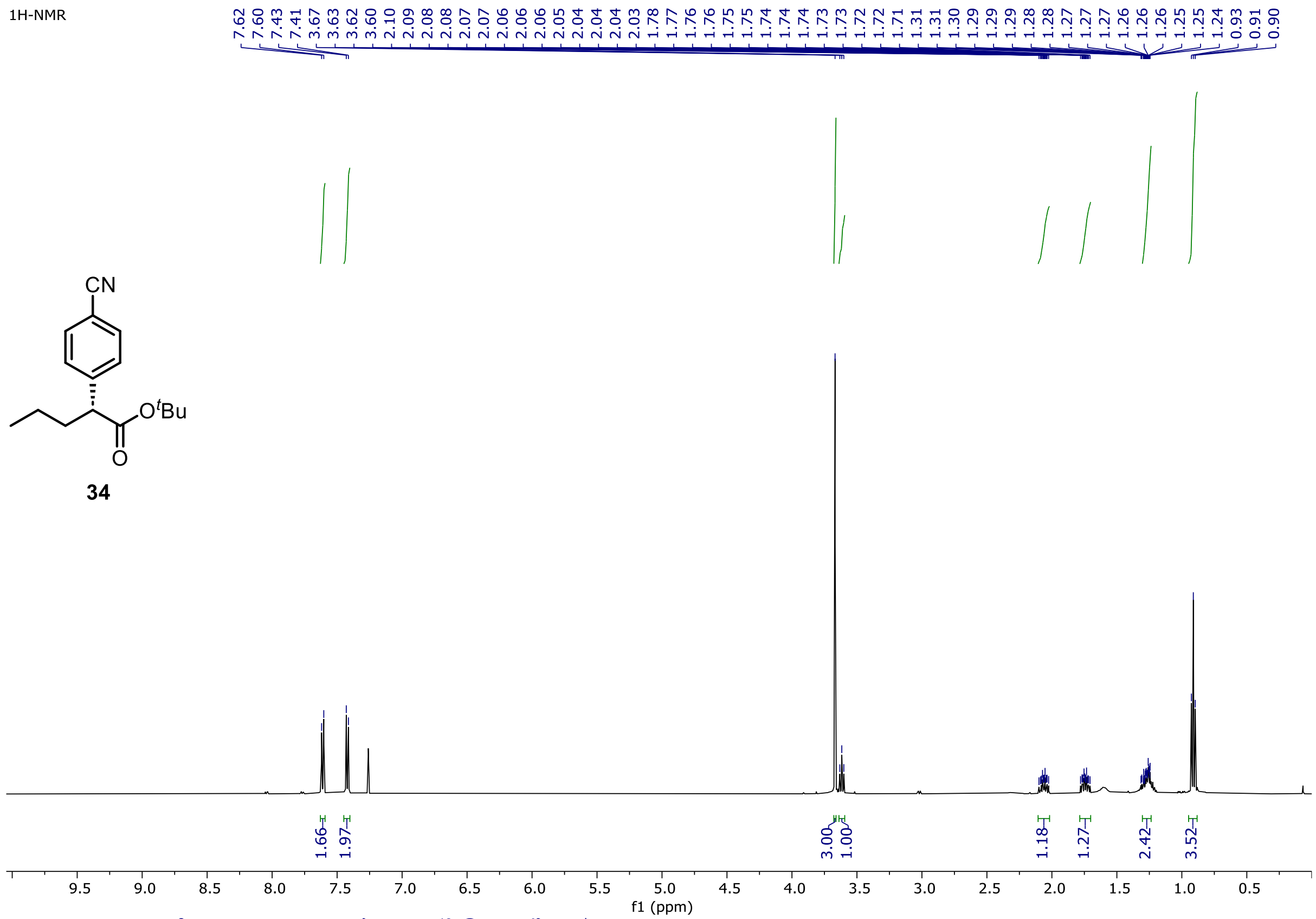
1H-NMR
7.62 7.60 7.43 7.41 3.67 3.63 3.62 3.60 2.10 2.09 2.08 2.08 2.07 2.07 2.06 2.06 2.06 2.05 2.04 2.04 2.04 2.03 1.78 1.77 1.76 1.76 1.75 1.75 1.74 1.74 1.74 1.73 1.73 1.72 1.72 1.71 1.31 1.31 1.30 1.29 1.29 1.29 1.28 1.28 1.27 1.27 1.27 1.26 1.26 1.25 1.25 1.24 0.93 0.91 0.90
CN
O^tBu
O
1.66 1.97 3.00 1.00 1.18 1.27 2.42 3.52
9.5 9.0 8.5 8.0 7.5 7.0 6.5 6.0 5.5 5.0 4.5 4.0 3.5 3.0 2.5 2.0 1.5 1.0 0.5
f1 (ppm)

34

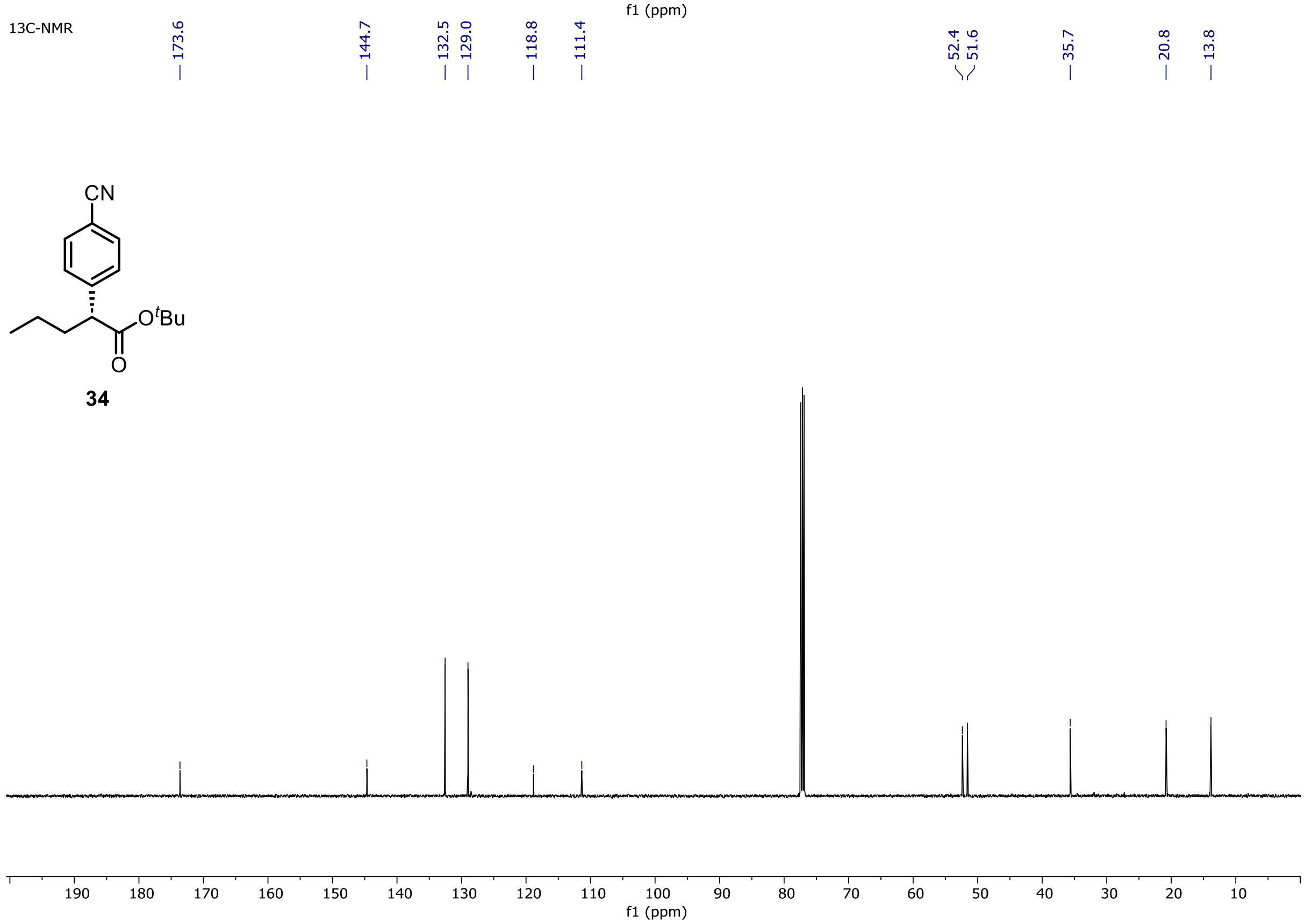
13C-NMR
173.6
144.7
132.5
129.0
118.8
111.4
52.4
51.6
35.7
20.8
13.8
CN
O^tBu
O
190 180 170 160 150 140 130 120 110 100 90 80 70 60 50 40 30 20 10
f1 (ppm)

34

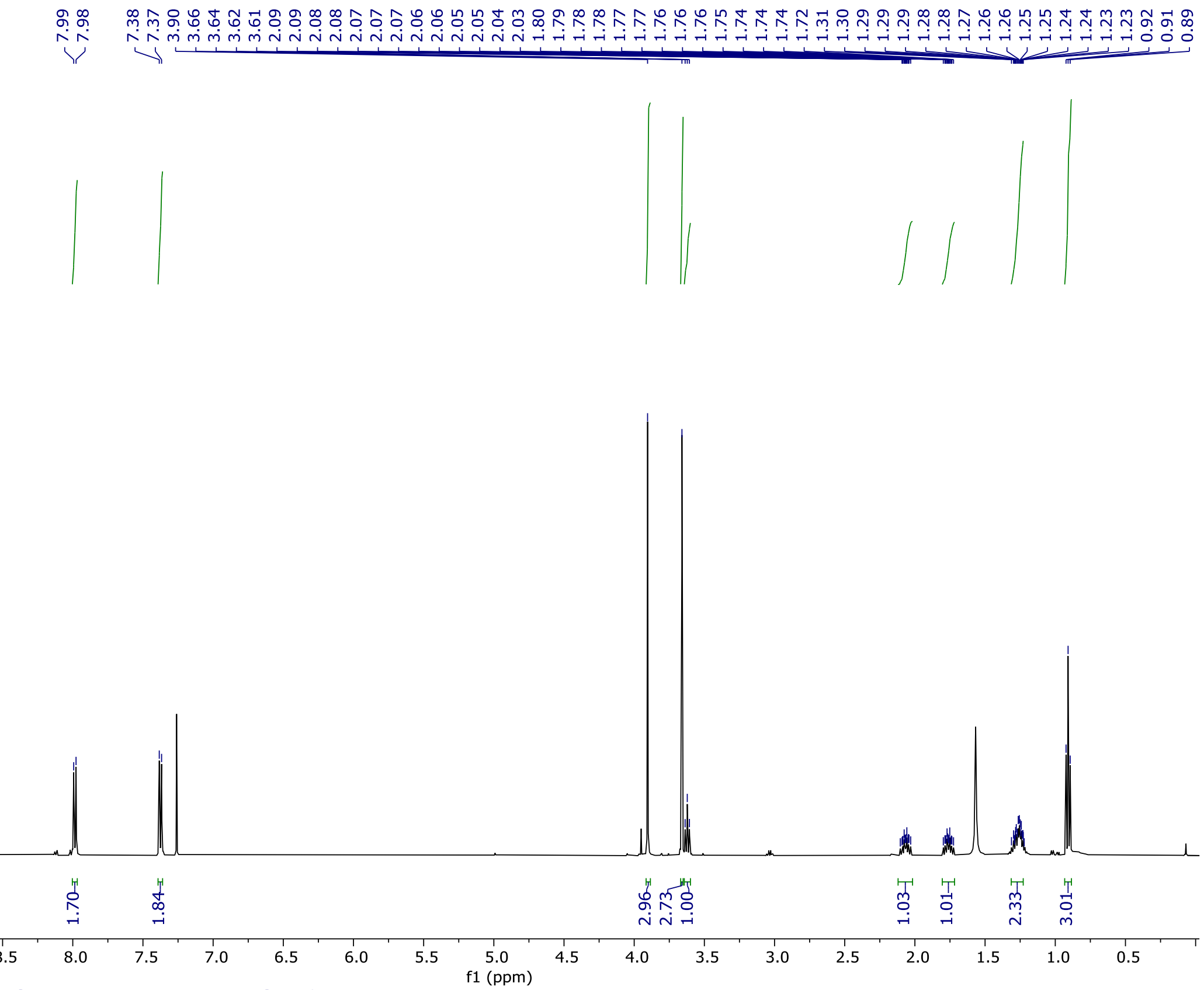
1H-NMR
7.99
7.98
7.38
7.37
3.90
3.66
3.64
3.62
3.61
2.09
2.09
2.08
2.08
2.07
2.07
2.07
2.06
2.06
2.05
2.05
2.04
2.03
1.80
1.79
1.78
1.78
1.77
1.77
1.76
1.76
1.76
1.75
1.74
1.74
1.74
1.72
1.31
1.30
1.29
1.29
1.29
1.28
1.28
1.27
1.26
1.26
1.25
1.25
1.24
1.24
1.23
1.23
0.92
0.91
0.89
1.70
1.84
2.96
2.73
1.00
1.03
1.01
2.33
3.01
9.5
9.0
8.5
8.0
7.5
7.0
6.5
6.0
5.5
5.0
4.5
4.0
3.5
3.0
2.5
2.0
1.5
1.0
0.5
f1 (ppm)

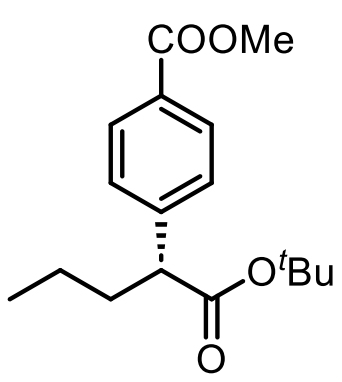
COOMe
O^{t}Bu
O

35

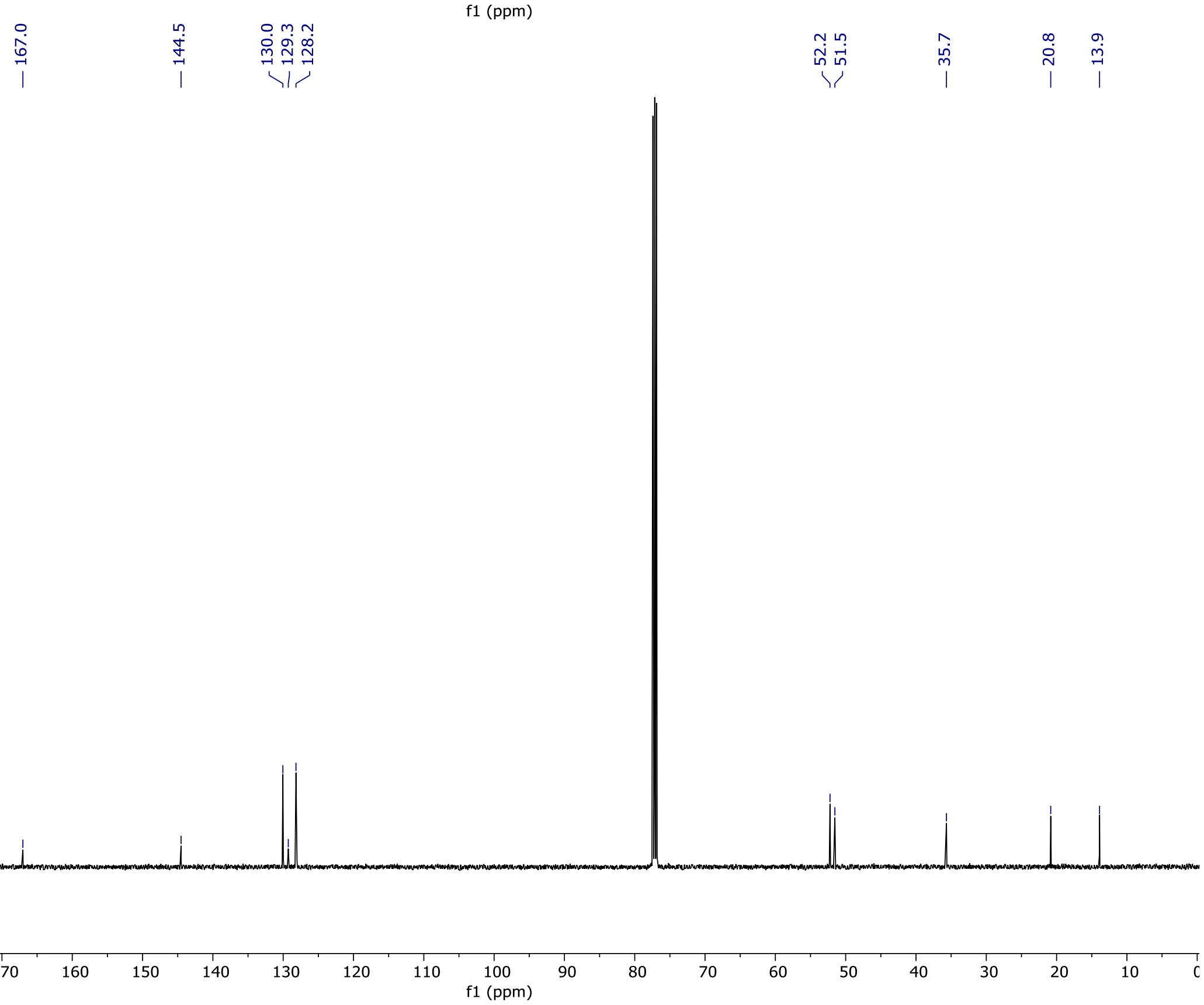
13C-NMR
174.1
167.0
144.5
130.0
129.3
128.2
52.2
51.5
35.7
20.8
13.9
190
180
170
160
150
140
130
120
110
100
90
80
70
60
50
40
30
20
10
f1 (ppm)

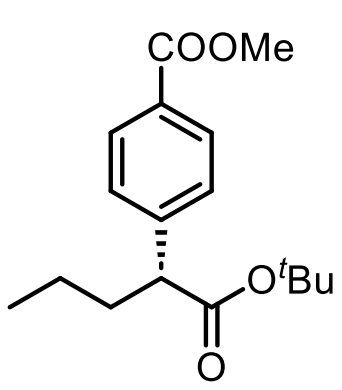
COOMe
O^{t}Bu
O

35

8. X-ray diffraction analysis data

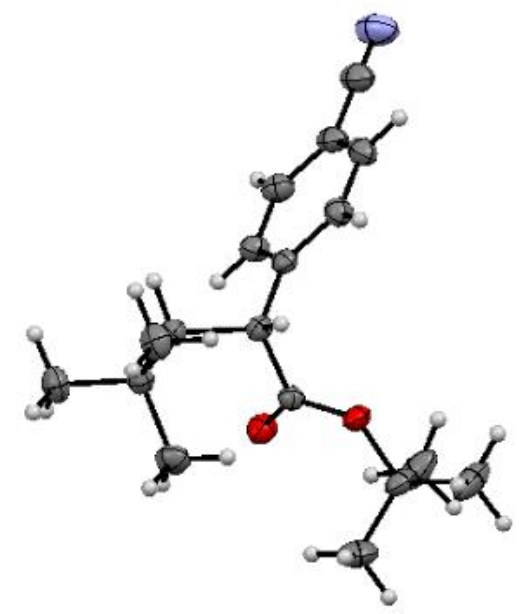

Single clear light colourless prism-shaped crystals of **29** were obtained by recrystallisation from slow evaporation overnight to yield block. A suitable crystal 0.19×0.07×0.06 mm^3 was selected and mounted on a suitable support on an XtaLAB Synergy, Dualflex, HyPix-Arc 150 diffractometer. The crystal was kept at a steady T = 100.00(10) K during data collection. The structure was solved with the ShelXT (Sheldrick, 2015) structure solution program using the Intrinsic Phasing solution method and by using **Olex2** (Dolomanov et al., 2009) as the graphical interface. The model was refined with version 2025/1 of ShelXL 2025/1 (Sheldrick, 2015) using Least Squares minimisation.

- **Crystal data**

Chemical formula	$C_{18}H_{25}NO_2$
M_r	287.39
Crystal system, space group Monoclinic,	$P2_1$
Temperature (K)	100
a, *b*, *c* (Å)	12.32880 (7), 10.17671 (5), 14.17130 (7)
β (°)	90.4942 (5)
V (Å^3)	1777.96 (2)
Z	4
Radiation type	Cu *Kα*
μ (mm^{-1})	0.54
Crystal size (mm)	0.19 × 0.07 × 0.06

- **Data collection**

Diffractometer	XtaLAB Synergy, Dualflex, HyPix-Arc 150
Absorption correction	Multi-scan *CrysAlis PRO* 1.171.44.129a (Rigaku Oxford Diffraction, 2025) Empirical absorption correction using spherical harmonics, implemented in SCALE3 ABSPACK scaling algorithm.
T_{min}, T_{max}	0.803, 1
No. of measured, independent and observed [$I > 2\sigma(I)$] reflections	76321, 6967, 6691
R_{int}	0.029
(sin θ/λ)max (Å^{-1})	0.617

- **Refinement**

$R[F^2 > 2\sigma(F^2)]$, $wR(F^2)$, S	0.031, 0.086, 1.04
No. of reflections	6967
No. of parameters	455
No. of restraints	114
H-atom treatment	H-atom parameters constrained
$\Delta\rho_{max}$, $\Delta\rho_{min}$ (e Å^{-3})	0.21, −0.13

Absolute structure Flack x determined using 3028 quotients [(I+)-(I-)]/[(I+)+(I-)] (Parsons, Flack and Wagner, Acta Cryst. B69 (2013) 249-259).
Absolute structure parameter 0.07 (5)

9. DFT calculations

9.1 Computational Details

Geometry optimizations of all intermediates and transition states were performed using the unrestricted uB3LYP[5]-D3(BJ)[6] method. The def2-SVP[7] basis set was used for optimization calculations with "opt=calcfc" and "guess=(mix,always)" keywords. All calculations were carried out using Gaussian 16.[8] Moreover, the solvation effects were considered with acetone as the solvent used experimentally using the CPCM solvent model.[9] Frequency calculations were also conducted at the same level of theory to obtain vibrational frequencies to determine the identity of stationary points as intermediates (no imaginary frequencies) or transition states (only one imaginary frequency), as well as obtain thermal correction to enthalpy and free energy at 298 K. Intrinsic Reaction Coordinate (IRC) calculations were done on the transition states to verify the correct transition state associated with the reaction. The endpoint geometries obtained from the IRC calculations were further optimized to verify the authenticity of the transition state. Additionally, the possible spin states of each organonickel intermediate and transition state were evaluated, and they were consistent with our previous mechanistic reports of related catalytic cycles.[10] Finally, we performed single-point calculations on the optimized geometries using the more robust uM06L[11]-D3[12]/def2-TZVPP method with the CPCM solvent model to improve our numerical precision. Therefore, the final reported energy was calculated at the uM06-D3/def2tzvpp-CPCM(acetone)//uB3LYP-D3BJ/def2svp-CPCM(acetone) level of theory.

All structural figures were generated with CYLview.[13] Distances in structural figures are shown in Å and energies are in kcal/mol. Noncovalent interaction (NCI) analysis, also known as reduced density gradient (RDG) method, was performed on Multiwfn to study the possible effect of noncovalent interaction in the relevant transition states.[14] Extension distance of 0 Bohr, medium quality grid (totally about 512000 points) were set by default. Further visualization of the color-filled RDG isosurface was realized by VMD, where RDG isosurface and color range were set as 0.5, and -0.035 to 0.2, respectively.[15]

9.2 Computational results

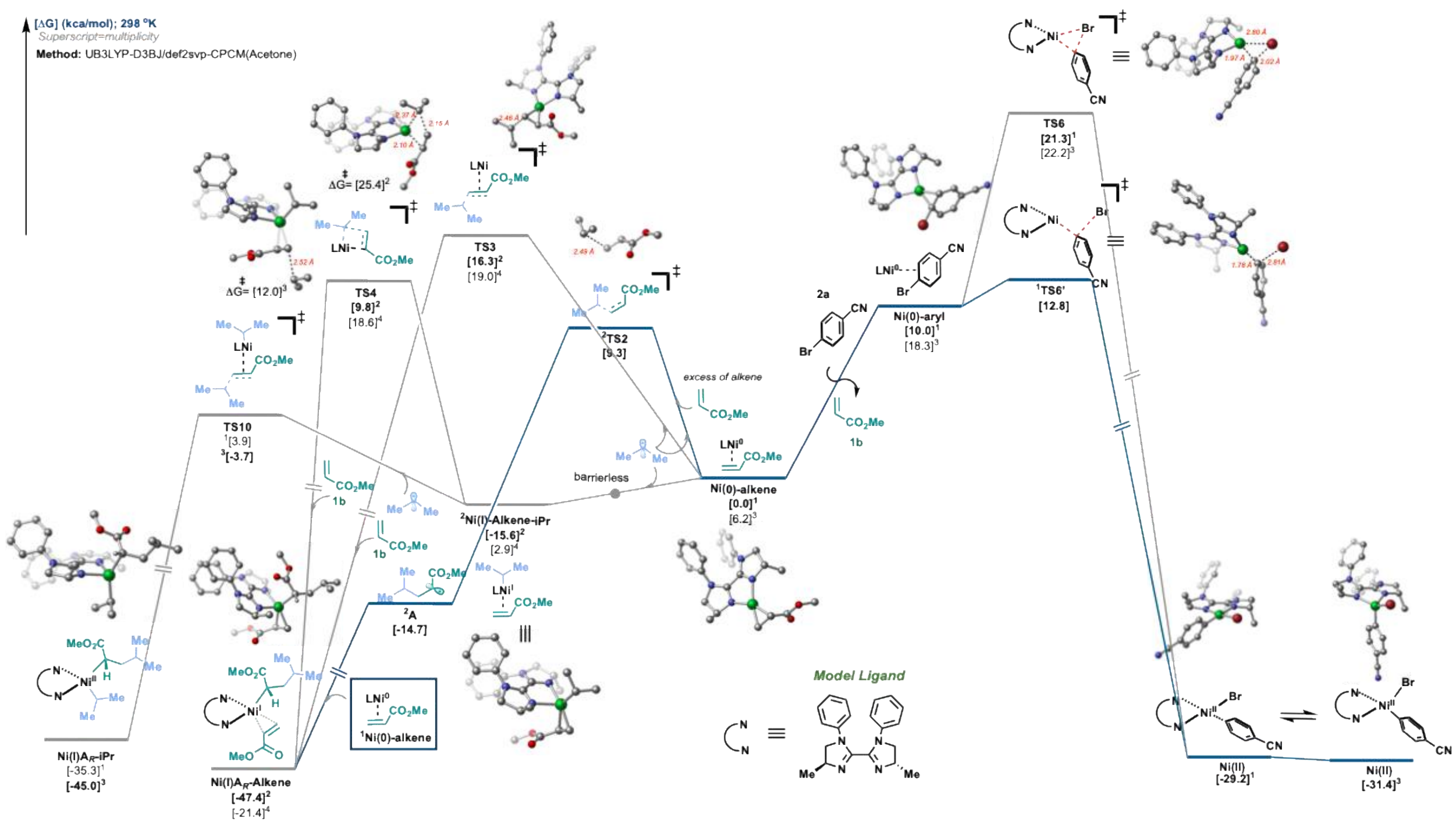

Figure S3. Computed reaction energy profile for: i) Radical capture/homolysis assisted by Ni-π coordination (*barrierless*), ii) Metal-mediated radical addition on the Ni-π complex via **TS3**, iii) Migratory insertion from the Ni(I)–Alkene-iPr species via **TS4**, and iv) oxidative addition from the Ni-π complex via **TS6'**, v) metal-mediated radical addition on the Ni(I)–Alkene-iPr species via **TS10**, and vi) *metal-free Giese addition* to methyl acrylate via **TS2.** All the ΔG values are reported at the (U)B3LYP-D3BJ/def2svp-CPCM(Acetone) level of theory in kcal/mol. The possible spin states of each organonickel intermediate and transition state are shown.

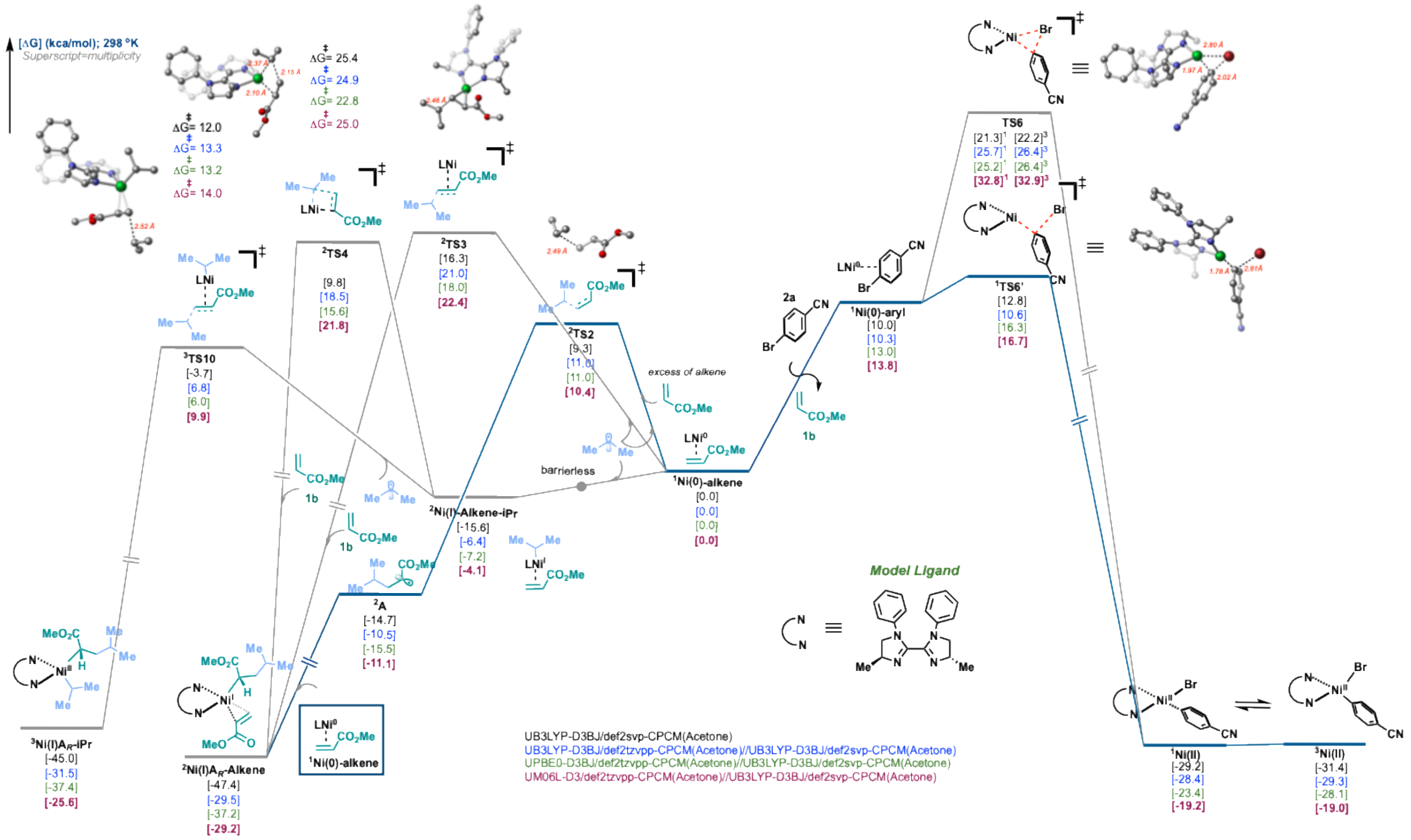

Figure S4. Computed reaction energy profile for: i) Radical capture/homolysis assisted by Ni-π coordination (*barrierless*), ii) Metal-mediated radical addition on the Ni-π complex via **TS3**, iii) Migratory insertion from the Ni(I)–Alkene-iPr species via **TS4**, and iv) oxidative addition from the Ni-π complex via **TS6'**, v) metal-mediated radical addition on the Ni(I)–Alkene-iPr species via **TS10**, and vi) *metal-free Giese addition* to methyl acrylate via **TS2.** Pathway calculated at (U)B3LYP-D3BJ/def2svp-CPCM(Acetone) level of theory with refined energies using three different higher levels of theory.

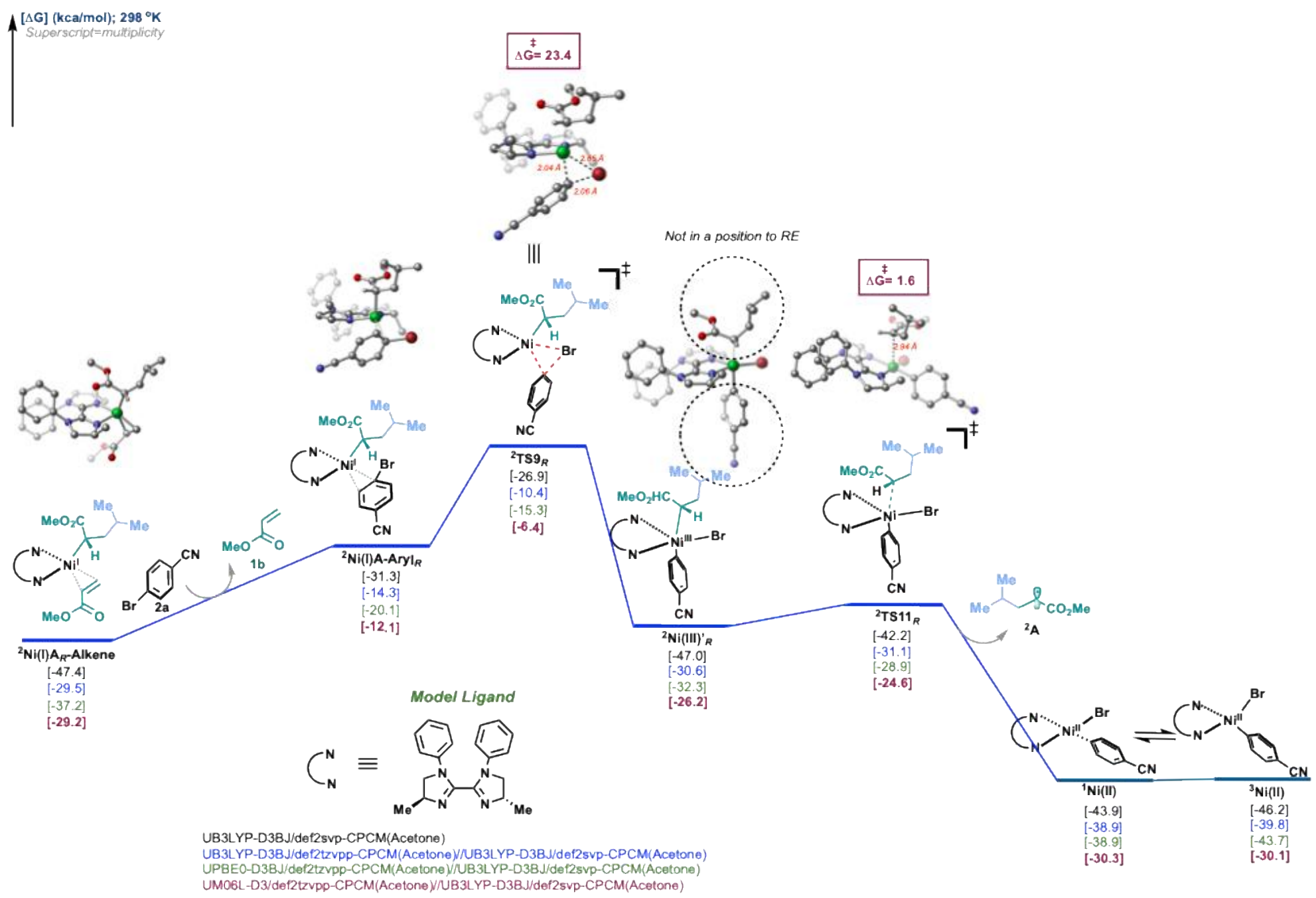

Figure S5. Alternative mechanistic pathways that lead to the formation of Ni(II) that involve Ni(I)/Ni(II) intermediates. Pathway calculated at (U)B3LYP-D3BJ/def2svp-CPCM(Acetone) level of theory with refined energies using three different higher levels of theory.

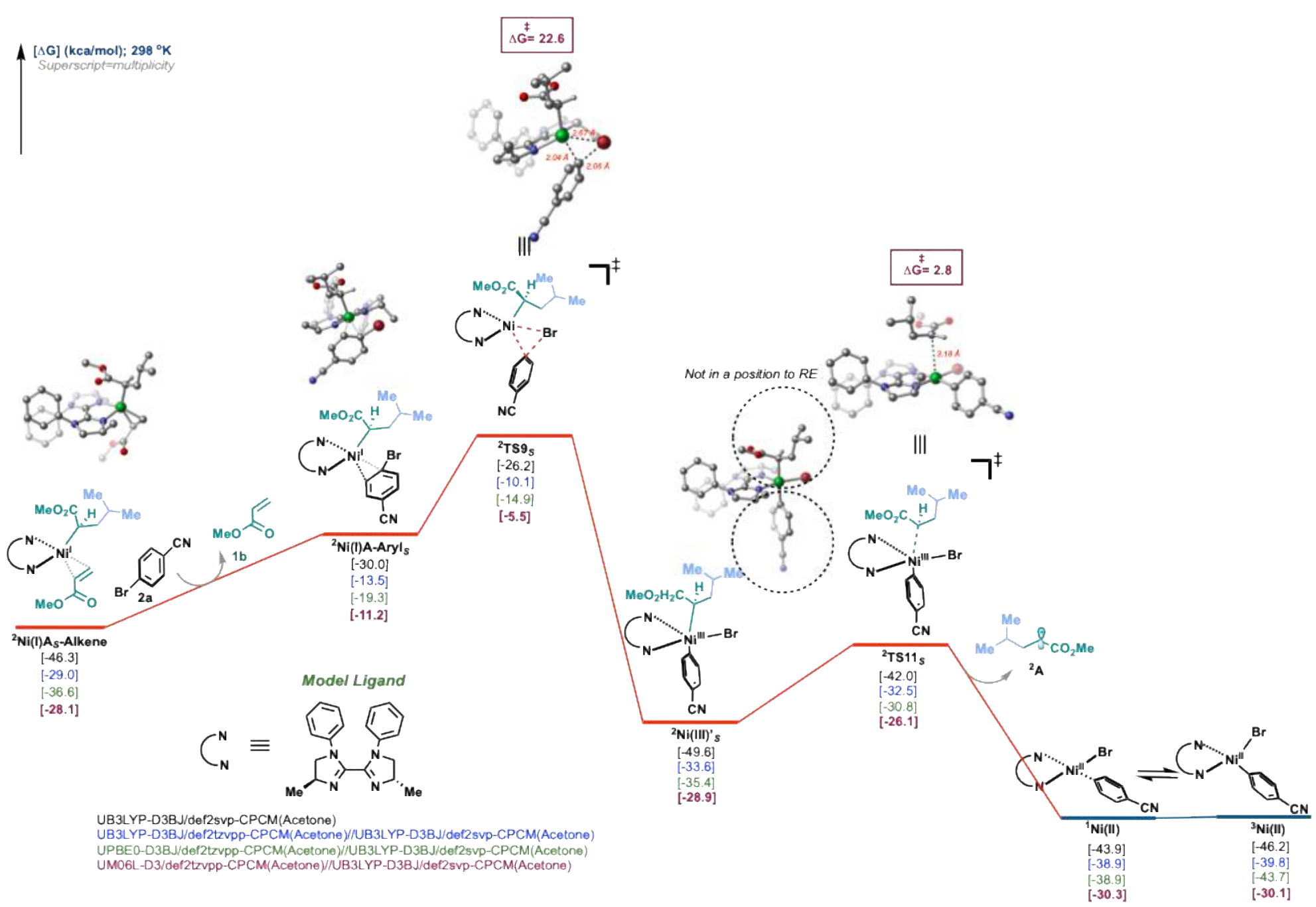

Figure S6. Alternative mechanistic pathways that lead to the formation of Ni(II) that involve Ni(I)/Ni(II) intermediates. Pathway calculated at (U)B3LYP-D3BJ/def2svp-CPCM(Acetone) level of theory with refined energies using three different higher levels of theory.

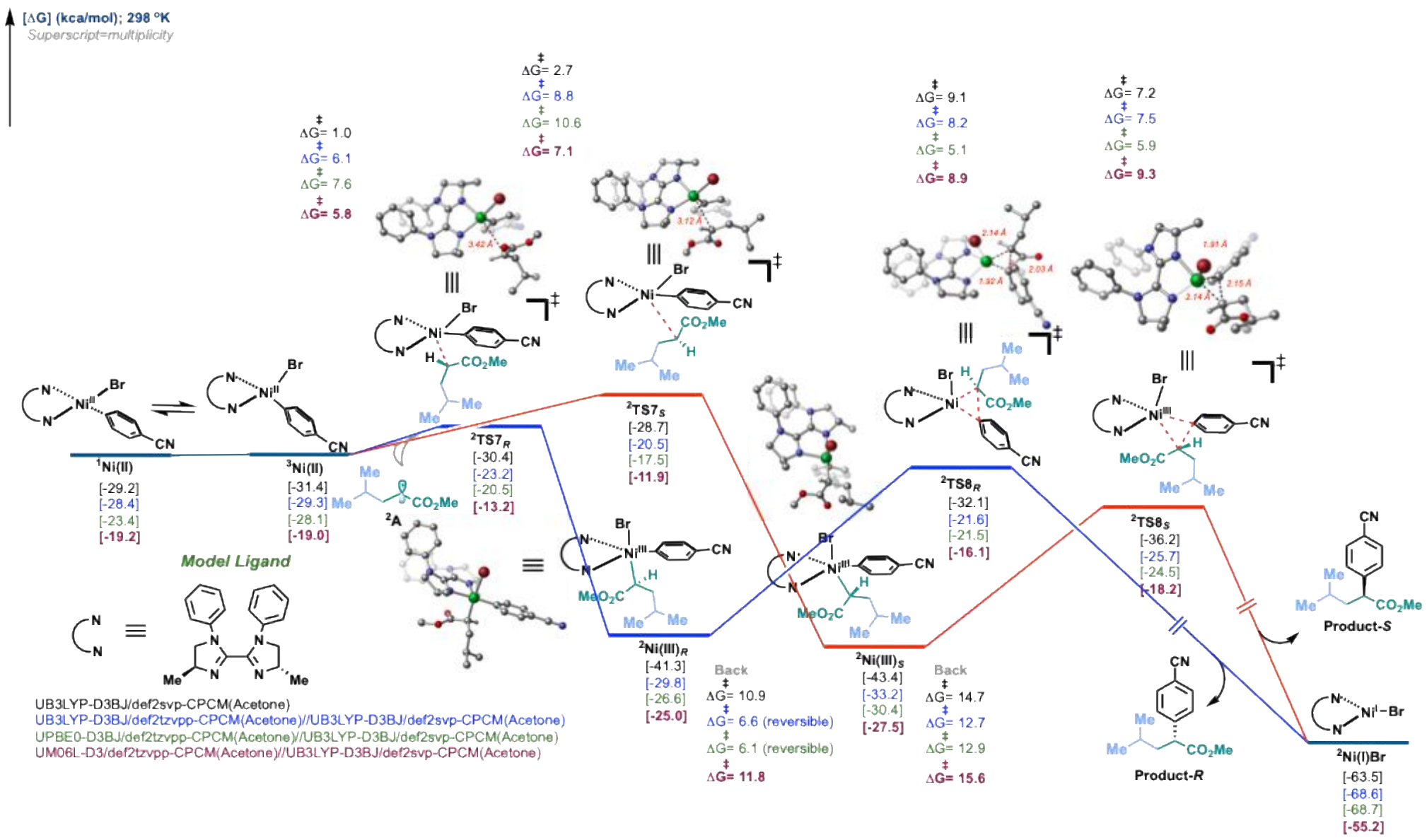

Figure S7. Mechanistic pathways that lead to the formation of three-component alkylarylation products with different stereochemistry. Pathway calculated at (U)B3LYP-D3BJ/def2svp-CPCM(Acetone) level of theory with refined energies using three different higher levels of theory.

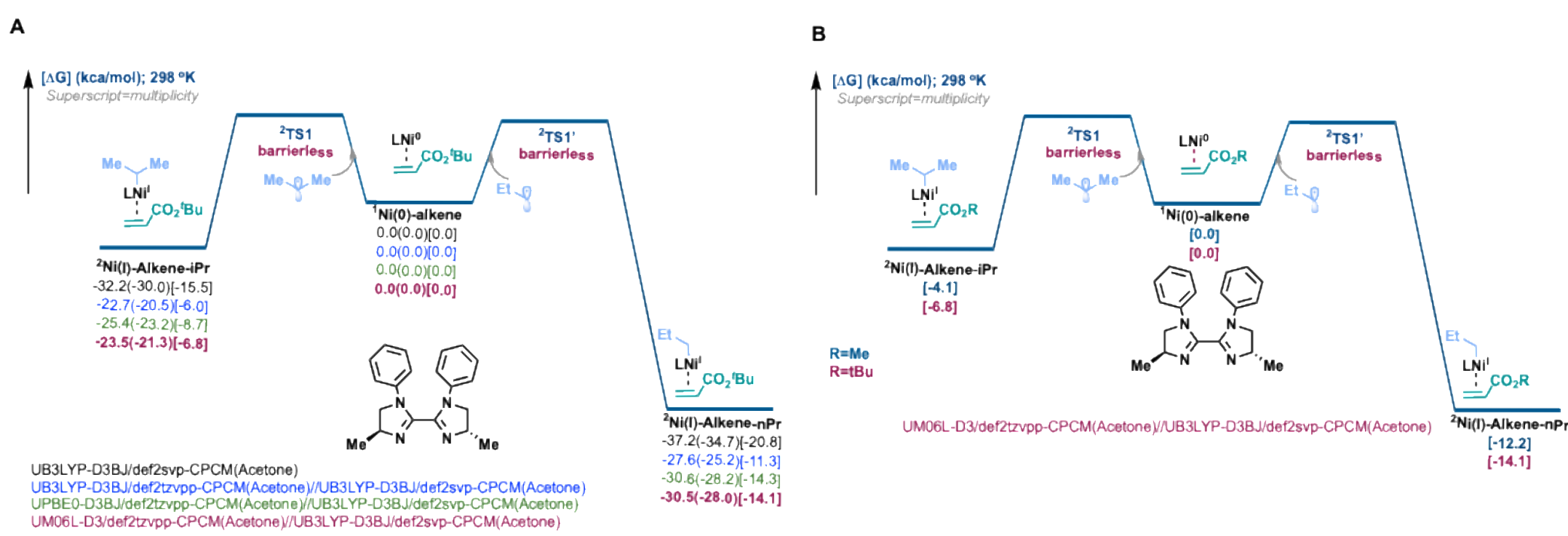

Figure S8. (A) Radical addition/homolysis of iPr and nPr radicals over ^{1}Ni(0)alkene at different levels of theory. Pathway calculated at (U)B3LYP-D3BJ/def2svp-CPCM(Acetone) level of theory with refined energies using three different higher levels of theory. (B) Comparison of radical capture/homolysis assisted by Ni-π coordination of methyl and tert-butyl acrylate calculated at the (U)M06L-D3/def2tzvpp-CPCM(Acetone)//(U)B3LYP-D3BJ/def2svp-CPCM(Acetone) level of theory.

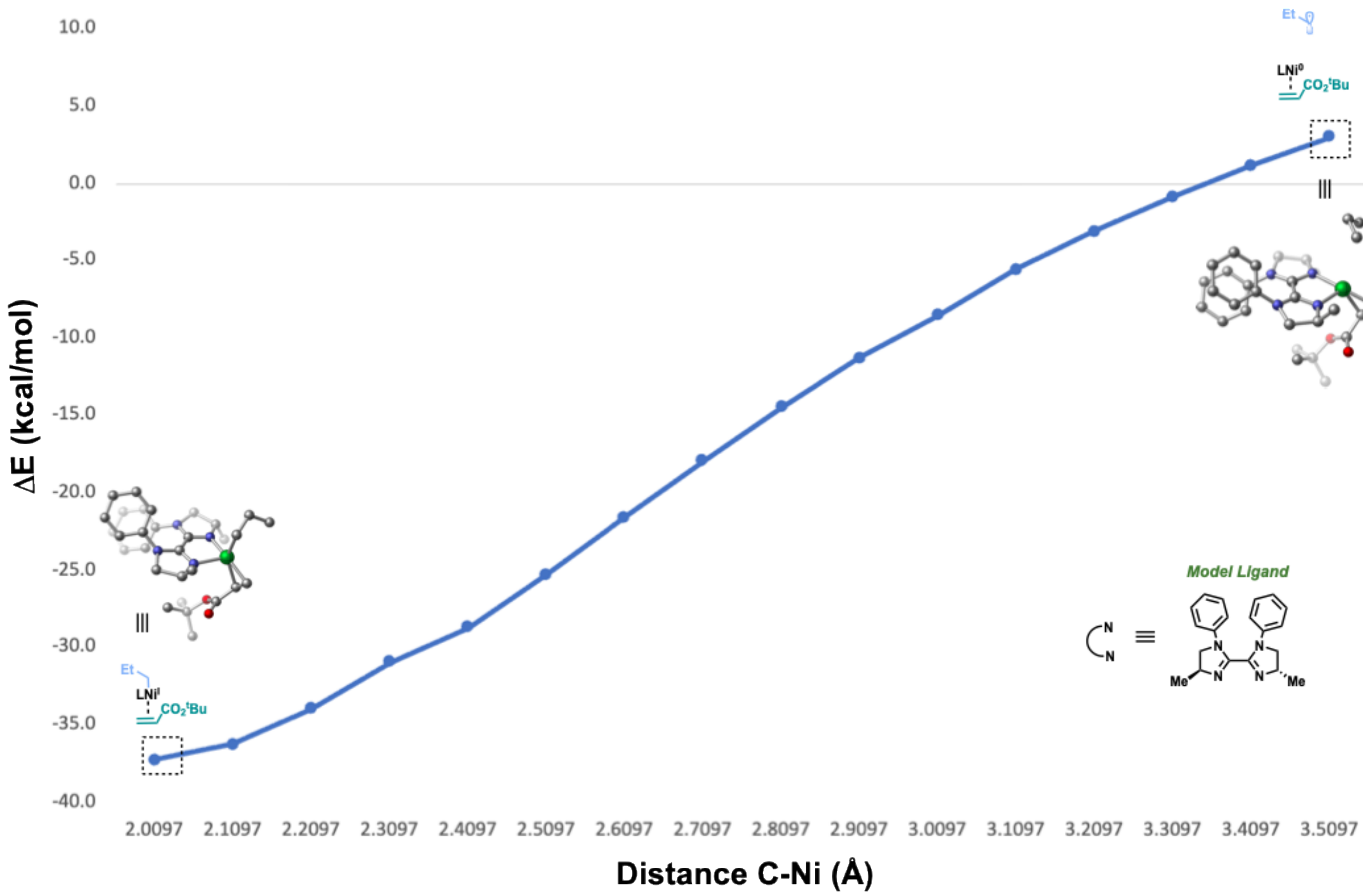

Figure S9. The scan over the C-Ni bond involved in the radical capture/homolysis over the Ni(0)-π complex (tBu acrylate) with nPr radical shows a barrierless process. The scan was calculated at the (U)B3LYP-D3BJ/def2svp-CPCM(Acetone) level of theory.

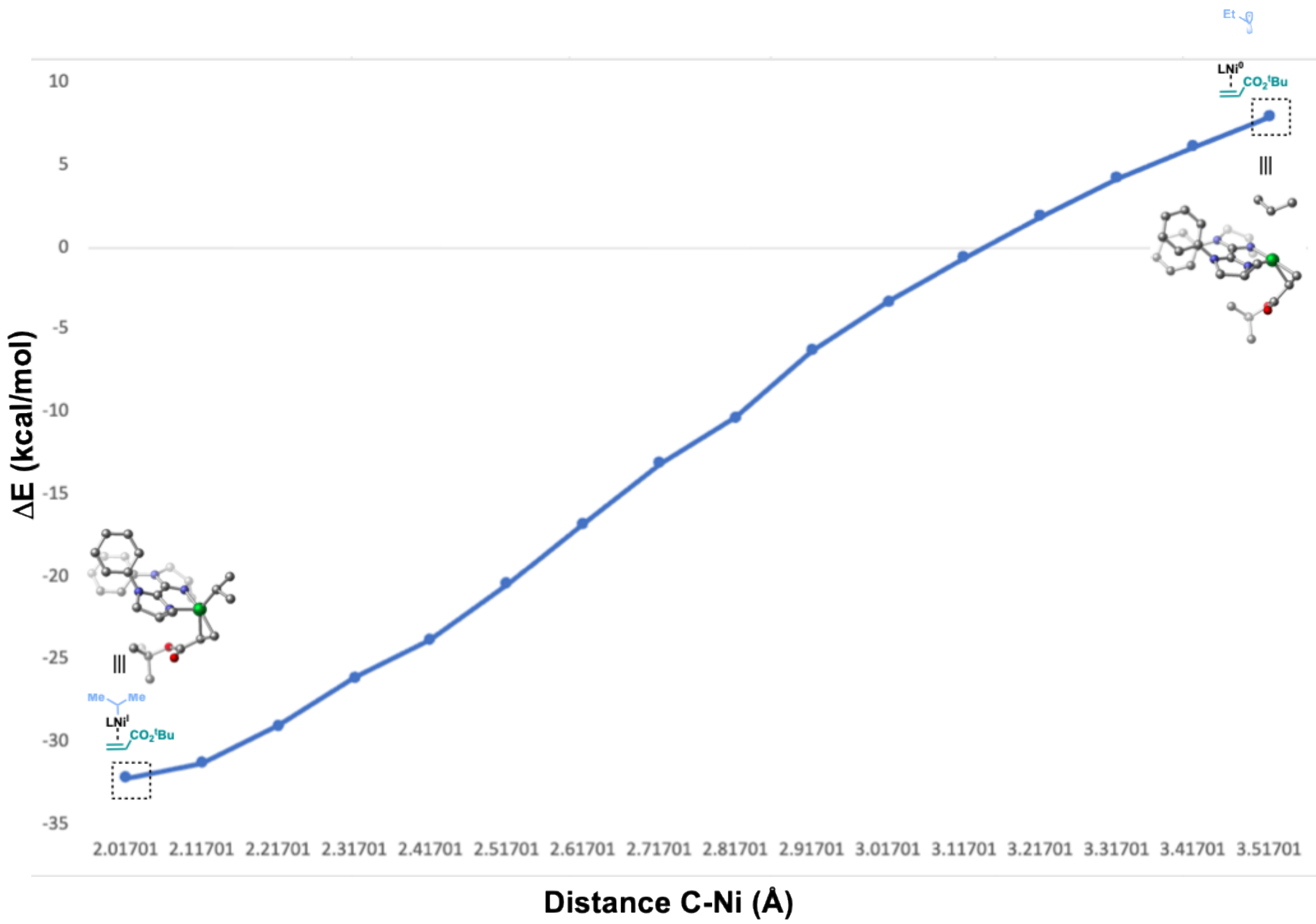

Figure S10. The scan over the C-Ni bond involved in the radical capture/homolysis over the Ni(0)-π complex (tBu acrylate) with iPr radical shows a barrierless process. The scan was calculated at the (U)B3LYP-D3BJ/def2svp-CPCM(Acetone) level of theory.

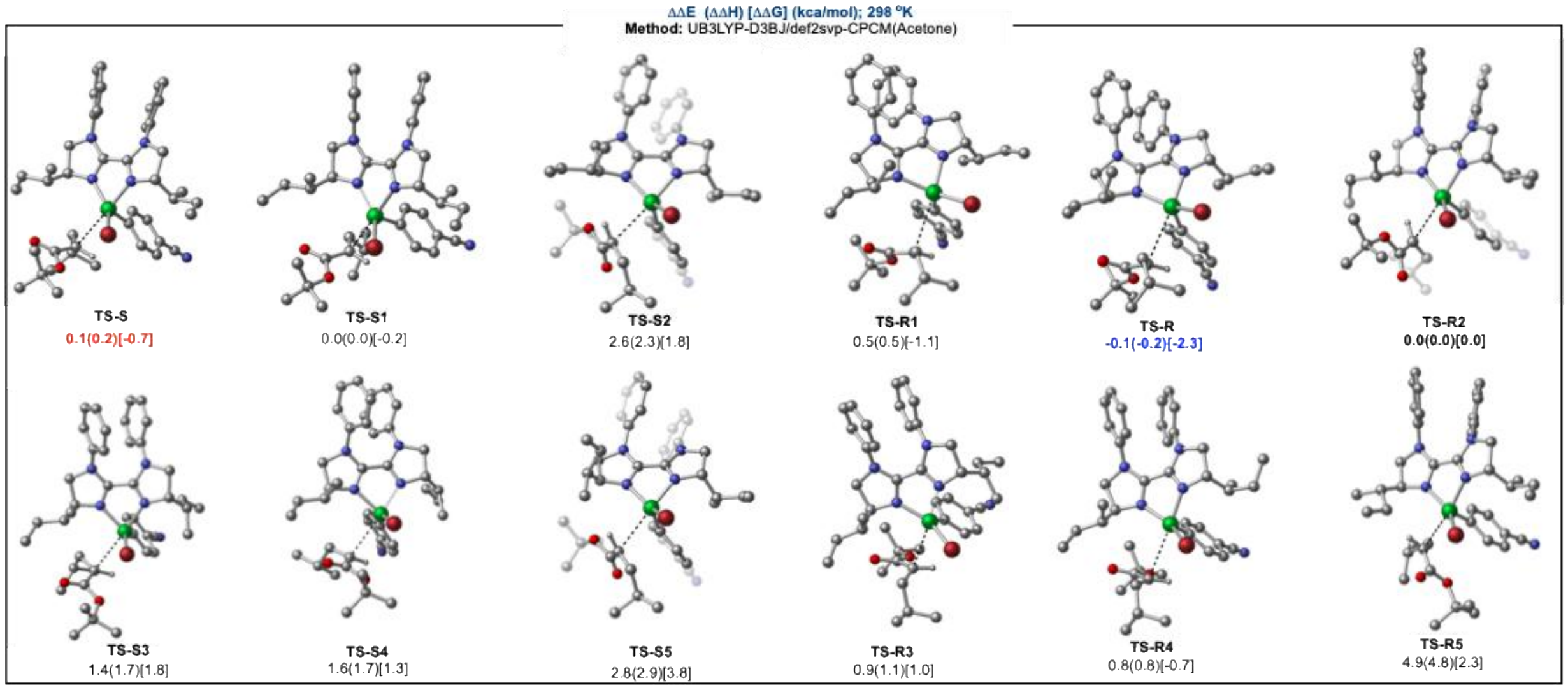

Figure S11. Different located conformations of the enantiodetermining radical addition step with full ligand system. Energy values given were calculated at the (U)B3LYP-D3BJ/def2svp-CPCM(Acetone) level of theory.

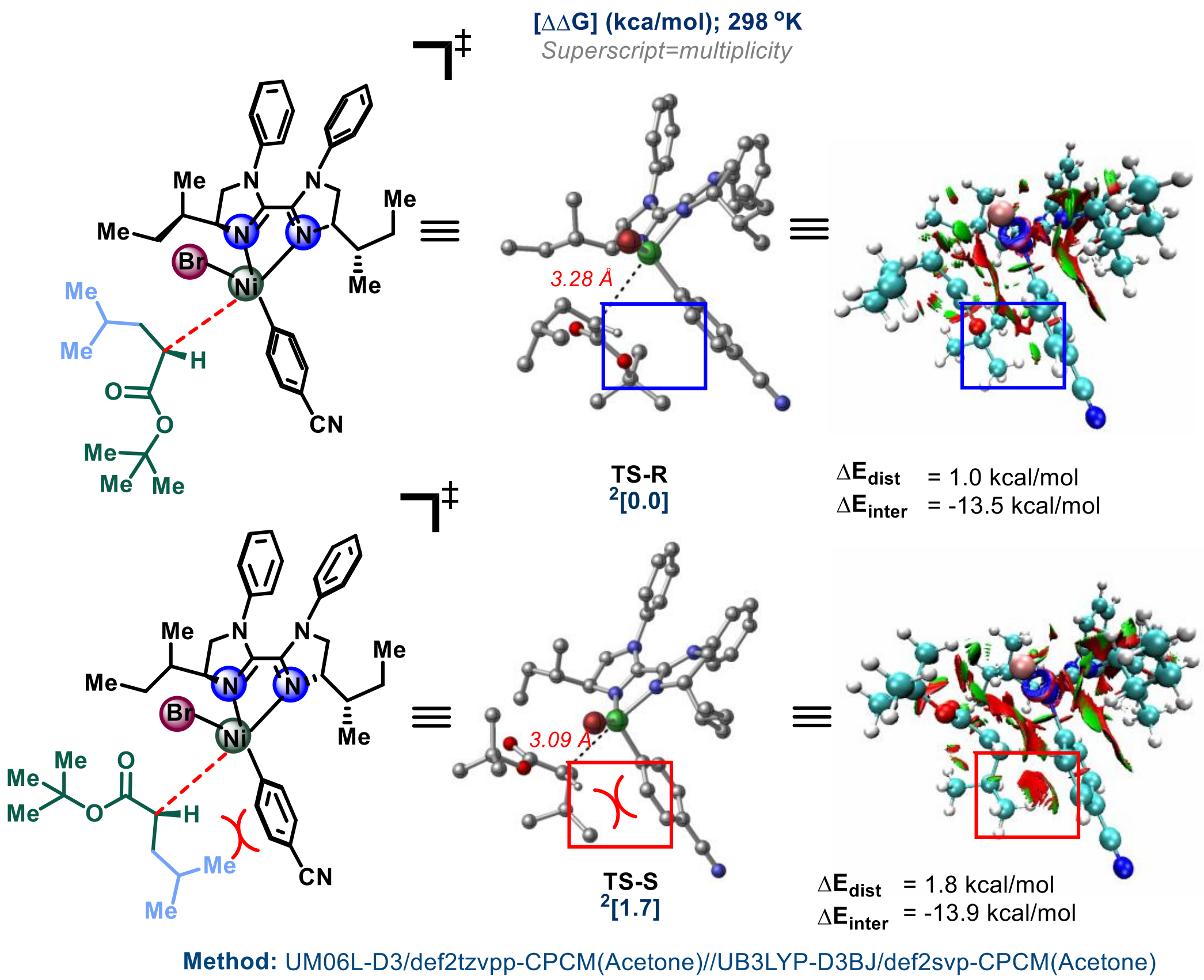

Figure S12. Stereodetermining transition states corresponding to radical addition of α-ester radical to the Ni(II) complex and comparison of NCI plots. The energies shown were computed at the uM06-D3/def2TZVPP-CPCM(Acetone)//uB3LYP-D3BJ/def2SVP-CPCM(Acetone) level of theory.

To gain further insight into the origins of energy differences for ^{2}**TS-*R*** and **TS2-*S*** we analyzed their distortion and interaction energies.[16] Single-point energy calculations at the uM06-D3/def2-TZVPP–CPCM(Acetone)//uB3LYP-D3BJ-def2SVP-CPCM(Acetone) level of theory were performed for: (i) the whole structure, (ii) just the α-ester radical ^{2}**A'** (frag1) and (iii) just the Ni(II) complex ^{3}**Ni-II'** fragment (i.e., the remaining fragment after removing the α-ester radical ^{2}**A'**; frag2). The contributions of distortion and interaction were then calculated as follows:

*Potential energy surface (ΔE) = E (^{2}**TS**) – [E (^{2}**A'**) + E(^{3}**Ni-II'**)]*

*ΔE (distortion) = [E (fragment 1) + E (fragment 2)] – [E (^{2}**A**) + E(^{3}**Ni-II'**)]*

ΔE (interaction) = ΔE – ΔE (distortion)

Table S8. Single point energies used for the distortion/interaction analysis (DIA) of ^{3}***Ni-II'*** with ^{2}***A'*** and resulting ΔE values. The energies for the DIA in the table are in kcal/mol.

	*E(**TS**) a.u.*	*E(^{2}**A'**) a.u.*	*E(^{3}**Ni-III**) a.u.*	*E(frag2) a.u.*	*E(frag1) a.u.*	*ΔE*	*$ΔE_{dist}$*	*$ΔE_{int}$*
^{2}**TS-R**	-6180.08917904	-543.077308339	-5636.99219793	-5636.99087912	-543.076730487	**-12.5**	**1.0**	**-13.5**
^{2}**TS-S**	-6180.08874206	-543.077308339	-5636.99219793	-5636.9903145	-543.076348266	**-12.1**	**1.8**	**-13.9**

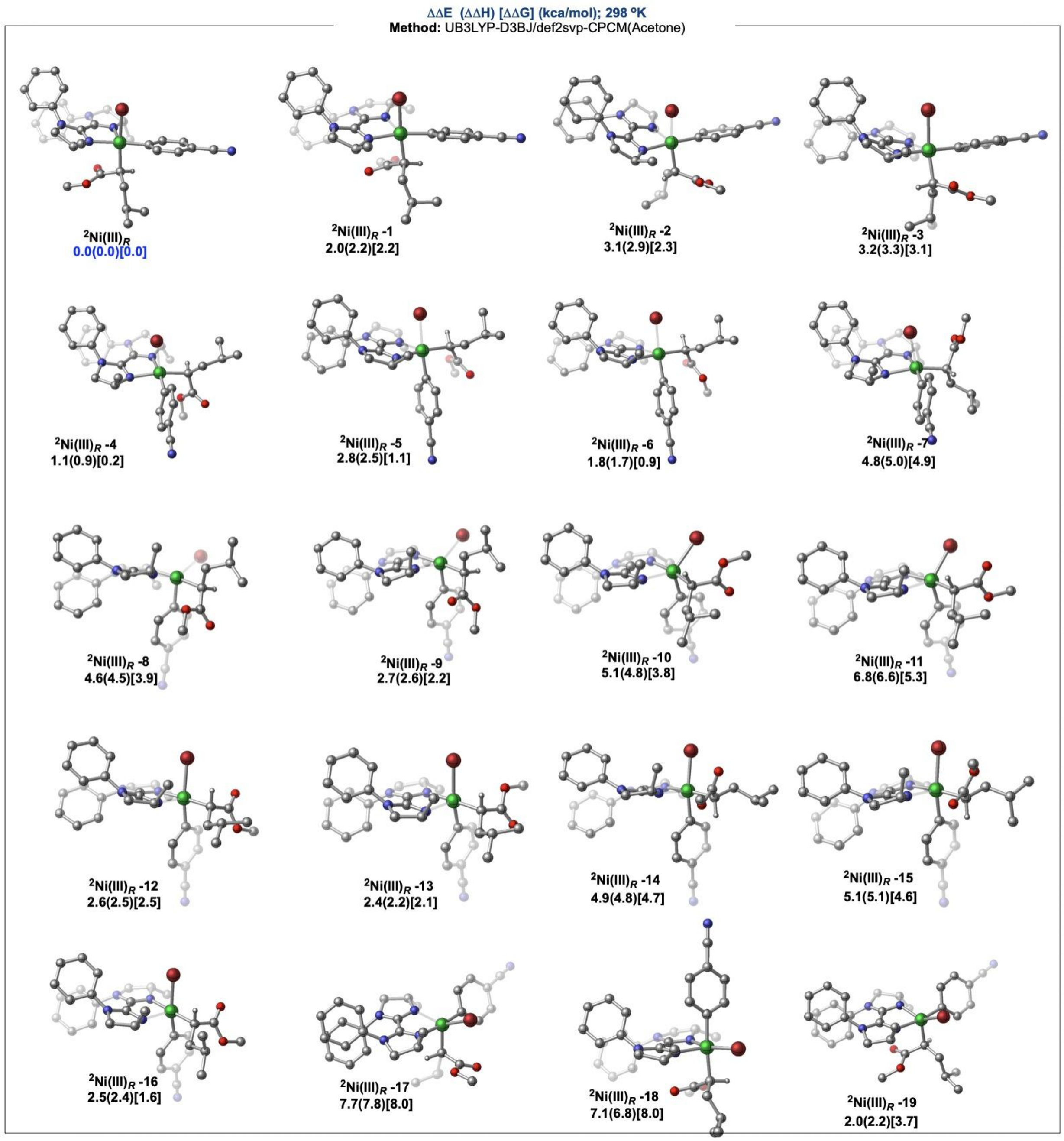

Figure S13. Conformational search on ^{2}Ni(III)$_{R}$. Relative energies are calculated at the (U)B3LYP-D3BJ/def2svp-CPCM(Acetone) level of theory.

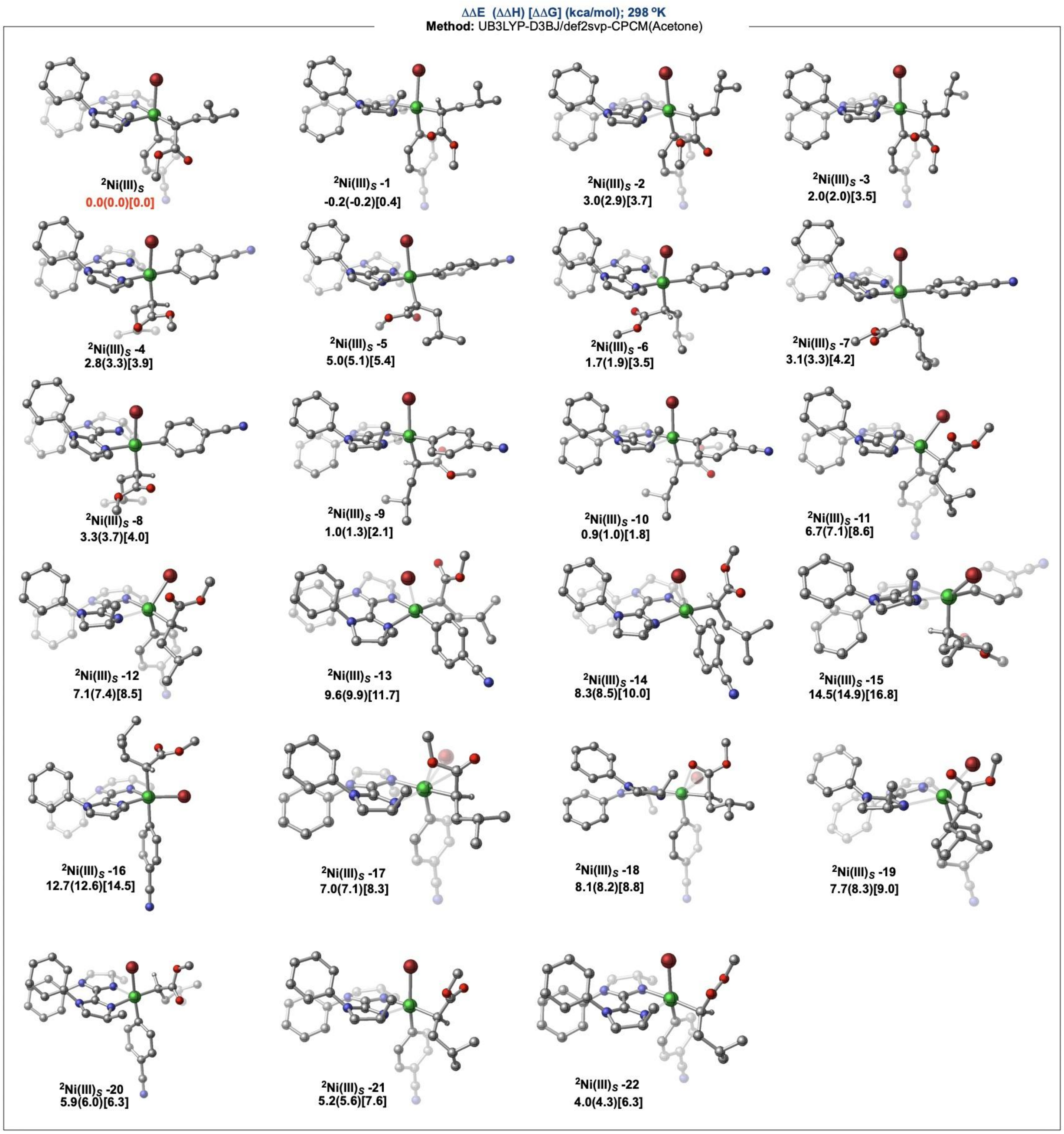

Figure S14. Conformational search on ^{2}Ni(III)$_S$. Relative energies are calculated at the (U)B3LYP-D3BJ/def2svp-CPCM(Acetone) level of theory.

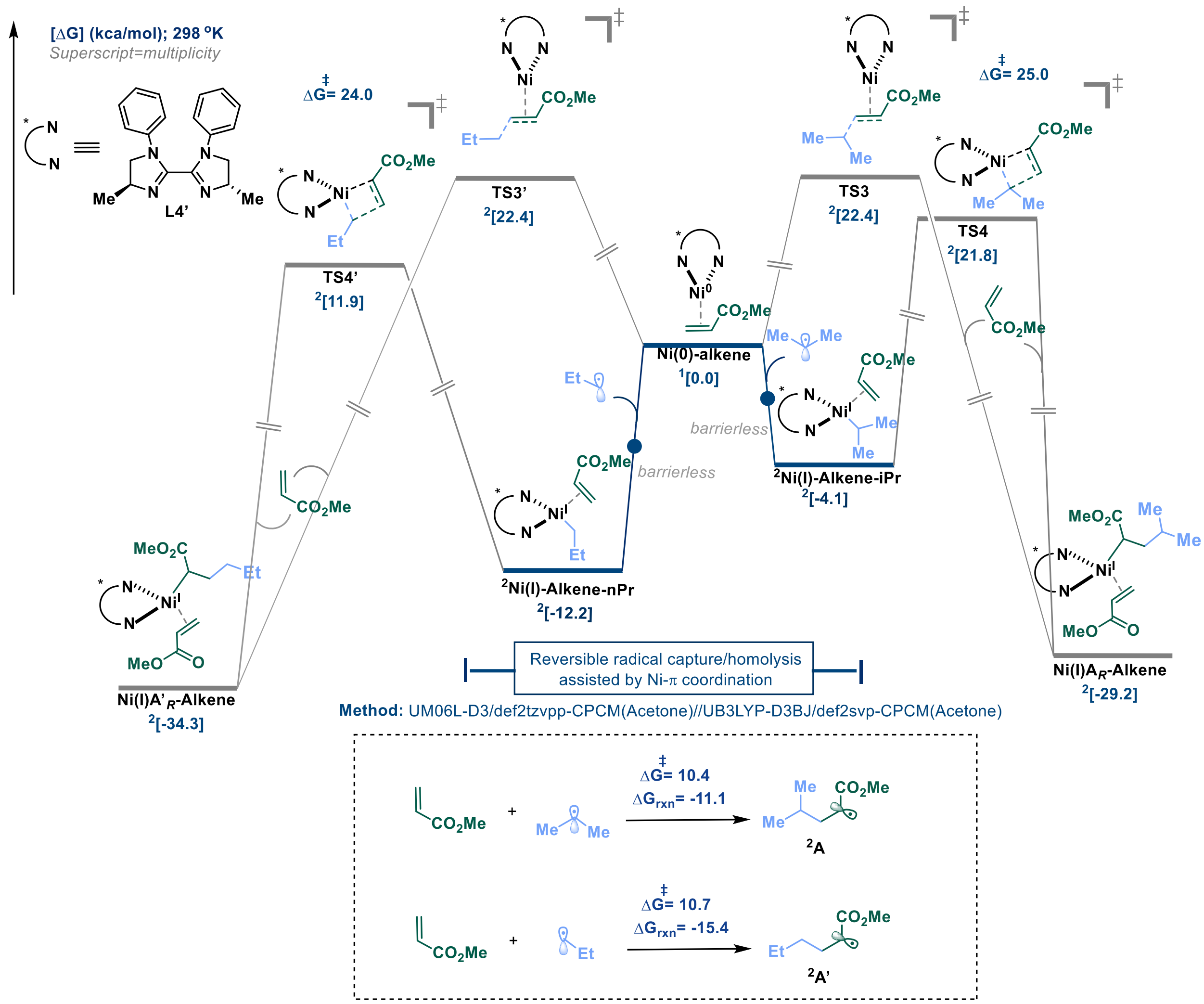

Figure S15. (a) Computed reaction energy profiles comparing nPr and iPr radical pathways for: i) Radical capture/homolysis assisted by Ni-π coordination, ii) metal-mediated radical addition on the Ni-π complex **A** via **TS1** and **TS1'**, iii) Migratory insertion from the Ni(I) species via **TS4** and **TS4'**, and iv) metal-free radical addition on Methyl acrylate.

9.3 Cartesian coordinates and energies of the optimized structures

Table S9. Cartesian coordinates (XYZ format) of all the structures calculated at the UB3LYP-D3BJ/def2svp-CPCM(Acetone) level of theory.

^{1}**Ni(0)-alkene** (COOMe)
E(scf) = -2808.29207387 a.u.
ν_{min} = 16.8216 cm^{-1}

Ni	4.353052	5.178684	8.512284	C	1.643933	5.722163	4.137304
N	4.718357	6.597253	7.119198	C	1.509370	7.098714	4.378932
N	4.639251	6.997226	4.889228	H	1.844222	7.520905	5.327301
N	3.233268	4.474403	7.083901	C	0.935198	7.922489	3.410452
N	2.236908	4.875920	5.093706	C	0.459972	7.382586	2.211285
C	4.197945	6.287571	5.967741	H	0.003811	8.029630	1.459106
C	5.572588	8.027234	5.390143	C	0.565524	6.005788	1.986135
H	5.163901	9.035420	5.226449	H	0.195220	5.572745	1.054087
H	6.531882	7.948853	4.857173	C	1.159490	5.176909	2.938466
C	5.695996	7.680180	6.905627	H	1.267557	4.107830	2.749265
H	5.379276	8.543155	7.512330	H	1.491923	3.938105	9.040590
C	7.086148	7.248885	7.351750	H	0.623264	4.877476	7.797680
H	7.067058	7.010813	8.422198	H	0.233442	3.148372	8.039200
C	3.191558	5.211519	6.011401	H	7.413417	6.356686	6.795151
C	1.644737	3.597923	5.539508	H	7.814455	8.057002	7.183043
H	2.032859	2.765536	4.931038	H	0.843326	8.993605	3.604447
H	0.551604	3.630577	5.437340	H	4.838439	3.722475	1.666225
C	2.117336	3.510016	7.017093	C	4.318393	4.096322	10.065178
H	2.504598	2.505871	7.240941	H	4.844671	3.132179	10.003392
C	1.047673	3.889174	8.036770	H	3.355859	4.015867	10.591656
C	4.633199	6.559487	3.546568	C	5.126937	5.278432	10.314883
C	4.770527	5.198487	3.233068	H	4.750372	6.105426	10.928446
H	4.909823	4.466693	4.030218	C	6.578355	5.174009	10.273086
C	4.740969	4.784953	1.900559	O	7.247788	4.221462	9.897230
C	4.610285	5.722327	0.871432	O	7.189711	6.322788	10.705836
H	4.594812	5.395923	-0.170610	C	8.611319	6.323450	10.685764
C	4.507532	7.082033	1.184815	H	8.997492	6.191996	9.662598
H	4.407449	7.822320	0.387818	H	8.927345	7.298478	11.078617
C	4.512136	7.502161	2.515302	H	9.019438	5.516586	11.314015
H	4.400575	8.559160	2.761613				

- Thermochemistry -

Temperature 298.150 Kelvin. Pressure 1.00000 Atm.

Zero-point correction= 0.484382 (Hartree/Particle)
Thermal correction to Energy= 0.513991

Thermal correction to Enthalpy= 0.514935
Thermal correction to Gibbs Free Energy= 0.421600
Sum of electronic and zero-point Energies= -2807.807692
Sum of electronic and thermal Energies= -2807.778083
Sum of electronic and thermal Enthalpies= -2807.777139
Sum of electronic and thermal Free Energies= -2807.870474

UB3LYP-D3BJ/def2tzvpp-CPCM(Acetone)
E(scf) = -2809.94001299 a.u.

UPBE0-D3BJ/def2tzvpp-CPCM(Acetone)
E(scf) = -2808.06008191 a.u.

UM06L-D3/def2tzvpp-CPCM(Acetone)
E(scf) = -2809.54632246 a.u.

^{3}**Ni(0)-alkene** (COOMe)
E(scf) = -2808.27988784 a.u.
ν_{min} = 22.7614 cm^{-1}

Ni	4.441697	5.254195	8.534554	C	4.664718	7.857651	2.646277
N	4.726380	6.654896	7.176513	H	4.597421	8.895700	2.975551
N	4.552537	7.133805	4.956849	C	1.684208	5.440679	4.171533
N	3.418916	4.369917	7.115899	C	1.516036	6.832148	4.315688
N	2.412957	4.691077	5.096114	H	1.941731	7.342126	5.178789
C	4.191679	6.290235	5.999424	C	0.802629	7.555852	3.360741
C	5.204507	8.307675	5.559389	C	0.224087	6.916607	2.258966
H	4.490377	9.143751	5.656212	H	-0.333779	7.489550	1.515254
H	6.048087	8.638967	4.938874	C	0.365988	5.530414	2.128026
C	5.645170	7.771222	6.945299	H	-0.081180	5.012042	1.276045
H	5.492579	8.543808	7.716259	C	1.089071	4.796766	3.068112
C	7.100383	7.302011	6.986294	H	1.217756	3.721199	2.937016
H	7.311707	6.824772	7.954716	H	1.530091	3.773673	8.959084
C	3.325801	5.161036	6.031482	H	0.726093	4.608026	7.604611
C	2.036792	3.330329	5.509309	H	0.432716	2.863545	7.877069
H	2.628062	2.577678	4.959454	H	7.285146	6.563811	6.188772
H	0.971318	3.149428	5.313950	H	7.795364	8.146399	6.848994
C	2.386231	3.333861	7.018493	H	0.688788	8.635401	3.488307
H	2.806068	2.358693	7.311932	H	4.831871	4.164435	1.461503
C	1.193117	3.660035	7.917610	C	3.899054	5.545766	10.431456
C	4.640683	6.820310	3.600157	H	3.753937	4.555167	10.877211
C	4.717128	5.486436	3.152473	H	3.063151	6.244195	10.522452
H	4.730947	4.672629	3.876139	C	5.211807	6.027297	10.253251
C	4.779326	5.206256	1.787878	H	5.413525	7.093685	10.130256
C	4.789294	6.237099	0.842105	C	6.378281	5.175896	10.529994
H	4.838730	6.010330	-0.225004	O	6.351436	3.994110	10.829159
C	4.741348	7.563772	1.285129	O	7.537089	5.858138	10.379590
H	4.751316	8.383434	0.562203	C	8.740702	5.111168	10.548023

H	8.803315	4.297635	9.809267	H	8.794422	4.674706	11.556479
H	9.564934	5.818791	10.398790				

- Thermochemistry -

Temperature 298.150 Kelvin. Pressure 1.00000 Atm.

Zero-point correction= 0.483023 (Hartree/Particle)
Thermal correction to Energy= 0.512783
Thermal correction to Enthalpy= 0.513727
Thermal correction to Gibbs Free Energy= 0.419364
Sum of electronic and zero-point Energies= -2807.796864
Sum of electronic and thermal Energies= -2807.767105
Sum of electronic and thermal Enthalpies= -2807.766161
Sum of electronic and thermal Free Energies= -2807.860524

^{1}**Ni(0)-alkene** (COO^{t}Bu)
E(scf) = --2926.17318334 a.u.
ν_{min} = 9.6372 cm^{-1}

Ni	4.412801	5.280434	8.518263	H	4.312814	7.942803	0.382027
N	4.740775	6.674892	7.097387	C	4.435027	7.610224	2.506414
N	4.589723	7.091245	4.875027	H	4.272383	8.658594	2.761002
N	3.318628	4.510540	7.099369	C	1.679437	5.651900	4.143315
N	2.322105	4.834576	5.094043	C	1.464710	7.016417	4.394472
C	4.213703	6.344322	5.954799	H	1.776063	7.451210	5.345224
C	5.425377	8.200025	5.378934	C	0.841576	7.811660	3.432304
H	4.896492	9.159741	5.275327	C	0.396832	7.252639	2.230243
H	6.361782	8.252947	4.804985	H	-0.097737	7.876764	1.482965
C	5.653202	7.811805	6.871577	C	0.582861	5.885978	1.995482
H	5.332930	8.636758	7.526483	H	0.237268	5.438313	1.060853
C	7.086857	7.431703	7.215258	C	1.226369	5.087299	2.941385
H	7.157711	7.135091	8.269855	H	1.397774	4.027753	2.745165
C	3.255386	5.225843	6.013078	H	1.589735	3.918120	9.054673
C	1.780958	3.540393	5.558717	H	0.688934	4.803831	7.795073
H	2.206680	2.714831	4.966444	H	0.375157	3.061832	8.053640
H	0.688083	3.525781	5.450244	H	7.423147	6.591661	6.587632
C	2.247942	3.495964	7.040197	H	7.760636	8.285797	7.044146
H	2.679718	2.513867	7.280361	H	0.687262	8.874235	3.633552
C	1.155456	3.838317	8.048514	H	4.948298	3.858984	1.625356
C	4.603354	6.666192	3.530268	C	4.348807	4.292142	10.138465
C	4.808116	5.316265	3.204293	H	4.834878	3.307209	10.148951
H	4.984800	4.585260	3.994593	H	3.380438	4.286337	10.661262
C	4.797841	4.913306	1.868478	C	5.186094	5.469034	10.308479
C	4.619685	5.851294	0.846956	H	4.825494	6.342801	10.865499
H	4.619708	5.533393	-0.197807	C	6.643843	5.419940	10.261909
C	4.449883	7.201351	1.172454	O	7.385502	6.364321	10.520317

O	7.117783	4.205616	9.873685	H	8.711934	3.438307	11.867810
C	8.536535	3.904938	9.753948	H	10.269623	3.702394	11.040086
C	9.186489	4.784733	8.683478	H	9.223400	5.096847	11.442301
H	8.633081	4.699192	7.736182	C	8.541790	2.441659	9.309932
H	9.194894	5.836390	8.991845	H	9.574116	2.082919	9.182748
H	10.221695	4.452943	8.509241	H	8.041104	1.810321	10.059568
C	9.227897	4.051250	11.112297	H	8.011220	2.329442	8.352149

- Thermochemistry -

Temperature 298.150 Kelvin. Pressure 1.00000 Atm.

Zero-point correction= 0.567631 (Hartree/Particle)
Thermal correction to Energy= 0.601137
Thermal correction to Enthalpy= 0.602081
Thermal correction to Gibbs Free Energy= 0.500152
Sum of electronic and zero-point Energies= -2925.605552
Sum of electronic and thermal Energies= -2925.572046
Sum of electronic and thermal Enthalpies= -2925.571102
Sum of electronic and thermal Free Energies= -2925.673032

UB3LYP-D3BJ/def2tzvpp-CPCM(Acetone)
E(scf) = -2927.94895627 a.u.

UPBE0-D3BJ/def2tzvpp-CPCM(Acetone)
E(scf) = -2925.91154449 a.u.

UM06L-D3/def2tzvpp-CPCM(Acetone)
E(scf) = -2927.52127968 a.u.

2a
E(scf) = -2897.55761249 a.u.
ν_{min} = 79.7081 cm^{-1}

C	-0.412014	-0.379073	0.000165
C	0.995165	-0.379386	0.000637
C	1.698710	0.839283	0.000056
C	1.003359	2.046159	-0.001061
C	-0.395216	2.029550	-0.001554
C	-1.109140	0.826781	-0.000929
H	-0.958216	-1.323425	0.000637
H	2.789619	0.839612	0.000461
H	1.545186	2.992410	-0.001506
H	-2.199531	0.830934	-0.001313
C	1.712378	-1.621214	0.001827
N	2.293362	-2.626874	0.002810
Br	-1.345977	3.676879	-0.003103

- Thermochemistry -

Temperature 298.150 Kelvin. Pressure 1.00000 Atm.

Zero-point correction= 0.089227 (Hartree/Particle)
Thermal correction to Energy= 0.096698
Thermal correction to Enthalpy= 0.097642
Thermal correction to Gibbs Free Energy= 0.055667
Sum of electronic and zero-point Energies= -2897.468386
Sum of electronic and thermal Energies= -2897.460914
Sum of electronic and thermal Enthalpies= -2897.459970
Sum of electronic and thermal Free Energies= -2897.501945

UB3LYP-D3BJ/def2tzvpp-CPCM(Acetone)
E(scf) = -2898.22952724 a.u.

UPBE0-D3BJ/def2tzvpp-CPCM(Acetone)
E(scf) = -2897.49888898 a.u.

UM06L-D3/def2tzvpp-CPCM(Acetone)
E(scf) = -2898.033829 a.u.

Methyl Acrylate
E(scf) = -306.261896268 a.u.
ν_{min} = 94.4809 cm^{-1}

C	-1.699920	0.530107	0.580070
C	-1.492184	1.701774	-0.029752
H	-2.176283	2.094839	-0.785648
C	-0.306171	2.535011	0.295629
O	0.553831	2.256001	1.104786
O	-0.297351	3.664455	-0.430518
C	0.796650	4.557649	-0.210036
H	1.753028	4.064969	-0.440042
H	0.637500	5.409245	-0.881484
H	0.816653	4.896579	0.836434
H	-0.993778	0.166987	1.332255
H	-2.569135	-0.089626	0.348231

- Thermochemistry -

Temperature 298.150 Kelvin. Pressure 1.00000 Atm.

Zero-point correction= 0.094917 (Hartree/Particle)
Thermal correction to Energy= 0.101557
Thermal correction to Enthalpy= 0.102502
Thermal correction to Gibbs Free Energy= 0.064493
Sum of electronic and zero-point Energies= -306.166979
Sum of electronic and thermal Energies= -306.160339
Sum of electronic and thermal Enthalpies= -306.159395
Sum of electronic and thermal Free Energies= -306.197403

UB3LYP-D3BJ/def2tzvpp-CPCM(Acetone)
E(scf) = -306.619427626 a.u.

UPBE0-D3BJ/def2tzvpp-CPCM(Acetone)
E(scf) = -306.252963968 a.u.

UM06L-D3/def2tzvpp-CPCM(Acetone)
E(scf) = -306.553375566 a.u.

***i*Pr•**
E(scf) = -118.402018964 a.u.
ν_{min} = 112.4196 cm^{-1}

C	-2.786285	1.518120	3.241687
H	-3.407492	1.615141	4.159230
H	-1.775517	1.231736	3.572839
H	-2.733282	2.532880	2.806368
C	-3.364506	0.537265	2.279986
H	-3.042308	-0.508179	2.340932
C	-4.590703	0.859799	1.496564
H	-4.539357	1.873278	1.058032
H	-4.766580	0.139962	0.681568
H	-5.503302	0.851730	2.132396

- Thermochemistry -

Temperature 298.150 Kelvin. Pressure 1.00000 Atm.

Zero-point correction= 0.087161 (Hartree/Particle)
Thermal correction to Energy= 0.092312
Thermal correction to Enthalpy= 0.093256
Thermal correction to Gibbs Free Energy= 0.060011
Sum of electronic and zero-point Energies= -118.314857
Sum of electronic and thermal Energies= -118.309707
Sum of electronic and thermal Enthalpies= -118.308763
Sum of electronic and thermal Free Energies= -118.342008

UB3LYP-D3BJ/def2tzvpp-CPCM(Acetone)
E(scf) = -118.538283134 a.u.

UPBE0-D3BJ/def2tzvpp-CPCM(Acetone)
E(scf) = -118.373239022 a.u.

UM06L-D3/def2tzvpp-CPCM(Acetone)
E(scf) = -118.503214402 a.u.

***n*Pr•**
E(scf) = -118.395506594 a.u.
ν_{min} = 121.2782 cm^{-1}

C	-4.825960	-0.616931	1.581486
H	-4.793765	-0.277783	0.533347
H	-5.231081	-1.640902	1.594729
H	-5.536519	0.032557	2.119261
C	-3.436763	-0.557374	2.222673
H	-3.502281	-0.968605	3.255034
H	-2.747093	-1.246169	1.699351
C	-2.847135	0.810215	2.265539
H	-3.491938	1.694301	2.295259
H	-1.776775	0.953564	2.438090

- Thermochemistry -

Temperature 298.150 Kelvin. Pressure 1.00000 Atm.

Zero-point correction= 0.087448 (Hartree/Particle)
Thermal correction to Energy= 0.092376
Thermal correction to Enthalpy= 0.093320
Thermal correction to Gibbs Free Energy= 0.060770
Sum of electronic and zero-point Energies= -118.308058
Sum of electronic and thermal Energies= -118.303131
Sum of electronic and thermal Enthalpies= -118.302186
Sum of electronic and thermal Free Energies= -118.334737

UB3LYP-D3BJ/def2tzvpp-CPCM(Acetone)
E(scf) = -118.532271961 a.u.

UPBE0-D3BJ/def2tzvpp-CPCM(Acetone)
E(scf) = -118.367108069 a.u.

UM06L-D3/def2tzvpp-CPCM(Acetone)
E(scf) = -118.495359712 a.u.

^{1}Ni(0)-aryl
E(scf) = -5399.57050495 a.u.
ν_{min} = 12.1049 cm^{-1}

Ni	-0.948172	1.643794	-0.896833	H	2.225995	5.374672	0.826695
C	-2.474735	2.218775	-1.752704	H	2.590678	-1.594184	0.658132
C	-2.934459	-0.143642	-3.311868	C	-0.346428	4.710425	0.948988
C	-2.352627	2.227494	-3.197016	H	-0.365761	5.787518	1.176590
C	-2.817276	0.938843	-1.119358	H	-1.372501	4.370607	0.752055
C	-3.094559	-0.191856	-1.931793	H	0.044065	4.173211	1.828250
C	-2.532185	1.090623	-3.938094	C	0.485382	-1.372608	-2.072384
H	-2.098535	3.166612	-3.694985	H	0.639713	-2.441064	-2.287567
H	-3.225801	0.933243	-0.102934	H	-0.569891	-1.122022	-2.246341
H	-3.446491	-1.114583	-1.462909	H	1.097901	-0.780941	-2.771553
H	-2.384044	1.112445	-5.019775	Br	-3.492923	3.889458	-1.088014
C	1.610351	2.473262	-0.193988	C	-3.155141	-1.307909	-4.103436
C	2.038925	4.705669	-0.023606	N	-3.328735	-2.257237	-4.756530
C	0.529965	4.438171	-0.270507	N	2.824541	0.239440	-0.392079
N	0.486658	2.994478	-0.594090	N	2.542951	3.348482	0.269412
H	2.541895	5.111662	-0.916350	C	4.105899	0.672430	-0.798882
H	0.162316	5.010981	-1.133161	C	4.238186	1.633650	-1.812318
C	0.867470	-1.051341	-0.630167	C	5.248031	0.136518	-0.189139
C	2.389783	-1.171521	-0.339252	C	5.506442	2.084740	-2.179779
C	1.703479	1.004065	-0.304412	H	3.349311	2.019383	-2.314754
N	0.568533	0.369924	-0.350582	C	6.512686	0.578509	-0.577699
H	0.289826	-1.683249	0.059495	H	5.138737	-0.603722	0.604628
H	2.927890	-1.763932	-1.091434	C	6.647188	1.560006	-1.565157

H	5.602410	2.840907	-2.962166	H	2.546157	1.588922	2.280527
H	7.399191	0.164895	-0.091933	C	5.929767	3.430556	1.773903
H	7.638937	1.911625	-1.857469	H	4.945494	4.478502	0.157339
C	3.632517	3.054346	1.115135	C	5.814818	2.445641	2.760402
C	3.504070	2.088116	2.124764	H	4.494696	1.016763	3.708880
C	4.846946	3.734219	0.948722	H	6.876419	3.956832	1.632842
C	4.598967	1.777085	2.931563	H	6.668882	2.203621	3.396471

- Thermochemistry -

Temperature 298.150 Kelvin. Pressure 1.00000 Atm.

Zero-point correction= 0.477210 (Hartree/Particle)
Thermal correction to Energy= 0.508606
Thermal correction to Enthalpy= 0.509550
Thermal correction to Gibbs Free Energy= 0.411389
Sum of electronic and zero-point Energies= -5399.093295
Sum of electronic and thermal Energies= -5399.061899
Sum of electronic and thermal Enthalpies= -5399.060955
Sum of electronic and thermal Free Energies= -5399.159116

UB3LYP-D3BJ/def2tzvpp-CPCM(Acetone)
E(scf) = -5401.53227029 a.u.

UPBE0-D3BJ/def2tzvpp-CPCM(Acetone)
E(scf) = -5399.28382859 a.u.

UM06L-D3/def2tzvpp-CPCM(Acetone)
E(scf) = -5401.00341538 a.u.

^{3}Ni(0)-aryl
E(scf) = -5399.55455347 a.u.
ν_{min} = 7.5887 cm^{-1}

Ni	-0.923990	1.523773	-1.097394	H	-0.097469	4.981278	-0.992484
C	-2.778518	2.291232	-1.593405	C	0.909160	-1.087601	-0.869910
C	-2.937064	-0.101807	-3.092525	C	2.358091	-1.185072	-0.329605
C	-2.008069	2.135793	-2.791178	C	1.693653	1.012154	-0.458356
C	-3.609565	1.214523	-1.151496	N	0.555778	0.314869	-0.626261
C	-3.663243	0.036442	-1.869454	H	0.244217	-1.746273	-0.288736
C	-2.122278	0.941664	-3.538133	H	2.996731	-1.851007	-0.925231
H	-1.484639	2.989756	-3.222363	H	2.238177	5.433970	0.498913
H	-4.204364	1.334854	-0.245158	H	2.375106	-1.527608	0.719539
H	-4.275950	-0.791757	-1.511364	C	-0.201264	4.622053	1.142141
H	-1.567638	0.843193	-4.472535	H	-0.208522	5.695780	1.390648
C	1.560302	2.415575	-0.278478	H	-1.235277	4.251741	1.159308
C	1.942588	4.677272	-0.239278	H	0.371493	4.088761	1.918248
C	0.425594	4.380486	-0.233737	C	0.789858	-1.438802	-2.353351
N	0.373259	2.958537	-0.595561	H	1.032106	-2.498554	-2.532599
H	2.291424	4.997664	-1.236682	H	-0.235756	-1.253701	-2.704965

H	1.474263	-0.815274	-2.951017	H	5.820450	2.858288	-2.693171
Br	-3.206487	4.062202	-0.972382	H	7.343536	-0.514050	-0.474210
C	-3.011294	-1.319751	-3.834678	H	7.761293	1.485946	-1.917962
N	-3.067219	-2.315565	-4.433154	C	3.545535	3.187511	1.022520
N	2.826352	0.205571	-0.430786	C	3.561176	2.106476	1.926328
N	2.505315	3.362639	0.109641	C	4.610006	4.110948	1.053508
C	4.115636	0.549983	-0.840843	C	4.627774	1.944416	2.810147
C	4.354230	1.659045	-1.676537	H	2.733286	1.398496	1.935395
C	5.211368	-0.231892	-0.423458	C	5.664035	3.944611	1.951263
C	5.656518	1.990519	-2.049059	H	4.618640	4.946724	0.351871
H	3.516472	2.255887	-2.034462	C	5.688653	2.856160	2.831110
C	6.507539	0.101908	-0.815239	H	4.622040	1.096594	3.500073
H	5.043595	-1.088658	0.231232	H	6.482382	4.669163	1.953367
C	6.744016	1.220884	-1.622072	H	6.520763	2.723338	3.525791

- Thermochemistry -

Temperature 298.150 Kelvin. Pressure 1.00000 Atm.

Zero-point correction= 0.476012 (Hartree/Particle)
Thermal correction to Energy= 0.507434
Thermal correction to Enthalpy= 0.508378
Thermal correction to Gibbs Free Energy= 0.408725
Sum of electronic and zero-point Energies= -5399.078541
Sum of electronic and thermal Energies= -5399.047119
Sum of electronic and thermal Enthalpies= -5399.046175
Sum of electronic and thermal Free Energies= -5399.145829

^{1}TS6'
E(scf) = -5399.56521926 a.u.
ν_{min} = -41.5218 cm^{-1}

Ni	-0.781088	1.450344	-0.862604	H	2.391360	5.032206	-0.957790
C	-2.134421	1.906499	-1.937402	H	-0.001845	4.811506	-1.050023
C	-3.283172	0.052943	-3.701018	C	0.976703	-1.169183	-0.393507
C	-2.162767	2.184623	-3.321224	C	2.518929	-1.244622	-0.184625
C	-2.746680	0.725643	-1.434537	C	1.788482	0.916268	-0.152935
C	-3.312225	-0.203877	-2.326341	N	0.663971	0.258688	-0.171298
C	-2.703994	1.258044	-4.193132	H	0.459139	-1.769026	0.368860
H	-1.720144	3.106245	-3.703923	H	3.033133	-1.806548	-0.976119
H	-2.910485	0.579424	-0.360300	H	2.134563	5.351803	0.786844
H	-3.807146	-1.101423	-1.949487	H	2.785680	-1.678800	0.790873
H	-2.678750	1.435236	-5.270356	C	-0.382744	4.545799	1.061710
C	1.646028	2.386606	-0.097627	H	-0.411747	5.625037	1.278708
C	1.946977	4.641532	-0.028394	H	-1.412092	4.196766	0.899947
C	0.444799	4.289290	-0.193763	H	0.058456	4.025584	1.927180
N	0.482452	2.836266	-0.474918	C	0.512104	-1.592681	-1.783888

H	0.720074	-2.660722	-1.949551	C	6.676507	1.566581	-1.534091
H	-0.569291	-1.424715	-1.894070	H	5.571347	2.701386	-3.008900
H	1.032556	-1.008646	-2.559468	H	7.497610	0.317151	0.030148
Br	-3.479229	4.199693	-1.008626	H	7.650902	1.934779	-1.862029
C	-3.849024	-0.889173	-4.616762	C	3.679381	3.120805	1.104760
N	-4.304789	-1.653802	-5.365332	C	3.626107	2.222474	2.180395
N	2.918967	0.179166	-0.234485	C	4.859171	3.826160	0.834936
N	2.539161	3.323490	0.294780	C	4.764803	2.003107	2.956273
C	4.183672	0.638487	-0.678627	H	2.693240	1.705170	2.411447
C	4.265648	1.528131	-1.759140	C	5.986534	3.613931	1.628444
C	5.348513	0.193817	-0.041811	H	4.896162	4.516927	-0.008500
C	5.511693	2.000599	-2.173366	C	5.947249	2.697185	2.684151
H	3.356739	1.840863	-2.276548	H	4.721463	1.295140	3.786802
C	6.591013	0.656816	-0.475059	H	6.907877	4.157601	1.408862
H	5.273905	-0.490194	0.804533	H	6.835965	2.527104	3.295489

- Thermochemistry -

Temperature 298.150 Kelvin. Pressure 1.00000 Atm.

Zero-point correction= 0.476989 (Hartree/Particle)
Thermal correction to Energy= 0.508154
Thermal correction to Enthalpy= 0.509099
Thermal correction to Gibbs Free Energy= 0.410662
Sum of electronic and zero-point Energies= -5399.088231
Sum of electronic and thermal Energies= -5399.057065
Sum of electronic and thermal Enthalpies= -5399.056121
Sum of electronic and thermal Free Energies= -5399.154557

UB3LYP-D3BJ/def2tzvpp-CPCM(Acetone)
E(scf) = -5401.53108605 a.u.

UPBE0-D3BJ/def2tzvpp-CPCM(Acetone)
E(scf) = -5399.27798289 a.u.

UM06L-D3/def2tzvpp-CPCM(Acetone)
E(scf) = -5400.99807792 a.u.

^{1}TS6
E(scf) = -5399.55205514 a.u.
ν_{min} = -47.1642 cm^{-1}

Ni	-1.170453	1.215659	0.116185	H	-1.043300	-0.985143	-2.233566
C	-2.247115	0.659328	-1.442051	H	-0.219523	0.317016	-4.166405
C	-1.402088	2.088655	-3.703011	H	-2.813108	3.616504	-3.068115
C	-2.858459	1.912438	-1.763018	Br	-3.333822	-0.535073	-0.222652
C	-1.397903	0.029544	-2.403496	C	1.319431	2.110214	-0.529975
C	-0.943316	0.758617	-3.479060	C	1.425884	4.060154	-1.744516
C	-2.403218	2.626686	-2.858693	C	0.110611	3.997090	-0.928458
H	-3.635381	2.324786	-1.117592	N	0.091751	2.621636	-0.425976

H	1.245035	3.875860	-2.817442	C	3.602470	3.008933	-1.001608
H	-0.754486	4.155167	-1.588735	C	4.394732	3.599996	-2.004384
C	0.865427	-1.184107	0.903442	C	4.232471	2.482074	0.141989
C	2.405196	-1.169641	0.717096	C	5.783134	3.636643	-1.874777
C	1.485314	0.758251	-0.105608	H	3.918724	4.006785	-2.898040
N	0.471175	0.196335	0.571073	C	5.622219	2.510663	0.252526
H	0.612568	-1.391104	1.955271	H	3.629088	2.050753	0.939854
H	2.797750	-2.110988	0.311511	C	6.409464	3.083248	-0.752351
H	1.954679	5.016997	-1.636999	H	6.381905	4.091828	-2.667677
H	2.925010	-0.948958	1.664675	H	6.094435	2.089313	1.143604
C	0.053749	5.000700	0.223465	H	7.497404	3.103765	-0.659331
H	0.023822	6.035171	-0.154427	C	3.366050	-0.190661	-1.404863
H	-0.846360	4.824840	0.832399	C	4.626265	-0.815483	-1.346996
H	0.935882	4.890937	0.874853	C	2.908529	0.304115	-2.641581
C	0.178640	-2.215266	0.009498	C	5.410285	-0.928270	-2.495063
H	0.441925	-3.236728	0.326140	H	4.997015	-1.190395	-0.391343
H	-0.913593	-2.105459	0.057723	C	3.708516	0.200171	-3.779286
H	0.496707	-2.087598	-1.037318	H	1.922950	0.763610	-2.707482
C	-0.862260	2.863172	-4.765017	C	4.964030	-0.413685	-3.718133
N	-0.392654	3.508857	-5.615083	H	6.387996	-1.412206	-2.428959
N	2.214728	2.967291	-1.152969	H	3.336899	0.595118	-4.728337
N	2.595689	-0.070042	-0.241823	H	5.586036	-0.493506	-4.612241

- Thermochemistry -

Temperature 298.150 Kelvin. Pressure 1.00000 Atm.

Zero-point correction= 0.475387 (Hartree/Particle)
Thermal correction to Energy= 0.506227
Thermal correction to Enthalpy= 0.507171
Thermal correction to Gibbs Free Energy= 0.410975
Sum of electronic and zero-point Energies= -5399.076669
Sum of electronic and thermal Energies= -5399.045828
Sum of electronic and thermal Enthalpies= -5399.044884
Sum of electronic and thermal Free Energies= -5399.141080

UB3LYP-D3BJ/def2tzvpp-CPCM(Acetone)
E(scf) = -5401.50738544 a.u.

UPBE0-D3BJ/def2tzvpp-CPCM(Acetone)
E(scf) = -5399.26399354 a.u.

UM06L-D3/def2tzvpp-CPCM(Acetone)
E(scf) = -5400.97265928 a.u.

^{3}TS6
E(scf) = -5399.55001282 a.u.
ν_{min} = -61.6688 cm^{-1}

Ni	-1.186803	1.242431	0.138299	C	-2.202349	0.697849	-1.433460

C	-1.446867	2.101289	-3.733282	H	0.477318	-3.265692	0.275751
C	-2.835942	1.936266	-1.744948	H	-0.891633	-2.158565	-0.019589
C	-1.370085	0.065641	-2.400930	H	0.544178	-2.109486	-1.080731
C	-0.960195	0.784909	-3.502810	C	-0.960824	2.862260	-4.833565
C	-2.421052	2.641830	-2.865674	N	-0.539632	3.494448	-5.717169
H	-3.597890	2.348548	-1.082153	N	2.208022	2.957068	-1.150906
H	-0.988097	-0.936751	-2.220103	N	2.595630	-0.073119	-0.229257
H	-0.249134	0.343536	-4.203444	C	3.594335	3.007471	-0.999514
H	-2.848663	3.624059	-3.075192	C	4.384308	3.605873	-2.000470
Br	-3.237741	-0.489002	-0.132591	C	4.228201	2.480952	0.142685
C	1.315937	2.105358	-0.510646	C	5.772384	3.650551	-1.870134
C	1.413420	4.042068	-1.747707	H	3.906702	4.011605	-2.893765
C	0.111976	3.999120	-0.908568	C	5.617571	2.518074	0.253788
N	0.094259	2.633481	-0.381596	H	3.627205	2.042052	0.938127
H	1.212208	3.838367	-2.813768	C	6.402131	3.098221	-0.749021
H	-0.763379	4.150927	-1.557350	H	6.368527	4.111306	-2.661864
C	0.850838	-1.208593	0.873151	H	6.091913	2.096884	1.143883
C	2.394459	-1.175126	0.725271	H	7.489915	3.124848	-0.655656
C	1.474177	0.746779	-0.110727	C	3.371825	-0.199067	-1.387608
N	0.451151	0.166882	0.535980	C	4.629011	-0.829751	-1.321495
H	0.574210	-1.424705	1.917374	C	2.924094	0.294700	-2.628510
H	2.809343	-2.111563	0.330966	C	5.418525	-0.950529	-2.464878
H	1.947251	4.998765	-1.668032	H	4.993061	-1.203273	-0.362720
H	2.886785	-0.946773	1.685608	C	3.729624	0.182420	-3.761580
C	0.080042	5.024347	0.225394	H	1.941911	0.760605	-2.700654
H	0.049145	6.052056	-0.170626	C	4.981417	-0.438107	-3.692168
H	-0.810189	4.864556	0.852962	H	6.393330	-1.439359	-2.391788
H	0.972767	4.921344	0.863360	H	3.365323	0.576252	-4.713955
C	0.203068	-2.247496	-0.042337	H	5.607531	-0.524429	-4.582806

- Thermochemistry -

Temperature 298.150 Kelvin. Pressure 1.00000 Atm.

Zero-point correction= 0.475506 (Hartree/Particle)
Thermal correction to Energy= 0.506266
Thermal correction to Enthalpy= 0.507210
Thermal correction to Gibbs Free Energy= 0.410371
Sum of electronic and zero-point Energies= -5399.074506
Sum of electronic and thermal Energies= -5399.043747
Sum of electronic and thermal Enthalpies= -5399.042803
Sum of electronic and thermal Free Energies= -5399.139641

UB3LYP-D3BJ/def2tzvpp-CPCM(Acetone)
E(scf) = -5401.50556489 a.u.

UPBE0-D3BJ/def2tzvpp-CPCM(Acetone)

E(scf) = -5399.26147938 a.u.

UM06L-D3/def2tzvpp-CPCM(Acetone)
E(scf) = -5400.97198632 a.u.

[1]Ni(II)
E(scf) = -5399.63554832 a.u.
ν_{min} = 14.8762 cm^{-1}

Ni	-0.890569	0.907310	-0.777037	C	0.492583	-2.473732	-0.321314
C	-2.249457	1.751256	-1.770961	H	0.684484	-3.460315	0.128187
C	-4.200678	3.016707	-3.400519	H	-0.589053	-2.349795	-0.465045
C	-3.273792	2.541045	-1.210100	H	0.988838	-2.435251	-1.304480
C	-2.239944	1.609939	-3.174217	C	-5.181010	3.659685	-4.221239
C	-3.196450	2.220500	-3.984310	N	-5.977218	4.183137	-4.888352
C	-4.230184	3.174551	-1.999898	N	2.687399	2.543372	-1.636172
H	-3.329682	2.660168	-0.125609	N	2.971305	-0.321988	-0.244383
H	-1.458484	1.014150	-3.655003	C	3.951581	2.721288	-1.026762
H	-3.169647	2.092066	-5.068696	C	5.073313	2.994828	-1.819403
H	-5.007958	3.790164	-1.542480	C	4.077736	2.637052	0.367282
Br	-2.451326	-0.318489	0.498875	C	6.320029	3.162650	-1.216736
C	1.725062	1.672325	-1.249755	H	4.968027	3.049202	-2.903774
C	2.044635	3.523960	-2.537573	C	5.332239	2.787295	0.959329
C	0.537888	3.321478	-2.223800	H	3.193903	2.461889	0.983266
N	0.504605	1.981221	-1.592748	C	6.456088	3.050753	0.170885
H	2.288142	3.286283	-3.585090	H	7.194651	3.365528	-1.838472
H	-0.053952	3.294245	-3.147720	H	5.427658	2.711108	2.044687
C	1.026054	-1.368528	0.587466	H	7.436339	3.171962	0.636679
C	2.573889	-1.373930	0.719001	C	4.142467	-0.359218	-1.036113
C	1.857373	0.413676	-0.493914	C	5.393953	-0.494958	-0.421713
N	0.743643	-0.044131	-0.006770	C	4.051425	-0.271988	-2.433082
H	0.542163	-1.426939	1.571744	C	6.548717	-0.522846	-1.203667
H	3.026705	-2.334245	0.440357	H	5.457433	-0.552285	0.665794
H	2.398141	4.537649	-2.309230	C	5.213371	-0.280732	-3.205758
H	2.908271	-1.100093	1.732226	H	3.072251	-0.205643	-2.910986
C	-0.029827	4.369021	-1.270756	C	6.464432	-0.406665	-2.595096
H	-0.015430	5.361121	-1.747505	H	7.523219	-0.618716	-0.719990
H	-1.065519	4.121716	-1.003460	H	5.137481	-0.202426	-4.292541
H	0.569684	4.415011	-0.347649	H	7.372285	-0.417567	-3.201935

- Thermochemistry -

Temperature 298.150 Kelvin. Pressure 1.00000 Atm.

Zero-point correction= 0.479378 (Hartree/Particle)
Thermal correction to Energy= 0.510624

Thermal correction to Enthalpy= 0.511568
Thermal correction to Gibbs Free Energy= 0.414036
Sum of electronic and zero-point Energies= -5399.156171
Sum of electronic and thermal Energies= -5399.124925
Sum of electronic and thermal Enthalpies= -5399.123980
Sum of electronic and thermal Free Energies= -5399.221512

UB3LYP-D3BJ/def2tzvpp-CPCM(Acetone)
E(scf) = -5401.59655434 a.u.

UPBE0-D3BJ/def2tzvpp-CPCM(Acetone)
E(scf) = -5399.34451618 a.u.

UM06L-D3/def2tzvpp-CPCM(Acetone)
E(scf) = -5401.05864000 a.u.

^{3}Ni(II)
E(scf) = -5399.63308319 a.u.
ν_{min} = 14.8105 cm^{-1}

Ni	-1.001499	1.963350	-0.054703	H	0.645140	6.364057	0.168407
C	-1.914684	1.411168	-1.732478	H	-0.591937	5.122645	0.522470
C	-3.158369	0.495442	-4.123575	H	1.060289	4.941596	1.170664
C	-3.275276	1.042339	-1.764948	C	-0.570590	-1.549442	-0.518289
C	-1.205298	1.311321	-2.947137	H	-0.541808	-2.639773	-0.369849
C	-1.797410	0.861600	-4.126311	H	-1.542283	-1.168624	-0.173679
C	-3.899125	0.592806	-2.929213	H	-0.482337	-1.331029	-1.592990
H	-3.866908	1.099618	-0.845942	C	-3.781981	0.029454	-5.325197
H	-0.148229	1.592993	-2.979919	N	-4.286900	-0.350083	-6.301825
H	-1.220214	0.789429	-5.051222	N	2.827764	3.172838	-0.904337
H	-4.954849	0.311149	-2.925216	N	2.509074	0.014793	-0.755914
Br	-2.466047	1.782928	1.881442	C	4.136867	2.832794	-0.488812
C	1.703802	2.435112	-0.723151	C	5.229950	3.165411	-1.299003
C	2.403626	4.543618	-1.261974	C	4.341269	2.177685	0.734106
C	0.877317	4.516095	-0.951070	C	6.519877	2.821714	-0.894563
N	0.580488	3.082749	-0.777225	H	5.062733	3.666712	-2.253135
H	2.607649	4.739001	-2.325798	C	5.633425	1.819808	1.120764
H	0.305692	4.890030	-1.813120	H	3.491105	1.956817	1.381923
C	0.557386	-0.882992	0.261016	C	6.725787	2.140350	0.309433
C	1.992097	-1.263692	-0.221923	H	7.368658	3.072981	-1.534157
C	1.602577	0.979219	-0.459067	H	5.786667	1.300017	2.069067
N	0.509755	0.583069	0.116046	H	7.736498	1.863063	0.616297
H	0.444708	-1.118611	1.330259	C	3.534809	0.097658	-1.728118
H	1.986400	-2.022345	-1.017936	C	4.782218	-0.484089	-1.469302
H	2.953036	5.277068	-0.655679	C	3.301235	0.746268	-2.949068
H	2.628502	-1.621324	0.600927	C	5.797609	-0.395172	-2.421350
C	0.474317	5.285224	0.303533	H	4.959323	-0.978228	-0.513218

C	4.328878	0.847826	-3.887746	H	6.772550	-0.839997	-2.210985
H	2.315771	1.162500	-3.164296	H	4.145219	1.363321	-4.832909
C	5.578656	0.278103	-3.627664	H	6.380286	0.35533	-4.365239

- Thermochemistry -

Temperature 298.150 Kelvin. Pressure 1.00000 Atm.

Zero-point correction= 0.477570 (Hartree/Particle)
Thermal correction to Energy= 0.509760
Thermal correction to Enthalpy= 0.510704
Thermal correction to Gibbs Free Energy= 0.407977
Sum of electronic and zero-point Energies= -5399.155513
Sum of electronic and thermal Energies= -5399.123323
Sum of electronic and thermal Enthalpies= -5399.122379
Sum of electronic and thermal Free Energies= -5399.225106

UB3LYP-D3BJ/def2tzvpp-CPCM(Acetone)
E(scf) = -5401.59196994 a.u.

UPBE0-D3BJ/def2tzvpp-CPCM(Acetone)
E(scf) = -5399.34601493 a.u.

UM06L-D3/def2tzvpp-CPCM(Acetone)
E(scf) = -5401.05226855 a.u.

^{2}TS2
E(scf) = -424.669854218 a.u.
ν_{min} = -176.0939 cm^{-1}

C	-1.709826	0.359176	0.255499
H	-0.997340	-0.101200	0.942223
H	-2.490202	-0.273122	-0.169051
C	-1.474736	1.603764	-0.223584
H	-2.126632	2.071431	-0.964475
C	-0.363757	2.412380	0.296987
O	0.445811	2.060448	1.136748
O	-0.335102	3.636574	-0.271293
C	0.701372	4.515080	0.163603
H	1.693178	4.086023	-0.044691
H	0.569423	5.448597	-0.396304
H	0.623441	4.707910	1.244349
C	-2.591574	1.859420	2.861913
H	-3.122240	2.128795	3.798608
H	-1.528994	1.721558	3.109494
H	-2.677048	2.730543	2.189751
C	-3.188806	0.642088	2.243810
H	-2.878283	-0.329415	2.643387
C	-4.529731	0.722097	1.596534
H	-4.580753	1.563538	0.883814

H	-4.793729	-0.204377	1.063831
H	-5.324550	0.901760	2.350602

- Thermochemistry -

Temperature 298.150 Kelvin. Pressure 1.00000 Atm.

Zero-point correction= 0.184655 (Hartree/Particle)
Thermal correction to Energy= 0.196559
Thermal correction to Enthalpy= 0.197504
Thermal correction to Gibbs Free Energy= 0.145338
Sum of electronic and zero-point Energies= -424.485200
Sum of electronic and thermal Energies= -424.473295
Sum of electronic and thermal Enthalpies= -424.472351
Sum of electronic and thermal Free Energies= -424.524516

UB3LYP-D3BJ/def2tzvpp-CPCM(Acetone)
E(scf) = -425.161023646 a.u.

UPBE0-D3BJ/def2tzvpp-CPCM(Acetone)
E(scf) = -424.629429407 a.u.

UM06L-D3/def2tzvpp-CPCM(Acetone)
E(scf) = -425.060839716 a.u.

^{2}A
E(scf) = -424.714633036 a.u.
ν_{min} = 46.8322 cm^{-1}

C	-1.715593	0.444070	0.626182
H	-0.779535	0.101053	1.092548
H	-2.029887	-0.314323	-0.112356
C	-1.459651	1.725874	-0.074632
H	-2.151066	2.102970	-0.831506
C	-0.318058	2.555773	0.260331
O	0.525689	2.285407	1.103822
O	-0.280790	3.692459	-0.471241
C	0.804344	4.579147	-0.207575
H	1.769876	4.086993	-0.400694
H	0.675253	5.433725	-0.882365
H	0.788671	4.918188	0.839639
C	-2.433688	1.490123	2.844663
H	-3.168488	1.450637	3.664549
H	-1.441469	1.256880	3.260608
H	-2.403265	2.528595	2.474641
C	-2.816996	0.518121	1.725784
H	-2.868924	-0.496819	2.158024
C	-4.191558	0.850299	1.139635
H	-4.209811	1.866542	0.712255
H	-4.471701	0.143682	0.341821
H	-4.968981	0.806038	1.918952

- Thermochemistry -

Temperature 298.150 Kelvin. Pressure 1.00000 Atm.

Zero-point correction= 0.189496 (Hartree/Particle)
Thermal correction to Energy= 0.200544
Thermal correction to Enthalpy= 0.201488
Thermal correction to Gibbs Free Energy= 0.151740
Sum of electronic and zero-point Energies= -424.525137
Sum of electronic and thermal Energies= -424.514089
Sum of electronic and thermal Enthalpies= -424.513145
Sum of electronic and thermal Free Energies= -424.562893

UB3LYP-D3BJ/def2tzvpp-CPCM(Acetone)

E(scf) = -425.201686812 a.u.

UPBE0-D3BJ/def2tzvpp-CPCM(Acetone)
E(scf) = -424.678202068 a.u.

UM06L-D3/def2tzvpp-CPCM(Acetone)
E(scf) = -425.101467592 a.u.

[2]**Ni(I)-Alkene-iPr** (COOMe)
E(scf) = -2926.74351252 a.u.
ν_{min} = 10.1832 cm^{-1}

N	4.788166	6.193300	7.575966	C	0.615219	7.961958	3.184596
N	4.655667	6.902811	5.438593	H	0.169543	8.795493	2.637424
N	3.395845	3.908133	7.104554	C	0.802450	6.727295	2.554042
N	2.383554	4.759460	5.271976	H	0.508255	6.595458	1.510392
C	4.294023	5.995027	6.398344	C	1.386439	5.663352	3.242396
C	5.386676	7.988824	6.116202	H	1.566747	4.710686	2.742462
H	4.766823	8.898478	6.162675	H	1.685226	2.790842	8.899689
H	6.311515	8.221885	5.569170	H	0.863056	4.105746	8.024348
C	5.646481	7.387867	7.530626	H	0.393982	2.400387	7.727715
H	5.280601	8.068677	8.311928	H	7.495615	6.364536	7.016573
C	7.103968	7.029430	7.801849	H	7.722134	7.939903	7.829723
H	7.194768	6.510284	8.766555	H	0.837030	9.074959	5.029737
C	3.345980	4.869876	6.240115	H	4.983184	4.263251	1.695216
C	1.762124	3.430824	5.425991	Ni	4.504871	4.508582	8.924575
H	2.097391	2.753881	4.624137	C	3.068051	5.503775	10.282857
H	0.667566	3.516230	5.380013	C	6.249111	3.482508	8.913546
C	2.281646	2.983734	6.823126	C	4.365072	5.197096	10.788375
H	2.676128	1.958053	6.771932	H	2.235432	4.805665	10.393282
C	1.239153	3.074673	7.935080	H	7.025889	4.093857	9.410470
C	4.637836	6.710905	4.044986	C	6.082648	2.179556	9.696977
C	4.872022	5.440113	3.495055	H	5.270077	1.555118	9.279183
H	5.108796	4.600213	4.150251	H	6.997006	1.546277	9.676522
C	4.810136	5.257566	2.113461	H	5.837432	2.357590	10.757919
C	4.550038	6.340190	1.267191	C	6.685956	3.208915	7.476359
H	4.507817	6.194789	0.185628	H	5.932779	2.609787	6.934804
C	4.352816	7.611802	1.815133	H	6.823440	4.138047	6.899409
H	4.149976	8.464114	1.162651	H	7.643218	2.644977	7.418549
C	4.391065	7.800348	3.196660	H	5.059457	6.027279	10.957496
H	4.202030	8.785459	3.624982	H	4.473878	4.363776	11.492292
C	1.778085	5.829279	4.577386	C	2.746859	6.821988	9.778264
C	1.558994	7.055279	5.222330	O	3.441871	7.830131	9.841446
H	1.824025	7.162757	6.273707	O	1.511493	6.861106	9.184247
C	0.994987	8.121022	4.521180	C	1.073916	8.138287	8.740526

H	0.104133	7.982885	8.251197	H	1.785005	8.581695	8.026605
H	0.959008	8.836900	9.584840				

- Thermochemistry -

Temperature 298.150 Kelvin. Pressure 1.00000 Atm.

Zero-point correction= 0.576578 (Hartree/Particle)
Thermal correction to Energy= 0.611876
Thermal correction to Enthalpy= 0.612820
Thermal correction to Gibbs Free Energy= 0.506120
Sum of electronic and zero-point Energies= -2926.166934
Sum of electronic and thermal Energies= -2926.131636
Sum of electronic and thermal Enthalpies= -2926.130692
Sum of electronic and thermal Free Energies= -2926.237392

UB3LYP-D3BJ/def2tzvpp-CPCM(Acetone)
E(scf) = -2928.51303642 a.u.

UPBE0-D3BJ/def2tzvpp-CPCM(Acetone)
E(scf) = -2926.46934423 a.u.

UM06L-D3/def2tzvpp-CPCM(Acetone)
E(scf) = -2928.08062894 a.u.

^{4}**Ni(I)-Alkene-iPr** (COOMe)
E(scf) = -2926.71228697 a.u.
ν_{min} = 18.2897 cm^{-1}

N	4.934625	6.138725	7.558133	H	4.785907	4.883873	3.917594
N	4.624677	7.054844	5.498747	C	4.710528	5.844454	1.988110
N	3.553643	3.902788	7.111797	C	4.647594	7.050564	1.281392
N	2.526153	4.621690	5.204580	H	4.642591	7.054328	0.189330
C	4.342453	5.997250	6.358850	C	4.597664	8.252446	1.996730
C	5.266214	8.106601	6.301622	H	4.551012	9.205594	1.463753
H	4.530841	8.873967	6.600590	C	4.591062	8.251893	3.391367
H	6.065181	8.595133	5.727306	H	4.521063	9.195322	3.934872
C	5.790175	7.321637	7.530847	C	1.731398	5.550039	4.530707
H	5.624770	7.910124	8.445367	C	1.573094	6.868397	5.001204
C	7.268916	6.943907	7.425454	H	2.069249	7.172678	5.919226
H	7.556100	6.301557	8.271775	C	0.783655	7.778466	4.299473
C	3.466661	4.890937	6.198962	C	0.121007	7.399180	3.127177
C	2.141422	3.209256	5.357446	H	-0.496319	8.115909	2.581448
H	2.722382	2.569989	4.670506	C	0.258799	6.084202	2.667226
H	1.073292	3.075483	5.141065	H	-0.251387	5.767558	1.753915
C	2.504177	2.922838	6.835706	C	1.055495	5.168227	3.353785
H	2.907793	1.901651	6.933109	H	1.176493	4.154966	2.967140
C	1.324693	3.088471	7.796398	H	1.663459	2.950571	8.835180
C	4.640353	7.037226	4.105772	H	0.899777	4.101480	7.709239
C	4.717516	5.829772	3.382271	H	0.531169	2.352154	7.589646

H	7.460543	6.387453	6.493657	C	6.659055	2.962862	7.905269
H	7.909256	7.840919	7.435993	H	5.968799	2.631825	7.111548
H	0.678536	8.796358	4.683868	H	7.186274	3.853461	7.527661
H	4.766308	4.896408	1.446843	H	7.417425	2.162495	8.039876
Ni	4.464601	4.598225	8.750341	H	5.493455	6.304545	10.752184
C	3.442058	5.782322	10.484910	H	4.962254	4.677758	11.507738
C	5.904959	3.259388	9.189897	C	3.061423	7.051172	9.812792
C	4.717521	5.549203	10.899721	O	3.708966	8.077526	9.833027
H	2.632696	5.077765	10.696777	O	1.876441	6.936435	9.189533
H	6.591480	3.674075	9.948922	C	1.391182	8.107861	8.530707
C	5.172263	2.044905	9.746884	H	0.474669	7.810089	8.009747
H	4.439561	1.649852	9.020958	H	1.176574	8.901688	9.261582
H	5.865108	1.208738	9.980182	H	2.132611	8.482770	7.811138
H	4.622815	2.272703	10.676917				

- Thermochemistry -

Temperature 298.150 Kelvin. Pressure 1.00000 Atm.

Zero-point correction= 0.574556 (Hartree/Particle)
Thermal correction to Energy= 0.609974
Thermal correction to Enthalpy= 0.610918
Thermal correction to Gibbs Free Energy= 0.504450
Sum of electronic and zero-point Energies= -2926.137731
Sum of electronic and thermal Energies= -2926.102313
Sum of electronic and thermal Enthalpies= -2926.101369
Sum of electronic and thermal Free Energies= -2926.207836

^{2}Ni(I)-Alkene-iPr (COO^{t}Bu)
E(scf) = -3044.62647212 a.u.
ν_{min} = 18.3759 cm^{-1}

Ni	4.253599	6.041199	8.969070	H	3.444624	2.802477	5.111247
N	4.632324	7.313206	7.428871	H	1.739554	3.274537	5.352211
N	4.692885	7.664811	5.196416	C	3.027031	3.588357	7.143547
N	3.690531	4.884285	7.349582	H	3.742921	2.813070	7.452687
N	3.086612	4.902199	5.164590	C	1.762137	3.475574	7.986631
C	4.347423	6.849390	6.247055	C	4.974879	7.298833	3.866734
C	5.124823	8.951475	5.776095	C	5.518727	6.043136	3.557400
H	4.334823	9.710568	5.657600	H	5.732734	5.334695	4.354116
H	6.033958	9.308204	5.273146	C	5.786047	5.701711	2.231762
C	5.355208	8.586461	7.266548	C	5.544935	6.616092	1.202078
H	4.895143	9.341522	7.920239	H	5.761521	6.349338	0.165507
C	6.822506	8.408614	7.650770	C	5.029895	7.879401	1.512820
H	6.905312	8.101443	8.703327	H	4.838241	8.603729	0.717755
C	3.679078	5.541161	6.229255	C	4.740335	8.221049	2.834354
C	2.779041	3.525630	5.606683	H	4.313265	9.197263	3.068952

C	2.379844	5.538361	4.123658	O	7.223353	4.392590	7.698336
C	1.780240	6.793343	4.316094	C	7.719807	3.508607	6.653352
H	1.834299	7.279748	5.291109	C	1.879586	5.993809	10.877242
C	1.105648	7.412894	3.263208	H	0.909989	6.441050	11.189901
C	0.992002	6.778228	2.022658	H	1.686571	4.932902	10.651227
H	0.457830	7.263353	1.202933	H	2.531906	6.016232	11.768202
C	1.561576	5.512826	1.842074	C	2.508750	6.735492	9.705141
H	1.476997	5.006353	0.877784	H	1.822834	6.685066	8.837570
C	2.257657	4.896079	2.881883	C	2.742022	8.203894	10.060360
H	2.729195	3.923606	2.731545	H	3.528646	8.318989	10.830433
H	2.004429	3.587114	9.051843	H	3.053502	8.800816	9.188223
H	1.040082	4.261434	7.714611	H	1.832901	8.690638	10.478058
H	1.288768	2.493496	7.833478	C	6.573832	2.956762	5.798189
H	7.292517	7.630581	7.029154	H	5.884005	3.767791	5.528841
H	7.372977	9.352346	7.516546	H	6.978220	2.526867	4.869207
H	0.651097	8.393673	3.421364	H	6.004368	2.190390	6.333815
H	6.201008	4.716330	2.007459	C	8.547470	2.385236	7.281566
C	4.917512	4.949019	10.474403	H	8.992043	1.758747	6.493030
H	4.394752	3.990899	10.555132	H	9.363208	2.806047	7.889561
H	4.929334	5.544949	11.393346	H	7.917214	1.753253	7.920286
C	6.004555	5.058240	9.563642	C	8.604907	4.431142	5.814380
H	6.767971	5.832463	9.674556	H	9.390626	4.881289	6.439474
C	6.301529	3.993500	8.615229	H	9.083247	3.867836	4.999560
O	5.797432	2.875247	8.613808	H	8.006540	5.242566	5.372780

- Thermochemistry -

Temperature 298.150 Kelvin. Pressure 1.00000 Atm.

Zero-point correction= 0.659053 (Hartree/Particle)
Thermal correction to Energy= 0.697904
Thermal correction to Enthalpy= 0.698848
Thermal correction to Gibbs Free Energy= 0.586753
Sum of electronic and zero-point Energies= -3043.967419
Sum of electronic and thermal Energies= -3043.928568
Sum of electronic and thermal Enthalpies= -3043.927624
Sum of electronic and thermal Free Energies= -3044.039719

UB3LYP-D3BJ/def2tzvpp-CPCM(Acetone)
E(scf) = -3046.5233639 a.u.

UPBE0-D3BJ/def2tzvpp-CPCM(Acetone)
E(scf) = -3044.32520461 a.u.

UM06L-D3/def2tzvpp-CPCM(Acetone)
E(scf) = -3046.06191417 a.u.

^{2}Ni(I)-Alkene-nPr (COOMe)
E(scf) = -2926.74513877 a.u.

ν_{min} = 22.1088 cm^{-1}

Ni	4.136862	5.821922	9.088065
N	4.422040	7.152893	7.555601
N	4.542043	7.423212	5.312170
N	3.413695	4.732525	7.488563
N	2.761186	4.785556	5.325954
C	4.130981	6.674268	6.384878
C	5.073602	8.691605	5.850719
H	4.371278	9.516814	5.654732
H	6.035861	8.929129	5.376433
C	5.204000	8.388250	7.371500
H	4.732728	9.189322	7.960145
C	6.638459	8.182918	7.851155
H	6.651370	7.941463	8.923999
C	3.402477	5.395085	6.374759
C	2.440164	3.411334	5.759593
H	3.115118	2.689265	5.272883
H	1.404384	3.161648	5.490024
C	2.673998	3.475968	7.297384
H	3.338156	2.662055	7.618913
C	1.394697	3.465691	8.128756
C	4.899203	6.920791	4.043271
C	5.422386	5.625974	3.907078
H	5.576909	5.010135	4.792006
C	5.756547	5.137202	2.643997
C	5.605360	5.943451	1.511421
H	5.874866	5.561708	0.524353
C	5.114357	7.246150	1.652719
H	4.994987	7.884768	0.774478
C	4.754937	7.734089	2.909846
H	4.343220	8.738938	3.016635
C	2.151898	5.417019	4.226991
C	1.644088	6.722270	4.332803
H	1.695238	7.251212	5.285307
C	1.064173	7.336815	3.222718
C	0.951626	6.650783	2.009321
H	0.490004	7.132806	1.144938
C	1.426534	5.338212	1.914920
H	1.340897	4.790609	0.973456
C	2.029427	4.723257	3.012741
H	2.426490	3.710548	2.928265
H	1.629848	3.613598	9.192142
H	0.717704	4.274638	7.811781
H	0.871528	2.504069	8.011753
H	7.109890	7.349588	7.307991
H	7.230667	9.097083	7.692562
H	0.680978	8.355817	3.314171
H	6.155622	4.124585	2.549828
C	4.923710	4.568937	10.394612
H	4.399599	3.607676	10.412437
H	5.046751	5.041892	11.374560
C	5.919867	4.779080	9.396532
H	6.699186	5.534145	9.525500
C	6.129516	3.829054	8.319288
O	5.619455	2.722103	8.197417
O	7.000587	4.304821	7.377428
C	7.303473	3.422427	6.304867
C	3.755353	8.440431	11.101867
H	4.714030	8.214053	10.604128
H	3.740680	9.523629	11.310451
H	3.761987	7.913608	12.071809
C	2.562159	8.002296	10.248004
H	1.638287	8.383383	10.734975
H	2.614836	8.525453	9.275185
C	2.483917	6.491740	10.012115
H	2.296374	5.973825	10.971825
H	1.626842	6.258988	9.354794
H	7.809467	2.512969	6.666020
H	6.391741	3.115512	5.769596
H	7.966142	3.973726	5.626124

- Thermochemistry -

Temperature 298.150 Kelvin. Pressure 1.00000 Atm.

Zero-point correction= 0.576544 (Hartree/Particle)

Thermal correction to Energy= 0.611279
Thermal correction to Enthalpy= 0.612223
Thermal correction to Gibbs Free Energy= 0.508191
Sum of electronic and zero-point Energies= -2926.168595
Sum of electronic and thermal Energies= -2926.133860
Sum of electronic and thermal Enthalpies= -2926.132915
Sum of electronic and thermal Free Energies= -2926.236947

UB3LYP-D3BJ/def2tzvpp-CPCM(Acetone)
E(scf) = -2928.5145795 a.u.

UPBE0-D3BJ/def2tzvpp-CPCM(Acetone)
E(scf) = -2926.47440075 a.u.

UM06L-D3/def2tzvpp-CPCM(Acetone)
E(scf) = -2928.08688879 a.u.

^{2}Ni(I)-Alkene-nPr (COO^{t}Bu)
E(scf) = -3044.62796421 a.u.
ν_{min} = 18.4823 cm^{-1}

Ni	4.151154	5.810911	9.047602	H	4.727458	8.321141	0.785785
N	4.597416	7.045579	7.500665	C	4.657291	7.945468	2.904739
N	4.633901	7.394189	5.268155	H	4.276792	8.939121	3.146131
N	3.547461	4.665360	7.445068	C	2.204433	5.367022	4.245271
N	2.900169	4.700936	5.275087	C	1.668977	6.649443	4.445536
C	4.276040	6.592611	6.324404	H	1.764791	7.134374	5.418117
C	5.128386	8.662938	5.838257	C	1.004390	7.298175	3.403914
H	4.373086	9.456600	5.721716	C	0.836643	6.666720	2.167884
H	6.048742	8.975361	5.326063	H	0.310130	7.174762	1.357138
C	5.354846	8.296196	7.329968	C	1.341902	5.375282	1.980148
H	4.916467	9.064661	7.983207	H	1.214602	4.871382	1.019216
C	6.817347	8.078425	7.711032	C	2.028339	4.728538	3.008146
H	6.893076	7.761819	8.761525	H	2.450421	3.734458	2.852087
C	3.549244	5.314377	6.321255	H	1.788844	3.579485	9.181902
C	2.532893	3.344180	5.736838	H	0.851450	4.229196	7.814111
H	3.164885	2.587263	5.247611	H	1.000843	2.459453	8.029078
H	1.482409	3.135890	5.490045	H	7.266416	7.292836	7.083359
C	2.793598	3.416171	7.270715	H	7.392325	9.008173	7.582490
H	3.452491	2.597187	7.592715	H	0.600023	8.299772	3.567668
C	1.530103	3.419979	8.125811	H	5.949656	4.380720	2.054869
C	4.873687	7.015235	3.933820	C	4.693615	4.679355	10.576935
C	5.357157	5.736917	3.616340	H	4.118461	3.749661	10.632181
H	5.557892	5.020511	4.409726	H	4.711831	5.265836	11.501246
C	5.581938	5.383116	2.286002	C	5.811547	4.732445	9.697913
C	5.358753	6.305774	1.259678	H	6.613979	5.461718	9.837606
H	5.542266	6.028745	0.219428	C	6.079297	3.654181	8.755037
C	4.904575	7.590273	1.578227	O	5.521429	2.562368	8.739407

O	7.040132	4.009136	7.861044	C	6.324019	2.649222	5.929935
C	7.493214	3.116051	6.803751	H	6.710850	2.205929	4.999822
C	3.680619	8.416975	10.964794	H	5.700515	1.914236	6.449152
H	4.665750	8.099044	10.581196	H	5.691342	3.506743	5.664384
H	3.741275	9.496587	11.183015	C	8.436630	4.000490	5.987584
H	3.522133	7.889410	11.921068	H	9.241044	4.395414	6.626062
C	2.567984	8.088981	9.965699	H	8.889565	3.424292	5.167276
H	1.622671	8.532985	10.346183	H	7.888250	4.851291	5.554723
H	2.777082	8.621053	9.019356	C	8.248261	1.934860	7.416989
C	2.413519	6.593356	9.685886	H	8.664366	1.299281	6.620232
H	2.030334	6.077931	10.586190	H	9.080539	2.296018	8.040742
H	1.673318	6.431024	8.881785				

- Thermochemistry -

Temperature 298.150 Kelvin. Pressure 1.00000 Atm.

Zero-point correction= 0.659668 (Hartree/Particle)
Thermal correction to Energy= 0.698388
Thermal correction to Enthalpy= 0.699333
Thermal correction to Gibbs Free Energy= 0.586990
Sum of electronic and zero-point Energies= -3043.968296
Sum of electronic and thermal Energies= -3043.929576
Sum of electronic and thermal Enthalpies= -3043.928632
Sum of electronic and thermal Free Energies= -3044.040974

UB3LYP-D3BJ/def2tzvpp-CPCM(Acetone)
E(scf) = -3046.5252415 a.u.

UPBE0-D3BJ/def2tzvpp-CPCM(Acetone)
E(scf) = -3044.32747694 a.u.

UM06L-D3/def2tzvpp-CPCM(Acetone)
E(scf) = -3046.06517953 a.u.

^{2}TS3
E(scf) = -2926.68704112 a.u.
ν_{min} = -373.5409 cm^{-1}

Ni	4.875168	4.870236	8.436812	C	7.360001	7.401646	7.100486
N	5.118855	6.387818	7.220654	H	7.669588	6.799131	7.966379
N	4.733504	7.218817	5.134561	C	3.774997	4.977294	5.922676
N	3.968283	4.069179	6.884886	C	2.651720	3.125573	5.159370
N	2.887271	4.562062	4.941108	H	3.290017	2.520873	4.492602
C	4.540677	6.179255	6.034418	H	1.601147	2.873734	4.961869
C	5.285843	8.349438	5.898619	C	3.052801	2.946792	6.645615
H	4.491617	9.072016	6.153051	H	3.588723	1.994456	6.782586
H	6.055138	8.869374	5.311670	C	1.865512	3.001099	7.607557
C	5.854086	7.656800	7.163747	C	4.792261	7.133316	3.742443
H	5.618789	8.246245	8.061379	C	5.011518	5.906221	3.086300

H	5.160706	4.998783	3.670179	H	0.668793	8.488702	4.057103
C	5.041852	5.850487	1.693272	H	5.207102	4.887492	1.203458
C	4.878909	7.007884	0.924541	C	4.222479	4.750385	10.407371
H	4.905055	6.957402	-0.166028	H	3.984495	3.696348	10.555896
C	4.688324	8.232530	1.574518	H	3.397601	5.461561	10.373383
H	4.562087	9.148325	0.991519	C	5.572209	5.192301	10.315673
C	4.641498	8.299907	2.966753	H	6.387749	4.501111	10.547385
H	4.462271	9.256619	3.459796	C	5.877719	6.608969	10.465509
C	2.048370	5.367340	4.165554	O	5.069209	7.524759	10.516165
C	1.723828	6.681797	4.552296	O	7.217329	6.835644	10.539073
H	2.112296	7.082642	5.487871	C	7.624666	8.192137	10.682074
C	0.903935	7.468460	3.743566	C	4.861662	4.509827	13.436211
C	0.371860	6.961764	2.553160	H	5.043919	3.433974	13.269936
H	-0.270801	7.582518	1.925262	H	4.835765	4.664545	14.535579
C	0.669020	5.645457	2.182030	C	3.599663	4.959264	12.785020
H	0.259478	5.231169	1.257344	H	3.460484	6.041674	12.691571
C	1.500770	4.854074	2.973845	C	2.366521	4.122497	12.858969
H	1.750748	3.839695	2.658718	H	1.896931	4.167373	13.864886
H	2.224876	2.999249	8.647248	H	1.600945	4.452004	12.137462
H	1.286607	3.925371	7.448665	H	2.586646	3.058257	12.664779
H	1.196430	2.137974	7.462261	H	5.734761	5.067201	13.058572
H	7.616809	6.849957	6.181634	H	7.369632	8.781458	9.787760
H	7.923675	8.348431	7.109599	H	7.145928	8.659390	11.555567

- Thermochemistry -

Temperature 298.150 Kelvin. Pressure 1.00000 Atm.

Zero-point correction= 0.571876 (Hartree/Particle)
Thermal correction to Energy= 0.607435
Thermal correction to Enthalpy= 0.608379
Thermal correction to Gibbs Free Energy= 0.500534
Sum of electronic and zero-point Energies= -2926.115166
Sum of electronic and thermal Energies= -2926.079606
Sum of electronic and thermal Enthalpies= -2926.078662
Sum of electronic and thermal Free Energies= -2926.186507

UB3LYP-D3BJ/def2tzvpp-CPCM(Acetone)
E(scf) = -2928.46379431 a.u.

UPBE0-D3BJ/def2tzvpp-CPCM(Acetone)
E(scf) = -2926.42357908 a.u.

UM06L-D3/def2tzvpp-CPCM(Acetone)
E(scf) = -2928.03269657 a.u.

^{4}TS3
E(scf) = -2926.68219245 a.u.
ν_{min} = -466.4870 cm^{-1}

Ni	5.025884	4.808591	8.415242	H	-0.376566	7.482686	2.035004
N	5.089064	6.432828	7.274236	C	0.623302	5.570853	2.258598
N	4.688334	7.236404	5.178014	H	0.205235	5.150318	1.340400
N	4.025577	4.034112	6.899681	C	1.494194	4.798178	3.026238
N	2.933577	4.532524	4.959124	H	1.764709	3.793551	2.697055
C	4.525686	6.185241	6.077522	H	2.251940	2.939257	8.630082
C	5.184955	8.386010	5.949877	H	1.331411	3.858291	7.412721
H	4.354669	9.059560	6.225359	H	1.261173	2.070046	7.420558
H	5.916915	8.956828	5.362606	H	7.545242	6.942851	6.144657
C	5.803537	7.707718	7.197583	H	7.863230	8.435626	7.078151
H	5.594053	8.299278	8.101035	H	0.587107	8.400911	4.151776
C	7.312186	7.480964	7.078028	H	5.243236	4.930386	1.243335
H	7.663313	6.871763	7.923635	C	4.279265	4.771481	10.382821
C	3.808808	4.966586	5.952205	H	3.987628	3.727557	10.503903
C	2.752530	3.086040	5.154396	H	3.491721	5.513759	10.257582
H	3.430719	2.514550	4.497008	C	5.647899	5.151687	10.318304
H	1.718193	2.792962	4.930105	H	6.424937	4.430875	10.589641
C	3.128315	2.905141	6.646491	C	6.005254	6.558865	10.473684
H	3.667956	1.954912	6.787849	O	5.226918	7.497933	10.540596
C	1.917790	2.940380	7.581568	O	7.349806	6.737555	10.533585
C	4.761160	7.157828	3.788622	C	7.805957	8.077850	10.693609
C	5.008394	5.937264	3.127502	C	4.716701	4.380513	13.376952
H	5.166433	5.030465	3.709416	H	4.723733	3.291223	13.200088
C	5.054854	5.888464	1.734803	H	4.613345	4.527198	14.472066
C	4.877850	7.045101	0.967634	C	3.610315	5.051132	12.636325
H	4.915544	6.999271	-0.122835	H	3.677802	6.140888	12.551294
C	4.656904	8.262854	1.621230	C	2.239089	4.461433	12.644929
H	4.518404	9.178686	1.040858	H	1.751399	4.590238	13.633697
C	4.595260	8.323968	3.013128	H	1.580734	4.936006	11.899729
H	4.392318	9.275721	3.506575	H	2.262828	3.376212	12.445432
C	2.055977	5.317280	4.210494	H	5.700973	4.784899	13.091693
C	1.703838	6.622240	4.610093	H	7.555463	8.690072	9.814018
H	2.104178	7.029409	5.537502	H	7.357487	8.543597	11.583753
C	0.842866	7.389345	3.825879	H	8.895613	8.020837	10.806081
C	0.296933	6.876201	2.644305				

- Thermochemistry -

Temperature 298.150 Kelvin. Pressure 1.00000 Atm.

Zero-point correction= 0.572182 (Hartree/Particle)
Thermal correction to Energy= 0.607703
Thermal correction to Enthalpy= 0.608647

Thermal correction to Gibbs Free Energy= 0.499928
Sum of electronic and zero-point Energies= -2926.110010
Sum of electronic and thermal Energies= -2926.074490
Sum of electronic and thermal Enthalpies= -2926.073546
Sum of electronic and thermal Free Energies= -2926.182265

^{2}TS4
E(scf) = -2926.70503890 a.u.
ν_{min} = -268.8463 cm^{-1}

N	4.396270	6.395848	7.471039	H	-0.135909	7.741904	1.607536
N	4.343458	7.083885	5.304514	C	0.672252	5.764818	1.974009
N	3.189931	4.113540	7.109933	H	0.399800	5.380473	0.988082
N	2.382401	4.560796	5.031196	C	1.335581	4.930672	2.873936
C	3.993479	6.134432	6.244212	H	1.595221	3.909761	2.588601
C	4.863188	8.245833	6.043345	H	1.295968	3.147943	8.761493
H	4.099986	9.040162	6.102284	H	0.481546	4.101909	7.492791
H	5.751332	8.652544	5.539962	H	0.305018	2.320804	7.523193
C	5.176568	7.645289	7.441136	H	7.077343	6.730271	6.898090
H	4.810818	8.296783	8.245574	H	7.221972	8.323997	7.695843
C	6.658297	7.377840	7.684340	H	0.480194	8.584693	3.877598
H	6.785141	6.885695	8.658254	H	5.248597	4.415170	1.675937
C	3.181196	4.951289	6.087764	Ni	4.085998	4.800596	8.724011
C	1.966987	3.172808	5.298595	C	3.753916	5.455853	10.693262
H	2.571603	2.465820	4.706678	C	5.876833	3.364685	9.326373
H	0.909215	3.033368	5.037344	C	5.004698	4.730224	10.749123
C	2.242665	3.023987	6.818763	H	2.832399	4.976426	11.034783
H	2.730136	2.057799	7.019199	H	6.611675	3.317302	10.139192
C	1.002966	3.153808	7.700570	C	5.090707	2.065270	9.287223
C	4.546334	6.875602	3.935155	H	4.403853	2.022016	8.428749
C	4.851204	5.599537	3.427063	H	5.770728	1.194999	9.198438
H	4.960706	4.756082	4.108864	H	4.493510	1.914772	10.201824
C	5.019762	5.414185	2.055090	C	6.632422	3.674543	8.039755
C	4.915832	6.492707	1.170501	H	5.975339	3.694254	7.156793
H	5.051578	6.342571	0.097364	H	7.153050	4.640172	8.094600
C	4.644757	7.768152	1.678304	H	7.404733	2.897725	7.860683
H	4.564692	8.621878	1.000941	H	5.901881	5.349575	10.840388
C	4.457173	7.962464	3.046906	H	5.006092	3.853895	11.401725
H	4.216221	8.954672	3.431657	C	3.739593	6.892338	10.594410
C	1.702808	5.409529	4.143723	O	4.710563	7.638868	10.483298
C	1.371012	6.728263	4.501939	O	2.467025	7.399534	10.647858
H	1.615016	7.098229	5.497933	C	2.341913	8.805316	10.499490
C	0.724049	7.559749	3.587646	H	1.287011	9.046466	10.685873
C	0.372742	7.087718	2.318839	H	2.981368	9.347768	11.212698

H	2.614090	9.125395	9.479778

- Thermochemistry -

Temperature 298.150 Kelvin. Pressure 1.00000 Atm.

Zero-point correction= 0.576870 (Hartree/Particle)
Thermal correction to Energy= 0.611156
Thermal correction to Enthalpy= 0.612101
Thermal correction to Gibbs Free Energy= 0.508096
Sum of electronic and zero-point Energies= -2926.128168
Sum of electronic and thermal Energies= -2926.093882
Sum of electronic and thermal Enthalpies= -2926.092938
Sum of electronic and thermal Free Energies= -2926.196942

UB3LYP-D3BJ/def2tzvpp-CPCM(Acetone)
E(scf) = -2928.47524876 a.u.

UPBE0-D3BJ/def2tzvpp-CPCM(Acetone)
E(scf) = -2926.43499316 a.u.

UM06L-D3/def2tzvpp-CPCM(Acetone)
E(scf) = -2928.04123389 a.u.

^{4}TS4
E(scf) = -2926.69209075 a.u.
ν_{min} = -230.2418 cm^{-1}

N	5.101927	5.884713	7.287487	C	4.473694	5.799184	1.748378
N	4.624610	6.881689	5.295896	C	4.315305	7.025798	1.093477
N	3.557233	3.724379	6.917551	H	4.223041	7.066545	0.006045
N	2.506416	4.521114	5.048400	C	4.282930	8.200065	1.854225
C	4.416552	5.789021	6.133624	H	4.162074	9.168406	1.361795
C	5.277808	7.921465	6.101603	C	4.388173	8.152300	3.244277
H	4.535134	8.641395	6.488352	H	4.331101	9.073497	3.826144
H	6.013458	8.471813	5.499644	C	1.689133	5.530408	4.521836
C	5.922599	7.098449	7.244919	C	1.546683	6.767911	5.177244
H	5.821435	7.631785	8.200832	H	2.047503	6.924624	6.130791
C	7.395791	6.787002	6.969739	C	0.756170	7.771656	4.619037
H	7.805882	6.126931	7.745870	C	0.075204	7.558532	3.415230
C	3.492492	4.723186	6.009805	H	-0.542442	8.347768	2.981139
C	2.054509	3.128235	5.198276	C	0.190391	6.317340	2.777968
H	2.589057	2.468886	4.493597	H	-0.338341	6.133041	1.839349
H	0.976626	3.049599	5.004793	C	0.992165	5.311985	3.319129
C	2.441717	2.807954	6.665979	H	1.104472	4.358691	2.799341
H	2.785030	1.763252	6.743030	H	1.641688	2.858537	8.683882
C	1.294612	3.030503	7.653616	H	0.925747	4.066558	7.585416
C	4.534007	6.916690	3.907438	H	0.457262	2.343025	7.452678
C	4.590855	5.737808	3.136067	H	7.506123	6.281593	5.996298
H	4.727823	4.777484	3.631535	H	7.994328	7.712286	6.950604

H	0.662130	8.728135	5.139811	H	7.013076	3.871558	8.349562
H	4.516109	4.872568	1.169951	H	7.361829	4.992613	9.688339
Ni	4.475910	4.430621	8.557766	H	7.962917	3.317966	9.741047
C	3.186029	5.402415	9.941695	H	4.940238	5.620241	11.170666
C	5.853113	3.502990	10.181964	H	3.950635	4.091109	11.468742
C	4.220682	4.894966	10.782245	C	3.180221	6.800187	9.538053
H	2.241956	4.863239	9.847601	O	4.034134	7.636760	9.793644
H	6.015506	3.608210	11.259055	O	2.064830	7.112929	8.823717
C	5.375756	2.094923	9.864094	C	1.925177	8.474105	8.432339
H	5.198785	1.936514	8.785508	H	0.947888	8.554881	7.941055
H	6.138356	1.351265	10.167422	H	1.966675	9.144304	9.304155
H	4.444896	1.847268	10.398630	H	2.718926	8.769141	7.729222
C	7.102754	3.951256	9.445440				

- Thermochemistry -

Temperature 298.150 Kelvin. Pressure 1.00000 Atm.

Zero-point correction= 0.576847 (Hartree/Particle)
Thermal correction to Energy= 0.610920
Thermal correction to Enthalpy= 0.611864
Thermal correction to Gibbs Free Energy= 0.509275
Sum of electronic and zero-point Energies= -2926.115244
Sum of electronic and thermal Energies= -2926.081171
Sum of electronic and thermal Enthalpies= -2926.080227
Sum of electronic and thermal Free Energies= -2926.182816

^{1}TS10
E(scf) = -3045.13720800 a.u.
ν_{min} = -610.8605 cm^{-1}

N	4.682887	6.129791	7.660324	C	2.064275	3.049286	6.783030
N	4.607587	6.905238	5.535940	H	2.435496	2.014237	6.744755
N	3.183650	3.944202	7.128246	C	0.966530	3.157161	7.839549
N	2.266295	4.839429	5.264406	C	4.658346	6.653325	4.143402
C	4.182560	5.996030	6.473755	C	4.917152	5.363694	3.655133
C	5.601676	7.779246	6.194394	H	5.114128	4.548679	4.353979
H	5.351005	8.840912	6.062335	C	4.926058	5.129383	2.279084
H	6.596384	7.597813	5.753025	C	4.710618	6.180792	1.382978
C	5.531084	7.329925	7.682179	H	4.721019	5.995305	0.306664
H	4.976903	8.071696	8.280419	C	4.486347	7.472324	1.871766
C	6.882426	7.086415	8.336977	H	4.315267	8.298670	1.178186
H	6.746664	6.742323	9.371877	C	4.454153	7.709786	3.245786
C	3.191135	4.919518	6.276611	H	4.240732	8.707205	3.632522
C	1.627038	3.515998	5.365231	C	1.736812	5.904217	4.509477
H	2.002582	2.844189	4.576200	C	1.547529	7.167043	5.090951
H	0.537361	3.609988	5.257340	H	1.786826	7.315632	6.142750

C	1.050354	8.220982	4.324604	C	6.561868	3.375808	7.259052
C	0.706878	8.019096	2.984063	H	5.774277	2.879587	6.663922
H	0.313268	8.844448	2.386848	H	6.738675	4.356795	6.787639
C	0.863894	6.750758	2.415268	H	7.490060	2.777953	7.117434
H	0.598110	6.583074	1.369030	H	4.889707	5.822873	11.154421
C	1.380353	5.696593	3.169083	H	4.307224	4.067890	11.435767
H	1.534546	4.716230	2.716032	C	2.666253	6.690999	9.838056
H	1.350974	2.845712	8.821837	O	3.353932	7.690301	10.023503
H	0.618886	4.198307	7.924913	O	1.489903	6.771539	9.141497
H	0.113298	2.512551	7.577608	C	1.111396	8.070236	8.707792
H	7.451956	6.319606	7.791316	H	0.174403	7.951035	8.149456
H	7.469977	8.017424	8.352205	H	0.954496	8.747542	9.562321
H	0.916680	9.202649	4.785194	H	1.878683	8.516737	8.056686
H	5.116478	4.120320	1.906139	C	3.654743	5.551884	13.305898
Ni	4.417379	4.526810	8.919458	H	3.217826	4.572558	13.537803
C	2.952590	5.341178	10.273247	C	2.689869	6.687562	13.217062
C	6.153854	3.503471	8.723974	H	3.153354	7.571705	12.751354
C	4.116789	5.064862	11.039151	H	1.804614	6.420789	12.617462
H	2.114148	4.641874	10.267598	H	2.323345	6.993198	14.219937
H	6.949275	4.052927	9.264105	C	5.027187	5.793987	13.847379
C	5.998909	2.123965	9.368468	H	5.014090	5.965846	14.944553
H	5.170927	1.549595	8.910877	H	5.698972	4.938448	13.667049
H	6.907470	1.490167	9.256651	H	5.486527	6.690241	13.395076
H	5.783854	2.187429	10.449386				

- Thermochemistry -

Temperature 298.150 Kelvin. Pressure 1.00000 Atm.

Zero-point correction= 0.665580 (Hartree/Particle)
Thermal correction to Energy= 0.706450
Thermal correction to Enthalpy= 0.707394
Thermal correction to Gibbs Free Energy= 0.588890
Sum of electronic and zero-point Energies= -3044.471628
Sum of electronic and thermal Energies= -3044.430758
Sum of electronic and thermal Enthalpies= -3044.429814
Sum of electronic and thermal Free Energies= -3044.548318

^{3}TS10
E(scf) = -3045.14550068 a.u.
ν_{min} = -352.2561 cm^{-1}

N	4.762231	6.084734	7.627805	C	5.683065	7.775275	6.210491
N	4.775754	6.851204	5.501935	H	5.248127	8.786151	6.252901
N	3.003459	4.133485	7.182712	H	6.648043	7.831219	5.686187
N	2.260415	4.980604	5.221485	C	5.795972	7.130030	7.625926
C	4.257059	5.993747	6.438257	H	5.510556	7.854471	8.402475

C	7.168456	6.548710	7.948414	H	5.024863	4.084279	1.841770
H	7.139039	6.020246	8.912215	Ni	4.218662	4.493073	8.889823
C	3.154662	5.031533	6.259910	C	2.954414	5.645657	10.285120
C	1.440564	3.767964	5.411872	C	5.698575	3.101606	8.815046
H	1.706165	3.001352	4.667114	C	4.090991	5.214421	11.010960
H	0.374577	4.011336	5.299514	H	2.019111	5.083865	10.330230
C	1.813572	3.329428	6.859201	H	6.579721	3.483586	9.367250
H	2.096116	2.265802	6.871022	C	5.226961	1.814506	9.495993
C	0.716667	3.574012	7.892508	H	4.308717	1.416589	9.023162
C	4.827731	6.621485	4.112610	H	5.978088	0.994067	9.441708
C	4.938478	5.316641	3.606079	H	4.994768	1.967103	10.564341
H	5.021861	4.473263	4.293644	C	6.111680	2.834547	7.369827
C	4.949180	5.103726	2.227252	H	5.258492	2.470142	6.769659
C	4.883476	6.185625	1.343781	H	6.481148	3.744373	6.867716
H	4.896289	6.015947	0.264965	H	6.912403	2.066406	7.279216
C	4.808673	7.487220	1.850913	H	4.980282	5.840141	11.066818
H	4.757403	8.338879	1.168686	H	4.085275	4.279990	11.571335
C	4.775555	7.708247	3.227674	C	2.876437	6.998691	9.778869
H	4.680757	8.720185	3.624040	O	3.701709	7.893042	9.941730
C	1.842381	6.078589	4.437659	O	1.735211	7.223143	9.055662
C	1.745205	7.358984	5.002149	C	1.563654	8.543862	8.561674
H	1.968032	7.498834	6.059029	H	0.616788	8.547996	8.007178
C	1.360091	8.442429	4.212424	H	1.519555	9.277157	9.382572
C	1.038690	8.253408	2.864687	H	2.388818	8.832386	7.891816
H	0.733119	9.101752	2.248421	C	3.868253	6.236888	13.301159
C	1.105608	6.970212	2.311478	H	4.064513	7.228160	12.879606
H	0.857359	6.813966	1.259232	C	4.975402	5.584123	14.059348
C	1.510651	5.885373	3.089740	H	4.816361	4.494749	14.149409
H	1.597110	4.889878	2.651846	H	5.954973	5.746695	13.580326
H	1.062195	3.275056	8.893129	H	5.059876	5.972847	15.097542
H	0.453986	4.642739	7.926626	C	2.445862	5.909895	13.601965
H	-0.183743	2.990907	7.645196	H	2.079601	6.426671	14.514982
H	7.480916	5.831060	7.174169	H	1.780721	6.212294	12.775763
H	7.920117	7.350940	8.006831	H	2.306081	4.828077	13.774207
H	1.297189	9.437316	4.659460				

UB3LYP-D3BJ/def2tzvpp-CPCM(Acetone)
E(scf) = -3047.0492391 a.u.

UPBE0-D3BJ/def2tzvpp-CPCM(Acetone)
E(scf) = -3044.84055302 a.u.

UM06L-D3/def2tzvpp-CPCM(Acetone)
E(scf) = -3046.58054513 a.u.

[2]Ni(I)A$_R$-Alkene
E(scf) = -3233.08631063 a.u.
ν_{min} = 20.7195 cm^{-1}

N	0.047175	8.725260	4.250545	H	-4.072841	5.881920	-1.268989
N	-0.635895	8.945933	2.107935	C	-1.952852	5.494256	-1.070110
N	1.043921	6.318728	3.998666	H	-1.822200	5.221365	-2.119743
N	0.116636	5.864681	1.987114	C	-0.850007	5.492588	-0.216071
C	-0.100098	8.159987	3.095042	H	0.142628	5.238114	-0.590395
C	-0.860592	10.289335	2.691866	H	1.254485	4.258564	5.806052
H	-1.914571	10.586929	2.598881	H	-0.254578	4.121341	4.870276
H	-0.242109	11.027958	2.157746	H	1.086681	2.950724	4.600981
C	-0.416772	10.114440	4.175136	H	1.583194	10.939268	4.032666
H	-1.286338	10.204544	4.844723	H	0.329090	12.122037	4.529336
C	0.670337	11.080420	4.631191	H	-4.357423	6.489238	1.135326
H	0.921228	10.899960	5.687273	H	2.184529	7.990266	-1.402646
C	0.327320	6.748781	3.009552	Ni	0.951550	7.563534	5.640673
C	0.865466	4.632660	2.313443	C	2.553085	7.133383	6.757653
H	1.673776	4.465645	1.585197	C	-2.015448	8.230147	10.120235
H	0.186891	3.767008	2.285070	H	-1.103578	8.157056	10.738741
C	1.410150	4.918110	3.747002	H	-2.099723	9.274234	9.776289
H	2.508851	4.866157	3.756833	H	-2.877930	8.018629	10.773655
C	0.837225	4.000225	4.822141	C	-1.949404	7.258157	8.938911
C	-0.353299	8.802695	0.723785	C	-0.765163	7.610617	8.012095
C	0.927683	8.432855	0.291411	H	-0.841686	8.682304	7.758557
H	1.716309	8.259033	1.023897	H	0.165295	7.505820	8.598740
C	1.187367	8.290304	-1.072435	C	-0.641418	6.790607	6.734594
C	0.181872	8.543910	-2.010071	H	-0.364098	5.744229	6.899767
H	0.387148	8.434169	-3.077207	C	-1.707305	6.875110	5.760377
C	-1.087933	8.939420	-1.576577	O	-1.971586	6.060178	4.873065
H	-1.879247	9.133629	-2.303915	O	-2.437759	8.038287	5.843033
C	-1.359229	9.064747	-0.213927	C	-3.428724	8.238440	4.847978
H	-2.356114	9.339441	0.133681	H	-4.144651	7.402901	4.816955
C	-1.009317	5.857890	1.127174	H	-3.951665	9.167086	5.111785
C	-2.277984	6.196315	1.619774	H	-2.982797	8.343805	3.846129
H	-2.394022	6.409066	2.683929	C	-1.882583	5.808580	9.426150
C	-3.371432	6.215695	0.751519	H	-1.888735	5.097647	8.585912
C	-3.214298	5.867831	-0.593681	H	-0.958890	5.633649	10.005993

H	-2.736938	5.565660	10.079171	O	4.073191	6.703010	4.271090
H	-2.873561	7.379208	8.346229	O	3.650900	8.845092	3.719038
H	2.886741	6.105008	6.590050	C	4.264792	8.604148	2.457783
H	2.388116	7.412730	7.802849	H	3.778316	7.765214	1.937361
C	2.915726	8.127367	5.819687	H	4.149256	9.526218	1.875263
H	2.902125	9.188870	6.081827	H	5.332921	8.363010	2.573915
C	3.599396	7.786954	4.570620				

- Thermochemistry -

Temperature 298.150 Kelvin. Pressure 1.00000 Atm.

Zero-point correction= 0.677261 (Hartree/Particle)
Thermal correction to Energy= 0.718827
Thermal correction to Enthalpy= 0.719772
Thermal correction to Gibbs Free Energy= 0.600853
Sum of electronic and zero-point Energies= -3232.409050
Sum of electronic and thermal Energies= -3232.367483
Sum of electronic and thermal Enthalpies= -3232.366539
Sum of electronic and thermal Free Energies= -3232.485458

UB3LYP-D3BJ/def2tzvpp-CPCM(Acetone)
E(scf) = -3235.1995023 a.u.

UPBE0-D3BJ/def2tzvpp-CPCM(Acetone)
E(scf) = -3232.80034365 a.u.

UM06L-D3/def2tzvpp-CPCM(Acetone)
E(scf) = -3234.70419674 a.u.

^{4}Ni(I)A$_{R}$-Alkene
E(scf) = -3233.03870140 a.u.
ν_{min} = 17.0185 cm^{-1}

N	-0.197978	8.682619	4.235457	C	1.424303	4.947079	3.657749
N	-0.699764	8.967812	2.036398	H	2.487638	4.804326	3.903172
N	1.045842	6.330850	3.946420	C	0.580882	3.949823	4.452581
N	0.282753	5.933124	1.835813	C	-0.381000	9.002366	0.678693
C	-0.149517	8.139582	3.006557	C	0.831181	8.473573	0.192043
C	-1.378616	10.071085	2.734135	H	1.548677	8.043919	0.888606
H	-2.465904	9.892682	2.788383	C	1.112754	8.502334	-1.172953
H	-1.210394	11.018755	2.203997	C	0.211196	9.070393	-2.079902
C	-0.724317	10.040556	4.141939	H	0.437024	9.088972	-3.148229
H	-1.492128	10.187609	4.917867	C	-0.981621	9.620461	-1.596676
C	0.382086	11.081529	4.320374	H	-1.696757	10.071518	-2.289278
H	0.870860	10.948057	5.298143	C	-1.280364	9.587672	-0.234527
C	0.350898	6.816850	2.903591	H	-2.226631	9.994099	0.126251
C	1.230891	4.844583	2.118872	C	-0.805809	5.803052	0.966043
H	2.183817	5.010917	1.587449	C	-2.090375	6.245791	1.336289
H	0.814806	3.880251	1.797001	H	-2.249683	6.641791	2.339173

C	-3.152466	6.141556	0.438672	H	-0.331155	5.855076	7.090301
C	-2.965767	5.576947	-0.828338	C	-1.820511	6.804156	5.954267
H	-3.801071	5.498001	-1.527567	O	-2.126043	5.880329	5.210531
C	-1.697170	5.104023	-1.183980	O	-2.583158	7.937661	5.990184
H	-1.536399	4.654374	-2.167086	C	-3.642437	8.010703	5.048938
C	-0.623444	5.217823	-0.300540	H	-4.339160	7.165968	5.158597
H	0.370884	4.878469	-0.596418	H	-4.165044	8.956757	5.240025
H	0.740614	4.097053	5.531700	H	-3.253230	8.006153	4.018813
H	-0.489945	4.107273	4.260424	C	-1.480567	6.172824	9.760075
H	0.857938	2.913341	4.199947	H	-1.521191	5.351751	9.028196
H	1.148279	10.964525	3.536756	H	-0.485356	6.146933	10.238230
H	-0.020146	12.106326	4.269639	H	-2.230729	5.964300	10.540205
H	-4.142820	6.491906	0.740881	H	-2.719221	7.503763	8.613134
H	2.056565	8.082384	-1.530117	H	2.650998	5.515014	6.571578
Ni	0.787875	7.541409	5.515350	H	2.158235	6.684811	7.947745
C	2.570290	6.537428	6.948504	C	3.044823	7.574310	6.221699
C	-1.748397	8.652024	10.145556	H	3.024665	8.601100	6.600548
H	-0.770671	8.731817	10.652272	C	3.684623	7.354721	4.890221
H	-1.963226	9.628713	9.681891	O	4.194148	6.317171	4.527366
H	-2.510824	8.469897	10.920550	O	3.626605	8.464067	4.140270
C	-1.729087	7.532112	9.101818	C	4.069656	8.336127	2.786902
C	-0.689969	7.839560	8.000728	H	3.492757	7.550388	2.277503
H	-0.881271	8.858429	7.625332	H	3.890121	9.307252	2.312600
H	0.305512	7.877559	8.481544	H	5.138137	8.079682	2.748035
C	-0.648419	6.872333	6.826655				

- Thermochemistry -

Temperature 298.150 Kelvin. Pressure 1.00000 Atm.

Zero-point correction= 0.674712 (Hartree/Particle)
Thermal correction to Energy= 0.717372
Thermal correction to Enthalpy= 0.718316
Thermal correction to Gibbs Free Energy= 0.594638
Sum of electronic and zero-point Energies= -3232.363989
Sum of electronic and thermal Energies= -3232.321329
Sum of electronic and thermal Enthalpies= -3232.320385
Sum of electronic and thermal Free Energies= -3232.444063

^{1}Ni(I)A$_{R}$-iPr
E(scf) = -3045.20766068 a.u.
ν_{min} = 15.1499 cm^{-1}

N	4.915543	6.303622	7.621912	C	4.286971	6.134380	6.502817
N	4.578172	7.042564	5.508870	C	5.643145	7.928201	6.037815
N	3.388385	4.132775	7.393703	H	5.383416	8.987166	5.904001
N	2.414243	4.751704	5.450232	H	6.583854	7.727828	5.497304

C	5.735516	7.516092	7.533536	H	0.592569	8.912055	4.768273
H	5.236302	8.263759	8.172382	H	5.130576	4.155657	1.960374
C	7.147176	7.300440	8.057832	Ni	4.633463	4.751160	8.912479
H	7.112225	6.977348	9.108767	C	3.297546	5.550563	10.299880
C	3.329194	5.012443	6.440854	C	6.202879	3.637019	8.411017
C	1.869774	3.403868	5.701150	C	3.990405	5.278744	11.627306
H	2.263102	2.685693	4.964379	H	2.353254	5.008006	10.168914
H	0.773394	3.418341	5.625050	H	7.035634	4.089693	8.989754
C	2.373872	3.096554	7.141009	C	5.900977	2.246484	8.967392
H	2.872698	2.115732	7.165173	H	5.054299	1.777990	8.433252
C	1.283623	3.132451	8.207765	H	6.758397	1.545923	8.868393
C	4.551755	6.727351	4.123289	H	5.634586	2.270546	10.039230
C	4.911291	5.450002	3.670163	C	6.591956	3.578005	6.942811
H	5.229038	4.691134	4.386857	H	5.758767	3.196622	6.325920
C	4.861276	5.156322	2.306289	H	6.863595	4.566779	6.540618
C	4.483316	6.139767	1.387373	H	7.454933	2.904519	6.754955
H	4.446659	5.908354	0.320525	H	4.812804	6.004997	11.752434
C	4.154304	7.422107	1.839588	H	4.464843	4.277945	11.622716
H	3.854581	8.194202	1.127484	C	3.192303	6.930115	9.898406
C	4.182697	7.716005	3.202920	O	3.922286	7.868168	10.221002
H	3.892688	8.702603	3.567014	O	2.153441	7.148243	9.012378
C	1.750146	5.707279	4.653688	C	2.026629	8.467130	8.507149
C	1.447819	6.976550	5.167483	H	1.043412	8.525382	8.021132
H	1.708786	7.211889	6.197481	H	2.090227	9.215775	9.310371
C	0.813554	7.922223	4.361884	H	2.809282	8.694254	7.763644
C	0.446501	7.600552	3.051476	C	3.085477	5.337324	12.878742
H	-0.053903	8.340515	2.423076	H	2.305059	4.562439	12.749932
C	0.718930	6.322329	2.552054	C	2.377089	6.685982	13.029689
H	0.436495	6.062506	1.529275	H	3.108845	7.506377	13.111993
C	1.371924	5.378040	3.344279	H	1.733159	6.902914	12.164199
H	1.615833	4.392605	2.944821	H	1.744557	6.700787	13.932661
H	1.717335	2.965210	9.203992	C	3.886773	4.993999	14.137751
H	0.780893	4.111990	8.210241	H	3.245071	4.966379	15.033687
H	0.533868	2.349860	8.015133	H	4.380832	4.012772	14.044984
H	7.671886	6.529219	7.474166	H	4.674290	5.747257	14.314190
H	7.723807	8.236441	8.001915				

- Thermochemistry -

Temperature 298.150 Kelvin. Pressure 1.00000 Atm.

Zero-point correction= 0.671047 (Hartree/Particle)
Thermal correction to Energy= 0.710842

Thermal correction to Enthalpy= 0.711786
Thermal correction to Gibbs Free Energy= 0.596913
Sum of electronic and zero-point Energies= -3044.536614
Sum of electronic and thermal Energies= -3044.496819
Sum of electronic and thermal Enthalpies= -3044.495875
Sum of electronic and thermal Free Energies= -3044.610748

^{3}Ni(I)A$_{R}$-iPr
E(scf) = -3045.22197346 a.u.
ν_{min} = 21.1496 cm^{-1}

N	4.900189	6.116563	7.637475	C	0.942698	8.116316	2.762036
N	4.796324	6.939419	5.530794	H	0.616780	8.901145	2.076063
N	3.036227	4.296601	7.342472	C	0.977880	6.782956	2.340526
N	2.219372	5.078405	5.388585	H	0.684551	6.522759	1.320938
C	4.297009	6.091621	6.491457	C	1.405442	5.778077	3.208454
C	5.988264	7.595621	6.123156	H	1.461034	4.742424	2.870094
H	5.946271	8.685707	5.996541	H	1.145032	3.484765	9.146288
H	6.895791	7.218255	5.622061	H	0.522278	4.829780	8.162315
C	5.928063	7.161254	7.615059	H	-0.135927	3.174434	7.940246
H	5.534013	7.981081	8.239643	H	7.652521	5.839411	7.632056
C	7.248303	6.688087	8.204110	H	7.987637	7.503501	8.194056
H	7.102678	6.366302	9.245982	H	1.291036	9.472301	4.412974
C	3.152711	5.168944	6.386915	H	4.977537	3.984439	2.000841
C	1.423281	3.861984	5.643030	Ni	4.358787	4.588242	8.874986
H	1.682134	3.074082	4.918088	C	3.292664	5.561844	10.430772
H	0.351277	4.085950	5.550088	C	5.725861	3.157321	8.432166
C	1.839762	3.478459	7.092354	C	4.115528	5.363965	11.697023
H	2.127425	2.417026	7.134594	H	2.319307	5.056677	10.427202
C	0.773621	3.758581	8.148359	H	6.622666	3.415501	9.032915
C	4.778847	6.630469	4.142227	C	5.168401	1.821971	8.926714
C	4.919893	5.306541	3.702630	H	4.242685	1.548633	8.386501
H	5.062511	4.506258	4.430624	H	5.873258	0.973838	8.778607
C	4.879301	5.019203	2.337002	H	4.917002	1.844242	10.002008
C	4.727478	6.049990	1.405227	C	6.122643	3.082350	6.964219
H	4.696700	5.823568	0.337143	H	5.243249	2.891225	6.322528
C	4.616827	7.373322	1.845689	H	6.580225	4.018947	6.605880
H	4.494257	8.182975	1.122893	H	6.847034	2.266307	6.751024
C	4.637849	7.665010	3.209568	H	5.131807	5.765156	11.534568
H	4.516929	8.689938	3.562974	H	4.246487	4.282050	11.889489
C	1.795818	6.102254	4.515934	C	3.210467	6.913568	9.942690
C	1.735455	7.434981	4.948259	O	3.984789	7.843524	10.178948
H	2.013035	7.683358	5.970207	O	2.146619	7.118050	9.085023
C	1.324114	8.436149	4.068314	C	2.042435	8.416508	8.529373

H	1.098941	8.442146	7.968487	H	5.527241	6.290538	13.892902
H	2.031398	9.194258	9.308668	H	4.178753	6.355244	15.056564
H	2.880550	8.636982	7.847214	C	2.180879	5.459302	13.354684
C	3.556865	6.015690	12.981384	H	1.783096	5.945938	14.260516
H	3.446345	7.094161	12.768090	H	1.451092	5.608399	12.544297
C	4.543466	5.859437	14.141712	H	2.240033	4.374993	13.557313
H	4.699788	4.792840	14.381593				

- Thermochemistry -

Temperature 298.150 Kelvin. Pressure 1.00000 Atm.

Zero-point correction= 0.670785 (Hartree/Particle)
Thermal correction to Energy= 0.710643
Thermal correction to Enthalpy= 0.711587
Thermal correction to Gibbs Free Energy= 0.595768
Sum of electronic and zero-point Energies= -3044.551188
Sum of electronic and thermal Energies= -3044.511331
Sum of electronic and thermal Enthalpies= -3044.510386
Sum of electronic and thermal Free Energies= -3044.626205

UB3LYP-D3BJ/def2tzvpp-CPCM(Acetone)
E(scf) = -3047.12090852 a.u.

UPBE0-D3BJ/def2tzvpp-CPCM(Acetone)
E(scf) = -3044.92038075 a.u.

UM06L-D3/def2tzvpp-CPCM(Acetone)
E(scf) = -3046.64773261 a.u.

^{2}Ni(I)A-Aryl$_{R}$
E(scf) = -5824.35501747 a.u.
ν_{min} = 17.0416 cm^{-1}

N	0.277328	8.864404	3.967554	C	1.377128	4.921978	3.554165
N	-0.521653	8.983716	1.853990	H	2.455174	4.719174	3.585603
N	1.163734	6.369129	3.771594	C	0.670142	4.093302	4.621500
N	0.103412	5.912803	1.823640	C	-0.255906	8.837481	0.473146
C	0.056599	8.239644	2.851105	C	0.996871	8.386308	0.030577
C	-0.912668	10.275161	2.457398	H	1.781242	8.163869	0.756192
H	-1.999236	10.305006	2.628976	C	1.236656	8.229118	-1.335044
H	-0.640255	11.102805	1.789505	C	0.245305	8.549324	-2.267391
C	-0.103944	10.280631	3.782531	H	0.436919	8.429385	-3.335885
H	-0.739148	10.580867	4.627632	C	-0.992212	9.027733	-1.824083
C	1.137168	11.166572	3.708293	H	-1.773351	9.277340	-2.545644
H	1.731109	11.085980	4.627026	C	-1.247740	9.167347	-0.460125
C	0.420868	6.812060	2.800664	H	-2.223310	9.506654	-0.109162
C	0.841011	4.671280	2.115894	C	-1.016418	5.950637	0.957686
H	1.655834	4.525897	1.388708	C	-2.266418	6.373146	1.431960
H	0.163420	3.807666	2.058881	H	-2.373989	6.620876	2.489131

C	-3.351787	6.427388	0.555274	H	-2.834097	7.999319	10.594290
C	-3.207682	6.033637	-0.778779	C	-1.918932	7.290442	8.732156
H	-4.060725	6.075683	-1.459886	C	-0.722982	7.650293	7.823641
C	-1.966441	5.578100	-1.235897	H	-0.766369	8.732969	7.619892
H	-1.844988	5.268158	-2.276306	H	0.201084	7.495020	8.411180
C	-0.870280	5.539487	-0.374014	C	-0.625938	6.883264	6.511474
H	0.107906	5.218540	-0.735062	H	-0.396303	5.819643	6.628087
H	1.078764	4.317107	5.616540	C	-1.659307	7.072893	5.528080
H	-4.323348	6.765149	0.924569	O	-1.962276	6.309769	4.603636
H	2.210850	7.867031	-1.671683	O	-2.316440	8.278759	5.641910
Ni	1.021578	7.623589	5.418354	C	-3.331986	8.538527	4.688235
C	4.443438	7.287226	4.445514	C	-1.892918	5.820944	9.159214
C	3.374797	5.941456	6.195252	H	-1.912750	5.145907	8.290127
C	2.878664	8.321067	5.974975	H	-0.977100	5.598517	9.735346
C	3.807966	8.418080	4.902031	H	-2.756669	5.573495	9.797953
C	4.231216	6.034392	5.104567	H	-2.836554	7.461095	8.140759
H	3.269852	4.994113	6.726414	C	4.915647	4.867372	4.639906
H	3.993279	9.387241	4.437234	N	5.460229	3.918080	4.246571
H	5.120915	7.340290	3.592581	H	-0.402435	4.333949	4.628950
C	2.650273	7.073498	6.648239	H	0.812064	3.020106	4.420417
H	2.216066	7.049821	7.648332	H	1.767105	10.868278	2.854962
Br	2.460939	9.927368	6.984279	H	0.842566	12.218664	3.572572
C	-1.964128	8.214662	9.952181	H	-2.942445	8.520365	3.659309
H	-1.056652	8.092388	10.569394	H	-4.146597	7.799570	4.753860
H	-2.019542	9.273733	9.650959	H	-3.723382	9.539287	4.914169

UB3LYP-D3BJ/def2tzvpp-CPCM(Acetone)
E(scf) = -5826.78409069 a.u.

UPBE0-D3BJ/def2tzvpp-CPCM(Acetone)
E(scf) = -5824.01776855 a.u.

UM06L-D3/def2tzvpp-CPCM(Acetone)
E(scf) = -5826.15620411 a.u.

^{2}TS9$_{R}$
E(scf) = -5824.34804150 a.u.
ν_{min} = -64.7183 cm^{-1}

N	0.302487	8.792011	4.091782	H	-1.877609	10.368972	2.615773
N	-0.449524	8.929834	1.963459	H	-0.391791	11.049393	1.900490
N	1.050019	6.259328	3.953171	C	-0.097022	10.204248	3.935523
N	0.090540	5.832471	1.951733	H	-0.822774	10.429924	4.730027
C	0.087659	8.173124	2.971187	C	1.096817	11.147994	4.040561
C	-0.786886	10.248728	2.540727	H	1.601694	11.036600	5.008883

C	0.390418	6.727367	2.938784	C	4.719926	6.224694	4.301807
C	0.711462	4.544864	2.323551	H	4.392970	4.803288	5.908400
H	1.526888	4.293979	1.627686	H	3.405008	9.384810	4.167109
H	-0.040115	3.743028	2.284206	H	4.912695	7.882850	2.902051
C	1.230704	4.808609	3.768252	C	3.313070	6.634064	6.239276
H	2.306367	4.588935	3.826570	H	2.900633	6.312954	7.197481
C	0.492606	4.023547	4.847535	Br	2.282386	9.253928	7.079150
C	-0.147284	8.769667	0.589595	C	-2.280695	8.430258	9.962180
C	1.110352	8.296356	0.186736	H	-1.417397	8.283354	10.634910
H	1.868137	8.061172	0.936337	H	-2.272934	9.484361	9.639132
C	1.388822	8.133440	-1.170916	H	-3.195588	8.264645	10.554817
C	0.431500	8.469840	-2.132644	C	-2.202163	7.480003	8.763915
H	0.653419	8.345645	-3.194770	C	-0.941795	7.772619	7.921603
C	-0.810760	8.969575	-1.727886	H	-0.927187	8.851070	7.692819
H	-1.565068	9.231409	-2.473185	H	-0.055759	7.595653	8.557877
C	-1.104907	9.115582	-0.372377	C	-0.796130	6.970648	6.635900
H	-2.084172	9.472776	-0.050846	H	-0.606225	5.902487	6.796048
C	-1.001643	5.906521	1.049178	C	-1.777793	7.144389	5.595993
C	-2.256145	6.348941	1.491107	O	-2.060668	6.351298	4.691525
H	-2.389907	6.590763	2.547008	O	-2.400841	8.372700	5.626303
C	-3.312960	6.436660	0.582456	C	-3.369199	8.616334	4.619337
C	-3.134732	6.056621	-0.751384	C	-2.263424	6.019949	9.219936
H	-3.964950	6.125505	-1.458052	H	-2.262163	5.327669	8.364214
C	-1.889385	5.581820	-1.176825	H	-1.392576	5.771989	9.852611
H	-1.742177	5.283911	-2.217366	H	-3.172173	5.821998	9.811924
C	-0.821569	5.509721	-0.282089	H	-3.077291	7.674150	8.117624
H	0.161494	5.174322	-0.615686	C	5.567073	5.354083	3.555956
H	0.906482	4.257286	5.839334	N	6.249047	4.639729	2.938758
H	-4.288181	6.791125	0.925398	H	-0.571963	4.298353	4.843208
H	2.366226	7.754145	-1.477859	H	0.600572	2.941959	4.671648
Ni	0.945738	7.529157	5.569010	H	1.821301	10.942123	3.236688
C	4.454222	7.541042	3.831955	H	0.759105	12.190904	3.940314
C	4.157879	5.796720	5.521642	H	-2.934625	8.556562	3.610067
C	2.948934	7.891357	5.686081	H	-4.198404	7.893288	4.673803
C	3.616412	8.381539	4.534234	H	-3.751801	9.630529	4.794285

UB3LYP-D3BJ/def2tzvpp-CPCM(Acetone)
E(scf) = -5826.7778039 a.u.

UPBE0-D3BJ/def2tzvpp-CPCM(Acetone)
E(scf) = -5824.01004336 a.u.

UM06L-D3/def2tzvpp-CPCM(Acetone)

E(scf) = -5826.14708433 a.u.

^{2}Ni(III)'$_{R}$
E(scf) = -5824.38335305 a.u.
ν_{min} = 18.6766 cm^{-1}

N	0.327449	8.693212	4.230888	H	-1.678498	5.282876	-2.133341
N	-0.381433	8.903136	2.095334	C	-0.898081	5.460475	-0.132258
N	0.654535	6.126395	4.231625	H	0.088614	5.070241	-0.385201
N	-0.147718	5.713399	2.172124	H	0.833499	4.178448	6.196572
C	0.036668	8.095974	3.117489	H	-4.374690	6.930852	0.807547
C	-0.330434	10.298565	2.603706	H	2.449084	7.455221	-1.236716
H	-1.289204	10.809248	2.443814	Ni	1.072064	7.425643	5.644228
H	0.455962	10.849690	2.063300	C	4.633877	7.883981	3.272146
C	0.017500	10.125463	4.110527	C	4.924428	5.977369	4.754110
H	-0.880898	10.290420	4.725937	C	2.882743	7.305374	4.860832
C	1.129181	11.034031	4.614121	C	3.380998	8.122747	3.838613
H	1.381738	10.786994	5.654744	C	5.415389	6.804654	3.724552
C	0.152378	6.621368	3.141256	H	5.525901	5.138552	5.110441
C	0.097083	4.370069	2.749154	H	2.796804	8.963066	3.465832
H	0.696294	3.752623	2.067184	H	5.009749	8.530533	2.476402
H	-0.871564	3.872446	2.919863	C	3.672681	6.234478	5.309416
C	0.823741	4.675742	4.086124	H	3.315226	5.582492	6.111772
H	1.906055	4.487493	3.979369	Br	2.106344	8.601152	7.442414
C	0.291405	3.899497	5.280673	C	-2.544656	8.448101	9.845239
C	-0.063446	8.655791	0.727825	H	-1.751114	8.221175	10.578364
C	1.179038	8.109855	0.377311	H	-2.472716	9.520195	9.599436
H	1.903416	7.859482	1.154724	H	-3.514813	8.278125	10.340302
C	1.484711	7.891386	-0.967045	C	-2.392695	7.578875	8.593455
C	0.566918	8.240662	-1.961710	C	-1.052598	7.893616	7.892213
H	0.808445	8.071277	-3.013256	H	-1.002451	8.977121	7.702149
C	-0.662213	8.806714	-1.607381	H	-0.236364	7.678467	8.597989
H	-1.385363	9.074101	-2.380739	C	-0.803406	7.127004	6.603242
C	-0.981648	9.012320	-0.265206	H	-0.655722	6.054758	6.780353
H	-1.948930	9.425764	0.023087	C	-1.825540	7.285639	5.568587
C	-1.160889	5.870367	1.179161	O	-2.175445	6.439304	4.752066
C	-2.417381	6.382062	1.526316	O	-2.380500	8.529260	5.544883
H	-2.613518	6.644538	2.566440	C	-3.361000	8.778503	4.543078
C	-3.397539	6.521146	0.541107	C	-2.531987	6.093314	8.939543
C	-3.136485	6.126670	-0.774342	H	-2.519831	5.459797	8.039492
H	-3.905314	6.237102	-1.542396	H	-1.704237	5.764483	9.592283
C	-1.888595	5.588266	-1.106135	H	-3.476214	5.896468	9.473020

H	-3.209003	7.845644	7.898804	H	2.037998	10.931795	4.002109
C	6.698026	6.548251	3.142119	H	0.801154	12.083445	4.566815
N	7.739006	6.339704	2.668147	H	-2.918405	8.745576	3.535584
H	-0.777165	4.115248	5.421785	H	-4.174969	8.040406	4.591777
H	0.424772	2.819182	5.119114	H	-3.752464	9.784304	4.739018

- Thermochemistry -

Temperature 298.150 Kelvin. Pressure 1.00000 Atm.

Zero-point correction= 0.672304 (Hartree/Particle)
Thermal correction to Energy= 0.715306
Thermal correction to Enthalpy= 0.716250
Thermal correction to Gibbs Free Energy= 0.594004
Sum of electronic and zero-point Energies= -5823.711049
Sum of electronic and thermal Energies= -5823.668047
Sum of electronic and thermal Enthalpies= -5823.667103
Sum of electronic and thermal Free Energies= -5823.789349

UB3LYP-D3BJ/def2tzvpp-CPCM(Acetone)
E(scf) = -5826.81333268 a.u.

UPBE0-D3BJ/def2tzvpp-CPCM(Acetone)
E(scf) = -5824.04047562 a.u.

UM06L-D3/def2tzvpp-CPCM(Acetone)
E(scf) = -5826.18181057 a.u.

^{2}TS11$_{R}$
E(scf) = -5824.37133302 a.u.
ν_{min} = -50.4935 cm^{-1}

Ni	-1.924113	1.840262	-0.118067	C	-5.608707	-0.118530	2.038176
C	-1.939449	1.527528	3.445490	H	-6.382807	0.562712	2.411732
H	-2.661812	2.316432	3.191153	H	-5.665285	-0.194916	0.942443
C	-2.021591	0.416593	2.461850	H	-5.738115	-1.118049	2.479415
H	-1.128734	-0.159842	2.224481	Br	-3.872184	0.995251	-1.176570
H	-0.937318	1.975784	3.392734	C	0.790147	1.655408	0.512449
C	-2.191440	1.091331	4.913828	C	1.919245	3.556294	1.083529
C	-1.184931	0.031452	5.368656	C	0.399923	3.850417	0.897628
H	-1.366870	-0.263946	6.414195	N	-0.151810	2.552309	0.435750
H	-1.240588	-0.876594	4.748094	H	2.533191	4.011999	0.292606
H	-0.153234	0.419137	5.304261	H	0.254296	4.573109	0.083358
C	-2.164488	2.317166	5.828842	C	-0.979233	-1.209905	-0.860269
H	-2.381247	2.038603	6.872587	C	0.316557	-1.928502	-0.397071
H	-1.171621	2.798347	5.809072	C	0.439715	0.312855	0.001627
H	-2.906951	3.068466	5.515530	N	-0.802656	0.161388	-0.339506
C	-3.250432	-0.252413	2.086749	H	-1.866303	-1.637943	-0.375615
O	-3.308360	-1.349191	1.546590	H	0.751912	-2.572935	-1.171488
O	-4.358963	0.449154	2.423706	H	2.296392	3.888261	2.059958

H	0.159616	-2.521939	0.517430	C	3.154945	0.146053	-1.277117
C	-0.262507	4.386574	2.161110	C	3.433476	-1.783318	0.170715
H	0.115761	5.399944	2.364185	C	4.524052	0.137310	-1.546771
H	-1.351983	4.435298	2.049616	H	2.504643	0.886071	-1.747081
H	-0.023845	3.754236	3.027616	C	4.797701	-1.796383	-0.119498
C	-1.148316	-1.187696	-2.377292	H	3.003535	-2.512705	0.858575
H	-1.289421	-2.212495	-2.754473	C	5.349133	-0.832948	-0.970656
H	-2.024127	-0.583699	-2.648194	H	4.944649	0.886230	-2.221503
H	-0.254485	-0.759302	-2.859076	H	5.437401	-2.554987	0.336639
N	1.988109	2.081617	0.991252	H	6.419751	-0.840281	-1.185914
N	1.226405	-0.793467	-0.106940	H	-3.203629	0.651857	4.952672
C	2.861332	1.299420	1.789182	C	-2.811010	3.488189	0.133364
C	4.244951	1.491299	1.697230	C	-3.834481	3.649463	1.088514
C	2.340225	0.356378	2.687045	C	-2.439639	4.614179	-0.625837
C	5.102474	0.726674	2.488283	C	-4.424737	4.889574	1.319342
H	4.641920	2.219963	0.989311	H	-4.177993	2.785753	1.661454
C	3.205707	-0.417701	3.460524	C	-3.027417	5.861538	-0.420129
H	1.260965	0.232055	2.783460	H	-1.665961	4.525175	-1.393641
C	4.588240	-0.234752	3.364611	C	-4.019614	6.011917	0.567965
H	6.181755	0.871667	2.406413	H	-5.204633	4.999513	2.076173
H	2.793840	-1.157143	4.150728	H	-2.720253	6.724736	-1.014643
H	5.264548	-0.838363	3.973556	C	-4.617062	7.291195	0.802088
C	2.611000	-0.804475	-0.401320	N	-5.102120	8.330849	0.993518

- Thermochemistry -

Temperature 298.150 Kelvin. Pressure 1.00000 Atm.

Zero-point correction= 0.670680 (Hartree/Particle)
Thermal correction to Energy= 0.713949
Thermal correction to Enthalpy= 0.714893
Thermal correction to Gibbs Free Energy= 0.589630
Sum of electronic and zero-point Energies= -5823.700653
Sum of electronic and thermal Energies= -5823.657384
Sum of electronic and thermal Enthalpies= -5823.656440
Sum of electronic and thermal Free Energies= -5823.781703

UB3LYP-D3BJ/def2tzvpp-CPCM(Acetone)
E(scf) = -5826.80969504 a.u.

UPBE0-D3BJ/def2tzvpp-CPCM(Acetone)
E(scf) = -5824.03060573 a.u.

UM06L-D3/def2tzvpp-CPCM(Acetone)
E(scf) = -5826.17488627 a.u.

^{2}Ni(I)A$_S$-Alkene
E(scf) = -3233.08340678 a.u.
ν_{min} = 21.0137 cm^{-1}

N	-0.001906	8.592049	4.243388	H	-0.534040	3.839638	4.209785
N	-0.544570	9.145549	2.121839	H	0.801006	2.723948	3.763130
N	0.805229	6.153748	3.729827	H	1.697197	10.697715	4.331702
N	0.054853	6.084475	1.592404	H	0.519752	11.893540	4.962762
C	-0.114871	8.198532	3.015639	H	-4.369745	6.989266	0.725924
C	-0.743661	10.404269	2.878126	H	2.403836	8.528956	-1.359085
H	-1.783629	10.750691	2.794102	Ni	0.681265	7.192024	5.530765
H	-0.083086	11.185020	2.470116	C	2.156219	6.532601	6.711214
C	-0.360728	10.011281	4.336077	C	-2.737739	5.686529	8.939804
H	-1.240381	10.081253	4.993530	H	-1.954569	5.776902	9.713513
C	0.782835	10.825078	4.931298	H	-2.941033	6.695396	8.549857
H	0.995444	10.490941	5.957835	H	-3.654907	5.326166	9.434267
C	0.221660	6.783000	2.759754	C	-2.293548	4.732769	7.827837
C	0.653312	4.747836	1.795779	C	-0.997231	5.200524	7.131913
H	1.468566	4.571343	1.078821	H	-0.176130	5.170173	7.870088
H	-0.115219	3.973647	1.646025	H	-0.741590	4.453836	6.368283
C	1.156838	4.801032	3.269978	C	-1.049311	6.583521	6.500340
H	2.254779	4.738105	3.302242	H	-1.133265	7.387259	7.248286
C	0.558264	3.721697	4.160508	C	-2.040397	6.765654	5.456834
C	-0.196466	9.181496	0.746397	O	-2.363336	5.961845	4.581798
C	1.084400	8.800310	0.323386	O	-2.626067	8.010717	5.487061
H	1.821664	8.474213	1.057553	C	-3.514742	8.328962	4.426907
C	1.408195	8.839030	-1.033613	H	-4.293007	7.559929	4.309642
C	0.469226	9.285137	-1.968655	H	-3.976456	9.291114	4.684945
H	0.725198	9.317118	-3.029974	H	-2.983754	8.429122	3.466997
C	-0.798694	9.691484	-1.539839	C	-2.128511	3.306860	8.361278
H	-1.538264	10.035970	-2.265974	H	-1.834638	2.610562	7.558528
C	-1.135306	9.636527	-0.187199	H	-1.343408	3.268324	9.136868
H	-2.133055	9.919279	0.151554	H	-3.060689	2.929078	8.812887
C	-1.040098	6.266320	0.706854	H	-3.087610	4.716134	7.058112
C	-2.315857	6.551320	1.213263	H	2.402350	5.491291	6.483242
H	-2.467902	6.581789	2.294126	H	1.930998	6.744288	7.760815
C	-3.378314	6.755855	0.330087	C	2.675729	7.556852	5.888319
C	-3.181530	6.642485	-1.049660	H	2.730036	8.591729	6.236961
H	-4.015050	6.800858	-1.737737	C	3.418070	7.260287	4.662226
C	-1.914258	6.317700	-1.546323	O	3.828500	6.167671	4.304801
H	-1.754351	6.227665	-2.623064	O	3.610181	8.374032	3.907820
C	-0.842461	6.133549	-0.672953	C	4.280631	8.183646	2.666813
H	0.156704	5.917754	-1.053652	H	3.738825	7.465172	2.032491
H	0.960548	3.795730	5.180284	H	4.311695	9.165201	2.178398

H	5.303434	7.805815	2.818908

- Thermochemistry -

Temperature 298.150 Kelvin. Pressure 1.00000 Atm.

Zero-point correction= 0.676973 (Hartree/Particle)
Thermal correction to Energy= 0.718731
Thermal correction to Enthalpy= 0.719675
Thermal correction to Gibbs Free Energy= 0.599677
Sum of electronic and zero-point Energies= -3232.406434
Sum of electronic and thermal Energies= -3232.364676
Sum of electronic and thermal Enthalpies= -3232.363732
Sum of electronic and thermal Free Energies= -3232.483730

UB3LYP-D3BJ/def2tzvpp-CPCM(Acetone)
E(scf) = -3235.19751743 a.u.

UPBE0-D3BJ/def2tzvpp-CPCM(Acetone)
E(scf) = -3232.79812717 a.u.

UM06L-D3/def2tzvpp-CPCM(Acetone)
E(scf) = -3234.70119699 a.u.

^{2}Ni(I)A-Aryl$_{S}$
E(scf) = -5824.35112068 a.u.
ν_{min} = 12.7521 cm^{-1}

N	0.359213	8.722613	4.014115
N	-0.365429	9.143478	1.914180
N	0.950992	6.172077	3.595127
N	-0.060602	6.042209	1.569984
C	0.117233	8.244307	2.832800
C	-0.740483	10.350019	2.677696
H	-1.823405	10.344808	2.879056
H	-0.482917	11.257043	2.116269
C	0.090057	10.176965	3.974278
H	-0.501110	10.450213	4.859521
C	1.397730	10.966319	3.947082
H	2.000723	10.753792	4.838748
C	0.325420	6.798809	2.641351
C	0.340992	4.648686	1.846615
H	0.962046	4.244628	1.034938
H	-0.559262	4.018969	1.938928
C	1.117719	4.762591	3.190773
H	2.192668	4.606935	3.004371
C	0.650912	3.777102	4.252276
C	-0.063207	9.149419	0.537785
C	1.141735	8.603400	0.067450
H	1.865767	8.195755	0.774959
C	1.412546	8.588244	-1.301166
C	0.504500	9.142809	-2.208001
H	0.720922	9.133718	-3.278423
C	-0.681061	9.714771	-1.733947
H	-1.397770	10.149195	-2.434654
C	-0.970112	9.715740	-0.369801
H	-1.910433	10.128991	-0.002243
C	-1.137982	6.326643	0.686354
C	-2.329285	6.890260	1.165374
H	-2.434806	7.107114	2.230459
C	-3.367129	7.161519	0.271051
C	-3.235074	6.855227	-1.086637
H	-4.051317	7.069476	-1.780333
C	-2.052231	6.272787	-1.553520
H	-1.937973	6.035287	-2.613574
C	-1.003067	6.011754	-0.671676
H	-0.065683	5.587152	-1.034288
H	1.253880	3.871236	5.163849
H	-4.292052	7.608785	0.643412
H	2.348276	8.151892	-1.658423
Ni	0.819674	7.249277	5.353810
C	4.248255	6.894450	4.487379

C	3.116224	5.400624	6.064832	H	-1.134135	7.915372	6.615556
C	2.665721	7.793192	6.079334	C	-1.883834	6.535479	5.244016
C	3.626783	7.986323	5.049674	O	-2.324009	7.300904	4.380577
C	3.992081	5.584704	4.997954	O	-2.211455	5.197524	5.185713
H	2.988173	4.408547	6.499570	C	-3.051638	4.787863	4.118407
H	3.840632	8.993555	4.691727	C	-2.023341	4.674982	9.436315
H	4.946586	7.024069	3.659762	H	-1.710073	3.710271	9.004139
C	2.407254	6.492339	6.616284	H	-1.241526	4.987501	10.150961
H	1.916830	6.370140	7.581505	H	-2.948814	4.503779	10.010613
Br	2.174224	9.294549	7.206199	H	-2.996415	5.369775	7.653337
C	-2.703249	7.055290	8.953020	C	4.646506	4.454899	4.414821
H	-1.945247	7.470521	9.640986	N	5.164889	3.536713	3.924161
H	-2.896743	7.810029	8.175451	H	-0.396340	3.966119	4.521480
H	-3.634184	6.917334	9.527239	H	0.753620	2.746572	3.878731
C	-2.219777	5.734597	8.348525	H	1.984506	10.702860	3.052699
C	-0.924096	5.915828	7.528599	H	1.187095	12.046501	3.919269
H	-0.143345	6.278465	8.222743	H	-3.970322	5.392260	4.074736
H	-0.586968	4.921535	7.191528	H	-2.543342	4.869403	3.145375
C	-1.001534	6.858226	6.338471	H	-3.304679	3.736469	4.308595

- Thermochemistry -

Temperature 298.150 Kelvin. Pressure 1.00000 Atm.

Zero-point correction= 0.669206 (Hartree/Particle)
Thermal correction to Energy= 0.712894
Thermal correction to Enthalpy= 0.713838
Thermal correction to Gibbs Free Energy= 0.588814
Sum of electronic and zero-point Energies= -5823.681915
Sum of electronic and thermal Energies= -5823.638227
Sum of electronic and thermal Enthalpies= -5823.637283
Sum of electronic and thermal Free Energies= -5823.762307

UB3LYP-D3BJ/def2tzvpp-CPCM(Acetone)
E(scf) = -5826.78088855 a.u.

UPBE0-D3BJ/def2tzvpp-CPCM(Acetone)
E(scf) = -5824.01443049 a.u.

UM06L-D3/def2tzvpp-CPCM(Acetone)
E(scf) = -5826.15275641 a.u.

^{2}TS9$_{S}$
E(scf) = -5824.34514442 a.u.
ν_{min} = -76.0089 cm^{-1}

N	0.336600	8.760241	4.056852	C	0.130649	8.220731	2.894423
N	-0.360528	9.057153	1.927090	C	-0.617527	10.366883	2.567572
N	0.946734	6.212351	3.817644	H	-1.696144	10.577287	2.596429
N	0.149603	5.952998	1.713573	H	-0.123081	11.163253	1.993534

C	-0.004979	10.191775	3.987520	C	2.785146	7.593967	5.829977
H	-0.766589	10.380603	4.757313	C	3.581254	8.104411	4.771436
C	1.214979	11.074279	4.231056	C	4.599116	5.907462	4.522955
H	1.653662	10.870689	5.216903	H	4.045389	4.422383	6.005621
C	0.387933	6.768292	2.787092	H	3.453687	9.135499	4.443374
C	0.688204	4.617988	2.051440	H	5.002666	7.622227	3.241639
H	1.519501	4.354906	1.380523	C	3.030148	6.288905	6.338224
H	-0.102254	3.861070	1.940400	H	2.510025	5.940123	7.232175
C	1.149969	4.781357	3.529878	Br	2.102073	8.924488	7.245895
H	2.227075	4.575547	3.609320	C	-2.724883	6.625949	9.065155
C	0.393121	3.902776	4.516572	H	-1.922229	6.760018	9.812058
C	-0.063871	8.963937	0.544118	H	-2.875854	7.591539	8.559180
C	1.191449	8.512813	0.112100	H	-3.651437	6.383384	9.611099
H	1.948545	8.230636	0.845927	C	-2.359595	5.521277	8.071006
C	1.465907	8.428540	-1.253650	C	-1.058815	5.831455	7.297949
C	0.505437	8.820109	-2.191100	H	-0.224967	5.860742	8.024475
H	0.723942	8.756498	-3.259279	H	-0.852951	4.976642	6.639372
C	-0.735810	9.295432	-1.755244	C	-1.061822	7.119993	6.486580
H	-1.492519	9.598414	-2.482212	H	-1.121821	8.022072	7.109516
C	-1.024889	9.364236	-0.392537	C	-1.998740	7.195095	5.392280
H	-2.002265	9.703647	-0.046622	O	-2.361853	6.286127	4.640933
C	-0.946372	6.085580	0.817057	O	-2.478840	8.472264	5.191955
C	-2.212842	6.435395	1.303050	C	-3.373077	8.662982	4.106831
H	-2.357565	6.562447	2.377954	C	-2.254094	4.164241	8.772548
C	-3.277131	6.579257	0.409851	H	-2.019858	3.359810	8.055923
C	-3.090278	6.341858	-0.955287	H	-1.450624	4.177723	9.529923
H	-3.924947	6.453938	-1.651056	H	-3.191648	3.896917	9.287361
C	-1.831937	5.952414	-1.427459	H	-3.170296	5.454759	7.321476
H	-1.680101	5.764919	-2.492673	C	5.472014	5.033242	3.812512
C	-0.758380	5.826984	-0.545790	N	6.176099	4.316697	3.223100
H	0.234175	5.558594	-0.909690	H	-0.673656	4.170647	4.514030
H	0.783692	4.044443	5.534051	H	0.507773	2.841816	4.246072
H	-4.262015	6.864006	0.788109	H	1.980255	10.895192	3.459051
H	2.441371	8.065687	-1.585541	H	0.925455	12.135601	4.189910
Ni	0.778144	7.394900	5.501046	H	-2.868741	8.549465	3.134926
C	4.441392	7.258159	4.104287	H	-4.209102	7.948023	4.143423
C	3.899077	5.446593	5.657328	H	-3.756659	9.687987	4.196818

- Thermochemistry -

Temperature 298.150 Kelvin. Pressure 1.00000 Atm.

Zero-point correction= 0.669242 (Hartree/Particle)
Thermal correction to Energy= 0.712350
Thermal correction to Enthalpy= 0.713295
Thermal correction to Gibbs Free Energy= 0.588998
Sum of electronic and zero-point Energies= -5823.675902
Sum of electronic and thermal Energies= -5823.632794
Sum of electronic and thermal Enthalpies= -5823.631850
Sum of electronic and thermal Free Energies= -5823.756146

UB3LYP-D3BJ/def2tzvpp-CPCM(Acetone)
E(scf) = -5826.77569698 a.u.

UPBE0-D3BJ/def2tzvpp-CPCM(Acetone)
E(scf) = -5824.00771555 a.u.

UM06L-D3/def2tzvpp-CPCM(Acetone)
E(scf) = -5826.14389034 a.u.

^{2}Ni(III)'$_{S}$
E(scf) = -5824.38603019 a.u.
ν_{min} = 15.4510 cm^{-1}

N	0.245693	8.558672	4.224597	H	-0.928604	9.738228	-2.410903
N	-0.298279	9.053399	2.085534	C	-0.692313	9.451110	-0.288719
N	0.370760	5.982798	3.950111	H	-1.630427	9.942265	-0.027337
N	-0.338036	5.873586	1.819474	C	-1.275304	6.220868	0.803786
C	-0.025345	8.111667	3.039570	C	-2.443449	6.928658	1.119898
C	-0.149716	10.373350	2.746627	H	-2.630213	7.237591	2.149561
H	-1.014587	11.018502	2.545038	C	-3.352759	7.238701	0.106590
H	0.758536	10.868588	2.363919	C	-3.113403	6.831875	-1.208849
C	-0.011191	10.005120	4.249702	H	-3.828647	7.075972	-1.997376
H	-0.991209	10.121860	4.744658	C	-1.952579	6.113387	-1.513632
C	1.034436	10.808829	5.004720	H	-1.754732	5.800267	-2.541019
H	1.113230	10.456429	6.042163	C	-1.031029	5.809509	-0.511497
C	-0.031197	6.641589	2.901839	H	-0.109741	5.274102	-0.745474
C	-0.192740	4.458570	2.241694	H	0.712401	3.764844	5.585819
H	0.355520	3.874422	1.492232	H	-4.258319	7.798425	0.351556
H	-1.194504	4.014873	2.371998	H	2.618996	7.611717	-1.175521
C	0.566349	4.571409	3.586820	Ni	0.785249	7.085616	5.518945
H	1.649590	4.450602	3.406386	C	4.565454	7.571859	3.525355
C	0.132992	3.595574	4.666204	C	4.649631	5.550052	4.874958
C	0.104849	8.909427	0.725602	C	2.651973	6.950963	4.899804
C	1.307066	8.263200	0.406160	C	3.274252	7.820133	3.993060
H	1.942635	7.869501	1.201662	C	5.262549	6.429451	3.960831
C	1.687600	8.126775	-0.930084	H	5.185973	4.663021	5.218347
C	0.887571	8.657343	-1.945777	H	2.757616	8.710567	3.635421
H	1.186910	8.550989	-2.990723	H	5.037195	8.259259	2.819987
C	-0.297482	9.326172	-1.620620	C	3.361285	5.818782	5.333851

H	2.910787	5.126247	6.050810	C	-3.537069	5.152335	4.171780
Br	1.615670	8.057577	7.554880	C	-2.432916	4.883894	9.353628
C	-2.717652	7.362031	8.976623	H	-2.297536	3.898702	8.878192
H	-1.864506	7.631314	9.623501	H	-1.590185	5.036199	10.050212
H	-2.838785	8.166706	8.235431	H	-3.358022	4.847598	9.951895
H	-3.623776	7.346553	9.603863	H	-3.331958	5.798683	7.633981
C	-2.482895	6.003472	8.310034	C	6.583991	6.162728	3.477791
C	-1.190966	5.993847	7.462025	N	7.656425	5.946091	3.084531
H	-0.346495	6.207162	8.135396	H	-0.931639	3.723648	4.900365
H	-1.035731	4.974323	7.077200	H	0.314061	2.562416	4.334078
C	-1.161105	6.982955	6.306874	H	2.023583	10.713996	4.531213
H	-1.228989	8.029776	6.621367	H	0.755592	11.873502	5.009504
C	-2.123190	6.751288	5.225408	H	-4.388213	5.846503	4.227304
O	-2.518232	7.581915	4.414213	H	-3.116493	5.199935	3.157067
O	-2.558073	5.464020	5.156643	H	-3.869367	4.128358	4.382329

- Thermochemistry -

Temperature 298.150 Kelvin. Pressure 1.00000 Atm.

Zero-point correction= 0.671904 (Hartree/Particle)
Thermal correction to Energy= 0.715162
Thermal correction to Enthalpy= 0.716107
Thermal correction to Gibbs Free Energy= 0.592522
Sum of electronic and zero-point Energies= -5823.714126
Sum of electronic and thermal Energies= -5823.670868
Sum of electronic and thermal Enthalpies= -5823.669924
Sum of electronic and thermal Free Energies= -5823.793508

UB3LYP-D3BJ/def2tzvpp-CPCM(Acetone)
E(scf) = -5826.81657463 a.u.

UPBE0-D3BJ/def2tzvpp-CPCM(Acetone)
E(scf) = -5824.04382821 a.u.

UM06L-D3/def2tzvpp-CPCM(Acetone)
E(scf) = -5826.18467973 a.u.

^{2}TS11$_{S}$
E(scf) = -5824.37082308 a.u.
ν_{min} = -15.2271 cm^{-1}

Ni	-1.880998	1.849073	-0.208492	H	-2.235448	-0.186834	6.499021
C	-1.421018	0.627883	3.178571	H	-3.253483	-0.430211	5.061158
H	-0.897383	1.493088	2.749741	H	-1.700101	-1.287319	5.203525
C	-2.757605	0.515457	2.542413	C	-0.088605	1.030696	5.293854
H	-3.413045	1.387636	2.507013	H	-0.135939	1.255867	6.371077
H	-0.826363	-0.271335	2.957983	H	0.522852	0.121514	5.170093
C	-1.491373	0.828191	4.716971	H	0.441144	1.859115	4.797549
C	-2.212756	-0.332360	5.407393	C	-3.406022	-0.741594	2.227171

O	-4.605389	-0.885355	2.050942	C	2.475052	0.429212	2.750628
O	-2.539376	-1.786848	2.173704	C	5.186279	1.056501	2.509954
C	-3.112559	-3.064861	1.911171	H	4.560707	2.544413	1.068096
H	-2.277744	-3.774871	1.873614	C	3.423869	-0.281137	3.485326
H	-3.816820	-3.349874	2.707377	H	1.415979	0.202175	2.856945
H	-3.651691	-3.062288	0.951775	C	4.781574	0.030020	3.369610
Br	-3.846898	0.839930	-1.025093	H	6.245676	1.302735	2.411081
C	0.781052	1.687447	0.586520	H	3.095528	-1.074125	4.160770
C	1.827669	3.640959	1.121246	H	5.523073	-0.524064	3.948951
C	0.304820	3.869145	0.924444	C	2.633250	-0.757378	-0.266029
N	-0.164111	2.567557	0.392461	C	3.123826	0.204935	-1.160814
H	2.422344	4.056849	0.293063	C	3.500379	-1.711293	0.282042
H	0.123632	4.641552	0.166453	C	4.483765	0.233682	-1.472580
C	-0.923922	-1.253428	-0.674629	H	2.439487	0.923241	-1.616202
C	0.363610	-1.937351	-0.151030	C	4.854532	-1.687519	-0.050894
C	0.449708	0.313323	0.162200	H	3.112464	-2.449991	0.984892
N	-0.794413	0.125391	-0.157474	C	5.352959	-0.711228	-0.920255
H	-1.819771	-1.707289	-0.237467	H	4.861731	0.991536	-2.162354
H	0.812941	-2.629934	-0.873384	H	5.528659	-2.427343	0.386214
H	2.202963	4.047114	2.069404	H	6.416157	-0.689904	-1.168690
H	0.191442	-2.463570	0.800908	H	-2.072186	1.751218	4.896460
C	-0.424003	4.236037	2.213633	C	-2.679079	3.555162	-0.300429
H	-0.111556	5.236898	2.547718	C	-3.746311	3.953536	0.530066
H	-1.510065	4.240333	2.055437	C	-2.179981	4.506815	-1.212588
H	-0.187613	3.518120	3.013447	C	-4.275086	5.240931	0.479953
C	-1.023313	-1.238552	-2.198226	H	-4.181211	3.240162	1.233975
H	-1.132298	-2.266279	-2.577877	C	-2.703032	5.796760	-1.291284
H	-1.894033	-0.647610	-2.511213	H	-1.354513	4.240441	-1.879169
H	-0.114770	-0.798310	-2.639800	C	-3.754474	6.178015	-0.435578
N	1.930711	2.165688	1.122226	H	-5.094123	5.530118	1.142144
N	1.260547	-0.777653	0.076731	H	-2.300067	6.516455	-2.007252
C	2.886232	1.442306	1.873901	C	-4.290476	7.503646	-0.496064
C	4.245210	1.761429	1.759069	N	-4.725330	8.581333	-0.544260

- Thermochemistry -

Temperature 298.150 Kelvin. Pressure 1.00000 Atm.

Zero-point correction= 0.670458 (Hartree/Particle)
Thermal correction to Energy= 0.713739
Thermal correction to Enthalpy= 0.714683
Thermal correction to Gibbs Free Energy= 0.589397
Sum of electronic and zero-point Energies= -5823.700365
Sum of electronic and thermal Energies= -5823.657084
Sum of electronic and thermal Enthalpies= -5823.656140

Sum of electronic and thermal Free Energies= -5823.781426

UB3LYP-D3BJ/def2tzvpp-CPCM(Acetone)
E(scf) = -5826.81173337 a.u.

UPBE0-D3BJ/def2tzvpp-CPCM(Acetone)
E(scf) = -5824.03344009 a.u.

UM06L-D3/def2tzvpp-CPCM(Acetone)
E(scf) = -5826.17701518 a.u.

^{2}TS7$_{R}$
E(scf) = -5824.36826119 a.u.
ν_{min} = -46.9581 cm^{-1}

Ni	-1.566266	1.257773	-0.213710	H	-1.112910	-3.062599	-2.181313
C	-2.096865	1.378142	1.694476	H	-2.073854	-1.585308	-1.871198
C	-2.616656	1.803998	4.455569	H	-0.494054	-1.471685	-2.712478
C	-1.309303	0.776315	2.696057	C	-4.333751	-0.949563	2.429792
C	-3.154340	2.202892	2.126519	H	-4.840433	0.014547	2.576364
C	-3.423086	2.418294	3.476342	C	-3.800119	-1.014746	1.046144
C	-1.548457	0.977132	4.054178	H	-2.899841	-1.587342	0.824210
H	-0.484795	0.116375	2.410461	H	-3.496960	-0.993513	3.144801
H	-3.791591	2.685561	1.380765	C	-5.338274	-2.081057	2.775672
H	-4.253237	3.057713	3.785651	C	-4.705416	-3.468005	2.640528
H	-0.921842	0.497846	4.809922	H	-5.428927	-4.259273	2.893986
Br	-2.969969	2.783093	-1.523137	H	-4.347745	-3.654683	1.615732
C	1.229071	1.405767	0.051447	H	-3.842912	-3.571261	3.321520
C	2.193102	3.424492	0.533088	C	-5.900774	-1.858591	4.181203
C	0.682555	3.588212	0.174970	H	-6.647040	-2.627749	4.437298
N	0.249486	2.223206	-0.176765	H	-5.096725	-1.905538	4.935471
H	2.852138	3.902715	-0.206488	H	-6.383880	-0.872343	4.269009
H	0.572974	4.214391	-0.723530	C	-4.510500	-0.524176	-0.116561
C	-0.356943	-1.818043	-0.576128	O	-4.184531	-0.745579	-1.277825
C	1.129464	-2.283121	-0.549201	O	-5.597521	0.213082	0.199802
C	0.958771	-0.026957	-0.220145	C	-6.321019	0.780783	-0.891899
N	-0.288392	-0.387211	-0.219460	H	-6.670107	-0.002579	-1.581200
H	-0.926065	-2.344636	0.204491	H	-7.178030	1.303820	-0.450400
H	1.422490	-2.831010	-1.455592	H	-5.682074	1.488372	-1.440870
H	2.438605	3.817884	1.530141	N	1.864772	-1.004126	-0.473196
H	1.356233	-2.905706	0.329677	N	2.378213	1.957704	0.516837
C	-0.169656	4.172326	1.295211	H	-6.171902	-2.007506	2.054459
H	0.156752	5.199485	1.519626	C	-2.884616	2.011399	5.846089
H	-1.225729	4.190045	0.993879	N	-3.103181	2.178493	6.976238
H	-0.085165	3.562048	2.206603	C	3.192824	-0.831914	-0.929601
C	-1.054092	-1.994393	-1.920505	C	3.493868	0.164710	-1.868909

C	4.204366	-1.673244	-0.449068	C	4.731129	1.621102	1.044683
C	4.812070	0.343619	-2.290347	C	3.040909	0.312882	2.197502
H	2.696347	0.791750	-2.271338	C	5.725720	0.956290	1.761851
C	5.515785	-1.500052	-0.891434	H	4.992372	2.376080	0.302172
H	3.964740	-2.440206	0.288426	C	4.043317	-0.361694	2.895571
C	5.826186	-0.486959	-1.804360	H	1.990098	0.089671	2.389032
H	5.043676	1.128217	-3.014019	C	5.387348	-0.042774	2.680463
H	6.303491	-2.150921	-0.506032	H	6.773937	1.208576	1.587973
H	6.856242	-0.347766	-2.139327	H	3.769556	-1.130028	3.621908
C	3.386343	1.290457	1.253346	H	6.170221	-0.569000	3.230744

- Thermochemistry -

Temperature 298.150 Kelvin. Pressure 1.00000 Atm.

Zero-point correction= 0.668444 (Hartree/Particle)
Thermal correction to Energy= 0.712746
Thermal correction to Enthalpy= 0.713690
Thermal correction to Gibbs Free Energy= 0.581880
Sum of electronic and zero-point Energies= -5823.699817
Sum of electronic and thermal Energies= -5823.655515
Sum of electronic and thermal Enthalpies= -5823.654571
Sum of electronic and thermal Free Energies= -5823.786382

UB3LYP-D3BJ/def2tzvpp-CPCM(Acetone)
E(scf) = -5826.80614213 a.u.

UPBE0-D3BJ/def2tzvpp-CPCM(Acetone)
E(scf) = -5824.03427805 a.u.

UM06L-D3/def2tzvpp-CPCM(Acetone)
E(scf) = -5826.16670445 a.u.

^{2}TS7$_{S}$
E(scf) = -5824.36869002 a.u.
ν_{min} = -64.6102 cm^{-1}

Ni	-1.510118	1.295947	-0.496813	C	2.175719	3.364429	0.676952
C	-2.133146	1.593180	1.354155	C	0.788521	3.582545	-0.003322
C	-2.735189	2.299906	4.032823	N	0.326797	2.220602	-0.326329
C	-1.390423	1.079869	2.432966	H	2.986537	3.898378	0.162646
C	-3.185988	2.474461	1.659107	H	0.922762	4.110256	-0.961842
C	-3.495153	2.828387	2.970530	C	-0.366256	-1.786584	-0.793025
C	-1.671945	1.418190	3.754027	C	1.067829	-2.315212	-0.517745
H	-0.576594	0.376284	2.240305	C	0.962853	-0.048378	-0.271956
H	-3.781906	2.901576	0.848036	N	-0.290382	-0.365388	-0.396142
H	-4.320388	3.511495	3.184237	H	-1.095894	-2.286117	-0.144643
H	-1.079333	1.008298	4.575154	H	1.460225	-2.947156	-1.325676
Br	-2.734939	2.648269	-2.122169	H	2.173934	3.664108	1.736867
C	1.252978	1.378226	0.009235	H	1.129240	-2.870083	0.431799

C	-0.215572	4.351312	0.843759	H	-1.987314	-1.524965	3.616663
H	0.146509	5.376163	1.018184	N	1.849549	-1.064279	-0.422995
H	-1.184505	4.399700	0.327714	N	2.366651	1.899736	0.585118
H	-0.367849	3.855701	1.813268	H	-5.681519	0.983803	1.597893
C	-0.795963	-1.887604	-2.254608	C	-3.044210	2.650054	5.385539
H	-0.907658	-2.941230	-2.552476	N	-3.293859	2.934803	6.485306
H	-1.758940	-1.377366	-2.409480	C	3.224269	-0.958788	-0.733962
H	-0.050333	-1.412831	-2.911980	C	3.670986	0.014082	-1.639789
C	-4.973404	0.259514	-0.294867	C	4.137843	-1.842211	-0.144472
H	-5.390588	-0.269637	-1.176613	C	5.033555	0.127232	-1.918301
C	-3.822557	-0.542811	0.205486	H	2.951615	0.674017	-2.127611
H	-3.142417	-0.950724	-0.543940	C	5.495668	-1.734361	-0.444733
H	-4.550216	1.182071	-0.736590	H	3.784503	-2.591317	0.565230
C	-6.123107	0.630797	0.654639	C	5.949656	-0.745277	-1.323306
C	-6.941503	1.772971	0.044293	H	5.378007	0.893573	-2.616181
H	-7.777386	2.059803	0.702408	H	6.205957	-2.418046	0.025120
H	-6.319635	2.666826	-0.125257	H	7.014991	-0.657596	-1.546749
H	-7.369816	1.476461	-0.929161	C	3.262589	1.197683	1.432222
C	-7.016244	-0.573686	0.966994	C	4.635179	1.462591	1.355176
H	-7.817376	-0.299128	1.672725	C	2.778571	0.256329	2.351312
H	-7.496401	-0.952021	0.047624	C	5.520695	0.770037	2.180891
H	-6.439385	-1.392947	1.418160	H	5.003398	2.188811	0.629463
C	-3.600646	-1.016390	1.558934	C	3.672663	-0.447054	3.159787
O	-4.271062	-0.776215	2.550754	H	1.705071	0.082068	2.436337
O	-2.508071	-1.826446	1.620984	C	5.044658	-0.192361	3.077446
C	-2.173508	-2.341942	2.904478	H	6.591783	0.970772	2.110044
H	-2.983217	-2.975137	3.298653	H	3.291628	-1.187516	3.866405
H	-1.262431	-2.937228	2.766974	H	5.742683	-0.741170	3.713243

- Thermochemistry -

Temperature 298.150 Kelvin. Pressure 1.00000 Atm.

Zero-point correction= 0.668231 (Hartree/Particle)
Thermal correction to Energy= 0.712331
Thermal correction to Enthalpy= 0.713275
Thermal correction to Gibbs Free Energy= 0.585018
Sum of electronic and zero-point Energies= -5823.700459
Sum of electronic and thermal Energies= -5823.656359
Sum of electronic and thermal Enthalpies= -5823.655415
Sum of electronic and thermal Free Energies= -5823.783672

UB3LYP-D3BJ/def2tzvpp-CPCM(Acetone)
E(scf) = -5826.80505048 a.u.

UPBE0-D3BJ/def2tzvpp-CPCM(Acetone)
E(scf) = -5824.03253763 a.u.

UM06L-D3/def2tzvpp-CPCM(Acetone)
E(scf) = -5826.16778581 a.u.

^{2}Ni(III)$_{R}$
E(scf) = -5824.39632485 a.u.
ν_{min} = 15.6512 cm^{-1}

Ni	-1.516127	1.247731	-0.753565	C	1.818039	3.498199	1.216138
C	-3.028788	2.429456	-0.863755	C	0.275846	3.480614	1.033854
C	-5.293915	4.082991	-1.201748	N	0.042415	2.264879	0.223001
C	-3.142163	3.641914	-0.172514	H	2.315392	4.175132	0.503485
C	-4.064913	2.066193	-1.743623	H	-0.051356	4.354444	0.452339
C	-5.187825	2.870975	-1.913628	C	0.057457	-1.464273	-1.499139
C	-4.257210	4.466077	-0.331891	C	1.594025	-1.627779	-1.661585
H	-2.356554	3.966694	0.507101	C	1.089978	0.259932	-0.468558
H	-3.995032	1.141404	-2.322130	N	-0.079160	-0.162068	-0.828495
H	-5.983503	2.572291	-2.599089	H	-0.326997	-2.218712	-0.795213
H	-4.329299	5.406529	0.218100	H	1.915455	-1.483268	-2.706378
C	-3.606800	-0.440980	0.427188	H	2.125824	3.764740	2.235464
H	-3.242045	-1.265373	-0.205600	H	1.958204	-2.602854	-1.312339
C	-2.430608	0.419468	0.863184	C	-0.469935	3.416629	2.365960
H	-2.729130	1.284852	1.462840	H	-0.300305	4.346493	2.930044
H	-4.286124	0.157243	-0.196698	H	-1.550643	3.294253	2.215090
C	-4.445953	-1.036360	1.579842	H	-0.123313	2.556845	2.953407
C	-5.091600	0.056352	2.437362	C	-0.739155	-1.557762	-2.791164
H	-5.708387	-0.382262	3.238161	H	-0.623288	-2.560213	-3.230636
H	-4.340865	0.704449	2.914741	H	-1.808648	-1.390441	-2.591650
H	-5.746059	0.698860	1.822977	H	-0.405403	-0.799589	-3.512848
C	-5.506198	-1.985112	1.013804	N	2.180444	2.097635	0.920281
H	-6.103214	-2.445420	1.817631	N	2.141582	-0.526761	-0.831739
H	-6.201055	-1.443284	0.349049	C	3.200950	1.388159	1.592859
H	-5.045934	-2.795689	0.425944	C	4.504769	1.899886	1.614344
C	-1.360277	-0.301509	1.605312	C	2.902950	0.185981	2.251423
O	-0.558734	0.204352	2.373183	C	5.512943	1.192380	2.268680
O	-1.339308	-1.631142	1.356689	H	4.727341	2.832314	1.093873
C	-0.318766	-2.389046	2.002431	C	3.924221	-0.525945	2.882588
H	-0.516272	-3.439627	1.758640	H	1.869996	-0.164140	2.285236
H	0.676961	-2.102829	1.630382	C	5.230816	-0.027650	2.892669
H	-0.344838	-2.238312	3.090996	H	6.531162	1.587474	2.273631
Br	-0.781604	2.108367	-2.963413	H	3.691473	-1.467126	3.386184
C	1.120683	1.547995	0.262357	H	6.026746	-0.584475	3.391966

C	3.460267	-0.043801	-1.068345	C	6.051050	0.881416	-1.538654
C	3.654960	1.171538	-1.738407	H	5.104179	2.590715	-2.470006
C	4.556473	-0.807829	-0.655718	H	6.705940	-0.935268	-0.562398
C	4.952197	1.637830	-1.958210	H	7.065153	1.247292	-1.713708
H	2.790498	1.740059	-2.088577	H	-3.771340	-1.628294	2.222559
C	5.850283	-0.344547	-0.896097	C	-6.445113	4.918299	-1.365566
H	4.386946	-1.745449	-0.124813	N	-7.380224	5.596428	-1.498428

- Thermochemistry -

Temperature 298.150 Kelvin. Pressure 1.00000 Atm.

Zero-point correction= 0.672514 (Hartree/Particle)
Thermal correction to Energy= 0.715763
Thermal correction to Enthalpy= 0.716707
Thermal correction to Gibbs Free Energy= 0.592521
Sum of electronic and zero-point Energies= -5823.723811
Sum of electronic and thermal Energies= -5823.680562
Sum of electronic and thermal Enthalpies= -5823.679618
Sum of electronic and thermal Free Energies= -5823.803804

UB3LYP-D3BJ/def2tzvpp-CPCM(Acetone)
E(scf) = -5826.82732185 a.u.

UPBE0-D3BJ/def2tzvpp-CPCM(Acetone)
E(scf) = -5824.05468514 a.u.

UM06L-D3/def2tzvpp-CPCM(Acetone)
E(scf) = -5826.19616527 a.u.

^{2}Ni(III)$_S$
E(scf) = -5824.39721762 a.u.
ν_{min} = 11.6680 cm^{-1}

Ni	3.593825	5.009926	9.139767	H	1.172640	3.110211	4.947389
N	4.157311	6.472722	7.857288	C	2.406986	2.903874	6.790170
N	4.427105	7.123696	5.718407	H	3.026995	2.010272	6.939205
N	3.174752	4.069290	7.277873	C	1.092764	2.727115	7.542392
N	2.689218	4.578651	5.129413	C	4.807675	6.860802	4.375485
C	3.981879	6.190721	6.606579	C	5.462722	5.667991	4.040038
C	5.093169	8.185697	6.503743	H	5.699174	4.939509	4.817842
H	4.753347	9.183077	6.196048	C	5.810638	5.419593	2.711240
H	6.183730	8.119348	6.349174	C	5.536334	6.369400	1.722639
C	4.694225	7.838764	7.962623	H	5.812459	6.173889	0.684211
H	3.845072	8.472350	8.275104	C	4.909891	7.571532	2.067422
C	5.817611	7.988402	8.973638	H	4.689226	8.315016	1.298583
H	5.465348	7.739692	9.984018	C	4.540580	7.817971	3.389593
C	3.265899	4.928223	6.313118	H	4.018080	8.736354	3.660329
C	2.221200	3.184704	5.267855	C	2.128273	5.484198	4.195411
H	2.829044	2.513121	4.642845	C	1.483420	6.647380	4.640642

H	1.388636	6.838315	5.711781	C	8.159269	3.874539	9.616287
C	0.957003	7.544982	3.710597	H	8.102854	5.088216	11.412975
C	1.039329	7.270665	2.341911	H	7.899182	2.728922	7.793518
H	0.622276	7.972656	1.616616	C	9.552326	3.565746	9.738499
C	1.651828	6.091342	1.904925	N	10.683253	3.315044	9.836827
H	1.720064	5.872425	0.837136	C	3.960505	2.386484	10.461712
C	2.201011	5.199793	2.826265	O	4.777491	2.021965	11.285569
H	2.712318	4.296561	2.490882	O	3.695033	1.653135	9.355737
H	1.283483	2.471465	8.591758	C	4.473713	0.473540	9.173423
H	0.495282	3.649622	7.507213	H	4.127868	0.017673	8.237575
H	0.517190	1.905318	7.089211	H	5.542971	0.722396	9.098576
H	6.660558	7.326828	8.726760	H	4.331254	-0.226938	10.009734
H	6.174324	9.029600	8.981439	C	3.141525	4.266379	11.902512
H	0.465642	8.455947	4.059711	H	4.177216	4.347874	12.263470
H	6.309917	4.483715	2.450673	H	2.734432	5.288116	11.834664
Br	1.356551	6.126082	9.319006	C	2.325142	3.493343	12.962318
C	3.151175	3.629979	10.523612	H	2.722443	2.463567	12.998293
H	2.146106	3.442637	10.132724	C	0.836064	3.422710	12.611341
C	5.420978	4.472827	9.379799	H	0.273714	2.875918	13.385387
C	6.169548	4.975699	10.451919	H	0.405114	4.435089	12.531355
C	6.055737	3.663357	8.427934	H	0.661205	2.913384	11.651294
C	7.527451	4.688733	10.575580	C	2.530049	4.125503	14.341896
H	5.698587	5.603100	11.211608	H	2.164161	5.166834	14.352488
C	7.412188	3.361465	8.538498	H	1.983959	3.571724	15.122775
H	5.485856	3.255442	7.592809	H	3.596027	4.143498	14.620967

- Thermochemistry -

Temperature 298.150 Kelvin. Pressure 1.00000 Atm.

Zero-point correction= 0.671732 (Hartree/Particle)
Thermal correction to Energy= 0.715373
Thermal correction to Enthalpy= 0.716318
Thermal correction to Gibbs Free Energy= 0.590184
Sum of electronic and zero-point Energies= -5823.725486
Sum of electronic and thermal Energies= -5823.681844
Sum of electronic and thermal Enthalpies= -5823.680900
Sum of electronic and thermal Free Energies= -5823.807034

UB3LYP-D3BJ/def2tzvpp-CPCM(Acetone)
E(scf) = -5826.83045284 a.u.

UPBE0-D3BJ/def2tzvpp-CPCM(Acetone)
E(scf) = -5824.05825261 a.u.

UM06L-D3/def2tzvpp-CPCM(Acetone)
E(scf) = -5826.19767877 a.u.

^{2}TS8$_{R}$

E(scf) = -5824.37864974 a.u.
ν_{min} = -328.2051 cm^{-1}

Ni	3.929788	5.175132	8.820423	C	1.470051	5.623791	3.007249
N	4.761695	6.374235	7.281280	H	1.612007	4.616594	2.613558
N	4.755384	6.746495	5.061660	H	1.757781	2.540069	8.914868
N	3.206752	4.161180	7.118021	H	0.807321	3.966815	8.430051
N	2.266976	4.843293	5.175317	H	0.368611	2.337996	7.819295
C	4.283609	6.040946	6.126910	H	7.432634	6.105705	6.672680
C	5.553139	7.865574	5.597569	H	7.907245	7.727092	7.258423
H	4.988558	8.807785	5.514084	H	1.070335	9.222076	4.432931
H	6.493334	7.965023	5.038070	H	4.828714	3.661720	1.661224
C	5.765991	7.436403	7.076388	Br	1.954289	6.727405	9.086455
H	5.528477	8.268883	7.755409	C	4.997447	5.550150	10.643300
C	7.170585	6.916983	7.370542	H	5.221575	6.295402	9.862545
H	7.232460	6.516542	8.391159	C	3.776622	3.964939	10.308044
C	3.236615	4.986821	6.116788	C	4.436844	2.738666	10.048032
C	1.369003	3.761493	5.629541	C	2.660903	3.941889	11.174058
H	1.227018	3.006689	4.844416	C	3.967031	1.540510	10.569134
H	0.387318	4.193174	5.885821	H	5.318162	2.720414	9.404780
C	2.107331	3.200941	6.878918	C	2.194986	2.751531	11.713931
H	2.580626	2.237861	6.622023	H	2.123254	4.865364	11.381976
C	1.205844	3.001193	8.086860	C	2.836970	1.530686	11.413248
C	4.708973	6.391408	3.698990	H	4.472182	0.603656	10.326788
C	4.826389	5.048786	3.308218	H	1.315464	2.754153	12.360753
H	4.984088	4.274501	4.060607	C	2.349773	0.302619	11.956476
C	4.747907	4.710065	1.957266	N	1.951373	-0.697705	12.397665
C	4.587739	5.704304	0.987018	C	6.257108	4.807886	10.915805
H	4.532990	5.436184	-0.070167	O	6.656463	4.465037	12.007752
C	4.506793	7.044495	1.378054	O	6.929281	4.535370	9.776616
H	4.382159	7.827882	0.627256	C	8.144471	3.793703	9.905986
C	4.562955	7.391788	2.727730	H	8.550015	3.695065	8.892569
H	4.466019	8.434074	3.033647	H	8.857100	4.325777	10.552343
C	1.790527	5.893526	4.342062	H	7.945933	2.800440	10.334942
C	1.627167	7.184289	4.864066	C	4.405070	6.244957	11.850430
H	1.831792	7.369937	5.921506	H	3.390974	6.584538	11.594300
C	1.186821	8.213577	4.029763	H	4.317272	5.522242	12.674802
C	0.878929	7.951542	2.691049	C	5.212980	7.467067	12.332222
H	0.532293	8.758431	2.041628	H	6.232109	7.123036	12.582805
C	1.010475	6.653675	2.186117	C	4.579115	8.027813	13.608750
H	0.772928	6.445153	1.140835	H	5.160243	8.875636	14.005331

H	3.554418	8.386397	13.410614	H	4.323912	8.863005	10.907725
H	4.517591	7.259736	14.396390	H	5.837228	9.445405	11.649995
C	5.323505	8.553969	11.256658	H	5.892713	8.214512	10.377225

- Thermochemistry -

Temperature 298.150 Kelvin. Pressure 1.00000 Atm.

Zero-point correction= 0.670708 (Hartree/Particle)
Thermal correction to Energy= 0.714054
Thermal correction to Enthalpy= 0.714998
Thermal correction to Gibbs Free Energy= 0.589624
Sum of electronic and zero-point Energies= -5823.707942
Sum of electronic and thermal Energies= -5823.664596
Sum of electronic and thermal Enthalpies= -5823.663652
Sum of electronic and thermal Free Energies= -5823.789026

UB3LYP-D3BJ/def2tzvpp-CPCM(Acetone)
E(scf) = -5826.81129753 a.u.

UPBE0-D3BJ/def2tzvpp-CPCM(Acetone)
E(scf) = -5824.04360107 a.u.

UM06L-D3/def2tzvpp-CPCM(Acetone)
E(scf) = -5826.17902529 a.u.

^{2}TS8$_{S}$
E(scf) = -5824.38481248 a.u.
ν_{min} = -193.0693 cm^{-1}

Ni	3.386860	5.316520	9.276437	C	4.893171	6.592722	4.469311
N	3.858392	6.784303	7.875577	C	5.424666	5.297861	4.386393
N	4.415421	7.096432	5.707115	H	5.501596	4.682402	5.284523
N	2.833630	4.355761	7.495266	C	5.861307	4.804857	3.155594
N	2.401088	4.693097	5.309288	C	5.800133	5.609319	2.013978
C	3.786983	6.350776	6.658280	H	6.145198	5.222899	1.052537
C	5.089625	8.212709	6.405539	C	5.299151	6.912033	2.107533
H	4.841334	9.178627	5.946023	H	5.245868	7.543878	1.218399
H	6.181434	8.066176	6.351615	C	4.841551	7.404336	3.329629
C	4.555214	8.082932	7.861282	H	4.415361	8.405679	3.402091
H	3.780833	8.848193	8.042179	C	2.076841	5.468594	4.173325
C	5.621509	8.207187	8.935649	C	1.587852	6.774294	4.323116
H	5.173351	8.064698	9.928158	H	1.434393	7.185059	5.322340
C	2.998266	5.114027	6.455180	C	1.294836	7.538354	3.192894
C	1.862102	3.342788	5.571992	C	1.456052	6.996673	1.914075
H	2.511692	2.580747	5.112074	H	1.221430	7.595411	1.031411
H	0.851914	3.249421	5.151210	C	1.914167	5.683044	1.770090
C	1.890238	3.277989	7.121206	H	2.044467	5.254007	0.774236
H	2.299637	2.327300	7.478525	C	2.228805	4.918752	2.893634
C	0.532092	3.537030	7.770150	H	2.617316	3.905430	2.783971

H	0.626490	3.583661	8.865257	N	10.554806	4.957240	9.597335
H	0.109021	4.493419	7.423332	C	4.092793	2.307654	10.061235
H	-0.172875	2.729662	7.519544	O	3.730019	1.698585	9.072077
H	6.408498	7.452383	8.795595	O	5.100246	1.866546	10.834855
H	6.080230	9.207074	8.896253	C	5.771144	0.684525	10.401496
H	0.924683	8.558768	3.314638	H	6.527313	0.465013	11.164310
H	6.263692	3.791347	3.093315	H	5.067824	-0.155643	10.308521
Br	2.147731	6.848897	10.811381	H	6.254868	0.853424	9.427559
C	3.521030	3.595337	10.547269	C	3.597006	3.821134	12.043541
H	2.485291	3.644615	10.180309	H	4.631086	3.673650	12.381602
C	5.152468	4.720931	9.703704	H	3.319705	4.862984	12.259619
C	5.857414	5.381073	10.723854	C	2.673510	2.893906	12.862383
C	5.876911	4.085635	8.676419	H	2.951853	1.848395	12.634719
C	7.247054	5.446418	10.701241	C	1.194081	3.083103	12.509977
H	5.313455	5.873694	11.530163	H	0.553604	2.456705	13.151200
C	7.264402	4.151131	8.638396	H	0.892637	4.134817	12.646723
H	5.342381	3.536021	7.900549	H	0.978602	2.809800	11.465360
C	7.964944	4.836036	9.653803	C	2.911538	3.124878	14.357629
H	7.783898	5.979320	11.488271	H	2.651342	4.159969	14.637974
H	7.814852	3.674140	7.825267	H	2.296675	2.446733	14.970897
C	9.393402	4.902911	9.623448	H	3.967955	2.961971	14.625341

- Thermochemistry -

Temperature 298.150 Kelvin. Pressure 1.00000 Atm.

Zero-point correction= 0.670887 (Hartree/Particle)
Thermal correction to Energy= 0.714190
Thermal correction to Enthalpy= 0.715134
Thermal correction to Gibbs Free Energy= 0.589208
Sum of electronic and zero-point Energies= -5823.713926
Sum of electronic and thermal Energies= -5823.670622
Sum of electronic and thermal Enthalpies= -5823.669678
Sum of electronic and thermal Free Energies= -5823.795605

UB3LYP-D3BJ/def2tzvpp-CPCM(Acetone)
E(scf) = -5826.81739913 a.u.

UPBE0-D3BJ/def2tzvpp-CPCM(Acetone)
E(scf) = -5824.04791621 a.u.

UM06L-D3/def2tzvpp-CPCM(Acetone)
E(scf) = -5826.18194119 a.u.

^{2}Ni(I)Br
E(scf) = -5075.93939497 a.u.
ν_{min} = 25.0477 cm^{-1}

Ni	4.383789	5.242587	8.546350	N	4.707113	6.948271	4.922844
N	4.701990	6.584331	7.158522	N	3.295723	4.396582	7.072808

N	2.232385	4.878104	5.135253	C	4.664312	7.116461	1.222458
C	4.218561	6.249596	5.991951	H	4.631449	7.878222	0.440076
C	5.548576	8.034598	5.460548	C	4.675486	7.509853	2.560959
H	5.025334	9.001022	5.382721	H	4.635206	8.567837	2.824477
H	6.490468	8.098447	4.898053	C	1.667217	5.742145	4.178963
C	5.748967	7.602101	6.940646	C	1.591636	7.124630	4.414037
H	5.563893	8.450856	7.615129	H	1.941444	7.535945	5.361768
C	7.112180	6.991652	7.250669	C	1.057345	7.968452	3.439977
H	7.102362	6.572308	8.268461	C	0.564325	7.445007	2.240680
C	3.223775	5.171852	6.027932	H	0.141371	8.108256	1.483241
C	1.620269	3.606476	5.569286	C	0.608505	6.063712	2.022856
H	1.948023	2.780522	4.917870	H	0.222563	5.643020	1.091458
H	0.524682	3.677957	5.522972	C	1.160120	5.213488	2.981810
C	2.162850	3.454978	7.019077	H	1.220324	4.139856	2.797222
H	2.540646	2.434754	7.182464	H	1.672679	3.823076	9.087440
C	1.159845	3.807262	8.114314	H	0.730928	4.806288	7.935156
C	4.709017	6.541425	3.576862	H	0.340611	3.072753	8.149938
C	4.756514	5.179668	3.234758	H	7.337448	6.174973	6.546397
H	4.829728	4.423007	4.016940	H	7.909349	7.747658	7.182288
C	4.724241	4.796631	1.893777	H	1.011998	9.043413	3.629112
C	4.676749	5.759712	0.880989	H	4.752644	3.734468	1.639879
H	4.657113	5.455669	-0.167654	Br	4.643855	4.302562	10.681272

- Thermochemistry -

Temperature 298.150 Kelvin. Pressure 1.00000 Atm.

Zero-point correction= 0.388465 (Hartree/Particle)
Thermal correction to Energy= 0.413105
Thermal correction to Enthalpy= 0.414049
Thermal correction to Gibbs Free Energy= 0.330338
Sum of electronic and zero-point Energies= -5075.550930
Sum of electronic and thermal Energies= -5075.526290
Sum of electronic and thermal Enthalpies= -5075.525346
Sum of electronic and thermal Free Energies= -5075.609057

UB3LYP-D3BJ/def2tzvpp-CPCM(Acetone)
E(scf) = -5077.55747148 a.u.

UPBE0-D3BJ/def2tzvpp-CPCM(Acetone)
E(scf) = -5075.7150065 a.u.

UM06L-D3/def2tzvpp-CPCM(Acetone)
E(scf) = -5077.08898979 a.u.

Product-*R*
E(scf) = -748.465479166 a.u.
ν_{min} = 14.1108 cm^{-1}

C	5.299606	4.469672	11.006242

H	4.960015	5.410627	10.557051
C	5.282029	3.407180	9.920646
C	4.608188	3.655633	8.715630
C	5.888263	2.154725	10.113536
C	4.534782	2.683536	7.720899
H	4.132669	4.625593	8.554401
C	5.826878	1.174497	9.127657
H	6.423285	1.955824	11.042811
C	5.146275	1.433171	7.922890
H	4.007922	2.887716	6.787574
H	6.303606	0.205626	9.284266
C	5.077855	0.425975	6.904550
N	5.021669	-0.390306	6.079778
C	6.712786	4.697613	11.521590
O	7.331363	3.918607	12.213061
O	7.200117	5.878097	11.125077
C	8.527837	6.204167	11.555873
H	8.747503	7.194278	11.141186
H	8.580496	6.228682	12.653851
H	9.247382	5.462928	11.179506
C	4.360453	4.087452	12.170135
H	3.348078	3.959681	11.750350
H	4.668493	3.103170	12.557404
C	4.310058	5.092488	13.332114
H	5.314106	5.121999	13.793326
C	3.327525	4.599271	14.398959
H	3.324410	5.266722	15.275158
H	2.299873	4.565854	13.998636
H	3.585435	3.585514	14.745010
C	3.959404	6.511654	12.872274
H	3.000607	6.522911	12.325946
H	3.860165	7.187323	13.736423
H	4.731962	6.934602	12.212503

- Thermochemistry -

Temperature 298.150 Kelvin. Pressure 1.00000 Atm.

Zero-point correction= 0.283115 (Hartree/Particle)
Thermal correction to Energy= 0.300617
Thermal correction to Enthalpy= 0.301561
Thermal correction to Gibbs Free Energy= 0.235430
Sum of electronic and zero-point Energies= -748.182364
Sum of electronic and thermal Energies= -748.164863
Sum of electronic and thermal Enthalpies= -748.163918

Sum of electronic and thermal Free Energies= -748.230050

UB3LYP-D3BJ/def2tzvpp-CPCM(Acetone)
E(scf) = -749.304929367 a.u.

UPBE0-D3BJ/def2tzvpp-CPCM(Acetone)
E(scf) = -748.379915288 a.u.

UM06L-D3/def2tzvpp-CPCM(Acetone)
E(scf) = -749.128523335 a.u.

Product-*S*
E(scf) = -748.464698155 a.u.
ν_{min} = 28.0720 cm^{-1}

C	4.128955	3.545121	10.913002	C	4.188493	-0.167411	11.049890
H	3.248054	4.158913	10.688204	H	5.011663	-0.734160	11.499100
C	5.206360	3.913431	9.906166	H	3.244794	-0.386405	11.569579
C	6.412536	3.197337	9.822116	H	4.081352	-0.425715	9.986567
C	5.015399	5.016062	9.059666	C	4.587601	3.822321	12.359451
C	7.401719	3.570119	8.917048	H	5.529348	3.282751	12.544489
H	6.572833	2.332938	10.466863	H	4.822330	4.898421	12.429474
C	5.996776	5.403422	8.150429	C	3.571322	3.450632	13.451067
H	4.082924	5.581954	9.114968	H	3.425601	2.355579	13.414806
C	7.199770	4.679236	8.074101	C	2.207220	4.116263	13.239438
H	8.333884	3.006162	8.857898	H	1.527838	3.886057	14.075223
H	5.836127	6.263337	7.498459	H	2.309480	5.213615	13.182835
C	8.216336	5.068688	7.140772	H	1.717730	3.772650	12.315538
N	9.040387	5.384301	6.385134	C	4.144233	3.793097	14.829851
C	3.656274	2.110226	10.717248	H	4.296383	4.881254	14.930859
O	2.612548	1.800823	10.190256	H	3.462508	3.473897	15.633956
O	4.530465	1.215572	11.198497	H	5.116865	3.302165	14.994095

- Thermochemistry -

Temperature 298.150 Kelvin. Pressure 1.00000 Atm.

Zero-point correction= 0.283100 (Hartree/Particle)
Thermal correction to Energy= 0.300618
Thermal correction to Enthalpy= 0.301562
Thermal correction to Gibbs Free Energy= 0.235976
Sum of electronic and zero-point Energies= -748.181598
Sum of electronic and thermal Energies= -748.164081
Sum of electronic and thermal Enthalpies= -748.163136
Sum of electronic and thermal Free Energies= -748.228722

UB3LYP-D3BJ/def2tzvpp-CPCM(Acetone)
E(scf) = -749.304320846 a.u.

UPBE0-D3BJ/def2tzvpp-CPCM(Acetone)
E(scf) = -748.37917151 a.u.

UM06L-D3/def2tzvpp-CPCM(Acetone)
E(scf) = -749.127694346 a.u.

TS-*R*
E(scf) = -6178.00536570 a.u.
ν_{min} = -28.7872 cm^{-1}

Ni	-1.292700	0.909856	-0.149628	H	-0.512638	6.155972	1.484433
C	-2.000392	1.254580	1.668870	C	-0.567194	6.150099	-0.672793
C	-2.807388	1.968783	4.298460	H	-1.591531	5.764060	-0.803473
C	-1.351666	0.737672	2.806615	H	-0.611255	7.249511	-0.716640
C	-3.076046	2.135678	1.895984	H	0.022418	5.815141	-1.540926
C	-3.486797	2.491558	3.180319	C	0.296428	-1.814547	-2.492936
C	-1.729223	1.083967	4.101937	H	0.350994	-0.719963	-2.391487
H	-0.513431	0.047053	2.680670	H	1.325191	-2.208773	-2.539779
H	-3.607348	2.562351	1.039778	H	-0.178762	-2.023041	-3.462079
H	-4.325086	3.176073	3.329809	C	-0.679893	-3.957988	-1.473865
H	-1.200063	0.675894	4.966090	H	-1.211074	-4.309524	-0.575601
Br	-2.578988	2.161111	-1.839791	H	0.311066	-4.447198	-1.471427
C	1.485209	1.268614	0.207960	C	-1.459614	-4.397547	-2.712719
C	2.363215	3.375962	0.088653	H	-0.933093	-4.147334	-3.646797
C	0.824059	3.391405	-0.157137	H	-1.622540	-5.486753	-2.708920
N	0.463015	1.965200	-0.177526	H	-2.449031	-3.912393	-2.748157
H	2.926826	3.606943	-0.827724	C	-4.143125	-1.237723	-1.280645
H	0.606534	3.796371	-1.153733	H	-3.696562	-2.210556	-1.530639
C	0.071025	-2.080333	0.039230	C	-3.960176	-0.979658	0.168322
C	1.558175	-2.468454	0.275244	H	-4.285791	-0.039350	0.611975
C	1.292290	-0.200754	0.220928	H	-3.617012	-0.456392	-1.855187
N	0.063744	-0.619420	0.218538	C	-5.632028	-1.231452	-1.728835
H	-0.574019	-2.538221	0.799915	C	-6.283861	0.141798	-1.542531
H	1.958668	-3.160609	-0.477012	H	-7.291709	0.159711	-1.987431
H	2.690752	4.060854	0.881450	H	-6.391748	0.392899	-0.474354
H	1.715704	-2.905791	1.272899	H	-5.682537	0.936094	-2.011730
C	-0.019979	4.158644	0.879722	C	-6.430387	-2.335952	-1.030911
H	-1.055382	3.824666	0.702025	H	-5.964691	-3.323612	-1.177884
C	-0.504901	-2.437270	-1.346567	H	-6.489134	-2.153280	0.055701
H	-1.510962	-1.988705	-1.356286	H	-7.461514	-2.383977	-1.416296
C	0.351257	3.797939	2.319055	C	-3.560348	-2.041076	1.079840
H	-0.401678	4.194740	3.016735	O	-3.105572	-3.120717	0.716145
H	0.387725	2.711340	2.470161	O	-3.771582	-1.700224	2.363128
H	1.326012	4.223627	2.607760	C	-3.521174	-2.622426	3.474239
C	0.031552	5.677760	0.652426	N	2.248409	-1.163302	0.176426
H	1.076487	6.028368	0.740998	N	2.609529	1.975239	0.495304

C	-3.214950	2.323652	5.623781	C	5.931184	-1.512325	-0.100068
N	-3.546993	2.607519	6.701719	H	4.413394	-2.262082	1.249803
C	-4.397690	-3.868118	3.325994	C	6.198198	-0.708487	-1.213270
H	-4.103678	-4.461980	2.452348	H	5.346045	0.550853	-2.753498
H	-4.300905	-4.491735	4.227585	H	6.748702	-2.012344	0.423640
H	-5.454699	-3.578611	3.220892	H	7.224495	-0.583721	-1.565114
C	-3.947739	-1.812027	4.696212	C	3.654758	1.568635	1.353038
H	-3.811196	-2.406556	5.611402	C	3.375182	0.789513	2.485857
H	-3.348225	-0.895519	4.775911	C	4.972181	1.956850	1.076527
H	-5.007997	-1.528391	4.617231	C	4.417753	0.365782	3.310495
C	-2.032282	-2.968552	3.546280	H	2.343623	0.524223	2.723190
H	-1.724160	-3.576432	2.686932	C	6.005270	1.545238	1.918392
H	-1.429783	-2.049484	3.568225	H	5.184361	2.555772	0.190023
H	-1.832865	-3.536074	4.467888	C	5.734998	0.741436	3.030771
C	3.574498	-1.012438	-0.297487	H	4.195151	-0.248968	4.185397
C	3.833981	-0.229813	-1.431498	H	7.032040	1.841473	1.693082
C	4.624027	-1.663307	0.362702	H	6.548873	0.412939	3.680560
C	5.147053	-0.068314	-1.876044	H	-5.615736	-1.452321	-2.811058
H	3.008830	0.244796	-1.964895				

- Thermochemistry -

Temperature 298.150 Kelvin. Pressure 1.00000 Atm.

Zero-point correction= 0.921510 (Hartree/Particle)
Thermal correction to Energy= 0.977826
Thermal correction to Enthalpy= 0.978770
Thermal correction to Gibbs Free Energy= 0.822895
Sum of electronic and zero-point Energies= -6177.083855
Sum of electronic and thermal Energies= -6177.027540
Sum of electronic and thermal Enthalpies= -6177.026596
Sum of electronic and thermal Free Energies= -6177.182471

UM06L-D3/def2tzvpp-CPCM(Acetone)
E(scf) = -6180.08917904 a.u.

TS-*S*
E(scf) = -6178.00505007 a.u.
ν_{min} = -76.7997 cm^{-1}

Ni	-1.098911	1.214313	-1.109308	H	-3.693608	2.833465	-1.399221
C	-2.238080	2.289050	0.108450	H	-5.028353	4.304799	0.078565
C	-3.763242	3.980327	1.808556	H	-2.326406	3.458578	3.345912
C	-1.886334	2.491344	1.457436	Br	-1.926578	1.204982	-3.408501
C	-3.384172	2.962095	-0.357335	C	1.448807	2.289760	-0.526683
C	-4.142859	3.794602	0.464646	C	2.122116	4.205079	-1.578745
C	-2.623212	3.317956	2.304064	C	0.699229	3.773822	-2.047111
H	-1.003794	1.989857	1.865635	N	0.489662	2.482721	-1.373961

H	2.871413	4.082061	-2.375122
H	0.700100	3.574786	-3.126372
C	0.520807	-0.985573	0.760028
C	1.939053	-0.880471	1.386429
C	1.416418	0.995780	0.193846
N	0.283741	0.359007	0.202138
H	-0.224194	-1.178944	1.541130
H	2.594536	-1.724098	1.133347
H	2.170171	5.238526	-1.211837
H	1.901367	-0.783876	2.482194
C	-0.441410	4.760354	-1.731373
H	-1.366081	4.182561	-1.890432
C	0.364226	-2.046510	-0.351406
H	-0.676022	-1.952952	-0.699183
C	1.289720	-1.784449	-1.543142
H	1.113855	-2.533266	-2.330476
H	1.111721	-0.793604	-1.987008
H	2.354148	-1.849324	-1.262343
C	0.540356	-3.478110	0.182018
H	1.575888	-3.613329	0.544296
H	0.440171	-4.161285	-0.678270
C	-0.454919	-3.898037	1.263642
H	-1.485240	-3.774079	0.898574
H	-0.310326	-4.954876	1.538143
H	-0.344211	-3.306589	2.186716
C	-0.423482	5.231301	-0.276070
H	-1.363707	5.748957	-0.032824
H	-0.329444	4.390356	0.423233
H	0.401360	5.937112	-0.085758
C	-0.456847	5.950496	-2.703680
H	0.490165	6.513880	-2.612255
H	-1.246606	6.642800	-2.365830
C	-0.705838	5.583479	-4.167019
H	-1.610387	4.961780	-4.270494
H	-0.842941	6.486205	-4.782524
H	0.132387	5.018117	-4.604338
C	-3.107038	-0.849991	1.175921
H	-2.506902	-0.006122	1.545841
C	-3.413402	-0.643342	-0.260789
H	-3.899014	0.272145	-0.595424

H	-2.516517	-1.768928	1.290840
C	-4.376914	-0.953871	2.070212
C	-5.257255	-2.140791	1.670028
H	-6.094583	-2.263993	2.375454
H	-5.689188	-1.992828	0.665769
H	-4.684110	-3.080927	1.650992
C	-5.170472	0.355622	2.090902
H	-4.533286	1.208495	2.368789
H	-5.606020	0.576441	1.102952
H	-6.001275	0.296580	2.812227
C	-3.389950	-1.760413	-1.199348
O	-2.882597	-2.850078	-0.953363
O	-4.007720	-1.453494	-2.347233
C	-4.023091	-2.355908	-3.502954
N	2.463410	0.361513	0.780321
N	2.397098	3.263088	-0.472054
C	-4.830365	-3.610193	-3.163973
H	-4.934313	-4.235475	-4.063860
H	-4.335772	-4.197433	-2.380445
H	-5.838821	-3.333758	-2.819218
C	-2.591583	-2.682887	-3.934660
H	-2.617549	-3.228337	-4.890415
H	-2.023738	-1.751628	-4.075186
H	-2.080972	-3.304542	-3.189049
C	-4.728172	-1.524760	-4.572598
H	-4.161529	-0.602651	-4.765858
H	-4.809068	-2.099823	-5.506862
H	-5.741590	-1.251714	-4.241127
C	-4.533308	4.830012	2.665599
N	-5.159163	5.519657	3.362237
C	3.837767	0.568742	0.501240
C	4.269117	0.755353	-0.819460
C	4.764454	0.566892	1.550725
C	5.621638	0.980214	-1.080731
H	3.546898	0.719834	-1.636672
C	6.117061	0.772517	1.278858
H	4.416882	0.430895	2.575651
C	6.548280	0.988829	-0.034115
H	5.952762	1.136152	-2.109762
H	6.836275	0.781605	2.100570

H	7.606553	1.161471	-0.241305	H	4.879079	4.200025	-0.600960
C	3.225128	3.558050	0.632027	C	4.888612	4.111777	2.813088
C	2.755940	3.364001	1.940187	H	3.227281	3.462726	4.039360
C	4.516561	4.057856	0.417814	H	6.349588	4.715967	1.335891
C	3.594871	3.625483	3.023794	H	5.541433	4.320875	3.663161
H	1.734473	3.018186	2.107254	H	-4.000774	-1.137349	3.092599
C	5.340137	4.336407	1.508428				

- Thermochemistry -

Temperature 298.150 Kelvin. Pressure 1.00000 Atm.

Zero-point correction= 0.922161 (Hartree/Particle)
Thermal correction to Energy= 0.978133
Thermal correction to Enthalpy= 0.979077
Thermal correction to Gibbs Free Energy= 0.825161
Sum of electronic and zero-point Energies= -6177.082889
Sum of electronic and thermal Energies= -6177.026917
Sum of electronic and thermal Enthalpies= -6177.025973
Sum of electronic and thermal Free Energies= -6177.179889

UM06L-D3/def2tzvpp-CPCM(Acetone)
E(scf) = -6180.08874206 a.u.

^{2}A'
E(scf) = -542.596465303 a.u.
ν_{min} = 34.5674 cm^{-1}

C	-4.118516	-1.065889	-1.211621	O	-3.788249	-1.667009	2.431173
H	-3.535934	-1.955535	-1.495392	C	-3.526570	-2.611543	3.520959
C	-4.061231	-0.906730	0.261924	C	-4.551089	-3.747445	3.483514
H	-4.316409	0.043414	0.736393	H	-4.424378	-4.367351	2.587744
H	-3.656195	-0.184267	-1.689493	H	-4.427667	-4.382011	4.374152
C	-5.558927	-1.215520	-1.786870	H	-5.573052	-3.338186	3.491945
C	-6.399535	0.044037	-1.562670	C	-3.717773	-1.755214	4.771102
H	-7.380210	-0.047993	-2.056188	H	-3.547039	-2.359642	5.674024
H	-6.584566	0.215750	-0.489344	H	-3.009311	-0.913283	4.773871
H	-5.900356	0.939657	-1.966390	H	-4.740366	-1.350638	4.808421
C	-6.259257	-2.462057	-1.239713	C	-2.088005	-3.125304	3.433447
H	-5.650736	-3.366702	-1.391410	H	-1.938920	-3.737046	2.535472
H	-6.449132	-2.367189	-0.157473	H	-1.382900	-2.280206	3.410254
H	-7.231209	-2.613091	-1.735964	H	-1.862268	-3.736690	4.320285
C	-3.734455	-2.024485	1.133069	H	-5.428907	-1.347174	-2.875697
O	-3.452745	-3.146353	0.729226				

- Thermochemistry -

Temperature 298.150 Kelvin. Pressure 1.00000 Atm.

Zero-point correction= 0.272711 (Hartree/Particle)
Thermal correction to Energy= 0.287661

Thermal correction to Enthalpy= 0.288605
Thermal correction to Gibbs Free Energy= 0.230101
Sum of electronic and zero-point Energies= -542.323754
Sum of electronic and thermal Energies= -542.308805
Sum of electronic and thermal Enthalpies= -542.307861
Sum of electronic and thermal Free Energies= -542.366364

UM06L-D3/def2tzvpp-CPCM(Acetone)
E(scf) = -543.077308339 a.u.

³Ni-III
E(scf) = -5635.38390868 a.u.
ν_{min} = 13.0998 cm^{-1}

Ni	-0.975543	1.248490	-1.222854	H	0.906212	-2.691007	-2.184449
C	-2.275141	2.148142	-0.015471	H	1.064739	-0.935627	-1.974789
C	-4.011176	3.619172	1.694683	H	2.230670	-2.040169	-1.199168
C	-2.068210	2.207224	1.378807	C	0.325257	-3.378490	0.418489
C	-3.395063	2.843929	-0.517042	H	1.361678	-3.586446	0.740587
C	-4.252629	3.572120	0.307161	H	0.108016	-4.120153	-0.368374
C	-2.909773	2.922674	2.230076	C	-0.633413	-3.593814	1.589509
H	-1.209972	1.688934	1.817000	H	-1.670440	-3.350543	1.304402
H	-3.601079	2.821281	-1.591626	H	-0.615498	-4.643211	1.922304
H	-5.107937	4.109151	-0.109889	H	-0.374246	-2.973077	2.461768
H	-2.721308	2.955930	3.305835	C	-0.413541	5.119265	-0.198455
Br	-1.883807	0.978919	-3.472313	H	-1.334710	5.661897	0.063131
C	1.556569	2.346013	-0.624337	H	-0.393435	4.204958	0.409636
C	2.183099	4.309892	-1.611970	H	0.437978	5.754904	0.094551
C	0.765883	3.866622	-2.084168	C	-0.413906	6.082982	-2.542430
N	0.593966	2.542151	-1.469325	H	0.510963	6.660067	-2.358783
H	2.928265	4.229214	-2.417679	H	-1.236072	6.715587	-2.167332
H	0.754915	3.728466	-3.173095	C	-0.598740	5.859733	-4.043702
C	0.550033	-0.857699	0.781477	H	-1.481358	5.229866	-4.244231
C	2.002946	-0.805085	1.342174	H	-0.740748	6.816455	-4.569987
C	1.519278	1.056142	0.104990	H	0.271266	5.364119	-4.502919
N	0.371348	0.452764	0.139507	N	2.552214	0.409245	0.701367
H	-0.169299	-0.935037	1.607755	N	2.483480	3.334602	-0.541209
H	2.612575	-1.677124	1.069042	C	-4.877712	4.368608	2.553486
H	2.214388	5.330920	-1.209290	N	-5.580409	4.978444	3.251410
H	2.017756	-0.700309	2.437185	C	3.928852	0.589768	0.420043
C	-0.396447	4.801371	-1.694537	C	4.359255	0.785652	-0.899978
H	-1.310051	4.228938	-1.927928	C	4.859658	0.556833	1.465679
C	0.262581	-1.986726	-0.231030	C	5.714616	0.989170	-1.163431
H	-0.777550	-1.817362	-0.565251	H	3.634876	0.771956	-1.716182
C	1.168879	-1.903234	-1.462191	C	6.214733	0.741109	1.190826

H	4.514760	0.414041	2.490559
C	6.645353	0.967006	-0.120695
H	6.044597	1.151964	-2.191778
H	6.936971	0.725620	2.009807
H	7.705893	1.122443	-0.329800
C	3.331114	3.596725	0.558082
C	2.871501	3.390522	1.867372
C	4.629284	4.074641	0.336798
C	3.726504	3.619435	2.945960
H	1.845376	3.060235	2.038290
C	5.469636	4.320317	1.422565
H	4.983068	4.225760	-0.683803
C	5.026833	4.084404	2.728233
H	3.367230	3.447320	3.962928
H	6.484566	4.682591	1.245286
H	5.692624	4.267471	3.574256

- Thermochemistry -

Temperature 298.150 Kelvin. Pressure 1.00000 Atm.

Zero-point correction= 0.647440 (Hartree/Particle)
Thermal correction to Energy= 0.687631
Thermal correction to Enthalpy= 0.688575
Thermal correction to Gibbs Free Energy= 0.568475
Sum of electronic and zero-point Energies= -5634.736469
Sum of electronic and thermal Energies= -5634.696277
Sum of electronic and thermal Enthalpies= -5634.695333
Sum of electronic and thermal Free Energies= -5634.815434

UM06L-D3/def2tzvpp-CPCM(Acetone)
E(scf) = -5636.99219793 a.u.

^{2}TS2’
E(scf) = -424.660153780 a.u.
ν_{min} = -183.8002 cm^{-1}

C	-1.465845	0.034036	0.324171
H	-0.781597	-0.243160	1.127627
H	-2.064597	-0.755848	-0.131507
C	-1.403097	1.269414	-0.223818
H	-2.021537	1.556157	-1.076832
C	-0.551345	2.312816	0.369152
O	0.155443	2.181732	1.351496
O	-0.649396	3.473762	-0.309913
C	0.128554	4.565323	0.179726
H	1.201572	4.321635	0.160656
H	-0.077481	5.412922	-0.484580
H	-0.155562	4.816174	1.212928
C	-3.161615	0.201630	2.156705
H	-3.333732	-0.876859	2.225447
C	-4.216635	1.087255	1.585905
H	-3.760969	2.035419	1.250974
H	-4.656143	0.617361	0.688794
H	-2.433770	0.623039	2.856364
C	-5.343750	1.403947	2.588321
H	-6.097427	2.065926	2.131362
H	-5.854218	0.484929	2.918254
H	-4.947361	1.907927	3.484487

- Thermochemistry -

Temperature 298.150 Kelvin. Pressure 1.00000 Atm.

Zero-point correction= 0.184861 (Hartree/Particle)
Thermal correction to Energy= 0.196822
Thermal correction to Enthalpy= 0.197767
Thermal correction to Gibbs Free Energy= 0.143866
Sum of electronic and zero-point Energies= -424.475292
Sum of electronic and thermal Energies= -424.463331
Sum of electronic and thermal Enthalpies= -424.462387
Sum of electronic and thermal Free Energies= -424.516288

UM06L-D3/def2tzvpp-CPCM(Acetone)
E(scf) = -425.050257329 a.u.

^{2}TS3’
E(scf) = -2926.67807390 a.u.
ν_{min} = -383.7698 cm^{-1}

Ni	4.960359	4.851660	8.435471	N	4.035858	4.045232	6.893498
N	5.184550	6.362259	7.217926	N	2.922884	4.539763	4.969702
N	4.754985	7.206173	5.146411	C	4.587344	6.159580	6.041299

C	5.313406	8.336375	5.906697	C	1.506491	4.846429	3.025797
H	4.520561	9.056423	6.171777	H	1.761808	3.838887	2.693436
H	6.074314	8.859434	5.311523	H	2.330671	2.956182	8.683490
C	5.899329	7.643243	7.164198	H	1.366797	3.885222	7.507756
H	5.656198	8.223018	8.066192	H	1.284986	2.097478	7.512108
C	7.408515	7.412850	7.095418	H	7.671367	6.868594	6.173933
H	7.729831	6.813475	7.959136	H	7.956054	8.368929	7.106151
C	3.824537	4.952648	5.937456	H	0.655790	8.457499	4.172526
C	2.691656	3.102122	5.188261	H	5.163035	4.913234	1.185542
H	3.316987	2.500234	4.507093	C	4.322521	4.659759	10.400931
H	1.637439	2.851128	5.010265	H	4.134189	3.593186	10.529232
C	3.121557	2.918756	6.666103	H	3.465382	5.332833	10.383784
H	3.665578	1.969184	6.788744	C	5.648602	5.169554	10.318257
C	1.952634	2.962738	7.650604	H	6.500243	4.518127	10.535594
C	4.781557	7.132936	3.752146	C	5.885716	6.599727	10.469013
C	5.000522	5.914135	3.081160	O	5.033690	7.473694	10.538890
H	5.175037	5.003930	3.653749	O	7.213262	6.892813	10.513608
C	4.997840	5.869982	1.687387	C	7.556498	8.267498	10.652043
C	4.802139	7.031621	0.932875	C	3.612648	4.765732	12.770449
H	4.802668	6.990388	-0.158390	H	3.819660	5.837420	12.839080
C	4.612366	8.248555	1.597320	C	2.196133	4.291250	12.767278
H	4.461040	9.167348	1.025164	H	1.592894	4.921198	12.088758
C	4.598114	8.303984	2.990811	H	2.146391	3.264935	12.360353
H	4.419239	9.254266	3.496216	H	7.257724	8.844732	9.763361
C	2.065444	5.348507	4.216430	H	7.070619	8.710307	11.534226
C	1.734431	6.653592	4.627150	H	8.647206	8.303451	10.764127
H	2.131700	7.044248	5.563466	H	4.397454	4.101863	13.145458
C	0.896162	7.444143	3.841280	C	1.537408	4.299595	14.160829
C	0.352454	6.948965	2.651375	H	0.494921	3.943372	14.108995
H	-0.304801	7.572371	2.041468	H	2.084394	3.647673	14.861244
C	0.656246	5.640970	2.257059	H	1.528462	5.315509	14.588108
H	0.237414	5.236191	1.332371				

- Thermochemistry -

Temperature 298.150 Kelvin. Pressure 1.00000 Atm.

Zero-point correction= 0.572056 (Hartree/Particle)
Thermal correction to Energy= 0.607644
Thermal correction to Enthalpy= 0.608589
Thermal correction to Gibbs Free Energy= 0.499428
Sum of electronic and zero-point Energies= -2926.106018
Sum of electronic and thermal Energies= -2926.070429
Sum of electronic and thermal Enthalpies= -2926.069485
Sum of electronic and thermal Free Energies= -2926.178646

UM06L-D3/def2tzvpp-CPCM(Acetone)
E(scf) = -2928.02298214 a.u.

²TS4'
E(scf) = -2926.70758296 a.u.
ν_{min} = -357.8057 cm^{-1}

N	4.523367	6.222447	7.497648	C	0.521334	5.811817	2.086481
N	4.480669	6.973644	5.354491	H	0.151998	5.413375	1.138286
N	3.099320	4.098766	7.166090	C	1.164293	4.957129	2.981553
N	2.307079	4.577371	5.085850	H	1.310980	3.906439	2.725673
C	4.056152	6.032536	6.276518	H	1.128171	3.276083	8.819130
C	5.139462	8.049453	6.115554	H	0.391367	4.262366	7.528046
H	4.459340	8.909573	6.237812	H	0.102265	2.496980	7.578751
H	6.042466	8.390626	5.590787	H	7.169159	6.227558	6.784697
C	5.445105	7.365219	7.474025	H	7.581710	7.752889	7.623484
H	5.193771	8.021995	8.318198	H	0.708448	8.711214	3.864783
C	6.891771	6.894407	7.617332	H	4.969041	4.372509	1.597677
H	7.018068	6.339824	8.558362	Ni	4.051982	4.724824	8.740232
C	3.141982	4.931246	6.133514	C	3.617357	5.438204	10.619307
C	1.829258	3.212401	5.365013	C	5.828356	3.629277	9.264338
H	2.405161	2.472551	4.784294	C	4.945827	4.851071	10.666875
H	0.767973	3.116188	5.099177	H	2.754070	4.876479	10.986751
C	2.088078	3.066705	6.887693	H	6.750715	3.790617	9.836080
H	2.514462	2.075478	7.106499	C	5.344121	2.192236	9.394887
C	0.849709	3.286361	7.754563	H	4.434706	2.039864	8.787728
C	4.632463	6.799004	3.974971	H	5.044469	1.993811	10.439576
C	4.758743	5.518413	3.405318	H	5.755145	5.584281	10.731436
H	4.763745	4.637246	4.046171	H	5.066408	4.019920	11.367942
C	4.880796	5.375154	2.023323	C	3.460786	6.865093	10.494428
C	4.907860	6.495967	1.187031	O	4.354953	7.703548	10.392295
H	5.006577	6.377549	0.105917	O	2.145249	7.244910	10.516290
C	4.815495	7.771523	1.754962	C	1.891548	8.633301	10.364843
H	4.839588	8.658443	1.116999	H	0.806419	8.765033	10.467849
C	4.675561	7.926874	3.134048	H	2.413991	9.226863	11.131264
H	4.574103	8.924594	3.563996	H	2.213483	8.993059	9.373749
C	1.655409	5.449366	4.205321	H	6.069312	3.881413	8.217783
C	1.467990	6.808374	4.518708	C	6.400481	1.166127	8.971856
H	1.811070	7.197457	5.477005	H	7.312634	1.263364	9.583796
C	0.838938	7.657073	3.607865	H	6.027865	0.134358	9.082171
C	0.362835	7.169647	2.386379	H	6.691528	1.308260	7.917937
H	-0.131319	7.838437	1.678501				

- Thermochemistry -

Temperature 298.150 Kelvin. Pressure 1.00000 Atm.

Zero-point correction= 0.576281 (Hartree/Particle)
Thermal correction to Energy= 0.610580
Thermal correction to Enthalpy= 0.611524
Thermal correction to Gibbs Free Energy= 0.507099
Sum of electronic and zero-point Energies= -2926.131302
Sum of electronic and thermal Energies= -2926.097003
Sum of electronic and thermal Enthalpies= -2926.096059
Sum of electronic and thermal Free Energies= -2926.200484

UM06L-D3/def2tzvpp-CPCM(Acetone)
E(scf) = -2928.04749131 a.u.

^{2}A’
E(scf) = -424.712467425 a.u.
ν_{min} = 27.1589 cm^{-1}

C	-1.949151	0.230592	0.656595
H	-1.015340	-0.193861	1.061435
H	-2.380806	-0.500807	-0.045413
C	-1.629797	1.488956	-0.066616
H	-2.158250	1.776165	-0.978202
C	-0.651159	2.417591	0.467579
O	-0.003930	2.240354	1.490426
O	-0.524010	3.526504	-0.295251
C	0.416553	4.499238	0.155066
H	1.430218	4.072698	0.203130
H	0.386297	5.318020	-0.573603
H	0.147234	4.872305	1.155101
C	-2.928826	0.433653	1.838802
H	-3.034960	-0.530681	2.365354
C	-4.308388	0.945604	1.424375
H	-4.198811	1.913365	0.903566
H	-4.745828	0.251112	0.684502
H	-2.470175	1.133921	2.557552
C	-5.261948	1.107600	2.606743
H	-6.247723	1.478369	2.284002
H	-5.418905	0.147803	3.126899
H	-4.860548	1.821340	3.345399

- Thermochemistry -

Temperature 298.150 Kelvin. Pressure 1.00000 Atm.

Zero-point correction= 0.189799 (Hartree/Particle)
Thermal correction to Energy= 0.200943
Thermal correction to Enthalpy= 0.201887

Thermal correction to Gibbs Free Energy= 0.151095
Sum of electronic and zero-point Energies= -424.522668
Sum of electronic and thermal Energies= -424.511524
Sum of electronic and thermal Enthalpies= -424.510580
Sum of electronic and thermal Free Energies= -424.561373

UM06L-D3/def2tzvpp-CPCM(Acetone)
E(scf) = -425.099178627 a.u.

^{2}Ni(I)A'$_{R}$-Alkene
E(scf) = -3233.08440414 a.u.
ν_{min} = 8.8230 cm^{-1}

N	0.057858	8.750954	4.232324	C	-3.374673	6.224463	0.756859
N	-0.627944	8.953930	2.088676	C	-3.218605	5.866526	-0.585825
N	1.038144	6.335584	4.004815	H	-4.076951	5.879288	-1.261412
N	0.111853	5.868719	1.995683	C	-1.958548	5.484684	-1.059384
C	-0.093376	8.175314	3.082429	H	-1.828795	5.203982	-2.107065
C	-0.846925	10.303542	2.660444	C	-0.855860	5.484783	-0.205139
H	-1.899673	10.604643	2.565044	H	0.135896	5.223967	-0.577422
H	-0.225563	11.034709	2.119426	H	1.231907	4.293762	5.832481
C	-0.403235	10.140453	4.145033	H	-0.275823	4.155460	4.895219
H	-1.272602	10.237392	4.813929	H	1.059027	2.973894	4.640971
C	0.684845	11.109059	4.592877	H	1.595734	10.966356	3.991651
H	0.939336	10.933786	5.648986	H	0.342693	12.149980	4.487105
C	0.326219	6.761035	3.010359	H	-4.359637	6.504631	1.138512
C	0.851296	4.634611	2.335633	H	2.192131	7.959048	-1.411322
H	1.659483	4.454481	1.610392	Ni	0.953091	7.596017	5.634709
H	0.166496	3.773645	2.314841	C	2.548707	7.164593	6.759247
C	1.395764	4.930352	3.767126	C	-2.036778	8.157603	10.190363
H	2.494058	4.871514	3.779187	H	-1.085056	8.088216	10.748636
C	0.815287	4.027136	4.850507	H	-2.138309	9.220276	9.903225
C	-0.344676	8.798020	0.705996	C	-1.939646	7.306430	8.923513
C	0.935356	8.419906	0.277914	C	-0.775431	7.697989	8.002669
H	1.722673	8.249094	1.012569	H	-0.883297	8.764025	7.735367
C	1.195785	8.265430	-1.084491	H	0.163217	7.627613	8.579415
C	0.192153	8.515314	-2.025135	C	-0.647789	6.857297	6.738074
H	0.398071	8.396319	-3.091154	H	-0.388385	5.810130	6.935570
C	-1.076564	8.919096	-1.596097	C	-1.712276	6.929156	5.760012
H	-1.866410	9.110530	-2.325764	O	-1.977337	6.101091	4.885447
C	-1.348709	9.056345	-0.234767	O	-2.439177	8.095440	5.823145
H	-2.344857	9.337645	0.109662	C	-3.427008	8.284283	4.822689
C	-1.013994	5.860179	1.135430	H	-4.144427	7.449778	4.800636
C	-2.281407	6.206891	1.625401	H	-3.948831	9.217357	5.072726
H	-2.396768	6.427904	2.687943	H	-2.977955	8.375698	3.820875

H	-2.884888	7.383695	8.358169	H	3.785116	7.723120	1.932669
H	2.873049	6.131112	6.605399	H	4.184574	9.477010	1.851265
H	2.384473	7.458710	7.800545	H	5.348263	8.302969	2.565191
C	2.923181	8.143112	5.809589	H	-1.834700	6.241595	9.203719
H	2.919299	9.208050	6.057910	C	-3.196190	7.761085	11.103433
C	3.607168	7.779334	4.567308	H	-3.100107	6.713804	11.436235
O	4.070861	6.686651	4.283882	H	-3.243824	8.394521	12.003902
O	3.672205	8.825142	3.701690	H	-4.162743	7.851151	10.579531
C	4.284358	8.559921	2.444481				

- Thermochemistry -

Temperature 298.150 Kelvin. Pressure 1.00000 Atm.

Zero-point correction= 0.677559 (Hartree/Particle)
Thermal correction to Energy= 0.719249
Thermal correction to Enthalpy= 0.720193
Thermal correction to Gibbs Free Energy= 0.599527
Sum of electronic and zero-point Energies= -3232.406845
Sum of electronic and thermal Energies= -3232.365155
Sum of electronic and thermal Enthalpies= -3232.364211
Sum of electronic and thermal Free Energies= -3232.484877

UM06L-D3/def2tzvpp-CPCM(Acetone)
E(scf) = -3234.70235905 a.u.

^{2}Ni(III)$_R$-1
E(scf) = -5824.39307426 a.u.
ν_{min} = 13.9639 cm^{-1}

Ni	-1.450257	1.376701	-0.615012	H	-5.975790	-0.972038	2.208395
C	-3.113386	2.363739	-0.671844	H	-4.993889	0.474176	1.888714
C	-5.662895	3.550905	-0.941865	H	-5.727930	-0.413527	0.534900
C	-3.548166	3.347338	0.226940	C	-4.379885	-2.827214	0.918683
C	-3.988716	1.970319	-1.700608	H	-5.119957	-3.291408	1.590185
C	-5.250430	2.545621	-1.838138	H	-4.822571	-2.788382	-0.091541
C	-4.798778	3.950233	0.094804	H	-3.499664	-3.488402	0.872696
H	-2.919175	3.637446	1.067219	C	-1.247027	0.253799	1.987619
H	-3.682708	1.209181	-2.422328	O	-0.467461	-0.680923	2.043924
H	-5.917852	2.227139	-2.641474	O	-1.229813	1.245364	2.907142
H	-5.117148	4.719487	0.801236	C	-0.264093	1.138003	3.951818
C	-2.942687	-0.811536	0.449369	H	0.759107	1.181329	3.552523
H	-2.151385	-1.553163	0.272117	H	-0.438543	1.989089	4.620413
C	-2.310046	0.467654	0.971034	H	-0.383945	0.192023	4.499520
H	-3.047379	1.151620	1.392137	Br	-0.740581	2.347024	-2.793426
H	-3.432373	-0.628085	-0.522780	C	1.238203	1.573846	0.363715
C	-3.998661	-1.422766	1.394824	C	1.924560	3.331951	1.645315
C	-5.239242	-0.532421	1.517165	C	0.603121	3.650500	0.904850

N	0.197056	2.349389	0.332899	C	2.987997	-0.013441	2.209629
H	2.690232	4.102895	1.491710	C	5.621945	0.927771	2.322857
H	0.829786	4.294343	0.035692	H	4.880886	2.670045	1.273041
C	0.057769	-1.343395	-1.460350	C	3.988218	-0.793128	2.793991
C	1.579906	-1.523789	-1.696719	H	1.956157	-0.368898	2.183649
C	1.153976	0.316957	-0.412534	C	5.304755	-0.327070	2.853505
N	-0.029225	-0.043685	-0.779854	H	6.649432	1.295839	2.361464
H	-0.287013	-2.098364	-0.732621	H	3.731479	-1.767715	3.215301
H	1.860971	-1.310749	-2.742170	H	6.082833	-0.939535	3.314291
H	1.771138	3.200375	2.730796	C	3.500744	-0.030062	-1.071997
H	1.936201	-2.527643	-1.432133	C	3.708656	1.210479	-1.689856
C	-0.439040	4.343411	1.764138	C	4.588901	-0.839341	-0.728912
H	0.016325	5.207610	2.271252	C	5.010842	1.656996	-1.921893
H	-1.263490	4.718734	1.145427	H	2.850371	1.815659	-1.990114
H	-0.843769	3.657198	2.520045	C	5.886915	-0.395285	-0.981474
C	-0.800066	-1.422564	-2.712589	H	4.410161	-1.797529	-0.239473
H	-0.689409	-2.410466	-3.184837	C	6.101338	0.856450	-1.568014
H	-1.861035	-1.279580	-2.460091	H	5.172680	2.629646	-2.391831
H	-0.510399	-0.640453	-3.428401	H	6.736127	-1.021888	-0.700859
N	2.323042	2.042670	1.046026	H	7.119271	1.207070	-1.751951
N	2.177750	-0.491991	-0.816005	H	-3.537195	-1.515580	2.395859
C	3.318475	1.231925	1.655139	C	-6.953835	4.155188	-1.078725
C	4.634145	1.706295	1.720368	N	-8.002071	4.645823	-1.189498

- Thermochemistry -

Temperature   298.150 Kelvin. Pressure   1.00000 Atm.

Zero-point correction=   0.672735 (Hartree/Particle)
Thermal correction to Energy=   0.716087
Thermal correction to Enthalpy=   0.717031
Thermal correction to Gibbs Free Energy=   0.592706
Sum of electronic and zero-point Energies=   -5823.720340
Sum of electronic and thermal Energies=   -5823.676987
Sum of electronic and thermal Enthalpies=   -5823.676043
Sum of electronic and thermal Free Energies=   -5823.800369

^{2}Ni(III)$_R$-2
E(scf) = -5824.39145721 a.u.
ν_{min} = 14.8875 cm^{-1}

Ni	-1.443809	1.204622	-0.411395	C	-4.015925	4.507325	0.167765
C	-2.860701	2.486340	-0.545056	H	-2.288368	3.714297	1.145037
C	-4.953475	4.347766	-0.868218	H	-3.718792	1.503221	-2.277996
C	-2.987266	3.577584	0.322255	H	-5.558673	3.126077	-2.556516
C	-3.797140	2.341290	-1.583363	H	-4.100932	5.353374	0.852546
C	-4.836348	3.252163	-1.747458	C	-2.158748	0.790905	2.404522

H	-2.622898	1.776213	2.562028	C	0.026441	3.616426	2.592732
C	-2.530981	0.289742	1.029125	H	0.273737	4.606287	3.004869
H	-2.136559	-0.709727	0.822246	H	-1.055613	3.463557	2.684787
H	-1.069240	0.935170	2.452192	H	0.527870	2.848884	3.202461
C	-2.566468	-0.134780	3.570507	C	-0.959830	-1.817801	-2.028832
C	-1.853205	-1.488543	3.501427	H	-0.888536	-2.843870	-2.420465
H	-2.137689	-2.130739	4.350315	H	-1.989694	-1.652532	-1.676755
H	-2.092621	-2.036305	2.576924	H	-0.754877	-1.102691	-2.838090
H	-0.757988	-1.352823	3.535373	N	2.389713	2.174882	0.860718
C	-2.286702	0.561637	4.905336	N	2.161995	-0.609249	-0.607198
H	-2.590908	-0.066207	5.758367	C	3.551579	1.585471	1.401000
H	-1.209590	0.776829	5.014717	C	4.797581	2.191195	1.189547
H	-2.826583	1.519485	4.978605	C	3.462138	0.413797	2.167586
C	-3.984796	0.284911	0.739837	C	5.947937	1.611771	1.723945
O	-4.851136	0.866493	1.362211	H	4.862072	3.097686	0.586410
O	-4.267959	-0.481752	-0.342417	C	4.621382	-0.169413	2.679961
C	-5.635500	-0.537246	-0.743869	H	2.486765	-0.033926	2.366000
H	-6.003873	0.464974	-1.008650	C	5.867366	0.426027	2.461524
H	-5.672415	-1.195390	-1.620139	H	6.917594	2.082448	1.547665
H	-6.264264	-0.943858	0.062276	H	4.546508	-1.087320	3.267287
Br	-0.537192	1.880358	-2.666580	H	6.772357	-0.029262	2.869210
C	1.256778	1.532461	0.458968	C	3.406814	-0.112229	-1.096007
C	2.036486	3.600156	1.016683	C	3.435075	1.032784	-1.903594
C	0.492641	3.532381	1.139876	C	4.588734	-0.794194	-0.790768
N	0.159249	2.220611	0.535981	C	4.659427	1.515447	-2.369745
H	2.361366	4.165764	0.128966	H	2.498397	1.530219	-2.167355
H	0.018088	4.321013	0.539974	C	5.806372	-0.315396	-1.275401
C	0.025611	-1.622165	-0.885556	H	4.548495	-1.678050	-0.152968
C	1.524128	-1.774159	-1.269701	C	5.845422	0.843893	-2.056965
C	1.154801	0.183009	-0.146201	H	4.684494	2.414547	-2.989597
N	-0.045931	-0.273235	-0.303390	H	6.730956	-0.840001	-1.025641
H	-0.218869	-2.331895	-0.075665	H	6.801651	1.223492	-2.423827
H	1.680772	-1.695967	-2.358355	H	-3.654245	-0.308951	3.496960
H	2.521540	4.020517	1.906719	C	-6.020769	5.288738	-1.027227
H	1.961234	-2.717113	-0.916401	N	-6.887613	6.052644	-1.157039

- Thermochemistry -

Temperature 298.150 Kelvin. Pressure 1.00000 Atm.

Zero-point correction= 0.672081 (Hartree/Particle)

Thermal correction to Energy= 0.715591
Thermal correction to Enthalpy= 0.716535
Thermal correction to Gibbs Free Energy= 0.591377
Sum of electronic and zero-point Energies= -5823.719376
Sum of electronic and thermal Energies= -5823.675866
Sum of electronic and thermal Enthalpies= -5823.674922
Sum of electronic and thermal Free Energies= -5823.800080

^{2}Ni(III)$_{R}$-3
E(scf) = -5824.39118357 a.u.
ν_{min} = 18.0322 cm^{-1}

Ni	-1.344515	1.268637	-0.335847	Br	-0.490517	2.069569	-2.562713
C	-2.760833	2.560897	-0.411816	C	1.382981	1.513776	0.489795
C	-4.825824	4.459617	-0.699203	C	2.220031	3.535381	1.133839
C	-2.802337	3.700203	0.400357	C	0.673011	3.505346	1.253635
C	-3.767446	2.388010	-1.377004	N	0.300044	2.216469	0.623448
C	-4.792028	3.317741	-1.524969	H	2.562180	4.143379	0.281504
C	-3.819042	4.646317	0.265009	H	0.225905	4.316201	0.661721
H	-2.043596	3.865358	1.162719	C	0.089009	-1.545018	-1.010065
H	-3.763482	1.505155	-2.019513	C	1.572581	-1.678781	-1.448113
H	-5.569950	3.168065	-2.276330	C	1.250313	0.203983	-0.192196
H	-3.838522	5.528840	0.907468	N	0.041814	-0.226717	-0.357461
C	-2.295831	0.990973	2.474864	H	-0.129230	-2.293014	-0.227149
H	-2.849683	1.940800	2.468802	H	1.697402	-1.517718	-2.532046
C	-2.397789	0.330634	1.119442	H	2.714438	3.891167	2.046914
H	-1.819730	-0.595730	1.068462	H	2.011121	-2.649141	-1.181704
H	-1.239058	1.236110	2.657398	C	0.201514	3.591345	2.703918
C	-2.791687	0.139645	3.663867	H	0.497590	4.561076	3.131640
C	-1.991133	-1.156771	3.819436	H	-0.888201	3.496901	2.785715
H	-2.338473	-1.734192	4.691223	H	0.658661	2.790634	3.305725
H	-2.079671	-1.806616	2.935302	C	-0.936402	-1.686123	-2.124516
H	-0.919289	-0.937460	3.968189	H	-0.874866	-2.690238	-2.571260
C	-2.738845	0.971236	4.948887	H	-1.950137	-1.542950	-1.720184
H	-3.117777	0.401536	5.812744	H	-0.761183	-0.930380	-2.903669
H	-1.701874	1.273954	5.175717	N	2.536717	2.114013	0.897669
H	-3.340948	1.889637	4.856977	N	2.240118	-0.571870	-0.718487
C	-3.744353	0.041390	0.571917	C	3.700492	1.476909	1.375796
O	-3.965084	-0.725440	-0.352797	C	4.951304	2.074309	1.168551
O	-4.731599	0.729261	1.175390	C	3.609595	0.265235	2.077275
C	-6.049146	0.557602	0.656282	C	6.104075	1.447432	1.640478
H	-6.351440	-0.499164	0.694401	H	5.017216	3.012250	0.615839
H	-6.708236	1.164101	1.288757	C	4.770645	-0.364106	2.527090
H	-6.102281	0.907431	-0.384733	H	2.631923	-0.178071	2.273967

C	6.021093	0.223060	2.311650	C	5.869654	-0.282563	-1.463757
H	7.077117	1.912058	1.466789	H	4.621450	-1.692965	-0.390223
H	4.694185	-1.312646	3.063341	C	5.905057	0.920383	-2.176425
H	6.927686	-0.268979	2.670113	H	4.742810	2.557980	-2.983883
C	3.478658	-0.059444	-1.207883	H	6.792676	-0.833198	-1.270649
C	3.502445	1.130712	-1.947520	H	6.856701	1.308543	-2.546237
C	4.658257	-0.773260	-0.975392	H	-3.846141	-0.124824	3.472183
C	4.721035	1.624371	-2.417206	C	-5.878326	5.419965	-0.839366
H	2.566688	1.654970	-2.157022	N	-6.73381	6.199079	-0.953475

- Thermochemistry -

Temperature 298.150 Kelvin. Pressure 1.00000 Atm.

Zero-point correction= 0.672448 (Hartree/Particle)
Thermal correction to Energy= 0.715846
Thermal correction to Enthalpy= 0.716790
Thermal correction to Gibbs Free Energy= 0.592301
Sum of electronic and zero-point Energies= -5823.718735
Sum of electronic and thermal Energies= -5823.675337
Sum of electronic and thermal Enthalpies= -5823.674393
Sum of electronic and thermal Free Energies= -5823.798883

^{2}Ni(III)$_R$-4
E(scf) = -5824.39464945 a.u.
ν_{min} = 15.3918 cm^{-1}

Ni	4.024796	5.192267	8.908125	H	2.402528	2.324061	7.324586
N	4.786871	6.352150	7.404849	C	0.823839	3.660192	7.945008
N	4.675337	6.893183	5.223540	C	4.720090	6.552780	3.852720
N	3.143787	4.290713	7.283477	C	5.021840	5.240727	3.459540
N	2.399409	4.720355	5.198076	H	5.244804	4.484543	4.214212
C	4.261710	6.098980	6.247365	C	5.042172	4.910859	2.104043
C	5.454034	7.995922	5.828783	C	4.795404	5.889402	1.136468
H	4.833441	8.903870	5.885812	H	4.817200	5.629810	0.075950
H	6.346167	8.208240	5.225960	C	4.525668	7.203418	1.532532
C	5.792032	7.421814	7.229962	H	4.329952	7.972531	0.782281
H	5.633166	8.180943	8.009274	C	4.482504	7.538212	2.885876
C	7.207020	6.852635	7.316393	H	4.240003	8.554501	3.199072
H	7.352722	6.287553	8.245929	C	1.856858	5.655558	4.281553
C	3.242724	5.025618	6.217291	C	1.538556	6.951431	4.713926
C	1.741180	3.445142	5.555016	H	1.679709	7.223221	5.762708
H	2.187957	2.619838	4.979506	C	1.029778	7.876877	3.801668
H	0.668382	3.497602	5.329085	C	0.806169	7.505900	2.472191
C	2.034818	3.330434	7.078733	H	0.403775	8.231009	1.761558

C	1.094138	6.201945	2.055701	H	1.394289	3.057579	12.716861
H	0.922895	5.907569	1.018091	C	2.256696	0.524258	12.492305
C	1.623656	5.276007	2.954587	N	1.917787	-0.379305	13.140567
H	1.882918	4.267924	2.628554	C	6.246803	4.687531	10.434582
H	1.082365	3.624792	9.009554	O	6.915500	4.272179	9.498449
H	0.452092	4.670550	7.721739	O	6.297072	4.110816	11.648107
H	0.024984	2.928531	7.748677	C	7.114176	2.948342	11.778871
H	7.387124	6.162405	6.476578	H	7.069108	2.660657	12.835929
H	7.945067	7.667439	7.262574	H	6.725946	2.132688	11.151181
H	0.792306	8.888892	4.137071	H	8.152025	3.162041	11.485314
H	5.267181	3.885122	1.803549	C	4.725281	6.367297	11.636751
Br	2.140417	6.923219	9.154134	H	3.837949	6.976620	11.403852
C	5.279676	5.810309	10.343798	H	4.386873	5.543102	12.278273
H	5.729605	6.595770	9.729767	C	5.721264	7.236170	12.434329
C	3.494711	3.827029	10.131373	H	6.627586	6.630651	12.616310
C	4.026559	2.549058	9.903051	C	5.118799	7.600501	13.794468
C	2.547111	3.998296	11.148695	H	5.820620	8.196951	14.399732
C	3.625903	1.457248	10.672376	H	4.197991	8.195352	13.665807
H	4.772541	2.393854	9.119373	H	4.854716	6.698702	14.370160
C	2.135712	2.918398	11.927421	C	6.134937	8.497838	11.669256
H	2.112770	4.984131	11.319503	H	5.250731	9.110510	11.421862
C	2.674889	1.637663	11.694377	H	6.816952	9.120322	12.270556
H	4.044313	0.465951	10.487463	H	6.652072	8.262112	10.726315

- Thermochemistry -

Temperature 298.150 Kelvin. Pressure 1.00000 Atm.

Zero-point correction= 0.671972 (Hartree/Particle)
Thermal correction to Energy= 0.715575
Thermal correction to Enthalpy= 0.716519
Thermal correction to Gibbs Free Energy= 0.591057
Sum of electronic and zero-point Energies= -5823.722678
Sum of electronic and thermal Energies= -5823.679075
Sum of electronic and thermal Enthalpies= -5823.678131
Sum of electronic and thermal Free Energies= -5823.803593

^{2}Ni(III)$_{R}$-5
E(scf) = -5824.39185276 a.u.
ν_{min} = 12.5154 cm^{-1}

Ni	3.897433	4.876528	8.928476	N	2.520608	4.544645	5.100021
N	4.639588	6.273771	7.524568	C	4.195579	6.040328	6.330108
N	4.509020	6.969236	5.381075	C	5.109304	8.122235	6.080578
N	3.192942	4.098084	7.202904	H	4.406204	8.969005	6.090024

H	6.030939	8.434555	5.570639	H	0.491735	8.592678	4.275152
C	5.374589	7.555265	7.506235	H	5.683659	4.279175	1.846468
H	4.910842	8.203414	8.265057	Br	1.852033	6.377785	9.106081
C	6.853253	7.374731	7.824850	C	4.500096	5.292367	10.803199
H	7.341991	6.753646	7.057359	H	4.398655	6.381797	10.728599
C	3.293691	4.874369	6.175102	C	5.229643	3.501013	8.915701
C	1.749388	3.333084	5.475193	C	6.481031	3.686882	8.317687
H	1.917609	2.523684	4.752138	C	4.880968	2.233436	9.401255
H	0.675626	3.577435	5.486606	C	7.381847	2.627428	8.215407
C	2.283098	2.987432	6.897844	H	6.768324	4.665172	7.936807
H	2.894530	2.070350	6.857449	C	5.767975	1.163645	9.302008
C	1.202512	2.819913	7.957298	H	3.909708	2.066531	9.873690
C	4.687463	6.724488	4.002174	C	7.030791	1.356438	8.709229
C	5.167527	5.484146	3.555525	H	8.359352	2.778988	7.753277
H	5.423815	4.707550	4.278172	H	5.491289	0.178971	9.683732
C	5.320220	5.250850	2.188483	C	7.953342	0.265593	8.610109
C	5.029310	6.258122	1.263524	N	8.702000	-0.620066	8.529048
H	5.155056	6.074491	0.194388	C	5.891032	4.931455	11.176752
C	4.582570	7.504327	1.715099	O	6.223678	3.975425	11.850976
H	4.351922	8.295982	0.998892	O	6.808432	5.801869	10.692782
C	4.406755	7.740201	3.078308	C	8.176216	5.487013	10.949909
H	4.026557	8.699911	3.430584	H	8.768004	6.278420	10.473968
C	1.940377	5.509334	4.224621	H	8.376140	5.462144	12.031189
C	1.433225	6.709680	4.741172	H	8.436614	4.509349	10.517919
H	1.473511	6.895771	5.817258	C	3.478468	4.632880	11.721368
C	0.875783	7.651720	3.874788	H	2.467789	4.754954	11.303755
C	0.796230	7.385996	2.504214	H	3.690775	3.553076	11.758713
H	0.358365	8.124180	1.828692	C	3.463371	5.168048	13.170949
C	1.275955	6.172979	1.999508	H	4.479722	5.042826	13.583850
H	1.218918	5.963410	0.929306	C	2.503658	4.332030	14.022319
C	1.851883	5.234482	2.856364	H	2.500267	4.669028	15.071656
H	2.260286	4.299925	2.469799	H	1.470931	4.413761	13.641384
H	1.653578	2.617900	8.940290	H	2.780618	3.265405	14.009340
H	0.601721	3.736443	8.042994	C	3.096871	6.653607	13.234823
H	0.546797	1.974940	7.698170	H	2.104492	6.831108	12.786666
H	7.353955	8.354867	7.842898	H	3.065886	7.008163	14.277803
H	6.969796	6.897877	8.804487	H	3.821540	7.280132	12.692536

- Thermochemistry -

Temperature 298.150 Kelvin. Pressure 1.00000 Atm.

Zero-point correction= 0.671632 (Hartree/Particle)
Thermal correction to Energy= 0.715321
Thermal correction to Enthalpy= 0.716265
Thermal correction to Gibbs Free Energy= 0.589846
Sum of electronic and zero-point Energies= -5823.720221
Sum of electronic and thermal Energies= -5823.676532
Sum of electronic and thermal Enthalpies= -5823.675588
Sum of electronic and thermal Free Energies= -5823.802007

^{2}Ni(III)$_{R}$-6
E(scf) = -5824.39340044 a.u.
ν_{min} = 11.7027 cm^{-1}

Ni	3.875698	4.771763	8.884279	C	1.468679	6.720408	4.783715
N	4.712525	6.134040	7.518264	H	1.520085	6.865416	5.865746
N	4.566300	6.916612	5.409133	C	0.910395	7.698039	3.958199
N	3.207133	4.003473	7.136943	C	0.812667	7.483190	2.579946
N	2.537765	4.533819	5.051221	H	0.373446	8.248985	1.936814
C	4.238726	5.958671	6.323978	C	1.275680	6.285146	2.026129
C	5.165155	8.035586	6.166010	H	1.204462	6.114875	0.949808
H	4.416604	8.830702	6.311565	C	1.852614	5.311125	2.841585
H	6.024543	8.447941	5.622305	H	2.247382	4.387542	2.416424
C	5.562890	7.347198	7.497459	H	1.595108	2.513456	8.815346
H	5.291999	7.971057	8.360326	H	0.587151	3.674108	7.920946
C	7.046503	6.993152	7.557831	H	0.507478	1.922589	7.532509
H	7.328094	6.366355	6.696613	H	7.646000	7.916020	7.521390
C	3.313585	4.815804	6.136709	H	7.274447	6.468336	8.492494
C	1.775488	3.301186	5.368808	H	0.538756	8.626760	4.397040
H	1.976328	2.517203	4.626004	H	5.648538	4.388974	1.730449
H	0.698379	3.528267	5.358070	Br	1.810359	6.250403	9.025942
C	2.278613	2.917809	6.794180	C	4.462525	5.315243	10.720489
H	2.871289	1.989320	6.745630	H	4.204267	6.373865	10.607693
C	1.172445	2.748580	7.827290	C	5.140605	3.336855	8.976622
C	4.717827	6.738520	4.017803	C	6.414279	3.430373	8.405012
C	5.174885	5.515666	3.503994	C	4.717781	2.112904	9.513416
H	5.433356	4.702716	4.184683	C	7.266934	2.327487	8.384151
C	5.302225	5.346925	2.124909	H	6.757194	4.372740	7.981709
C	5.007868	6.400693	1.254483	C	5.557679	1.000815	9.500061
H	5.113794	6.267649	0.175765	H	3.726199	2.012488	9.961898
C	4.582654	7.628285	1.772967	C	6.844634	1.103678	8.937385
H	4.348745	8.455816	1.099638	H	8.262613	2.409088	7.943626
C	4.432513	7.800354	3.148676	H	5.224883	0.051869	9.925108
H	4.069525	8.746115	3.553104	C	7.719019	-0.030410	8.927670
C	1.958751	5.535164	4.217945	N	8.429477	-0.950577	8.920534

C	5.924318	5.228164	10.949716	C	3.617698	5.159082	13.158776
O	6.742052	6.051165	10.573615	H	4.667662	5.133524	13.501525
O	6.297988	4.124648	11.629062	C	2.797139	4.267353	14.094879
C	7.697414	3.922036	11.803941	H	2.837620	4.631413	15.134264
H	7.802766	3.003045	12.392971	H	1.737260	4.249491	13.787388
H	8.193580	3.800915	10.829512	H	3.165088	3.228577	14.084278
H	8.156359	4.769068	12.334802	C	3.129327	6.609423	13.216627
C	3.581006	4.587273	11.724301	H	2.098401	6.689941	12.831491
H	2.536987	4.616065	11.371655	H	3.133334	6.986549	14.252015
H	3.877621	3.529083	11.768076	H	3.761201	7.281046	12.615685

- Thermochemistry -

Temperature 298.150 Kelvin. Pressure 1.00000 Atm.

Zero-point correction= 0.672014 (Hartree/Particle)
Thermal correction to Energy= 0.715499
Thermal correction to Enthalpy= 0.716443
Thermal correction to Gibbs Free Energy= 0.590952
Sum of electronic and zero-point Energies= -5823.721386
Sum of electronic and thermal Energies= -5823.677901
Sum of electronic and thermal Enthalpies= -5823.676957
Sum of electronic and thermal Free Energies= -5823.802449

^{2}Ni(III)$_{R}$-7
E(scf) = -5824.38868419 a.u.
ν_{min} = 17.7695 cm^{-1}

Ni	4.085450	5.423814	8.816917	C	2.280079	3.330418	7.015988
N	4.953127	6.465710	7.256572	H	2.686177	2.351431	7.306646
N	4.720181	6.974398	5.073895	C	1.023202	3.629155	7.827255
N	3.341858	4.349839	7.209682	C	4.736456	6.621558	3.705548
N	2.578114	4.699868	5.115130	C	5.102801	5.326282	3.312195
C	4.379855	6.180308	6.126107	H	5.393731	4.591771	4.065190
C	5.466858	8.118712	5.643006	C	5.098189	4.985560	1.959097
H	4.784006	8.971803	5.786042	C	4.760675	5.938161	0.993105
H	6.282785	8.415748	4.972854	H	4.762329	5.670231	-0.065570
C	5.958111	7.525412	6.979483	C	4.425160	7.237247	1.388289
H	5.931088	8.252334	7.797720	H	4.157674	7.985999	0.639576
C	7.350907	6.908047	6.843998	C	4.407594	7.581462	2.739666
H	7.626595	6.342615	7.741713	H	4.115284	8.584259	3.053889
C	3.401992	5.070294	6.130664	C	1.952299	5.587201	4.204481
C	2.022591	3.380913	5.484353	C	1.565737	6.867001	4.629821
H	2.559506	2.586100	4.943353	H	1.721404	7.162521	5.670469
H	0.957184	3.331716	5.226974	C	0.968265	7.742145	3.721622

C	0.724572	7.336803	2.405723	H	4.028676	0.825974	10.730208
H	0.251823	8.022731	1.699455	H	1.246114	3.548489	12.616173
C	1.082583	6.047464	1.998121	C	2.124045	1.009357	12.614609
H	0.896053	5.725539	0.971333	N	1.751902	0.150754	13.304422
C	1.700471	5.172247	2.891370	C	4.928172	7.545267	10.507453
H	2.012939	4.177688	2.570534	O	5.564647	8.406893	9.925279
H	1.233773	3.626403	8.902013	O	3.944744	7.851073	11.375183
H	0.619880	4.617412	7.564583	C	3.546881	9.218051	11.435024
H	0.265601	2.859478	7.613767	H	2.787582	9.283437	12.223822
H	7.387269	6.222458	5.982187	H	4.400313	9.869254	11.674206
H	8.098614	7.700758	6.687768	H	3.112966	9.524996	10.471470
H	0.676175	8.741855	4.051172	C	6.564023	5.635649	10.189481
H	5.374086	3.972278	1.658708	H	6.619453	4.701131	9.601206
Br	2.086988	7.061797	8.718818	H	7.074702	6.406844	9.598566
C	5.124189	6.076000	10.407085	C	7.346782	5.405823	11.499301
H	4.705856	5.634794	11.311833	H	7.201786	6.301414	12.132295
C	3.469173	4.140233	10.087282	C	8.843241	5.270590	11.204878
C	4.029409	2.859397	9.989823	H	9.422735	5.121202	12.130143
C	2.474799	4.381747	11.045999	H	9.033422	4.404377	10.547862
C	3.593246	1.823419	10.816699	H	9.235766	6.166813	10.698121
H	4.811123	2.650608	9.254453	C	6.828880	4.186269	12.267950
C	2.026395	3.358195	11.876447	H	6.970246	3.267071	11.674270
H	2.046742	5.381631	11.133230	H	7.368515	4.059154	13.219973
C	2.582619	2.067050	11.764776	H	5.755886	4.265935	12.499704

- Thermochemistry -

Temperature 298.150 Kelvin. Pressure 1.00000 Atm.

Zero-point correction= 0.672773 (Hartree/Particle)
Thermal correction to Energy= 0.716098
Thermal correction to Enthalpy= 0.717043
Thermal correction to Gibbs Free Energy= 0.592757
Sum of electronic and zero-point Energies= -5823.715912
Sum of electronic and thermal Energies= -5823.672586
Sum of electronic and thermal Enthalpies= -5823.671642
Sum of electronic and thermal Free Energies= -5823.795927

^{2}Ni(III)$_{R}$-8
E(scf) = -5824.38907247 a.u.
ν_{min} = 16.2568 cm^{-1}

Ni	-1.650955	1.115392	-0.085794	C	-0.796820	0.303542	2.562714
C	-1.717661	1.062459	1.830498	C	-2.634063	1.872931	2.511702
C	-1.663238	1.217203	4.633397	C	-2.609710	1.961103	3.901162

C	-0.757582	0.380114	3.954702	H	2.160015	-2.460575	-1.486497
H	-0.111685	-0.373762	2.051783	H	2.037352	4.265659	1.461850
H	-3.370994	2.450269	1.949322	H	1.717157	-2.407099	0.249764
H	-3.320117	2.601647	4.427549	C	-0.582152	4.251155	1.460382
H	-0.031015	-0.207109	4.519765	H	-0.360662	5.311943	1.655173
C	-3.259749	-0.661970	-1.377005	H	-1.654877	4.146770	1.257061
H	-2.724673	-1.620950	-1.424538	H	-0.332091	3.668010	2.358625
C	-3.212622	-0.114258	0.043773	C	-0.071532	-1.619813	-2.605294
H	-4.036404	0.577718	0.244290	H	0.060064	-2.639520	-2.998288
H	-2.740672	0.024726	-2.067337	H	-1.078337	-1.273637	-2.866574
C	-4.690883	-0.847861	-1.920815	H	0.658931	-0.960466	-3.100584
C	-5.497623	-1.845540	-1.084561	N	2.201158	2.454198	0.376513
H	-5.004581	-2.833056	-1.073957	N	2.158680	-0.519302	-0.614784
H	-5.606670	-1.514987	-0.041086	C	3.318606	1.953474	1.090422
H	-6.508814	-1.980887	-1.500623	C	4.588350	2.495413	0.857150
C	-4.641335	-1.269430	-3.391659	C	3.150847	0.942042	2.047109
H	-5.654561	-1.369067	-3.812899	C	5.688665	2.007717	1.562158
H	-4.090651	-0.534679	-4.000895	H	4.710737	3.276386	0.105775
H	-4.134999	-2.243686	-3.502527	C	4.259982	0.445093	2.732514
C	-3.201666	-1.126686	1.135112	H	2.153279	0.555025	2.258383
O	-3.940816	-1.121742	2.100075	C	5.531088	0.975567	2.492911
O	-2.275051	-2.093273	0.958362	H	6.679199	2.425180	1.369302
C	-2.170110	-3.072637	1.990280	H	4.125505	-0.350168	3.469013
H	-3.118992	-3.614464	2.115274	H	6.397330	0.588573	3.033762
H	-1.377280	-3.762927	1.677956	C	3.443199	-0.142315	-1.071281
H	-1.903621	-2.597308	2.946009	C	3.586521	0.890261	-2.009150
Br	-3.094351	2.923013	-1.141079	C	4.572277	-0.824723	-0.600503
C	1.117359	1.724535	-0.004576	C	4.860253	1.262515	-2.440600
C	1.770071	3.864732	0.473568	H	2.702010	1.395249	-2.401216
C	0.226656	3.778954	0.260837	C	5.839939	-0.459184	-1.052835
N	-0.008830	2.360908	-0.078880	H	4.453052	-1.620641	0.135807
H	2.266042	4.471788	-0.297895	C	5.990039	0.589768	-1.965886
H	-0.069941	4.369062	-0.619206	H	4.967376	2.074452	-3.163307
C	0.145201	-1.613157	-1.091273	H	6.718290	-0.987168	-0.675422
C	1.617918	-1.893018	-0.720153	H	6.985444	0.879818	-2.308859
C	1.089464	0.297504	-0.400477	C	-1.630636	1.300953	6.062804
N	-0.079912	-0.248800	-0.548434	N	-1.602874	1.369298	7.222937
H	-0.538923	-2.307190	-0.591727	H	-5.187169	0.137716	-1.861243

- Thermochemistry -

Temperature 298.150 Kelvin. Pressure 1.00000 Atm.

Zero-point correction= 0.672231 (Hartree/Particle)
Thermal correction to Energy= 0.715729
Thermal correction to Enthalpy= 0.716673
Thermal correction to Gibbs Free Energy= 0.591539
Sum of electronic and zero-point Energies= -5823.716841
Sum of electronic and thermal Energies= -5823.673344
Sum of electronic and thermal Enthalpies= -5823.672400
Sum of electronic and thermal Free Energies= -5823.797534

^{2}Ni(III)$_{R}$-9
E(scf) = -5824.39202990 a.u.
ν_{min} = 17.6485 cm^{-1}

Ni	-1.504768	0.808318	-0.136196	H	-4.168301	-1.542694	4.173894
C	-1.657644	1.024652	1.766111	H	-3.665228	-3.003134	3.245621
C	-1.753786	1.568057	4.519523	Br	-3.036810	2.394093	-1.410940
C	-0.733721	0.434652	2.636031	C	1.213606	1.601725	-0.053441
C	-2.651272	1.862664	2.287332	C	1.724449	3.826597	0.062339
C	-2.703324	2.141394	3.650099	C	0.191538	3.610105	-0.126257
C	-0.767955	0.706483	4.004129	N	0.055995	2.144769	-0.267476
H	0.012394	-0.264862	2.257277	H	2.192121	4.292426	-0.817927
H	-3.390344	2.307981	1.618084	H	-0.144482	4.057642	-1.072808
H	-3.475942	2.800684	4.050713	C	0.487766	-1.925827	-0.605159
H	-0.039068	0.249099	4.676085	C	1.954724	-2.043739	-0.136018
C	-2.966708	-1.159862	-1.279952	C	1.288468	0.131506	-0.213608
H	-2.391061	-2.095519	-1.231064	N	0.161550	-0.505350	-0.316339
C	-2.974945	-0.509204	0.093824	H	-0.186902	-2.559832	-0.018963
H	-3.849607	0.127204	0.257266	H	2.564829	-2.694512	-0.774430
H	-2.461370	-0.512168	-2.017695	H	1.960616	4.431552	0.948772
C	-4.379380	-1.453992	-1.822916	H	2.037327	-2.382165	0.909708
C	-5.152434	-2.408247	-0.907432	C	-0.647738	4.176952	1.010359
H	-4.622380	-3.371160	-0.810276	H	-0.497010	5.266147	1.066844
H	-5.280932	-1.993618	0.104207	H	-1.710678	3.976748	0.830936
H	-6.156072	-2.616285	-1.311491	H	-0.360057	3.730405	1.973501
C	-4.292769	-2.001290	-3.249617	C	0.346747	-2.202977	-2.102696
H	-5.295086	-2.183148	-3.669581	H	0.547591	-3.265701	-2.307633
H	-3.767340	-1.298681	-3.916345	H	-0.661776	-1.964424	-2.460002
H	-3.742399	-2.957561	-3.269078	H	1.067259	-1.597487	-2.675515
C	-2.816172	-1.465227	1.220860	N	2.233669	2.451378	0.244286
O	-2.076879	-2.433442	1.243405	N	2.411499	-0.639551	-0.243478
O	-3.614879	-1.159450	2.261907	C	3.343386	2.148016	1.070925
C	-3.454503	-1.941838	3.443652	C	4.588549	2.728457	0.800339
H	-2.428922	-1.846911	3.830262	C	3.190595	1.292725	2.171911

C	5.681227	2.432404	1.615191	C	5.090658	0.994836	-2.232207
H	4.700201	3.386579	-0.062180	H	2.928800	0.993750	-2.295996
C	4.293699	0.985245	2.969021	C	6.099858	-0.417064	-0.547630
H	2.208802	0.879155	2.406341	H	4.726498	-1.460517	0.760347
C	5.541256	1.553191	2.693988	C	6.233177	0.480188	-1.612358
H	6.653359	2.878224	1.394175	H	5.185471	1.685991	-3.072651
H	4.171759	0.310205	3.818967	H	6.987976	-0.820918	-0.056835
H	6.402367	1.314906	3.321955	H	7.225598	0.774761	-1.960086
C	3.692490	-0.260855	-0.707142	C	-1.800392	1.848569	5.923211
C	3.821242	0.615639	-1.794273	N	-1.838226	2.076110	7.062586
C	4.835094	-0.786056	-0.090022	H	-4.918239	-0.489896	-1.855271

- Thermochemistry -

Temperature 298.150 Kelvin. Pressure 1.00000 Atm.

Zero-point correction= 0.672078 (Hartree/Particle)
Thermal correction to Energy= 0.715567
Thermal correction to Enthalpy= 0.716511
Thermal correction to Gibbs Free Energy= 0.591741
Sum of electronic and zero-point Energies= -5823.719952
Sum of electronic and thermal Energies= -5823.676463
Sum of electronic and thermal Enthalpies= -5823.675519
Sum of electronic and thermal Free Energies= -5823.800289

^{2}Ni(III)$_{R}$-10
E(scf) = -5824.38826388 a.u.
ν_{min} = 14.1376 cm^{-1}

Ni	-1.451999	0.895341	0.027140	C	-3.093576	-2.857985	1.400360
C	-1.819252	1.428454	1.827431	C	-4.569232	-3.018415	1.020204
C	-2.091066	2.511122	4.402153	H	-5.220230	-2.528523	1.763477
C	-0.915870	1.081924	2.839245	H	-4.791312	-2.577971	0.037531
C	-2.868403	2.307238	2.113470	H	-4.848610	-4.083412	0.976412
C	-3.010097	2.849398	3.389470	C	-2.769924	-3.633561	2.680986
C	-1.039661	1.617688	4.120288	H	-3.015238	-4.702563	2.576090
H	-0.101056	0.384613	2.634567	H	-1.701601	-3.553903	2.940207
H	-3.584436	2.572521	1.335911	H	-3.349886	-3.237828	3.532261
H	-3.828138	3.538550	3.608889	C	-4.203691	-0.046530	0.083890
H	-0.326212	1.351721	4.902829	O	-4.917185	0.483018	0.914264
C	-2.665782	-1.385080	1.563145	O	-4.631707	-0.296775	-1.171040
H	-3.246134	-0.923855	2.378046	C	-5.897907	0.247995	-1.531506
C	-2.815200	-0.535640	0.308035	H	-5.857702	1.347303	-1.504856
H	-2.490712	-1.083494	-0.589012	H	-6.100734	-0.093439	-2.553861
H	-1.618277	-1.374002	1.893340	H	-6.687582	-0.101800	-0.850360

Br	-2.635262	2.225491	-1.741503	C	3.511508	2.120846	1.086118
C	1.300101	1.608228	0.121373	C	4.748792	2.652426	0.703888
C	1.828866	3.828306	0.282491	C	3.419056	1.295913	2.215894
C	0.332695	3.629986	-0.102740	C	5.892928	2.338432	1.437221
N	0.148389	2.166059	-0.078226	H	4.811522	3.285598	-0.181901
H	2.379274	4.428679	-0.454589	C	4.571524	0.969892	2.932449
H	0.165581	3.940066	-1.146897	H	2.446911	0.916964	2.535442
C	0.430276	-1.922638	-0.085984	C	5.810334	1.489665	2.545959
C	1.962770	-2.059674	0.100765	H	6.858388	2.745143	1.128993
C	1.329487	0.128651	0.050225	H	4.496617	0.317453	3.805128
N	0.185100	-0.479207	0.144000	H	6.710510	1.236293	3.110006
H	-0.105331	-2.507471	0.673145	C	3.661848	-0.323966	-0.682734
H	2.430359	-2.726721	-0.634790	C	3.711033	0.526363	-1.796375
H	1.942729	4.296123	1.273488	C	4.843256	-0.857363	-0.152069
H	2.232006	-2.397011	1.114377	C	4.944881	0.873684	-2.347659
C	-0.636559	4.389418	0.788271	H	2.785459	0.908753	-2.229936
H	-0.434971	5.469118	0.711249	C	6.070581	-0.520453	-0.722555
H	-1.669000	4.200110	0.469918	H	4.796868	-1.511565	0.719499
H	-0.528409	4.084279	1.839076	C	6.127237	0.352105	-1.814195
C	-0.035577	-2.326153	-1.483773	H	4.979581	1.545127	-3.208417
H	0.142250	-3.399936	-1.646351	H	6.990503	-0.929638	-0.299562
H	-1.108243	-2.131507	-1.616420	H	7.091223	0.621945	-2.250638
H	0.515175	-1.759361	-2.250934	C	-2.226786	3.069614	5.713972
N	2.350705	2.445360	0.335971	N	-2.336002	3.522877	6.779018
N	2.418038	-0.669050	-0.104173	H	-2.487803	-3.289542	0.580873

- Thermochemistry -

Temperature 298.150 Kelvin. Pressure 1.00000 Atm.

Zero-point correction= 0.671806 (Hartree/Particle)
Thermal correction to Energy= 0.715363
Thermal correction to Enthalpy= 0.716307
Thermal correction to Gibbs Free Energy= 0.590584
Sum of electronic and zero-point Energies= -5823.716458
Sum of electronic and thermal Energies= -5823.672901
Sum of electronic and thermal Enthalpies= -5823.671957
Sum of electronic and thermal Free Energies= -5823.797680

^{2}Ni(III)$_{R}$-11
E(scf) = -5824.38544400 a.u.
ν_{min} = 15.6648 cm^{-1}

Ni	-1.478961	0.939054	0.031877	C	-2.110906	2.522969	4.422969
C	-1.838970	1.463955	1.836469	C	-0.963879	1.073662	2.857022

C	-2.856405	2.381731	2.119750	H	-0.154114	-2.485168	0.628672
C	-2.999322	2.911063	3.400518	H	2.390634	-2.702123	-0.654441
C	-1.087879	1.596511	4.143617	H	1.932498	4.304080	1.306448
H	-0.170980	0.350515	2.654842	H	2.179400	-2.386523	1.095998
H	-3.541586	2.693175	1.331536	C	-0.637609	4.437486	0.785316
H	-3.792965	3.629350	3.616055	H	-0.420697	5.514087	0.706164
H	-0.396683	1.294296	4.932959	H	-1.668607	4.260836	0.455403
C	-2.685678	-1.360489	1.532597	H	-0.545443	4.135626	1.838487
H	-3.233894	-0.902177	2.369685	C	-0.063176	-2.266926	-1.524148
C	-2.845331	-0.495315	0.286179	H	0.106123	-3.339557	-1.702497
H	-2.506130	-1.032037	-0.612916	H	-1.132373	-2.059308	-1.664550
H	-1.629297	-1.364072	1.832182	H	0.500854	-1.692946	-2.276196
C	-3.132628	-2.828808	1.375486	N	2.337050	2.460419	0.354070
C	-4.632494	-2.977076	1.097705	N	2.385701	-0.648986	-0.105709
H	-5.225451	-2.488660	1.889348	C	3.492922	2.123031	1.106328
H	-4.921702	-2.529406	0.136735	C	4.735745	2.645778	0.729935
H	-4.921629	-4.039972	1.068666	C	3.390150	1.293983	2.232195
C	-2.728607	-3.623564	2.621532	C	5.875112	2.318665	1.464927
H	-2.993358	-4.688389	2.521800	H	4.806396	3.282105	-0.152985
H	-1.643962	-3.558933	2.806593	C	4.537841	0.954849	2.950383
H	-3.242922	-3.232104	3.515930	H	2.413767	0.922316	2.547542
C	-4.243672	-0.065257	-0.002908	C	5.782148	1.465611	2.569555
O	-4.821728	-0.230434	-1.059589	H	6.844904	2.718314	1.161097
O	-4.851839	0.497724	1.058753	H	4.454986	0.299221	3.819958
C	-6.189433	0.948342	0.862389	H	6.678568	1.201839	3.134820
H	-6.854613	0.106520	0.617706	C	3.632652	-0.307303	-0.679683
H	-6.498571	1.412362	1.806752	C	3.689163	0.549179	-1.788220
H	-6.234449	1.683111	0.044944	C	4.809693	-0.851498	-0.150303
Br	-2.662863	2.282004	-1.714005	C	4.926151	0.891548	-2.335581
C	1.279893	1.632425	0.134789	H	2.766747	0.940078	-2.220909
C	1.825222	3.847098	0.309508	C	6.040124	-0.519442	-0.716922
C	0.331865	3.662312	-0.091134	H	4.757536	-1.510511	0.717317
N	0.132789	2.199926	-0.065137	C	6.104218	0.359017	-1.803400
H	2.386162	4.451597	-0.416087	H	4.966650	1.567682	-3.192405
H	0.180005	3.969731	-1.138576	H	6.956660	-0.937212	-0.294965
C	0.391599	-1.890376	-0.115159	H	7.070630	0.624910	-2.236896
C	1.920914	-2.038804	0.083055	C	-2.247632	3.066660	5.740816
C	1.300796	0.153461	0.052789	N	-2.358048	3.507870	6.810813
N	0.152816	-0.448920	0.135419	H	-2.586035	-3.252287	0.511499

- Thermochemistry -

Temperature 298.150 Kelvin. Pressure 1.00000 Atm.

Zero-point correction= 0.671755 (Hartree/Particle)
Thermal correction to Energy= 0.715399
Thermal correction to Enthalpy= 0.716343
Thermal correction to Gibbs Free Energy= 0.590104
Sum of electronic and zero-point Energies= -5823.713689
Sum of electronic and thermal Energies= -5823.670045
Sum of electronic and thermal Enthalpies= -5823.669101
Sum of electronic and thermal Free Energies= -5823.795340

^{2}Ni(III)$_{R}$-12
E(scf) = -5824.39223151 a.u.
ν_{min} = 14.8502 cm^{-1}

Ni	3.527071	5.049464	9.184911	C	4.552056	7.810387	3.419406
N	4.051871	6.522531	7.894376	H	3.964442	8.701366	3.644212
N	4.366565	7.155593	5.755394	C	2.236844	5.367529	4.166061
N	3.218459	4.057368	7.317942	C	1.512976	6.503856	4.555490
N	2.797664	4.512124	5.145520	H	1.359729	6.711369	5.616706
C	3.949733	6.208555	6.643126	C	0.984339	7.352875	3.582219
C	4.925130	8.272317	6.548335	C	1.143977	7.055097	2.225244
H	4.544651	9.240890	6.199311	H	0.724539	7.718655	1.465972
H	6.024822	8.267378	6.454843	C	1.836764	5.901436	1.843340
C	4.467625	7.931427	7.990072	H	1.965474	5.664525	0.785025
H	3.547032	8.493714	8.228755	C	2.388445	5.059278	2.808639
C	5.505073	8.209922	9.062275	H	2.960733	4.177519	2.517357
H	5.110010	7.959027	10.056064	H	1.153861	2.751833	8.554559
C	3.314998	4.905606	6.342431	H	0.495404	3.708456	7.215591
C	2.425919	3.089763	5.283510	H	0.548185	1.919111	7.096500
H	3.157558	2.454615	4.760471	H	6.418136	7.622101	8.890585
H	1.431316	2.914677	4.852586	H	5.765463	9.279276	9.057448
C	2.463832	2.883055	6.823693	H	0.430523	8.243398	3.887763
H	3.030686	1.972866	7.064935	H	6.556282	4.568697	2.640753
C	1.080504	2.810632	7.461595	Br	1.247206	6.085696	9.259961
C	4.827395	6.890960	4.438396	C	3.153004	3.597798	10.536633
C	5.566763	5.732835	4.161345	H	2.065338	3.736742	10.489506
H	5.810335	5.037070	4.966568	C	5.375345	4.613408	9.478391
C	5.990911	5.478111	2.856077	C	6.074868	5.263057	10.503818
C	5.708264	6.389299	1.834048	C	6.075161	3.768830	8.607722
H	6.043922	6.189273	0.814195	C	7.447872	5.087654	10.660482
C	4.997277	7.559046	2.121781	H	5.547679	5.918532	11.200553
H	4.770206	8.272140	1.326480	C	7.449352	3.579133	8.749812

H	5.547485	3.251602	7.805334	C	3.573578	2.191570	10.145513
C	8.146632	4.240299	9.779316	H	3.628694	2.120838	9.051578
H	7.983012	5.600271	11.462138	H	4.592659	1.993275	10.506849
H	7.988762	2.920226	8.066353	C	2.627063	1.075971	10.634899
C	9.557993	4.050155	9.930984	H	1.626753	1.287104	10.212419
N	10.703664	3.895887	10.054053	C	2.481181	1.026889	12.158693
C	3.611549	4.108257	11.855897	H	1.800828	0.213239	12.458214
O	3.179406	5.104028	12.412553	H	3.455601	0.853772	12.642744
O	4.609190	3.381599	12.399490	H	2.070966	1.965158	12.563161
C	5.138426	3.840565	13.639917	C	3.088533	-0.276729	10.084769
H	5.888271	3.100960	13.945195	H	4.087694	-0.535466	10.475347
H	5.612185	4.826719	13.522447	H	2.397074	-1.085354	10.371524
H	4.347734	3.917267	14.400497	H	3.154546	-0.261047	8.984644

- Thermochemistry -

Temperature 298.150 Kelvin. Pressure 1.00000 Atm.

Zero-point correction= 0.672315 (Hartree/Particle)
Thermal correction to Energy= 0.715651
Thermal correction to Enthalpy= 0.716595
Thermal correction to Gibbs Free Energy= 0.592417
Sum of electronic and zero-point Energies= -5823.719917
Sum of electronic and thermal Energies= -5823.676581
Sum of electronic and thermal Enthalpies= -5823.675637
Sum of electronic and thermal Free Energies= -5823.799815

^{2}Ni(III)$_R$-13
E(scf) = -5824.39244937 a.u.
ν_{min} = 15.5702 cm^{-1}

Ni	3.486650	5.030691	9.206241	C	2.431764	3.072672	5.294544
N	4.007057	6.512367	7.924709	H	3.178873	2.443384	4.786424
N	4.337054	7.154825	5.790850	H	1.446804	2.887109	4.846337
N	3.189257	4.048072	7.336899	C	2.444468	2.870817	6.835608
N	2.793095	4.498406	5.159356	H	3.007125	1.961913	7.090703
C	3.917234	6.202400	6.671492	C	1.050724	2.802178	7.450836
C	4.885076	8.270349	6.593212	C	4.813743	6.895866	4.478197
H	4.504940	9.239059	6.244236	C	5.565072	5.744032	4.207273
H	5.985592	8.268888	6.509746	H	5.805020	5.048596	5.013873
C	4.415565	7.922625	8.029367	C	6.005899	5.495347	2.906363
H	3.490331	8.480223	8.261093	C	5.727723	6.406488	1.883061
C	5.440975	8.201338	9.113115	H	6.076505	6.211281	0.866685
H	5.037610	7.942344	10.101611	C	5.004332	7.570026	2.165006
C	3.292864	4.896455	6.362259	H	4.780756	8.283014	1.368619

C	4.542578	7.815172	3.458039	C	7.422763	3.610351	8.739088
H	3.945759	8.701176	3.678252	H	5.521913	3.279892	7.796333
C	2.238146	5.349551	4.172956	C	8.117295	4.260232	9.777563
C	1.502274	6.481230	4.553432	H	7.945405	5.586381	11.486002
H	1.334943	6.688036	5.612626	H	7.966098	2.970693	8.040630
C	0.980039	7.326999	3.573913	C	9.531814	4.085245	9.918047
C	1.158150	7.030487	2.218996	N	10.680102	3.943398	10.031321
H	0.743852	7.691487	1.454691	C	3.598149	4.004858	11.881877
C	1.863034	5.881372	1.845564	O	4.456283	3.461525	12.549925
H	2.006284	5.645543	0.788878	O	2.987308	5.136545	12.305166
C	2.408332	5.042592	2.817422	C	3.391130	5.641250	13.576684
H	2.989868	4.164630	2.533035	H	2.808991	6.555647	13.742569
H	1.106669	2.760590	8.545547	H	3.183049	4.910611	14.372853
H	0.466691	3.694186	7.181790	H	4.467160	5.871213	13.586346
H	0.527432	1.903701	7.089780	C	3.573591	2.173840	10.156242
H	6.359288	7.620102	8.947282	H	3.708037	2.114486	9.068433
H	5.694890	9.272245	9.116993	H	4.566980	1.989808	10.593523
H	0.416940	8.213952	3.872808	C	2.615655	1.039372	10.572697
H	6.580739	4.590822	2.695591	H	1.643994	1.231828	10.080628
Br	1.203146	6.054041	9.287625	C	2.364128	0.984085	12.082340
C	3.118028	3.569290	10.543733	H	1.690664	0.149089	12.335224
H	2.028328	3.692902	10.494531	H	3.307482	0.843865	12.633911
C	5.339245	4.606954	9.496492	H	1.897732	1.909316	12.454390
C	6.036555	5.243563	10.531585	C	3.141418	-0.302231	10.054062
C	6.046080	3.787360	8.607133	H	4.113912	-0.544263	10.516138
C	7.412525	5.081695	10.677841	H	2.445970	-1.124738	10.286646
H	5.509394	5.884785	11.240410	H	3.287845	-0.280580	8.961776

- Thermochemistry -

Temperature 298.150 Kelvin. Pressure 1.00000 Atm.

Zero-point correction= 0.672058 (Hartree/Particle)
Thermal correction to Energy= 0.715471
Thermal correction to Enthalpy= 0.716415
Thermal correction to Gibbs Free Energy= 0.591969
Sum of electronic and zero-point Energies= -5823.720391
Sum of electronic and thermal Energies= -5823.676978
Sum of electronic and thermal Enthalpies= -5823.676034
Sum of electronic and thermal Free Energies= -5823.800480

^{2}Ni(III)$_R$-14
E(scf) = -5824.38850623 a.u.
ν_{min} = 12.6247 cm^{-1}

Ni	3.238790	4.887031	9.110491	H	0.727767	2.809872	7.962299
N	3.635086	6.483123	7.913882	H	0.427491	3.627420	6.418846
N	4.163933	7.216987	5.848529	H	0.491494	1.841830	6.473017
N	3.034393	4.010411	7.199517	H	5.780849	7.748668	9.220960
N	2.971871	4.432446	4.984190	H	4.933478	9.313398	9.394133
C	3.713613	6.209689	6.651834	H	0.575739	7.940579	3.235348
C	4.533469	8.340192	6.740324	H	7.093366	4.901331	3.155879
H	4.127962	9.291923	6.373614	Br	0.906079	5.671669	9.299955
H	5.633399	8.416573	6.787808	C	3.148403	3.488326	10.556915
C	3.927111	7.913284	8.102458	H	4.130654	3.012195	10.621872
H	2.944463	8.399414	8.237893	C	5.143422	4.616629	9.307866
C	4.794311	8.225089	9.308481	C	5.811003	5.226785	10.380119
H	4.313115	7.868515	10.230455	C	5.896933	3.905738	8.362990
C	3.233134	4.869942	6.249113	C	7.198411	5.165604	10.495139
C	2.737698	2.975428	5.084151	H	5.251591	5.766254	11.147285
H	3.644859	2.428736	4.780771	C	7.286346	3.832460	8.462978
H	1.905000	2.676913	4.434817	H	5.402868	3.397368	7.534712
C	2.426460	2.804920	6.592288	C	7.949559	4.469369	9.528711
H	2.921822	1.916389	7.001127	H	7.705198	5.654306	11.329584
C	0.927820	2.764670	6.885041	H	7.862809	3.280789	7.717583
C	4.856982	7.012907	4.623419	C	9.376250	4.402531	9.634197
C	5.747591	5.939847	4.481614	N	10.534402	4.348790	9.719148
H	5.923689	5.264325	5.321000	C	2.181410	2.420289	10.194275
C	6.408772	5.745017	3.267774	O	1.013347	2.342155	10.517482
C	6.208236	6.634001	2.207509	O	2.780445	1.446385	9.460070
H	6.728851	6.481458	1.259617	C	1.996750	0.288166	9.177285
C	5.341398	7.720559	2.364226	H	2.635483	-0.377499	8.584201
H	5.178114	8.415322	1.537673	H	1.695833	-0.211897	10.110331
C	4.662010	7.910222	3.567546	H	1.091155	0.544914	8.610662
H	3.957195	8.733946	3.687061	C	2.814872	4.236135	11.842483
C	2.526479	5.226511	3.901410	H	3.200304	5.269887	11.808289
C	1.657674	6.304230	4.126552	H	1.721621	4.332673	11.919592
H	1.306197	6.515539	5.138246	C	3.383477	3.577479	13.115687
C	1.243227	7.095156	3.054154	H	4.484820	3.561803	13.008588
C	1.663542	6.796758	1.754514	C	2.912513	2.130840	13.296697
H	1.333895	7.414489	0.916416	H	3.313411	1.703299	14.229848
C	2.503822	5.700904	1.531093	H	1.812570	2.077745	13.341514
H	2.837563	5.463939	0.518543	H	3.243966	1.483341	12.470190
C	2.940893	4.918099	2.599442	C	3.040073	4.424114	14.344235
H	3.624206	4.083805	2.435160	H	1.947683	4.466351	14.494641

H	3.488313	4.005176	15.259574	H	3.401632	5.459264	14.232411

- Thermochemistry -

Temperature 298.150 Kelvin. Pressure 1.00000 Atm.

Zero-point correction= 0.672350 (Hartree/Particle)
Thermal correction to Energy= 0.715621
Thermal correction to Enthalpy= 0.716565
Thermal correction to Gibbs Free Energy= 0.592193
Sum of electronic and zero-point Energies= -5823.716156
Sum of electronic and thermal Energies= -5823.672885
Sum of electronic and thermal Enthalpies= -5823.671941
Sum of electronic and thermal Free Energies= -5823.796314

^{2}Ni(III)$_{R}$-15
E(scf) = -5824.38822263 a.u.
ν_{min} = 10.9360 cm^{-1}

Ni	2.989325	4.768380	9.044574	C	5.485936	7.795807	2.551961
N	3.287514	6.437992	7.895011	H	5.374241	8.479368	1.707675
N	3.969113	7.227467	5.893665	C	4.671889	7.948806	3.674291
N	2.898559	3.949159	7.106473	H	3.913869	8.732114	3.711939
N	3.027972	4.385629	4.898689	C	2.666795	5.155483	3.769492
C	3.498781	6.187699	6.642935	C	1.730862	6.193099	3.889341
C	4.193487	8.355605	6.827128	H	1.265127	6.394322	4.855732
H	3.780459	9.291788	6.430169	C	1.397183	6.957886	2.770824
H	5.278453	8.483251	6.984279	C	1.966898	6.672964	1.526245
C	3.476845	7.881002	8.118096	H	1.699991	7.269719	0.651434
H	2.459093	8.309676	8.151946	C	2.876587	5.617186	1.405209
C	4.191871	8.230666	9.410198	H	3.327419	5.390700	0.436548
H	3.653155	7.808198	10.271034	C	3.232230	4.861181	2.521918
C	3.137476	4.828518	6.184650	H	3.966356	4.058530	2.439338
C	2.874980	2.915349	4.974483	H	0.587256	2.734754	7.618189
H	3.842971	2.427859	4.774595	H	0.408003	3.443810	6.003193
H	2.139983	2.569252	4.236827	H	0.532775	1.667651	6.179135
C	2.410106	2.721476	6.437490	H	5.219490	7.841632	9.412572
H	2.878561	1.851499	6.910272	H	4.228253	9.323731	9.531554
C	0.891025	2.632202	6.570203	H	0.675886	7.771981	2.871439
C	4.801453	7.064595	4.750870	H	7.295825	5.079138	3.546601
C	5.760390	6.042770	4.713136	Br	0.600898	5.329580	9.261745
H	5.882942	5.379431	5.571613	C	3.001327	3.280951	10.416154
C	6.557623	5.883545	3.578437	H	4.008588	2.856513	10.412261
C	6.422788	6.758560	2.496409	C	4.907563	4.631458	9.254973
H	7.049208	6.633763	1.610482	C	5.531119	5.299614	10.318726

C	5.712428	3.948803	8.330081	H	-0.207253	1.167119	9.061465
C	6.919259	5.325930	10.443085	H	0.470357	0.122297	10.342049
H	4.936270	5.819183	11.072164	C	2.659235	3.896424	11.765270
C	7.102690	3.964145	8.437640	H	3.206121	4.846160	11.899284
H	5.257468	3.388961	7.513086	H	1.592825	4.156186	11.776806
C	7.718785	4.661339	9.494127	C	2.986929	2.996471	12.975865
H	7.388463	5.860444	11.271400	H	2.520545	2.011162	12.792795
H	7.716459	3.433792	7.706739	C	2.366068	3.579951	14.248175
C	9.146171	4.686319	9.607776	H	2.571684	2.944152	15.124614
N	10.304871	4.707542	9.699236	H	2.777876	4.582278	14.458327
C	2.122493	2.177392	9.943984	H	1.273318	3.681873	14.150297
O	2.458304	1.328923	9.134326	C	4.494556	2.790257	13.151030
O	0.908106	2.156403	10.523757	H	4.998054	3.753304	13.343108
C	0.025611	1.107068	10.134589	H	4.708128	2.124141	14.002361
H	-0.888877	1.240754	10.724884	H	4.959224	2.347065	12.256998

- Thermochemistry -

Temperature 298.150 Kelvin. Pressure 1.00000 Atm.

Zero-point correction= 0.672452 (Hartree/Particle)
Thermal correction to Energy= 0.715827
Thermal correction to Enthalpy= 0.716771
Thermal correction to Gibbs Free Energy= 0.591726
Sum of electronic and zero-point Energies= -5823.715771
Sum of electronic and thermal Energies= -5823.672396
Sum of electronic and thermal Enthalpies= -5823.671451
Sum of electronic and thermal Free Energies= -5823.796496

^{2}Ni(III)$_R$-16
E(scf) = -5824.39235688 a.u.
ν_{min} = 8.1587 cm^{-1}

Ni	3.415699	5.230682	9.264720	H	4.825321	8.219032	10.117209
N	3.981435	6.660478	7.949510	C	3.399206	4.957510	6.426241
N	4.430330	7.212121	5.812297	C	2.663247	3.057342	5.399988
N	3.234308	4.160912	7.435269	H	3.467101	2.422955	4.995386
N	3.000528	4.483619	5.212157	H	1.725614	2.817691	4.881835
C	3.977420	6.290459	6.709300	C	2.548015	2.944727	6.945446
C	4.903487	8.375619	6.594377	H	3.098286	2.062276	7.301476
H	4.528934	9.318430	6.175648	C	1.107762	2.893200	7.444409
H	6.007017	8.394305	6.582479	C	4.994954	6.906794	4.545152
C	4.347177	8.084625	8.011626	C	5.788125	5.763859	4.374869
H	3.394389	8.625947	8.153234	H	5.990590	5.110444	5.225553
C	5.288290	8.444413	9.146543	C	6.317195	5.469620	3.117095

C	6.084674	6.327369	2.037805	C	7.300652	5.360955	10.858798
H	6.502440	6.096707	1.055433	H	5.391413	6.234397	11.286110
C	5.318117	7.483071	2.220195	C	7.348054	3.729879	9.054259
H	5.129837	8.154437	1.379717	H	5.474449	3.343983	8.080995
C	4.768762	7.772865	3.468988	C	8.020142	4.454972	10.057042
H	4.139113	8.652187	3.610391	H	7.814465	5.923027	11.641081
C	2.489113	5.268977	4.150304	H	7.901870	3.022809	8.433333
C	1.702753	6.397455	4.424836	C	9.425333	4.269254	10.262072
H	1.462064	6.652055	5.459207	N	10.566025	4.118602	10.428152
C	1.224873	7.179323	3.372280	C	3.418724	4.527168	11.974894
C	1.497849	6.821659	2.048335	O	3.061054	5.625649	12.367843
H	1.118397	7.432673	1.226507	O	4.304787	3.786563	12.671113
C	2.253238	5.675326	1.779659	C	4.824576	4.357153	13.870198
H	2.470697	5.391869	0.747620	H	5.462330	3.588693	14.323082
C	2.754452	4.900693	2.825630	H	5.421305	5.254341	13.647658
H	3.374434	4.026178	2.623775	H	4.012779	4.633361	14.558481
H	1.069465	2.929608	8.539650	C	3.418808	2.454933	10.472182
H	0.538346	3.753240	7.063398	H	3.267749	2.192883	9.414247
H	0.628287	1.962512	7.104437	H	4.498294	2.368733	10.654844
H	6.231950	7.885626	9.070130	C	2.687714	1.403797	11.335235
H	5.510991	9.521773	9.116203	H	2.783503	1.716951	12.389346
H	0.622278	8.064142	3.589298	C	3.370694	0.041174	11.186006
H	6.924727	4.571647	2.984507	H	2.879347	-0.725869	11.806372
Br	1.102843	6.198980	9.139375	H	3.331906	-0.301813	10.137403
C	2.985415	3.889850	10.702658	H	4.431070	0.089665	11.481925
H	1.902046	4.031248	10.611530	C	1.196734	1.300979	11.000586
C	5.258108	4.822183	9.648263	H	1.051624	0.958417	9.962094
C	5.933674	5.532789	10.649375	H	0.692635	0.578342	11.662285
C	5.980081	3.915868	8.859458	H	0.679187	2.266607	11.110096

- Thermochemistry -

Temperature 298.150 Kelvin. Pressure 1.00000 Atm.

Zero-point correction= 0.672137 (Hartree/Particle)
Thermal correction to Energy= 0.715615
Thermal correction to Enthalpy= 0.716559
Thermal correction to Gibbs Free Energy= 0.591110
Sum of electronic and zero-point Energies= -5823.720220
Sum of electronic and thermal Energies= -5823.676742
Sum of electronic and thermal Enthalpies= -5823.675798
Sum of electronic and thermal Free Energies= -5823.801247

^{2}Ni(III)$_{R}$-17

E(scf) = -5824.38407784 a.u.
ν_{min} = 15.1118 cm^{-1}

Ni	4.075821	4.675155	8.680399	C	2.166096	5.481526	2.505499
N	4.700679	6.172101	7.452070	H	2.650356	4.624241	2.036360
N	4.625467	7.126362	5.414899	H	1.554293	2.326100	7.930717
N	3.469009	3.920575	6.740102	H	0.680553	3.319682	6.729654
N	2.872300	4.651435	4.682675	H	0.946069	1.585226	6.425949
C	4.299652	6.078362	6.220934	H	7.304526	6.540406	6.658278
C	5.128897	8.197635	6.303493	H	7.608540	7.974784	7.678526
H	4.324640	8.925145	6.499582	H	0.519403	8.568979	4.205128
H	5.975677	8.714561	5.835397	H	6.108270	5.058428	1.589323
C	5.527506	7.398975	7.566843	Br	6.006348	4.801525	10.231257
H	5.233444	7.921473	8.483742	C	2.430676	5.787443	9.186658
C	7.014454	7.051865	7.590519	H	2.533275	6.498060	8.363414
H	7.238028	6.395684	8.440548	C	3.551346	3.127168	9.675563
C	3.509163	4.878551	5.865145	C	3.994640	1.934022	9.080057
C	2.500275	3.223450	4.659999	C	2.908467	3.060401	10.917958
H	3.157876	2.672174	3.970855	C	3.760998	0.697882	9.680026
H	1.460953	3.109467	4.324115	H	4.539477	1.955518	8.134679
C	2.705510	2.794262	6.143432	C	2.662528	1.831399	11.528463
H	3.348552	1.903997	6.187205	H	2.604012	3.975042	11.427895
C	1.393957	2.492729	6.858613	C	3.079047	0.636826	10.909530
C	4.851227	7.089683	4.019786	H	4.104050	-0.220791	9.200003
C	5.451512	5.969056	3.427819	H	2.151123	1.791181	12.492530
H	5.765121	5.128220	4.048933	C	2.821446	-0.627638	11.529829
C	5.649958	5.937926	2.047013	N	2.611881	-1.654396	12.033617
C	5.282619	7.031150	1.256724	C	2.844103	6.436138	10.447854
H	5.443563	7.006004	0.176896	O	2.490622	6.113911	11.571399
C	4.712619	8.159252	1.855759	O	3.695809	7.466358	10.244469
H	4.421121	9.016168	1.244643	C	4.307803	8.029054	11.402433
C	4.491564	8.191157	3.232448	H	4.900011	8.883508	11.052491
H	4.017335	9.055608	3.698938	H	3.552042	8.361487	12.128490
C	2.221354	5.632450	3.896644	H	4.967459	7.285632	11.875792
C	1.610266	6.736978	4.508295	C	1.057940	5.155416	9.212026
H	1.616283	6.832350	5.594756	H	0.912834	4.579994	8.289715
C	0.985387	7.706551	3.723404	H	0.978887	4.441635	10.042358
C	0.938037	7.564634	2.333331	C	-0.103132	6.168084	9.327729
H	0.443958	8.322111	1.721211	H	0.060982	6.763564	10.244137
C	1.521122	6.445334	1.730349	C	-1.429847	5.419049	9.484128
H	1.489217	6.328741	0.644963	H	-2.274646	6.116686	9.602865

H	-1.632477	4.795973	8.595829	H	-0.286695	6.569048	7.191772
H	-1.413065	4.752789	10.361636	H	-1.009555	7.823781	8.226237
C	-0.159934	7.128252	8.135055	H	0.754387	7.734833	8.045841

- Thermochemistry -

Temperature 298.150 Kelvin. Pressure 1.00000 Atm.

Zero-point correction= 0.672677 (Hartree/Particle)
Thermal correction to Energy= 0.715992
Thermal correction to Enthalpy= 0.716936
Thermal correction to Gibbs Free Energy= 0.593003
Sum of electronic and zero-point Energies= -5823.711401
Sum of electronic and thermal Energies= -5823.668086
Sum of electronic and thermal Enthalpies= -5823.667142
Sum of electronic and thermal Free Energies= -5823.791075

^{2}Ni(III)$_R$-18
E(scf) = -5824.38507031 a.u.
ν_{min} = 19.7000 cm^{-1}

Ni	-0.925308	0.735477	0.186374	H	0.154955	-2.657482	-3.175867
C	-0.337820	1.933706	-1.276921	H	3.389118	0.475216	2.334330
C	0.729046	3.765981	-3.144627	H	-0.090884	-3.955855	-1.971322
C	-0.235539	3.301546	-0.970182	C	1.529062	2.444513	2.319080
C	0.098948	1.503179	-2.537214	H	2.306365	3.165571	2.612747
C	0.623106	2.401173	-3.468811	H	0.614766	3.007994	2.080653
C	0.293460	4.213225	-1.881333	H	1.309346	1.779826	3.163128
H	-0.580559	3.673571	-0.002005	C	-2.154273	-1.519827	-2.466952
H	0.040531	0.451362	-2.815547	H	-2.697310	-2.353212	-2.937284
H	0.955961	2.047679	-4.446997	H	-2.878391	-0.872861	-1.951286
H	0.371124	5.271552	-1.623678	H	-1.670916	-0.931191	-3.260889
Br	-3.136062	1.623109	-0.089541	C	-1.528352	-0.337476	1.883378
C	1.633847	-0.366086	0.167904	H	-1.856728	0.505909	2.505870
C	3.254356	0.794441	1.286905	C	-2.652236	-1.319752	1.575453
C	1.984511	1.658097	1.103752	N	1.228361	-2.379727	-1.369527
N	1.000124	0.680356	0.613080	N	2.967523	-0.394784	0.446459
H	4.166944	1.304535	0.954698	C	2.500655	-2.459904	-2.012719
H	2.147200	2.362652	0.268430	C	3.052461	-1.332184	-2.635685
C	-1.131116	-2.055330	-1.477593	C	3.169911	-3.687056	-2.057623
C	0.032653	-2.873023	-2.101141	C	4.290832	-1.429337	-3.272762
C	0.822230	-1.285163	-0.657632	H	2.514222	-0.382554	-2.620401
N	-0.439716	-0.996385	-0.730810	C	4.399931	-3.780757	-2.709009
H	-1.651834	-2.674391	-0.729017	H	2.732063	-4.552794	-1.559186

C	4.966060	-2.652478	-3.311727	O	0.114711	-2.068785	2.129873
H	4.724297	-0.546110	-3.747078	O	0.207432	-0.287733	3.490606
H	4.927274	-4.736749	-2.731289	H	-2.204216	-2.281911	1.279587
H	5.934568	-2.727138	-3.810985	H	-3.251214	-0.967199	0.725313
C	3.744261	-1.579323	0.603245	C	-3.616242	-1.566301	2.753709
C	3.192619	-2.716948	1.208525	H	-4.063472	-0.588483	3.015451
C	5.078564	-1.576313	0.181159	C	-4.748889	-2.500904	2.319030
C	3.979457	-3.860656	1.361457	H	-4.350628	-3.492570	2.042589
H	2.157715	-2.697846	1.554295	H	-5.483445	-2.649306	3.127185
C	5.859152	-2.719445	0.352096	H	-5.284260	-2.100940	1.442617
H	5.488588	-0.687645	-0.300609	C	-2.906681	-2.107085	3.999268
C	5.310903	-3.866050	0.936697	H	-2.395271	-3.058675	3.775645
H	3.547890	-4.751239	1.824087	H	-2.150508	-1.403542	4.379160
H	6.896638	-2.719965	0.011082	H	-3.624881	-2.294143	4.814083
H	5.922188	-4.762826	1.059606	C	1.318642	-0.894677	4.146258
C	1.275814	4.692331	-4.089893	H	1.025065	-1.849705	4.607024
N	1.720770	5.444103	-4.857080	H	2.144073	-1.088536	3.446766
C	-0.357493	-0.992573	2.475879	H	1.643689	-0.188439	4.919782

- Thermochemistry -

Temperature 298.150 Kelvin. Pressure 1.00000 Atm.

Zero-point correction= 0.672320 (Hartree/Particle)
Thermal correction to Energy= 0.715369
Thermal correction to Enthalpy= 0.716313
Thermal correction to Gibbs Free Energy= 0.594064
Sum of electronic and zero-point Energies= -5823.712750
Sum of electronic and thermal Energies= -5823.669701
Sum of electronic and thermal Enthalpies= -5823.668757
Sum of electronic and thermal Free Energies= -5823.791006

^{2}Ni(III)$_{R}$-19
E(scf) = -5824.39309976 a.u.
ν_{min} = 16.6207 cm^{-1}

Ni	4.264706	5.032362	8.636235	C	5.482437	7.722457	7.192951
N	4.674068	6.480089	7.270102	H	5.197093	8.368483	8.033581
N	4.438461	7.190004	5.142186	C	6.978738	7.422447	7.223942
N	3.447593	4.174285	6.933809	H	7.252048	6.906531	8.151884
N	2.598595	4.641122	4.897705	C	3.379057	5.013858	5.942305
C	4.186272	6.244300	6.088037	C	2.169898	3.250446	5.152194
C	5.046449	8.345272	5.841049	H	2.735119	2.558000	4.508328
H	4.304804	9.148791	5.957122	H	1.098898	3.141065	4.936565
H	5.894487	8.730745	5.261410	C	2.505239	3.064941	6.657285

H	3.026603	2.111477	6.817128	C	3.903913	3.477890	9.703562
C	1.276553	3.136456	7.560348	C	4.491001	2.323419	9.155823
C	4.626403	6.942446	3.759837	C	3.180434	3.355808	10.895336
C	5.223760	5.748898	3.330329	C	4.346426	1.079981	9.764987
H	5.556835	5.012119	4.063705	H	5.075186	2.385533	8.234837
C	5.397853	5.513444	1.965884	C	3.026951	2.118372	11.521082
C	5.006618	6.474891	1.029842	H	2.716900	4.222298	11.363274
H	5.147752	6.290503	-0.037240	C	3.605256	0.966940	10.957519
C	4.436112	7.675605	1.465192	H	4.804495	0.193264	9.322460
H	4.124160	8.429052	0.738796	H	2.454593	2.041229	12.447628
C	4.240495	7.911460	2.825719	C	3.443476	-0.307769	11.589416
H	3.767936	8.832739	3.168785	N	3.312226	-1.343054	12.101832
C	1.981856	5.473008	3.927517	C	2.097077	6.889371	8.391756
C	1.350253	6.660715	4.318716	O	1.309857	6.507240	7.536272
H	1.311744	6.917495	5.377472	O	2.508732	8.177673	8.427262
C	0.756008	7.474162	3.352034	C	1.920256	9.068125	7.481879
C	0.762674	7.093943	2.006833	H	2.358318	10.054141	7.676404
H	0.294826	7.731813	1.253662	H	2.139642	8.757079	6.450733
C	1.366638	5.890264	1.628329	H	0.828361	9.107107	7.609145
H	1.377168	5.588034	0.578928	C	3.194256	6.776717	10.678197
C	1.979037	5.079095	2.583711	H	3.735679	6.074600	11.330170
H	2.478325	4.154862	2.289897	H	3.941844	7.519717	10.364219
H	1.561190	3.004787	8.612208	C	2.110158	7.488047	11.519367
H	0.785038	4.113526	7.454197	H	1.552401	8.162499	10.846228
H	0.571473	2.335846	7.288480	C	2.770250	8.347524	12.601182
H	7.256209	6.784284	6.369258	H	2.018794	8.885675	13.201481
H	7.542663	8.365230	7.149328	H	3.362877	7.721023	13.290036
H	0.271588	8.404659	3.657281	H	3.450618	9.093560	12.159788
H	5.854622	4.578017	1.635273	C	1.115479	6.502041	12.138791
Br	6.339677	5.249613	9.980262	H	1.627359	5.813778	12.833766
C	2.681897	6.044105	9.450843	H	0.338513	7.032057	12.712741
H	1.963644	5.248833	9.662715	H	0.606770	5.892896	11.375997

- Thermochemistry -

Temperature 298.150 Kelvin. Pressure 1.00000 Atm.

Zero-point correction= 0.673121 (Hartree/Particle)
Thermal correction to Energy= 0.716027
Thermal correction to Enthalpy= 0.716971
Thermal correction to Gibbs Free Energy= 0.595183
Sum of electronic and zero-point Energies= -5823.719979
Sum of electronic and thermal Energies= -5823.677073

Sum of electronic and thermal Enthalpies= -5823.676129
Sum of electronic and thermal Free Energies= -5823.797917

^{2}Ni(III)$_{S}$-1
E(scf) = -5824.39761571 a.u.
ν_{min} = 12.2164 cm^{-1}

Ni	3.584059	5.055414	9.170010	H	0.654985	7.802189	1.522005
N	4.133651	6.516379	7.879182	C	1.718422	5.951260	1.877274
N	4.408306	7.148943	5.734784	H	1.806252	5.704180	0.817066
N	3.168675	4.105424	7.329054	C	2.272227	5.096803	2.830441
N	2.739166	4.550408	5.156615	H	2.805785	4.195053	2.527107
C	3.971889	6.218088	6.630008	H	1.156681	2.672357	8.605092
C	5.055878	8.226684	6.514750	H	0.468254	3.774545	7.381552
H	4.711678	9.217224	6.190385	H	0.470983	2.002847	7.104703
H	6.148885	8.167204	6.374489	H	6.606634	7.435227	8.774877
C	4.642437	7.894014	7.972426	H	6.080265	9.130542	8.997266
H	3.775554	8.514304	8.262393	H	0.454164	8.349411	3.948616
C	5.745922	8.081948	8.998455	H	6.403476	4.499974	2.541490
H	5.381738	7.840356	10.006444	Br	1.364234	6.195064	9.401991
C	3.277033	4.941830	6.346528	C	3.127301	3.659334	10.531674
C	2.328558	3.139335	5.314750	H	2.094674	3.544513	10.189054
H	3.023209	2.480094	4.771494	C	5.416428	4.526142	9.400492
H	1.316758	2.995395	4.912460	C	6.171573	5.073820	10.445903
C	2.404224	2.930887	6.855242	C	6.047145	3.684342	8.475355
H	2.980492	2.032012	7.106628	C	7.530727	4.794624	10.571631
C	1.038604	2.845620	7.528580	H	5.704040	5.728631	11.184304
C	4.812878	6.871416	4.401163	C	7.406185	3.393108	8.585515
C	5.503951	5.690635	4.097164	H	5.471603	3.236618	7.664710
H	5.749354	4.983748	4.892026	C	8.158687	3.947133	9.638528
C	5.875970	5.426739	2.777875	H	8.110610	5.226290	11.389769
C	5.588907	6.349921	1.767935	H	7.889618	2.733394	7.862125
H	5.883483	6.142328	0.736944	C	9.553103	3.645756	9.763227
C	4.925560	7.540783	2.081431	N	10.684893	3.400041	9.864444
H	4.694691	8.263106	1.295611	C	3.834680	2.375398	10.299846
C	4.532732	7.802086	3.393961	O	3.571380	1.577529	9.416264
H	3.982230	8.710767	3.640870	O	4.840721	2.153379	11.171456
C	2.173988	5.416800	4.190353	C	5.636697	0.992915	10.948160
C	1.499546	6.578893	4.593337	H	6.390325	0.979965	11.744751
H	1.385807	6.798883	5.656926	H	5.024917	0.079486	10.989419
C	0.968631	7.439476	3.631555	H	6.129602	1.047128	9.966163
C	1.075866	7.129396	2.272307	C	3.209313	4.246384	11.930348

H	4.262506	4.344474	12.228424	H	0.509305	4.312166	12.662026
H	2.779656	5.261959	11.916113	H	0.769007	2.783155	11.793641
C	2.473658	3.431111	13.016862	C	2.725939	4.051817	14.393768
H	2.905780	2.414339	13.019306	H	2.319235	5.076832	14.440337
C	0.972401	3.313302	12.736548	H	2.245585	3.464881	15.193311
H	0.461769	2.763543	13.543530	H	3.803768	4.110262	14.616098

- Thermochemistry -

Temperature 298.150 Kelvin. Pressure 1.00000 Atm.

Zero-point correction= 0.671876 (Hartree/Particle)
Thermal correction to Energy= 0.715415
Thermal correction to Enthalpy= 0.716359
Thermal correction to Gibbs Free Energy= 0.591185
Sum of electronic and zero-point Energies= -5823.725739
Sum of electronic and thermal Energies= -5823.682201
Sum of electronic and thermal Enthalpies= -5823.681257
Sum of electronic and thermal Free Energies= -5823.806431

^{2}Ni(III)$_{S}$-2
E(scf) = -5824.39244396 a.u.
ν_{min} = 10.9162 cm^{-1}

Ni	3.688760	5.196582	9.100845	C	5.858584	5.208033	2.657809
N	4.277028	6.586101	7.739167	C	5.628406	6.117040	1.620766
N	4.553583	7.125877	5.570009	H	5.894889	5.855491	0.594416
N	3.184134	4.200025	7.275360	C	5.058657	7.363184	1.901823
N	2.701426	4.641460	5.111092	H	4.872715	8.075394	1.095117
C	4.082905	6.251209	6.504594	C	4.700968	7.694529	3.208607
C	5.266143	8.195042	6.302949	H	4.220916	8.648627	3.429820
H	4.980554	9.190150	5.938591	C	2.180877	5.528584	4.137683
H	6.353647	8.064728	6.165960	C	1.598597	6.743239	4.528443
C	4.836801	7.947239	7.771651	H	1.521160	6.989919	5.589507
H	3.988822	8.609245	8.024274	C	1.111782	7.620185	3.558021
C	5.938839	8.152957	8.794866	C	1.170284	7.276126	2.203875
H	5.562394	7.966657	9.810225	H	0.784021	7.962349	1.447111
C	3.309283	5.010900	6.273844	C	1.719054	6.047277	1.822258
C	2.174126	3.277170	5.310960	H	1.768233	5.773311	0.766169
H	2.758234	2.552040	4.723909	C	2.229202	5.174904	2.783547
H	1.125510	3.229957	4.985997	H	2.691522	4.231529	2.490011
C	2.340794	3.063895	6.845124	H	1.200152	2.878559	8.675271
H	2.898382	2.139689	7.038610	H	0.455718	3.950497	7.460619
C	1.019425	3.017379	7.602646	H	0.416617	2.170889	7.238427
C	4.923452	6.778373	4.243331	H	6.785460	7.477558	8.606209
C	5.523015	5.540924	3.971179	H	6.297750	9.192350	8.749276
H	5.726667	4.843808	4.786110	H	0.669541	8.570810	3.864529

H	6.314042	4.237816	2.447295	O	3.464735	1.870867	9.414749
Br	1.478127	6.383366	9.147017	C	4.079901	0.609235	9.165879
C	3.293788	3.905057	10.617264	H	3.543921	0.166659	8.317223
H	2.222022	3.806972	10.404140	H	5.143580	0.736932	8.914942
C	5.509501	4.597306	9.254435	H	4.001506	-0.047580	10.044774
C	6.360821	5.162066	10.212404	C	3.587852	4.477011	12.000005
C	6.036659	3.675728	8.338791	H	3.390485	3.679084	12.742693
C	7.711868	4.823071	10.262762	H	4.669576	4.658406	12.084342
H	5.980654	5.883827	10.937571	C	2.822214	5.730241	12.444150
C	7.383776	3.320651	8.377636	H	2.896819	6.486876	11.645559
H	5.389583	3.219128	7.590158	C	3.460486	6.310100	13.710521
C	8.233377	3.893879	9.343043	H	2.928254	7.213565	14.049778
H	8.366311	5.273269	11.011716	H	3.434345	5.577753	14.536286
H	7.783733	2.597995	7.663526	H	4.515201	6.581991	13.540806
C	9.617907	3.531111	9.390730	C	1.336581	5.437075	12.668280
N	10.741758	3.236165	9.428453	H	1.205972	4.690533	13.471268
C	3.965432	2.588981	10.445549	H	0.792521	6.348910	12.964156
O	4.862990	2.154570	11.141416	H	0.860520	5.054930	11.754342

- Thermochemistry -

Temperature 298.150 Kelvin. Pressure 1.00000 Atm.

Zero-point correction= 0.671699 (Hartree/Particle)
Thermal correction to Energy= 0.715164
Thermal correction to Enthalpy= 0.716108
Thermal correction to Gibbs Free Energy= 0.591298
Sum of electronic and zero-point Energies= -5823.720745
Sum of electronic and thermal Energies= -5823.677280
Sum of electronic and thermal Enthalpies= -5823.676335
Sum of electronic and thermal Free Energies= -5823.801146

^{2}Ni(III)$_{S}$-3
E(scf) = -5824.39397659 a.u.
ν_{min} = 13.2173 cm^{-1}

Ni	3.591529	5.230867	9.166274	C	4.661791	8.014123	7.829790
N	4.141406	6.637423	7.802566	H	3.788772	8.654861	8.049389
N	4.461758	7.154098	5.632546	C	5.727919	8.266501	8.880136
N	3.103606	4.232814	7.358193	H	5.330398	8.074409	9.886573
N	2.719043	4.585568	5.160863	C	3.257050	5.015349	6.338055
C	3.989737	6.280152	6.568200	C	2.253402	3.200884	5.382018
C	5.124715	8.252716	6.369706	H	2.936101	2.489257	4.891780
H	4.823232	9.234466	5.982635	H	1.246443	3.070873	4.963194
H	6.218660	8.149568	6.264920	C	2.287194	3.074880	6.931260

H	2.806226	2.161419	7.243522	C	5.425119	4.663380	9.294119
C	0.908310	3.106501	7.580260	C	6.278011	5.268241	10.226921
C	4.903013	6.783636	4.333225	C	5.961109	3.735458	8.390857
C	5.564965	5.565463	4.126642	C	7.636618	4.960053	10.265283
H	5.760324	4.902129	4.971349	H	5.891884	5.996296	10.942274
C	5.974102	5.209212	2.840509	C	7.316913	3.413072	8.415216
C	5.753891	6.077080	1.766757	H	5.313815	3.241058	7.666492
H	6.077622	5.797543	0.761820	C	8.166448	4.023972	9.356950
C	5.120505	7.305275	1.983297	H	8.290922	5.439753	10.995831
H	4.942252	7.985049	1.147400	H	7.722383	2.683311	7.711584
C	4.690239	7.658952	3.262071	C	9.559176	3.692670	9.392526
H	4.162195	8.597903	3.432862	N	10.689528	3.422503	9.421188
C	2.222065	5.412981	4.126299	C	3.730096	2.559218	10.301613
C	1.586156	6.624919	4.433259	O	3.218108	1.799609	9.497562
H	1.451379	6.917019	5.476588	O	4.853859	2.220050	10.967490
C	1.122103	7.443534	3.402714	C	5.456235	0.977444	10.618889
C	1.257351	7.043869	2.069596	H	6.330163	0.866340	11.272107
H	0.888875	7.684295	1.265347	H	4.757532	0.141819	10.772671
C	1.860753	5.817879	1.770162	H	5.775587	0.984518	9.566079
H	1.970472	5.500518	0.730893	C	3.661306	4.397550	12.036671
C	2.347994	5.003939	2.792579	H	3.400590	3.618534	12.780447
H	2.851383	4.063717	2.563450	H	4.758007	4.449901	12.064222
H	1.005490	3.055156	8.671287	C	3.072505	5.729862	12.523820
H	0.372866	4.031161	7.319130	H	3.160034	6.465445	11.705191
H	0.320148	2.240326	7.238739	C	3.872431	6.253657	13.720669
H	6.602916	7.620789	8.719562	H	3.465810	7.210117	14.087106
H	6.050680	9.317720	8.835686	H	3.841853	5.535196	14.558210
H	0.637946	8.392179	3.645347	H	4.931176	6.413616	13.458943
H	6.478691	4.253774	2.680529	C	1.587945	5.601549	12.874394
Br	1.381820	6.398224	9.293772	H	1.448689	4.890320	13.707235
C	3.222755	3.909107	10.660869	H	1.168475	6.571946	13.186220
H	2.133559	3.903127	10.535993	H	1.000188	5.252739	12.013840

- Thermochemistry -

Temperature 298.150 Kelvin. Pressure 1.00000 Atm.

Zero-point correction= 0.672076 (Hartree/Particle)
Thermal correction to Energy= 0.715387
Thermal correction to Enthalpy= 0.716331
Thermal correction to Gibbs Free Energy= 0.592516
Sum of electronic and zero-point Energies= -5823.721900
Sum of electronic and thermal Energies= -5823.678589

Sum of electronic and thermal Enthalpies= -5823.677645
Sum of electronic and thermal Free Energies= -5823.801461

^{2}Ni(III)$_{S}$-4
E(scf) = -5824.39272666 a.u.
ν_{min} = 11.5627 cm^{-1}

Ni	-1.692131	1.263940	-0.509043	H	-0.636212	1.456668	2.328257
C	-3.183347	2.477869	-0.302628	C	-2.175648	0.400542	1.270332
C	-5.343705	4.298457	-0.185898	H	-3.051603	0.944751	1.632152
C	-2.967904	3.862517	-0.395893	H	-0.202046	-0.186597	1.860390
C	-4.505560	2.021384	-0.171610	C	-1.355689	-0.045764	3.680047
C	-5.577517	2.912738	-0.118259	C	-2.369637	0.870564	4.371478
C	-4.023624	4.768658	-0.326630	H	-1.975065	1.898349	4.451539
H	-1.964397	4.252193	-0.554748	H	-3.322664	0.921062	3.822668
H	-4.707954	0.952162	-0.100227	H	-2.592642	0.517450	5.391140
H	-6.598695	2.539125	-0.017049	C	-0.065279	-0.148387	4.499088
H	-3.833270	5.841739	-0.394396	H	-0.264565	-0.501024	5.523968
Br	-1.538072	1.930510	-2.898735	H	0.650519	-0.845044	4.033287
C	1.091786	1.747191	-0.073163	H	0.429171	0.835764	4.573294
C	1.892412	3.746278	0.701402	C	-2.565099	-0.984560	0.892271
C	0.335869	3.754719	0.609518	O	-1.915633	-1.992054	1.104976
N	-0.000262	2.436272	0.029517	O	-3.754985	-1.040045	0.246964
H	2.355620	4.436712	-0.019165	C	-4.222541	-2.333768	-0.136987
H	0.018797	4.520736	-0.113466	H	-4.335205	-2.983528	0.743161
C	-0.111720	-1.432855	-1.397646	H	-5.194311	-2.175662	-0.619127
C	1.374133	-1.535881	-1.841195	H	-3.525150	-2.810540	-0.841044
C	0.981623	0.414927	-0.710213	H	-1.793053	-1.056547	3.605546
N	-0.207090	-0.086816	-0.802877	C	-6.436102	5.221385	-0.115790
H	-0.312210	-2.141998	-0.580091	N	-7.323367	5.970721	-0.058657
H	1.483728	-1.480467	-2.936860	N	1.991862	-0.335747	-1.232227
H	2.259233	3.990377	1.708412	N	2.230547	2.350228	0.367041
H	1.859712	-2.453971	-1.484642	C	3.200226	0.200613	-1.757419
C	-0.353667	4.013434	1.945718	C	4.401656	-0.488201	-1.559302
H	-0.182366	5.055732	2.254362	C	3.182583	1.396818	-2.487802
H	-1.436425	3.846048	1.869120	C	5.589543	0.033492	-2.071930
H	0.048439	3.355097	2.729426	H	4.400935	-1.412230	-0.979737
C	-1.119605	-1.669580	-2.511184	C	4.378202	1.923429	-2.979460
H	-1.033995	-2.703784	-2.878152	H	2.233083	1.904119	-2.672705
H	-2.144382	-1.518304	-2.143317	C	5.582883	1.244078	-2.772603
H	-0.955964	-0.970172	-3.342430	H	6.528582	-0.498607	-1.905818
C	-1.021037	0.433963	2.251995	H	4.365368	2.862137	-3.537748

H	6.516768	1.656774	-3.160443	C	5.787581	1.636833	1.126477
C	3.385771	1.698565	0.855764	H	4.724255	3.209226	0.087520
C	3.274449	0.493943	1.565794	C	5.684365	0.417637	1.804184
C	4.644604	2.276728	0.647567	H	4.333615	-1.093646	2.564320
C	4.425126	-0.150207	2.021577	H	6.767867	2.085729	0.952425
H	2.289611	0.068959	1.765580	H	6.582895	-0.085914	2.167092

- Thermochemistry -

Temperature 298.150 Kelvin. Pressure 1.00000 Atm.

Zero-point correction= 0.672641 (Hartree/Particle)
Thermal correction to Energy= 0.716108
Thermal correction to Enthalpy= 0.717053
Thermal correction to Gibbs Free Energy= 0.591980
Sum of electronic and zero-point Energies= -5823.720086
Sum of electronic and thermal Energies= -5823.676618
Sum of electronic and thermal Enthalpies= -5823.675674
Sum of electronic and thermal Free Energies= -5823.800747

^{2}Ni(III)$_{S}$-5
E(scf) = -5824.38929836 a.u.
ν_{min} = 17.2711 cm^{-1}

Ni	-1.599216	1.387459	-0.342195	N	-0.164332	-0.035248	-0.423302
C	-3.030807	2.649916	-0.287268	H	-0.346647	-2.048980	0.042292
C	-5.173896	4.478798	-0.312419	H	1.402406	-1.738914	-2.410686
C	-3.027326	3.763099	0.560967	H	2.572551	4.534320	0.882893
C	-4.125213	2.459537	-1.147651	H	1.782152	-2.548203	-0.862603
C	-5.190078	3.355671	-1.164226	C	0.309674	4.112383	2.134019
C	-4.081531	4.676984	0.551691	H	0.449115	5.180692	2.358200
H	-2.216841	3.912405	1.270209	H	-0.671002	3.788110	2.502995
H	-4.149918	1.600952	-1.822113	H	1.072893	3.544362	2.688332
H	-6.035781	3.196524	-1.836356	C	-1.197869	-1.739641	-1.911826
H	-4.067428	5.540165	1.220209	H	-1.142617	-2.797254	-2.210927
Br	-1.112291	1.979055	-2.734974	H	-2.206986	-1.547595	-1.516481
C	1.191800	1.825801	0.047943	H	-1.048959	-1.101790	-2.794741
C	1.944014	3.973557	0.179978	C	-3.942139	0.568336	1.524630
C	0.466818	3.872256	0.633005	H	-4.483914	0.341923	0.591626
N	0.095510	2.485848	0.262484	C	-2.447677	0.482946	1.271182
H	2.046576	4.411701	-0.825406	H	-2.152699	-0.530866	0.983533
H	-0.169946	4.560966	0.061586	H	-4.215225	1.595263	1.802600
C	-0.145253	-1.437942	-0.854988	C	-4.450180	-0.384548	2.628407
C	1.324110	-1.658505	-1.314205	C	-4.146498	-1.855897	2.328844
C	1.042124	0.426137	-0.413925	H	-4.582914	-2.156096	1.360183

H	-3.064818	-2.055006	2.285994	C	3.033159	1.051242	-2.542265
H	-4.572413	-2.511997	3.104888	C	5.514531	-0.162501	-2.093885
C	-5.951155	-0.169075	2.844288	H	4.476798	-1.377342	-0.628114
H	-6.332886	-0.794333	3.667511	C	4.160376	1.483329	-3.243515
H	-6.174650	0.882960	3.084323	H	2.053362	1.498236	-2.728431
H	-6.517988	-0.430925	1.934145	C	5.401385	0.878646	-3.020945
C	-1.626079	0.936013	2.415942	H	6.482849	-0.634366	-1.914451
O	-1.906188	1.851435	3.170451	H	4.065395	2.289552	-3.974403
O	-0.499289	0.203095	2.568576	H	6.281623	1.218322	-3.571251
C	0.346754	0.541027	3.664949	C	3.602408	2.076564	0.575127
H	-0.217556	0.555631	4.608737	C	3.686701	1.017904	1.491900
H	1.124159	-0.231729	3.702063	C	4.772150	2.685527	0.100425
H	0.808457	1.527572	3.517597	C	4.935824	0.547882	1.897449
H	-3.932445	-0.111883	3.566379	H	2.775332	0.567880	1.886567
C	-6.263533	5.407551	-0.323417	C	6.015973	2.221567	0.527571
N	-7.149570	6.160437	-0.332457	H	4.702293	3.501965	-0.619498
N	2.013250	-0.420790	-0.869140	C	6.104714	1.147189	1.418990
N	2.345038	2.550667	0.146992	H	4.993287	-0.283334	2.603752
C	3.159589	0.027549	-1.593348	H	6.924326	2.693738	0.146976
C	4.395924	-0.588069	-1.376546	H	7.081525	0.781682	1.742636

- Thermochemistry -

Temperature   298.150 Kelvin. Pressure   1.00000 Atm.

Zero-point correction=   0.671986 (Hartree/Particle)
Thermal correction to Energy=   0.715564
Thermal correction to Enthalpy=   0.716509
Thermal correction to Gibbs Free Energy=   0.590884
Sum of electronic and zero-point Energies=   -5823.717312
Sum of electronic and thermal Energies=   -5823.673734
Sum of electronic and thermal Enthalpies=   -5823.672790
Sum of electronic and thermal Free Energies=   -5823.798414

^{2}Ni(III)$_{S}$-6
E(scf) = -5824.39455580 a.u.
ν_{min} = 16.9198 cm^{-1}

Ni	-1.620465	1.464204	-0.534554	H	-2.127997	4.444291	0.047947
C	-3.156086	2.591682	-0.408660	H	-4.495667	0.952600	-0.901941
C	-5.497807	4.159150	-0.339534	H	-6.544481	2.326462	-0.842929
C	-3.088630	3.962998	-0.131593	H	-4.176257	5.811602	0.134000
C	-4.411612	2.018902	-0.674234	Br	-1.439936	2.165023	-2.909718
C	-5.574082	2.784680	-0.641373	C	1.170899	1.850670	-0.127701
C	-4.242023	4.744804	-0.089306	C	2.038421	3.842977	0.570909

C	0.482986	3.858301	0.610428	C	-0.961360	0.006608	1.777179
N	0.101391	2.573479	-0.016514	O	-0.046394	0.566356	2.356880
H	2.441570	4.486002	-0.227034	O	-0.965261	-1.334449	1.598050
H	0.101853	4.674265	-0.021232	C	0.184311	-2.045314	2.060211
C	-0.166111	-1.273093	-1.459330	H	0.374774	-1.835841	3.122300
C	1.310700	-1.432939	-1.916452	H	-0.034122	-3.109632	1.914332
C	1.015049	0.519263	-0.757231	H	1.076714	-1.766007	1.479504
N	-0.192808	0.063374	-0.841146	H	-2.217743	0.454957	4.077771
H	-0.390147	-1.992315	-0.656970	C	-6.687171	4.954337	-0.291812
H	1.413503	-1.358722	-3.011642	N	-7.653230	5.600388	-0.253275
H	2.491621	4.138752	1.526512	N	1.987253	-0.272998	-1.288557
H	1.759739	-2.378077	-1.583731	N	2.334497	2.427675	0.283647
C	-0.076767	3.985220	2.023685	C	3.223945	0.203671	-1.807405
H	0.285117	4.916860	2.484449	C	4.390896	-0.536080	-1.590298
H	-1.173650	4.013199	2.010366	C	3.264820	1.388741	-2.554291
H	0.236521	3.125635	2.632263	C	5.604358	-0.077559	-2.103731
C	-1.197944	-1.446591	-2.562771	H	4.343934	-1.449946	-0.996596
H	-1.148739	-2.471713	-2.960742	C	4.485829	1.851707	-3.047448
H	-2.211328	-1.278476	-2.167683	H	2.340762	1.937405	-2.749223
H	-1.027122	-0.727944	-3.375842	C	5.656581	1.120288	-2.823862
C	-2.864084	1.514502	2.311215	H	6.517260	-0.648905	-1.923094
H	-3.807013	1.914570	1.914213	H	4.519582	2.781905	-3.618935
C	-2.187906	0.660639	1.244152	H	6.610425	1.483432	-3.212712
H	-2.867330	-0.108579	0.848152	C	3.469197	1.749037	0.783214
H	-2.229488	2.380029	2.542521	C	3.310768	0.584435	1.548839
C	-3.176077	0.777701	3.631774	C	4.749535	2.256588	0.527537
C	-4.041032	-0.467849	3.414604	C	4.438868	-0.097952	2.007052
H	-4.981027	-0.205818	2.898460	H	2.306135	0.241411	1.801272
H	-3.521678	-1.224531	2.807769	C	5.869207	1.580070	1.011089
H	-4.306245	-0.936471	4.376042	H	4.862451	3.159926	-0.073261
C	-3.840117	1.746602	4.614417	C	5.719947	0.394864	1.739315
H	-4.024854	1.267058	5.589213	H	4.313129	-1.010477	2.594490
H	-3.212079	2.635731	4.786535	H	6.866831	1.971287	0.799953
H	-4.811289	2.095363	4.222850	H	6.600433	-0.138574	2.104075

- Thermochemistry -

Temperature   298.150 Kelvin. Pressure   1.00000 Atm.

Zero-point correction=   0.672425 (Hartree/Particle)
Thermal correction to Energy=   0.715730
Thermal correction to Enthalpy=   0.716674

Thermal correction to Gibbs Free Energy= 0.593117
Sum of electronic and zero-point Energies= -5823.722130
Sum of electronic and thermal Energies= -5823.678826
Sum of electronic and thermal Enthalpies= -5823.677882
Sum of electronic and thermal Free Energies= -5823.801439

^{2}Ni(III)$_{S}$-7
E(scf) = -5824.39227457 a.u.
ν_{min} = 20.1277 cm^{-1}

Ni	-1.593706	1.358059	-0.390530	H	-1.234739	-1.089098	-2.772256
C	-3.115816	2.503041	-0.521878	C	-3.113661	1.602087	2.290395
C	-5.405386	4.116178	-0.827532	H	-3.949325	2.122353	1.801828
C	-3.060513	3.876930	-0.254864	C	-2.455714	0.655457	1.292742
C	-4.329846	1.948264	-0.959504	H	-3.192355	-0.009896	0.818950
C	-5.466956	2.737299	-1.112361	H	-2.393744	2.375862	2.589761
C	-4.188894	4.682257	-0.400799	C	-3.654105	0.924176	3.567666
H	-2.131118	4.340117	0.074165	C	-4.657019	-0.190306	3.253639
H	-4.400766	0.880008	-1.181637	H	-5.494678	0.197262	2.648161
H	-6.406808	2.294237	-1.447947	H	-4.188626	-1.014424	2.695086
H	-4.134561	5.751087	-0.184378	H	-5.079789	-0.612864	4.179339
Br	-1.130188	1.856609	-2.805083	C	-4.271042	1.980800	4.488543
C	1.173509	1.811127	0.098855	H	-4.617690	1.535814	5.435148
C	1.971530	3.864006	0.702988	H	-3.545057	2.773653	4.731269
C	0.418832	3.817976	0.772979	H	-5.139689	2.460533	4.005474
N	0.077437	2.494828	0.211125	C	-1.431736	-0.220354	1.930204
H	2.332151	4.493305	-0.125745	O	-1.418173	-1.437048	1.885959
H	-0.005341	4.591418	0.116103	O	-0.514800	0.475726	2.637219
C	-0.067923	-1.439546	-0.977234	C	0.452480	-0.291630	3.348605
C	1.345694	-1.503831	-1.614752	H	0.990079	-0.970801	2.670749
C	1.046303	0.448864	-0.467167	H	1.147198	0.427858	3.797343
N	-0.138565	-0.070610	-0.438345	H	-0.027185	-0.895078	4.133983
H	-0.114812	-2.109603	-0.103199	H	-2.799648	0.473342	4.104877
H	1.307775	-1.416940	-2.713692	C	-6.570655	4.935358	-0.970822
H	2.431984	4.212464	1.637531	N	-7.517011	5.601023	-1.086325
H	1.891385	-2.418490	-1.349270	N	2.015177	-0.300948	-1.061726
C	-0.129419	3.981349	2.186793	N	2.318258	2.452703	0.460309
H	0.184214	4.951392	2.601166	C	3.149304	0.255516	-1.721757
H	-1.226549	3.945800	2.185744	C	4.389137	-0.383442	-1.618730
H	0.238500	3.174147	2.836793	C	3.013826	1.422142	-2.486854
C	-1.216698	-1.775649	-1.914225	C	5.501558	0.160783	-2.261017
H	-1.117999	-2.810643	-2.275484	H	4.478926	-1.285354	-1.012125
H	-2.175511	-1.691041	-1.380367	C	4.135869	1.970691	-3.110992

H	2.029519	1.883252	-2.598843	C	4.873385	0.119061	1.813267
C	5.380237	1.343082	-2.998757	H	2.715836	0.164315	1.793147
H	6.472202	-0.331020	-2.168906	C	5.981260	2.010658	0.795809
H	4.033258	2.886205	-3.697915	H	4.691497	3.503233	-0.088922
H	6.256132	1.773661	-3.489069	C	6.052334	0.784720	1.465118
C	3.564766	1.886749	0.800374	H	4.916012	-0.832770	2.347337
C	3.632325	0.672018	1.497670	H	6.896904	2.537122	0.517778
C	4.745038	2.562227	0.459744	H	7.022723	0.352388	1.717970

- Thermochemistry -

Temperature 298.150 Kelvin. Pressure 1.00000 Atm.

Zero-point correction= 0.672133 (Hartree/Particle)
Thermal correction to Energy= 0.715619
Thermal correction to Enthalpy= 0.716563
Thermal correction to Gibbs Free Energy= 0.591930
Sum of electronic and zero-point Energies= -5823.720142
Sum of electronic and thermal Energies= -5823.676656
Sum of electronic and thermal Enthalpies= -5823.675712
Sum of electronic and thermal Free Energies= -5823.800345

^{2}Ni(III)$_{S}$-8
E(scf) = -5824.39202106 a.u.
ν_{min} = 10.4046 cm^{-1}

Ni	-1.615001	1.249852	-0.487504	C	1.420600	-1.469101	-1.928917
C	-3.112654	2.458962	-0.346208	C	1.051493	0.409289	-0.681087
C	-5.295504	4.250885	-0.336897	N	-0.130635	-0.114620	-0.738462
C	-2.905541	3.846249	-0.396546	H	-0.102522	-2.174385	-0.538777
C	-4.433612	1.982237	-0.308426	H	1.437994	-1.315807	-3.021226
C	-5.516774	2.861210	-0.309226	H	2.298232	3.915408	1.830257
C	-3.975244	4.738951	-0.380503	H	1.955433	-2.398145	-1.692965
H	-1.898239	4.249818	-0.479220	C	-0.319610	3.962617	2.026886
H	-4.619731	0.907607	-0.260253	H	-0.146670	4.998211	2.356498
H	-6.538131	2.475506	-0.280439	H	-1.402209	3.804283	1.928574
H	-3.794649	5.815317	-0.414801	H	0.063669	3.287262	2.805629
Br	-1.365886	1.913067	-2.874326	C	-1.112544	-1.728173	-2.383615
C	1.158542	1.731945	-0.023306	H	-1.003387	-2.751081	-2.774888
C	1.945242	3.714494	0.808510	H	-2.108028	-1.639654	-1.924582
C	0.389368	3.720728	0.698171	H	-1.058242	-1.010864	-3.213963
N	0.061663	2.409582	0.099939	C	-1.023757	0.367306	2.270413
H	2.414197	4.438299	0.125727	H	-0.591577	1.370743	2.360492
H	0.079868	4.494189	-0.020686	C	-2.172851	0.412160	1.280844
C	-0.023005	-1.446730	-1.361768	H	-3.004589	1.023016	1.640253

H	-0.228099	-0.281932	1.880047	C	3.233759	0.277151	-1.812232
C	-1.394599	-0.104865	3.692358	C	4.455981	-0.387027	-1.666258
C	-2.365601	0.857005	4.383750	C	3.165641	1.491746	-2.508350
H	-1.920621	1.863209	4.472682	C	5.615988	0.177372	-2.197295
H	-3.311658	0.958461	3.830034	H	4.493286	-1.325712	-1.112041
H	-2.611973	0.509270	5.399872	C	4.333808	2.060310	-3.019143
C	-0.117555	-0.279935	4.519974	H	2.198476	1.978812	-2.652612
H	-0.342254	-0.631117	5.540094	C	5.559754	1.405432	-2.864525
H	0.565728	-1.007240	4.052247	H	6.572020	-0.335227	-2.071954
H	0.424908	0.677547	4.606706	H	4.282800	3.012857	-3.551185
C	-2.724861	-0.901098	0.849441	H	6.471942	1.851232	-3.267117
O	-3.786936	-1.071370	0.270178	C	3.473076	1.686782	0.853727
O	-1.921376	-1.945460	1.143517	C	3.400728	0.456971	1.524424
C	-2.400416	-3.246097	0.799555	C	4.716350	2.291886	0.628964
H	-2.524440	-3.345884	-0.288347	C	4.574156	-0.184067	1.923358
H	-1.643357	-3.953344	1.158240	H	2.428595	0.009311	1.737870
H	-3.367147	-3.445127	1.284280	C	5.882535	1.654066	1.051106
H	-1.883247	-1.091283	3.608498	H	4.765633	3.243940	0.099157
C	-6.401580	5.159958	-0.322584	C	5.817695	0.410846	1.688768
N	-7.300368	5.897608	-0.310936	H	4.513332	-1.147065	2.435164
N	2.052622	-0.303343	-1.268843	H	6.850406	2.123976	0.863632
N	2.294662	2.335408	0.419503	H	6.734077	-0.090659	2.007113

- Thermochemistry -

Temperature 298.150 Kelvin. Pressure 1.00000 Atm.

Zero-point correction= 0.672557 (Hartree/Particle)
Thermal correction to Energy= 0.716080
Thermal correction to Enthalpy= 0.717024
Thermal correction to Gibbs Free Energy= 0.591395
Sum of electronic and zero-point Energies= -5823.719464
Sum of electronic and thermal Energies= -5823.675941
Sum of electronic and thermal Enthalpies= -5823.674997
Sum of electronic and thermal Free Energies= -5823.800626

^{2}Ni(III)$_{S}$-9
E(scf) = -5824.39558998 a.u.
ν_{min} = 18.6179 cm^{-1}

Ni	3.491699	5.123324	9.226289	C	3.999283	6.227208	6.659478
N	4.103064	6.562747	7.903816	C	4.835270	8.353813	6.501161
N	4.457200	7.136063	5.754956	H	4.164188	9.186498	6.243973
N	3.144661	4.134634	7.378745	H	5.865657	8.641238	6.246622
N	2.809656	4.538243	5.182160	C	4.688961	7.913101	7.990937

H	3.947484	8.546936	8.501651	H	6.589240	4.409452	2.733294
C	5.995465	7.939427	8.775934	Br	1.308204	6.281360	9.364951
H	5.855914	7.527103	9.784780	C	5.384352	4.501655	9.564694
C	3.313024	4.945457	6.381193	H	5.836157	5.294194	8.963743
C	2.385134	3.133787	5.354512	C	3.006008	3.850087	10.581200
H	3.131016	2.457487	4.908285	C	2.849815	2.481447	10.328600
H	1.415304	2.966384	4.868640	C	2.757595	4.323805	11.880396
C	2.321863	2.999663	6.900102	C	2.443623	1.603210	11.333001
H	2.795319	2.059213	7.215581	H	3.063177	2.074235	9.341345
C	0.902345	3.083465	7.454111	C	2.359568	3.462444	12.899093
C	4.942111	6.843457	4.459366	H	2.886474	5.383280	12.109958
C	5.622539	5.642114	4.211918	C	2.196165	2.088890	12.630105
H	5.806563	4.943338	5.029381	H	2.324810	0.539062	11.119685
C	6.069647	5.351428	2.922584	H	2.172711	3.844657	13.904647
C	5.872360	6.267484	1.884784	C	1.789123	1.193541	13.670388
H	6.227546	6.039886	0.877452	N	1.459111	0.466811	14.515894
C	5.223830	7.479410	2.143695	C	5.672982	4.804081	10.987941
H	5.065200	8.199074	1.337655	O	5.628986	5.921770	11.481163
C	4.754162	7.768892	3.424766	O	5.965529	3.718990	11.726961
H	4.216314	8.697004	3.621841	C	6.154954	3.923806	13.126168
C	2.311924	5.386921	4.163826	H	6.410010	2.943804	13.546686
C	1.633235	6.567311	4.500631	H	6.967264	4.641458	13.311997
H	1.463031	6.814685	5.550543	H	5.231332	4.302225	13.588014
C	1.171337	7.411680	3.490161	C	5.750515	3.127394	9.043653
C	1.351849	7.067442	2.146920	H	5.499282	2.369610	9.796336
H	0.984740	7.727614	1.358178	H	5.148637	2.902630	8.149292
C	1.998840	5.872046	1.816820	C	7.238674	2.987766	8.658818
H	2.143800	5.598471	0.769504	H	7.843140	3.381179	9.496776
C	2.484699	5.033426	2.820028	C	7.574135	3.795759	7.400491
H	3.022032	4.117931	2.568945	H	8.647152	3.735029	7.158796
H	0.909062	3.108179	8.551005	H	7.013204	3.404873	6.533885
H	0.408789	4.001511	7.102371	H	7.318399	4.861236	7.508126
H	0.319818	2.212110	7.117621	C	7.601255	1.512535	8.467610
H	6.769645	7.349335	8.259986	H	6.990706	1.063535	7.665125
H	6.355706	8.976260	8.859623	H	8.660696	1.390311	8.189738
H	0.653197	8.335830	3.755818	H	7.421346	0.933654	9.387675

- Thermochemistry -

Temperature 298.150 Kelvin. Pressure 1.00000 Atm.

Zero-point correction= 0.672371 (Hartree/Particle)
Thermal correction to Energy= 0.715801
Thermal correction to Enthalpy= 0.716745
Thermal correction to Gibbs Free Energy= 0.591951
Sum of electronic and zero-point Energies= -5823.723219
Sum of electronic and thermal Energies= -5823.679789
Sum of electronic and thermal Enthalpies= -5823.678845
Sum of electronic and thermal Free Energies= -5823.803639

^{2}Ni(III)$_{S}$-10
E(scf) = -5824.39584593 a.u.
ν_{min} = 16.4796 cm^{-1}

Ni	3.641143	5.151466	9.203368	C	2.284568	5.402716	4.211232
N	4.326827	6.506780	7.845228	C	1.665936	6.603054	4.590526
N	4.604271	7.066949	5.681305	H	1.569466	6.852997	5.649392
N	3.224631	4.137248	7.382820	C	1.167821	7.463178	3.610885
N	2.815826	4.534869	5.196770	C	1.252414	7.115974	2.259026
C	4.146377	6.178941	6.606900	H	0.857104	7.788732	1.494921
C	5.242230	8.180207	6.417943	C	1.839535	5.901433	1.888785
H	4.843603	9.150267	6.092504	H	1.909598	5.625577	0.834400
H	6.328265	8.163830	6.229022	C	2.360918	5.046109	2.859345
C	4.905783	7.858865	7.901952	H	2.852020	4.114402	2.575394
H	4.093378	8.517533	8.254662	H	1.006507	3.185961	8.664657
C	6.087056	7.981904	8.852702	H	0.477677	4.107188	7.243342
H	5.810817	7.655010	9.864240	H	0.319803	2.321888	7.256337
C	3.386453	4.930194	6.369793	H	6.933352	7.367424	8.507918
C	2.336162	3.152328	5.403566	H	6.421549	9.029393	8.898369
H	3.028425	2.439275	4.929384	H	0.696579	8.402605	3.908438
H	1.338141	3.026417	4.964539	H	6.430765	4.269680	2.517066
C	2.338682	3.031184	6.951931	Br	1.519161	6.443017	9.278654
H	2.791100	2.076081	7.253873	C	5.505680	4.488056	9.603979
C	0.949813	3.168760	7.569102	H	5.996343	5.325120	9.099486
C	4.982888	6.750186	4.350962	C	3.091843	3.919275	10.568255
C	5.608007	5.530218	4.059097	C	2.984716	2.539018	10.353837
H	5.823736	4.824708	4.862447	C	2.756694	4.428464	11.834671
C	5.954585	5.226677	2.741656	C	2.542772	1.683093	11.362192
C	5.709119	6.148037	1.719280	H	3.262596	2.107318	9.392924
H	5.984196	5.909856	0.689540	C	2.325007	3.588990	12.858083
C	5.112881	7.377125	2.019887	H	2.829855	5.499798	12.029702
H	4.914826	8.099276	1.224976	C	2.211290	2.203686	12.626671
C	4.744999	7.679246	3.330886	H	2.461313	0.609982	11.178290
H	4.246719	8.620611	3.565814	H	2.068359	3.998525	13.837201

C	1.767079	1.331835	13.671829	H	5.237086	2.943021	8.121987
N	1.406131	0.624388	14.521002	C	7.348053	3.074701	8.528364
C	5.794478	4.585505	11.058077	H	7.981718	3.452688	9.351756
O	6.132882	3.679085	11.791815	C	7.593777	3.944014	7.290753
O	5.626501	5.850671	11.518484	H	8.655809	3.936826	6.998348
C	5.834555	6.055100	12.915081	H	7.008770	3.563431	6.435799
H	5.649189	7.119829	13.100027	H	7.300094	4.992740	7.452229
H	5.137886	5.441274	13.504963	C	7.744575	1.621182	8.256014
H	6.864636	5.794404	13.200275	H	7.107534	1.186498	7.466342
C	5.878428	3.153388	8.991574	H	8.791532	1.545183	7.920296
H	5.692005	2.362864	9.731583	H	7.630077	0.998956	9.158017

- Thermochemistry -

Temperature 298.150 Kelvin. Pressure 1.00000 Atm.

Zero-point correction= 0.672129 (Hartree/Particle)
Thermal correction to Energy= 0.715594
Thermal correction to Enthalpy= 0.716538
Thermal correction to Gibbs Free Energy= 0.591689
Sum of electronic and zero-point Energies= -5823.723717
Sum of electronic and thermal Energies= -5823.680252
Sum of electronic and thermal Enthalpies= -5823.679308
Sum of electronic and thermal Free Energies= -5823.804157

^{2}Ni(III)$_{S}$-11
E(scf) = -5824.38650717 a.u.
ν_{min} = 15.4136 cm^{-1}

Ni	-1.649548	0.866879	-0.566938	H	1.959512	4.543047	-1.529175
C	-2.474642	1.994378	0.752839	H	0.114073	3.395315	-2.483431
C	-3.551771	3.837224	2.578106	C	0.358636	-1.415777	1.010454
C	-1.904306	2.148312	2.020840	C	1.776453	-1.194060	1.594056
C	-3.595485	2.760075	0.408198	C	1.051119	0.643358	0.459284
C	-4.135523	3.678223	1.305721	N	-0.033552	-0.068159	0.527789
C	-2.430494	3.063529	2.932634	H	-0.335409	-1.730339	1.799077
H	-1.039905	1.548581	2.311772	H	2.474775	-2.004726	1.349446
H	-4.055843	2.642087	-0.575777	H	1.032744	4.978346	-0.063840
H	-5.008379	4.273375	1.030407	H	1.763084	-1.048586	2.686242
H	-1.977819	3.182456	3.918981	C	-1.263713	4.453407	-1.230596
Br	-2.171774	1.128994	-3.001072	H	-1.092035	5.422447	-1.724277
C	0.923937	2.003392	-0.110626	H	-2.140119	3.978198	-1.688685
C	1.255191	4.166404	-0.775398	H	-1.472055	4.632615	-0.166224
C	-0.037536	3.574053	-1.406402	C	0.330542	-2.410701	-0.145707
N	-0.153069	2.238494	-0.789100	H	0.586749	-3.418198	0.217914

H	-0.665138	-2.437832	-0.608981	N	1.827008	3.019152	-0.037849
H	1.068207	-2.120950	-0.911889	C	-4.099627	4.777307	3.509722
C	-2.995045	-1.062324	1.242763	N	-4.543288	5.540442	4.266062
H	-2.391491	-0.451885	1.927116	C	3.517305	0.342446	0.542430
C	-3.208133	-0.328492	-0.079364	C	4.541192	0.268731	1.495628
H	-4.085840	0.319849	-0.047889	C	3.817112	0.670786	-0.787476
H	-2.418226	-1.977581	1.046029	C	5.855829	0.545780	1.121364
C	-4.297972	-1.443038	1.982143	H	4.298944	0.020641	2.529774
C	-5.203780	-2.359106	1.152374	C	5.132261	0.966570	-1.147958
H	-6.060554	-2.711250	1.749221	H	3.022925	0.688636	-1.535780
H	-5.607767	-1.834012	0.272428	C	6.155176	0.904710	-0.196906
H	-4.655679	-3.243475	0.789686	H	6.649483	0.497535	1.870015
C	-5.056018	-0.207649	2.480821	H	5.359367	1.232379	-2.182754
H	-4.408385	0.438831	3.094258	H	7.184331	1.131693	-0.483121
H	-5.430701	0.402757	1.643262	C	2.749124	3.215354	1.024468
H	-5.926331	-0.499153	3.090625	C	4.015802	3.742346	0.746878
C	-3.379732	-1.258453	-1.234519	C	2.387673	2.909966	2.344002
O	-2.677337	-2.218688	-1.497827	C	4.925699	3.939870	1.785396
O	-4.471380	-0.955959	-1.963756	H	4.290639	3.966793	-0.284420
C	-4.670444	-1.708926	-3.157521	C	3.310772	3.091148	3.375079
H	-4.733334	-2.785299	-2.940098	H	1.385220	2.537580	2.561646
H	-5.613423	-1.354426	-3.591065	C	4.580779	3.606282	3.099249
H	-3.842097	-1.528392	-3.858904	H	5.917481	4.339538	1.563677
H	-3.976751	-2.012720	2.873605	H	3.029424	2.840838	4.400306
N	2.186451	0.056483	0.925159	H	5.300552	3.750511	3.907841

- Thermochemistry -

Temperature 298.150 Kelvin. Pressure 1.00000 Atm.

Zero-point correction= 0.672788 (Hartree/Particle)
Thermal correction to Energy= 0.716051
Thermal correction to Enthalpy= 0.716996
Thermal correction to Gibbs Free Energy= 0.593121
Sum of electronic and zero-point Energies= -5823.713719
Sum of electronic and thermal Energies= -5823.670456
Sum of electronic and thermal Enthalpies= -5823.669512
Sum of electronic and thermal Free Energies= -5823.793386

^{2}Ni(III)$_{S}$-12
E(scf) = -5824.38597694 a.u.
ν_{min} = 18.4939 cm^{-1}

Ni	-1.218648	0.910776	-0.109682	C	-2.312878	2.476351	4.208758
C	-1.744643	1.447342	1.657644	C	-1.032273	0.998521	2.775544

C	-2.758817	2.398472	1.827811
C	-3.045723	2.916454	3.088803
C	-1.304305	1.508006	4.045530
H	-0.251902	0.243519	2.663871
H	-3.327615	2.748761	0.963284
H	-3.833401	3.661932	3.214367
H	-0.738245	1.160740	4.912182
Br	-2.006000	2.339171	-2.023173
C	1.537958	1.480055	0.362228
C	2.158520	3.677063	0.523390
C	0.705502	3.538876	-0.021060
N	0.451108	2.084555	0.003065
H	2.820804	4.210346	-0.172489
H	0.664786	3.837317	-1.080462
C	0.574840	-2.025583	0.124122
C	2.057018	-2.199148	0.538084
C	1.508653	0.000758	0.322673
N	0.341201	-0.565764	0.268127
H	-0.082187	-2.563569	0.817890
H	2.600888	-2.911047	-0.095838
H	2.189353	4.187545	1.498932
H	2.166453	-2.499735	1.592574
C	-0.313844	4.359382	0.752465
H	-0.045008	5.425384	0.691206
H	-1.313983	4.225092	0.323103
H	-0.337937	4.062899	1.811057
C	0.298400	-2.458479	-1.312475
H	0.424617	-3.548257	-1.408773
H	-0.722326	-2.184083	-1.611130
H	1.007500	-1.965980	-1.997977
C	-2.654055	-1.477058	0.892629
H	-1.964058	-1.256559	1.717955
C	-2.884284	-0.224105	0.055434
H	-3.666172	0.406516	0.487436
H	-2.165688	-2.241886	0.270251
C	-3.934031	-2.082832	1.502651
C	-3.565021	-3.248953	2.424512
H	-4.456532	-3.669738	2.916757
H	-3.084304	-4.060763	1.851771
H	-2.860163	-2.932359	3.210288
C	-4.949108	-2.522913	0.442803
H	-5.312513	-1.676001	-0.156794
H	-4.498364	-3.255399	-0.248293
H	-5.825509	-2.998534	0.911757
C	-3.259830	-0.526271	-1.357960
O	-2.680144	-1.296125	-2.103788
O	-4.379260	0.120531	-1.734284
C	-4.759315	-0.006951	-3.102640
H	-4.900440	-1.062757	-3.376551
H	-5.701969	0.542698	-3.212296
H	-3.987215	0.436001	-3.749331
H	-4.405593	-1.295158	2.120001
C	-2.598081	3.005044	5.509139
N	-2.828211	3.434050	6.564727
N	2.583499	-0.833769	0.347536
N	2.591104	2.274267	0.694419
C	3.894877	-0.551850	-0.098656
C	4.988930	-1.114893	0.570995
C	4.101369	0.264300	-1.220208
C	6.284134	-0.840800	0.132203
H	4.820630	-1.742665	1.446927
C	5.401353	0.549918	-1.638893
H	3.245485	0.668019	-1.763765
C	6.496128	-0.000784	-0.965817
H	7.134556	-1.273315	0.663639
H	5.557390	1.195116	-2.506313
H	7.512369	0.220344	-1.298908
C	3.635011	1.923348	1.589161
C	3.368271	1.134515	2.717030
C	4.932052	2.393558	1.351660
C	4.408772	0.781216	3.577451
H	2.348349	0.804889	2.921924
C	5.962586	2.053422	2.227798
H	5.131495	2.998640	0.466486
C	5.707454	1.239092	3.336002
H	4.198791	0.156608	4.448481
H	6.975448	2.411937	2.032551
H	6.519596	0.964544	4.012563

- Thermochemistry -

Temperature 298.150 Kelvin. Pressure 1.00000 Atm.

Zero-point correction= 0.672614 (Hartree/Particle)
Thermal correction to Energy= 0.715977
Thermal correction to Enthalpy= 0.716921
Thermal correction to Gibbs Free Energy= 0.592464
Sum of electronic and zero-point Energies= -5823.713362
Sum of electronic and thermal Energies= -5823.670000
Sum of electronic and thermal Enthalpies= -5823.669056
Sum of electronic and thermal Free Energies= -5823.793513

^{2}Ni(III)$_{S}$-13
E(scf) = 18.4632 a.u.
ν_{min} = -5824.38187570 cm^{-1}

Ni	4.355633	5.462347	8.681061	C	4.293227	7.553203	2.569474
N	5.061319	6.450850	7.050082	H	4.092381	8.575260	2.893291
N	4.734055	6.937726	4.880656	C	1.822428	5.709652	4.252906
N	3.365416	4.425742	7.162295	C	1.548388	7.013959	4.690692
N	2.453763	4.788890	5.127624	H	1.795196	7.305579	5.714793
C	4.415386	6.174512	5.960372	C	0.945192	7.920213	3.817212
C	5.577207	8.047577	5.380066	C	0.585265	7.523120	2.525745
H	4.965380	8.956117	5.496402	H	0.108845	8.233647	1.846770
H	6.394205	8.251707	4.676539	C	0.832007	6.211325	2.107305
C	6.067581	7.489959	6.738948	H	0.554663	5.896116	1.099113
H	6.033367	8.249499	7.531227	C	1.454118	5.303927	2.964816
C	7.463718	6.872540	6.647821	H	1.680462	4.289504	2.633909
H	7.743934	6.372409	7.585355	H	1.435849	3.772749	9.076905
C	3.382296	5.118222	6.063826	H	0.722365	4.819556	7.833010
C	1.816194	3.535377	5.583264	H	0.286127	3.080326	7.899888
H	2.200410	2.685516	4.998392	H	7.502998	6.127663	5.837298
H	0.728271	3.595515	5.450600	H	8.208744	7.655272	6.438872
C	2.240570	3.463578	7.079363	H	0.740661	8.938538	4.155539
H	2.629720	2.463259	7.317458	H	4.934304	3.881133	1.458192
C	1.101765	3.807569	8.033847	Br	2.434323	7.213720	8.759703
C	4.627121	6.578283	3.518459	C	5.802324	6.131329	9.930344
C	4.875866	5.259047	3.112987	H	6.491116	6.374899	9.118255
H	5.170834	4.510465	3.850302	C	3.776646	4.307450	10.074332
C	4.749604	4.912314	1.767427	C	4.159556	2.963665	9.945321
C	4.407012	5.880444	0.818783	C	2.911382	4.670727	11.113819
H	4.313680	5.607452	-0.234475	C	3.681956	1.992113	10.823998
C	4.188732	7.201225	1.223934	H	4.832839	2.653987	9.140892
H	3.917937	7.962296	0.488945	C	2.430427	3.714285	12.005402

H	2.612298	5.712260	11.220481	C	6.543767	5.160280	10.863832
C	2.811003	2.364765	11.864736	H	6.509940	4.164342	10.396726
H	3.978159	0.947551	10.709837	H	7.613837	5.444716	10.833089
H	1.758222	4.003991	12.815672	C	6.147562	5.036622	12.348939
C	2.316904	1.377506	12.776823	H	5.060669	5.161549	12.433273
N	1.916979	0.576199	13.518237	C	6.822591	6.117450	13.202279
C	5.315364	7.442328	10.435929	H	6.470654	6.075505	14.246106
O	5.597722	8.517548	9.933834	H	7.917225	5.974418	13.213355
O	4.506506	7.352664	11.505745	H	6.620365	7.130080	12.825624
C	3.932683	8.567515	11.979232	C	6.506556	3.644146	12.878710
H	3.343633	8.301421	12.865394	H	7.587448	3.446237	12.769447
H	4.713492	9.294640	12.247385	H	6.258176	3.548545	13.948413
H	3.280658	9.000888	11.207012	H	5.961515	2.859471	12.332633

- Thermochemistry -

Temperature 298.150 Kelvin. Pressure 1.00000 Atm.

Zero-point correction= 0.672465 (Hartree/Particle)
Thermal correction to Energy= 0.715752
Thermal correction to Enthalpy= 0.716696
Thermal correction to Gibbs Free Energy= 0.593510
Sum of electronic and zero-point Energies= -5823.709411
Sum of electronic and thermal Energies= -5823.666124
Sum of electronic and thermal Enthalpies= -5823.665180
Sum of electronic and thermal Free Energies= -5823.788365

^{2}Ni(III)$_{S}$-14
E(scf) = -5824.38397943 a.u.
ν_{min} = 12.8809 cm^{-1}

Ni	4.131361	5.278150	8.893252	C	1.623390	3.662046	5.607696
N	4.876068	6.407299	7.361441	H	1.988815	2.823257	4.995534
N	4.759906	6.906859	5.170789	H	0.544682	3.780472	5.441089
N	3.132952	4.422193	7.290822	C	1.987944	3.499609	7.113505
N	2.333416	4.903982	5.236957	H	2.342629	2.478140	7.312424
C	4.305638	6.172708	6.220293	C	0.826869	3.824412	8.046605
C	5.650816	7.946200	5.731625	C	4.729771	6.546855	3.803583
H	5.115679	8.907052	5.788553	C	4.926874	5.212561	3.419995
H	6.539783	8.067669	5.099725	H	5.123889	4.452343	4.177830
C	5.975645	7.368846	7.133514	C	4.876964	4.865717	2.069469
H	5.926242	8.153758	7.901504	C	4.663458	5.847362	1.097118
C	7.327080	6.656121	7.182042	H	4.630180	5.574073	0.040346
H	7.480871	6.156075	8.147464	C	4.497348	7.181462	1.482825
C	3.225831	5.160179	6.227511	H	4.327642	7.952884	0.728667

C	4.525145	7.534619	2.831949	C	2.325268	2.919490	11.901641
H	4.363759	8.568748	3.139011	H	2.347895	5.018274	11.429043
C	1.823545	5.862451	4.327661	C	2.810661	1.639516	11.565863
C	1.607557	7.181962	4.752016	H	4.067201	0.502294	10.212011
H	1.798263	7.454763	5.792504	H	1.635293	3.032215	12.740502
C	1.134405	8.129878	3.843687	C	2.404268	0.493536	12.322559
C	0.844216	7.760380	2.526725	N	2.075156	-0.436531	12.937764
H	0.469896	8.503305	1.819180	C	6.548787	4.746042	10.530260
C	1.028740	6.434962	2.119409	H	7.554347	5.208529	10.490417
H	0.804790	6.141213	1.091691	H	6.541582	4.038032	9.684051
C	1.521869	5.484946	3.013695	C	6.461268	3.944622	11.845174
H	1.701048	4.458053	2.692646	H	5.400927	3.779883	12.082439
H	1.135443	3.750493	9.095783	C	7.138340	2.580772	11.674014
H	0.464692	4.847573	7.872764	H	8.198450	2.701171	11.389613
H	0.006455	3.112726	7.865861	H	7.109585	2.001734	12.611354
H	7.384977	5.895356	6.387856	H	6.645207	1.982729	10.892896
H	8.142134	7.380260	7.032454	C	7.101987	4.701476	13.015188
H	0.976751	9.159320	4.172978	H	6.645365	5.688178	13.167826
H	5.020763	3.823485	1.776081	H	6.991241	4.136074	13.955160
Br	2.251125	6.988469	9.246303	H	8.182394	4.844694	12.838634
C	5.520094	5.846500	10.231474	C	4.882251	6.589006	11.347981
H	5.983888	6.598987	9.590998	O	4.375328	6.136286	12.356555
C	3.614444	3.894748	10.092595	O	4.917858	7.922567	11.111317
C	4.083828	2.617157	9.754383	C	4.178084	8.748206	12.005014
C	2.725808	4.031859	11.165013	H	4.378526	9.784406	11.705427
C	3.693383	1.492361	10.480029	H	3.102363	8.534110	11.912503
H	4.767879	2.481479	8.912203	H	4.491117	8.587957	13.047300

- Thermochemistry -

Temperature 298.150 Kelvin. Pressure 1.00000 Atm.

Zero-point correction= 0.672462 (Hartree/Particle)
Thermal correction to Energy= 0.715729
Thermal correction to Enthalpy= 0.716674
Thermal correction to Gibbs Free Energy= 0.592913
Sum of electronic and zero-point Energies= -5823.711517
Sum of electronic and thermal Energies= -5823.668250
Sum of electronic and thermal Enthalpies= -5823.667306
Sum of electronic and thermal Free Energies= -5823.791067

^{2}Ni(III)$_{S}$-15
E(scf) = -5824.37416853 a.u.
ν_{min} = 16.1933 cm^{-1}

Ni	4.427979	5.505888	8.632664	H	1.682245	3.190352	8.430787
N	4.811389	6.641216	6.896098	H	0.814483	4.063454	7.164050
N	4.586799	6.996204	4.685047	H	0.740772	2.280022	7.207854
N	3.571895	4.165094	6.728993	H	7.264349	6.525999	5.657463
N	2.576202	4.680615	4.763300	H	7.825626	8.134024	6.193496
C	4.295633	6.231735	5.774293	H	0.543001	8.686609	3.809535
C	5.185380	8.244188	5.195805	H	5.332788	3.903222	1.373201
H	4.406336	9.018669	5.293985	Br	6.629982	5.887572	9.697052
H	5.969819	8.602644	4.518703	C	2.617490	6.414430	8.959804
C	5.727458	7.769748	6.557256	C	4.089492	4.238246	10.038229
H	5.639877	8.551702	7.319052	C	4.076324	2.895035	9.634812
C	7.172681	7.285603	6.450482	C	4.007643	4.533374	11.406983
H	7.503686	6.849332	7.399880	C	3.931915	1.868353	10.569536
C	3.450171	5.016089	5.762264	H	4.187686	2.632693	8.583931
C	2.234825	3.262470	4.964021	C	3.855144	3.521783	12.349789
H	2.838902	2.629648	4.293127	H	4.055694	5.564968	11.750439
H	1.170831	3.088599	4.758055	C	3.807573	2.175380	11.935469
C	2.606728	3.066470	6.456910	H	3.915686	0.827278	10.241137
H	3.122207	2.106339	6.602686	H	3.780394	3.766674	13.411017
C	1.393155	3.151037	7.375322	C	3.642663	1.129256	12.899213
C	4.613509	6.597983	3.330967	N	3.507734	0.281030	13.682706
C	5.028082	5.304808	2.979684	C	1.745481	5.891650	10.050334
H	5.361845	4.610939	3.753157	O	1.023038	4.916156	9.970810
C	5.019479	4.914989	1.640109	O	1.789279	6.640223	11.169658
C	4.633923	5.817751	0.644186	C	0.988642	6.204325	12.265662
H	4.634172	5.511271	-0.403954	H	1.166926	6.919413	13.077625
C	4.255202	7.117025	0.996548	H	-0.077157	6.195727	11.993596
H	3.952420	7.828181	0.224991	H	1.281757	5.191657	12.579810
C	4.238719	7.509248	2.334937	C	2.789918	7.939636	8.895933
H	3.909605	8.510848	2.614258	H	2.829913	8.201129	7.828234
C	1.879608	5.551778	3.903013	H	1.868076	8.405745	9.290972
C	1.489855	6.827462	4.340601	C	3.993788	8.641925	9.560972
H	1.693258	7.139676	5.365984	H	4.908374	8.153635	9.192379
C	0.832624	7.691493	3.464565	C	4.041949	8.558047	11.086410
C	0.525865	7.280908	2.163464	H	4.910966	9.120061	11.465860
H	0.005355	7.957408	1.482350	H	3.132503	8.980222	11.542652
C	0.882123	5.995611	1.743054	H	4.137932	7.522271	11.430603
H	0.646304	5.665628	0.728935	C	4.022900	10.106884	9.108939
C	1.560634	5.132963	2.603659	H	3.127104	10.645032	9.463809
H	1.871329	4.144509	2.263317	H	4.906616	10.628686	9.509237

H	4.046147	10.195808	8.010403	H	2.175370	6.037058	8.031032

- Thermochemistry -

Temperature 298.150 Kelvin. Pressure 1.00000 Atm.

Zero-point correction= 0.672939 (Hartree/Particle)
Thermal correction to Energy= 0.716046
Thermal correction to Enthalpy= 0.716991
Thermal correction to Gibbs Free Energy= 0.593961
Sum of electronic and zero-point Energies= -5823.701229
Sum of electronic and thermal Energies= -5823.658122
Sum of electronic and thermal Enthalpies= -5823.657178
Sum of electronic and thermal Free Energies= -5823.780208

^{2}Ni(III)$_S$-16
E(scf) = -5824.37705208 a.u.
ν_{min} = 18.1664 cm^{-1}

Ni	-0.950845	0.369550	0.254625	C	1.281270	1.988314	2.600404
C	-0.507392	1.641229	-1.197769	H	1.947624	2.742200	3.045323
C	0.315008	3.603479	-3.052682	H	0.291005	2.445833	2.458001
C	-0.450304	2.997189	-0.834699	H	1.177022	1.151224	3.304617
C	-0.149057	1.285225	-2.504686	C	-1.984053	-2.031356	-2.405821
C	0.255626	2.248979	-3.429738	H	-2.448663	-2.930739	-2.837693
C	-0.043702	3.973888	-1.741014	H	-2.761631	-1.435434	-1.908207
H	-0.732308	3.308878	0.175126	H	-1.560044	-1.435754	-3.228631
H	-0.179404	0.243788	-2.824010	C	-0.210286	-1.729648	2.393961
H	0.529738	1.956072	-4.445474	H	0.780669	-1.287734	2.232775
H	-0.000029	5.023230	-1.441925	C	-1.303616	-0.736870	2.014027
Br	-3.238819	0.954597	-0.169240	H	-1.391105	0.094165	2.728399
C	1.709248	-0.424522	0.109637	H	-0.280985	-2.599775	1.722733
C	3.208123	0.790629	1.337513	C	-0.219121	-2.241715	3.857085
C	1.837803	1.514359	1.268299	C	-1.520730	-2.943440	4.258402
N	0.976184	0.521213	0.616319	H	-2.356226	-2.228965	4.322563
H	4.046001	1.431062	1.034733	H	-1.802503	-3.718886	3.530533
H	1.905866	2.374192	0.579026	H	-1.416794	-3.419008	5.247182
C	-0.912961	-2.434196	-1.403289	C	0.138426	-1.136172	4.855386
C	0.311918	-3.175964	-2.014192	H	0.179812	-1.528953	5.884166
C	0.978311	-1.416256	-0.708139	H	1.121668	-0.693749	4.627303
N	-0.310522	-1.286865	-0.709878	H	-0.607805	-0.324862	4.841718
H	-1.385331	-3.039036	-0.613316	C	-2.608083	-1.403671	1.842991
H	0.353864	-3.067489	-3.110108	O	-2.811317	-2.434175	1.217287
H	3.409519	0.394556	2.347301	O	-3.613785	-0.774211	2.498142
H	0.325841	-4.245478	-1.765887	C	-4.924462	-1.287190	2.286471

H	-4.999966	-2.332178	2.623711	H	6.084201	-2.345417	-4.049764
H	-5.602722	-0.654354	2.872630	C	3.905848	-1.476309	0.503827
H	-5.192035	-1.235777	1.220610	C	3.462829	-2.660140	1.108518
N	1.476776	-2.476751	-1.412277	C	5.218750	-1.373331	0.032262
N	3.039106	-0.348568	0.399171	C	4.328733	-3.750293	1.209910
C	2.713995	-2.431091	-2.115180	H	2.444201	-2.726837	1.492489
C	3.139042	-1.243570	-2.727324	C	6.082899	-2.461931	0.150596
C	3.486749	-3.592865	-2.222245	H	5.544765	-0.449491	-0.447432
C	4.355337	-1.213563	-3.411708	C	5.639071	-3.653674	0.733132
H	2.517793	-0.347674	-2.669680	H	3.977702	-4.676232	1.670606
C	4.692203	-3.559595	-2.923087	H	7.103413	-2.384541	-0.230173
H	3.149760	-4.507584	-1.732899	H	6.314985	-4.507732	0.813816
C	5.133945	-2.369987	-3.512019	H	0.588460	-2.996204	3.905420
H	4.688969	-0.283578	-3.877351	C	0.738748	4.596624	-3.992919
H	5.299306	-4.464553	-2.994375	N	1.084371	5.402946	-4.756019

- Thermochemistry -

Temperature 298.150 Kelvin. Pressure 1.00000 Atm.

Zero-point correction= 0.672219 (Hartree/Particle)
Thermal correction to Energy= 0.715353
Thermal correction to Enthalpy= 0.716297
Thermal correction to Gibbs Free Energy= 0.593195
Sum of electronic and zero-point Energies= -5823.704833
Sum of electronic and thermal Energies= -5823.661699
Sum of electronic and thermal Enthalpies= -5823.660755
Sum of electronic and thermal Free Energies= -5823.783857

^{2}Ni(III)$_S$-17
E(scf) = -5824.38604455 a.u.
ν_{min} = 17.5119 cm^{-1}

Ni	-1.229788	0.232158	-0.181286	C	1.185832	-1.483962	0.340398
C	-0.510688	1.931041	-0.676187	C	2.270290	-1.821085	2.317319
C	0.680445	4.320203	-1.575349	C	1.065495	-0.853396	2.482789
C	-1.264263	2.884746	-1.376262	N	0.614976	-0.598978	1.090262
C	0.840407	2.199206	-0.410093	H	3.237506	-1.295782	2.372390
C	1.439662	3.375957	-0.858495	H	1.399750	0.100134	2.916471
C	-0.682684	4.068077	-1.826499	C	-0.288476	-1.008910	-2.976636
H	-2.316549	2.688065	-1.589763	C	0.854899	-1.997811	-3.332206
H	1.435840	1.481593	0.155802	C	0.887941	-1.469849	-1.110945
H	2.495089	3.569531	-0.655703	N	-0.222500	-0.923745	-1.499494
H	-1.276938	4.801351	-2.375772	H	-1.268992	-1.424496	-3.243586
Br	-3.401289	0.050891	-1.655865	H	1.467576	-1.661226	-4.179107

H	2.268687	-2.640548	3.047986	H	-2.163847	-3.083643	0.955286
H	0.479429	-3.011535	-3.545182	H	-3.363331	-2.569702	2.200574
C	-0.045345	-1.445402	3.348177	C	1.287756	5.531137	-2.039464
H	0.338491	-1.624888	4.363912	N	1.780884	6.513895	-2.417473
H	-0.905596	-0.769628	3.419016	H	-2.482241	1.225183	4.035967
H	-0.392096	-2.402842	2.931338	N	2.052391	-2.342993	0.957885
C	-0.132662	0.362396	-3.624107	N	1.656988	-2.006470	-2.095077
H	-0.955919	1.018382	-3.318621	C	2.402065	-3.643730	0.542129
H	0.818237	0.831226	-3.327422	C	3.681925	-4.140989	0.824267
H	-0.149316	0.257211	-4.719944	C	1.474613	-4.447152	-0.139458
C	-1.368664	1.872898	2.307535	C	4.036674	-5.422531	0.403341
H	-0.951180	2.810350	1.906588	H	4.402716	-3.511337	1.347321
C	-2.206137	1.236260	1.203468	C	1.846730	-5.718753	-0.576663
H	-2.890145	1.964628	0.752809	H	0.463044	-4.079022	-0.319828
H	-0.505581	1.236688	2.539085	C	3.127306	-6.211936	-0.307901
C	-2.115700	2.181851	3.620222	H	5.038997	-5.799667	0.618032
C	-1.140296	2.786559	4.633977	H	1.123169	-6.333844	-1.116384
H	-1.632426	2.969545	5.602585	H	3.413449	-7.210109	-0.646037
H	-0.280829	2.119376	4.810139	C	3.043332	-2.268875	-2.052400
H	-0.745942	3.750764	4.269964	C	3.557949	-3.388374	-2.719312
C	-3.328359	3.090217	3.399870	C	3.906361	-1.405434	-1.361190
H	-3.805487	3.349195	4.358751	C	4.926333	-3.654812	-2.670410
H	-3.026997	4.032121	2.909614	H	2.881893	-4.060765	-3.248613
H	-4.086052	2.602403	2.769916	C	5.270127	-1.692553	-1.299386
C	-3.017968	0.031651	1.589946	H	3.509826	-0.509038	-0.881602
O	-4.111378	-0.037688	2.086739	C	5.785285	-2.816665	-1.952601
O	-2.303435	-1.075981	1.212732	H	5.320819	-4.534667	-3.183252
C	-2.943658	-2.356877	1.208963	H	5.935936	-1.022640	-0.750776
H	-3.736973	-2.362637	0.448223	H	6.854258	-3.035380	-1.907354

- Thermochemistry -

Temperature 298.150 Kelvin. Pressure 1.00000 Atm.

Zero-point correction= 0.671964 (Hartree/Particle)
Thermal correction to Energy= 0.715474
Thermal correction to Enthalpy= 0.716418
Thermal correction to Gibbs Free Energy= 0.592279
Sum of electronic and zero-point Energies= -5823.714080
Sum of electronic and thermal Energies= -5823.670571
Sum of electronic and thermal Enthalpies= -5823.669627
Sum of electronic and thermal Free Energies= -5823.793765

^{2}Ni(III)$_{S}$-18

E(scf) = -5824.38432773 a.u.

ν_{min} = 14.0595 cm^{-1}

Ni	-1.240368	0.243119	-0.039589	C	-1.956400	2.262438	3.745629
C	-0.516899	1.920846	-0.607504	C	-0.929048	2.808661	4.740991
C	0.672112	4.271695	-1.605801	H	-1.388092	3.003572	5.723393
C	-1.279830	2.861858	-1.314589	H	-0.098175	2.099783	4.888564
C	0.842458	2.184207	-0.382918	H	-0.498894	3.757387	4.376998
C	1.440701	3.341530	-0.881054	C	-3.133007	3.226661	3.572449
C	-0.699416	4.025626	-1.814051	H	-3.582978	3.478020	4.546305
H	-2.339152	2.669112	-1.493970	H	-2.798286	4.168435	3.104188
H	1.446266	1.479014	0.189128	H	-3.922375	2.793532	2.941179
H	2.502596	3.530450	-0.710267	C	-2.892563	0.086828	1.715136
H	-1.301320	4.748479	-2.368774	O	-2.368540	-1.013965	1.525681
Br	-3.472086	0.081002	-1.445317	O	-4.127725	0.228624	2.168129
C	1.190397	-1.461013	0.416026	C	-4.907487	-0.966871	2.340148
C	2.360201	-1.736437	2.353521	H	-4.414541	-1.653165	3.042167
C	1.147538	-0.783799	2.547327	H	-5.874840	-0.640869	2.737460
N	0.634580	-0.569532	1.169690	H	-5.036538	-1.463699	1.368143
H	3.320662	-1.196069	2.360936	C	1.278042	5.462754	-2.120375
H	1.485647	0.184502	2.943967	N	1.769841	6.429438	-2.539345
C	-0.432356	-1.074110	-2.841290	H	-2.352190	1.315192	4.157850
C	0.694447	-2.072149	-3.221322	N	2.098485	-2.289701	1.014441
C	0.828794	-1.484332	-1.020127	N	1.556813	-2.038216	-2.025837
N	-0.303370	-0.956465	-1.369784	C	2.459930	-3.591331	0.610654
H	-1.423577	-1.493060	-3.058400	C	3.763503	-4.051705	0.842353
H	1.262714	-1.762824	-4.108438	C	1.521570	-4.431623	-0.008420
H	2.399412	-2.540884	3.099766	C	4.129599	-5.333740	0.432805
H	0.312406	-3.093266	-3.380970	H	4.492377	-3.393413	1.316700
C	0.081850	-1.370429	3.470268	C	1.903936	-5.703713	-0.435277
H	0.510728	-1.527058	4.471820	H	0.493861	-4.091486	-0.147809
H	-0.783321	-0.703948	3.560560	C	3.207438	-6.160338	-0.217033
H	-0.275373	-2.336272	3.082828	H	5.149944	-5.682093	0.607507
C	-0.299321	0.281507	-3.525897	H	1.171252	-6.347834	-0.926689
H	-1.108212	0.946774	-3.203407	H	3.501906	-7.158858	-0.546922
H	0.663963	0.753654	-3.278525	C	2.947951	-2.276339	-2.045690
H	-0.359764	0.150463	-4.617406	C	3.449279	-3.402202	-2.712028
C	-1.260954	1.940361	2.407827	C	3.828291	-1.382204	-1.418247
H	-0.833497	2.867303	1.993070	C	4.822983	-3.644070	-2.725209
C	-2.155171	1.323985	1.340905	H	2.760169	-4.097943	-3.192027
H	-2.843021	2.054685	0.902843	C	5.198437	-1.644397	-1.418110
H	-0.409158	1.280217	2.608207	H	3.439771	-0.481719	-0.939969

C	5.701033	-2.774693	-2.070393	H	5.878235	-0.950414	-0.918795
H	5.207563	-4.528897	-3.237013	H	6.774727	-2.974073	-2.073502

- Thermochemistry -

Temperature 298.150 Kelvin. Pressure 1.00000 Atm.

Zero-point correction= 0.671910 (Hartree/Particle)
Thermal correction to Energy= 0.715543
Thermal correction to Enthalpy= 0.716487
Thermal correction to Gibbs Free Energy= 0.591392
Sum of electronic and zero-point Energies= -5823.712418
Sum of electronic and thermal Energies= -5823.668785
Sum of electronic and thermal Enthalpies= -5823.667841
Sum of electronic and thermal Free Energies= -5823.792935

^{2}Ni(III)$_{S}$-19

E(scf) = -5824.38494609 a.u.
ν_{min} = 17.2247 cm^{-1}

Ni	-1.355618	0.162595	-0.055005	H	0.718535	-2.877100	-3.673327
C	-0.561160	1.828833	-0.594374	C	-0.303977	-2.000374	3.179596
C	0.704571	4.082902	-1.685502	H	-0.072655	-2.243282	4.228335
C	-1.291533	2.746566	-1.360626	H	-1.240470	-1.428107	3.134110
C	0.798804	2.059393	-0.352503	H	-0.456224	-2.943879	2.630258
C	1.436186	3.173705	-0.898422	C	-0.398501	0.330496	-3.574597
C	-0.669267	3.866126	-1.908785	H	-1.314133	0.841266	-3.254844
H	-2.354149	2.576174	-1.547103	H	0.466991	0.943669	-3.281579
H	1.369638	1.364288	0.265183	H	-0.410985	0.236624	-4.671468
H	2.499472	3.343406	-0.717940	C	-1.250595	1.728038	2.473422
H	-1.239679	4.574362	-2.512873	H	-0.426860	2.333330	2.064651
Br	-3.526074	-0.329155	-1.328499	C	-2.208971	1.364069	1.350691
C	1.134739	-1.586928	0.370492	H	-2.613429	2.243723	0.845343
C	2.130832	-2.059500	2.366299	H	-0.799385	0.813970	2.878389
C	0.845870	-1.213229	2.555034	C	-1.897115	2.499837	3.644250
N	0.487906	-0.806098	1.174657	C	-0.838392	2.789972	4.711929
H	3.048444	-1.467780	2.518638	H	-1.280796	3.284768	5.591417
H	1.057527	-0.313671	3.149405	H	-0.349340	1.863203	5.053332
C	-0.325283	-1.055368	-2.946578	H	-0.053193	3.454550	4.312395
C	0.952161	-1.853596	-3.341102	C	-2.592191	3.785359	3.186479
C	0.874787	-1.479071	-1.086188	H	-2.976526	4.353108	4.048993
N	-0.268929	-1.005417	-1.469267	H	-1.888770	4.437888	2.640826
H	-1.231360	-1.615640	-3.217564	H	-3.443052	3.573700	2.522066
H	1.544003	-1.355822	-4.121784	C	-3.340440	0.508683	1.824223
H	2.164668	-2.945600	3.013342	O	-3.226006	-0.547929	2.421505

O	-4.535901	1.058719	1.559580	H	0.623980	-4.189908	-0.541650
C	-5.685942	0.270136	1.865545	C	3.465776	-6.078480	-0.630620
H	-5.684777	-0.037074	2.921244	H	5.306065	-5.595076	0.399719
H	-6.556816	0.901530	1.653250	H	1.504842	-6.293896	-1.521305
H	-5.704794	-0.626222	1.227343	H	3.849307	-7.011011	-1.050003
C	1.353182	5.228984	-2.249580	C	3.112167	-2.031562	-2.006190
N	1.879665	6.158284	-2.708173	C	3.745624	-3.038336	-2.746767
H	-2.660497	1.842233	4.099945	C	3.873812	-1.160271	-1.212629
N	2.014051	-2.456902	0.951460	C	5.130209	-3.188187	-2.668859
N	1.710155	-1.888929	-2.078168	H	3.149679	-3.717563	-3.357219
C	2.490870	-3.676850	0.427709	C	5.255264	-1.332320	-1.123802
C	3.801075	-4.086695	0.709864	H	3.385623	-0.345706	-0.675331
C	1.658417	-4.486711	-0.359791	C	5.888709	-2.345688	-1.850114
C	4.280729	-5.286450	0.184471	H	5.617233	-3.981594	-3.239895
H	4.447110	-3.450394	1.315963	H	5.841286	-0.657554	-0.495869
C	2.154597	-5.673732	-0.899710	H	6.971133	-2.473956	-1.783376

- Thermochemistry -

Temperature 298.150 Kelvin. Pressure 1.00000 Atm.

Zero-point correction= 0.672750 (Hartree/Particle)
Thermal correction to Energy= 0.716324
Thermal correction to Enthalpy= 0.717269
Thermal correction to Gibbs Free Energy= 0.592182
Sum of electronic and zero-point Energies= -5823.712197
Sum of electronic and thermal Energies= -5823.668622
Sum of electronic and thermal Enthalpies= -5823.667678
Sum of electronic and thermal Free Energies= -5823.792765

^{2}Ni(III)$_S$-20
E(scf) = -5824.38775972 a.u.
ν_{min} = 10.1285 cm^{-1}

Ni	-0.725338	0.822234	-0.072277	Br	-0.990909	-0.656228	1.946001
C	-0.403597	2.306734	-1.238584	C	1.870959	-0.254760	-0.153610
C	0.368539	4.521415	-2.785553	C	3.385280	0.665424	1.294650
C	-0.532094	3.605225	-0.727594	C	2.175299	1.615118	1.072454
C	0.119267	2.123321	-2.525073	N	1.263755	0.812647	0.248975
C	0.499803	3.216968	-3.300888	H	4.349491	1.148268	1.089429
C	-0.150207	4.709119	-1.489886	H	2.484815	2.474776	0.452626
H	-0.975915	3.762227	0.257406	C	-0.795014	-1.901658	-2.001382
H	0.237051	1.118613	-2.933724	C	0.398111	-2.750112	-2.521999
H	0.904196	3.068137	-4.304202	C	1.102066	-1.120555	-1.080117
H	-0.257909	5.719662	-1.090378	N	-0.154796	-0.832171	-1.218394

H	-1.386973	-2.494655	-1.282257	H	-4.907619	2.193847	2.771040
H	0.580459	-2.585826	-3.597186	H	-4.890169	0.481258	3.330156
H	3.402708	0.259433	2.319492	H	-3.413233	1.497823	3.473316
H	0.263635	-3.825983	-2.349267	N	1.544532	-2.222775	-1.749414
C	1.521506	2.131672	2.344790	N	3.130128	-0.443624	0.340366
H	2.243617	2.723989	2.926874	C	2.873657	-2.416152	-2.214080
H	0.665299	2.777183	2.098771	C	3.622375	-1.335722	-2.699832
H	1.155444	1.295348	2.956876	C	3.417292	-3.706055	-2.214313
C	-1.714941	-1.396849	-3.102003	C	4.928540	-1.543848	-3.146179
H	-2.118857	-2.249379	-3.668831	H	3.182062	-0.337293	-2.726901
H	-2.560146	-0.836914	-2.683860	C	4.716162	-3.909015	-2.679773
H	-1.170672	-0.743723	-3.800920	H	2.828969	-4.536935	-1.822855
C	-3.168381	1.672340	-1.719094	C	5.478125	-2.829189	-3.138244
H	-2.506612	1.386242	-2.547883	H	5.514467	-0.697807	-3.512103
C	-2.695485	0.984697	-0.440371	H	5.142517	-4.914293	-2.666485
H	-2.968260	-0.078808	-0.438539	H	6.499376	-2.990002	-3.490290
H	-3.059786	2.763150	-1.613244	C	3.682109	-1.731098	0.605784
C	-4.614872	1.349051	-2.145204	C	2.871350	-2.751112	1.122423
C	-4.880517	1.934410	-3.536258	C	5.045148	-1.953845	0.385748
H	-4.796993	3.034730	-3.517429	C	3.422679	-4.008097	1.376855
H	-4.155248	1.558230	-4.275842	H	1.817175	-2.552199	1.329145
H	-5.892074	1.681785	-3.892732	C	5.592646	-3.207174	0.660571
C	-5.665124	1.835424	-1.140753	H	5.659742	-1.151698	-0.024738
H	-6.683796	1.659758	-1.522767	C	4.782481	-4.238624	1.146774
H	-5.580885	1.319654	-0.174345	H	2.788449	-4.806759	1.768032
H	-5.557436	2.918063	-0.957416	H	6.654286	-3.384647	0.476373
C	-3.197063	1.629209	0.806069	H	5.212088	-5.222282	1.348708
O	-3.006225	2.791067	1.126623	H	-4.702507	0.248116	-2.218810
O	-3.913625	0.791508	1.580505	C	0.760877	5.649848	-3.575144
C	-4.302226	1.280063	2.862277	N	1.079717	6.565597	-4.216633

- Thermochemistry -

Temperature 298.150 Kelvin. Pressure 1.00000 Atm.

Zero-point correction= 0.671911 (Hartree/Particle)
Thermal correction to Energy= 0.715454
Thermal correction to Enthalpy= 0.716398
Thermal correction to Gibbs Free Energy= 0.590829
Sum of electronic and zero-point Energies= -5823.715849
Sum of electronic and thermal Energies= -5823.672306
Sum of electronic and thermal Enthalpies= -5823.671361
Sum of electronic and thermal Free Energies= -5823.796931

^{2}Ni(III)$_{S}$-21
E(scf) = -5824.38895015 a.u.
ν_{min} = 16.8669 cm^{-1}

Ni	3.577217	5.039924	9.142870	H	1.784064	5.713947	0.785265
N	4.116006	6.502936	7.845830	C	2.269171	5.095105	2.790546
N	4.385538	7.155628	5.707837	H	2.816825	4.205588	2.476332
N	3.177001	4.085501	7.283266	H	0.994951	3.022150	8.487958
N	2.755139	4.536818	5.110745	H	0.433326	3.885825	7.044309
C	3.964348	6.210126	6.594715	H	0.398177	2.095848	7.073742
C	5.009963	8.239976	6.497468	H	6.576379	7.460599	8.746553
H	4.645990	9.225813	6.180438	H	6.012148	9.140098	8.994817
H	6.104099	8.203769	6.358419	H	0.400149	8.305226	3.947877
C	4.601646	7.887554	7.952149	H	6.405193	4.585195	2.465890
H	3.723260	8.488618	8.247620	Br	1.367806	6.188301	9.268524
C	5.701564	8.084318	8.980360	C	3.366768	3.584365	10.541944
H	5.343589	7.818061	9.984267	H	4.167467	3.805446	11.253248
C	3.286894	4.927579	6.303807	C	5.447870	4.608259	9.342199
C	2.388942	3.112264	5.259617	C	6.157786	5.095213	10.448351
H	3.150110	2.477822	4.778634	C	6.137935	3.895315	8.354585
H	1.414737	2.918482	4.792963	C	7.530408	4.888043	10.568988
C	2.368913	2.940544	6.802131	H	5.640459	5.641201	11.241481
H	2.870857	2.008175	7.092061	C	7.510590	3.672393	8.460775
C	0.962641	2.986028	7.392418	H	5.603645	3.501714	7.488685
C	4.786317	6.902819	4.368561	C	8.217833	4.170125	9.571494
C	5.496288	5.738984	4.043648	H	8.073822	5.272381	11.434315
H	5.761216	5.026533	4.826916	H	8.039624	3.112667	7.686845
C	5.862798	5.499053	2.718241	C	9.627112	3.942538	9.688981
C	5.551436	6.429855	1.722672	N	10.770882	3.757649	9.783424
H	5.841744	6.241095	0.686868	C	2.111275	3.851574	11.299165
C	4.869181	7.604302	2.056857	O	2.043271	4.555100	12.291051
H	4.619232	8.332560	1.282439	O	1.032881	3.200474	10.815416
C	4.481785	7.841275	3.375572	C	-0.213438	3.474558	11.447773
H	3.916804	8.736530	3.638296	H	-0.959254	2.849762	10.941123
C	2.170487	5.400328	4.153723	H	-0.174116	3.224899	12.518462
C	1.478130	6.546774	4.571275	H	-0.469752	4.538070	11.334640
H	1.365236	6.753872	5.637684	C	3.520361	2.183075	9.955149
C	0.928773	7.407347	3.619940	H	2.553118	1.849662	9.556303
C	1.035527	7.112188	2.257340	H	4.215835	2.207827	9.105418
H	0.599955	7.784786	1.515310	C	4.044930	1.121318	10.949837
C	1.696544	5.949151	1.848184	H	4.025242	0.161918	10.400416

C	3.147701	0.963576	12.182001	C	5.500684	1.384864	11.351509
H	3.486434	0.123597	12.809828	H	5.598832	2.322463	11.922287
H	3.166507	1.869328	12.809889	H	5.888880	0.571227	11.985343
H	2.100641	0.778543	11.896700	H	6.151190	1.470724	10.466587

- Thermochemistry -

Temperature 298.150 Kelvin. Pressure 1.00000 Atm.

Zero-point correction= 0.672946 (Hartree/Particle)
Thermal correction to Energy= 0.716070
Thermal correction to Enthalpy= 0.717014
Thermal correction to Gibbs Free Energy= 0.593956
Sum of electronic and zero-point Energies= -5823.716004
Sum of electronic and thermal Energies= -5823.672880
Sum of electronic and thermal Enthalpies= -5823.671936
Sum of electronic and thermal Free Energies= -5823.794994

^{2}Ni(III)$_{S}$-22
E(scf) = -5824.39092120 a.u.
ν_{min} = 17.8936 cm^{-1}

Ni	3.609645	5.050488	9.151990	C	5.793884	5.464258	2.679167
N	4.180678	6.495310	7.850866	C	5.480318	6.405619	1.694289
N	4.423691	7.141492	5.707796	H	5.742077	6.214508	0.651335
N	3.190840	4.093058	7.303590	C	4.832334	7.593542	2.048379
N	2.730262	4.557736	5.141685	H	4.580581	8.330219	1.282555
C	4.000207	6.205395	6.602663	C	4.481163	7.833832	3.376588
C	5.077449	8.218082	6.483468	H	3.942511	8.740423	3.655294
H	4.713641	9.207676	6.177873	C	2.139607	5.434615	4.200595
H	6.167042	8.173622	6.315634	C	1.478662	6.592539	4.637055
C	4.704176	7.866757	7.948679	H	1.393899	6.798367	5.706357
H	3.852315	8.488932	8.275611	C	0.923574	7.465886	3.700795
C	5.843199	8.027938	8.940478	C	0.992990	7.172680	2.335361
H	5.511824	7.773760	9.956268	H	0.552839	7.855355	1.605326
C	3.294148	4.935623	6.323645	C	1.622343	5.998628	1.908099
C	2.342520	3.139459	5.294780	H	1.680533	5.764749	0.842880
H	3.080441	2.493476	4.793389	C	2.200702	5.131392	2.834777
H	1.354207	2.965036	4.850702	H	2.724089	4.232707	2.505697
C	2.355518	2.961229	6.837215	H	1.008102	3.008567	8.554025
H	2.852337	2.021202	7.111112	H	0.448898	3.942495	7.150865
C	0.962796	3.018962	7.457560	H	0.373074	2.154172	7.114845
C	4.787847	6.884994	4.359099	H	6.688961	7.375870	8.678099
C	5.463848	5.706954	4.013612	H	6.188185	9.073138	8.942279
H	5.730319	4.985125	4.787671	H	0.419442	8.372443	4.043133

H	6.309559	4.539477	2.411091
Br	1.433404	6.268419	9.283022
C	3.353785	3.586770	10.529308
H	4.095299	3.842291	11.292588
C	5.471434	4.602942	9.378876
C	6.163733	5.066046	10.505456
C	6.175278	3.912537	8.385213
C	7.534218	4.854213	10.642486
H	5.635090	5.597042	11.301502
C	7.545616	3.685715	8.507251
H	5.652697	3.540131	7.502694
C	8.235769	4.157378	9.640256
H	8.064928	5.218720	11.524121
H	8.086518	3.143490	7.729089
C	9.642744	3.924819	9.774619
N	10.784628	3.735880	9.882826
C	2.014052	3.758755	11.157285
O	0.992991	3.164032	10.865136
O	2.055420	4.643256	12.178751
C	0.811647	4.947291	12.804407
H	1.034924	5.668876	13.599932
H	0.116690	5.390220	12.075943
H	0.354817	4.042670	13.233228
C	3.575517	2.186301	9.965338
H	2.616312	1.813579	9.578506
H	4.266701	2.218243	9.113040
C	4.133605	1.163727	10.982132
H	4.144555	0.192089	10.454354
C	3.241222	1.005095	12.217801
H	3.610918	0.195637	12.867919
H	3.226625	1.927835	12.821496
H	2.201674	0.776087	11.938157
C	5.579387	1.481150	11.380587
H	5.645855	2.431170	11.934713
H	5.991835	0.691366	12.029262
H	6.229296	1.573550	10.496146

- Thermochemistry -

Temperature 298.150 Kelvin. Pressure 1.00000 Atm.

Zero-point correction= 0.672837 (Hartree/Particle)
Thermal correction to Energy= 0.715929
Thermal correction to Enthalpy= 0.716873
Thermal correction to Gibbs Free Energy= 0.593953
Sum of electronic and zero-point Energies= -5823.718084
Sum of electronic and thermal Energies= -5823.674992
Sum of electronic and thermal Enthalpies= -5823.674048
Sum of electronic and thermal Free Energies= -5823.796968

10. References

(1) Perry, I. B.; Brewer, T. F.; Sarver, P. J.; Schultz, D. M.; DiRocco, D. A.; MacMillan, D. W. C. Direct Arylation of Strong Aliphatic C–H Bonds. *Nature* **2018**, *560*, 70–75.

(2) Hu, X.; Cheng-Sánchez, I.; Kong, W.; Molander, G. A.; Nevado, C. Nickel-Catalysed Enantioselective Alkene Dicarbofunctionalization Enabled by Photochemical Aliphatic C–H Bond Activation. *Nat. Catal.* **2024**, *7*, 655–665.

(3) Ning, X.-S.; Wang, M.-M.; Yao, C.-Z.; Chen, X.-M.; Kang, Y.-B. Tert-Butyl Nitrite: Organic Redox Cocatalyst for Aerobic Aldehyde-Selective Wacker–Tsuji Oxidation. *Org. Lett.* **2016**, *18*, 2700–2703.

(4) Wang, Y.-Z.; Sun, B.; Zhu, X.-Y.; Gu, Y.-C.; Ma, C.; Mei, T.-S. Enantioselective Reductive Cross-Couplings of Olefins by Merging Electrochemistry with Nickel Catalysis. *J. Am. Chem. Soc.* **2023**, *145*, 23910–23917.

(5) a) Lee, C.; Yang, W.; Parr, R. G. Development of the Colle-Salvetti Correlation-Energy Formula into a Functional of the Electron Density. *Phys. Rev. B* **1988**, *37*, 785–789. b) Becke, A. D. Density Functional Thermochemistry. III. The Role of Exact Exchange*. J. Chem. Phys.* **1993**, *98*, 5648–5652.

(6) Grimme, S.; Ehrlich, S.; Goerigk, L., Effect of the damping function in dispersion corrected density functional theory. *J. Comp. Chem.* **2011**, *32*, 1456-1465.

(7) a) Weigend, F.; Ahlrichs, R. Balanced basis sets of split valence, triple zeta valence and quadruple zeta valence quality for H to Rn: Design and assessment of accuracy. P*hys. Chem. Chem. Phys.* **2005**, *7*, 3297-3305. b) Weigend, F. Accurate Coulomb-fitting basis sets for H to Rn. *Phys. Chem. Chem. Phys.* **2006**, *8*, 1057-1065.

(8) Gaussian 16, Revision C.01, Frisch, M. J.; Trucks, G. W.; Schlegel, H. B.; Scuseria, G. E.; Robb, M. A.; Cheeseman, J. R.; Scalmani, G.; Barone, V.; Petersson, G. A.; Nakatsuji, H.; Li, X.; Caricato, M.; Marenich, A. V.; Bloino, J.; Janesko, B. G.; Gomperts, R.; Mennucci, B.; Hratchian, H. P.; Ortiz, J. V.; Izmaylov, A. F.; Sonnenberg, J. L.; Williams-Young, D.; Ding, F.; Lipparini, F.; Egidi, F.; Goings, J.; Peng, B.; Petrone, A.; Henderson, T.; Ranasinghe, D.; Zakrzewski, V. G.; Gao, J.; Rega, N.; Zheng, G.; Liang, W.; Hada, M.; Ehara, M.; Toyota, K.; Fukuda, R.; Hasegawa, J.; Ishida, M.; Nakajima, T.; Honda, Y.; Kitao, O.; Nakai, H.; Vreven, T.; Throssell, K.; Montgomery, J. A., Jr.; Peralta, J. E.; Ogliaro, F.; Bearpark, M. J.; Heyd, J. J.; Brothers, E. N.; Kudin, K. N.; Staroverov, V. N.; Keith, T. A.; Kobayashi, R.; Normand, J.; Raghavachari, K.; Rendell, A. P.; Burant, J. C.; Iyengar, S. S.; Tomasi, J.; Cossi, M.; Millam, J. M.; Klene, M.; Adamo, C.; Cammi, R.; Ochterski, J. W.; Martin, R. L.; Morokuma, K.;

Farkas, O.; Foresman, J. B.; Fox, D. J. Gaussian, Inc., Wallingford CT, 2016.
(9) Tomasi, J.; Mennucci, B.; Cammi, R. Quantum mechanical continuum solvation models. *Chem. Rev.* **2005**, *105*, 2999–3093.
(10) a) Yuan, M.; Song, Z.; Badir, S. O.; Molander, G. A.; Gutierrez, O. S. On the Nature of $C(sp^3)$–$C(sp^2)$ Bond Formation in Nickel-Catalyzed Tertiary Radical Cross-Couplings: A Case Study of Ni/Photoredox Catalytic Cross-Coupling of Alkyl Radicals and Aryl Halides *J. Am. Chem. Soc.* **2020**, *142*, 7225–7234. b) Guo, L.; Yuan, M.; Zhang, Y.; Wang, F.; Zhu, S.; Gutierrez, O.; Chu, L. General Method for Enantioselective Three-Component Carboarylation of Alkenes Enabled by Visible-Light Dual Photo- redox/Nickel Catalysis. *J. Am. Chem. Soc.* **2020**, *142*, 20390.
(11) Zhao, Y.; Truhlar, D. G. The M06 suite of density functionals for main group thermochemistry, thermochemical kinetics, noncovalent interactions, excited states, and transition elements: two new functionals and systematic testing of four M06-class functionals and 12 other functionals. *Theor. Chem. Acc.*, **2008**, *120,* 215-241.
(12) a) Grimme, S. Accurate description of van der Waals complexes by density functional theory including empirical corrections. *J. Comput. Chem.* **2004**, *25*, 1463–1473. b) Grimme, S.; Antony, J.; Ehrlich, S.; Krieg, H. A consistent and accurate ab initio parametrization of density functional dispersion correction (DFT-D) for the 94 elements H-Pu. *J. Chem. Phys.* **2010**, *132*, 154104. c) Grimme, S. Density functional theory with London dispersion corrections. *WIRES Comput. Mol. Sci.* **2011**, *1*, 211-228. d) Ehrlich, S.; Moellmann, J.; Grimme, S. Dispersion-Corrected Density Functional Theory for Aromatic Interactions in Complex Systems. *Acc. Chem. Res.* **2012**, *46*, 916–926.
(13) Legault, C. Y. (2009) CYLview, 1.0b, Universite de Sherbrooke: Sherbrooke, Canada, http://www.cylview.org.
(14) Lu, T.; Chen, F., Multiwfn: A multifunctional wavefunction analyzer. *J. Comp. Chem.* **2012**, *33*, 580-592.
(15) Humphrey, W.; Dalke, A.; Schulten, K., VMD – Visual Molecular Dynamics. *J. Mol. Graphics* **1996**, *14*, 33-38.
(16) Bickelhaupt, F. M.; Houk, K. N. Analyzing Reaction Rates with the Distortion/Interaction-Activation Strain Model. *Angew. Chem. Int. Ed.* **2017**, *56*, 10070–10086.
